# Transition metal-free phosphonocarboxylation of alkenes with carbon dioxide via visible-light photoredox catalysis

**DOI:** 10.1038/s41467-019-11528-8

**Published:** 2019-08-09

**Authors:** Qiang Fu, Zhi-Yu Bo, Jian-Heng Ye, Tao Ju, He Huang, Li-Li Liao, Da-Gang Yu

**Affiliations:** 1 Key Laboratory of Green Chemistry & Technology of Ministry of Education, College of Chemistry, Sichuan University, 610064 Chengdu, P. R. China; 2 School of Pharmacy, Southwest Medical University, 646000 Luzhou, P. R. China; 3 State Key Laboratory of Elemento-Organic Chemistry, Nankai University, 300071 Tianjin, P. R. China

**Keywords:** Homogeneous catalysis, Synthetic chemistry methodology, Photochemistry

## Abstract

Catalytic difunctionalization of alkenes has been an ideal strategy to generate structurally complex molecules with diverse substitution patterns. Although both phosphonyl and carboxyl groups are valuable functional groups, the simultaneous incorporation of them via catalytic difunctionalization of alkenes, ideally from abundant, inexpensive and easy-to-handle raw materials, has not been realized. Herein, we report the phosphonocarboxylation of alkenes with CO_2_ via visible-light photoredox catalysis. This strategy is sustainable, general and practical, providing facile access to important β-phosphono carboxylic acids, including structurally complex unnatural α-amino acids. Diverse alkenes, including enamides, styrenes, enolsilanes and acrylates, undergo such reactions efficiently under mild reaction conditions. Moreover, this method represents a rare example of redox-neutral difunctionalization of alkenes with H-P(O) compounds, including diaryl- and dialkyl- phosphine oxides and phosphites. Importantly, these transition-metal-free reactions also feature low catalyst loading, high regio- and chemo-selectivities, good functional group tolerance, easy scalability and potential for product derivatization.

## Abstract (web summary)

Phosphonyl and carboxyl groups are valuable functional groups, however their simultaneous incorporation via catalytic difunctionalization of alkenes has not been realized yet. Here the authors report the phosphonocarboxylation of alkenes with CO_2_ via visible-light photoredox catalysis.

## Introduction

**Fig. 1:**
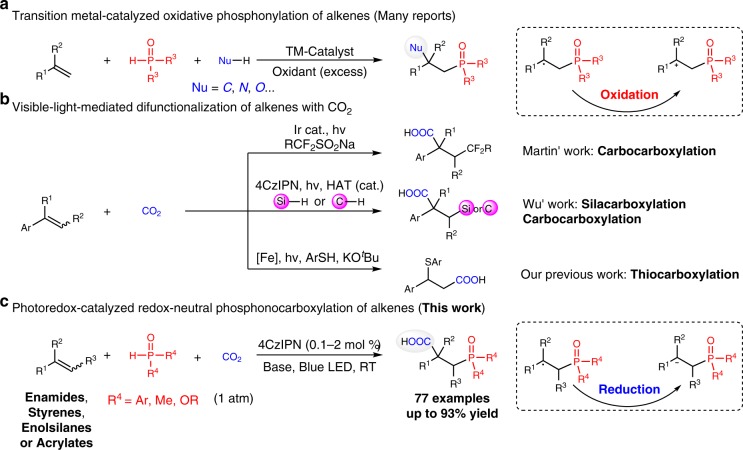
Catalytic difunctionalization of alkenes. **a** Oxidative difunctionalization of alkenes with H-P(O) compounds. **b** Limited examples for visible-light-mediated difunctionalization of alkenes with CO_2_ reported by Martin, Wu and our group. **c** Redox-neutral difunctionalization of alkenes with H-P(O) to generate important β-phosphono carboxylic acids, including structurally complex unnatural α-amino acids

Difunctionalization of alkenes has developed into a powerful tool in organic synthesis for generation of highly functionalized skeletons due to the easy availability of alkenes with different functional groups and diverse substitution modes^1–4^. Catalytic difunctionalization of alkenes with H-P(O) compounds is an important and ideal method to generate valuable organophosphine derivatives^5–11^, which are of great importance in agrochemicals^12^, functional materials^13,14^, synthetic^15^, and medicinal^16,17^ chemistry. Huge progress has been achieved in oxidative transformations (Fig. 1a)^18–21^, in which the electron-rich alkyl radicals, in situ generated through addition of phosphonyl radicals to alkenes, are oxidized to alkyl cations and then trapped by the nucleophilic components. However, the redox-neutral difunctionalization of alkenes with H-P(O) compounds remains very rare^22^.

Phosphorus-containing carboxylic acids are highly valuable compounds and widely exist in natural products^23^, materials^24^, and pharmaceuticals, which exhibit a diverse range of biological activities, acting as inhibitors of urease, glutamate carboxypeptidase II, neuropeptidase *N*-acetylated α-linked acidic dipeptidase (NAALADase), and so on^25–28^. Notably, the β-phosphono α-amino acids are important motifs in peptidic drugs, supramolecular catalysis (artificial metalloenzymes), and organic synthesis^29–31^. However, synthetic methods for such important compounds are extremely limited to de novo synthesis, which suffers from poor diversity, multiple steps, limited substrate scope, and/or harsh reaction conditions. We envisioned that the simultaneous incorporation of both phosphonyl and carboxyl groups via selective difunctionalization of enamides and other alkenes would serve as an ideal route to deliver important β-phosphono carboxylic acids, including β-phosphono α-amino acids. Different from the above mentioned oxidative functionalization of the generated alkyl radicals^18–21^, we hypothesized that reduction of such key alkyl radicals to anions, which might undergo nucleophilic attack to CO_2_, could realize redox-neutral difunctionalization of alkenes with H-P(O) compounds. To the best of our knowledge, this strategy has never been realized to generate such valuable targets.

Carbon dioxide (CO_2_) has been regarded as a ubiquitous, green and recyclable one carbon (C1) building block in organic synthesis^32–38^. Although the thermodynamic stability and kinetic inertness of CO_2_ introduces daunting challenges, a wide range of transformation using this gaseous reagent have been developed to construct important carboxylic acids, which are found in myriad natural products, agrochemicals, and pharmaceuticals^39^. Notably, catalytic carboxylation of unsaturated compounds with CO_2_ has attracted much attention of chemists^38,40–49^. Compared with widely investigated hydrocarboxylation of alkenes, however, catalytic difunctionalization of alkenes with CO_2_, which is obviously more attractive and cost-effective to obtain structurally complex molecules with diverse substitution patterns, is more challenging and much less investigated^38^. Although chemists have been mimicking Nature’s ability for long time to harness light in organic transformations^50–54^ and transform CO_2_ to value-added products^55,56^, the visible-light-mediated difunctionalization of alkenes with CO_2_ is still scarce and yet underdeveloped with limited examples reported by Martin, Wu and our group, independently (Fig. 1b)^57–59^. Moreover, photocatalytic difucnctionlization of the electron-rich alkenes, such as enamine and enol derivatives, with CO_2_ has not been reported yet, thus calling for a strategy to generate structurally more diverse α-amino acids^60–62^ and α-hydroxy acids^63^.

Herein, we report the catalytic phosphonocarboxylation of diverse alkenes, including enamides, styrenes, enolsilanes, and acrylates, with CO_2_ (Fig. 1c). This strategy is sustainable, general, and practical, representing a rare example of redox-neutral difunctionalization of alkenes with H-P(O) compounds to generate important β-phosphono carboxylic acids with high efficiency and selectivity under mild reaction conditions.

## Results

### Reaction design

**Fig. 2:**
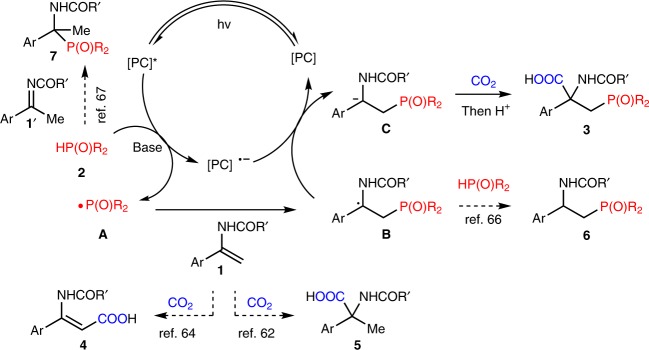
Proposed mechanism for selective phosphonocarboxylation of enamides **1** with **2** to give **3**. Compounds **4**, **5**, **6**, and **7** are possible byproducts. [PC] = photocatalyst

At the beginning of this project, we challenged ourselves with realization of selective phosphonocarboxylation of enamides with CO_2_ via visible-light photoredox catalysis to generate valuable β-phosphono α-amino acids. As proposed in Fig. 2, the phosphonyl radicals **A**, generated via single-electron transfer (SET) between H-P(O) compounds **2** and a photo-excited photocatalyst in the presence of a base, might undergo facile addition to the C = C double bonds of enamides **1** to selectively generate the α-amido radicals **B**, which was stabilized by phenyl and amide groups. A subsequent SET between **B** and the reduced photocatalyst might give rise to the α-amido carbanions **C**, which then could react with CO_2_ to deliver the desired β-phosphono α-amino acids **3**. However, the possible hydrocarboxylation^62^, C-H bond carboxylation^64^ of enamides as well as hydrophosphinylation (Pudovik reaction)^65^ via hydrogen atom transfer (HAT)^66^ between **B** and **2** could be competitive side reactions and generate **4**, **5**, and **6**, respectively. The tautomerization between enamides **1** with imines **1′** also should be considered, given that the latter species could be attacked by **2** to give α-amidophosphonate **7**^67^.

### Investigations of reaction conditions

We began our investigations using *N*-(1-phenylvinyl)benzamide **1a** and diphenylphosphine oxide **2a** as model substrates with atmospheric CO_2_ under visible light irradiation at room temperature (Table 1). To our delight, we detected the formation of β-phosphono α-amino acid **3aa** using Ru(bpy)_3_Cl_2_ as the catalyst and Cs_2_CO_3_ as the base, albeit in trace amounts (Table 1, entry 1). When we tested other photocatalysts, we found that an Ir-based photocatalyst significantly improved the efficiency for generation of **3aa** (70%, Table 1, entry 2) and the organic photocatalyst 1,2,3,5-tetrakis(carbazol-9-yl)-4,6-dicyanobenzene (4CzIPN) provided the best result (75%, Table 1, entry 3). Importantly, in the absence of such photocatalysts, **4a** (19%)^64^ and **7a** (41%)^67^ were generated instead of **3aa** (Table 1, entry 4). Further screening of various bases (Table 1, entries 5–7) showed that K_2_CO_3_ was the best choice (Table 1, entry 6), while the use of triethylamine as the base (Table 1, entry 7) would generate **5a** (29%)^62^ and **6a** (24%)^65,66^ along with **3aa** (32%). Intriguingly, the amount of photocatalyst could be even reduced to 0.1% without interfering the reaction (89%, Table 1, entry 8), illustrating the high efficiency of the reaction. Control experiments revealed that CO_2_, light, photocatalyst, and base were all crucial for this transformation (Table 1, entries 9–12).

**Table 1:** Screening the reaction conditions^a^


Entry	[PC]	Base	Yield (%)
1	Ru(bpy)_3_Cl_2_	Cs_2_CO_3_	<5
2	Ir[(ppy)_2_(dtbpy)]PF_6_	Cs_2_CO_3_	70
3	4CzIPN	Cs_2_CO_3_	75
4	—	Cs_2_CO_3_	N.D.
5	4CzIPN	Na_2_CO_3_	83
6	4CzIPN	K_2_CO_3_	89
7	4CzIPN	Et_3_N	32
8^b^	4CzIPN	K_2_CO_3_	89
9^b, c^	4CzIPN	K_2_CO_3_	<5
10	—	K_2_CO_3_	N.D.
11^b, d^	4CzIPN	K_2_CO_3_	N.D.
12^b^	4CzIPN	—	21

*LED* light-emitting diode, *DMF* *N*, *N*-dimethylformamide, *N.D.* not detected

^a^Reaction conditions: **1a** (0.2 mmol), **2a** (1.2 eq.), [PC] (2 mol%), Base (1.5 eq.), DMF (2 mL), RT. The yields are of isolated products

^b^0.1 mol% of [PC]

^c^Without CO_2_

^d^Without light

### Substrate scope of enamides

**Fig. 3:**
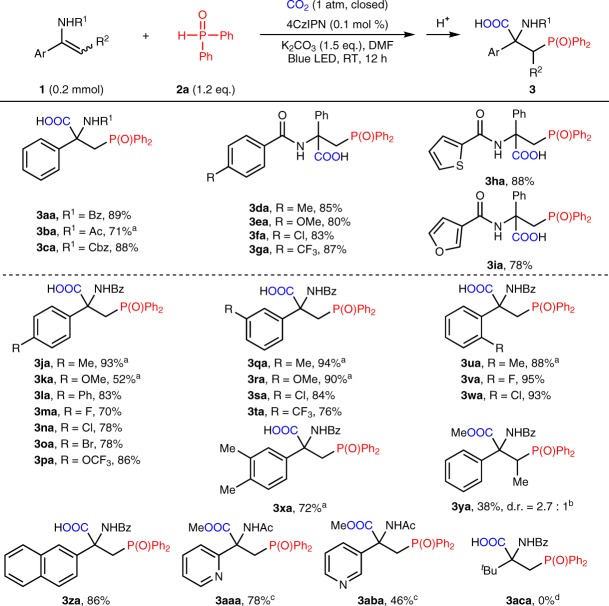
Substrate scope of enamides. ^a^4CzIPN (0.5 mol%) was used ^b^R^2^ = Me. ^c^Isolated as methyl ester by treating the reaction mixture with CH_3_I at 65 °C for 2 h. ^d^The hydrophosphinylation product **6ac** was obtained

With the acceptable reaction conditions in hand, a variety of β-phosphono α-amino acids bearing a quaternary carbon center were obtained in moderate to excellent yields. As illustrated in Fig. 3, a diverse array of protecting groups for the enamines (**3aa**-**3ca**), including the readily removed Ac (**3ba**) and Cbz (**3ca**) carbamate, proved to be compatible with the light-driven phosphocarboxylation reaction. Notably, the enamides bearing electron-donating (methoxyl, **3ea**) or electron-withdrawing groups (trifluoromethyl, **3ga**) as well as heteroarenes, such as thiophene (**3ha**) and furan (**3ia**), all reacted well. We next turned our attention to the substituents on arenes. As also shown in Fig. 3, a broad range of aryl enamides bearing different functional groups, including methyl (**3ja**), methoxyl (**3ka**), phenyl (**3la**), halogens (**3ma**-**3oa**), and trifluoromethoxyl (**3pa**) at the para-position, afforded the desired products in moderate to excellent yields. The enamides with meta- (**3qa**-**3ta**) and ortho-substituted arenes (**3ua**-**3wa**) also underwent such a transformation with high efficiency. The current protocol could also be applied to the substrates bearing di-substitution (**3xa**), internal enamides (**3ya**) and naphthalene (**3za**). Enamides containing pyridine (**3aaa** and **3aba**) could also be tolerated in the reaction. Unfortunately, when alkyl enamide (**1ac**) was used as substrate in the reaction, we did not detected the desired carboxylative product **3aca** while the hydrophosphinylation product **6ac** was obtained.

### Substrate scope of H-P(O) compounds

**Fig. 4:**
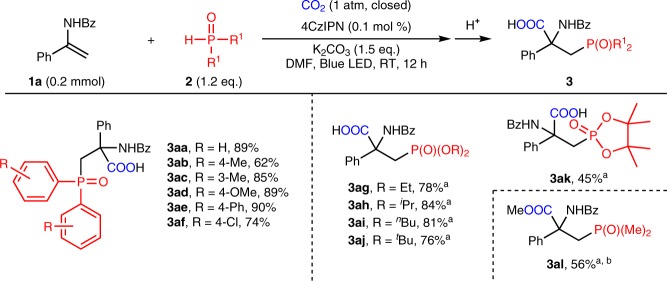
Substrate scope of phosphine oxides and phosphites. ^a^4CzIPN (2 mol%) and Cs_2_CO_3_ (1.5 eq.) were used. ^b^The yield for methyl ester of corresponding carboxylic acid is provided

Having demonstrated the good functional group compatibility in the enamide substrates, we next investigated the scope of the H-P(O) compounds **2**. As illustrated in Fig. 4, a broad range of phosphorus-containing α-amino acids were obtained. Both electron-donating (**3ab**-**3ad**) and mildly electron-withdrawing substituents (**3ae**-**3af**) on the aryl groups were well tolerated, leading to the desired products in good yields (62–90%). It is important to note that this transformation is not restricted to diarylphosphine oxides, various phosphites (**3ag**-**3ak**), which usually did not work well in the photochemical reactions, also could deliver the corresponding products smoothly, illustrating the synthetic utility and flexibility of this approach. Notably, dialkyl phosphine oxide, such as **3al**, also took part in the reaction to provide the desired product in moderate yield (56%), which demonstrates the generality of our transformation.

### Substrate scope of styrenes

**Fig. 5:**
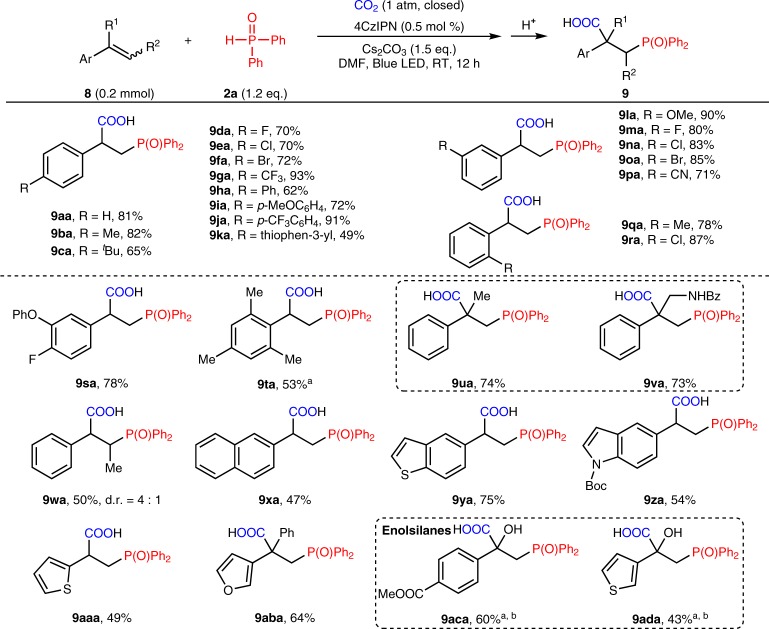
Substrate scope of styrenes. ^a^4CzIPN (2 mol%) was used ^b^Enolsilane was used as substrate, R^1^ = OTBS, TBS = tert-butyldimethylsilyl. Desilylation occurred upon acidifying the reaction mixture with aq. HCl (2 N), providing α-hydroxycarboxylic acid as the final product

Considering the importance of β-phosphono carboxylic acids^23^, we wondered whether styrenes **8** could be utilized in this phosphonocarboxylation process. To our delight, this protocol was easily applicable to a range of electronically diverse styrenes (Fig. 5). Diverse functional groups, including halogens (**9da**-**9fa**, **9ma**-**9oa**, **9ra**, **9ta**), trifluoromethyl (**9ga**), nitrile (**9pa**), and heteroarenes (**9ka**, **9va**) were tolerated well under the mild reaction conditions. The styrenes bearing disubstituted (**9sa**) and sterically hindered (2,4,6-trimethyl, **9ta**) benzenes also delivered the desired products smoothly. Notably, the reaction also worked well for α-methyl or aminomethyl substituted styrenes (**9ua**, **9va**), generating the products bearing quaternary carbon centers in good yields (74% and 73%, respectively). Other challenging alkenes, such as internal styrene (**9wa**) and heteroaryl-substituted styrenes (**9xa**-**9aba**), proved to be competent substrates in the reaction. Importantly, enolsilanes could also deliver the α-hydroxy acids (**9aca-9ada**) in moderate yields. In addition, we did not detect the desired product when alkyl olefin, such as dec-1-ene, was subjected to the reaction.

### Substrate scope of acrylates

**Fig. 6:**
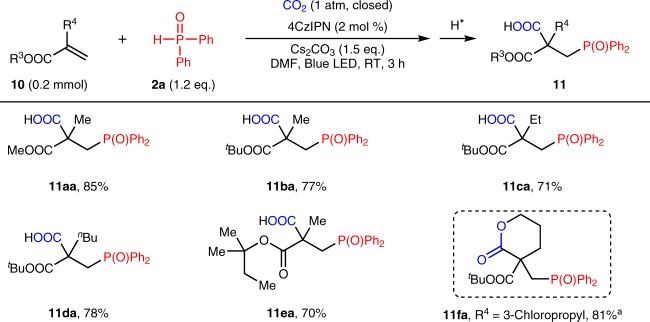
Substrate scope of acrylates. ^a^When 3-chloropropylacrylate **10f** was used as the substrate, a lactone **11fa** was obtained via cascade intramolecular cyclization

To further demonstrate the generality of the phosphonocarboxylation reaction, we also explored electron-deficient acrylates (Fig. 6). Although the hydrophosphinylation of electron-deficient alkenes with **2** via nucleophilic addition has been well documented^68^, we were pleased to find that a broad range of acrylates **10** smoothly underwent our photocatalyzed phosphonocarboxylation transformation with diphenylphosphine oxide **2a**. It is worth noting that when 3-chloropropylacrylate was used as substrate, a multi-substituted six-membered ring lactone **11fa** was obtained in good yield via intramolecular cyclization.

### Synthetic applications

**Fig. 7:**
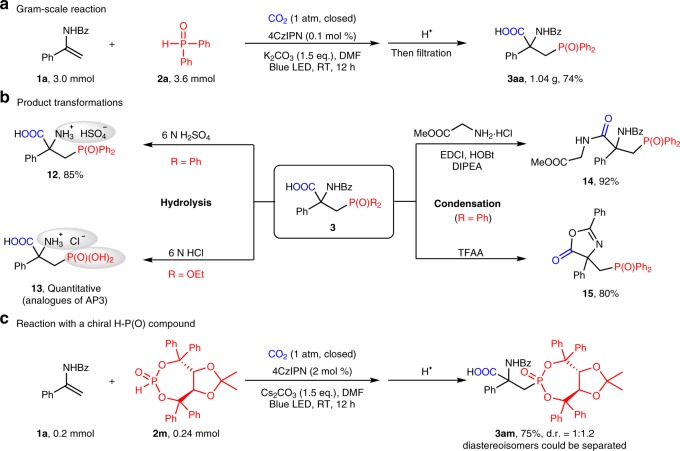
Synthetic applications of the method. **a** Gram-scale synthesis free of isolation with chromatography. **b** Transformations of the product via hydrolysis and condensation. **c** Reaction with a chiral H-P(O) compound. The diastereoisomers could be easily separated by column chromatography

To demonstrate the potential application of the protocol, a gram-scale reaction was carried out to afford **3aa** in 74% isolated yield without need of column chromatography (Fig. 7a). Moreover, the utility of the method was validated by facile derivatization of the products (Fig. 7b). For example, the benzoyl group could be removed easily to generate free β-phosphono α-amino acid **12** in good yield (85%). Hydrolysis of the benzamide and phosphite ester moieties in **3ag** (R = OEt) quantitatively generated **13**, an analogs of bioacitve AP3^69^. Importantly, amino acids condensation between **3aa** and methyl glycinate hydrochloride provided the phosphorus-containing dipeptide **14** (92%) and intramolecular condensation of **3aa** with the assistance of TFFA easily afforded cyclic oxazolinone **15**. Furthermore, since reactions of chiral phosphorus-centered radicals could proceed stereoselectively with retention of configuration^70^, a chiral H-P(O) compound derived from (4R, 5R)-Taddol derivative was used to achieve an enantioselective photocatalytic method with CO_2_ (Fig. 7c). Although the current protocol provides poor diasteroselectivity ratio, the diastereoisomers could be completely separated by column chromatography with good yields, which provides an alternative method for obtaining phosphonic acids-containing chiral α-amino acids upon hydrolysis (For more information regarding other types of chiral H-P(O) compounds, see Supplementary Figs. 2 and 3). The success of these experiments indicates the great potential application of the method in designing and synthesis of peptide drugs and ligands.

### Preliminary investigation of reaction mechanism

**Fig. 8:**
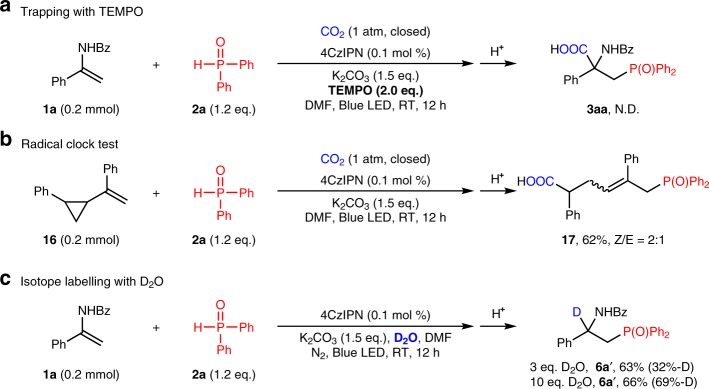
Preliminary mechanistic studies. **a** The reaction was totally suppressed when the radical scavenger 2,2,6,6-tetramethyl-piperidinyloxyl (TEMPO) was employed. **b** Ring-opening occurred when cyclopropane substituted styrene was used as the substrate. **c** When different amounts of deuterated water was added, different degrees of deuterated products were obtained

To gain more insight to the reaction mechanism, several control experiments were conducted. As illustrated in Fig. 8a, the reaction was suppressed when the radical scavenger 2,2,6,6-tetramethyl-piperidinyloxyl (TEMPO) was employed. Moreover, the radical clock test (Fig. 8b) also suggested that this transformation might rely on a radical process. Additionally, isotope-labeling studies provided a strong support for α-amino benzylic anionic species acted as key intermediates (Fig. 8c). Besides, Stern-Volmer luminescence studies demonstrated that the excited state of 4CzIPN was quenched by **2a** in the presence of base (see Supplementary Page 36) instead of **1a**. These results indicate the involvement of a reductive quenching photocatalytic cycle in the reaction.

## Discussion

In summary, we have described a general and practical strategy to realize the phosphonocarboxylation of alkenes with CO_2_ via visible-light photoredox catalysis. This method is suitable for diverse alkenes (enamides, styrenes, enolsilanes, and acrylates) and H-P(O) compounds (diaryl- and dialkyl- phosphine oxides and phosphites), all of which undergo such reactions efficiently to access important and potentially bioactive β-phosphono carboxylic acids, including β-phosphono α-amino acids. Notably, these redox-neutral and transition-metal-free reactions feature low catalyst loading, mild reaction conditions, high regio- and chemo-selectivities, good functional group tolerance, facile scalability, and easy product derivatization. Further application of this strategy is underway in our laboratory.

## Methods

### General procedure

An oven-dried Schlenk tube (10 mL) containing a stirring bar was charged with the substrates (0.2 mmol). The Schlenk tube was then introduced in a glovebox, where it was charged with H-P(O) compound (49 mg, 0.24 mmol, 1.2 eq.) and K_2_CO_3_ (41 mg, 0.3 mmol, 1.5 eq.). The tube was taken out of the glovebox and connected to a vacuum line where it was evacuated and back-filled with CO_2_ for 3 times. Then DMF (2 mL) and 4CzIPN (32 μL, 0.1 mol%, 5 mg dissolved in 1 mL DMF) were added under CO_2_ flow. Finally, the reaction mixture in sealed tube was placed at a distance of 2–3 cm from a 30 W blue LED and stirred at room temperature (25 °C) for 12 h. Then, the mixture was quenched with 4.5 mL of H_2_O and 0.5 mL of 2 N HCl (aq.), extracted with ethyl acetate (EA) for at least 5 times, then concentrated in vacuo. The residue was purified by silica gel flash chromatography (0.2% AcOH in CH_2_Cl_2_/MeOH 100/1 ~ 20/1) to give the pure desired product. Note: (1) for styrenes (0.5 mol% 4CzIPN and Cs_2_CO_3_ was used), flashed with petroleum ether/AcOEt 1/1 to 0.67% AcOH in petroleum ether/AcOEt 1/1; (2) For acrylates (2 mol% 4CzIPN and Cs_2_CO_3_ was used), flashed with petroleum ether/AcOEt 1/1 to 0.67% AcOH in petroleum ether/AcOEt 1/1; (3) for phosphites (2 mol% 4CzIPN and Cs_2_CO_3_ was used), before the addition of 0.5 mL 2N HCl (aq.), the quenched reaction mixture was extract three times for removing the inpurity, then 0.5 mL of 2N HCl (aq.) was added, the reaction mixture was extracted for 4 times, then the combined organic phase was concentrated in vacuum to obtain the pure product without chromatography.

## Supplementary information

Supplementary Information

## Data Availability

The authors declare that the data supporting the findings of this study are available within the article and its Supplemental Information files. Extra data are available from the author upon request. The crystallography data have been deposited at the Cambridge Crystallographic Data Center (CCDC) under accession number CCDC: 1885892 (methyl ester of **3aa**) and can be obtained free of charge from www.ccdc.cam.ac.uk/getstructures.
