# Supplementary material for: Transition metal-free phosphonocarboxylation of alkenes with carbon dioxide via visible-light photoredox catalysis
Source: Nat Commun. 2019 Aug 9;10:3592. doi: 10.1038/s41467-019-11528-8 (PMC6689110; doi:10.1038/s41467-019-11528-8)
Supplement: Supplementary file 1 — Supplementary Information [file 41467_2019_11528_MOESM1_ESM.pdf]

Supplementary Information

Transition metal-free phosphonocarboxylation of alkenes with carbon dioxide via visible-light photoredox catalysis

Fu et al.

Supplementary Methods

General Information

All reactions were set up with glovebox and carried out under carbon dioxide atmosphere in Schlenk tubes. Anhydrous solvent (including DMF, 99.8%, Water< 0.005%) were purchased from J&K, and used as received. Commercially available compounds were obtained from J&K, Accela, Adamas, Across, TCI and used as received unless otherwise stated.

^{1}H and ^{13}C NMR spectra were recorded on a Brüker Advance 400 spectrometer (^{1}H: 400 MHz, ^{13}C: 101 MHz, 31P: 162 MHz, ^{19}F: 376 MHz). Chemical shifts (δ) for ^{1}H and ^{13}C NMR spectra are given in ppm relative to TMS, The residual solvent signals were used as references for ^{1}H and ^{13}C NMR spectra and the chemical shifts converted to the TMS scale ($CDCl_3$: δH = 7.26 ppm, δC = 77.16 ppm; CD_2Cl_2: δH = 5.32 ppm, δC = 53.84 ppm; CD_3OD: δH = 3.31 ppm, δC = 49.00 ppm; DMSO-d_6: δH = 2.50 ppm, δC = 39.52 ppm). All ^{31}P spectra were measured using ^{1}H decoupling. The following abbreviations were used to explain the multiplicities: s = singlet, d = doublet, t = triplet, q = quartet, m = multiplet, b = broad.

UPLC yields were recorded on waters ACQUITY UPLC M-Class. TLC was performed using commercially prepared 100-400 mesh silica gel plates (GF254), and visualization was effected at 254 nm. Exact ESI mass spectra were recorded on a SHIMADZU LCMS-IT-TOF. ESI-MS were obtained on a Thermo-LTQ mass spectrometer.

Synthesis of Substrates

The substrates in **Table 2** were prepared according to procedures described in the literature reported before.[1-3]

The substrates (**2c - 2f**) in **Table 3** were prepared according to procedures described in the literature reported before.[4]

All the protocols were employed without any optimization of the reaction conditions.

Experimental Procedures and Characterization Data

An oven-dried Schlenk tube (10 mL) containing a stirring bar was charged with the substrates (0.2 mmol).[S1] The Schlenk tube was then introduced in a glovebox, where it was charged with H-P(O) compound[S2] (49 mg, 0.24 mmol, 1.2 eq.) and K_2CO_3 (41 mg, 0.3 mmol, 1.5 eq.). The tube was taken out of the glovebox and connected to a vacuum line where it was evacuated and back-filled with CO_2 for 3 times. Then DMF (2 mL) and 4CzIPN (32 μL, 0.1 mol%, 5 mg dissolved in 1 mL DMF) were added under CO_2 flow. Finally, the reaction mixture in sealed tube was placed at a distance of 2 ~ 3 cm from a 30 W blue LED and stirred at room temperature (25 ℃) for 12 h. Then, the mixture was quenched with 4.5 mL of H_2O and 0.5 mL of 2N HCl (aq.), extracted with ethyl acetate (EA) for at least 5 times, then concentrated in vacuo. The residue was purified by silica gel flash chromatography (0.2% AcOH in CH_2Cl_2/MeOH 100/1 ~ 20/1) to give the pure desired product. ***Note***: (1) for styrenes (0.5 mol% 4CzIPN and Cs_2CO_3 was used), flashed with petroleum ether/AcOEt 1/1 to 0.67% AcOH in petroleum ether/AcOEt 1/1; (2) For acrylates (2 mol% 4CzIPN and Cs_2CO_3 was used), flashed with petroleum ether/AcOEt 1/1 to 0.67% AcOH in petroleum ether/AcOEt 1/1; (3) for phosphites (2 mol% 4CzIPN and Cs_2CO_3 was used), before the addition of 0.5 mL 2N HCl (aq.), the quenched reaction mixture was extract three times for removing the inpurity, then 0.5 mL of 2N HCl (aq.) was added, the reaction mixture was extracted for 4 times, then the combined organic phase was concentrated in vacuum to obtain the pure product without chromatography.

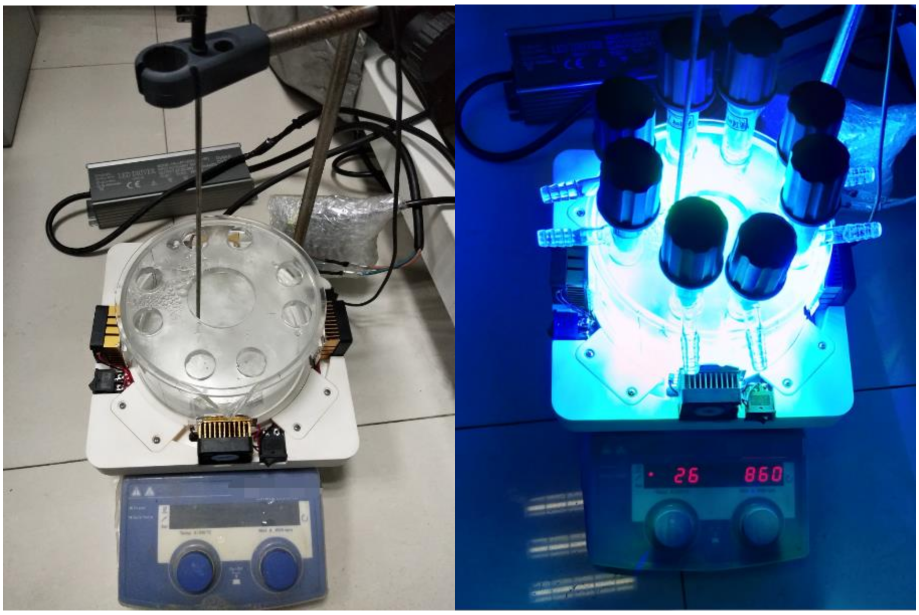

Supplementary Figure 1. Blue LED Photoreactor

2-benzamido-3-(diphenylphosphoryl)-2-phenylpropanoic acid (3aa)

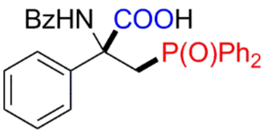

83.6 mg, 0.178 mmol, 89%;

White solid;

Mp: 196 - 197 ℃;

^{1}H NMR (400 MHz, DMSO-d_6) δ 13.36 (s, 1H), 8.48 (s, 1H), 7.90 – 7.79 (m, 2H), 7.68 – 7.59 (m, 2H), 7.58 – 7.43 (m, 8H), 7.42 – 7.35 (m, 2H), 7.29 – 7.13 (m, 6H), 4.16 (dd, *J* = 15.0, 6.6 Hz, 1H), 3.68 – 3.58 (m, 1H).

^{13}C NMR (101 MHz, DMSO-d_6) δ 172.85 (d, *J* = 5.6 Hz), 164.94, 140.16 (d, *J* = 9.1 Hz), 135.72 (d, *J* = 98.9 Hz), 133.90, 133.17 (d, *J* = 99.0 Hz), 132.04 (d, *J* = 2.6 Hz), 131.88, 131.29 (d, *J* = 2.9 Hz), 130.78 (d, *J* = 9.5 Hz), 130.40 (d, *J* = 9.3 Hz), 129.13 (d, *J* = 11.5 Hz), 128.65, 128.36 (d, *J* = 11.8 Hz), 128.34, 127.70, 127.01, 126.44, 62.58 (d, *J* = 5.0 Hz), 34.10 (d, *J* = 70.9 Hz).

^{31}P NMR (162 MHz, DMSO-d_6) δ 26.92.

HRMS (ESI+): calculated m/z $[M+H]^+$ for $[C_{28}H_{24}NO_4PH]^+$: 470.1516, found: 470.1513.

2-acetamido-3-(diphenylphosphoryl)-2-phenylpropanoic acid (3ba)

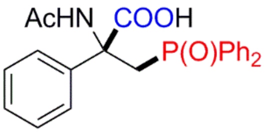

57.8 mg, 0.142 mmol, 71%;

White solid;

Mp: 204 - 205 ℃;

^{1}H NMR (400 MHz, DMSO-d_6) δ 13.19 (s, 1H), 8.02 (s, 1H), 7.88 – 7.72 (m, 4H), 7.58 – 7.41 (m, 8H), 7.30 – 7.16 (m, 3H), 4.06 – 3.94 (m, 1H), 3.71 – 3.55 (m, 1H), 1.19 (s, 3H).

^{13}C NMR (101 MHz, DMSO-d_6) δ 172.75 (d, *J* = 4.5 Hz), 169.17, 141.29 (d, *J* = 9.7 Hz), 136.08 (d, *J* = 98.3 Hz), 134.20 (d, *J* = 98.6 Hz), 131.82 (d, *J* = 2.3 Hz), 131.41 (d, *J* = 2.1 Hz), 131.11 (d, *J* = 9.3 Hz), 130.49 (d, *J* = 9.2 Hz), 129.05 (d, *J* = 11.3 Hz), 128.56 (d, *J* = 11.5 Hz), 128.32, 127.46, 126.17, 62.11 (d, *J* = 5.0 Hz), 33.14 (d, *J* = 72.7 Hz), 22.52.

^{31}P NMR (162 MHz, DMSO-d_6) δ 25.02.

HRMS (ESI+): calculated m/z $[M+H]^+$ for $[C_{23}H_{22}NO_4PH]^+$: 408.1359, found: 408.1364.

2-(((benzyloxy)carbonyl)amino)-3-(diphenylphosphoryl)-2-phenylpropanoic acid (3ca)

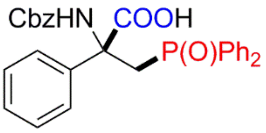

87.9 mg, 0.176 mmol, 88%;

White solid;

Mp: 218 - 219 ℃;

^{1}H NMR (400 MHz, DMSO-d_6) δ 13.52 (s, 1H), 7.88 – 7.82 (m, 2H), 7.81 – 7.74 (m, 2H), 7.60 – 7.39 (m, 8H), 7.36 – 7.25 (m, 5H), 7.22 (t, *J* = 7.1 Hz, 1H), 7.18 – 7.14 (m, 2H), 6.95 (s, 1H), 4.57 (d, *J* = 12.5 Hz, 1H), 4.12 (d, *J* = 12.5 Hz, 1H), 3.98 (dd, *J* = 14.9, 6.5 Hz, 1H), 3.69 – 3.56 (m, 1H).

^{13}C NMR (101 MHz, DMSO-d_6) δ 172.52 (d, *J* = 4.1 Hz), 153.47, 141.00 (d, *J* = 10.3 Hz), 136.70, 135.91 (d, *J* = 98.7 Hz), 133.93 (d, *J* = 98.6 Hz), 131.97 (d, *J* = 1.6 Hz), 1319.4 (d, *J* = 1.8 Hz), 131.07 (d, *J* = 9.4 Hz), 130.49 (d, *J* = 9.2 Hz), 129.11 (d, *J* = 11.5 Hz), 128.67, 128.50, 128.48 (d, *J* = 11.7 Hz), 128.16, 127.86, 126.21, 65.69, 62.10 (d, *J* = 4.7 Hz), 34.30 (d, *J* = 71.8 Hz).

^{31}P NMR (162 MHz, DMSO-d_6) δ 25.57.

HRMS (ESI+): calculated m/z $[M+H]^+$ for $[C_{25}H_{26}NO_4PH]^+$: 500.1621, found: 500.1617.

3-(diphenylphosphoryl)-2-(4-methylbenzamido)-2-phenylpropanoic acid (3da)

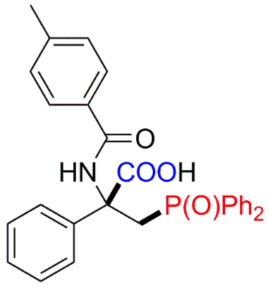

82.1 mg, 0.170 mmol, 85%;

White solid;

Mp: 191 - 193 ℃;

[1]**H NMR** (400 MHz, DMSO-d_6) δ 13.49 (s, 1H), 8.43 (s, 1H), 7.87 – 7.79 (m, 2H), 7.65 – 7.58 (m, 2H), 7.58 – 7.44 (m, 5H), 7.37 (d, *J* = 8.1 Hz, 2H), 7.26 – 7.13 (m, 8H), 4.14 (dd, *J* = 14.9, 6.7 Hz, 1H), 3.66 – 3.55 (m, 1H), 2.34 (s, 3H).

[13]**C NMR** (101 MHz, DMSO-d_6) δ 172.86 (d, *J* = 5.7 Hz), 164.90, 141.78, 140.18 (d, *J* = 8.9 Hz), 135.76 (d, *J* = 98.9 Hz), 133.17 (d, *J* = 99.0 Hz), 132.02 (d, *J* = 1.9 Hz), 131.28, 131.28 (d, overlapped), 130.76 (d, *J* = 9.5 Hz), 130.39 (d, *J* = 9.3 Hz), 129.18 (overlapped), 129.12 (d, *J* = 11.4 Hz), 128.34 (d, *J* = 10.9 Hz), 128.29 (overlapped), 127.66, 127.03, 126.45, 62.54 (d, *J* = 5.0 Hz), 34.22 (d, *J* = 70.6 Hz), 21.42.

[31]**P NMR** (162 MHz, DMSO-d_6) δ 26.95.

HRMS (ESI+): calculated m/z $[M+H]^+$ for $[C_{29}H_{26}NO_4PH]^+$: 484.1672, found: 484.1669.

3-(diphenylphosphoryl)-2-(4-methoxybenzamido)-2-phenylpropanoic acid (3ea)

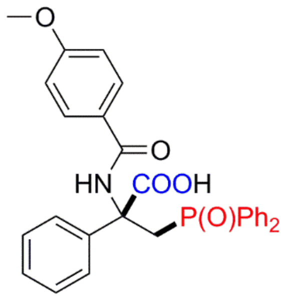

79.9 mg, 0.160 mmol, 80%;

White solid;

Mp: 227 - 228 ℃;

[1]**H NMR** (400 MHz, DMSO-d_6) δ 13.45 (s, 1H), 8.40 (s, 1H), 7.86 – 7.79 (m, 2H), 7.65 – 7.57 (m, 2H), 7.55 – 7.42 (m, 7H), 7.25 – 7.12 (m, 6H), 6.95 – 6.90 (m, 2H), 4.13 (dd, *J* = 14.9, 6.7 Hz, 1H), 3.80 (s, 3H), 3.65 – 3.53 (m, 1H).

[13]**C NMR** (101 MHz, DMSO-d_6) δ 172.89 (d, *J* = 5.8 Hz), 164.52, 162.15, 140.23 (d, *J* = 8.9 Hz), 135.74 (d, *J* = 98.8 Hz), 133.12 (d, *J* = 99.1 Hz), 132.02 (d, *J* = 2.7 Hz), 131.24 (d, *J* = 2.7 Hz), 130.76 (d, *J* = 9.6 Hz), 130.39 (d, *J* = 9.2 Hz), 129.18, 129.11 (d, *J* = 11.4 Hz), 128.91, 128.30 (d, *J* = 10.5 Hz), 128.29, 128.25 (overlapped), 127.61, 126.49, 126.28, 113.86, 62.56 (d, *J* = 5.0 Hz), 55.81, 34.38 (d, *J* = 70.9 Hz).

[31]**P NMR** (162 MHz, DMSO-d_6) δ 27.07.

HRMS (ESI+): calculated m/z $[M+H]^+$ for $[C_{29}H_{26}NO_5PH]^+$: 500.1621, found: 500.1620.

2-(4-chlorobenzamido)-3-(diphenylphosphoryl)-2-phenylpropanoic acid (3fa)

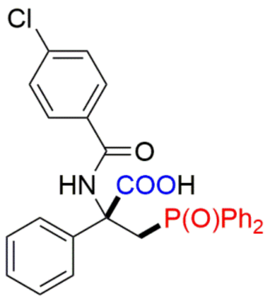

78.5 mg, 0.56 mmol, 78%;

White solid;

Mp: 224 - 226 ℃;

[1]**H NMR** (400 MHz, DMSO-d_6) δ 13.55 (s, 1H), 8.45 (s, 1H), 7.88 – 7.80 (m, 2H), 7.69 – 7.60 (m, 2H), 7.58 – 7.47 (m, 5H), 7.46 – 7.43 (m, 4H), 7.28 – 7.15 (m, 6H), 4.14 (dd, *J* = 15.0, 6.6 Hz, 1H), 3.70 – 3.60 (m, 1H).

[13]**C NMR** (101 MHz, DMSO-d_6) δ 172.78 (d, *J* = 5.1 Hz), 163.91 , 140.15 (d, *J* = 9.5 Hz), 136.65 , 135.65 (d, *J* = 99.0 Hz), 133.25 (d, *J* = 98.9 Hz), 132.53 , 132.03 (d, *J* = 2.0 Hz), 131.31 (d, *J* = 2.0 Hz), 130.81 (d, *J* = 9.5 Hz), 130.41 (d, *J* = 9.3 Hz), 129.12 (d, *J* = 11.5 Hz), 129.03 , 128.66 , 128.39 , 128.38 (d, *J* = 11.7 Hz), 127.75 , 126.39 , 62.60 (d, *J* = 5.0 Hz), 33.81 (d, *J* = 71.4 Hz).

^{31}**P NMR** (162 MHz, DMSO-d_6) δ 26.53.
HRMS (ESI+): calculated m/z [M+H]$^+$ for [C28H23ClNO4PH]$^+$: 504.1126, found: 504.1130.

3-(diphenylphosphoryl)-2-phenyl-2-(4-(trifluoromethyl)benzamido)propanoic acid (3ga)

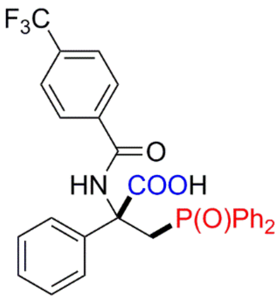

93.5 mg, 0.174 mmol, 87%;
White solid;
Mp: 212 - 214 ℃；
^{1}**H NMR** (400 MHz, DMSO-d_6) δ 13.62 (s, 1H), 8.57 (s, 1H), 7.88 – 7.80 (m, 2H), 7.75 (d, *J* = 8.2 Hz, 2H), 7.70 – 7.60 (m, 4H), 7.58 – 7.46 (m, 5H), 7.29 – 7.24 (m, 2H), 7.23 – 7.13 (m, 4H), 4.16 (dd, *J* = 15.0, 6.7 Hz, 1H), 3.77 – 3.62 (m, 1H).
^{13}**C NMR** (101 MHz, DMSO-d_6) δ 172.71 (d, *J* = 4.9 Hz), 163.79, 140.13 (d, *J* = 9.4 Hz), 137.38, 135.61 (d, *J* = 99.0 Hz), 133.33 (d, *J* = 98.8 Hz), 132.04 (d, *J* = 2.5 Hz), 131.67 (q, *J* = 32.0 Hz), 131.31 (d, *J* = 2.5 Hz), 130.83 (d, *J* = 9.6 Hz), 130.42 (d, *J* = 9.4 Hz), 129.12 (d, *J* = 11.4 Hz), 128.44, 128.39 (d, *J* = 10.7 Hz), 128.08, 127.81, 126.36, 125.58 (q, *J* = 3.8 Hz), 124.32 (d, *J* = 272.4 Hz), 62.68 (d, *J* = 4.9 Hz), 33.61 (d, *J* = 71.3 Hz).
^{31}**P NMR** (162 MHz, DMSO-d_6) δ 26.36.
^{19}**F NMR** (376 MHz, DMSO-d_6) δ -61.35.
HRMS (ESI+): calculated m/z [M+H]$^+$ for [$C_{29}H_{23}F_3NO_4PH$]$^+$: 538.1390, found: 538.1392.

3-(diphenylphosphoryl)-2-phenyl-2-(thiophene-2-carboxamido)propanoic acid (3ha)

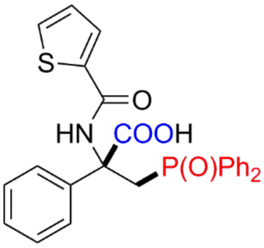

83.6 mg, 0.176 mmol, 88%;
White solid;
Mp: 217 - 218 ℃；
^{1}**H NMR** (400 MHz, DMSO-d_6) δ 13.52 (s, 1H), 8.36 (s, 1H), 7.87 – 7.79 (m, 2H), 7.68 (dd, *J* = 5.0, 1.1 Hz, 1H), 7.66 – 7.59 (m, 2H), 7.57 – 7.45 (m, 6H), 7.27 – 7.14 (m, 6H), 7.06 (dd, *J* = 5.0, 3.7 Hz, 1H), 4.08 (dd, *J* = 14.9, 6.7 Hz, 1H), 3.68 – 3.56 (m, 1H).
^{13}**C NMR** (101 MHz, DMSO-d_6) δ 172.59 (d, *J* = 5.5 Hz), 159.89, 140.25 (d, *J* = 9.2 Hz), 138.78, 135.55 (d, *J* = 98.9 Hz), 133.13 (d, *J* = 99.0 Hz), 132.01 (d, *J* = 2.4 Hz), 131.38, 131.18 (d, *J* = 2.0 Hz), 130.77 (d, *J* = 9.6 Hz), 130.45 (d, *J* = 9.3 Hz), 129.10 (d, *J* = 11.4 Hz), 128.90, 128.33 (overlapped), 128.28 (d, *J* = 11.9 Hz), 128.06, 127.71, 126.43, 62.65 (d, *J* = 5.0 Hz), 34.31 (d, *J* = 71.7 Hz).
^{31}**P NMR** (162 MHz, DMSO-d_6) δ 26.66.
HRMS (ESI+): calculated m/z [M+H]$^+$ for [$C_{26}H_{22}NO_4PSH$]$^+$: 476.1080, found: 476.1081.

3-(diphenylphosphoryl)-2-(furan-3-carboxamido)-2-phenylpropanoic acid (3ia)

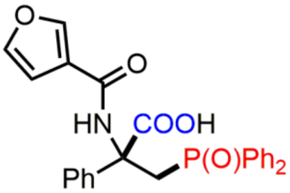

71.6 mg, 0.156 mmol, 78%;
White solid;
Mp: 196 - 197 ℃；
^{1}**H NMR** (400 MHz, DMSO-d_6) δ 13.28 (s, 1H), 8.07 (s, 1H), 7.96 (s, 1H), 7.87 – 7.76 (m, 2H), 7.73 – 7.59 (m, 3H), 7.58 – 7.41 (m, 5H), 7.36 – 7.01 (m, 6H), 6.46 (s, 1H), 4.06 (dd, *J* = 14.9, 6.9 Hz, 1H), 3.67 – 3.58 (m, 1H).

^{13}C NMR (101 MHz, DMSO-d_6) δ 172.60 (d, J = 5.7 Hz), 160.80, 145.37, 144.08, 140.35 (d, J = 8.8 Hz), 135.62 (d, J = 98.9 Hz), 133.39 (d, J = 98.9 Hz), 131.94 (d, J = 2.4 Hz), 131.20 (d, J = 2.5 Hz), 130.82 (d, J = 9.6 Hz), 130.44 (d, J = 9.3 Hz), 129.07 (d, J = 11.3 Hz), 128.34 (d, J = 11.2 Hz), 128.29, 127.64, 126.42, 122.17, 109.10, 62.43 (d, J = 4.9 Hz), 34.03 (d, J = 71.0 Hz).
^{31}P NMR (162 MHz, DMSO-d_6) δ 26.37.
HRMS (ESI+): calculated m/z $[M+H]^+$ for $[C_{26}H_{22}NO_5PH]^+$: 460.1308, found: 460.1306.

2-benzamido-3-(diphenylphosphoryl)-2-(*p*-tolyl)propanoic acid (3ja)

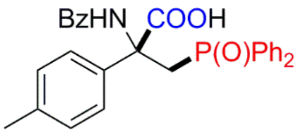

89.9 mg, 0.186 mmol, 93%;
White solid;
Mp: 202 - 204 ℃;
^{1}H NMR (400 MHz, DMSO-d_6) δ 13.46 (s, 1H), 8.45 (s, 1H), 7.88 – 7.78 (m, 2H), 7.65 – 7.58 (m, 2H), 7.57 – 7.44 (m, 6H), 7.41 – 7.32 (m, 4H), 7.25 – 7.14 (m, 3H), 7.01 (d, J = 8.1 Hz, 2H), 4.12 (dd, J = 14.9, 6.6 Hz, 1H), 3.64 – 3.52 (m, 1H), 2.21 (s, 3H).
^{13}C NMR (101 MHz, DMSO-d_6) δ 172.95 (d, J = 5.7 Hz), 164.90, 137.21 (d, J = 9.1 Hz), 136.86, 135.73 (d, J = 98.7 Hz), 133.99, 133.18 (d, J = 98.8 Hz), 132.01 (d, J = 2.2 Hz), 131.84, 131.13 (d, J = 2.8 Hz), 130.79 (d, J = 9.6 Hz), 130.40 (d, J = 9.3 Hz), 129.11 (d, J = 11.5 Hz), 128.88, 128.65, 128.32 (d, J = 11.8 Hz), 126.99, 126.35, 62.36 (d, J = 5.0 Hz), 34.28 (d, J = 71.1 Hz), 20.92.
^{31}P NMR (162 MHz, DMSO-d_6) δ 26.88.
HRMS (ESI+): calculated m/z $[M+H]^+$ for $[C_{29}H_{26}NO_4PH]^+$: 484.1672, found: 484.1663.

2-benzamido-3-(diphenylphosphoryl)-2-(4-methoxyphenyl)propanoic acid (3ka)

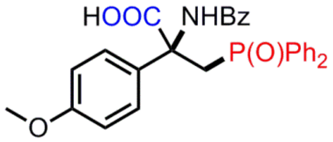

51.9 mg, 0.104 mmol, 52%;
White solid;
Mp: 209 - 211 ℃;
^{1}H NMR (400 MHz, DMSO-d_6) δ 13.26 (s, 1H), 8.52 (s, 1H), 7.89 – 7.77 (m, 2H), 7.67 – 7.46 (m, 8H), 7.44 – 7.31 (m, 4H), 7.27 – 7.15 (m, 3H), 6.74 (d, J = 8.7 Hz, 2H), 4.09 (dd, J = 14.9, 6.6 Hz, 1H), 3.69 (s, 3H), 3.55 (t, J = 14.3 Hz, 1H).
^{13}C NMR (101 MHz, DMSO-d_6) δ 173.01 (d, J = 6.2 Hz), 164.92, 158.75, 135.70 (d, J = 98.8 Hz), 133.99, 133.14 (d, J = 99.0 Hz), 132.02 (d, J = 2.5 Hz), 131.87, 131.85 (d, J = 8.1 Hz), 131.20 (d, J = 1.9 Hz), 130.78 (d, J = 9.7 Hz), 130.38 (d, J = 9.3 Hz), 129.12 (d, J = 11.5 Hz), 128.68, 128.31 (d, J = 11.9 Hz), 127.76, 127.01, 113.59, 62.18 (d, J = 4.9 Hz), 55.43, 34.47 (d, J = 70.2 Hz).
^{31}P NMR (162 MHz, DMSO-d_6) δ 27.10.
HRMS (ESI+): calculated m/z $[M+H]^+$ for $[C_{29}H_{26}NO_5PH]^+$: 500.1621, found: 500.1618.

2-([1,1'-biphenyl]-4-yl)-2-benzamido-3-(diphenylphosphoryl)propanoic acid (3la)

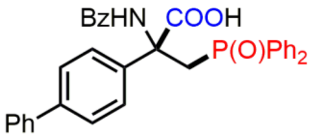

90.5 mg, 0.166 mmol, 83%;
White solid;
Mp: 228 - 230 ℃;
^{1}H NMR (400 MHz, DMSO-d_6) δ 13.52 (s, 1H), 8.70 (s, 1H), 7.91 – 7.81 (m, 2H), 7.65 – 7.49 (m, 12H), 7.49 – 7.39 (m, 6H), 7.35 (t, J = 7.2 Hz, 1H), 7.25 – 7.08 (m, 3H), 4.17 (dd, J = 15.0, 6.7 Hz, 1H), 3.61 (t, J = 14.4 Hz, 1H).
^{13}C NMR (101 MHz, DMSO-d_6) δ 172.68 (d, J = 6.5 Hz), 165.04, 140.22, 139.59, 139.00 (d, J = 8.2 Hz), 135.57 (d, J = 99.0 Hz), 133.85, 132.97 (d, J = 99.1 Hz), 132.10 (d, J = 2.3 Hz), 131.98,

131.17 (d, J = 2.7 Hz), 130.82 (d, J = 9.6 Hz), 130.41 (d, J = 9.4 Hz), 129.32, 129.15 (d, J = 11.5 Hz), 128.74, 128.33 (d, J = 11.9 Hz), 127.84, 127.28, 127.11, 126.60, 62.65 (d, J = 5.2 Hz), 34.66 (d, J = 71.0 Hz).

^{31}P NMR (162 MHz, DMSO-d_6) δ 27.45.

HRMS (ESI+): calculated m/z $[M+H]^+$ for $[C_{34}H_{28}NO_4PH]^+$: 546.1829, found: 546.1826.

2-benzamido-3-(diphenylphosphoryl)-2-(4-fluorophenyl)propanoic acid (3ma)

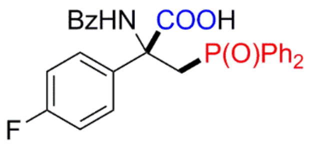

68.2 mg, 0.140 mmol, 70%;

White solid;

Mp: 224 - 225 ℃;

^{1}H NMR (400 MHz, DMSO-d_6) δ 13.46 (s, 1H), 8.80 (s, 1H), 7.88 – 7.80 (m, 2H), 7.62 – 7.40 (m, 12H), 7.28 – 7.15 (m, 3H), 7.02 – 6.93 (m, 2H), 4.12 (dd, J = 15.0, 6.8 Hz, 1H), 3.59 – 3.47 (m, 1H).

^{13}C NMR (101 MHz, DMSO-d_6) δ 172.53 (d, J_{C-P} = 7.2 Hz), 165.06, 161.71 (d, J_{C-F} = 243.7 Hz), 135.68 (dd, J_{C-P} = 8.3 Hz, J_{C-F} = 2.5 Hz), 135.41 (d, J_{C-P} = 99.4 Hz), 133.75, 132.80 (d, J_{C-P} = 99.1 Hz), 132.14 (d, J_{C-P} = 2.6 Hz), 132.02, 131.29 (d, J_{C-P} = 2.6 Hz), 130.79 (d, J_{C-P} = 9.6 Hz), 130.37 (d, J_{C-P} = 9.4 Hz), 129.15 (d, J_{C-P} = 11.5 Hz), 128.98 (d, J_{C-F} = 8.3 Hz), 128.76, 128.36 (d, J_{C-P} = 11.9 Hz), 127.12, 114.80 (d, J_{C-F} = 21.5 Hz), 62.49 (d, J_{C-P} = 5.0 Hz), 34.90 (d, J_{C-P} = 70.2 Hz).

^{31}P NMR (162 MHz, DMSO-d_6) δ 27.76.

^{19}F NMR (376 MHz, DMSO-d_6) δ -115.87.

HRMS (ESI+): calculated m/z $[M+H]^+$ for $[C_{28}H_{23}FNO_4PH]^+$: 488.1421, found: 488.1513.

2-benzamido-2-(4-chlorophenyl)-3-(diphenylphosphoryl)propanoic acid (3na)

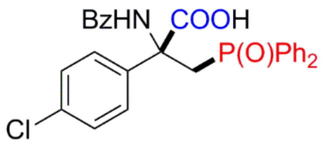

78.5 mg, 0.156 mmol, 78%;

White solid;

Mp: 198 - 200 ℃;

^{1}H NMR (400 MHz, DMSO-d_6) δ 13.55 (s, 1H), 8.79 (s, 1H), 7.87 – 7.79 (m, 2H), 7.61 – 7.48 (m, 8H), 7.46 – 7.40 (m, 4H), 7.27 – 7.15 (m, 5H), 4.09 (dd, J = 15.0, 6.8 Hz, 1H), 3.58 – 3.45 (m, 1H).

^{13}C NMR (101 MHz, DMSO-d_6) δ 172.14 (d, J = 7.5 Hz), 165.10, 141.95 (d, J = 7.9 Hz), 135.28 (d, J = 99.6 Hz), 133.59, 133.07, 132.69 (d, J = 99.4 Hz), 132.18 (d, J = 2.9 Hz, overlapped), 132.10, 131.41 (d, J = 2.7 Hz), 130.69 (d, J = 9.7 Hz), 130.40 (d, J = 9.4 Hz), 129.94, 129.15 (d, J = 11.6 Hz), 128.81, 128.33 (d, J = 11.9 Hz), 127.69, 127.29, 127.16, 125.31, 62.73 (d, J = 5.0 Hz), 34.62 (d, J = 70.2 Hz).

^{31}P NMR (162 MHz, DMSO-d_6) δ 27.77.

HRMS (ESI+): calculated m/z $[M+H]^+$ for $[C_{28}H_{23}ClNO_4PH]^+$: 504.1126, found: 504.1128.

2-benzamido-2-(4-bromophenyl)-3-(diphenylphosphoryl)propanoic acid (3oa)

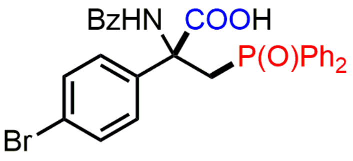

85.3 mg, 0.156 mmol, 78%;

White solid;

Mp: 196 - 197 ℃;

^{1}H NMR (400 MHz, DMSO-d_6) δ 13.53 (s, 1H), 8.78 (s, 1H), 7.89 – 7.79 (m, 2H), 7.62 – 7.48 (m, 8H), 7.47 – 7.32 (m, 6H), 7.28 – 7.22 (m, 1H), 7.21 – 7.14 (m, 2H), 4.09 (dd, J = 15.0, 6.8 Hz, 1H), 3.51 (t, J = 14.5 Hz, 2H).

^{13}C NMR (101 MHz, DMSO-d_6) δ 172.26 (d, J = 7.1 Hz), 165.06, 139.10 (d, J = 8.2 Hz), 135.29 (d, J = 99.4 Hz), 133.63, 132.69 (d, J = 99.3 Hz), 132.17 (d, J = 2.1 Hz), 132.06, 131.22 (d, J = 2.2 Hz), 130.99, 130.78 (d, J = 9.7 Hz), 130.39 (d, J = 9.3 Hz), 129.16 (d, J = 11.5 Hz), 129.12, 128.76, 128.39 (d, J = 11.9 Hz), 127.13, 121.17, 62.61 (d, J = 5.0 Hz), 34.78 (d, J = 70.3 Hz).
^{31}P NMR (162 MHz, DMSO-d_6) δ 27.76.
HRMS (ESI+): calculated m/z $[M+H]^+$ for $[C_{28}H_{23}BrNO_4PH]^+$: 548.0621, found: 548.0625.

2-benzamido-3-(diphenylphosphoryl)-2-(4-(trifluoromethoxy)phenyl)propanoic acid (3pa)

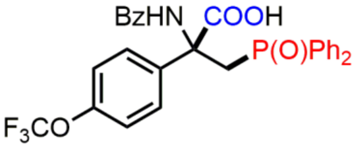

95.1 mg, 0.172 mmol, 86%;
White solid;
Mp: 180 - 182 ℃;
^{1}H NMR (400 MHz, DMSO-d_6) δ 13.53 (s, 1H), 9.00 (s, 1H), 7.89 – 7.79 (m, 2H), 7.70 – 7.39 (m, 12H), 7.27 – 7.21 (m, 1H), 7.22 – 6.99 (m, 4H), 4.11 (dd, J = 15.1, 6.8 Hz, 1H), 3.50 (t, J = 14.6 Hz, 1H).
^{13}C NMR (101 MHz, DMSO-d_6) δ 172.22 (d, J = 8.1 Hz), 165.13, 147.79 (q, J = 1.5 Hz), 138.50 (d, J = 7.5 Hz), 135.23 (d, J = 99.8 Hz), 133.58, 132.50 (d, J = 99.0 Hz), 132.21 (d, J = 1.9 Hz), 132.12, 131.36 (d, J = 2.7 Hz), 130.76 (d, J = 9.7 Hz), 130.36 (d, J = 9.4 Hz), 129.17 (d, J = 11.6 Hz), 129.10, 128.82, 128.34 (d, J = 12.0 Hz), 127.19, 120.44, 120.42 (q, J = 256.2 Hz), 62.74 (d, J = 5.1 Hz), 35.09 (d, J = 70.0 Hz).
^{31}P NMR (162 MHz, DMSO-d_6) δ 28.24.
HRMS (ESI+): calculated m/z $[M+H]^+$ for $[C_{29}H_{23}F_3NO_5PH]^+$: 554.1339, found: 554.1342.

2-benzamido-3-(diphenylphosphoryl)-2-(*m*-tolyl)propanoic acid (3qa)

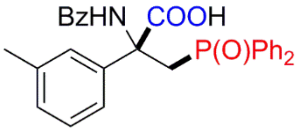

90.8 mg, 0.188 mmol, 94%;
White solid;
Mp: 204 - 206 ℃;
^{1}H NMR (400 MHz, DMSO-d_6) δ 13.45 (s, 1H), 8.47 (s, 1H), 7.87 – 7.77 (m, 2H), 7.66 – 7.58 (m, 2H), 7.57 – 7.43 (m, 6H), 7.41 – 7.35 (m, 2H), 7.28 (s, 1H), 7.26 – 7.14 (m, 4H), 7.10 (t, J = 7.7 Hz, 1H), 6.96 (d, J = 7.4 Hz, 1H), 4.13 (dd, J = 14.9, 6.4 Hz, 1H), 3.65 – 3.52 (m, 1H), 2.21 (s, 3H).
^{13}C NMR (101 MHz, DMSO) δ 172.42 (d, J = 5.5 Hz), 164.51, 139.90 (d, J = 5.6 Hz), 136.88, 135.26 (d, J = 99.9 Hz), 133.66, 132.68 (d, J = 99.6 Hz), 131.61, 131.37, 130.84, 130.35 (d, J = 9.6 Hz), 130.00 (d, J = 9.3 Hz), 128.68 (d, J = 11.5 Hz), 128.21, 127.86 (d, J = 11.8 Hz), 127.84, 127.77, 126.69, 126.58, 122.92, 62.13 (d, J = 4.9 Hz), 33.69 (d, J = 73.3 Hz), 21.14.
^{31}P NMR (162 MHz, DMSO-d_6) δ 27.00.
HRMS (ESI+): calculated m/z $[M+H]^+$ for $[C_{29}H_{26}NO_4PH]^+$: 484.1672, found: 484.1677.

2-benzamido-3-(diphenylphosphoryl)-2-(3-methoxyphenyl)propanoic acid (3ra)

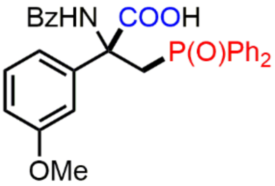

89.8 mg, 0.180 mmol, 90%;
White solid;
Mp: 184 - 185 ℃;
^{1}H NMR (400 MHz, DMSO-d_6) δ 12.95 (s, 1H), 9.11 (s, 1H), 7.85 – 7.77 (m, 2H), 7.73 – 7.66 (m, 2H), 7.58 – 7.42 (m, 8H), 7.33 (dd, J = 7.9, 1.7 Hz, 1H), 7.28 – 7.16 (m, 3H), 7.05 (td, J = 7.8, 1.6 Hz, 1H), 6.81 (td, J = 7.6, 1.1 Hz, 1H),

6.56 (d, J = 8.3 Hz, 0H), 6.56 (dd, J = 8.3, 1.1 Hz, 1H), 4.25 (dd, J = 15.0, 7.4 Hz, 1H), 3.60 (s, 3H), 3.28 (t, J = 14.3 Hz, 1H).

^{13}C NMR (101 MHz, DMSO-d_6) δ 171.07 (d, J = 10.2 Hz), 164.77, 157.10, 135.19 (d, J = 99.4 Hz), 134.34, 132.33 (d, J = 99.5 Hz), 132.18 (d, J = 2.1 Hz), 131.97, 131.27 (d, J = 2.2 Hz), 130.50 (d, J = 9.6 Hz), 130.44 (d, J = 9.1 Hz), 129.47, 129.17 (d, J = 11.4 Hz), 128.96, 128.25 (d, J = 11.9 Hz), 127.96, 127.16, 126.84 (d, J = 6.1 Hz), 120.04, 111.74, 62.13 (d, J = 4.6 Hz), 55.54, 34.01 (d, J = 70.6 Hz).

^{31}P NMR (162 MHz, DMSO-d_6) δ 28.49.

HRMS (ESI+): calculated m/z $[M+H]^+$ for $[C_{29}H_{26}NO_5PH]^+$: 500.1621, found: 500.1614.

2-benzamido-2-(3-chlorophenyl)-3-(diphenylphosphoryl)propanoic acid (3sa)

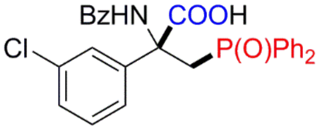

84.5 mg, 0.168 mmol, 84%;

White solid;

Mp: 210 - 212 ℃;

^{1}H NMR (400 MHz, DMSO-d_6) δ 13.55 (s, 1H), 8.77 (s, 1H), 7.89 – 7.79 (m, 2H), 7.62 – 7.48 (m, 8H), 7.46 – 7.38 (m, 4H), 7.29 – 7.14 (m, 5H), 4.09 (dd, J = 15.0, 6.6 Hz, 1H), 3.62 – 3.48 (m, 1H).

^{13}C NMR (101 MHz, DMSO-d_6) δ 172.14 (d, J = 7.4 Hz), 165.10, 141.96 (d, J = 7.9 Hz), 135.28 (d, J = 99.6 Hz), 133.59, 133.07, 132.69 (d, J = 99.3 Hz), 132.18 (d, J = 3.0 Hz), 132.10, 131.42 (d, J = 2.7 Hz), 130.69 (d, J = 9.6 Hz), 130.40 (d, J = 9.4 Hz), 129.94, 129.15 (d, J = 11.6 Hz), 128.80, 128.33 (d, J = 11.8 Hz), 127.69, 127.29, 127.16, 125.31, 62.73 (d, J = 5.0 Hz), 34.62 (d, J = 69.8 Hz).

^{31}P NMR (162 MHz, DMSO-d_6) δ 27.86.

HRMS (ESI+): calculated m/z $[M+H]^+$ for $[C_{28}H_{23}ClNO_4PH]^+$: 504.1126, found: 504.1127.

2-benzamido-3-(diphenylphosphoryl)-2-(3-(trifluoromethyl)phenyl)propanoic acid (3ta)

81.7 mg, 0.152 mmol, 76%;

White solid;

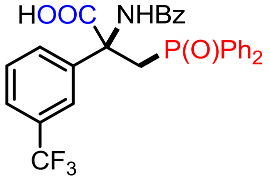

Mp: 210 - 212 ℃;

^{1}H NMR (400 MHz, DMSO) δ 13.49 (s, 1H), 9.23 (s, 1H), 7.90 – 7.80 (m, 2H), 7.76 – 7.65 (m, 4H), 7.61 – 7.41 (m, 9H), 7.36 (m, 1H), 7.23 (m, 1H), 7.15 (m, 2H), 4.15 (dd, J = 15.1, 6.9 Hz, 1H), 3.50 (t, J = 14.7 Hz, 1H).

^{13}C NMR (101 MHz, dmso) δ 171.99 (d, J = 8.9 Hz), 165.28, 140.25 (d, J = 6.8 Hz), 135.02 (d, J = 100.0 Hz), 133.48, 132.28 (d, J = 2.2 Hz), 132.28 (d, J = 99.1 Hz), 132.23, 131.42 (d, J = 2.2 Hz), 131.09, 130.66 (d, J = 9.8 Hz), 130.36 (d, J = 9.5 Hz), 129.18 (d, J = 11.7 Hz), 129.10, 129.07 (q, J = 31.6 Hz), 128.93, 128.38 (d, J = 12.1 Hz), 127.21, 124.57 (q, J = 272.5 Hz), 124.51 (q, J = 3.8 Hz), 124.13 (q, J = 3.3 Hz), 63.07 (d, J = 5.1 Hz), 35.13 (d, J = 69.4 Hz).

^{31}P NMR (162 MHz, DMSO) δ 28.90.

^{19}F NMR (376 MHz, DMSO) δ -60.88.

HRMS (ESI+): calculated m/z $[M+H]^+$ for $[C_{29}H_{23}F_3NO_4PH]^+$: 538.1390, found: 538.1395.

2-benzamido-3-(diphenylphosphoryl)-2-(*o*-tolyl)propanoic acid (3ua)

85.0 mg, 0.176 mmol, 88%;

White solid;

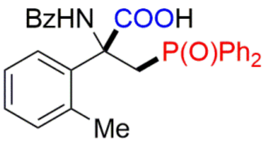

Mp: 221 - 222 ℃;

^{1}H NMR (400 MHz, DMSO-d_6) δ 13.81 (s, 1H), 8.04 (s, 1H), 7.91 – 7.84 (m, 2H), 7.75 – 7.64 (m, 3H), 7.59 – 7.44 (m, 4H), 7.40 – 7.32 (m, 4H), 7.27 – 7.21 (m, 1H), 7.18 – 7.10 (m, 4H), 7.01 – 6.96 (m, 1H), 4.26 (dd, *J* = 14.6, 7.5 Hz, 1H), 3.71 – 3.59 (m, 1H), 2.22 (s, 3H).

^{13}C NMR (101 MHz, DMSO) δ 172.24, 163.89, 138.56 (d, *J* = 7.5 Hz), 135.63 (d, *J* = 99.1 Hz), 135.57, 133.54 (d, *J* = 99.8 Hz), 133.46, 132.66, 132.05 (d, *J* = 1.7 Hz), 131.81, 131.30 (d, *J* = 2.0 Hz), 130.67 (d, *J* = 7.7 Hz), 130.58 (d, *J* = 9.1 Hz), 129.13 (d, *J* = 11.4 Hz), 128.64, 128.46 (d, *J* = 11.8 Hz), 128.32, 127.68, 126.92, 126.37, 62.49 (d, *J* = 4.3 Hz), 35.00 (d, *J* = 71.6 Hz), 21.34.

^{31}P NMR (162 MHz, DMSO-d_6) δ 25.72.

HRMS (ESI+): calculated m/z $[M+H]^+$ for $[C_{29}H_{26}NO_4PH]^+$: 484.1672, found: 484.1673.

2-Benzamido-3-(diphenylphosphoryl)-2-(2-fluorophenyl)propanoic acid (3va)

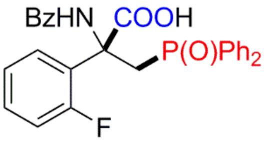

92.6 mg, 0.190 mmol, 95%;

White solid;

Mp: 192 - 193 ℃;

^{1}H NMR (400 MHz, DMSO-d_6) δ 13.47 (s, 1H), 9.21 (s, 1H), 7.91 – 7.81 (m, 2H), 7.72 – 7.65 (m, 2H), 7.62 – 7.44 (m, 8H), 7.42 – 7.32 (m, 1H), 7.30 – 7.14 (m, 3H), 7.18 – 7.08 (m, 1H), 7.09 – 7.00 (m, 1H), 6.79 – 6.69 (m, 1H), 4.07 (dd, *J* = 15.2, 6.8 Hz, 1H), 3.47 – 3.34 (m, 1H).

^{13}C NMR (101 MHz, DMSO-d_6) δ 170.57 (d, J_{C-P} = 9.5 Hz), 164.95, 160.23 (d, J_{C-F} = 247.2 Hz), 134.91 (d, J_{C-P} = 100.1 Hz), 133.61, 132.36 (d, J_{C-P} = 2.4 Hz), 132.23, 131.93 (d, J_{C-P} = 99.4 Hz), 131.48 (d, J_{C-P} = 2.0 Hz), 130.53 (d, J_{C-P} = 10.0 Hz), 130.44 (d, J_{C-P} = 9.5 Hz), 130.34 (d, J_{C-F} = 10.5 Hz, overlapped), 129.25 (d, J_{C-P} = 11.6 Hz), 129.01, 128.91 (d, J_{C-F} = 3.0 Hz), 128.42 (d, J_{C-P} = 11.9 Hz), 127.16, 126.01 (dd, J_{C-F} = 10.9 Hz, J_{C-P} = 6.3 Hz), 124.03 (d, J_{C-F} = 2.7 Hz), 116.15 (d, J_{C-F} = 23.2 Hz), 61.62 (dd, J_{C-P} = 4.8 Hz, J_{C-F} = 2.8 Hz), 34.27 (d, J_{C-P} = 69.1 Hz).

^{31}P NMR (162 MHz, DMSO-d_6) δ 28.89.

^{19}F NMR (376 MHz, DMSO-d_6) δ -108.24.

HRMS (ESI+): calculated m/z $[M+H]^+$ for $[C_{28}H_{23}FNO_4PH]^+$: 488.1421, found: 488.1425.

2-benzamido-2-(2-chlorophenyl)-3-(diphenylphosphoryl)propanoic acid (3wa)

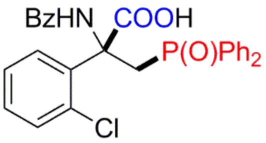

93.6 mg, 0.186 mmol, 93%;

White solid;

Mp: 206 - 207 ℃;

^{1}H NMR (400 MHz, DMSO-d_6) δ 13.76 (s, 1H), 8.47 (s, 1H), 7.92 – 7.78 (m, 3H), 7.67 – 7.59 (m, 2H), 7.58 – 7.48 (m, 6H), 7.40 (t, *J* = 7.6 Hz, 2H), 7.37 – 7.31 (m, 1H), 7.25 – 7.16 (m, 5H), 4.31 (dd, *J* = 14.8, 8.1 Hz, 1H), 3.58 – 3.45 (m, 1H).

^{13}C NMR (101 MHz, DMSO-d_6) δ 170.47 (d, *J* = 5.3 Hz), 164.32, 137.18 (d, *J* = 9.5 Hz), 134.91 (d, *J* = 99.7 Hz), 133.73, 133.01 (d, *J* = 99.6 Hz), 132.20 (d, *J* = 2.1 Hz), 131.93, 131.39, 130.61 (d, *J* = 9.2 Hz), 130.55 (d, *J* = 9.6 Hz), 130.34, 129.57, 129.14 (d, *J* = 11.5 Hz), 128.75, 128.50 (d, *J* = 11.8 Hz), 127.23, 127.07, 62.17 (d, *J* = 4.1 Hz), 33.98 (d, *J* = 70.4 Hz).

^{31}P NMR (162 MHz, DMSO-d_6) δ 26.19.

HRMS (ESI+): calculated m/z $[M+H]^+$ for $[C_{28}H_{23}ClNO_4PH]^+$: 504.1126, found: 504.1123.

2-benzamido-2-(3,4-dimethylphenyl)-3-(diphenylphosphoryl)propanoic acid (3xa)

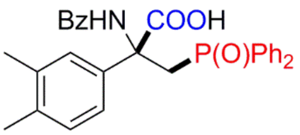

71.6 mg, 0.144 mmol, 72%;

White solid;

Mp: 207 - 208 ℃;

^{1}H NMR (400 MHz, DMSO-d_6) δ 13.38 (s, 1H), 8.47 (s, 1H), 7.87 – 7.76 (m, 2H), 7.63 – 7.46 (m, 8H), 7.43 – 7.36 (m, 2H), 7.26 – 7.12 (m, 5H), 6.95 (d, *J* = 8.0 Hz, 1H), 4.10 (dd, *J* = 14.9, 6.5 Hz, 1H), 3.61 – 3.49 (m, 1H), 2.10 (s, 6H).

^{13}C NMR (101 MHz, DMSO-d_6) δ 172.98 (d, *J* = 6.3 Hz), 164.93, 137.35 (d, *J* = 8.8 Hz), 135.89, 135.76 (d, *J* = 98.8 Hz), 135.61, 134.03, 133.09 (d, *J* = 98.9 Hz), 132.02 (d, *J* = 2.3 Hz), 131.85, 131.06 (d, *J* = 2.4 Hz), 130.74 (d, *J* = 9.5 Hz), 130.38 (d, *J* = 9.3 Hz), 129.37, 129.12 (d, *J* = 11.4 Hz), 128.68, 128.20 (d, *J* = 11.8 Hz), 127.68, 127.02, 123.65, 62.31 (d, *J* = 5.0 Hz), 34.36 (d, *J* = 71.1 Hz), 20.01, 19.32.

^{31}P NMR (162 MHz, DMSO-d_6) δ 27.07.

HRMS (ESI+): calculated m/z $[M+H]^+$ for $[C_{30}H_{28}NO_4PH]^+$: 498.1829, found: 498.1827.

methyl 2-benzamido-3-(diphenylphosphoryl)-2-phenylbutanoate (3ya)

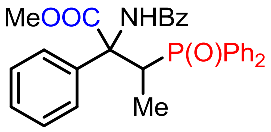

37.8 mg, 0.076 mmol, 38%;

White solid;

Mp: 204 - 205 ℃;

^{1}H NMR (400 MHz, DMSO-d_6) δ 9.85 (s, 1H), 9.72 (s, 1H), 8.07 – 7.84 (m, 4H), 7.80 – 7.35 (m, 11H), 7.29 – 7.11 (m, 3H), 6.98 – 6.86 (m, 2H), 4.02 (p, *J* = 7.2 Hz, 1H), 3.78 (dq, *J* = 15.0, 7.3 Hz, 1H), 3.46 (s, 2H), 2.87 (s, 1H), 1.20 (dd, *J* = 16.5, 7.2 Hz, 2H), 0.92 (dd, *J* = 15.7, 7.4 Hz, 1H).

^{13}C NMR (101 MHz, DMSO-d_6) δ 170.62 (d, *J* = 5.0 Hz), 170.39 (d, *J* = 12.4 Hz), 166.95 , 166.15, 137.82 (d, *J* = 3.7 Hz), 137.23 (d, *J* = 7.8 Hz), 134.28, 134.24, 133.13 (d, *J* = 96.1 Hz), 133.03 (d, *J* = 95.7 Hz), 132.97 (d, *J* = 96.8 Hz), 132.36, 132.32 (d, *J* = 97.7 Hz), 132.19 (d, *J* = 1.9 Hz), 132.08 (d, *J* = 1.8 Hz), 131.16 (d, *J* = 2.2 Hz), 131.01 (d, *J* = 8.7 Hz), 130.81 (d, *J* = 9.6 Hz), 130.49 (d, *J* = 9.5 Hz), 129.28, 129.23 (d, *J* = 10.6 Hz), 129.18 (d, *J* = 11.6 Hz), 128.97 (d, *J* = 11.6 Hz), 128.60, 128.41 (d, *J* = 11.8 Hz), 127.86, 127.77, 127.62, 127.57, 127.56 (d, *J* = 12.3 Hz), 127.49, 127.49, 67.58 (d, *J* = 2.8 Hz), 67.39 (d, *J* = 2.3 Hz), 52.55, 52.11, 40.11 (d, *J* = 63.0 Hz), 38.77 (d, *J* = 63.5 Hz), 12.89, 11.99.

^{31}P NMR (162 MHz, DMSO-d_6) δ 37.85.

HRMS (ESI+): calculated m/z $[M+Na]^+$ for $[C_{30}H_{28}NNaO_4P]^+$: 520.1648, found: 520.1641.

2-benzamido-3-(diphenylphosphoryl)-2-(naphthalen-2-yl)propanoic acid (3za)

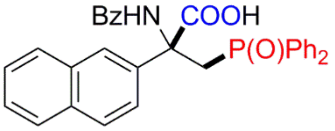

89.3 mg, 0.172 mmol, 86%;

White solid;

Mp: 220 - 221 ℃;

^{1}H NMR (400 MHz, DMSO-d_6) δ 13.63 (s, 1H), 8.56 (s, 1H), 8.12 – 8.08 (m, 1H), 7.92 – 7.79 (m, 4H), 7.75 (d, *J* = 8.8 Hz, 1H), 7.67 – 7.44 (m, 11H), 7.41 – 7.36 (m, 2H), 7.14 – 7.04 (m, 3H), 4.27 (dd, *J* = 14.9, 6.6 Hz, 1H), 3.83 – 3.70 (m, 1H).

^{13}C NMR (101 MHz, DMSO-d_6) δ 172.83 (d, *J* = 5.4 Hz), 165.04, 137.90 (d, *J* = 9.2 Hz), 135.69 (d, *J* = 98.8 Hz), 133.91, 133.07 (d, *J* = 98.9 Hz), 132.95, 132.53, 132.07 (d, *J* = 2.5 Hz), 131.89,

131.14 (d, J = 2.0 Hz), 130.73 (d, J = 9.5 Hz), 130.47 (d, J = 9.3 Hz), 129.15 (d, J = 11.5 Hz), 128.66, 128.51, 128.23 (d, J = 11.8 Hz), 127.93, 127.61, 127.06, 126.58, 126.54, 125.64, 124.28, 62.74 (d, J = 5.0 Hz), 34.19 (d, J = 71.2 Hz).

^{31}P NMR (162 MHz, DMSO-d_6) δ 26.94.

HRMS (ESI+): calculated m/z $[M+H]^+$ for $[C_{32}H_{26}NO_4PH]^+$: 520.1672, found: 520.1667.

methyl 2-acetamido-3-(diphenylphosphoryl)-2-(pyridin-2-yl)propanoate (3aaa)

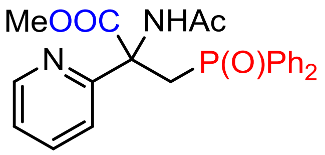

65.9 mg, 0.156 mmol, 78%;

White solid;

Mp: 97-98 ℃;

^{1}H NMR (400 MHz, DMSO-d_6) δ 8.44 (d, J = 4.2 Hz, 1H), 8.39 (s, 1H), 7.76 (m, 3H), 7.70 – 7.63 (m, 2H), 7.57 – 7.41 (m, 7H), 7.25 (dd, J = 7.3, 4.9 Hz, 1H), 3.83 (m, 2H), 3.51 (s, 3H), 1.34 (s, 3H).

^{13}C NMR (101 MHz, DMSO-d_6) δ 171.30 (d, J = 9.7 Hz), 169.80, 156.88 (d, J = 5.7 Hz), 148.41, 137.42, 135.24 (d, J = 98.6 Hz), 134.67 (d, J = 98.5 Hz), 131.75 (d, J = 2.4 Hz), 131.55 (d, J = 2.6 Hz), 130.69 (d, J = 9.3 Hz), 130.63 (d, J = 9.1 Hz), 128.97 (d, J = 11.4 Hz), 128.75 (d, J = 11.5 Hz), 123.35, 120.99 (s), 64.08 (d, J = 4.1 Hz), 53.20, 33.55 (d, J = 73.9 Hz), 22.29.

^{31}P NMR (162 MHz, DMSO-d_6) δ 24.71.

HRMS (ESI+): calculated m/z $[M+Na]^+$ for $[C_{23}H_{23}N_2O_4PNa]^+$: 445.1288, found: 445.1290.

methyl 2-acetamido-3-(diphenylphosphoryl)-2-(pyridin-3-yl)propanoate (3aba)

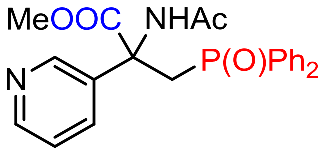

38.9 mg, 0.092 mmol, 46%;

White solid;

Mp: 99 - 100 ℃;

^{1}H NMR (400 MHz, DMSO-d_6) δ 8.68 (d, J = 2.1 Hz, 1H), 8.50 (s, 1H), 8.40 (dd, J = 4.7, 1.4 Hz, 1H), 7.88 – 7.77 (m, 3H), 7.75 – 7.67 (m, 2H), 7.58 – 7.39 (m, 6H), 7.27 (dd, J = 8.0, 4.7 Hz, 1H), 3.95 (dd, J = 15.1, 7.8 Hz, 1H), 3.64 (dd, J = 14.8, 13.0 Hz, 1H), 3.54 (s, 3H), 1.31 (s, 3H).

^{13}C NMR (101 MHz, DMSO-d_6) δ 171.47 (d, J = 7.2 Hz), 169.81, 148.74, 147.84, 135.39 (d, J = 7.7 Hz), 135.18 (d, J = 99.2 Hz), 134.29, 133.97 (d, J = 99.0 Hz), 131.97 (d, J = 2.3 Hz), 131.62 (d, J = 2.3 Hz), 130.86 (d, J = 9.5 Hz), 130.58 (d, J = 9.4 Hz), 129.06 (d, J = 11.6 Hz), 128.72 (d, J = 11.7 Hz), 123.31, 61.52 (d, J = 4.8 Hz), 53.35, 33.26 (d, J = 71.8 Hz), 22.31.

^{31}P NMR (162 MHz, DMSO-d_6) δ 25.50.

HRMS (ESI+): calculated m/z $[M+Na]^+$ for $[C_{23}H_{23}N_2O_4PNa]^+$: 445.1288, found: 445.1286.

2-benzamido-3-(di-p-tolylphosphoryl)-2-phenylpropanoic acid (3ab)

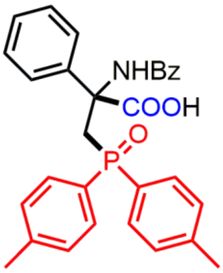

61.6 mg, 0.124 mmol, 62%;

White solid;

Mp: 191 - 193 ℃;

^{1}H NMR (400 MHz, DMSO-d_6) δ 8.37 (s, 1H), 7.68 (dd, J = 11.1, 7.8 Hz, 2H), 7.59 – 7.34 (m, 9H), 7.34 – 7.28 (m, 2H), 7.23 (dt, J = 14.7, 7.1 Hz, 3H), 7.01 – 6.86 (m, 2H), 4.04 (dd, J = 14.9, 6.6 Hz, 1H), 3.57 (t, J = 14.5 Hz, 1H), 2.33 (s, 3H), 2.10 (s, 3H).

^{13}**C NMR** (101 MHz, DMSO-d_6) δ 172.90 (d, J = 5.0 Hz), 164.65, 141.95 (d, J = 2.6 Hz), 141.17 (d, J = 2.8 Hz), 140.48 (d, J = 9.4 Hz), 133.71, 132.62 (d, J = 101.2 Hz), 131.29 (d, J = 102.0 Hz), 130.88, 130.78, 130.52, 130.44 (d, J = 9.6 Hz), 129.65 (d, J = 11.8 Hz), 129.51, 128.94 (d, J = 12.2 Hz), 128.50, 128.35, 127.59, 126.97, 126.36, 62.45 (d, J = 4.9 Hz), 33.91 (d, J = 71.1 Hz), 21.47, 21.30.

^{31}**P NMR** (162 MHz, DMSO-d_6) δ 26.96.

HRMS (ESI+): calculated m/z $[M+H]^+$ for $[C_{30}H_{28}NO_4PH]^+$: 498.1829, found: 498.1827.

2-benzamido-3-(di-*m*-tolylphosphoryl)-2-phenylpropanoic acid (3ac)

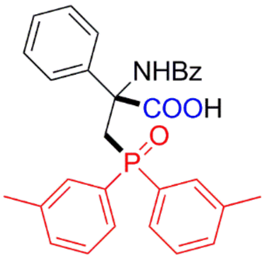

84.5 mg, 0.170 mmol, 85%;

White solid;

Mp: 198 - 200 ℃;

^{1}**H NMR** (400 MHz, DMSO-d_6) δ 13.51 (s, 1H), 8.49 (s, 1H), 7.71 – 7.58 (m, 2H), 7.55 – 7.43 (m, 5H), 7.44 – 7.31 (m, 6H), 7.26 – 7.21 (m, 2H), 7.20 – 7.14 (m, 1H), 7.13 – 7.03 (m, 1H), 6.96 (d, J = 7.6 Hz, 1H), 4.09 (dd, J = 14.9, 6.5 Hz, 1H), 3.66 – 3.52 (m, 1H), 2.34 (s, 3H), 2.03 (s, 3H).

^{13}**C NMR** (101 MHz, DMSO-d_6) δ 172.89 (d, J = 5.5 Hz), 164.67, 140.18 (d, J = 9.1 Hz), 138.52 (d, J = 11.4 Hz), 137.65 (d, J = 11.5 Hz), 135.65 (d, J = 98.6 Hz), 133.70, 133.13 (d, J = 98.5 Hz), 132.64 (d, overlapped), 131.86, 131.86 (d, overlapped),131.15 (d, J = 9.1 Hz), 130.66 (d, J = 9.1 Hz), 129.03 (d, J = 12.1 Hz), 128.62, 128.29 (d, J = 12.7 Hz, overlapped), 128.25, 127.91 (d, J = 10.0 Hz), 127.66, 127.52 (d, J = 9.4 Hz), 126.96, 126.45, 62.53 (d, J = 4.9 Hz), 34.13 (d, J = 70.5 Hz), 21.42, 21.16.

^{31}**P NMR** (162 MHz, DMSO-d_6) δ 26.90.

HRMS (ESI+): calculated m/z $[M+H]^+$ for $[C_{30}H_{28}NO_4PH]^+$: 498.1829, found: 498.1824.

2-benzamido-3-(bis(4-methoxyphenyl)phosphoryl)-2-phenylpropanoic acid (3ad)

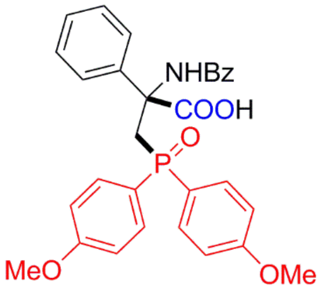

94.2 mg, 0.178 mmol, 89%;

White solid;

Mp: 196 - 197 ℃;

^{1}**H NMR** (400 MHz, DMSO-d_6) δ 13.42 (s, 1H), 8.47 (s, 1H), 7.75 – 7.66 (m, 2H), 7.56 – 7.35 (m, 9H), 7.28 – 7.13 (m, 3H), 7.09 – 7.01 (m, 2H), 6.73 – 6.64 (m, 2H), 3.98 (dd, J = 14.9, 6.6 Hz, 1H), 3.79 (s, 3H), 3.61 (s, 3H), 3.58 – 3.46 (m, 1H).

^{13}**C NMR** (101 MHz, DMSO-d_6) δ 172.91 (d, J = 5.5 Hz), 164.62, 162.07 (d, J = 2.6 Hz), 161.58 (d, J = 3.0 Hz), 140.37 (d, J = 9.1 Hz), 133.74, 132.65 (d, J = 11.1 Hz), 132.31 (d, J = 10.6 Hz), 131.84, 128.57, 128.31, 127.59, 127.09 (d, J = 105.3 Hz), 127.00, 126.43, 124.24 (d, J = 105.8 Hz), 114.61 (d, J = 12.3 Hz), 113.92 (d, J = 12.7 Hz), 62.54 (d, J = 5.0 Hz), 55.75, 55.43, 34.21 (d, J = 72.3 Hz).

^{31}**P NMR** (162 MHz, DMSO-d_6) δ 27.04.

HRMS (ESI+): calculated m/z $[M+H]^+$ for $[C_{30}H_{28}NO_6PH]^+$: 530.1727, found: 530.1726.

2-benzamido-3-(di([1,1'-biphenyl]-4-yl)phosphoryl)-2-phenylpropanoic acid (3ae)

111.8 mg, 0.180 mmol, 90%;

White solid;
Mp: 222 - 224 ℃;

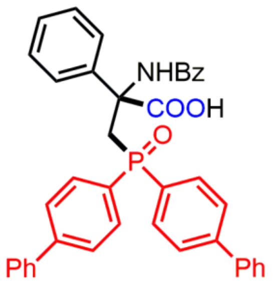

^{1}H NMR (400 MHz, DMSO-d_6) δ 13.61 (s, 1H), 8.46 (s, 1H), 7.97 (dd, *J* = 11.1, 8.2 Hz, 2H), 7.82 (dd, *J* = 8.3, 2.4 Hz, 2H), 7.78 – 7.68 (m, 4H), 7.55 – 7.36 (m, 15H), 7.27 (q, *J* = 8.0 Hz, 4H), 7.22 – 7.17 (m, 1H), 4.25 (dd, *J* = 14.9, 6.4 Hz, 1H), 3.72 (t, *J* = 14.5 Hz, 1H).

^{13}C NMR (101 MHz, DMSO-d_6) δ 172.93 (d, *J* = 5.1 Hz), 164.82, 143.68 (d, *J* = 2.5 Hz), 143.17 (d, *J* = 2.6 Hz), 140.41 (d, *J* = 9.3 Hz), 139.62 (d, *J* = 16.5 Hz), 134.42 (d, *J* = 99.9 Hz), 133.79, 131.96 (d, *J* = 100.3 Hz), 131.68 (d, *J* = 24.3 Hz), 131.13 (d, *J* = 9.5 Hz), 129.51, 129.29, 128.67, 128.48 (d, *J* = 16.2 Hz), 127.66, 127.45 (d, *J* = 11.8 Hz), 127.43, 127.36, 126.92, 126.79 (d, *J* = 12.0 Hz), 126.44, 62.54 (d, *J* = 5.0 Hz), 34.07 (d, *J* = 71.4 Hz).

^{31}P NMR (162 MHz, DMSO-d_6) δ 26.66.

HRMS (ESI+): calculated m/z $[M+H]^+$ for $[C_{40}H_{32}NO_4PH]^+$: 622.2142, found: 622.2140.

2-benzamido-3-(bis(4-chlorophenyl)phosphoryl)-2-phenylpropanoic acid (3af)

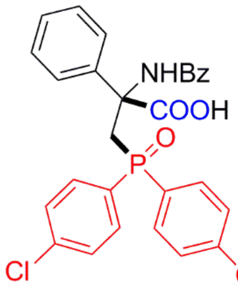

79.5 mg, 0.148 mmol, 74%;
White solid;
Mp: 192 - 193 ℃;

^{1}H NMR (400 MHz, DMSO-d_6) δ 13.57 (s, 1H), 8.34 (s, 1H), 7.90 – 7.83 (m, 2H), 7.68 – 7.56 (m, 4H), 7.54 – 7.49 (m, 3H), 7.45 (d, *J* = 7.6 Hz, 2H), 7.40 (d, *J* = 7.4 Hz, 2H), 7.32 – 7.17 (m, 5H), 4.20 (dd, *J* = 14.3, 5.5 Hz, 2H), 3.72 – 3.64 (m, 1H).

^{13}C NMR (101 MHz, DMSO-d_6) δ 172.81 (d, *J* = 4.9 Hz), 164.68, 140.31 (d, *J* = 9.8 Hz), 137.41 (d, *J* = 3.1 Hz), 136.93 (d, *J* = 3.4 Hz), 134.04 (d, *J* = 99.9 Hz), 133.36, 132.78 (d, *J* = 10.6 Hz), 132.44 (d, *J* = 10.3 Hz), 132.01, 131.77 (d, *J* = 100.0 Hz), 129.33 (d, *J* = 12.0 Hz), 128.61 (overlapped), 128.55 (d, *J* = 12.3 Hz), 128.45, 127.76, 126.90, 126.34, 62.32 (d, *J* = 5.0 Hz), 33.69 (d, *J* = 72.4 Hz).

^{31}P NMR (162 MHz, DMSO-d_6) δ 25.98.

HRMS (ESI+): calculated m/z $[M+H]^+$ for $[C_{28}H_{22}Cl_2NO_4PH]^+$: 538.0736, found: 538.0732.

2-benzamido-3-(diethoxyphosphoryl)-2-phenylpropanoic acid (**3ag**)

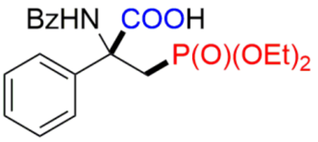

63.2 mg, 0.156 mmol, 78%;
White solid;
Mp: 134 - 136 ℃;

^{1}H NMR (400 MHz, DMSO-d_6) δ 13.59 (s, 1H), 8.24 (s, 1H), 7.86 – 7.77 (m, 2H), 7.61 – 7.56 (m, 1H), 7.56 – 7.45 (m, 4H), 7.38 – 7.31 (m, 2H), 7.32 – 7.26 (m, 1H), 4.00 – 3.89 (m, 2H), 3.86 – 3.70 (m, 2H), 3.44 – 3.33 (m, 2H), 3.15 (dd, *J* = 18.7, 15.2 Hz, 1H), 1.13 (t, *J* = 7.0 Hz, 3H), 0.97 (t, *J* = 7.0 Hz, 3H).

^{13}C NMR (101 MHz, DMSO-d_6) δ 172.82 (d, *J* = 6.0 Hz), 165.38, 140.40 (d, *J* = 13.7 Hz), 134.65, 132.09, 129.07, 128.63, 127.89, 127.33, 126.23 (d, *J* = 1.4 Hz), 61.85 (d, *J* = 4.6 Hz), 61.65 (d, *J* = 6.1 Hz), 61.51 (d, J = 6.7 Hz), 30.12 (d, *J* = 139.8 Hz), 16.47 (d, *J* = 6.1 Hz), 16.26 (d, *J* = 6.2 Hz).

^{31}P NMR (162 MHz, DMSO-d_6) δ 26.28.

HRMS (ESI+): calculated m/z $[M+H]^+$ for $[C_{20}H_{24}NO_6PH]^+$: 406.1414, found: 406.1413.

2-benzamido-3-(diisopropoxyphosphoryl)-2-phenylpropanoic acid (**3ah**)

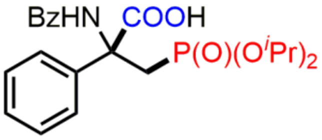

72.8 mg, 0.168 mmol, 84%;
White solid;
Mp: 138 - 140 ℃;
^{1}H NMR (400 MHz, DMSO-d_6) δ 13.57 (s, 1H), 8.23 (s, 1H), 7.89 – 7.76 (m, 2H), 7.61 – 7.56 (m, 1H), 7.56 – 7.46 (m, 4H), 7.38 – 7.31 (m, 2H), 7.30 – 7.25 (m, 1H), 4.53 (dp, *J* = 7.7, 6.1 Hz, 1H), 4.40 (dp, *J* = 7.6, 6.1 Hz, 1H), 3.30 (dd, *J* = 17.9, 15.1 Hz, 1H), 3.08 (dd, *J* = 18.8, 15.1 Hz, 1H), 1.16 (dd, *J* = 9.8, 6.2 Hz, 6H), 1.10 (d, *J* = 6.1 Hz, 3H), 0.94 (d, *J* = 6.2 Hz, 3H).
^{13}C NMR (101 MHz, DMSO-d_6) δ 172.89 (d, *J* = 6.0 Hz), 165.08, 140.53 (d, *J* = 13.7 Hz), 134.69, 132.07, 129.04, 128.61, 127.82, 127.31, 126.21, 70.25 (d, *J* = 6.1 Hz), 69.87 (d, *J* = 6.9 Hz), 61.91 (d, *J* = 5.0 Hz), 31.74 (d, *J* = 141.7 Hz), 24.13 (d, *J* = 3.9 Hz), 23.97 (d, *J* = 4.0 Hz), 23.81 (d, *J* = 4.7 Hz).
^{31}P NMR (162 MHz, DMSO-d_6) δ 24.54.
HRMS (ESI+): calculated m/z $[M+H]^+$ for $[C_{22}H_{28}NO_6PH]^+$: 434.1727, found: 434.1729.

2-benzamido-3-(dibutoxyphosphoryl)-2-phenylpropanoic acid (3ai)

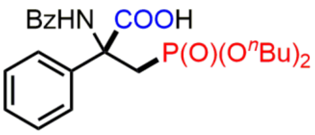

74.7 mg, 0.162 mmol, 81%;
White solid;
Mp: 122 - 124 ℃;
^{1}H NMR (400 MHz, DMSO-d_6) δ 13.58 (s, 1H), 8.25 (s, 1H), 7.87 – 7.77 (m, 2H), 7.62 – 7.56 (m, 1H), 7.56 – 7.47 (m, 4H), 7.38 – 7.31 (m, 2H), 7.31 – 7.25 (m, 1H), 3.93 – 3.83 (m, 2H), 3.81 – 3.73 (m, 1H), 3.71 – 3.63 (m, 1H), 3.38 (dd, *J* = 17.6, 15.2 Hz, 1H), 3.16 (dd, *J* = 18.7, 15.2 Hz, 1H), 1.53 – 1.39 (m, 2H), 1.32 – 1.21 (m, 4H), 1.17 – 1.06 (m, 2H), 0.81 (t, *J* = 7.4 Hz, 3H), 0.71 (t, *J* = 7.3 Hz, 3H).
^{13}C NMR (101 MHz, DMSO-d_6) δ 172.83 (d, *J* = 5.9 Hz), 165.26, 140.41 (d, *J* = 13.7 Hz), 134.56, 132.09, 129.03, 128.99, 128.63, 127.88, 127.33, 126.21, 65.21 (d, *J* = 6.2 Hz), 65.03 (d, *J* = 6.9 Hz), 61.85 (d, *J* = 4.6 Hz), 32.25 (d, *J* = 6.1 Hz), 32.11 (d, *J* = 6.3 Hz), 30.09 (d, *J* = 139.6 Hz), 18.63, 18.52, 13.86, 13.76.
^{31}P NMR (162 MHz, DMSO-d_6) δ 26.51.
HRMS (ESI+): calculated m/z $[M+H]^+$ for $[C_{24}H_{32}NO_6PH]^+$: 462.2040, found: 462.2043.

2-benzamido-3-(di-tert-butoxyphosphoryl)-2-phenylpropanoic acid (3aj)

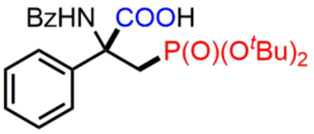

70.1 mg, 0.152 mmol, 76%;
White solid;
Mp: 122 - 124 ℃;
^{1}H NMR (400 MHz, DMSO-d_6) δ 13.35 (s, 1H), 8.22 (s, 1H), 7.85 – 7.79 (m, 2H), 7.58 – 7.51 (m, 3H), 7.49 – 7.45 (m, 2H), 7.36 – 7.31 (m, 2H), 7.29 – 7.24 (m, 1H), 3.23 (dd, *J* = 17.6, 14.9 Hz, 1H), 3.03 (dd, *J* = 18.8, 15.0 Hz, 1H), 1.34 (s, 9H), 1.29 (s, 9H).
^{13}C NMR (101 MHz, DMSO-d_6) δ 173.06 (d, *J* = 5.5 Hz), 164.86, 140.93 (d, *J* = 13.8 Hz), 134.83, 132.03, 129.07, 128.59, 127.70, 127.26, 126.20, 82.16 (d, *J* = 9.1 Hz), 82.13 (d, *J* = 8.0 Hz), 61.96 (d, *J* = 5.9 Hz), 34.44 (d, *J* = 145.1 Hz), 30.20 (d, *J* = 4.1 Hz), 30.07 (d, *J* = 3.8 Hz).

^{31}**P NMR** (162 MHz, DMSO-d_6) δ 18.00.

HRMS (ESI-): calculated m/z $[M-H]^-$ for $[C_{24}H_{31}NO_6P]^-$: 460.1894, found: 460.1889.

2-benzamido-2-phenyl-3-(4,4,5,5-tetramethyl-2-oxido-1,3,2-dioxaphospholan-2-yl)propanoic acid (3ak)

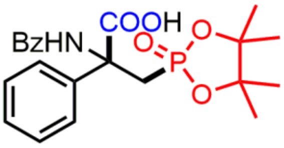

38.8 mg, 0.090 mmol, 45%;

White solid;

Mp: 196 - 197 ℃;

^{1}**H NMR** (400 MHz, DMSO-d_6) δ 13.71 (s, 1H), 8.26 (s, 1H), 7.84 – 7.74 (m, 2H), 7.58 (t, *J* = 7.2 Hz, 1H), 7.52 (dd, *J* = 8.3, 6.6 Hz, 4H), 7.34 (t, *J* = 7.5 Hz, 2H), 7.27 (t, *J* = 7.2 Hz, 1H), 3.50 – 3.36 (m, 2H), 1.32 (d, *J* = 15.7 Hz, 6H), 1.18 (d, *J* = 13.9 Hz, 6H).

^{13}**C NMR** (101 MHz, DMSO-d_6) δ 172.58 (d, *J* = 4.3 Hz), 165.81, 135.21, 131.91, 129.01, 128.63, 127.81, 127.30, 126.16, 88.34 (d, *J* = 1.5 Hz), 88.03 (d, *J* = 0.6 Hz), 62.03 (d, *J* = 4.7 Hz), 33.82 (d, *J* = 132.7 Hz), 24.92 (d, *J* = 3.0 Hz), 24.52 (d, *J* = 4.6 Hz), 23.68 (d, *J* = 5.8 Hz), 23.60 (d, *J* = 4.1 Hz).

^{31}**P NMR** (162 MHz, DMSO-d_6) δ 37.75.

HRMS (ESI+): calculated m/z $[M+H]^+$ for $[C_{22}H_{26}NO_6PH]^+$: 432.1571, found: 432.1570.

methyl 2-benzamido-3-(dimethylphosphoryl)-2-phenylpropanoate (3al)

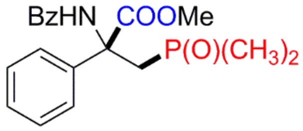

38.7 mg, 0.112 mmol, 56%;

White solid;

Mp: 196 - 197 ℃;

^{1}**H NMR** (400 MHz, DMSO-d_6) δ 9.83 (s, 1H), 7.94 – 7.87 (m, 2H), 7.65 – 7.59 (m, 1H), 7.57 – 7.51 (m, 2H), 7.48 – 7.43 (m, 2H), 7.41 – 7.30 (m, 3H), 3.63 (s, 3H), 3.05 – 2.86 (m, 2H), 1.48 (d, *J* = 13.4 Hz, 3H), 0.77 (d, *J* = 13.6 Hz, 3H).

^{13}**C NMR** (101 MHz, DMSO-d_6) δ 171.60 (d, *J* = 12.6 Hz), 165.67, 138.23 (d, *J* = 3.8 Hz), 133.51, 132.46, 129.19, 128.31, 128.23, 127.74, 127.53, 63.57 (d, *J* = 5.3 Hz), 53.16, 37.68 (d, *J* = 64.2 Hz), 18.11 (d, *J* = 69.4 Hz), 16.43 (d, *J* = 67.5 Hz).

^{31}**P NMR** (162 MHz, DMSO-d_6) δ 47.85.

HRMS (ESI+): calculated m/z $[M+H]^+$ for $[C_{18}H_{20}NO_4PH]^+$: 346.1203, found: 346.1961.

3-(diphenylphosphoryl)-2-phenylpropanoic acid (9aa)

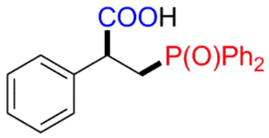

56.7 mg, 0.162 mmol, 81%;

White solid;

Mp: 175 - 177 ℃;

^{1}**H NMR** (400 MHz, DMSO-d_6) δ 12.50 (s, 1H), 7.87 – 7.77 (m, 2H), 7.77 – 7.66 (m, 2H), 7.59 – 7.45 (m, 4H), 7.45 – 7.37 (m, 2H), 7.29 – 7.12 (m, 5H), 3.90 – 3.74 (m, 1H), 3.29 – 3.14 (m, 1H), 2.97 – 2.79 (m, 1H).

^{13}**C NMR** (101 MHz, DMSO-d_6) δ 173.90 (d, *J* = 6.7 Hz), 139.68 (d, *J* = 9.3 Hz), 134.53 (d, *J* = 97.7 Hz), 133.80 (d, *J* = 97.0 Hz), 132.04 (d, *J* = 2.7 Hz), 131.82 (d, *J* = 2.7 Hz), 130.90 (d, *J* = 9.0 Hz), 130.82 (d, *J* = 9.1 Hz), 129.00 (d, *J* = 11.5 Hz), 128.87 (d, *J* = 11.4 Hz), 128.84, 128.19, 127.58, 45.10 (d, *J* = 2.1 Hz), 32.74 (d, *J* = 70.1 Hz).

^{31}**P NMR** (162 MHz, DMSO-d_6) δ 27.89.

HRMS (ESI-): calculated m/z $[M-H]^-$ for $[C_{21}H_{18}O_3P]^-$: 349.0999, found: 349.0999.

3-(diphenylphosphoryl)-2-(*p*-tolyl)propanoic acid (9ba)

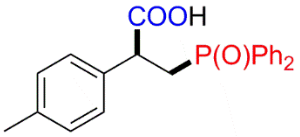

59.7 mg, 0.164 mmol, 82%;

White solid;

Mp: 167 - 169 ℃;

^{1}H NMR (400 MHz, DMSO-d_6) δ 12.44 (s, 1H), 7.85 – 7.77 (m, 2H), 7.74 – 7.67 (m, 2H), 7.58 – 7.46 (m, 4H), 7.45 – 7.38 (m, 2H), 7.08 (d, *J* = 8.1 Hz, 2H), 7.01 (d, *J* = 7.9 Hz, 2H), 3.78 (ddd, *J* = 11.9, 8.3, 5.3 Hz, 1H), 3.20 (ddd, *J* = 15.2, 12.3, 8.4 Hz, 1H), 2.82 (ddd, *J* = 14.9, 9.0, 5.4 Hz, 1H), 2.21 (s, 3H).

^{13}C NMR (101 MHz, DMSO-d_6) δ 174.02 (d, *J* = 6.8 Hz), 136.72, 136.70 (d, *J* = 9.4 Hz), 134.51 (d, *J* = 97.5 Hz), 133.81 (d, *J* = 97.0 Hz), 132.03 (d, *J* = 2.8 Hz), 131.71 (d, *J* = 2.7 Hz), 130.90 (d, *J* = 9.3 Hz), 130.82 (d, *J* = 9.3 Hz), 129.36, 128.99 (d, *J* = 11.4 Hz), 128.85 (d, *J* = 11.5 Hz), 128.04, 44.67 (d, *J* = 2.0 Hz), 32.86 (d, *J* = 70.0 Hz), 21.01.

^{31}P NMR (162 MHz, DMSO-d_6) δ 27.94.

HRMS (ESI+): calculated m/z $[M+H]^+$ for $[C_{22}H_{19}O_3PH]^+$: 363.1156, found: 363.1157.

2-(4-(*tert*-butyl)phenyl)-3-(diphenylphosphoryl)propanoic acid (9ca)

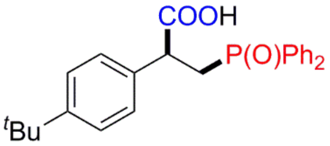

52.8 mg, 0.130 mmol, 65%;

White solid;

Mp: 175 - 176 ℃;

^{1}H NMR (400 MHz, $CDCl_3$) δ 9.56 (s, 1H), 7.75 – 7.52 (m, 4H), 7.51 – 7.23 (m, 8H), 7.22 – 7.12 (m, 2H), 4.36 – 4.13 (m, 1H), 3.56 – 3.34 (m, 1H), 2.83 – 2.60 (m, 1H), 1.25 (s, 9H).

^{13}C NMR (101 MHz, $CDCl_3$) δ 174.24 (d, *J* = 7.3 Hz), 150.12, 135.48 (d, *J* = 10.1 Hz), 132.37 (d, *J* = 102.3 Hz, overlapped), 132.09 (d, *J* = 2.4 Hz), 131.83 (d, *J* = 2.4 Hz), 131.03 (d, *J* = 9.5 Hz), 130.57 (d, *J* = 9.8 Hz), 130.36 (d, *J* = 101.2 Hz), 128.68 (d, *J* = 12.0 Hz), 128.54 (d, *J* = 12.1 Hz), 127.76, 125.43, 44.64 (d, *J* = 1.2 Hz), 34.38, 33.65 (d, *J* = 70.6 Hz), 31.31.

^{31}P NMR (162 MHz, $CDCl_3$) δ 33.84.

HRMS (ESI+): calculated m/z $[M+H]^+$ for $[C_{25}H_{27}O_3PH]^+$: 407.1771, found: 407.1772.

3-(diphenylphosphoryl)-2-(4-fluorophenyl)propanoic acid (9da)

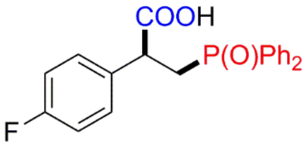

51.5 mg, 0.140 mmol, 70%;

White solid;

Mp: 164- 166 ℃;

^{1}H NMR (400 MHz, $CDCl_3$) δ 9.27 (s, 1H), 7.71 – 7.63 (m, 2H), 7.61 – 7.54 (m, 2H), 7.52 – 7.45 (m, 2H), 7.44 – 7.34 (m, 4H), 7.34 – 7.28 (m, 2H), 6.86 – 6.79 (m, 2H), 4.30 – 4.19 (m, 1H), 3.51 – 3.36 (m, 1H), 2.74 – 2.63 (m, 1H).

^{13}C NMR (101 MHz, $CDCl_3$) δ 174.00 (d, J_{C-P} = 6.7 Hz), 162.09 (d, J_{C-F} = 246.0 Hz), 134.26 (dd, J_{C-P} = 9.6 Hz, J_{C-F} = 3.0 Hz), 132.29 (d, J_{C-P} = 2.6 Hz), 132.02 (d, J_{C-P} = 102.3 Hz), 132.00 (d, J_{C-P} = 2.8 Hz), 130.94 (d, J_{C-P} = 9.6 Hz), 130.53 (d, J_{C-P} = 9.9 Hz), 130.07 (d, J_{C-P} = 101.0 Hz), 129.79 (d, J_{C-F} = 8.1 Hz), 128.78 (d, J_{C-P} = 11.9 Hz), 128.66 (d, J_{C-P} = 11.8 Hz), 115.32 (d, J_{C-F} = 21.4 Hz), 44.42 (d, J_{C-P} = 1.5 Hz), 33.79 (d, J_{C-P} = 70.4 Hz).

^{31}P NMR (162 MHz, $CDCl_3$) δ 34.01.

^{19}**F NMR** (376 MHz, $CDCl_3$) δ -115.10.
HRMS (ESI-): calculated m/z [M-H]$^-$ for $[C_{21}H_{17}FO_3P]^-$: 367.0905, found: 367.0903.

2-(4-chlorophenyl)-3-(diphenylphosphoryl)propanoic acid (9ea)

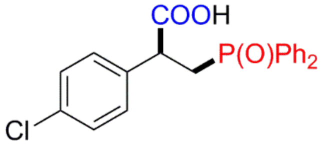

53.9 mg, 0.140 mmol, 70%;
White solid;
Mp: 171 - 173 ℃;
^{1}**H NMR** (400 MHz, DMSO-d_6) δ 12.64 (s, 1H), 7.85 – 7.77 (m, 2H), 7.71 – 7.62 (m, 2H), 7.60 – 7.44 (m, 4H), 7.42 – 7.35 (m, 2H), 7.26 – 7.18 (m, 4H), 3.91 – 3.80 (m, 1H), 3.22 – 3.09 (m, 1H), 3.04 – 2.89 (m, 1H).
^{13}**C NMR** (101 MHz, DMSO-d_6) δ 173.76 (d, J = 8.9 Hz), 138.27 (d, J = 7.9 Hz), 134.53 (d, J = 97.7 Hz), 133.68 (d, J = 97.2 Hz), 132.30, 132.12 (d, J = 2.7 Hz), 131.71 (d, J = 2.7 Hz), 130.92 (d, J = 9.2 Hz), 130.81 (d, J = 9.5 Hz), 130.32, 129.07 (d, J = 11.5 Hz), 128.82 (d, J = 11.5 Hz), 128.67, 44.63, 32.59 (d, J = 70.6 Hz).
^{31}**P NMR** (162 MHz, CDCl3) δ 33.93.
HRMS (ESI+): calculated m/z [M+H]$^+$ for $[C_{21}H_{18}ClO_3PH]^+$: 385.0755, found: 385.0751.

2-(4-bromophenyl)-3-(diphenylphosphoryl)propanoic acid (9fa)

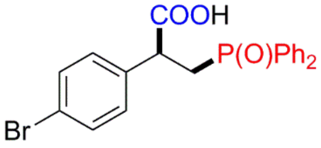

61.8 mg, 0.144 mmol, 72%;
White solid;
Mp: 173 - 175 ℃;
^{1}**H NMR** (400 MHz, DMSO-d_6) δ 12.63 (s, 1H), 7.89 – 7.75 (m, 2H), 7.74 – 7.61 (m, 2H), 7.60 – 7.43 (m, 4H), 7.43 – 7.30 (m, 4H), 7.24 – 7.08 (m, 2H), 3.94 – 3.74 (m, 1H), 3.22 – 3.10 (m, 1H), 3.01 – 2.90 (m, 1H).
^{13}**C NMR** (101 MHz, DMSO-d_6) δ 173.64 (d, J = 8.8 Hz), 138.66 (d, J = 7.8 Hz), 134.48 (d, J = 97.8 Hz), 133.65 (d, J = 97.1 Hz), 132.08 (d, J = 2.6 Hz), 131.65 (d, J = 2.3 Hz), 131.56, 130.87 (d, J = 10.0 Hz), 130.77 (d, J = 10.0 Hz), 130.62, 129.03 (d, J = 11.4 Hz), 128.79 (d, J = 11.4 Hz), 120.83, 44.67 (d, J = 1.7 Hz), 32.53 (d, J = 70.4 Hz).
^{31}**P NMR** (162 MHz, DMSO-d_6) δ 27.67.
HRMS (ESI+): calculated m/z [M+H]$^+$ for $[C_{21}H_{18}BrO_3PH]^+$: 429.0250, found: 429.0252.

3-(diphenylphosphoryl)-2-(4-(trifluoromethyl)phenyl)propanoic acid (9ga)

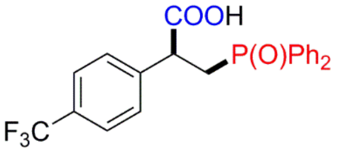

67.7 mg, 0.162 mmol, 93%;
White solid;
Mp: 161 - 162 ℃;
^{1}**H NMR** (400 MHz, DMSO-d_6) δ 12.78 (s, 1H), 7.87 – 7.77 (m, 2H), 7.69 – 7.59 (m, 2H), 7.59 – 7.47 (m, 5H), 7.46 – 7.38 (m, 3H), 7.38 – 7.29 (m, 2H), 4.04 – 3.91 (m, 1H), 3.24 – 3.11 (m, 1H), 3.13 – 3.00 (m, 1H).
^{13}**C NMR** (101 MHz, DMSO-d_6) δ 173.47 (d, J_{C-P} = 9.8 Hz), 143.69 (d, J_{C-P} = 7.4 Hz), 134.50 (d, J_{C-P} = 98.1 Hz), 133.49 (d, J_{C-P} = 97.3 Hz), 132.10 (d, J_{C-P} = 2.6 Hz), 131.65 (d, J_{C-P} = 2.8 Hz), 130.86 (d, J_{C-P} = 9.4 Hz), 130.73 (d, J_{C-P} = 9.3 Hz), 129.43, 129.05 (d, J_{C-P} = 11.5 Hz), 128.69 (d, J_{C-P} = 11.5 Hz), 128.17 (q, J_{C-F} = 31.7 Hz), 125.49 (q, J_{C-F} = 3.8 Hz), 124.56 (q, J_{C-F} = 272.0 Hz), 45.15 (d, J_{C-P} = 2.0 Hz), 32.42 (d, J_{C-P} = 70.7 Hz).
^{31}**P NMR** (162 MHz, DMSO-d_6) δ 27.63.

^{19}**F NMR** (376 MHz, DMSO-d_6) δ -61.14.
HRMS (ESI+): calculated m/z $[M+H]^+$ for $[C_{22}H_{18}F_3O_3PH]^+$: 419.1018, found: 419.1016.

2-([1,1'-biphenyl]-4-yl)-3-(diphenylphosphoryl)propanoic acid (9ha)

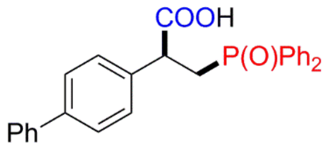

52.9 mg, 0.124 mmol, 62%;
White solid;
Mp: 205 - 207 ℃;
^{1}**H NMR** (400 MHz, DMSO-d_6) δ 12.58 (s, 1H), 7.87 – 7.80 (m, 2H), 7.74 – 7.68 (m, 2H), 7.60 – 7.42 (m, 10H), 7.42 – 7.32 (m, 3H), 7.29 (d, *J* = 7.9 Hz, 2H), 3.94 – 3.83 (m, 1H), 3.23 (ddd, *J* = 15.2, 12.5, 7.8 Hz, 1H), 2.96 (ddd, *J* = 15.1, 8.7, 5.9 Hz, 1H).
^{13}**C NMR** (101 MHz, DMSO-d_6) δ 173.91 (d, *J* = 7.7 Hz), 140.32, 139.56, 138.69 (d, *J* = 8.7 Hz), 134.59 (d, *J* = 97.6 Hz), 133.74 (d, *J* = 97.1 Hz), 132.06 (d, *J* = 2.6 Hz), 131.66 (d, *J* = 2.6 Hz), 130.93 (d, *J* = 9.3 Hz), 130.81 (d, *J* = 9.3 Hz), 129.34, 129.03 (d, *J* = 11.5 Hz), 128.86, 128.81 (d, *J* = 11.3 Hz), 127.81, 127.15, 127.05, 44.84 (d, *J* = 2.2 Hz), 32.76 (d, *J* = 70.2 Hz).
^{31}**P NMR** (162 MHz, DMSO-d_6) δ 27.88.
HRMS (ESI+): calculated m/z $[M+H]^+$ for $[C_{27}H_{23}O_3PH]^+$: 427.1458, found: 427.1462.

3-(diphenylphosphoryl)-2-(4'-methoxy-[1,1'-biphenyl]-4-yl)propanoic acid (9ia)

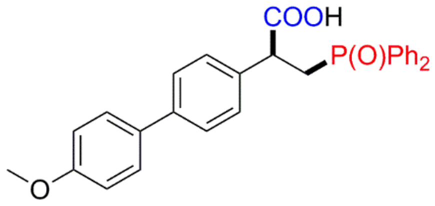

65.7 mg, 0.144 mmol, 72%;
White solid;
Mp: 152 - 154 ℃;
^{1}**H NMR** (400 MHz, DMSO-d_6) δ 12.52 (s, 1H), 7.89 – 7.78 (m, 2H), 7.74 – 7.67 (m, 2H), 7.60 – 7.49 (m, 5H), 7.49 – 7.36 (m, 5H), 7.28 – 7.22 (m, 2H), 7.04 – 6.99 (m, 2H), 3.89 – 3.81 (m, 1H), 3.79 (s, 3H), 3.29 – 3.14 (m, 1H), 2.99 – 2.88 (m, 1H).
^{13}**C NMR** (101 MHz, DMSO-d_6) δ 173.93 (d, *J* = 7.5 Hz), 159.30, 139.23, 137.94 (d, *J* = 8.8 Hz), 134.57 (d, *J* = 97.6 Hz), 133.77 (d, *J* = 97.2 Hz), 132.65, 132.05 (d, *J* = 2.8 Hz), 131.67 (d, *J* = 2.7 Hz), 130.92 (d, *J* = 9.4 Hz), 130.81 (d, *J* = 9.4 Hz), 129.02 (d, *J* = 11.4 Hz), 128.81 (d, *J* = 11.7 Hz), 128.76, 128.11, 126.64, 114.78, 55.59, 44.77 (d, *J* = 2.0 Hz), 32.77 (d, *J* = 70.2 Hz).
^{31}**P NMR** (162 MHz, DMSO-d_6) δ 27.91.
HRMS (ESI+): calculated m/z $[M+H]^+$ for $[C_{28}H_{25}O_4PH]^+$: 457.1563, found: 457.1562.

3-(diphenylphosphoryl)-2-(4'-(trifluoromethyl)-[1,1'-biphenyl]-4-yl)propanoic acid (9ja)

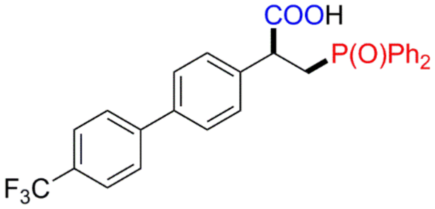

90.0 mg, 0.182 mmol, 91%;
White solid;
Mp: 207 - 208 ℃;
^{1}**H NMR** (400 MHz, DMSO-d_6) δ 12.62 (s, 1H), 7.89 – 7.74 (m, 6H), 7.74 – 7.64 (m, 2H), 7.59 – 7.48 (m, 5H), 7.47 – 7.41 (m, 1H), 7.40 – 7.30 (m, 4H), 3.99 – 3.86 (m, 1H), 3.22 (ddd, *J* = 15.2, 12.7, 7.5 Hz, 1H), 3.00 (ddd, *J* = 15.1, 8.6, 6.4 Hz, 1H).
^{13}**C NMR** (101 MHz, DMSO-d_6) δ 173.83 (d, J_{C-P} = 8.4 Hz), 144.28 (q, J_{C-F} = 1.9 Hz), 139.66 (d, J_{C-P} = 8.2 Hz), 137.92, 134.56 (d, J_{C-P} = 97.7 Hz), 133.66 (d, J_{C-P} = 97.2 Hz), 132.07 (d, J_{C-P} = 2.5 Hz), 131.61 (d, J_{C-P} = 2.7 Hz), 130.92 (d, J_{C-P} = 9.4 Hz), 130.79 (d, J_{C-P} = 9.4 Hz), 129.13, 129.03

(d, J_{C-P} = 11.4 Hz), 128.76 (d, J_{C-P} = 11.4 Hz), 128.20 (q, J_{C-F} = 31.8 Hz), 127.80, 127.47, 126.19 (q, J_{C-F} = 3.7 Hz), 124.79 (q, J_{C-F} = 271.7 Hz), 44.89 (d, J_{C-P} = 2.0 Hz), 32.70 (d, J_{C-P} = 70.3 Hz).

^{31}P NMR (162 MHz, DMSO-d_6) δ 27.88.

HRMS (ESI+): calculated m/z $[M+H]^+$ for $[C_{28}H_{22}F_3O_3PH]^+$: 495.1331, found: 495.1328.

3-(diphenylphosphoryl)-2-(4-(thiophen-3-yl)phenyl)propanoic acid (9ka)

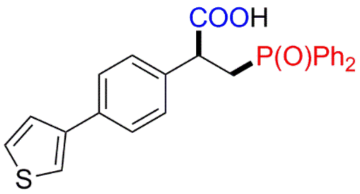

42.4 mg, 0.098 mmol, 49%;

White solid;

Mp: 192 - 194 ℃；

^{1}H NMR (400 MHz, DMSO-d_6) δ 12.53 (s, 1H), 7.88 – 7.77 (m, 3H), 7.77 – 7.67 (m, 2H), 7.66 – 7.59 (m, 1H), 7.60 – 7.42 (m, 7H), 7.45 – 7.35 (m, 2H), 7.23 (d, *J* = 8.1 Hz, 2H), 3.84 (ddd, *J* = 11.9, 8.1, 5.6 Hz, 1H), 3.23 (ddd, *J* = 15.4, 12.4, 8.2 Hz, 1H), 2.91 (ddd, *J* = 15.0, 9.0, 5.7 Hz, 1H).

^{13}C NMR (101 MHz, DMSO-d_6) δ 173.88 (d, *J* = 7.3 Hz), 141.55, 138.38 (d, *J* = 9.0 Hz), 134.58, 134.50 (d, *J* = 97.7 Hz), 133.80 (d, *J* = 97.2 Hz), 132.06 (d, *J* = 2.5 Hz), 131.75 (d, *J* = 2.6 Hz), 130.92 (d, *J* = 8.5 Hz), 130.84 (d, *J* = 9.1 Hz), 129.02 (d, *J* = 11.4 Hz), 128.86 (d, *J* = 11.4 Hz), 128.74, 127.49, 126.59, 126.56, 121.29, 44.80 (d, *J* = 1.6 Hz), 32.70 (d, *J* = 69.8 Hz).

^{31}P NMR (162 MHz, DMSO-d_6) δ 27.92.

HRMS (ESI+): calculated m/z $[M+H]^+$ for $[C_{25}H_{21}O_3PSH]^+$: 433.1022, found: 433.1021.

3-(diphenylphosphoryl)-2-(3-methoxyphenyl)propanoic acid (9la)

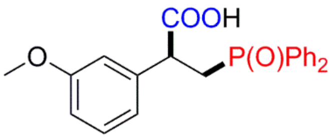

68.5 mg, 0.180 mmol, 90%;

White solid;

Mp: 133 - 134 ℃；

^{1}H NMR (400 MHz, DMSO-d_6) δ 12.53 (s, 1H), 7.86 – 7.76 (m, 2H), 7.76 – 7.66 (m, 2H), 7.60 – 7.43 (m, 4H), 7.45 – 7.36 (m, 2H), 7.17 – 7.08 (m, 1H), 6.82 – 6.69 (m, 3H), 3.88 – 3.73 (m, 1H), 3.68 (s, 3H), 3.18 (ddd, *J* = 15.1, 12.6, 8.1 Hz, 1H), 2.89 (ddd, *J* = 14.8, 8.7, 5.6 Hz, 1H).

^{13}C NMR (101 MHz, DMSO-d_6) δ 173.81 (d, *J* = 7.2 Hz), 159.56, 141.03 (d, *J* = 8.9 Hz), 134.57 (d, *J* = 97.7 Hz), 133.75 (d, *J* = 97.0 Hz), 132.04 (d, *J* = 2.6 Hz), 131.77 (d, *J* = 2.7 Hz), 130.88 (d, *J* = 9.0 Hz), 130.79 (d, *J* = 9.1 Hz), 129.87, 128.99 (d, *J* = 11.5 Hz), 128.79 (d, *J* = 11.4 Hz), 120.43, 113.88, 113.06, 55.38, 45.07 (d, *J* = 1.9 Hz), 32.71 (d, *J* = 70.0 Hz).

^{31}P NMR (162 MHz, DMSO-d_6) δ 27.93.

HRMS(ESI+): calculated m/z $[M+H]^+$ for $[C_{22}H_{21}O_4PH]^+$: 381.1250, found: 381.1243.

3-(diphenylphosphoryl)-2-(3-fluorophenyl)propanoic acid (9ma)

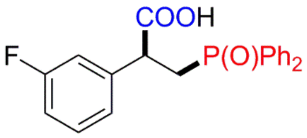

9.9 mg, 0.160 mmol, 80%;

White solid;

Mp: 175 - 177 ℃；

^{1}H NMR (400 MHz, DMSO-d_6) δ 12.66 (s, 1H), 7.86 – 7.78 (m, 2H), 7.73 – 7.66 (m, 2H), 7.59 – 7.43 (m, 4H), 7.43 – 7.34 (m, 2H), 7.28 – 7.17 (m, 1H), 7.09 – 7.00 (m, 2H), 7.02 – 6.92 (m, 1H), 3.95 – 3.83 (m, 1H), 3.17 (ddd, *J* = 15.3, 12.8, 7.3 Hz, 1H), 3.00 (ddd, *J* = 15.3, 8.7, 6.6 Hz, 1H).

^{13}C NMR (101 MHz, DMSO-d_6) δ 173.58 (d, $J_{C\text{-}P}$ = 8.6 Hz), 162.34 (d, $J_{C\text{-}F}$ = 243.5 Hz), 141.95 (dd, $J_{C\text{-}P}$ = 7.9 Hz, $J_{C\text{-}F}$ = 7.9 Hz), 134.53 (d, $J_{C\text{-}P}$ = 98.0 Hz), 133.66 (d, $J_{C\text{-}P}$ = 97.3 Hz), 132.07 (d, $J_{C\text{-}P}$ = 2.7 Hz), 131.78 (d, $J_{C\text{-}P}$ = 2.8 Hz), 130.87 (d, $J_{C\text{-}P}$ = 9.7 Hz), 130.77 (d, $J_{C\text{-}P}$ = 9.6 Hz), 130.60 (d, $J_{C\text{-}F}$ = 8.4 Hz), 129.02 (d, $J_{C\text{-}P}$ = 11.5 Hz), 128.75 (d, $J_{C\text{-}P}$ = 11.5 Hz), 124.64 (d, $J_{C\text{-}F}$ = 2.6 Hz), 115.20 (d, $J_{C\text{-}F}$ = 21.9 Hz), 114.39 (d, $J_{C\text{-}F}$ = 20.9 Hz), 44.90, 32.39 (d, $J_{C\text{-}P}$ = 70.4 Hz).
^{31}P NMR (162 MHz, DMSO-d_6) δ 27.72.
^{19}F NMR (376 MHz, DMSO-d_6) δ -113.24.
HRMS (ESI+): calculated m/z $[M+H]^+$ for $[C_{21}H_{18}FO_3PH]^+$: 369.1050, found: 369.1045.

2-(3-chlorophenyl)-3-(diphenylphosphoryl)propanoic acid (9na)

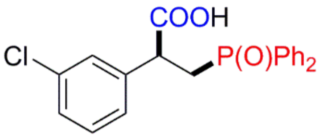

63.9 mg, 0.166 mmol, 83%;
White solid;
Mp: 164 - 166 ℃;
^{1}H NMR (400 MHz, DMSO-d_6) δ 12.67 (s, 1H), 7.88 – 7.77 (m, 2H), 7.73 – 7.64 (m, 2H), 7.59 – 7.42 (m, 4H), 7.42 – 7.34 (m, 2H), 7.25 (s, 1H), 7.23 – 7.12 (m, 3H), 3.94 – 3.83 (m, 1H), 3.16 (ddd, *J* = 15.4, 12.8, 7.1 Hz, 1H), 3.02 (ddd, *J* = 15.4, 8.6, 7.0 Hz, 1H).
^{13}C NMR (101 MHz, DMSO-d_6) δ 173.57 (d, *J* = 9.1 Hz), 141.49 (d, *J* = 7.9 Hz), 134.55 (d, *J* = 98.0 Hz), 133.62 (d, *J* = 97.2 Hz), 133.33, 132.07 (d, *J* = 2.7 Hz), 131.76 (d, *J* = 2.7 Hz), 130.84 (d, *J* = 9.3 Hz), 130.76 (d, *J* = 9.5 Hz), 130.52, 129.02 (d, *J* = 11.5 Hz), 128.71 (d, *J* = 11.5 Hz), 128.32, 127.56, 127.26, 44.88 (d, *J* = 2.3 Hz), 32.33 (d, *J* = 70.4 Hz).
^{31}P NMR (162 MHz, DMSO-d_6) δ 27.69.
HRMS (ESI+): calculated m/z $[M+H]^+$ for $[C_{21}H_{18}ClO_3PH]^+$: 385.0755, found: 385.0751.

2-(3-bromophenyl)-3-(diphenylphosphoryl)propanoic acid (9oa)

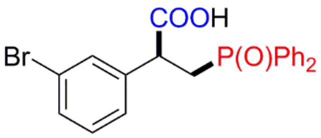

73.0 mg, 0.170 mmol, 85%;
White solid;
Mp: 177 - 180 ℃;
^{1}H NMR (400 MHz, DMSO-d_6) δ 12.69 (s, 1H), 7.86 – 7.78 (m, 2H), 7.72 – 7.64 (m, 2H), 7.60 – 7.29 (m, 8H), 7.20 (d, *J* = 7.5 Hz, 1H), 7.13 (t, *J* = 7.8 Hz, 1H), 3.93 – 3.82 (m, 1H), 3.21 – 3.09 (m, 1H), 3.07 – 2.94 (m, 1H).
^{13}C NMR (101 MHz, DMSO-d_6) δ 173.58 (d, *J* = 9.3 Hz), 141.74 (d, *J* = 7.9 Hz), 134.56 (d, *J* = 96.8 Hz), 133.60 (d, *J* = 96.9 Hz), 132.07 (d, *J* = 2.2 Hz), 131.76 (d, *J* = 2.4 Hz), 131.16, 130.83 (d, *J* = 9.4 Hz), 130.83, 130.76 (d, *J* = 9.4 Hz), 130.46, 129.01 (d, *J* = 11.4 Hz), 128.71 (d, *J* = 11.6 Hz), 127.64, 122.00, 44.86 (d, *J* = 0.6 Hz), 32.34 (d, *J* = 70.2 Hz).
^{31}P NMR (162 MHz, DMSO-d_6) δ 27.66.
HRMS (ESI+): calculated m/z $[M+H]^+$ for $[C_{21}H_{18}BrO_3PH]^+$: 429.0250, found: 429.0245.

2-(3-cyanophenyl)-3-(diphenylphosphoryl)propanoic acid (9pa)

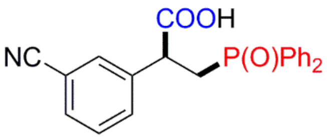

53.3 mg, 0.142 mmol, 71%;
White solid;
Mp: 175 - 177 ℃;
^{1}H NMR (400 MHz, DMSO-d_6) δ 12.79 (s, 1H), 7.85 – 7.77 (m,

2H), 7.67 – 7.60 (m, 3H), 7.59 – 7.49 (m, 5H), 7.46 – 7.40 (m, 1H), 7.39 – 7.31 (m, 3H), 4.05 – 3.93 (m, 1H), 3.21 – 3.08 (m, 2H).
^{13}C NMR (101 MHz, DMSO-d_6) δ 173.49 (d, J = 10.7 Hz), 140.34 (d, J = 6.4 Hz), 134.55 (d, J = 98.3 Hz), 133.79, 133.50 (d, J = 97.0 Hz), 132.36, 132.11 (d, J = 2.7 Hz), 131.70 (d, J = 2.7 Hz), 131.31, 130.83 (d, J = 9.4 Hz), 130.70 (d, J = 9.4 Hz), 129.78, 129.06 (d, J = 11.5 Hz), 128.66 (d, J = 11.5 Hz), 118.99, 111.61, 44.80 (d, J = 2.2 Hz), 32.04 (d, J = 70.9 Hz).
^{31}P NMR (162 MHz, CDCl3) δ 34.15.
HRMS (ESI+): calculated m/z $[M+H]^+$ for $[C_{22}H_{18}NO_3PH]^+$: 376.1097, found: 376.1100.

3-(diphenylphosphoryl)-2-(*o*-tolyl)propanoic acid (9qa)

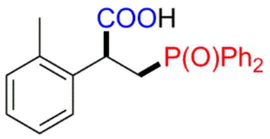

56.8 mg, 0.156 mmol, 78%;
White solid;
Mp: 160 - 163 ℃;
^{1}H NMR (400 MHz, DMSO-d_6) δ 12.45 (s, 1H), 7.87 – 7.79 (m, 2H), 7.74 – 7.65 (m, 2H), 7.59 – 7.44 (m, 4H), 7.42 – 7.36 (m, 2H), 7.26 – 7.22 (m, 1H), 7.09 – 6.98 (m, 3H), 4.06 (ddd, J = 13.0, 7.6, 5.6 Hz, 1H), 3.20 (ddd, J = 15.2, 12.8, 7.6 Hz, 1H), 2.87 – 2.78 (m, 1H), 2.08 (s, 3H).
^{13}C NMR (101 MHz, DMSO-d_6) δ 174.20 (d, J = 7.6 Hz), 138.28 (d, J = 8.2 Hz), 135.88, 134.51 (d, J = 97.3 Hz), 133.61 (d, J = 96.8 Hz), 132.08 (d, J = 2.7 Hz), 131.79 (d, J = 2.7 Hz), 130.89 (d, J = 9.1 Hz), 130.80 (d, J = 9.3 Hz), 130.55, 129.04 (d, J = 11.4 Hz), 128.76 (d, J = 11.4 Hz), 127.40, 127.33, 126.56, 40.28 (d, J = 1.9 Hz), 32.84 (d, J = 69.9 Hz).
^{31}P NMR (162 MHz, DMSO-d_6) δ 28.06.
HRMS (ESI+): calculated m/z $[M+H]^+$ for $[C_{22}H_{21}O_3PH]^+$: 365.1301, found: 365.1302.

2-(2-chlorophenyl)-3-(diphenylphosphoryl)propanoic acid (9ra)

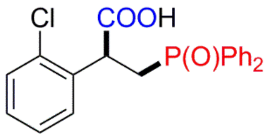

66.8 mg, 0.174 mmol, 87%;
White solid;
Mp: 172 - 175 ℃;
^{1}H NMR (400 MHz, DMSO-d_6) δ 12.70 (s, 1H), 7.85 – 7.79 (m, 2H), 7.72 – 7.65 (m, 2H), 7.59 – 7.43 (m, 4H), 7.41 – 7.36 (m, 3H), 7.29 – 7.26 (m, 1H), 7.22 – 7.14 (m, 2H), 4.40 – 4.28 (m, 1H), 3.26 – 3.12 (m, 1H), 3.00 – 2.87 (m, 1H).
^{13}C NMR (101 MHz, DMSO-d_6) δ 173.29 (d, J = 8.5 Hz), 137.17 (d, J = 7.6 Hz), 134.44 (d, J = 98.0 Hz), 133.47 (d, J = 97.0 Hz), 133.14, 132.10 (d, J = 2.7 Hz), 131.79 (d, J = 2.6 Hz), 130.90 (d, J = 9.2 Hz), 130.80 (d, J = 9.6 Hz), 130.19, 129.70, 129.16, 129.05 (d, J = 11.5 Hz), 128.73 (d, J = 11.5 Hz), 127.64, 41.55, 32.13 (d, J = 70.3 Hz).
^{31}P NMR (162 MHz, CDCl3) δ 33.33.
HRMS(ESI+): calculated m/z $[M+H]^+$ for $[C_{21}H_{18}ClO_3PH]^+$: 385.0755, found: 385.0750.

3-(diphenylphosphoryl)-2-(4-fluoro-3-phenoxyphenyl)propanoic acid (9sa)

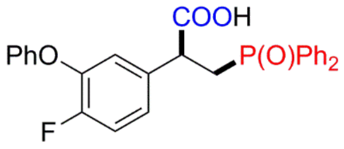

71.8 mg, 0.156 mmol, 78%;
White solid;
Mp: 175 - 176 ℃;
^{1}H NMR (400 MHz, DMSO-d_6) δ 12.67 (s, 1H), 7.83 – 7.75 (m,

2H), 7.71 – 7.64 (m, 2H), 7.58 – 7.46 (m, 4H), 7.43 – 7.35 (m, 4H), 7.20 – 7.05 (m, 3H), 6.99 (d, *J* = 7.9 Hz, 1H), 6.87 (d, *J* = 8.0 Hz, 2H), 3.93 – 3.82 (m, 1H), 3.16 – 2.97 (m, 2H).
^{13}C NMR (101 MHz, DMSO-d_6) δ 173.78 (d, $J_{C\text{-}P}$ = 10.2 Hz), 157.17, 153.09 (d, $J_{C\text{-}F}$ = 246.7 Hz), 142.41 (d, $J_{C\text{-}F}$ = 11.7 Hz), 136.46 (dd, $J_{C\text{-}P}$ = 6.4 Hz, $J_{C\text{-}F}$ = 3.6 Hz), 134.63 (d, $J_{C\text{-}P}$ = 97.9 Hz), 133.68 (d, $J_{C\text{-}P}$ = 97.3 Hz), 132.04 (d, $J_{C\text{-}P}$ = 1.9 Hz), 131.73 (d, $J_{C\text{-}P}$ = 2.7 Hz), 130.86 (d, $J_{C\text{-}P}$ = 9.4 Hz), 130.70 (d, $J_{C\text{-}P}$ = 9.4 Hz), 130.40, 129.01 (d, $J_{C\text{-}P}$ = 11.4 Hz), 128.72 (d, $J_{C\text{-}P}$ = 11.4 Hz), 125.66 (d, $J_{C\text{-}F}$ = 7.2 Hz), 123.63, 122.27, 117.22 (d, $J_{C\text{-}F}$ = 18.3 Hz), 117.04, 44.49, 32.57 (d, $J_{C\text{-}P}$ = 70.7 Hz).
^{31}P NMR (162 MHz, DMSO-d_6) δ 27.58.
^{19}F NMR (376 MHz, DMSO-d_6) δ -133.05.
HRMS (ESI+): calculated m/z $[M+H]^+$ for $[C_{27}H_{22}FO_4PH]^+$: 461.1313, found: 461.1311.

3-(diphenylphosphoryl)-2-mesitylpropanoic acid (9ta)

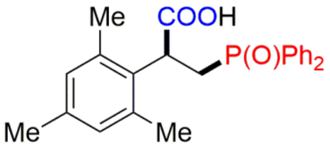

41.6 mg, 0.106 mmol, 53%;
White solid;
Mp: 151 - 153 ℃;
^{1}H NMR (400 MHz, DMSO-d_6) δ 12.51 (s, 1H), 7.87 – 7.78 (m, 2H), 7.62 – 7.48 (m, 5H), 7.46 – 7.40 (m, 1H), 7.32 (td, *J* = 7.7, 2.7 Hz, 2H), 6.60 (d, *J* = 18.0 Hz, 2H), 4.37 – 4.23 (m, 1H), 3.35 – 3.25 (m, 1H), 2.81 – 2.70 (m, 1H), 2.17 (s, 3H), 2.07 (d, *J* = 18.7 Hz, 6H).
^{13}C NMR (101 MHz, DMSO-d_6) δ 174.65 (d, *J* = 9.4 Hz), 136.88, 136.21, 135.81, 134.66 (d, *J* = 99.7 Hz), 134.20, 133.46 (d, *J* = 96.9 Hz), 132.02 (d, *J* = 1.8 Hz), 131.50 (d, *J* = 2.3 Hz), 130.77 (d, *J* = 9.8 Hz), 130.67 (d, *J* = 9.8 Hz), 130.28, 129.04 (d, *J* = 11.2 Hz), 128.61 (d, *J* = 11.2 Hz), 38.68, 30.80 (d, *J* = 71.7 Hz), 20.80, 20.69, 20.16.
^{13}C NMR (101 MHz, DMSO-d_6) δ 174.65 (d, *J* = 9.2 Hz), 136.89, 136.22, 135.81, 134.68 (d, *J* = 96.6 Hz), 134.18 (d, *J* = 4.3 Hz), 133.46 (d, *J* = 97.2 Hz), 132.02 (d, *J* = 1.2 Hz), 131.50 (d, *J* = 2.2 Hz), 130.76 (d, *J* = 9.7 Hz), 130.67 (d, *J* = 9.7 Hz), 130.28, 129.04 (d, *J* = 11.4 Hz), 128.90, 128.61 (d, *J* = 11.3 Hz), 38.68, 30.80 (d, *J* = 72.0 Hz), 20.79, 20.68, 20.16.
^{31}P NMR (162 MHz, DMSO-d_6) δ 29.12.

3-(diphenylphosphoryl)-2-methyl-2-phenylpropanoic acid (9ua)

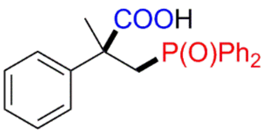

53.9 mg, 0.148 mmol, 74%;
White solid;
Mp: 175 - 177 ℃;
^{1}H NMR (400 MHz, DMSO-d_6) δ 12.58 (s, 1H), 7.85 – 7.74 (m, 4H), 7.57 – 7.35 (m, 8H), 7.27 – 7.19 (m, 2H), 7.20 – 7.11 (m, 1H), 3.29 – 3.12 (m, 2H), 1.55 (s, 3H).
^{13}C NMR (101 MHz, DMSO-d_6) δ 176.17 (d, *J* = 6.0 Hz), 143.97 (d, *J* = 10.6 Hz), 136.59 (d, *J* = 98.0 Hz), 135.53 (d, *J* = 96.0 Hz), 131.69 (d, *J* = 2.6 Hz), 131.47 (d, *J* = 2.6 Hz), 130.75 (d, *J* = 9.2 Hz), 130.38 (d, *J* = 9.2 Hz), 128.96 (d, *J* = 11.4 Hz), 128.82 (d, *J* = 11.4 Hz), 128.58, 127.18, 126.19, 48.33 (d, *J* = 3.0 Hz), 38.55 (d, *J* = 70.4 Hz), 24.27 (d, *J* = 3.1 Hz).
^{31}P NMR (162 MHz, DMSO-d_6) δ 25.52.
HRMS (ESI+): calculated m/z $[M+H]^+$ for $[C_{22}H_{21}O_3PH]^+$: 365.1301, found: 365.1296.

3-benzamido-2-((diphenylphosphoryl)methyl)-2-phenylpropanoic acid (9va)

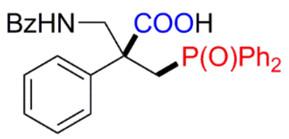

69.6 mg, 0.144 mmol, 72%;
White solid;
Mp: 166 - 168 ℃;
^{1}H NMR (400 MHz, DMSO-d_6) δ 12.94 (s, 1H), 8.53 – 8.44 (m, 1H), 7.85 – 7.78 (m, 2H), 7.75 – 7.64 (m, 4H), 7.54 – 7.40 (m, 7H), 7.38 – 7.30 (m, 4H), 7.21 – 7.07 (m, 3H), 4.18 – 4.03 (m, 2H), 3.51 (dd, *J* = 15.5, 9.3 Hz, 1H), 3.46 – 3.34 (m, 1H).
^{13}C NMR (101 MHz, DMSO-d_6) δ 174.89 (d, *J* = 7.2 Hz), 166.54, 141.02 (d, *J* = 9.2 Hz), 135.42 (d, *J* = 101.0 Hz), 134.92 (overlapped), 134.51 (d, *J* = 97.6 Hz), 131.83 (d, *J* = 1.7 Hz), 131.61 (d, *J* = 2.0 Hz), 131.54, 130.69 (d, *J* = 9.6 Hz), 130.62 (d, *J* = 9.1 Hz), 128.91 (d, *J* = 11.6 Hz), 128.78 (d, *J* = 11.4 Hz), 128.68, 128.44, 127.45, 127.41, 126.87, 52.94 (d, *J* = 2.9 Hz), 45.17, 35.52 (d, *J* = 69.3 Hz).
^{31}P NMR (162 MHz, DMSO-d_6) δ 28.02.
HRMS (ESI+): calculated m/z $[M+H]^+$ for $[C_{29}H_{26}NO_4PH]^+$: 484.1672, found: 484.1670

3-(diphenylphosphoryl)-2-phenylbutanoic acid (9wa)

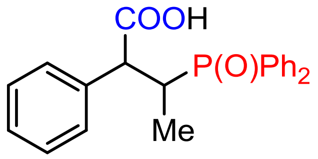

36.4 mg, 0.100 mmol, 50%;
White solid;
Mp: 133 - 134 ℃;
^{1}H NMR (400 MHz, DMSO-d_6) δ 12.54 (s, 1H), 7.96 – 7.44 (m, 8H), 7.35 – 7.10 (m, 5H), 6.97 – 6.89 (m, 2H), 3.80 (m, 1H), 3.58 – 3.46 (m, 1H), 1.11 (dd, *J* = 16.3, 7.0 Hz, 2.36H), 0.68 (dd, *J* = 16.5, 7.3 Hz, 0.59H).
^{13}C NMR (101 MHz, DMSO-d_6) δ 173.78 (d, *J* = 4.8 Hz), 173.62 (d, *J* = 14.2 Hz), 137.50 (d, *J* = 9.8 Hz), 136.52 (d, *J* = 4.4 Hz), 134.36 (d, *J* = 94.9 Hz), 134.00 (d, *J* = 94.3 Hz), 133.15 (d, *J* = 94.7 Hz), 133.09 (d, *J* = 93.6 Hz), 132.08 (d, *J* = 2.9 Hz), 131.95 (d, *J* = 2.5 Hz), 131.63 (d, *J* = 2.7 Hz), 131.60 (d, *J* = 8.8 Hz), 131.28 (d, *J* = 8.5 Hz), 130.79 (d, *J* = 8.6 Hz), 130.57 (d, *J* = 9.3 Hz), 129.50, 129.44, 129.36, 129.04 (d, *J* = 11.0 Hz), 128.99 (d, *J* = 11.2 Hz), 128.78 (d, *J* = 11.2 Hz), 128.76, 128.14 (d, *J* = 10.8 Hz), 128.08, 127.72, 127.55, 52.57, 50.58, 34.85 (d, *J* = 71.5 Hz), 33.21 (d, *J* = 69.5 Hz), 13.32 (d, *J* = 1.9 Hz), 12.51 (d, *J* = 2.0 Hz).
^{31}P NMR (162 MHz, DMSO-d_6) δ 34.62, 32.64.
HRMS (ESI+): calculated m/z $[M+Na]^+$ for $[C_{22}H_{21}O_3PNa]^+$: 387.1121, found: 365.1116.

3-(diphenylphosphoryl)-2-(naphthalen-2-yl)propanoic acid (9xa)

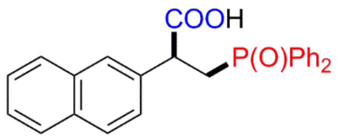

37.6 mg, 0.094 mmol, 47%;
White solid;
Mp: 202 - 204 ℃;
^{1}H NMR (400 MHz, DMSO-d_6) δ 12.59 (s, 1H), 7.87 – 7.75 (m, 5H), 7.74 – 7.66 (m, 3H), 7.60 – 7.43 (m, 5H), 7.40 – 7.29 (m, 4H), 4.00 (ddd, *J* = 11.8, 7.9, 5.7 Hz, 1H), 3.31 (ddd, *J* = 15.2, 12.4, 8.0 Hz, 1H), 3.00 (ddd, *J* = 15.0, 9.0, 5.8 Hz, 1H).
^{13}C NMR (101 MHz, DMSO-d_6) δ 173.91 (d, *J* = 7.3 Hz), 137.06 (d, *J* = 8.8 Hz), 134.52 (d, *J* = 97.6 Hz), 133.74 (d, *J* = 97.2 Hz), 133.20, 132.53, 132.07 (d, *J* = 2.6 Hz), 131.65 (d, *J* = 2.7 Hz), 130.89 (d, *J* = 9.3 Hz), 129.03 (d, *J* = 11.4 Hz), 128.72 (d, *J* = 11.5 Hz), 128.43, 128.06, 127.80, 127.09, 126.56, 126.33, 126.28, 45.30 (d, *J* = 2.1 Hz), 32.66 (d, *J* = 70.2 Hz).
^{31}P NMR (162 MHz, DMSO-d_6) δ 27.89.
HRMS (ESI+): calculated m/z $[M+H]^+$ for $[C_{25}H_{21}O_3PH]^+$: 401.1301, found: 401.1298.

2-(benzo[b]thiophen-5-yl)-3-(diphenylphosphoryl)propanoic acid (9ya)

61.0 mg, 0.150 mmol, 75%;

White solid;

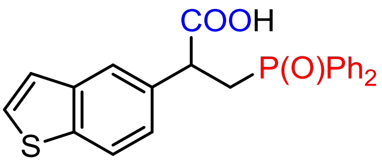

Mp: 110 - 112 ℃;

^{1}H NMR (400 MHz, DMSO-d_6) δ 12.52 (s, 1H), 7.83 (t, J = 8.0 Hz, 3H), 7.75 – 7.63 (m, 4H), 7.61 – 7.46 (m, 3H), 7.43 – 7.29 (m, 4H), 7.21 (d, J = 8.3 Hz, 1H), 4.03 – 3.90 (m, 1H), 3.27 (ddd, J = 15.0, 12.6, 7.9 Hz, 1H), 2.96 (ddd, J = 15.0, 8.7, 6.1 Hz, 1H).

^{13}C NMR (101 MHz, DMSO-d_6) δ 174.10 (d, J = 7.7 Hz), 139.96, 138.42, 135.80 (d, J = 8.8 Hz), 134.53 (d, J = 97.6 Hz), 133.69 (d, J = 97.2 Hz), 132.06 (d, J = 2.5 Hz), 131.59 (d, J = 2.3 Hz), 130.81 (d, J = 9.3 Hz), 129.02 (d, J = 11.5 Hz), 128.66 (d, J = 11.5 Hz), 128.28, 124.62, 124.29, 123.31, 122.93, 45.05, 33.01 (d, J = 70.2 Hz).

^{31}P NMR (162 MHz, DMSO-d_6) δ 27.92.

HRMS (ESI+): calculated m/z $[M+H]^+$ for $[C_{23}H_{19}O_3PSH]^+$: 407.0865, found: 407.0864.

2-(1-(*tert*-butoxycarbonyl)-1*H*-indol-5-yl)-3-(diphenylphosphoryl)propanoic acid (9za)

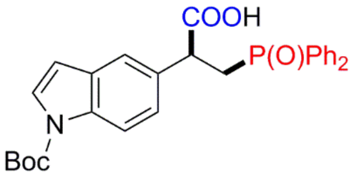

52.8 mg, 0.108 mmol, 54%;

White solid;

Mp: 135 - 136 ℃;

^{1}H NMR (400 MHz, DMSO-d_6) δ 12.44 (s, 1H), 7.90 – 7.78 (m, 3H), 7.74 – 7.64 (m, 2H), 7.64 (d, J = 3.7 Hz, 1H), 7.59 – 7.48 (m, 3H), 7.47 – 7.32 (m, 4H), 7.17 (dd, J = 8.7, 1.8 Hz, 1H), 6.63 (d, J = 3.7 Hz, 1H), 3.91 (ddd, J = 11.7, 8.0, 5.5 Hz, 1H), 3.27 (ddd, J = 15.2, 12.2, 8.2 Hz, 1H), 2.99 – 2.85 (m, 1H), 1.62 (s, 9H).

^{13}C NMR (101 MHz, DMSO-d_6) δ 174.18 (d, J = 7.0 Hz), 149.43, 134.54 (d, J = 97.1 Hz), 134.33, 134.08 (d, J = 1.4 Hz), 133.84 (d, J = 97.0 Hz), 132.02 (d, J = 2.2 Hz), 131.62 (d, J = 2.3 Hz), 130.85 (d, J = 9.4 Hz), 130.83 (d, J = 9.4 Hz), 130.69, 128.99 (d, J = 11.4 Hz), 128.69 (d, J = 11.4 Hz), 126.85, 124.49, 120.74, 115.01, 107.87, 84.20, 44.94 (d, J = 1.7 Hz), 33.15 (d, J = 70.2 Hz), 28.09.

^{31}P NMR (162 MHz, DMSO-d_6) δ 27.83.

HRMS (ESI+): calculated m/z $[M+H]^+$ for $[C_{28}H_{28}NO_5PH]^+$: 490.1778, found: 490.1780.

3-(diphenylphosphoryl)-2-(thiophen-2-yl)propanoic acid (9aaa)

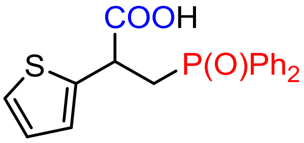

35.8 mg, 0.098 mmol, 49%;

White solid;

Mp: 103- 104 ℃;

^{1}H NMR (400 MHz, DMSO-d_6) δ 12.62 (s, 1H), 7.83 – 7.68 (m, 4H), 7.57 – 7.36 (m, 6H), 7.30 (dd, J = 5.1, 0.9 Hz, 1H), 6.89 (d, J = 3.4 Hz, 1H), 6.81 (dd, J = 5.1, 3.5 Hz, 1H), 4.05 (m, 1H), 3.14 (m, 1H), 2.98 – 2.87 (m, 1H).

^{13}C NMR (101 MHz, DMSO-d_6) δ 173.07 (d, J = 6.3 Hz), 141.73 (d, J = 11.2 Hz), 134.29 (d, J = 98.0 Hz), 133.59 (d, J = 97.5 Hz), 132.12 (d, J = 2.6 Hz), 131.93 (d, J = 2.5 Hz), 130.89 (d, J = 8.5 Hz), 130.81 (d, J = 9.3 Hz), 129.03 (d, J = 11.4 Hz), 128.92 (d, J = 10.7 Hz), 127.07, 126.28, 125.64, 40.69 (d, J = 1.8 Hz), 33.70 (d, J = 69.6 Hz).

^{31}P NMR (162 MHz, DMSO-d_6) δ 27.35.
HRMS (ESI+): calculated m/z $[M+Na]^+$ for $[C_{19}H_{17}O_3PSNa]^+$: 379.0528, found: 357.0525.

3-(diphenylphosphoryl)-2-(furan-3-yl)-2-phenylpropanoic acid (9aba)

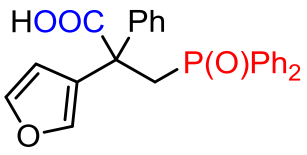

53.3 mg, 0.128 mmol, 64%;
White solid;
Mp: 184 - 185 ℃;
^{1}H NMR (400 MHz, DMSO-d_6) δ 12.79 (s, 1H), 7.67 (m, 2H), 7.63 – 7.53 (m, 3H), 7.48 – 7.34 (m, 6H), 7.30 m, 2H), 7.17 (dd, J = 8.2, 6.7 Hz, 2H), 7.14 – 7.07 (m, 1H), 7.03 (t, J = 1.7 Hz, 1H), 5.82 (dd, J = 1.8, 0.7 Hz, 1H), 3.61 – 3.48 (m, 2H).
^{13}C NMR (101 MHz, DMSO-d_6) δ 173.82 (d, J = 3.1 Hz), 142.86 (d, J = 10.8 Hz), 142.06, 141.92, 136.48 (d, J = 98.2 Hz), 134.92 (d, J = 98.5 Hz), 131.43 (d, J = 2.5 Hz), 131.02 (d, J = 2.1 Hz), 130.59 (d, J = 9.3 Hz), 130.35 (d, J = 9.1 Hz), 128.77 (d, J = 11.3 Hz), 128.35 (d, J = 11.6 Hz), 128.23, 127.77, 127.34, 126.59 (d, J = 5.6 Hz), 111.48, 50.99 (d, J = 3.3 Hz), 38.06 (d, J = 73.1 Hz).
^{31}P NMR (162 MHz, DMSO-d_6) δ 25.15.
HRMS (ESI+): calculated m/z $[M+H]^+$ for $[C_{25}H_{21}O_4PH]^+$: 417.1250, found: 417.1252.

3-(diphenylphosphoryl)-2-hydroxy-2-(4-(methoxycarbonyl)phenyl)propanoic acid (9aca)

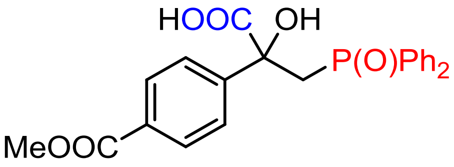

50.9 mg, 0.120 mmol, 60%;
White solid;
Mp: 190 – 191 (decomposed) ℃;
^{1}H NMR (400 MHz, DMSO-d_6) δ 13.07 (s, 1H), 7.84 (d, J = 8.5 Hz, 2H), 7.74 (m, 4H), 7.67 (d, J = 8.4 Hz, 2H), 7.55 – 7.35 (m, 6H), 6.05 (s, 1H), 3.81 (s, 1H), 3.43 (dd, J = 14.9, 12.6 Hz, 1H), 3.24 (dd, J = 15.1, 7.8 Hz, 1H).
^{13}C NMR (101 MHz, DMSO-d_6) δ 166.40, 148.97 (d, J = 9.9 Hz), 135.60 (d, J = 98.2 Hz), 135.25 (d, J = 100.4 Hz), 131.84 (d, J = 2.3 Hz), 131.45 (d, J = 2.2 Hz), 130.78 (d, J = 9.6 Hz), 130.57 (d, J = 9.4 Hz), 129.20, 129.09, 128.93 (d, J = 11.5 Hz), 128.62 (d, J = 11.9 Hz), 126.27 (s), 76.63 (d, J = 5.6 Hz), 52.57, 39.10 (d, J = 76.8 Hz).
^{31}P NMR (162 MHz, DMSO-d_6) δ 27.30.
HRMS (ESI+): calculated m/z $[M+H]^+$ for $[C_{23}H_{21}O_6PH]^+$: 425.1149, found: 425.1150.

3-(diphenylphosphoryl)-2-hydroxy-2-(thiophen-3-yl)propanoic acid (9ada)

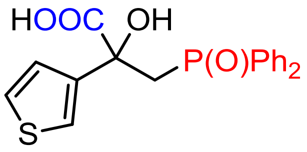

32.02 mg, 0.086 mmol, 43%;
White solid;
Mp: 210 – 211 (decomposed) ℃;
^{1}H NMR (400 MHz, DMSO-d_6) δ 13.10 (s, 1H), 7.80 – 7.69 (m, 7H), 7.59 – 7.46 (m, 4H), 7.41 (m, 2H), 6.18 (s, 1H), 3.44 (dd, J = 15.1, 12.4 Hz, 1H), 3.29 (dd, J = 15.2, 8.1 Hz, 1H).
^{13}C NMR (101 MHz, DMSO-d_6) δ 173.90 (d, J = 5.4 Hz), 148.96 (d, J = 9.3 Hz), 135.51 (d, J = 98.5 Hz), 135.11 (d, J = 100.2 Hz), 132.23, 131.86 (d, J = 2.5 Hz), 131.46 (d, J = 2.5 Hz), 130.77

(d, J = 9.6 Hz), 130.56 (d, J = 9.4 Hz), 128.93 (d, J = 11.5 Hz), 128.62 (d, J = 11.9 Hz), 127.01, 119.17, 76.60 (d, J = 5.6 Hz), 38.79 (d, J = 69.7 Hz).

^{31}P NMR (162 MHz, DMSO-d_6) δ 27.43.

HRMS (ESI+): calculated m/z $[M+H]^+$ for $[C_{19}H_{17}O_4PSH]^+$: 373.0658, found: 373.0660.

2-((diphenylphosphoryl)methyl)-3-methoxy-2-methyl-3-oxopropanoic acid (11aa)

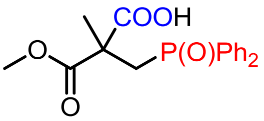

58.9 mg, 0.170 mmol, 85%;

White solid;

Mp: 114 - 116 ℃;

^{1}H NMR (400 MHz, DMSO-d_6) δ 13.15 (s, 1H), 7.89 – 7.77 (m, 4H), 7.60 – 7.45 (m, 6H), 3.44 (s, 3H), 3.16 – 2.93 (m, 2H), 1.35 (s, 3H).

^{13}C NMR (101 MHz, DMSO-d_6) δ 172.79 (d, J = 12.6 Hz), 171.34 (d, J = 6.3 Hz), 135.32 (d, J = 98.0 Hz), 134.91 (d, J = 98.1 Hz), 132.02 (d, J = 2.6 Hz), 131.97 (d, J = 2.6 Hz), 130.71 (d, J = 9.4 Hz), 130.65 (d, J = 9.3 Hz), 52.61, 51.29 (d, J = 2.5 Hz), 34.95 (d, J = 71.1 Hz), 21.09 (d, J = 2.7 Hz).

^{31}P NMR (162 MHz, DMSO-d_6) δ 25.18.

HRMS (ESI+): calculated m/z $[M+H]^+$ for $[C_{18}H_{19}O_5PH]^+$: 347.1043, found: 347.1042

3-(*tert*-butoxy)-2-((diphenylphosphoryl)methyl)-2-methyl-3-oxopropanoic acid (11ba)

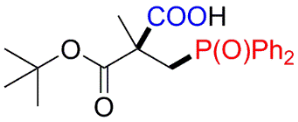

59.8 mg, 0.154 mmol, 77%;

White solid;

Mp: 121 - 124 ℃;

^{1}H NMR (400 MHz, $CDCl_3$) δ 8.21 (s, 1H), 7.87 – 7.74 (m, 4H), 7.59 – 7.39 (m, 6H), 3.28 (dd, J = 15.5, 12.8 Hz, 1H), 2.93 (dd, J = 15.5, 7.0 Hz, 1H), 1.39 (s, 9H), 1.35 (s, 3H).

^{13}C NMR (101 MHz, $CDCl_3$) δ 172.07, 171.47 (d, J = 12.8 Hz), 133.33 (d, J = 102.6 Hz), 132.64 (d, J = 100.9 Hz), 131.93 (d, J = 2.8 Hz), 131.83 (d, J = 2.9 Hz), 130.95 (d, J = 9.6 Hz), 130.69 (d, J = 9.9 Hz), 128.71 (d, J = 11.6 Hz), 128.59 (d, J = 11.8 Hz), 82.41, 52.35 (d, J = 1.6 Hz), 35.09 (d, J = 72.1 Hz), 27.63, 21.74.

^{31}P NMR (162 MHz, $CDCl_3$) δ 31.02.

HRMS (ESI+): calculated m/z $[M+H]^+$ for $[C_{21}H_{25}O_5PH]^+$: 389.1512, found: 389.1509.

2-(tert-butoxycarbonyl)-2-((diphenylphosphoryl)methyl)butanoic acid (11ca)

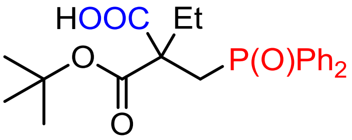

57.1 mg, 0.142 mmol, 71%;

White solid;

Mp: 123 - 124 ℃;

^{1}H NMR (400 MHz, DMSO-d_6) δ 12.84 (s, 1H), 7.82 (m, 4H), 7.51 (m, 6H), 3.01 – 2.85 (m, 2H), 1.91 – 1.76 (m, 2H), 1.27 (s, 9H), 0.46 (t, J = 7.4 Hz, 3H).

^{13}C NMR (101 MHz, DMSO-d_6) δ 171.93 (d, J = 10.3 Hz), 169.44 (d, J = 9.2 Hz), 135.54 (d, J = 97.6 Hz), 135.39 (d, J = 97.2 Hz), 131.95 (d, J = 2.3 Hz), 130.66 (d, J = 9.3 Hz), 130.64 (d, J = 9.2 Hz), 129.05 (d, J = 11.4 Hz), 129.01 (d, J = 11.4 Hz), 81.31, 56.64 (d, J = 1.4 Hz), 31.61 (d, J = 72.1 Hz), 27.75, 25.52 , 8.91.

^{31}P NMR (162 MHz, DMSO-d_6) δ 24.37.

HRMS (ESI+): calculated m/z $[M+H]^+$ for $[C_{22}H_{27}O_5PH]^+$: 403.1669, found: 403.1662.

2-(*tert*-butoxycarbonyl)-2-((diphenylphosphoryl)methyl)hexanoic acid (11da)

67.1 mg, 0.156 mmol, 78%;

White solid;

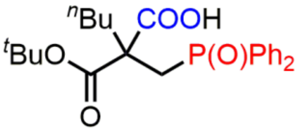

Mp: 95 - 97 ℃;

^{1}H NMR (400 MHz, DMSO-d_6) δ 12.88 (s, 1H), 7.99 – 7.76 (m, 4H), 7.65 – 7.38 (m, 6H), 2.98 (d, J = 10.0 Hz, 2H), 1.97 – 1.57 (m, 2H), 1.33 (s, 9H), 0.88 – 0.63 (m, 4H), 0.54 (t, J = 6.8 Hz, 3H).

^{13}C NMR (101 MHz, DMSO-d_6) δ 171.97 (d, J = 10.3 Hz), 169.44 (d, J = 9.4 Hz), 135.56 (d, J = 97.5 Hz), 135.38 (d, J = 97.1 Hz), 131.90 (d, J = 2.5 Hz), 130.71 (d, J = 9.2 Hz), 130.63 (d, J = 9.0 Hz), 129.03 (d, J = 11.4 Hz), 129.00 (d, J = 11.4 Hz), 81.24, 56.07, 32.30 (d, J = 33.2 Hz), 31.93 (d, J = 39.4 Hz), 27.80, 26.41, 22.51, 13.97.

^{31}P NMR (162 MHz, DMSO-d_6) δ 24.34.

HRMS (ESI+): calculated m/z $[M+H]^+$ for $[C_{24}H_{31}O_5PH]^+$: 431.1982, found: 431.1981.

2-((diphenylphosphoryl)methyl)-2-methyl-3-oxo-3-(tert-pentyloxy)propanoic acid (11ea)

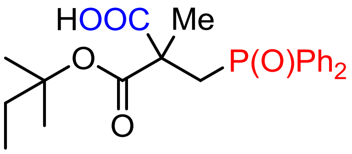

56.3 mg, 0.140 mmol, 70%;

White solid;

Mp: 119 - 120 ℃;

^{1}H NMR (400 MHz, DMSO-d_6) δ 12.82 (s, 1H), 7.80 (m, 4H), 7.51 (m, 6H), 2.98 (d, J = 10.1 Hz, 2H), 1.67 – 1.49 (m, 2H), 1.28 (d, J = 9.9 Hz, 6H), 1.20 (s, 3H), 0.74 (t, J = 7.4 Hz, 3H).

^{13}C NMR (101 MHz, DMSO-d_6) δ 172.78 (d, J = 10.4 Hz), 169.97 (d, J = 8.5 Hz), 135.68 (d, J = 97.8 Hz), 135.56 (d, J = 97.3 Hz), 131.92 (d, J = 2.4 Hz), 130.63 (d, J = 9.1 Hz), 130.55 (d, J = 9.1 Hz), 129.07 (d, J = 11.4 Hz), 52.18 (d, J = 2.4 Hz), 34.96 (d, J = 71.7 Hz), 25.16 (d, J = 7.3 Hz), 20.88 (d, J = 2.9 Hz), 8.25

^{31}P NMR (162 MHz, DMSO-d_6) δ 24.97.

HRMS (ESI+): calculated m/z $[M+H]^+$ for $[C_{22}H_{27}O_5PH]^+$: 403.1669, found: 403.1667.

tert-butyl 3-((diphenylphosphoryl)methyl)-2-oxotetrahydro-2H-pyran-3-carboxylate (11fa)

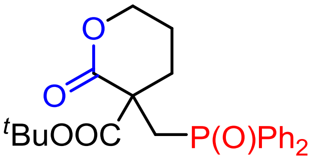

67.1 mg, 0.162 mmol, 81%;

Semi-solid;

^{1}H NMR (400 MHz, DMSO-d_6) δ 7.86 (m, 2H), 7.75 – 7.67 (m, 2H), 7.56 – 7.43 (m, 6H), 4.42 – 4.24 (m, 2H), 3.15 (dd, J = 15.4, 10.8 Hz, 1H), 2.97 (dd, J = 15.4, 8.1 Hz, 1H), 2.18 – 2.07 (m, 1H), 1.93 (dd, J = 13.4, 3.8 Hz, 1H), 1.83 (m, 1H), 1.77 – 1.65 (m, 1H), 1.35 (s, 9H).

^{13}C NMR (101 MHz, DMSO-d_6) δ 170.05 (d, J = 14.1 Hz), 169.27 (d, J = 4.1 Hz), 135.49 (d, J = 99.7 Hz), 135.46 (d, J = 97.4 Hz), 132.13 (d, J = 2.6 Hz), 132.00 (d, J = 2.5 Hz), 130.67 (d, J = 9.6 Hz), 130.54 (d, J = 9.6 Hz), 129.29 (d, J = 11.6 Hz), 129.02 (d, J = 11.7 Hz), 83.00, 69.99, 52.70 (d, J = 2.8 Hz), 35.83 (d, J = 71.1 Hz), 29.93 (d, J = 2.8 Hz), 27.69, 20.85.

^{31}P NMR (162 MHz, DMSO-d_6) δ 25.81.

HRMS (ESI+): calculated m/z $[M+H]^+$ for $[C_{23}H_{27}O_5PH]^+$: 415.1669, found: 415.1669.

The application of the reaction

a Gram-scale reaction

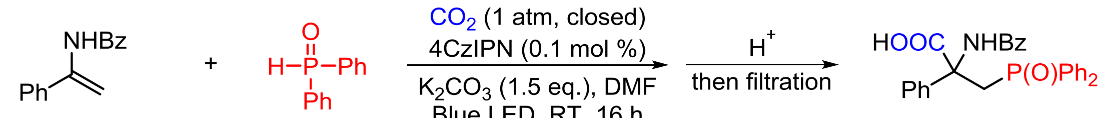

1a, 3.0 mmol **2a**, 3.6 mmol **3aa**, 1.04 g, 74%

An oven-dried Schlenk tube (100 mL) containing a stirring bar was charged with the enamide **1a** (3.0 mmol, 670 mg) and 4CzIPN (2.4 mg, 0.1 mol %). The Schlenk tube was then introduced in a glovebox, where it was charged with H-P(O)Ph_2 **2a** (728 mg, 3.6 mmol, 1.2 equiv.) and K_2CO_3 (622 mg, 4.5 mmol, 1.5 eq.). The tube was taken out of the glovebox and connected to a vacuum line where it was evacuated and back-filled with CO_2 for 3 times. Then DMF (15 mL) were added under CO_2 flow. Finally, the reaction mixture in sealed tube was placed at the center of four 30 W blue LEDs and stirred at room temperature (25 ℃). After 16 h, the mixture was quenched with 67.5 mL H_2O, then the mixture was transferred to a 200 mL beaker and 30 mL ethyl acetate and 7.5 mL of HCl (2 N) were added in sequence. After that, white solid was precipitated from the solution. The two phase solution was stirring for about 5 minutes at ice bath conditions. After filtration and drying, the desired white solid product 1.04 g was obtained.

b Product transformations

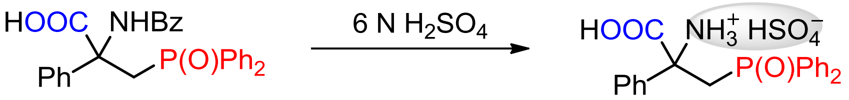

3aa **12**, 85%

A 10 mL flask was charged with **3aa** (50 mg, 0.106 mmol, 1.0 eq.) and 6 N H_2SO_4 (aq.) solution 5 mL. The reaction mixture was heated at 120 ℃ for 24 h. After cooling to room temperature and standing for 12 h, the transparent acicular crystals were precipitated from the reaction mixture, the crude product was obtained after filtration and washed with EA to remove the benzoic acid to get the pure sulfate product (42 mg, yield: 85%).

2-amino-3-(diphenylphosphoryl)-2-phenylpropanoic acid sulfate (12)

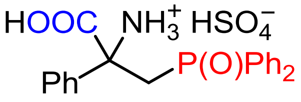

42.0 mg, 0.170 mmol, 85%;

White solid;

Mp: 177 - 179 ℃;

^{1}H NMR (400 MHz, DMSO-d_6) δ 9.21 (s, 3H), 7.92 – 7.86 (m, 2H), 7.74 – 7.68 (m, 2H), 7.66 – 7.61 (m, 1H), 7.60 – 7.50 (m, 5H), 7.48 – 7.42 (m, 2H), 7.35 – 7.28 (m, 3H), 3.73 (dd, J = 15.9, 10.0 Hz, 1H), 3.52 (dd, J = 15.8, 10.2 Hz, 1H).

^{13}C NMR (101 MHz, DMSO-d_6) δ 170.13 (d, J = 7.1 Hz), 135.44 (d, J = 8.9 Hz), 132.96 (d, J = 101.6 Hz), 132.87(d, J = 1.9 Hz), 132.53 (d, J = 2.1 Hz), 132.31 (d, J = 100.9 Hz), 130.91 (d, J = 9.9 Hz), 130.54 (d, J = 10.1 Hz), 129.62, 129.22 (d, J = 12.3 Hz), 129.10 (d, J = 12.1 Hz), 129.01, 126.14, 63.39 (d, J = 4.4 Hz), 33.87 (d, J = 65.3 Hz).

^{31}P NMR (162 MHz, DMSO-d_6) δ 31.72.

HRMS (ESI+): calculated m/z $[M\text{-}H_2SO_4+H]^+$ for $[C_{21}H_{20}NO_3PH]^+$: 366.1254, found: 366.1252.

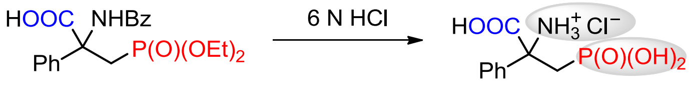

3ah

13, quantitative
(anologues of AP3)

A 10 mL flask was charged with **3ah** (35 mg, 0.086 mmol, 1.0 equiv.) and 6 N HCl (aq.) solution 6 mL. The reaction mixture was heated at 100 ℃ for 12 h. After removing H_2O under heat conditions, the crude product was washed with EA to give 2-amino-2-phenylpropanoic acid hydrochloride **13** (24.2 mg, quantitative yield) as white solid (hygroscopicity).

2-amino-2-phenyl-3-phosphonopropanoic acid hydrochloride (13)

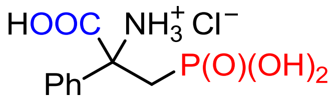

24.2 mg, 0.200 mmol, 100%;
White solid;
^{1}H NMR (400 MHz, DMSO-d_6) δ 9.17 (s, 3H), 7.60 – 7.52 (m, 2H), 7.50 – 7.34 (m, 3H), 2.82 (dd, J = 18.5, 15.4 Hz, 1H), 2.59 (t, J = 15.9 Hz, 1H).
^{13}C NMR (101 MHz, DMSO-d_6) δ 170.53 (d, J = 6.7 Hz), 136.53 (d, J = 13.4 Hz), 129.53, 129.20, 126.27, 61.96 (d, J = 3.4 Hz), 32.50 (d, J = 130.1 Hz).
^{31}P NMR (162 MHz, DMSO-d_6) δ 19.76.
HRMS (ESI-): calculated m/z $[M\text{-}HCl\text{-}H]^-$ for $[C_9H_{11}NO_5P]^-$: 244.0380, found: 244.0381.

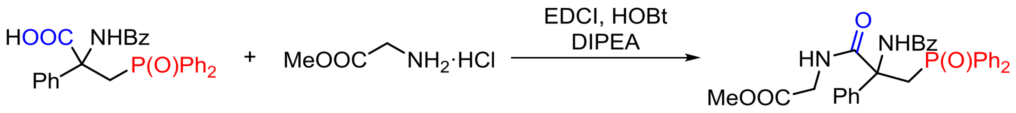

3aa

14, 92%

A 10 mL flask was charged with **3aa** (50 mg, 0.106 mmol, 1.0 equiv.), glycine methyl ester hydrochloride (0.212 mmol, 2.0 equiv.), EDCI (46 mg, 0.24 mmol) and HOBt (32.4 mg, 0.24 mmol). 4 mL DMF was added and the mixture was stirred at 70 ℃ for 8 h. After quenching with water, then reaction mixture was extracted with EA for 3 times. The combined organic phase was concentrated in vacuum and the residue was purified by silica gel flash chromatography (petroleum ether/AcOEt 2/1 ~ 1/1) to give the pure desired product **14** (52.7 mg, 92%) as white solid.

methyl (2-benzamido-3-(diphenylphosphoryl)-2-phenylpropanoyl)glycinate (14)

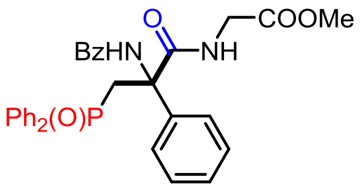

52.7 mg, 0.901 mmol, 92%;
White solid;
Mp: 201 - 202 ℃;
^{1}H NMR (400 MHz, DMSO-d_6) δ 9.06 (s, 1H), 8.35 (t, J = 5.6 Hz, 1H), 7.84 – 7.76 (m, 2H), 7.73 – 7.65 (m, 2H), 7.62 – 7.40 (m, 10H), 7.29 – 7.19 (m, 3H), 7.14 (t, J = 7.3 Hz, 2H), 7.11 – 7.06 (m, 1H), 3.94 (dd, J = 15.2, 8.8 Hz, 1H), 3.76 – 3.65 (m, 2H), 3.61 – 3.54 (m, 1H), 3.52 (s, 3H).
^{13}C NMR (101 MHz, DMSO-d_6) δ 171.37 (d, J = 6.9 Hz), 170.22, 165.25, 139.96 (d, J = 7.8 Hz), 135.05 (d, J = 98.8 Hz), 134.48, 133.41 (d, J = 99.1 Hz), 131.98 (d, J = 2.2 Hz), 131.89, 131.33 (d, J = 2.2 Hz), 130.72 (d, J = 9.3 Hz), 130.63 (d, J = 9.4 Hz), 128.92 (d, J = 11.5 Hz), 128.73, 128.45 (d, J = 11.7 Hz), 127.92, 127.47, 127.36, 126.93, 63.24 (d, J = 4.9 Hz), 51.98, 41.82, 34.67 (d, J = 70.1 Hz).
^{31}P NMR (162 MHz, DMSO-d_6) δ 27.44.
HRMS (ESI+): calculated m/z $[M+Na]^+$ for $[C_{31}H_{29}N_2O_5PNa]^+$: 563.1706, found: 563.1702.

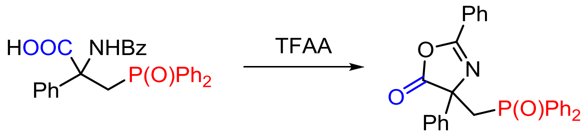

3aa **15**, 80%

A dry reaction tube was charged with **3aa** (117 mg, 0.25 mmol) under an N_2 atmosphere. Then, trifluoroacetic anhydride (76 μL, 0.55 mmol, 2.2 eq.) and DCM (5 mL) were added and the mixture was stirred at room temperature for 2 hours. The product were purified by silica gel column chromatographic (petroleum ether/AcOEt 3/1 to 2/1) to give 4-((diphenylphosphoryl) methyl)-2,4-diphenyloxazol-5(4H)-one (**15**) (90.3 mg, 80% yield) as white solid.

4-((diphenylphosphoryl)methyl)-2,4-diphenyloxazol-5(4*H*)-one (15)

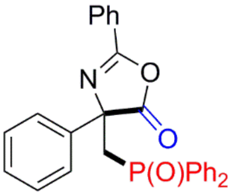

90.3 mg, 0.160 mmol, 80%;

White solid;

Mp: 188 - 191 ℃;

^{1}H NMR (400 MHz, DMSO-d_6) δ 7.82 – 7.75 (m, 2H), 7.74 – 7.64 (m, 3H), 7.60 – 7.46 (m, 10H), 7.45 – 7.35 (m, 5H), 3.90 (dd, *J* = 15.3, 5.5 Hz, 1H), 3.53 – 3.41 (m, 1H).

^{13}C NMR (101 MHz, DMSO-d_6) δ 178.15 (d, *J* = 1.5 Hz), 161.31, 139.04 (d, *J* = 11.6 Hz), 135.11 (d, *J* = 98.8 Hz), 134.39 (d, *J* = 100.3 Hz), 133.67, 132.21 (d, *J* = 2.7 Hz), 131.59 (d, *J* = 2.9 Hz), 130.94 (d, *J* = 9.7 Hz), 130.42 (d, *J* = 9.5 Hz), 129.37, 129.22, 129.17 (d, *J* = 11.9 Hz), 129.06, 128.67 (d, *J* = 12.0 Hz), 128.26, 125.93, 125.53, 70.90 (d, *J* = 5.5 Hz), 39.09 (d, *J* = 69.4 Hz).

^{31}P NMR (162 MHz, DMSO-d_6) δ 26.92.

HRMS (ESI+): calculated m/z $[M+H]^+$ for $[C_{28}H_{22}NO_3PH]^+$: 452.1410, found: 452.1406.

c Reaction with a chiral H-P(O) compound

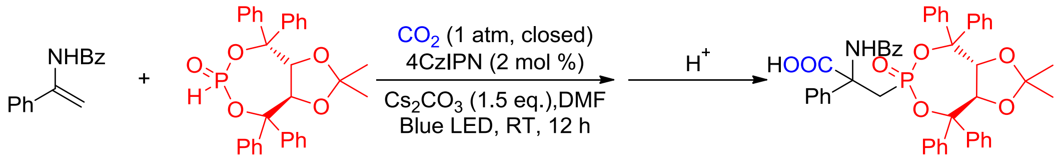

1a, 0.2 mmol **2m**, 0.24 mmol **3am**, 75%, d.r. = 1:1.2

diastereoisomers could be separated

An oven-dried Schlenk tube (10 mL) containing a stirring bar was charged with enamide **1a** (45 mg, 0.2 mmol), H-P(O) compound **2m** (123 mg, 0.24 mmol) and 4CzIPN (3.2 mg, 2 mol%). The Schlenk tube was then introduced in a glovebox, where it was charged with Cs_2CO_3 (98 mg, 0.3 mmol, 1.5 eq.). The tube was taken out of the glovebox and connected to a vacuum line where it was evacuated and back-filled with CO_2 for 3 times. Then DMF (2 mL) was added under CO_2 flow. Finally, the reaction mixture in sealed tube was placed at a distance of 2 ~ 3 cm from a 30 W blue LED and stirred at room temperature (25 ℃) for 12 h. Then, the mixture was quenched with 4.5 mL of H_2O and 0.5 mL of 2N HCl (aq.), extracted with ethyl acetate (EA) for at least 4 times, then the organic phase concentrated in vacuo. The residue was purified by silica gel flash

chromatography (0.67% AcOH in Petrolum ether/Ethyl acetate 2/1 ~ 1/1) to give the two diastereoisomers.

2-benzamido-3-((3aR,8aR)-2,2-dimethyl-6-oxido-4,4,8,8-tetraphenyltetrahydro-[1,3]dioxolo[4,5-e][1,3,2]dioxaphosphepin-6-yl)-2-phenylpropanoic acid (3am)

R_f = 0.3 (PE / EA = 1:1 with 2% acetic acids)

53.1 mg, 0.068 mmol, 34%;

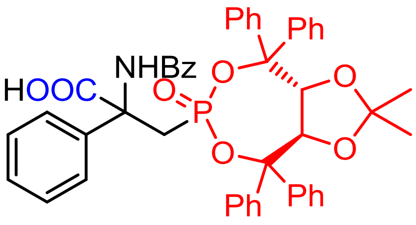

Minor

White solid;

Mp: 254 - 255 ℃;

^{1}H NMR (400 MHz, DMSO-d_6) δ 13.80 (s, 1H), 8.06 (s, 1H), 7.58 – 7.47 (m, 7H), 7.41 – 7.26 (m, 20H), 7.22 – 7.14 (m, 3H), 5.50 (d, *J* = 8.1 Hz, 1H), 4.89 (d, *J* = 8.1 Hz, 1H), 3.86 – 3.71 (m, 1H), 3.54 (dd, *J* = 19.6, 15.4 Hz, 1H), 1.08 (s, 3H), 0.30 (s, 3H).

^{13}C NMR (101 MHz, DMSO-d_6) δ 172.56 (d, *J* = 4.3 Hz), 165.54, 144.72 (d, *J* = 8.7 Hz), 144.58, 139.84 (d, *J* = 0.8 Hz), 139.68 (d, *J* = 9.6 Hz), 134.53, 131.85, 131.36, 130.03 (d, *J* = 10.0 Hz), 129.03, 128.78, 128.76, 128.70, 128.54, 128.50, 128.34, 128.16, 128.09, 127.98, 127.86, 127.60, 127.41, 126.94, 126.84, 126.08, 87.40 (d, *J* = 10.9 Hz), 86.28 (d, *J* = 7.9 Hz), 81.67 (d, *J* = 1.0 Hz), 79.44 (d, *J* = 2.7 Hz), 61.75 (d, *J* = 3.4 Hz), 32.92 (d, *J* = 148.7 Hz), 27.38, 25.82.

^{31}P NMR (162 MHz, DMSO-d_6) δ 21.80.

HRMS (ESI+): calculated m/z $[M+Na]^+$ for $[C_{47}H_{42}NO_8PNa]^+$: 802.2540, found: 802.2532.

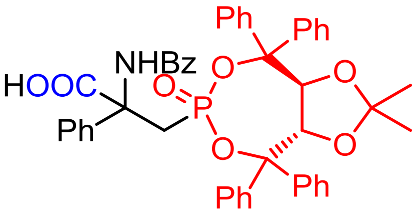

Major

R_f = 0.2 (PE / EA = 1:1 with 2% acetic acids)

63.4 mg, 0.082 mmol, 41%;

White solid;

Mp: 119 - 120 ℃;

^{1}H NMR (400 MHz, DMSO-d_6) δ 13.59 (s, 1H), 8.15 (s, 1H), 7.67 – 7.61 (m, 2H), 7.51 – 7.32 (m, 12H), 7.31 – 7.18 (m, 9H), 7.14 (m, 5.9, 2.8 Hz, 1H), 7.08 (m, 2H), 7.02 (m, 4H), 5.34 (d, *J* = 8.0 Hz, 1H), 4.98 (d, *J* = 8.0 Hz, 1H), 3.54 – 3.44 (m, 1H), 3.23 (dd, *J* = 19.8, 15.5 Hz, 1H), 0.78 (s, 3H), 0.30 (s, 3H).

^{13}C NMR (101 MHz, DMSO-d_6) δ 172.29 (d, *J* = 6.9 Hz), 165.23, 144.09 (d, *J* = 5.6 Hz), 143.83, 140.03 (d, *J* = 3.5 Hz), 139.42 (d, *J* = 9.8 Hz), 134.05, 132.15, 130.08, 128.98, 128.90, 128.85, 128.81, 128.76, 128.67, 128.23, 128.16, 128.09, 128.03, 127.72, 127.55, 127.45, 127.19, 126.69, 126.10, 113.75, 87.71 (d, *J* = 10.4 Hz), 87.02 (d, *J* = 8.8 Hz), 61.85 (d, *J* = 3.8 Hz), 33.29 (d, *J* = 147.4 Hz), 27.12, 26.17.

^{31}P NMR (162 MHz, DMSO-d_6) δ 20.30.

HRMS (ESI+): calculated m/z $[M+H]^+$ for $[C_{47}H_{42}NO_8PH]^+$: 780.2721, found: 780.2723.

d Reaction with other types of chiral H-P(O) compounds

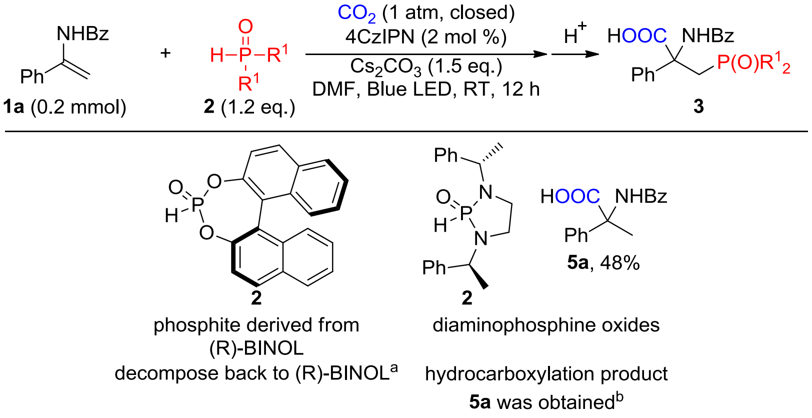

Supplementary Figure 2. Results of reaction with different types of H-P(O) compounds. **a** Phosphite derived from (R)-Binol decomposed back to (R)-Binol and no desired product **3** was detected **b** hydrocarboxylation product **5a** was obtained in 48% yield and no desired product **3** was detected

e Reaction with more steric hindrance chiral H-P(O) compound derived from (4R, 5R)-Taddol[a]

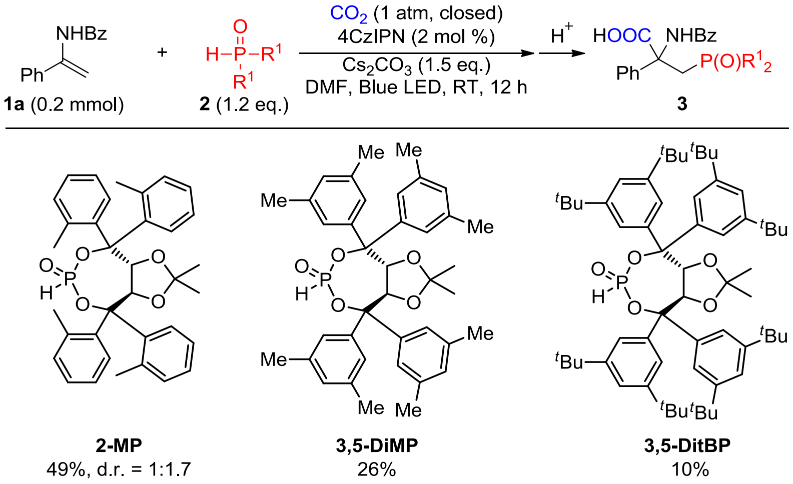

Supplementary Figure 3. Increasing the steric hindrance of H-P(O) compounds. [a]The yields of the products decreased significantly with the increasing of the steric hindrance. The reaction mixtures for 3,5-DiMP and 3,5-DitMP are too complicated and messy to figure out the correspoding d.r. value.

Mechanistic Studies

Trapping with TEMPO

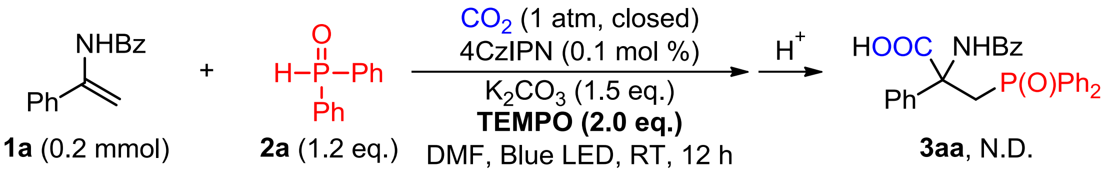

An oven-dried Schlenk tube (10 mL) containing a stirring bar was charged with the substrate (45 mg, 0.2 mmol) and TEMPO (62 mg, 0.4 mmol). The Schlenk tube was then introduced in a glovebox, where it was charged with H-P(O)Ph$_2$ (49 mg, 0.24 mmol, 1.2 eq.) and K$_2$CO$_3$ (41 mg,

0.3 mmol, 1.5 eq.). The tube was taken out of the glovebox and connected to a vacuum line where it was evacuated and back-filled with CO_2 for 3 times. Then DMF (2 mL) and 4CzIPN (32 µL, 0.1 mol %, 5 mg dissolved in 1 mL DMF) were added under CO_2 flow. Finally, the reaction mixture in sealed tube was placed at a distance of 2 ~ 3 cm from a 30 W blue LED and stirred at room temperature (25 ℃) for 12 h.

Radical clock test

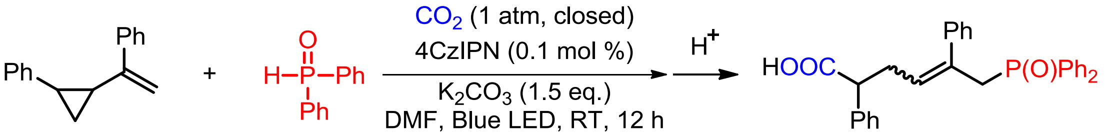

An oven-dried Schlenk tube (10 mL) containing a stirring bar was charged with the substrate **16** (0.2 mmol). The Schlenk tube was then introduced in a glovebox, where it was charged with H-P(O) compound **2a** (49 mg, 0.24 mmol, 1.2 eq.) and K_2CO_3 (41 mg, 0.3 mmol, 1.5 eq.). The tube was taken out of the glovebox and connected to a vacuum line where it was evacuated and back-filled with CO_2 for 3 times. Then DMF (2 mL), 4CzIPN (32 µL, 0.1 mol %, 5 mg dissolved in 1 mL DMF) were added under CO_2 flow. Finally, the reaction mixture in sealed tube was placed at a distance of 2 ~ 3 cm from a 30 W blue LED and stirred at room temperature (25 ℃) for 12 h. Then, the mixture was quenched with 4.5 mL of H_2O and 0.5 mL of 2N HCl (aq.), extracted with ethyl acetate (EA) for at least 5 times, then the organic phase was concentrated in vacuo. The residue was purified by silica gel flash chromatography (PE/EA 2/1 ~ 1/1) to give 58 mg desired product **17**.

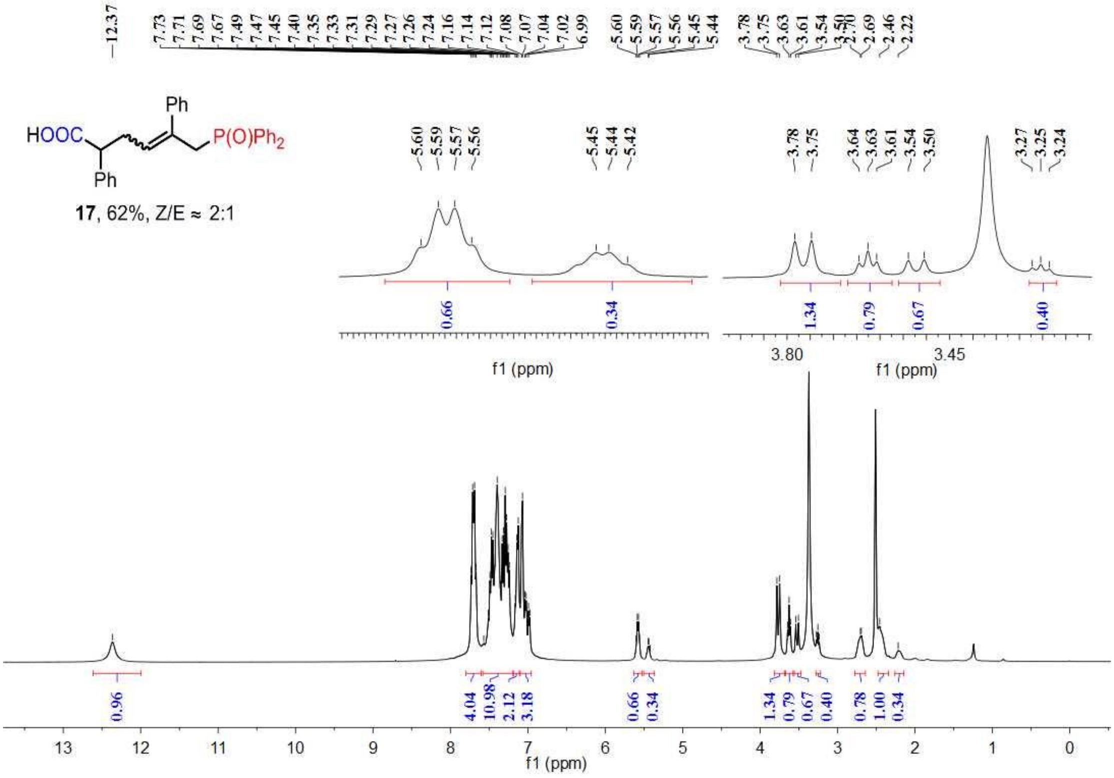

Supplementary Figure 4. ^{1}H NMR Spectra of compound **17**

Isotope labelling with D_2O

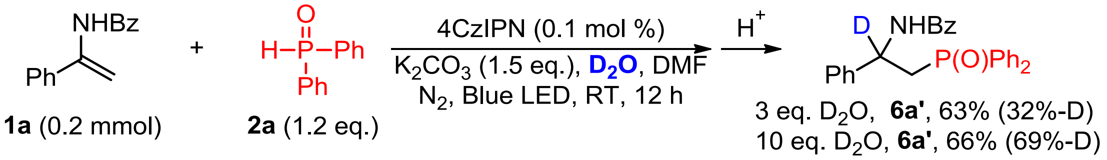

An oven-dried Schlenk tube (10 mL) containing a stirring bar was charged with the substrate **1a** (0.2 mmol). The Schlenk tube was then introduced in a glovebox, where it was charged with H-P(O) compound **2a** (49 mg, 0.24 mmol, 1.2 eq.) and K_2CO_3 (41 mg, 0.3 mmol, 1.5 eq.). The tube was taken out of the glovebox and connected to a vacuum line where it was evacuated and back-filled with N_2 for 3 times. Then DMF (2 mL), 4CzIPN (32 μL, 0.1 mol %, 5 mg dissolved in 1 mL DMF) and D_2O (11 μL, 3 eq. or 36.7 μL, 10 eq.) were added under N_2 flow. Finally, the reaction mixture in sealed tube was placed at a distance of 2 ~ 3 cm from a 30 W blue LED and stirred at room temperature (25 ℃) for 12 h. Then, the mixture was quenched with 5 mL of H_2O, extracted with Ethyl Acetate for at least 3times, then concentrated in vacuo. The residue was purified by silica gel flash chromatography (Petroleum ether/AcOEt 2/1 ~ 1/1) to give the D-labelled product.

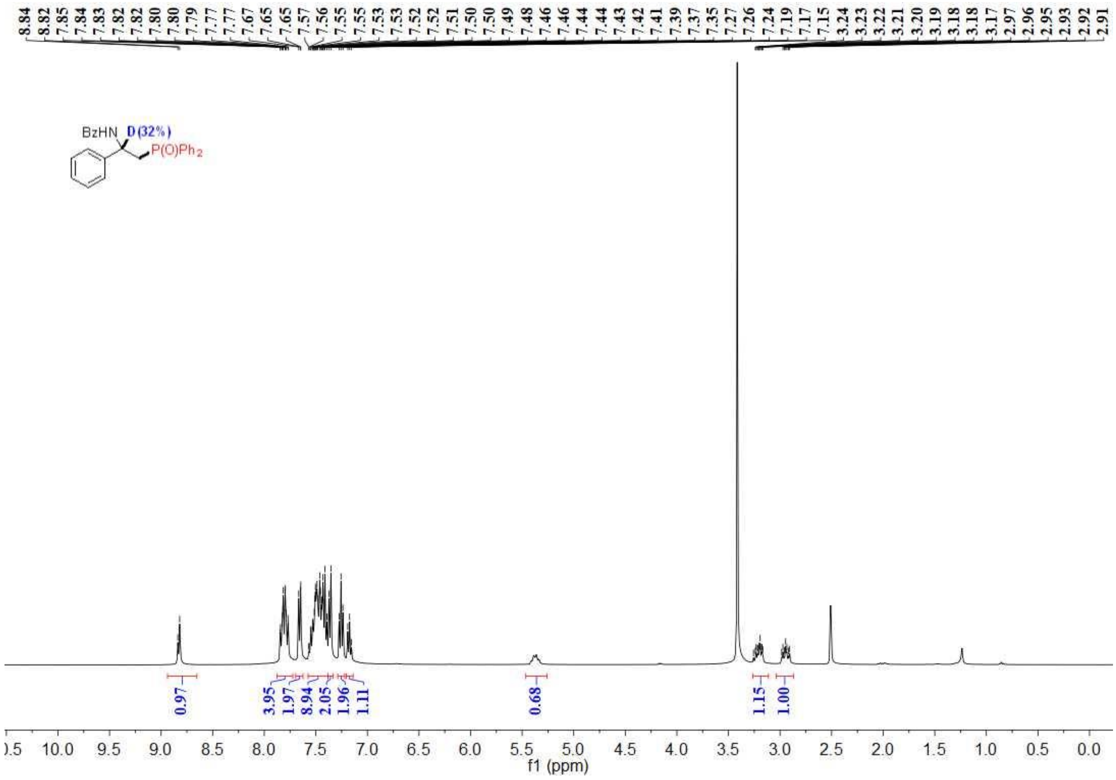

Supplementary Figure 5. ^{1}H NMR Spectra of compound **6a’** (3 eq. D_2O)

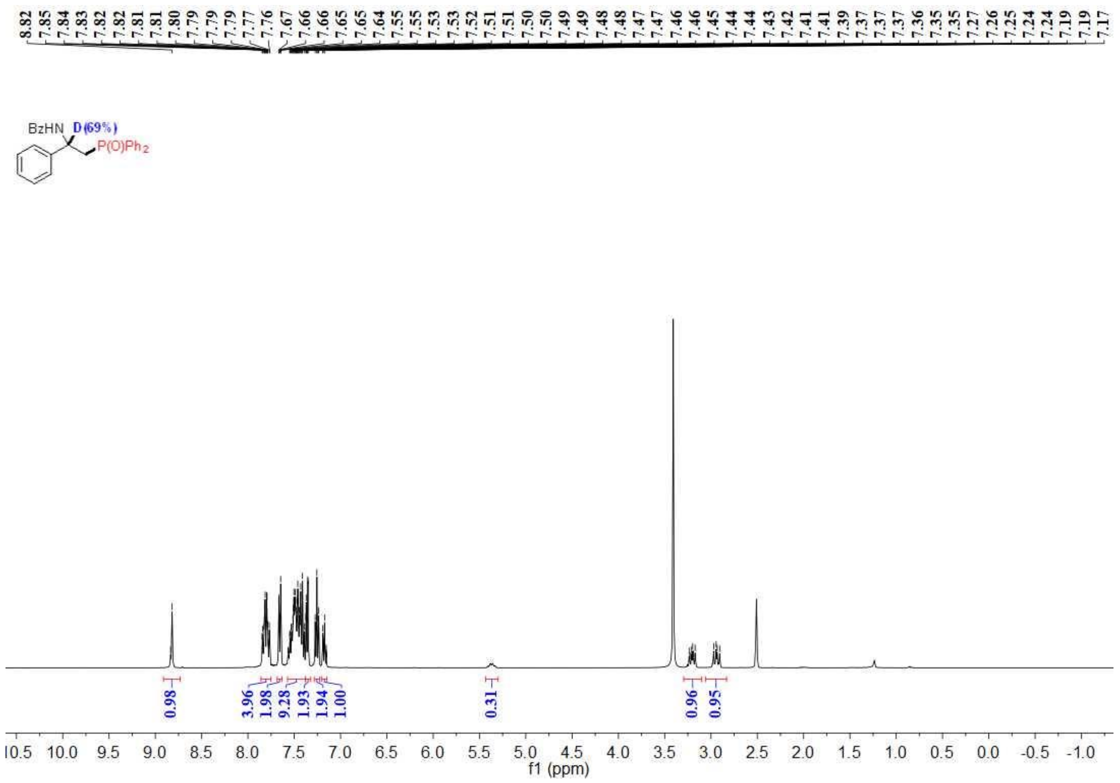

Supplementary Figure 6. ^{1}H NMR Spectra of compound **6a'** (10 eq. D_2O)

Stern-Volmer emission quenching

Fluorescence spectra were collected on Fluorolog Horiba Jobin Yvon spectrofluorimeter. Samples for the quenching experiments were prepared in a 4 mL quartz cuvette with a cap. 4CzIPN was irradiated at 440 nm and the emission intensity at about 540 nm was observed. In a typical experiment, the emission spectrum of a 10^{-5} M solution of 4CzIPN in DMF was collected.

(a) Stern-Volmer Plot with enamides 1a:

N-(1-phenylvinyl)benzamide **1a**: A stock solution of **1a** (27.9 mg, 0.125 mmol) in 25 mL of DMF was prepared. Then, different amounts of this stock solution were added to 3.0 mL of 4CzIPN in DMF (10^{-5} M).

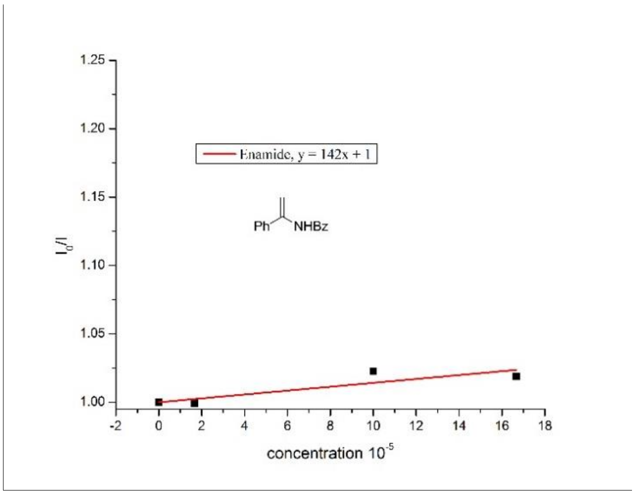

Supplementary Figure 7. Stern-Volmer quenching plot of enamide **1a**

(b) Stern-Volmer Plot with 2a + K_2CO_3:

N-(1-phenylvinyl)benzamide **1a**: A stock solution of **1a** (27.9 mg, 0.125 mmol) and K_2CO_3 100 mg in 25 mL of DMF was prepared. Then, different amounts of this stock solution were added to 3.0 mL of 4CzIPN in DMF (10^{-5} M).

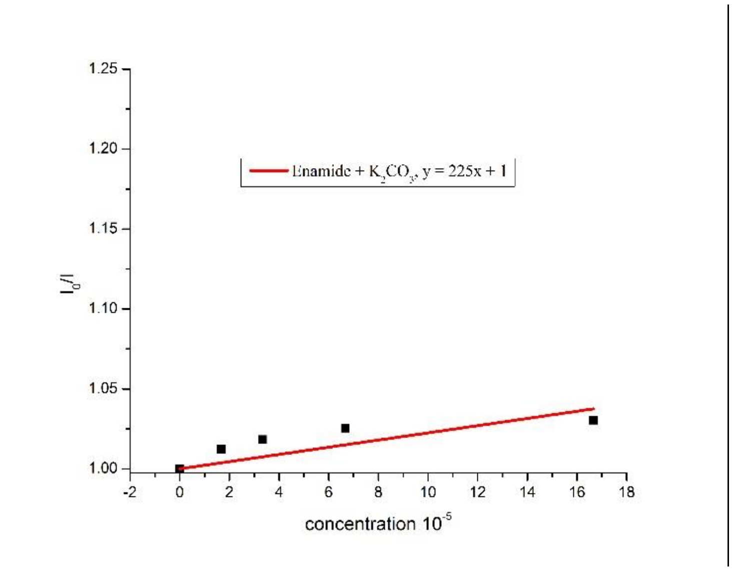

Supplementary Figure 8. Stern-Volmer quenching plot of enamide **1a** with K_2CO_3

(c) Stern-Volmer Plot with HP(O)Ph$_2$ 2a:

A stock solution of **2a** (25.3 mg, 0.125 mmol) in 25 mL of DMF was prepared. Then, different amounts of this stock solution were added to 3.0 mL of 4CzIPN in DMF (10^{-5} M).

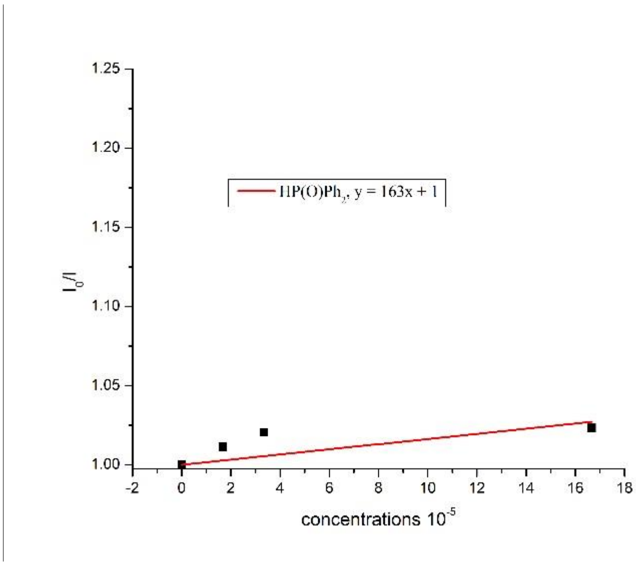

Supplementary Figure 9. Stern-Volmer quenching plot of HP(O)Ph$_2$ **2a**

(d) Stern-Volmer Plot with HP(O)Ph$_2$ 2a + K_2CO_3:

A stock solution of **2a** (25.3 mg, 0.125 mmol) and K_2CO_3 100mg in 25 mL of DMF was prepared. Then, different amounts of this stock solution ere added to 3.0 mL of 4CzIPN in DMF (10^{-5} M).

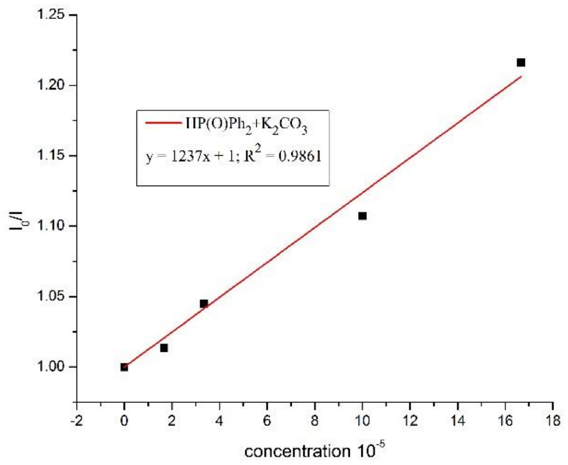

Supplementary Figure 10. Stern-Volmer quenching plot of $HP(O)Ph_2$ **2a** with K_2CO_3

(e)

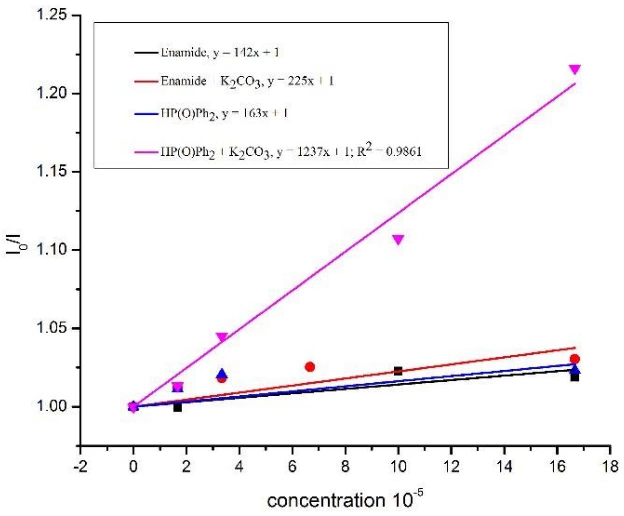

Supplementary Figure 11. Summary of the Stern-Volmer quenching plots

The luminescence of 4CzIPN at λ_{max} = 537 nm was quenched more significantly by H-P(O)Ph_2 in the presence of K_2CO_3 than by enamide **1a**. Based on the luminescence quenching studies, we proposed that a reductive quenching step was involved in the reaction.

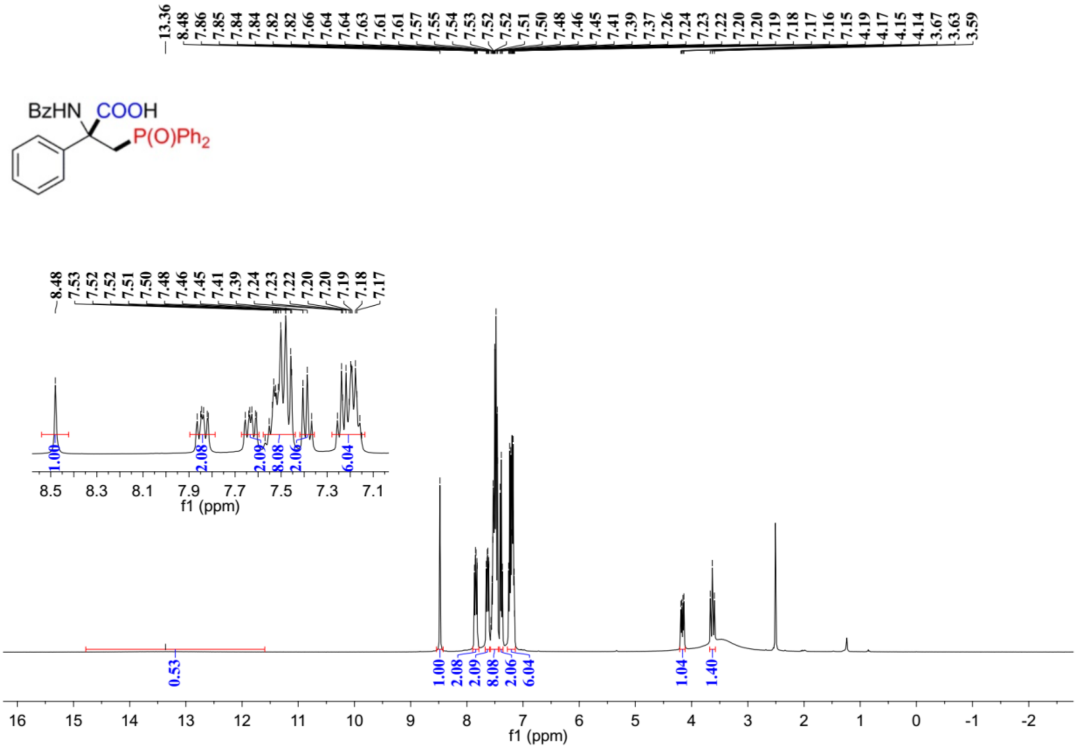

Supplementary Figure 12. ^{1}H NMR spectra of **3aa**

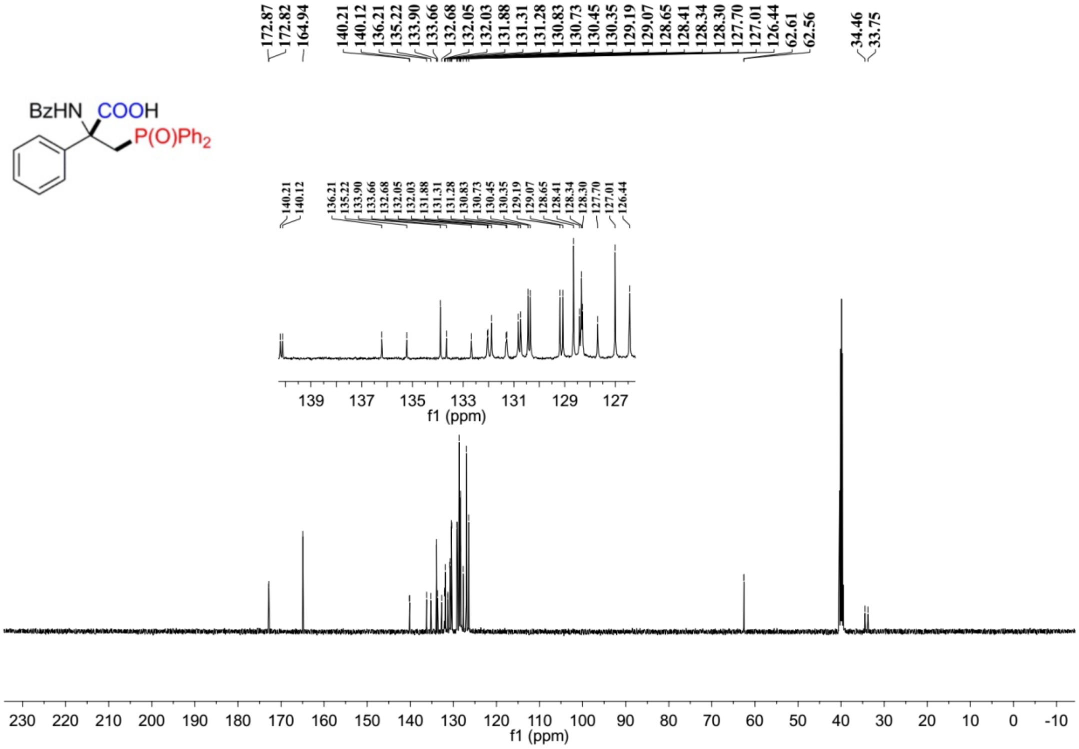

Supplementary Figure 13. ^{13}C NMR spectra of **3aa**

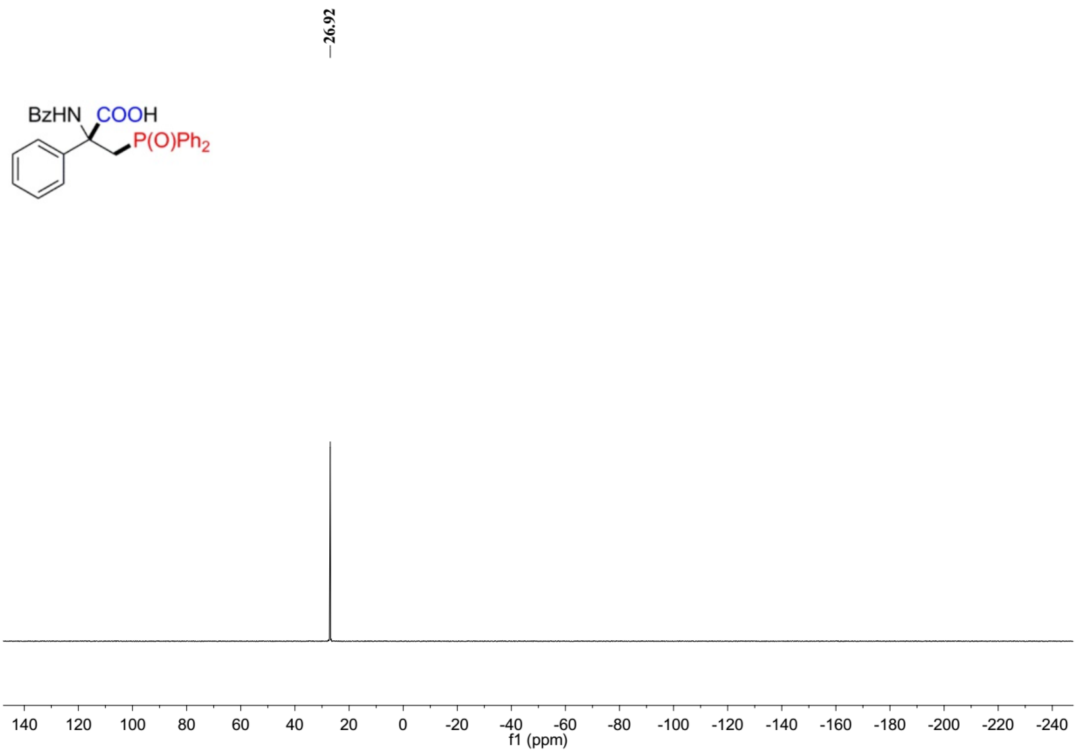

Supplementary Figure 14. ^{31}P NMR spectra of **3aa**

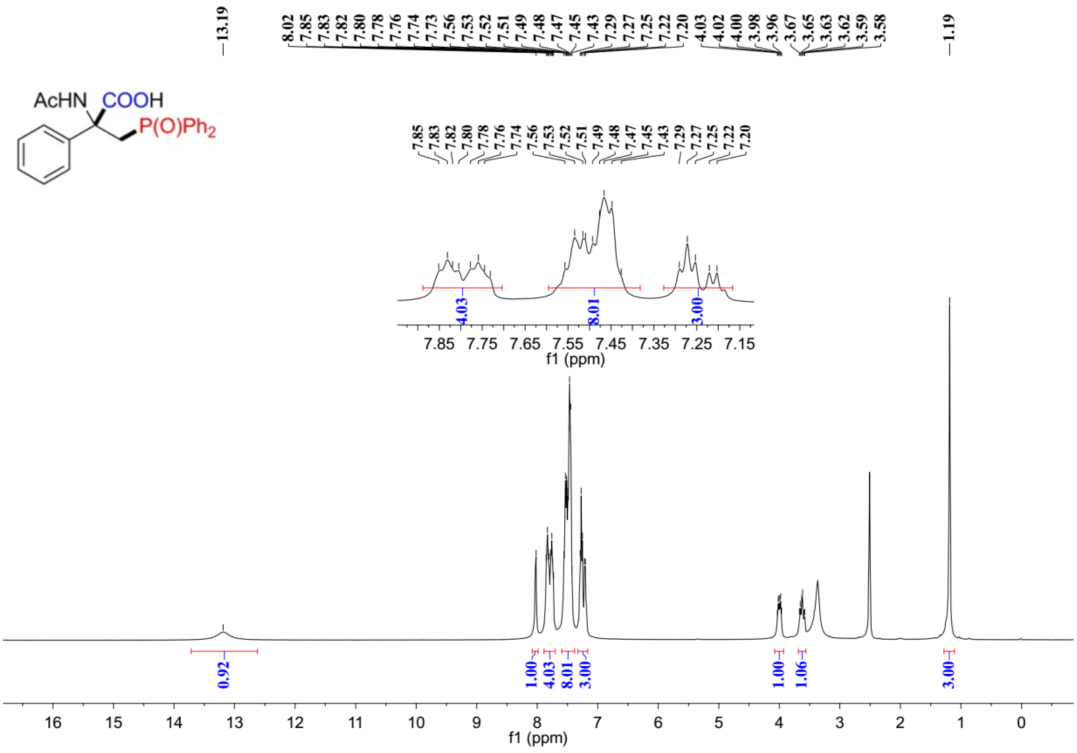

Supplementary Figure 15. ^{1}H NMR spectra of **3ba**

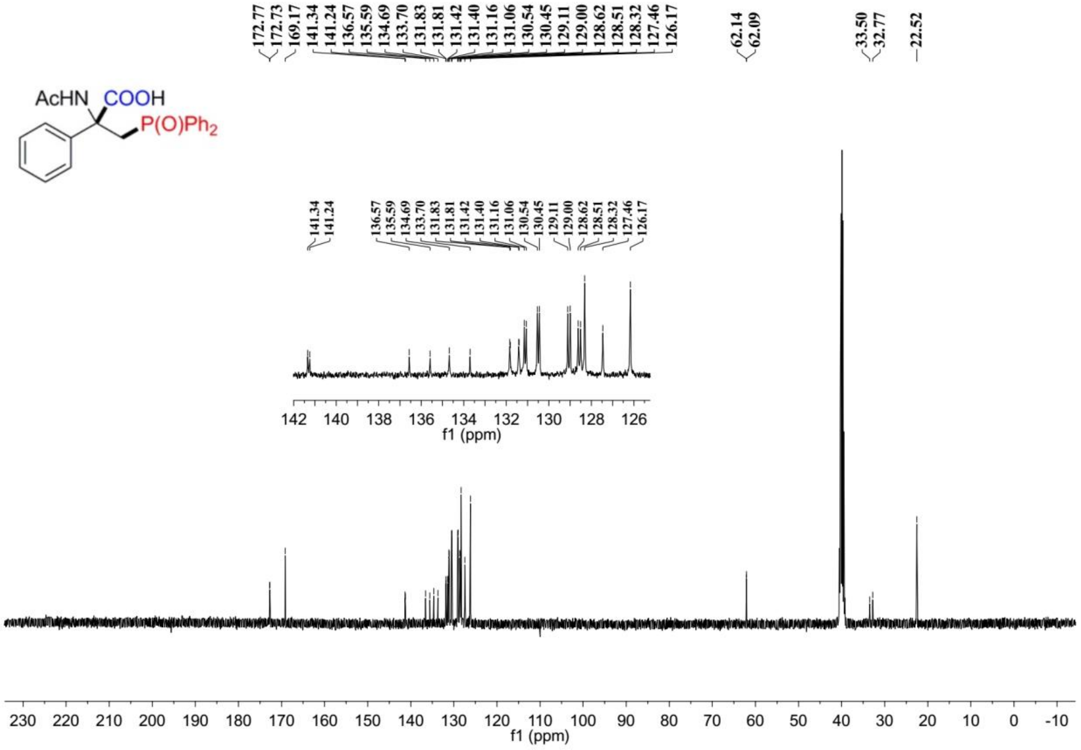

Supplementary Figure 16. ^{13}C NMR spectra of **3ba**

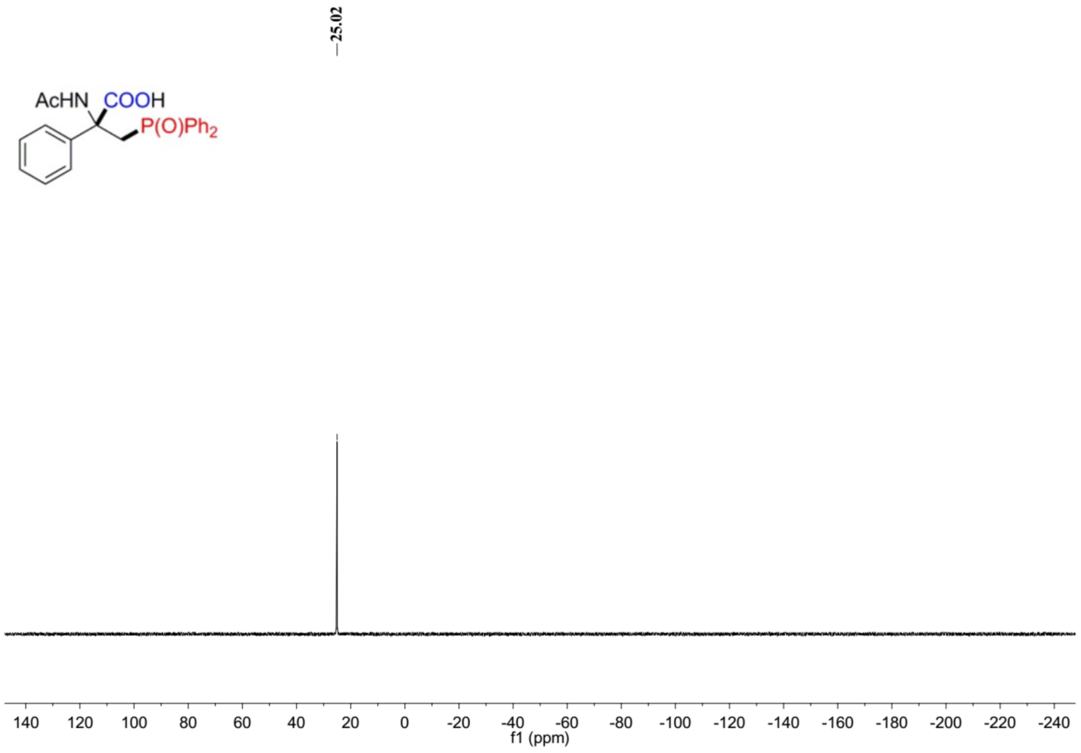

Supplementary Figure 17. ^{31}P NMR spectra of **3ba**

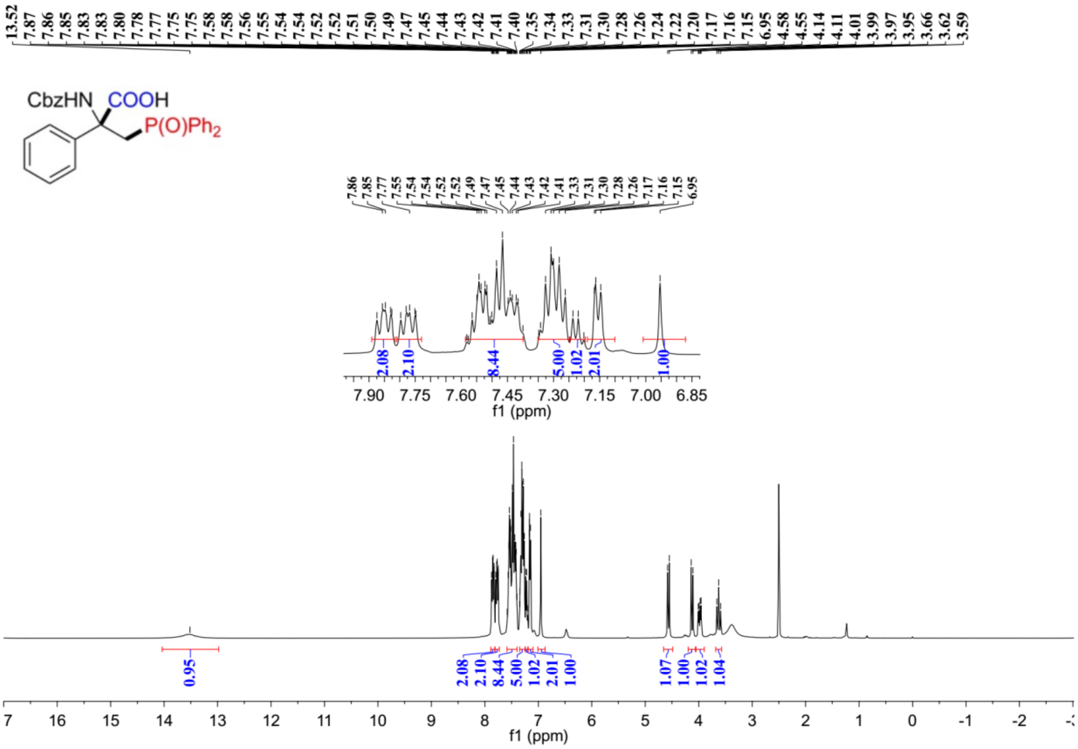

Supplementary Figure 18. ^{1}H NMR spectra of **3ca**

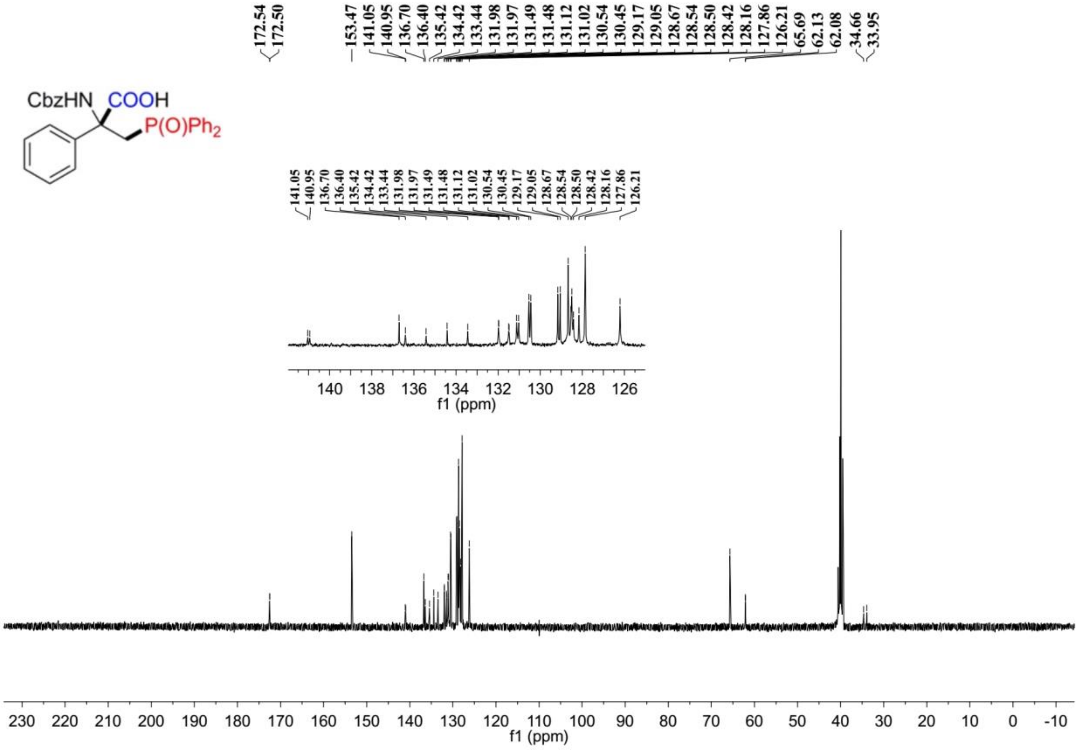

Supplementary Figure 19. ^{13}C NMR spectra of **3ca**

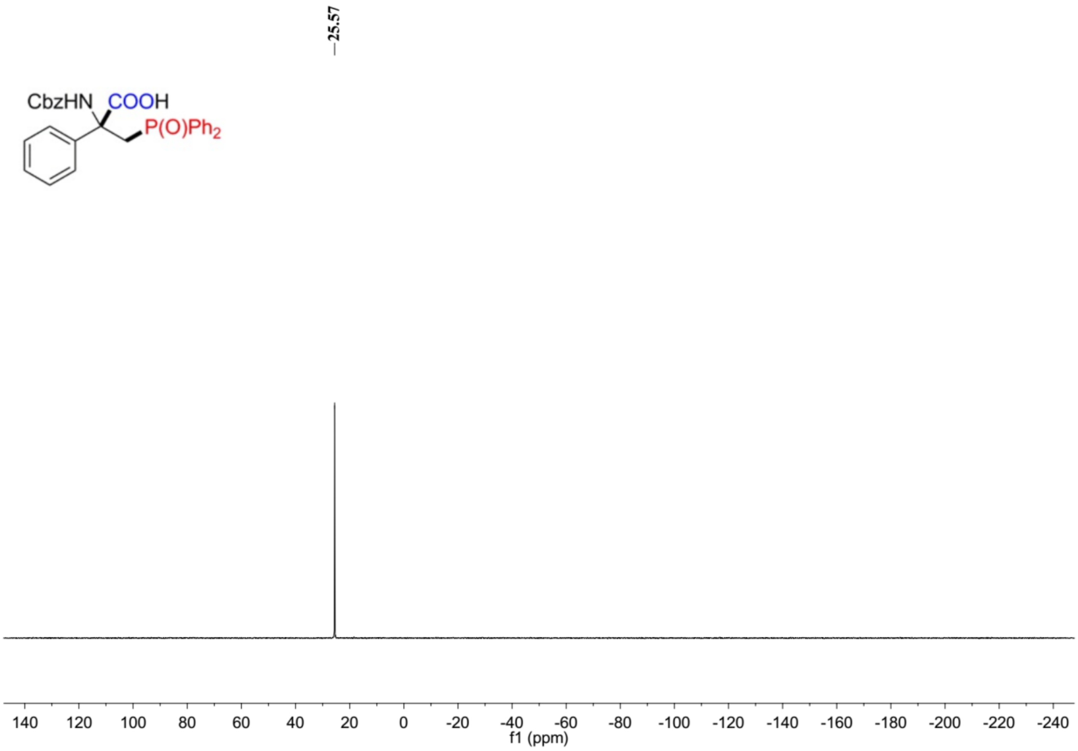

Supplementary Figure 20. ^{31}P NMR spectra of **3ca**

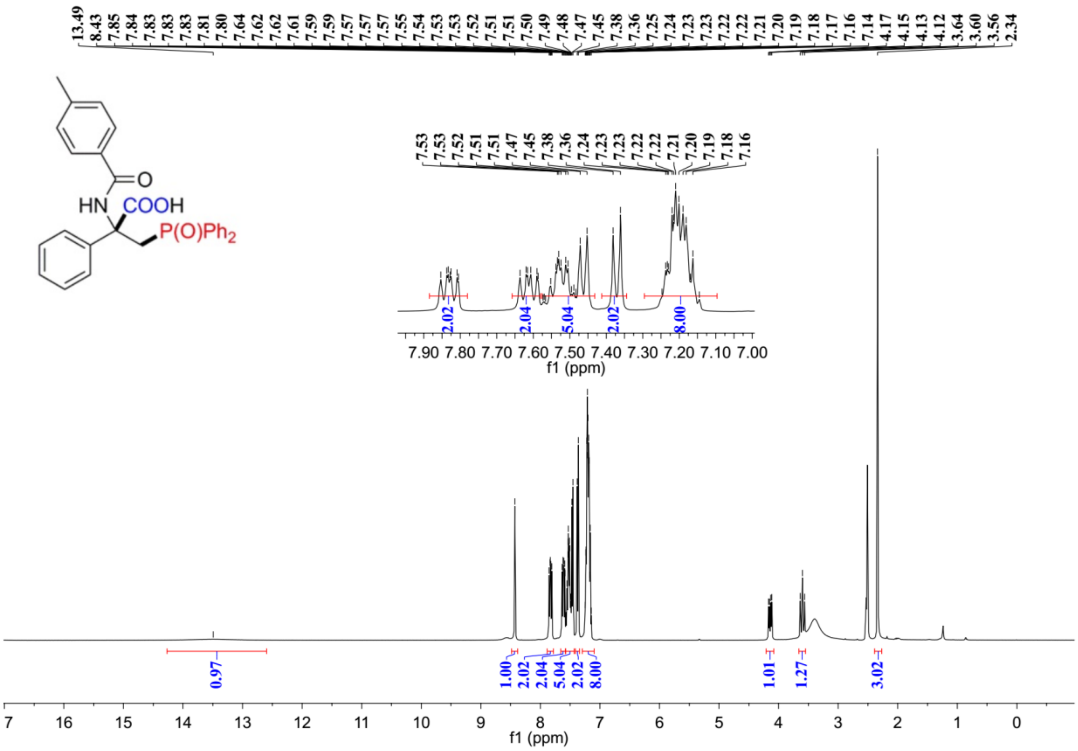

Supplementary Figure 21. ^{1}H NMR spectra of **3da**

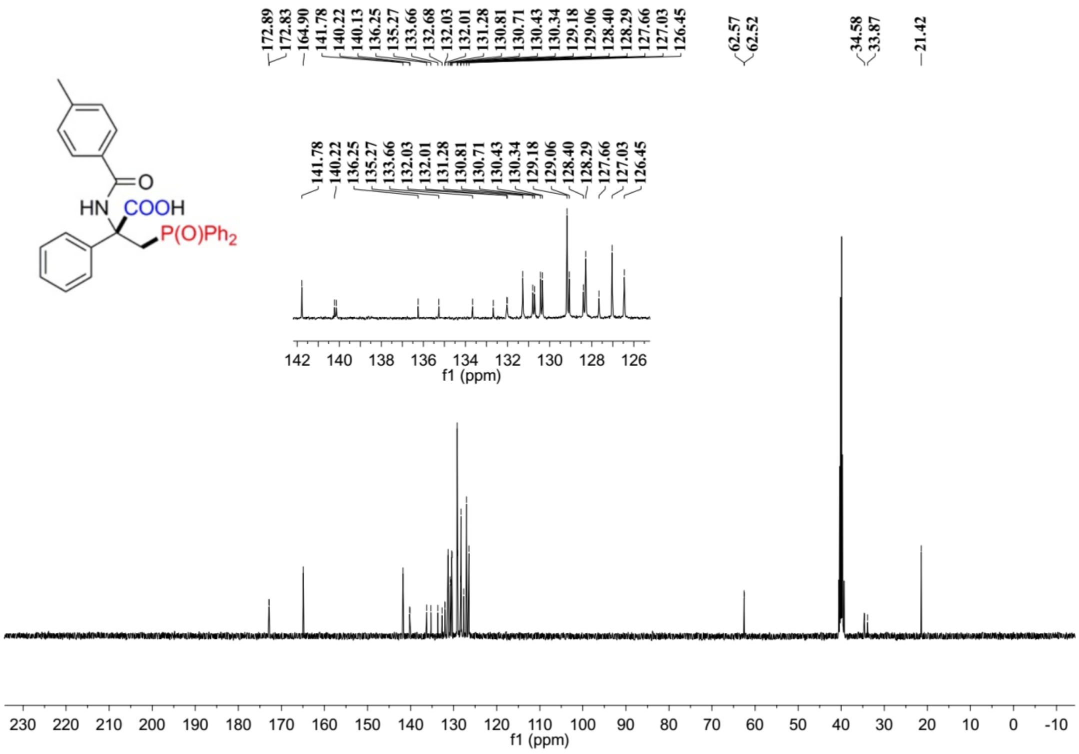

Supplementary Figure 22. ^{13}C NMR spectra of **3da**

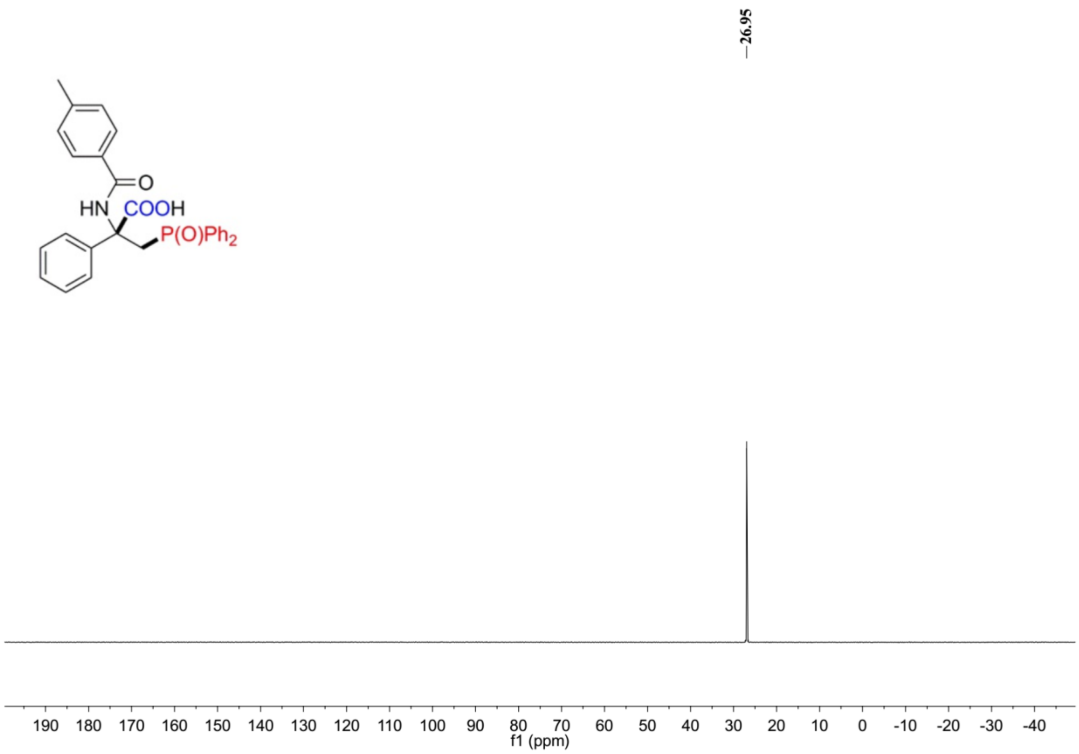

Supplementary Figure 23. ^{31}P NMR spectra of **3da**

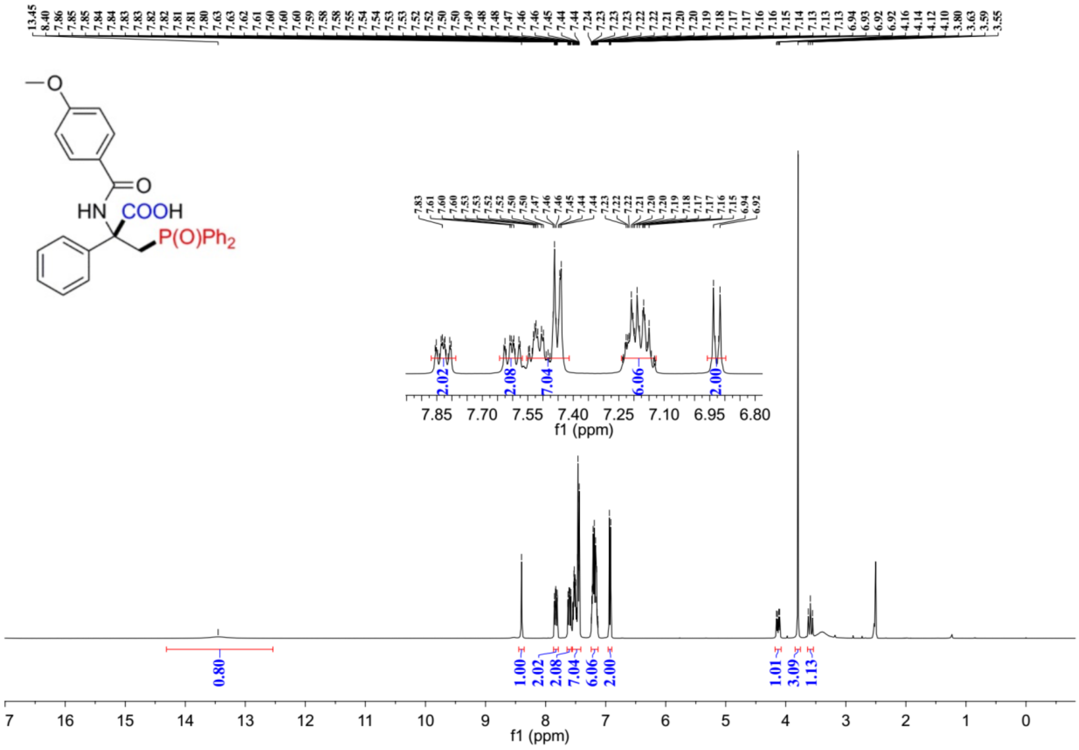

Supplementary Figure 24. ^{1}H NMR spectra of **3ea**

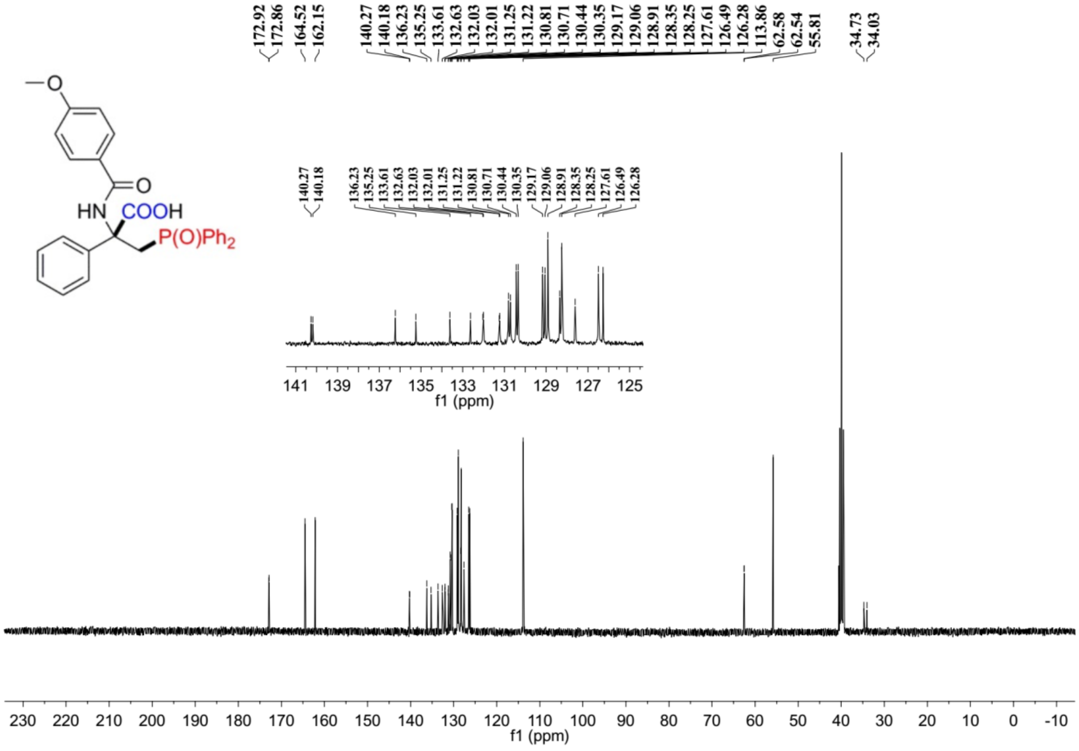

Supplementary Figure 25. ^{13}C NMR spectra of **3ea**

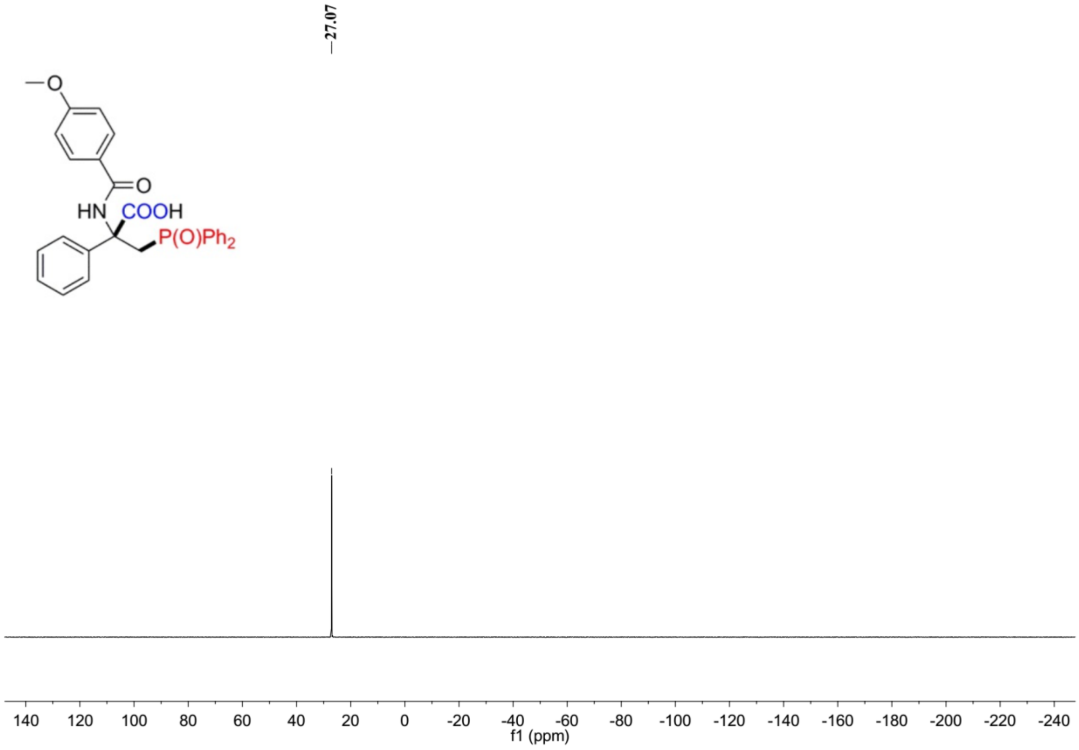

Supplementary Figure 26. ^{31}P NMR spectra of **3ea**

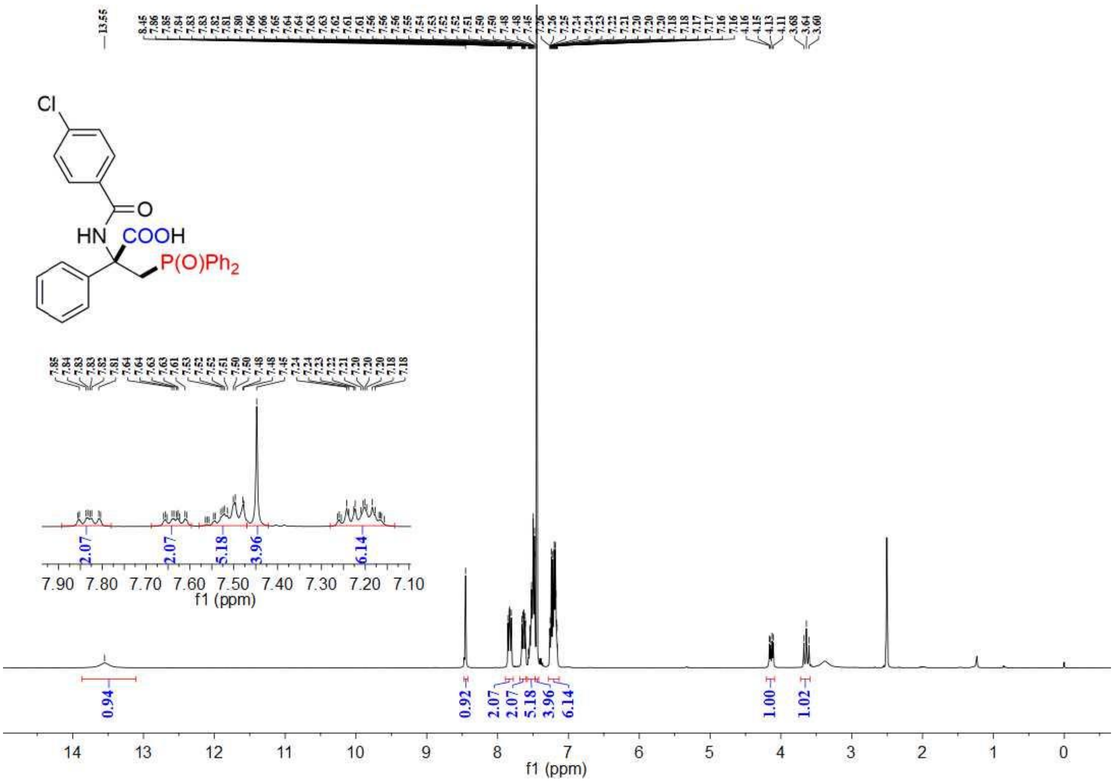

Supplementary Figure 27. ^{1}H NMR spectra of **3fa**

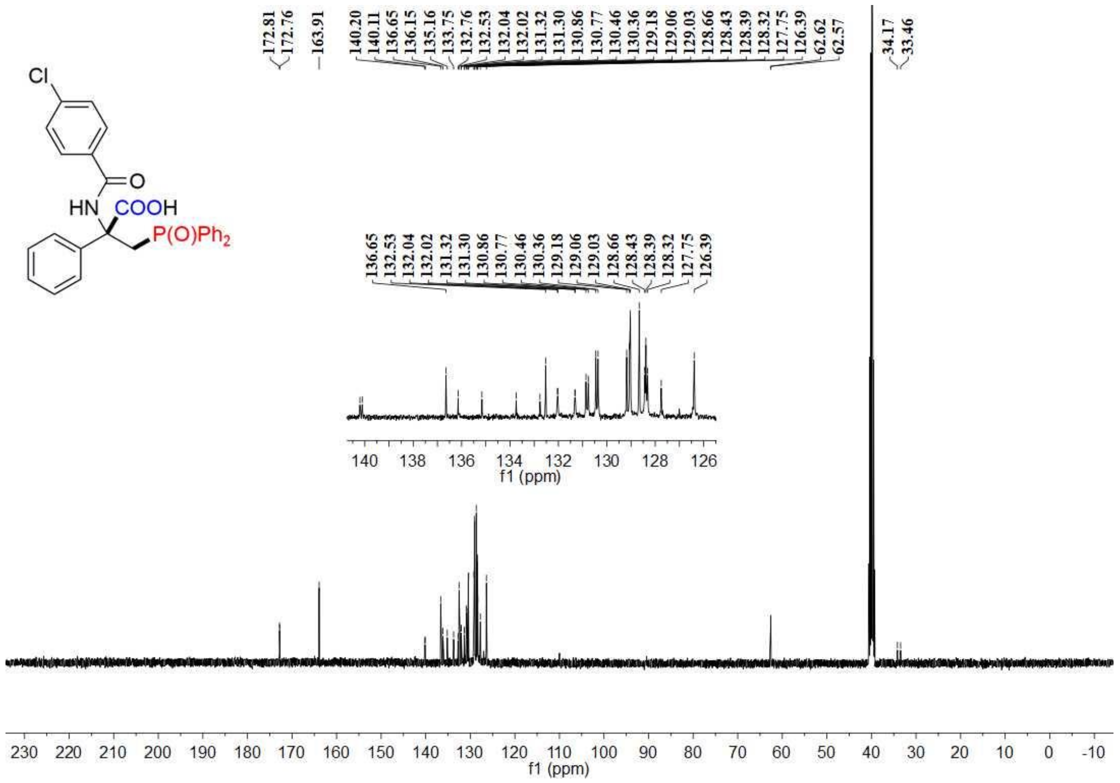

Supplementary Figure 28. ^{13}C NMR spectra of **3fa**

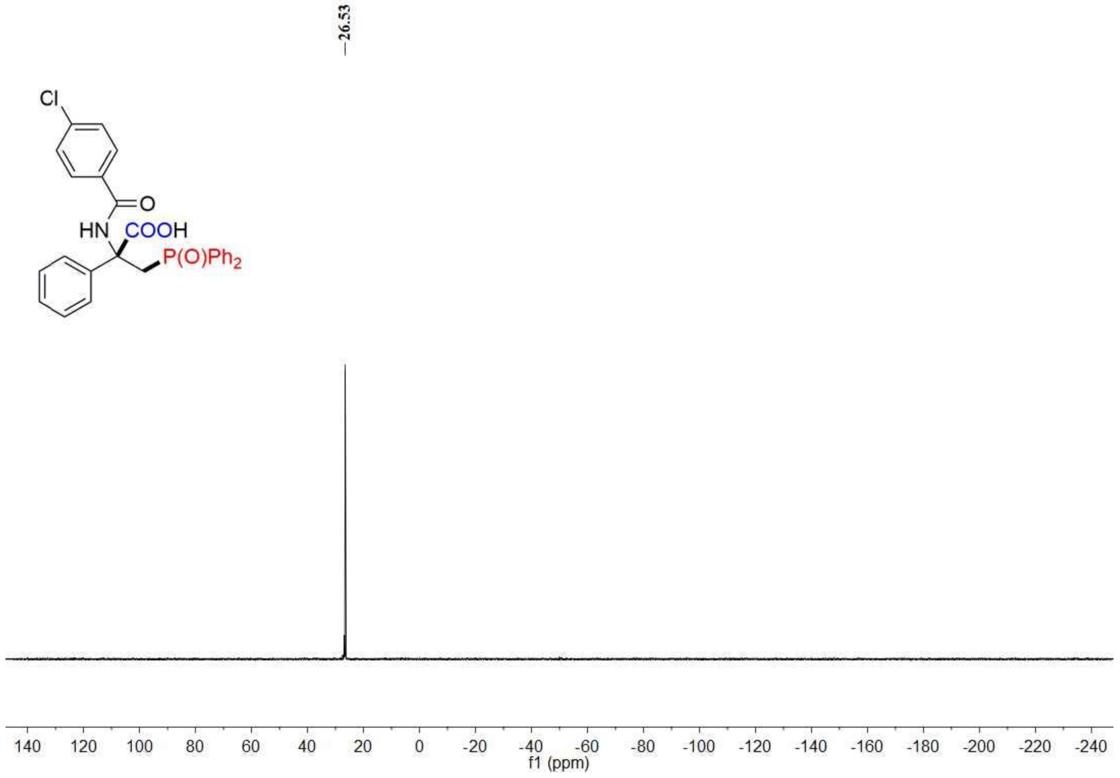

Supplementary Figure 29. ^{31}P NMR spectra of **3fa**

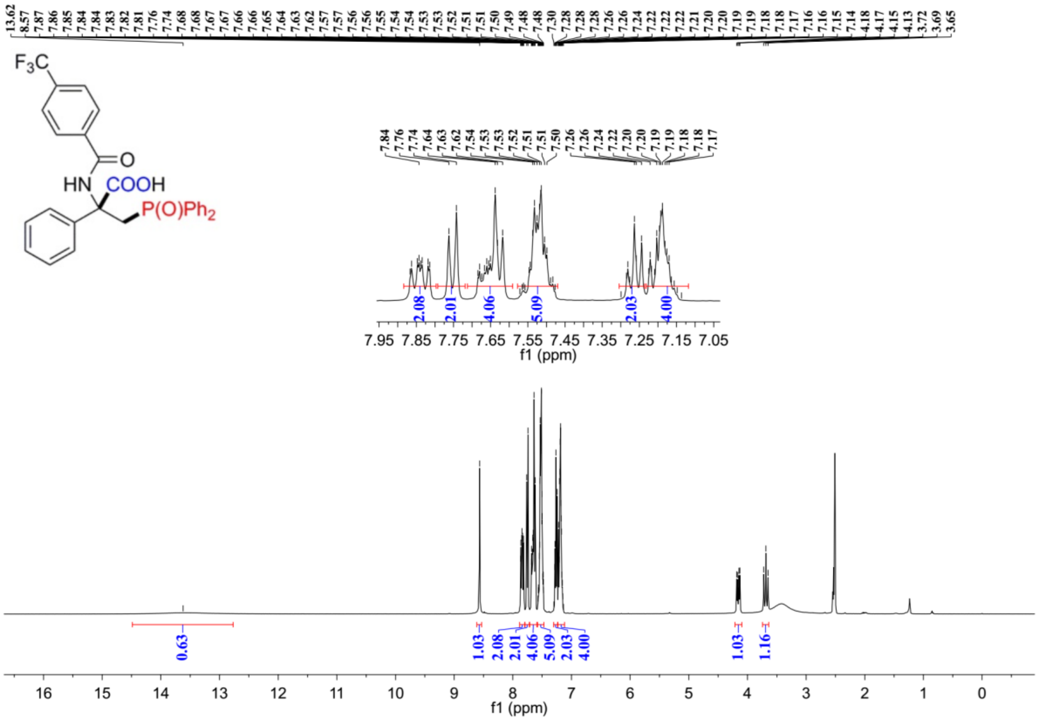

Supplementary Figure 30. ^{1}H NMR spectra of **3ga**

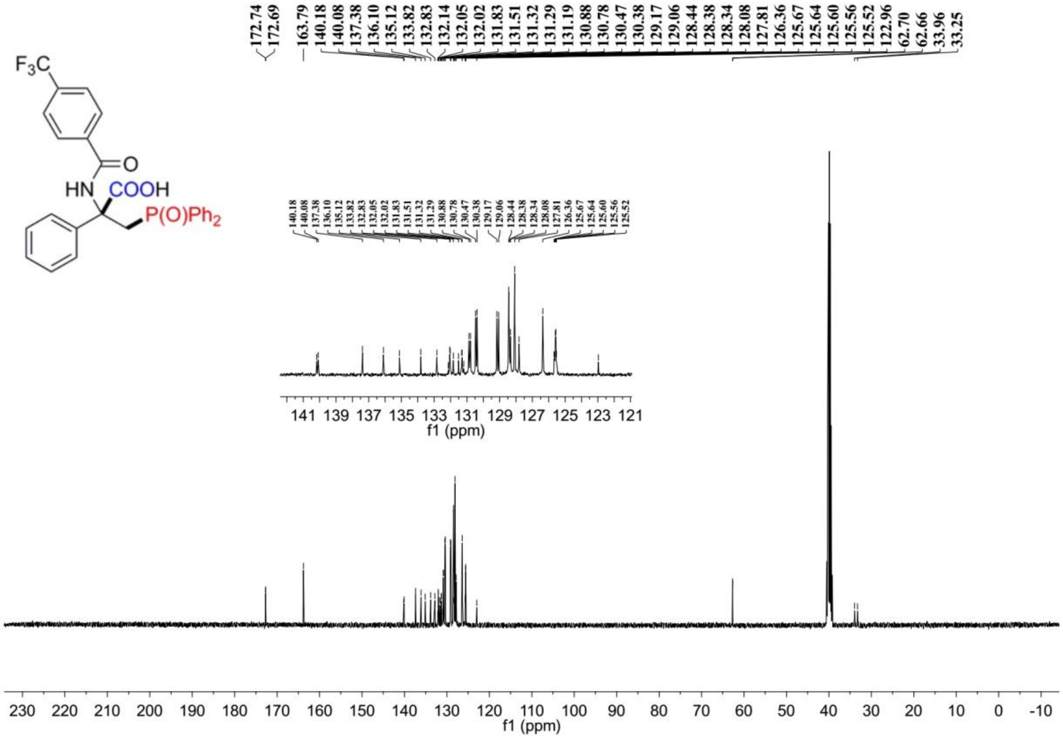

Supplementary Figure 31. ^{13}C NMR spectra of **3ga**

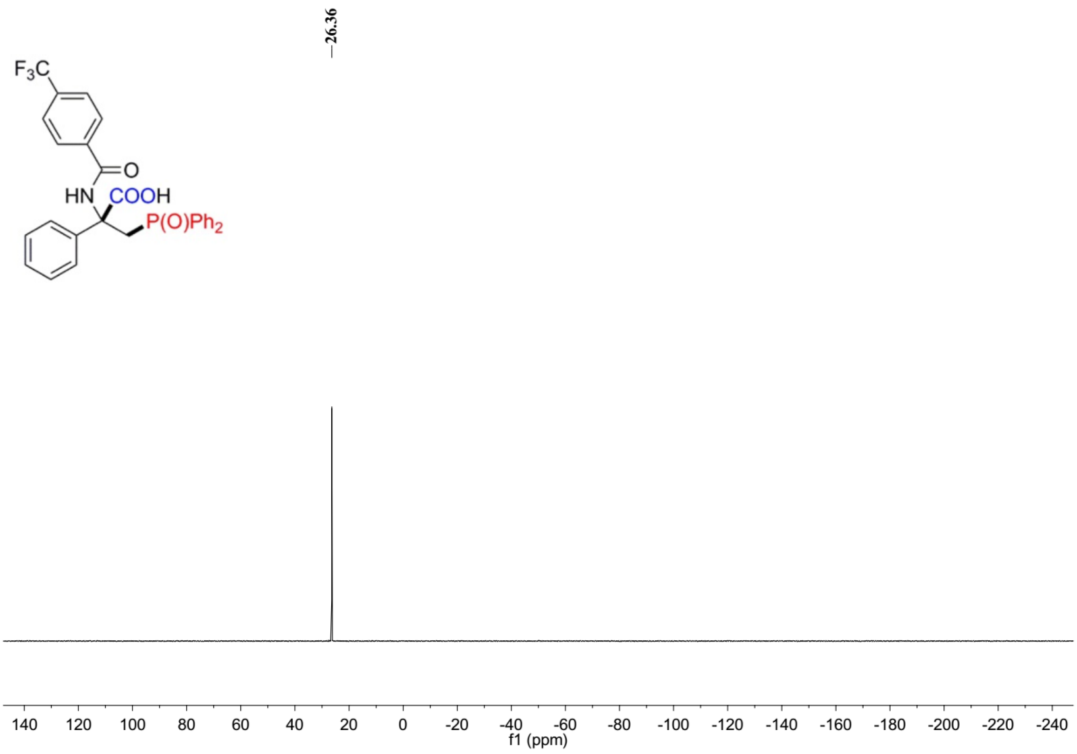

Supplementary Figure 32. ^{31}P NMR spectra of **3ga**

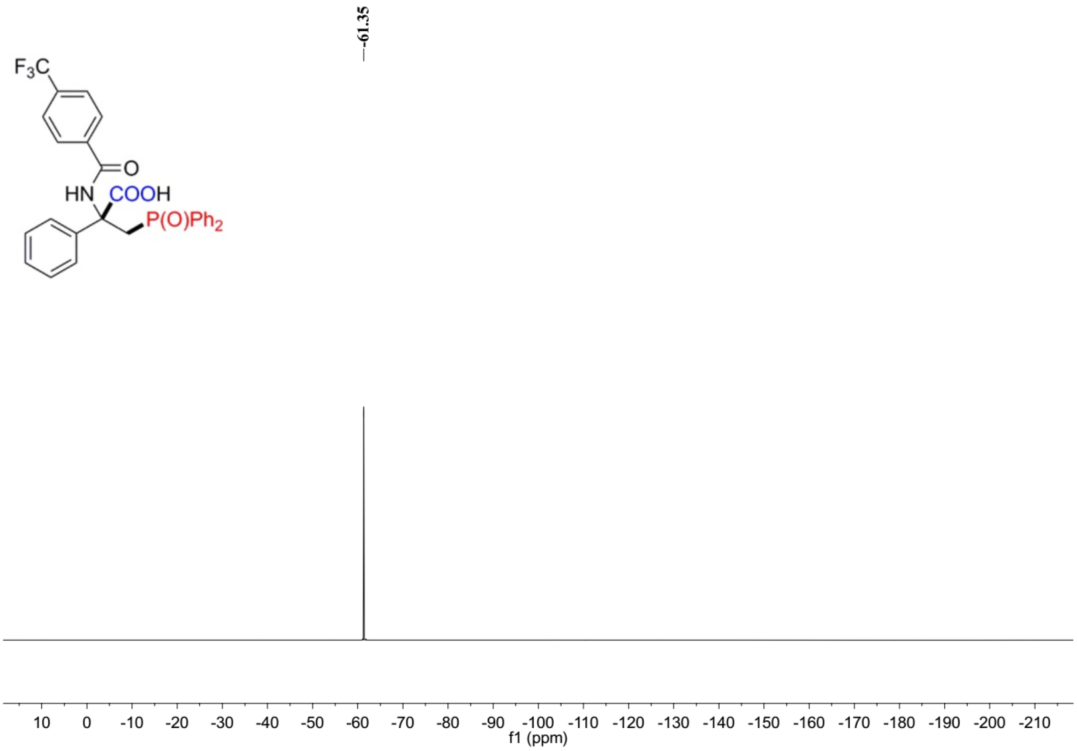

Supplementary Figure 33. ^{19}F NMR spectra of **3ga**

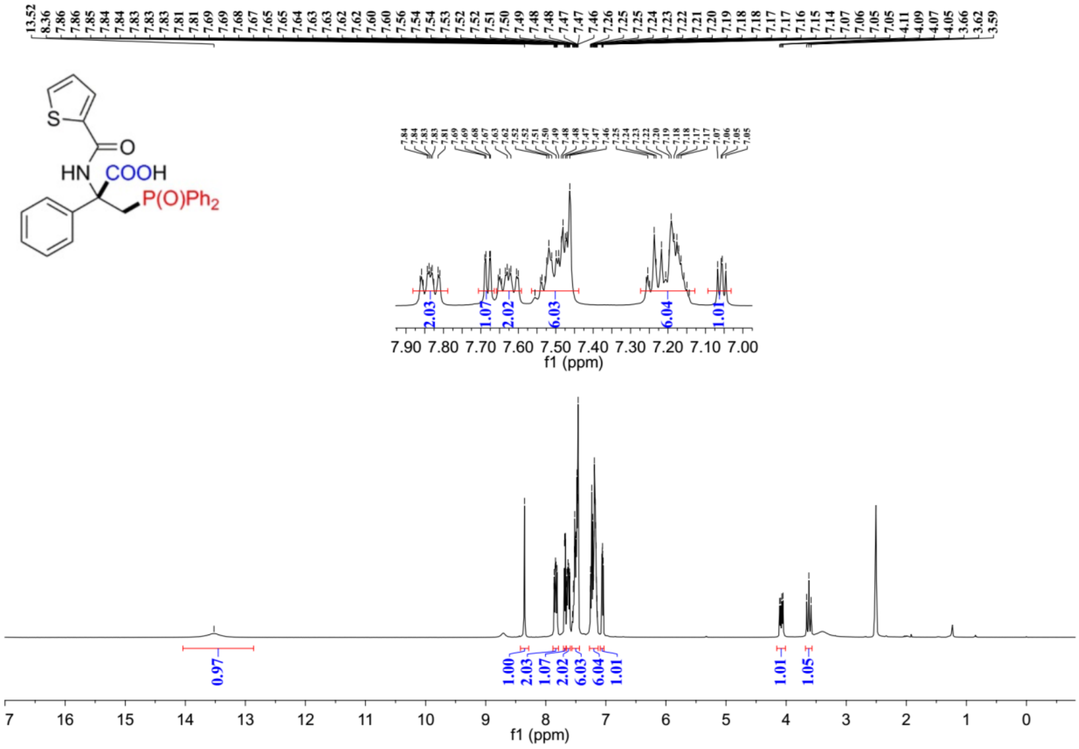

Supplementary Figure 34. ^{1}H NMR spectra of **3ha**

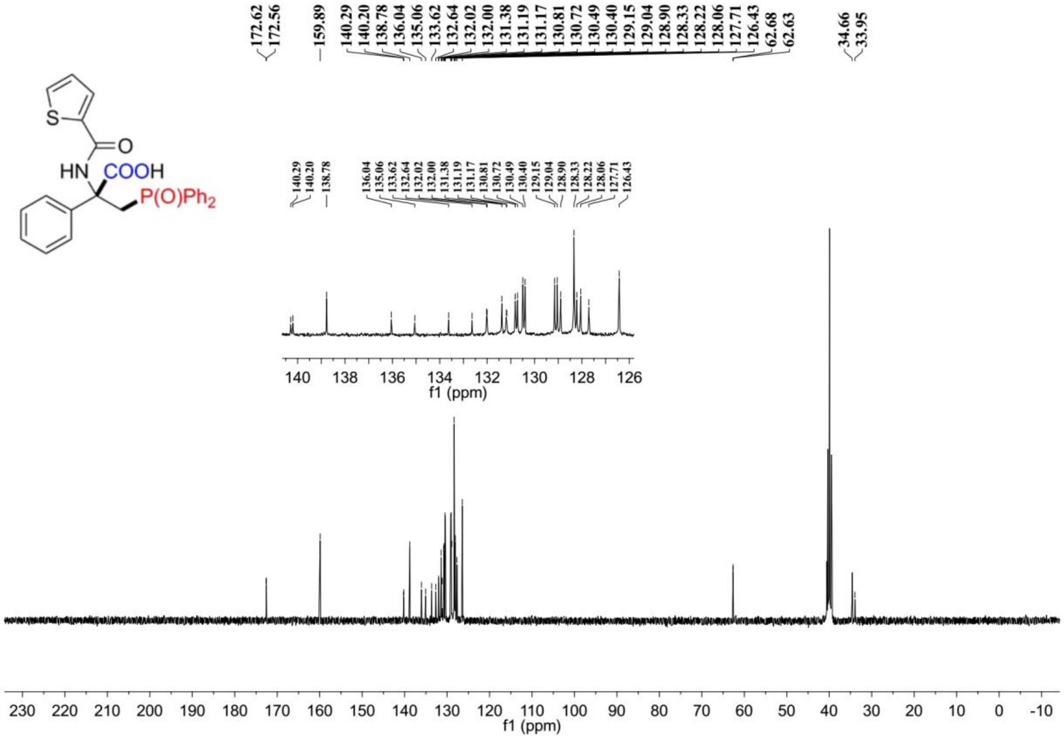

Supplementary Figure 35. ^{13}C NMR spectra of **3ha**

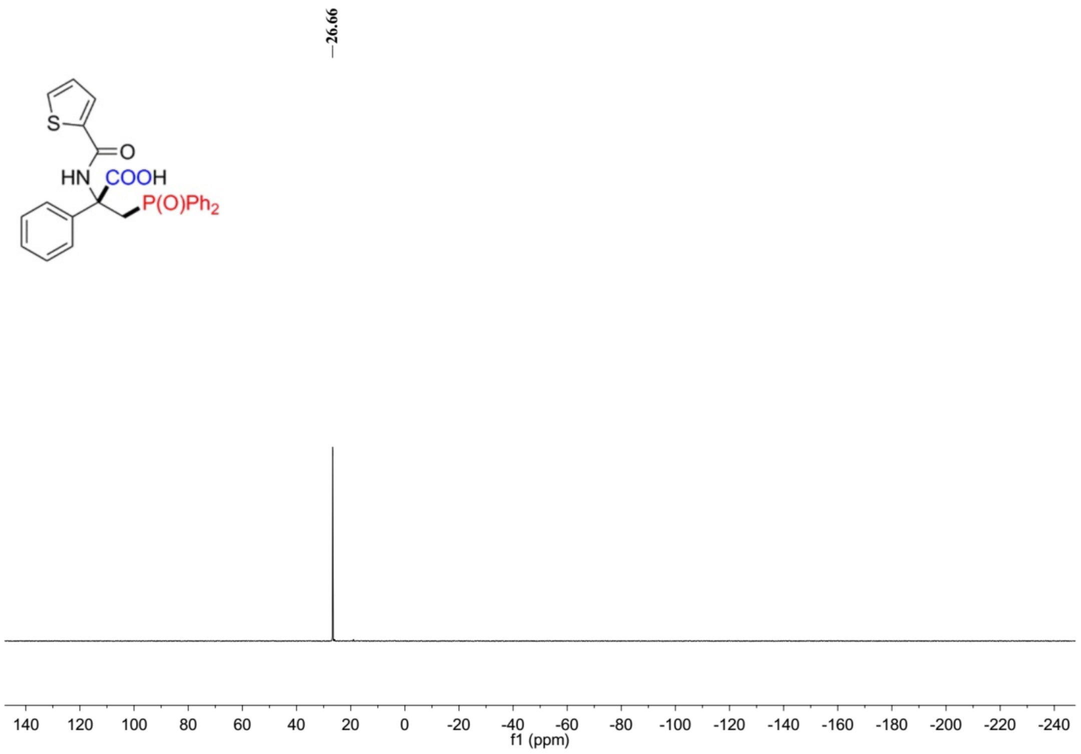

Supplementary Figure 36. ^{31}P NMR spectra of **3ha**

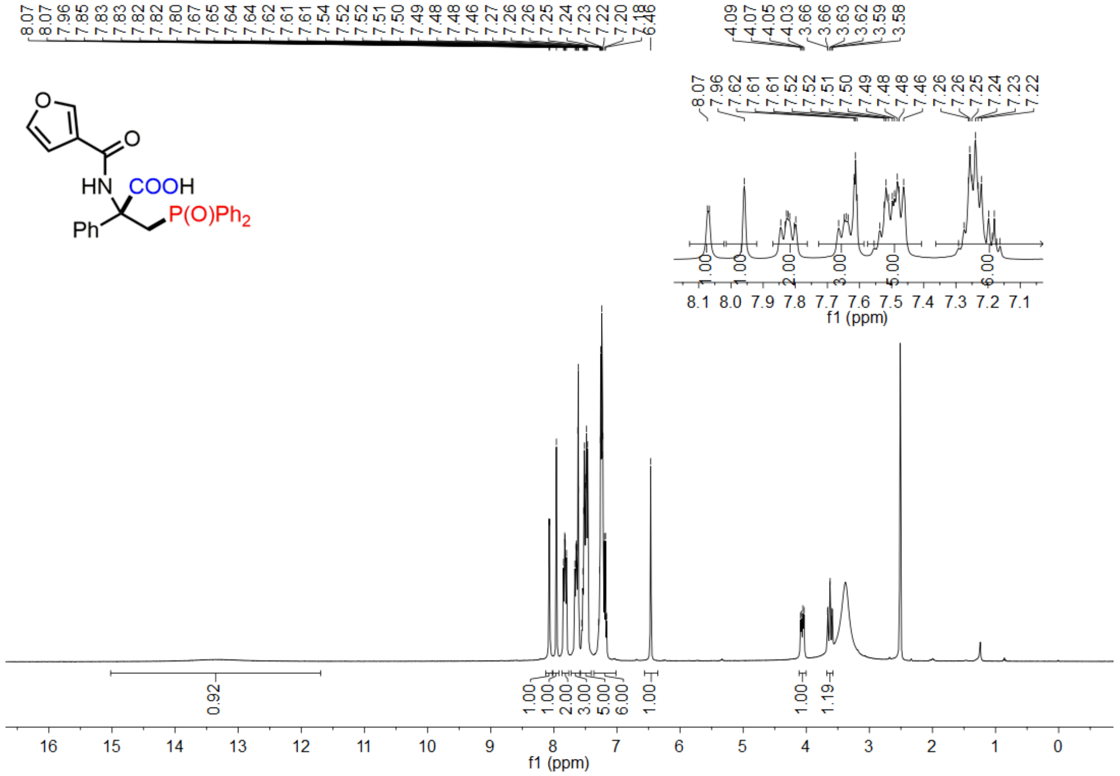

Supplementary Figure 37. ^{1}H NMR spectra of **3ia**

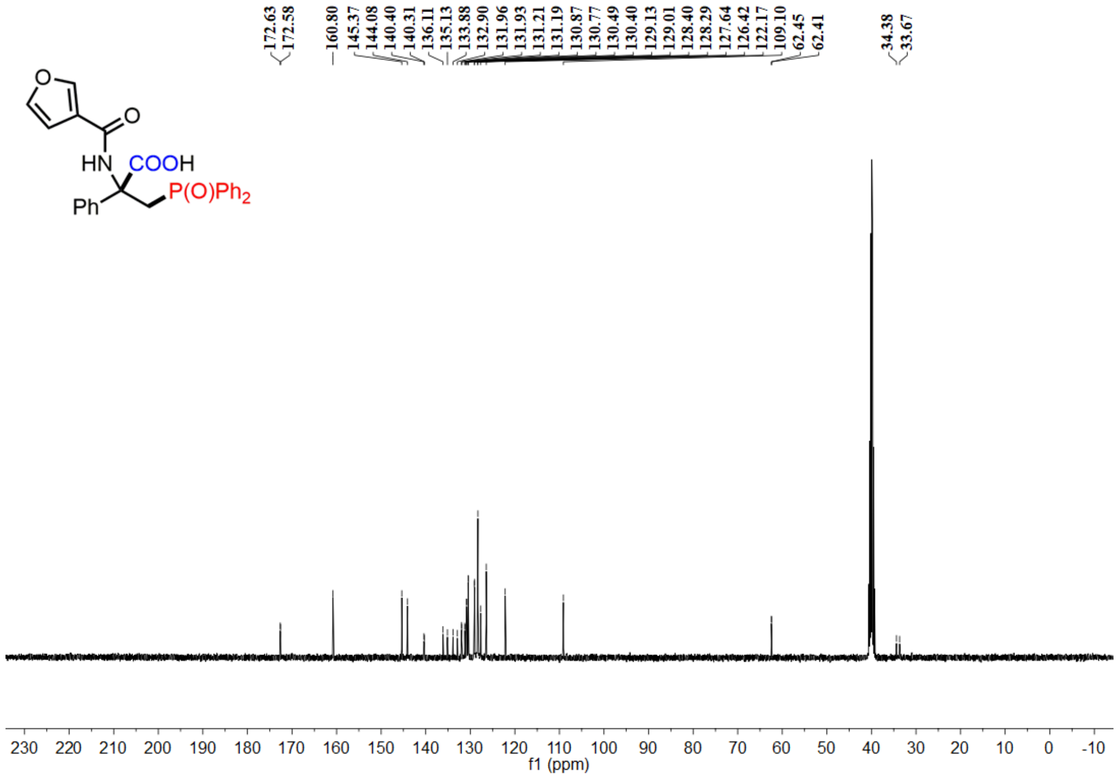

Supplementary Figure 38. ^{13}C NMR spectra of **3ia**

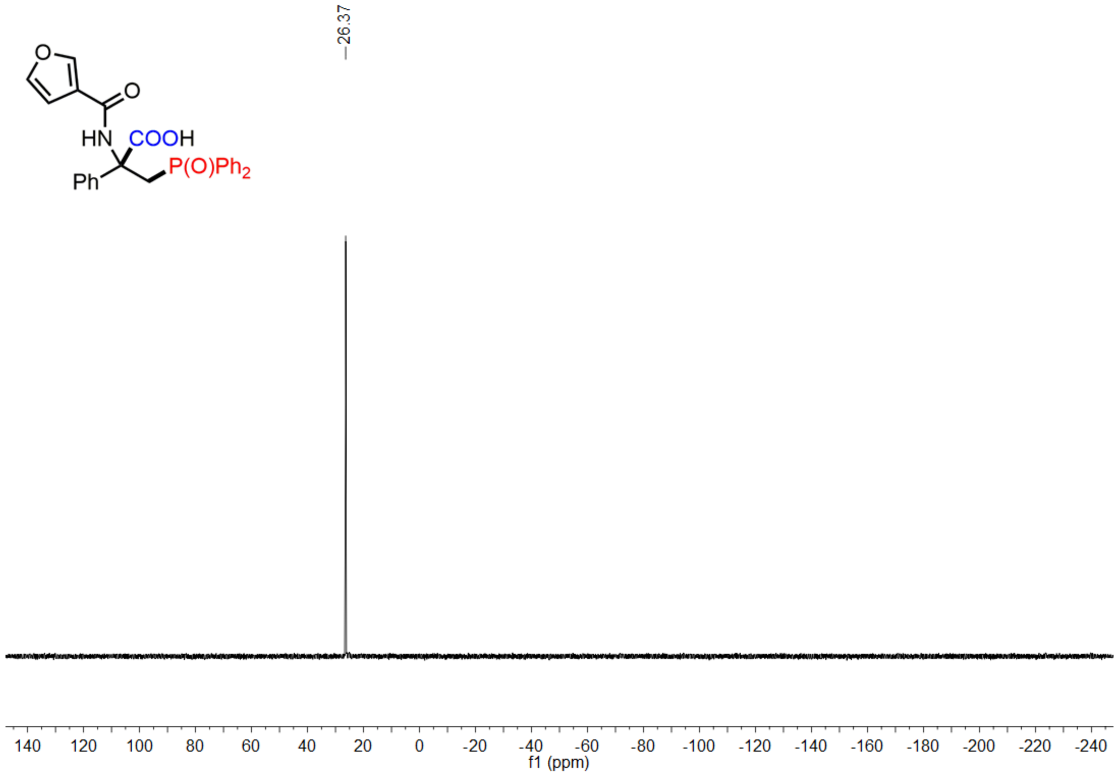

Supplementary Figure 39. ^{31}P NMR spectra of **3ia**

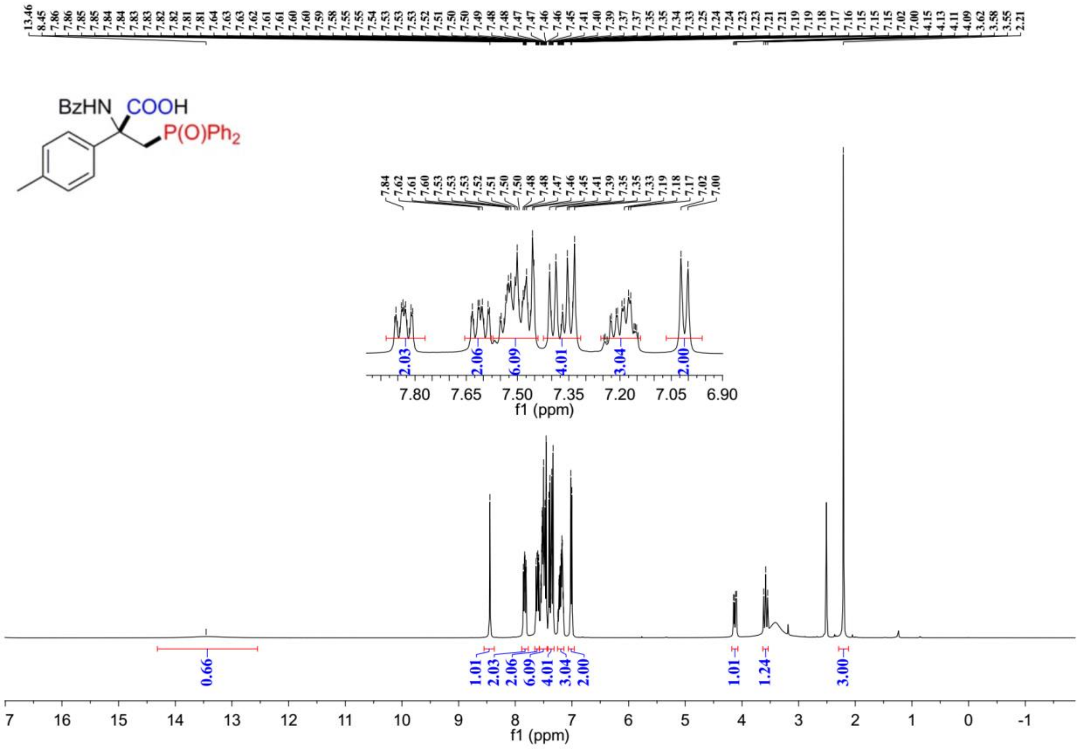

Supplementary Figure 40. ^{1}H NMR spectra of **3ja**

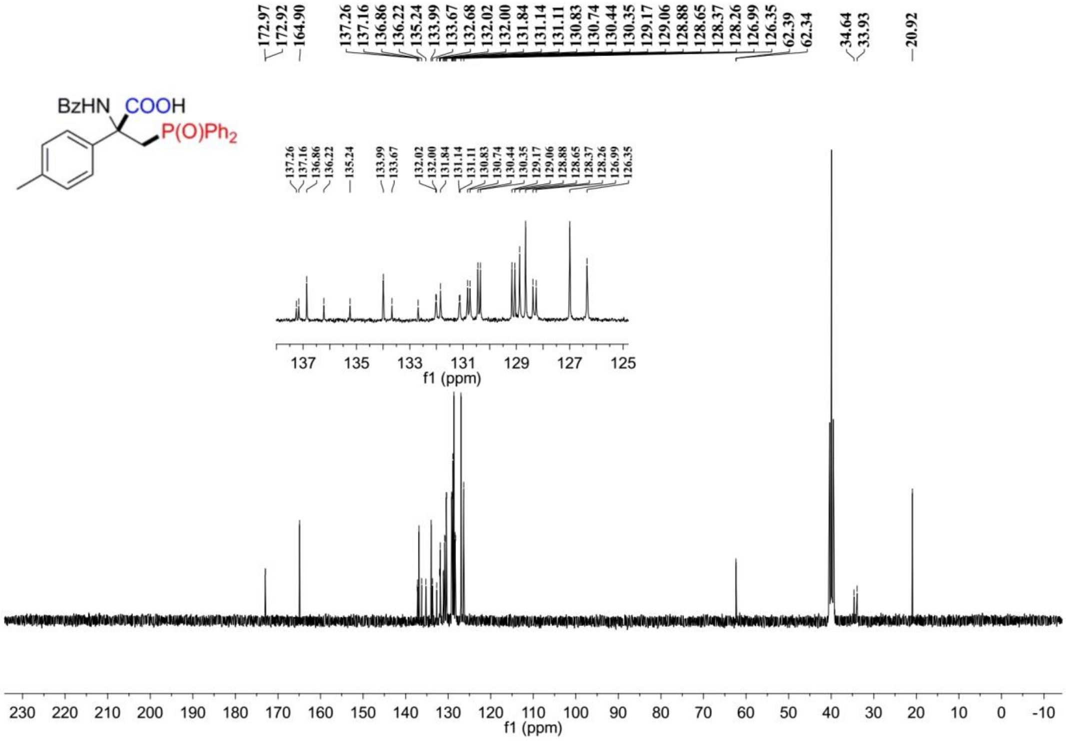

Supplementary Figure 41. ^{13}C NMR spectra of **3ja**

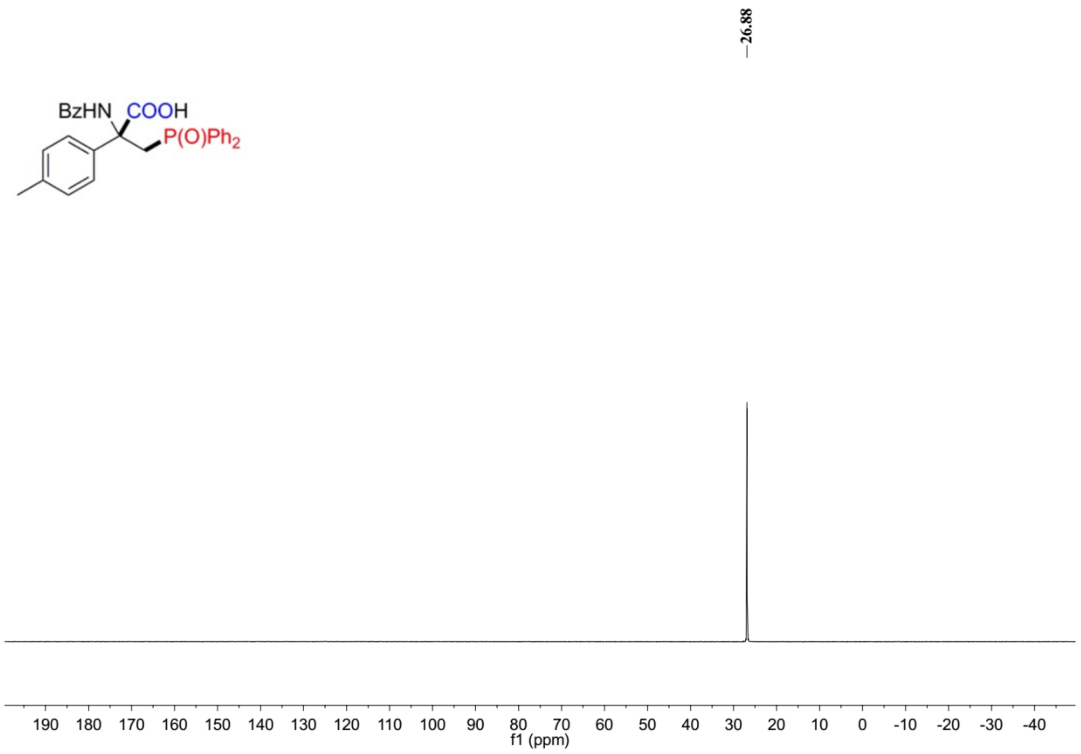

Supplementary Figure 42. ^{31}P NMR spectra of **3ja**

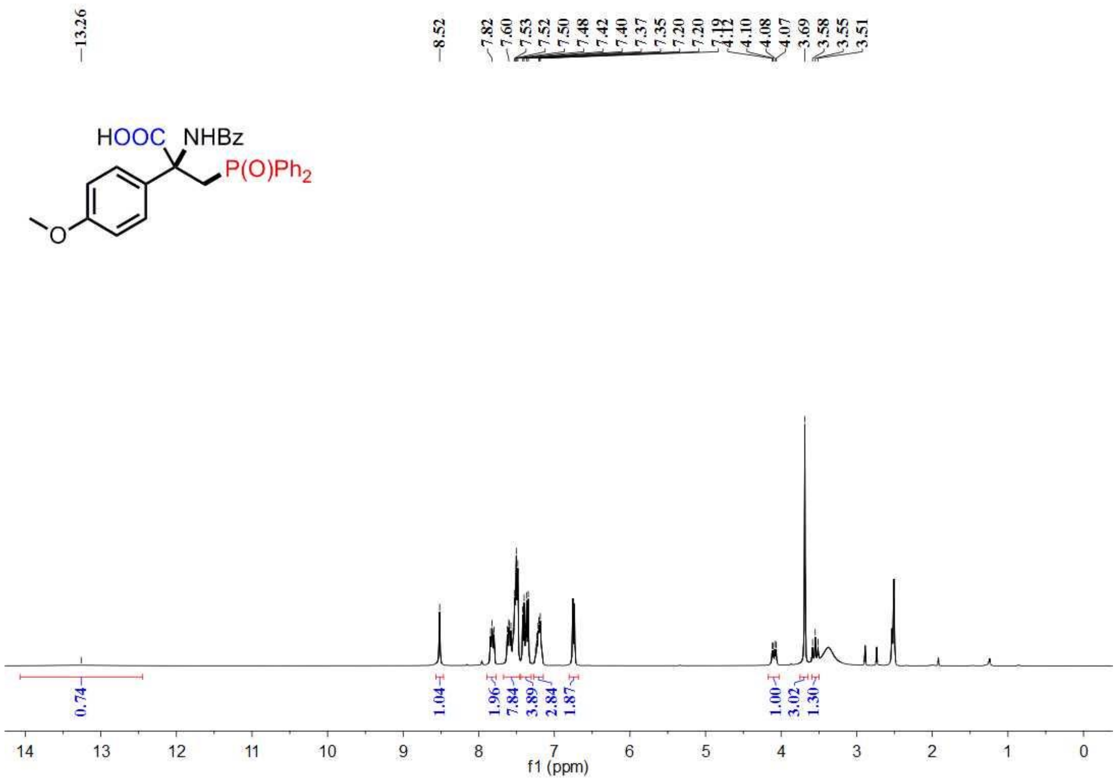

Supplementary Figure 43. ^{1}H NMR spectra of **3ka**

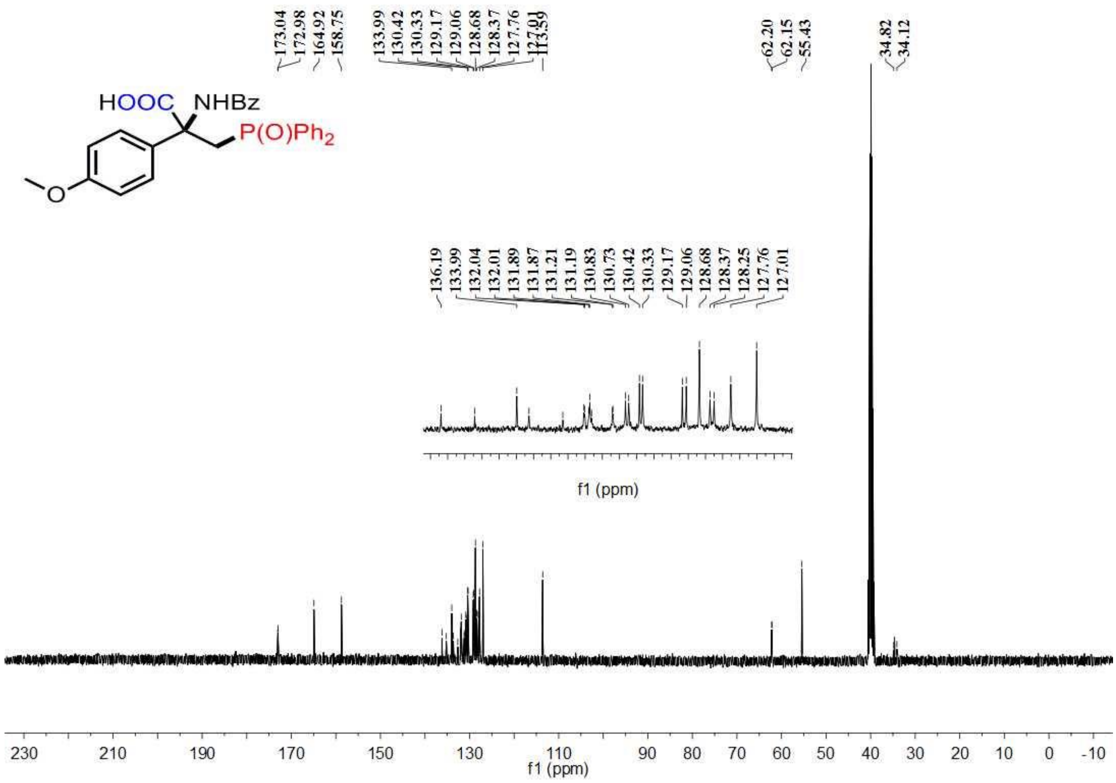

Supplementary Figure 44. ^{13}C NMR spectra of **3ka**

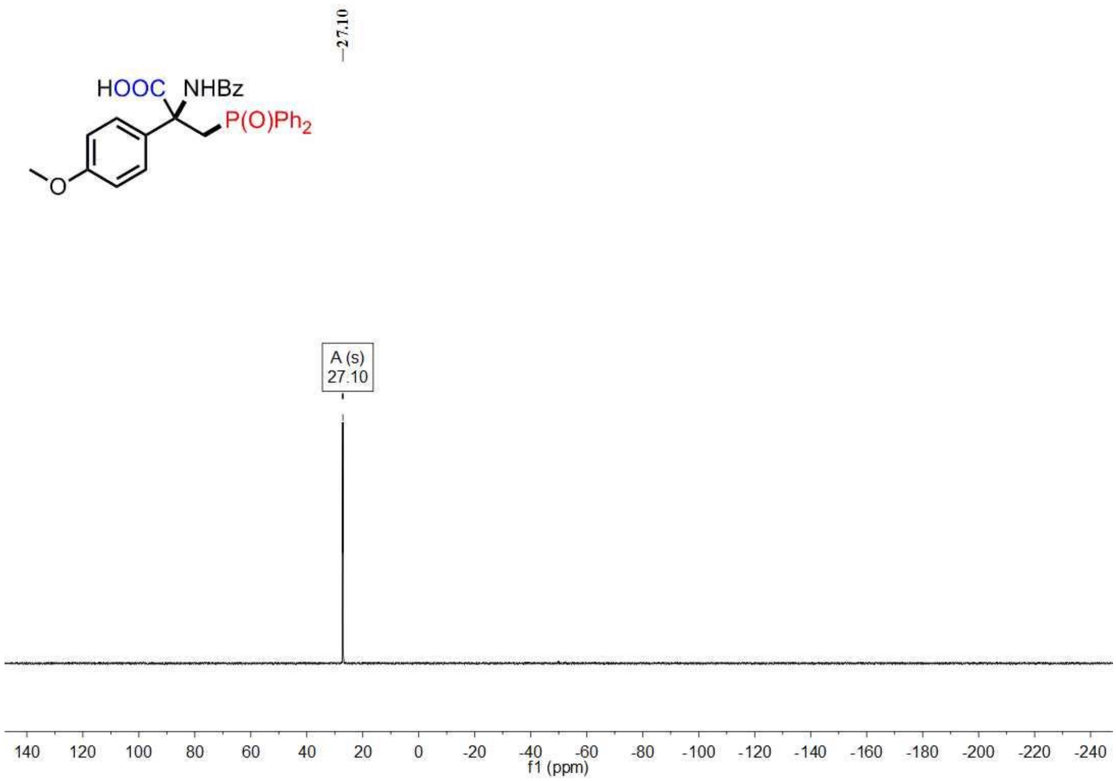

Supplementary Figure 45. ^{31}P NMR spectra of **3ka**

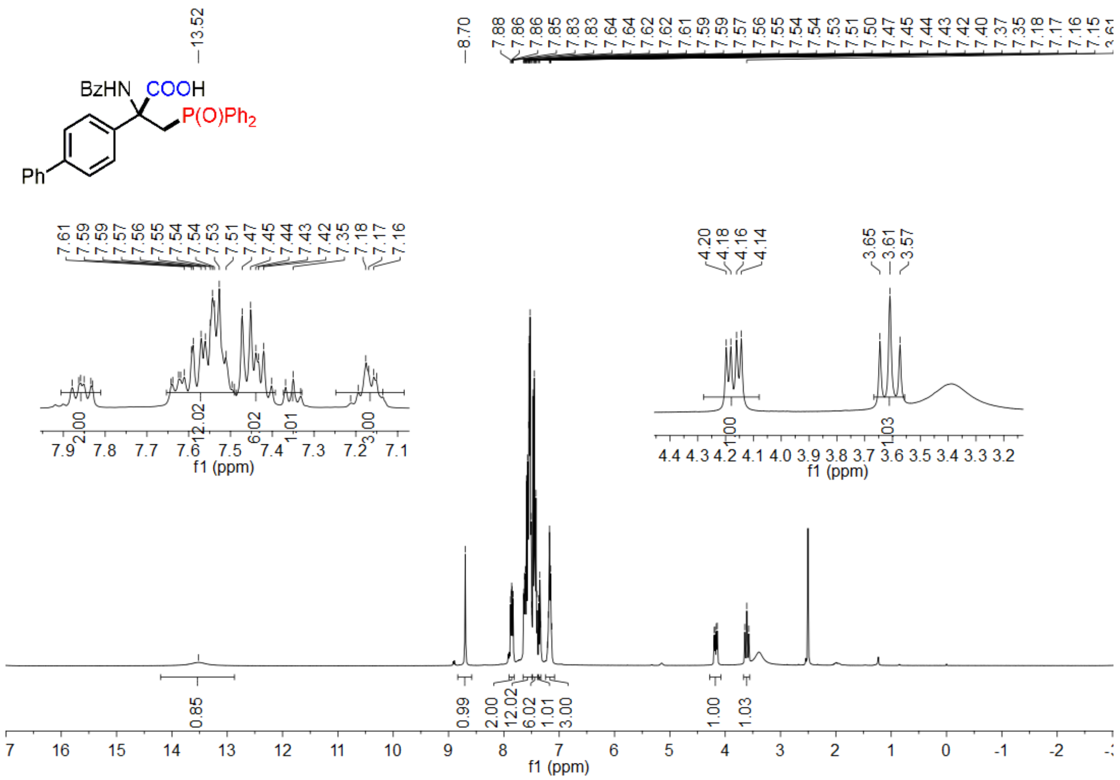

Supplementary Figure 46. ^{1}H NMR spectra of **3la**

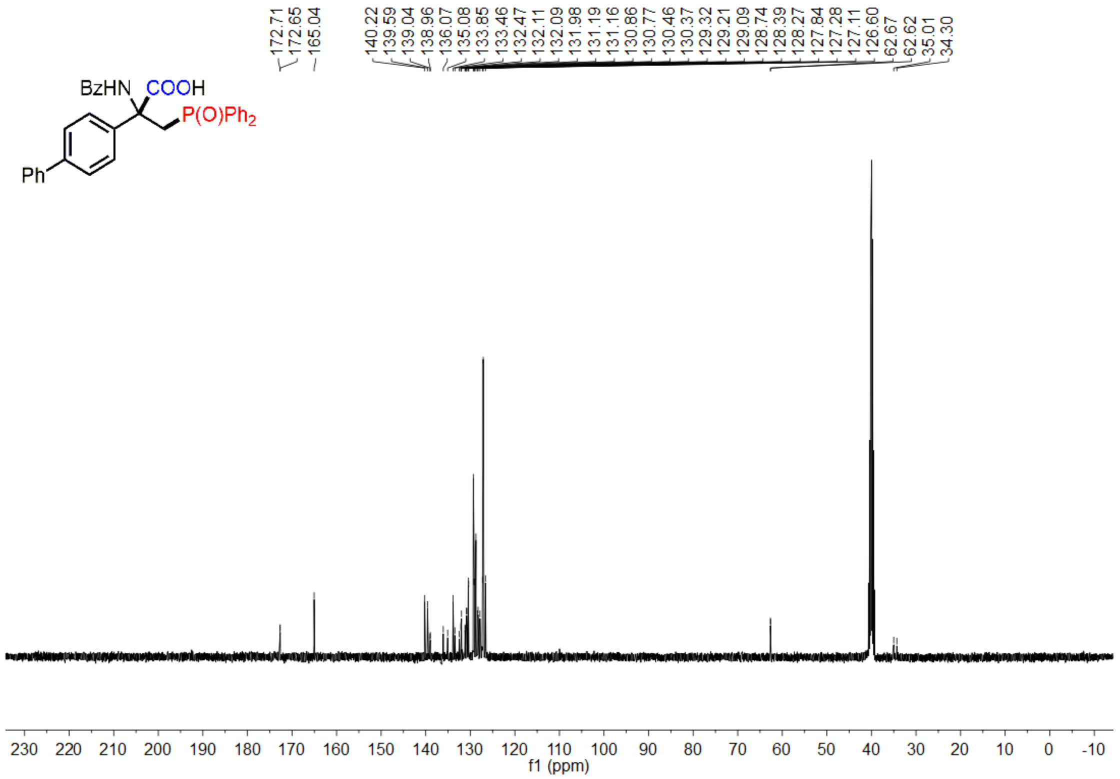

Supplementary Figure 47. ^{13}C NMR spectra of **3la**

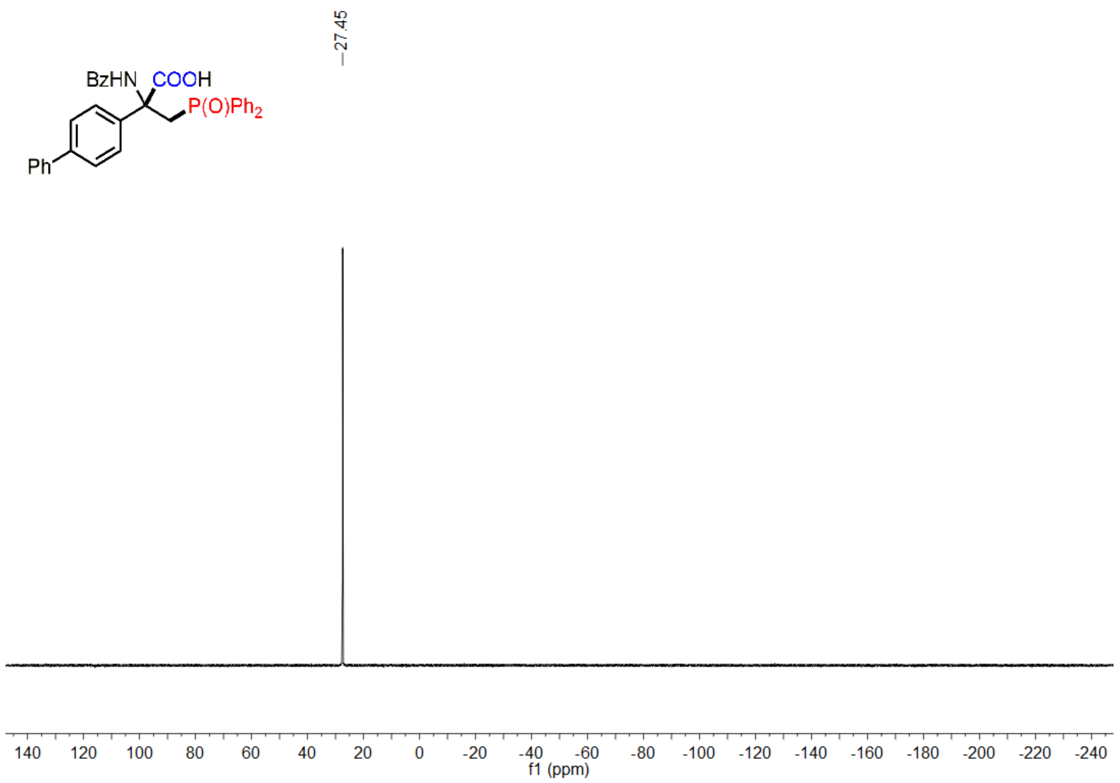

Supplementary Figure 48. ^{31}P NMR spectra of **3la**

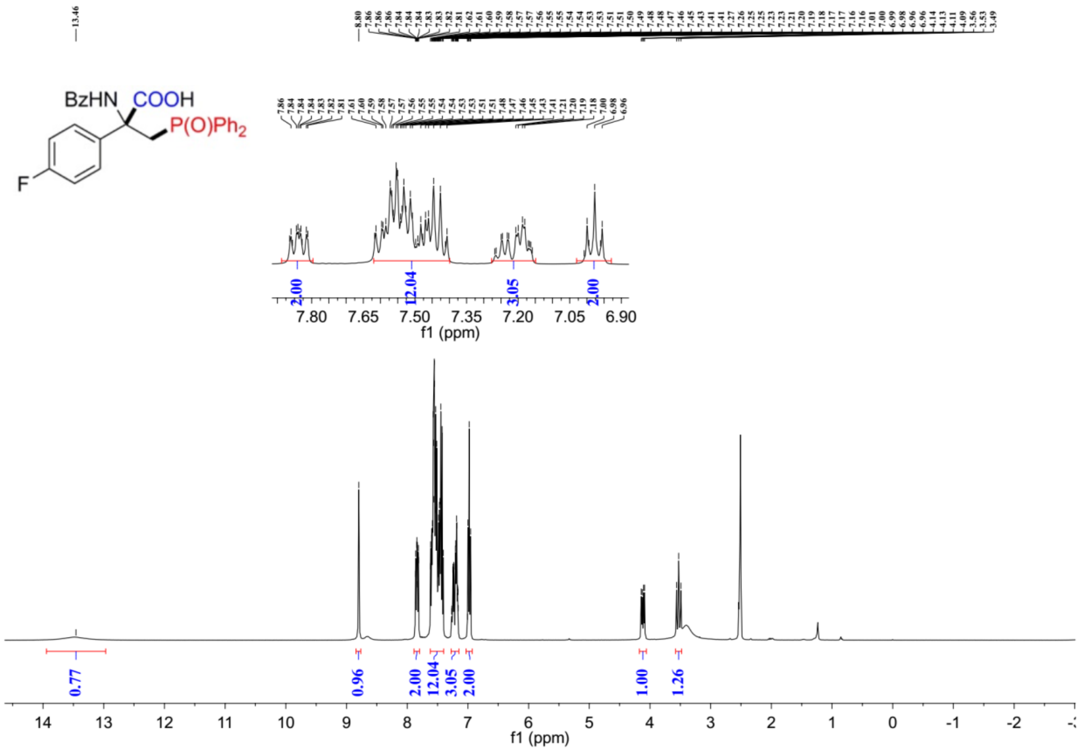

Supplementary Figure 49. ^{1}H NMR spectra of **3ma**

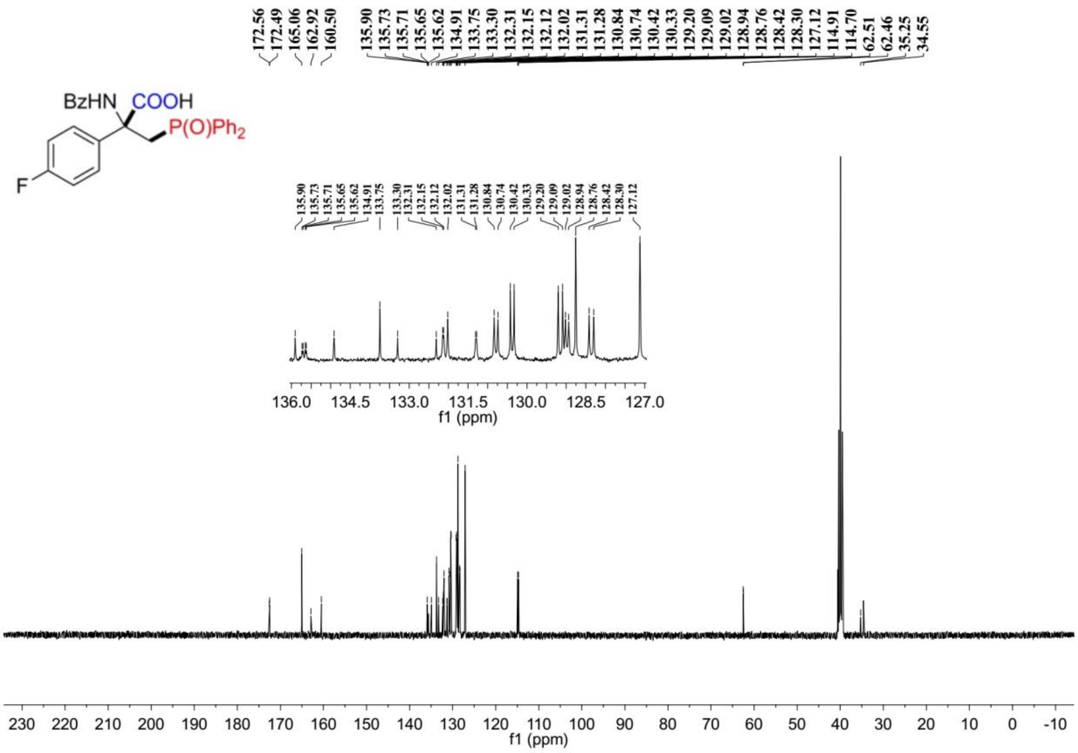

Supplementary Figure 50. ^{13}C NMR spectra of **3ma**

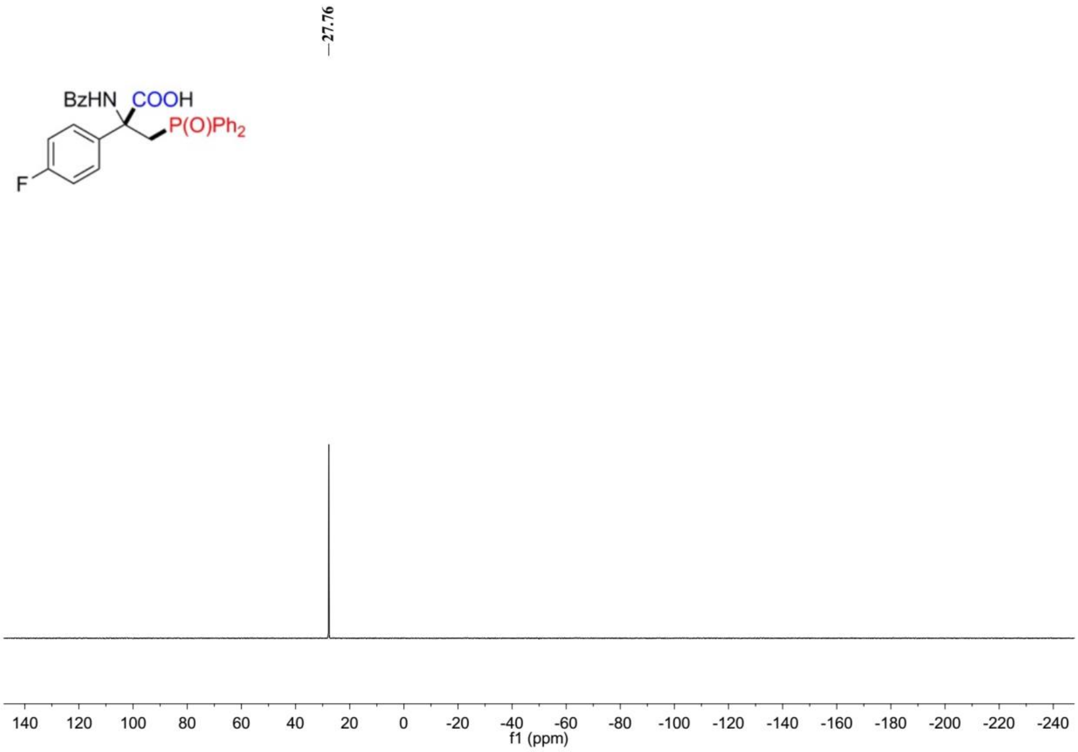

Supplementary Figure 51. ^{31}P NMR spectra of **3ma**

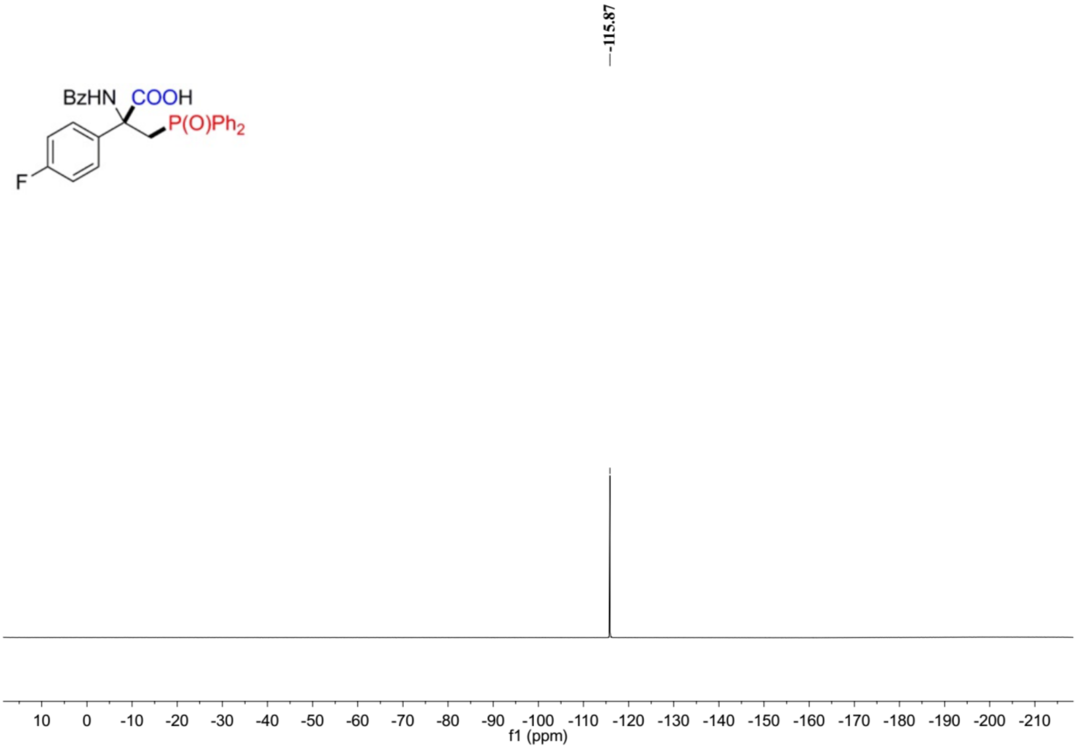

Supplementary Figure 52. ^{19}F NMR spectra of **3ma**

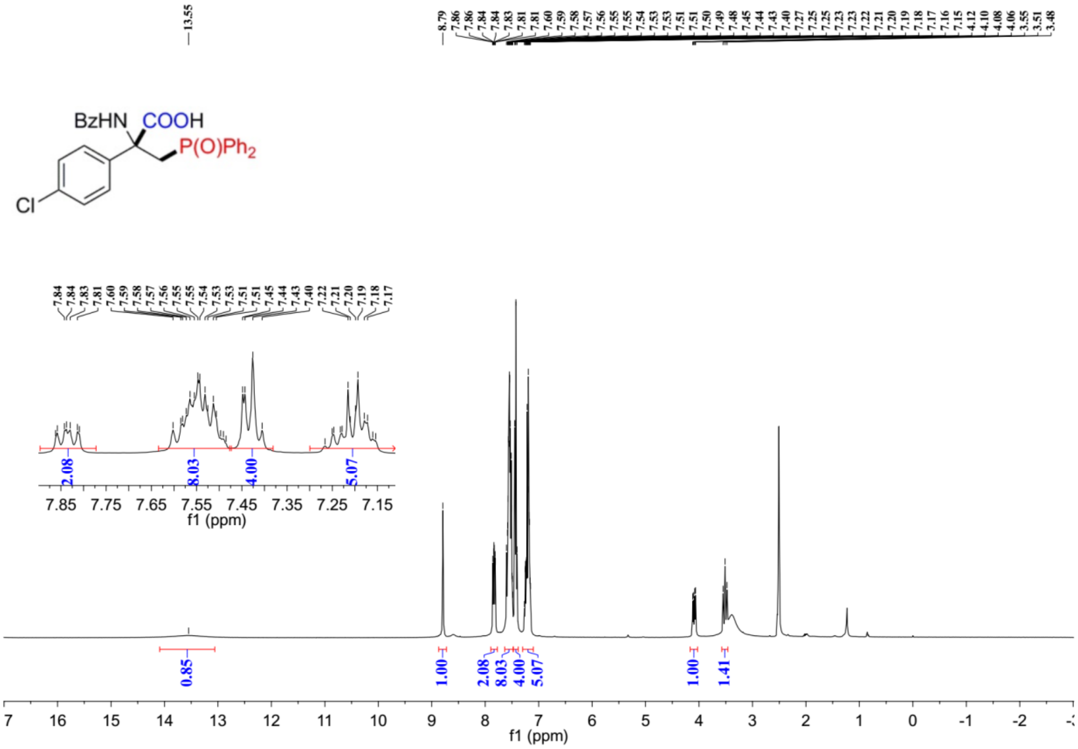

Supplementary Figure 53. ^{1}H NMR spectra of **3na**

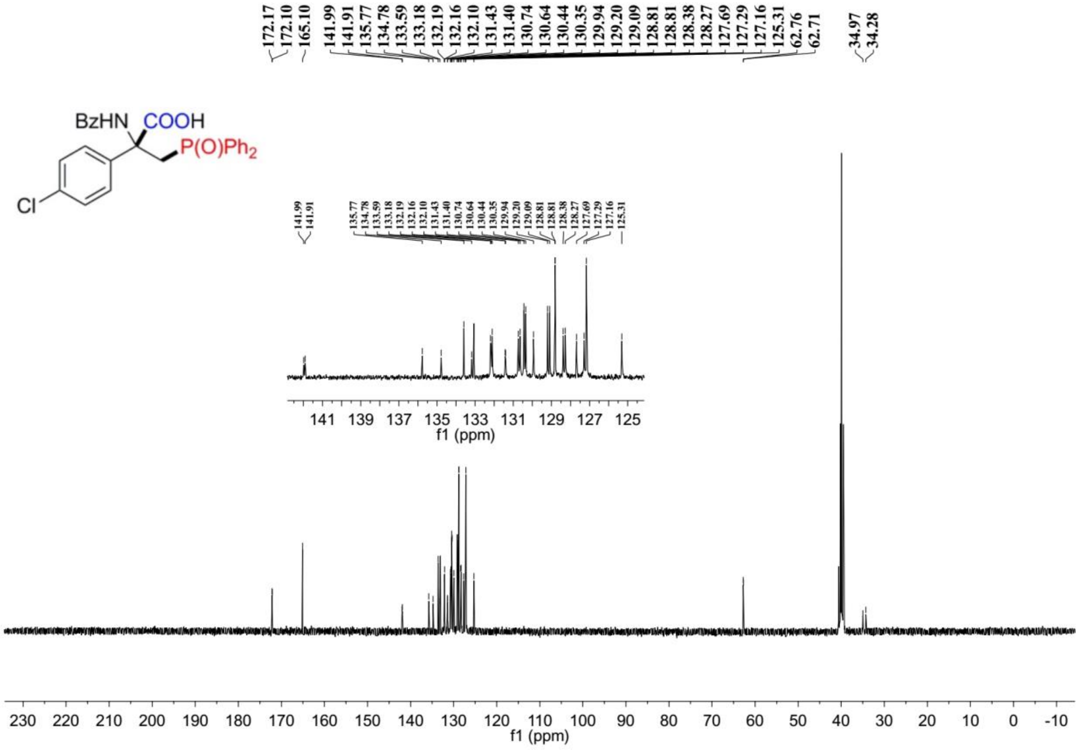

Supplementary Figure 54. ^{13}C NMR spectra of **3na**

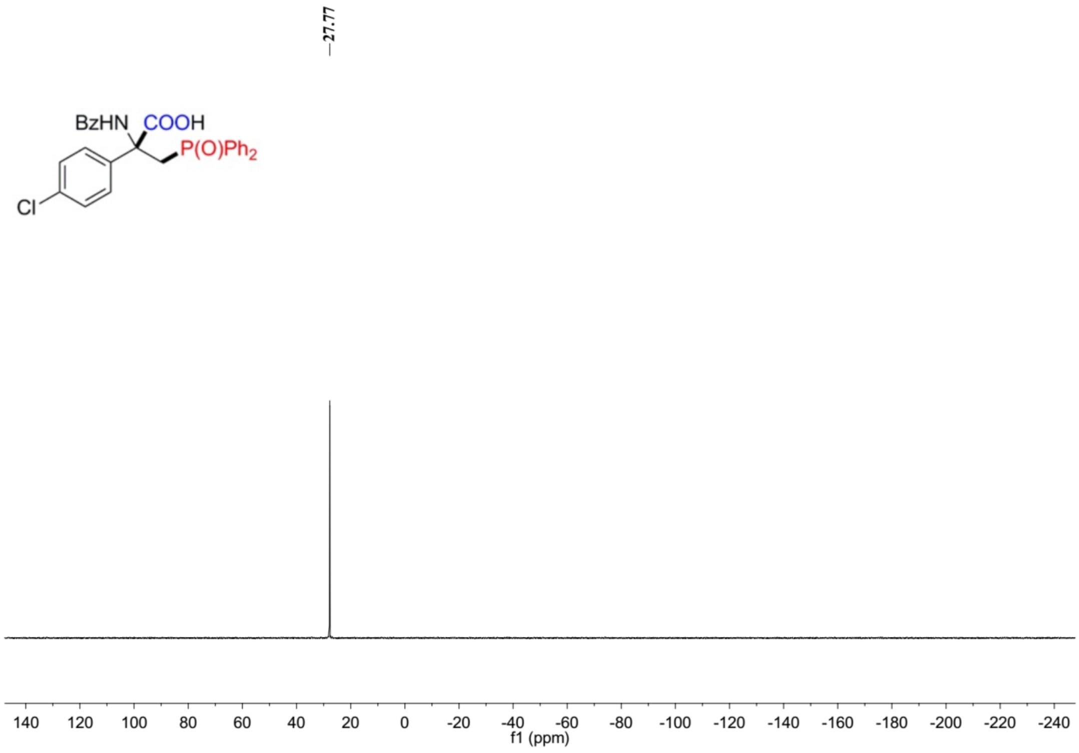

Supplementary Figure 55. ^{31}P NMR spectra of **3na**

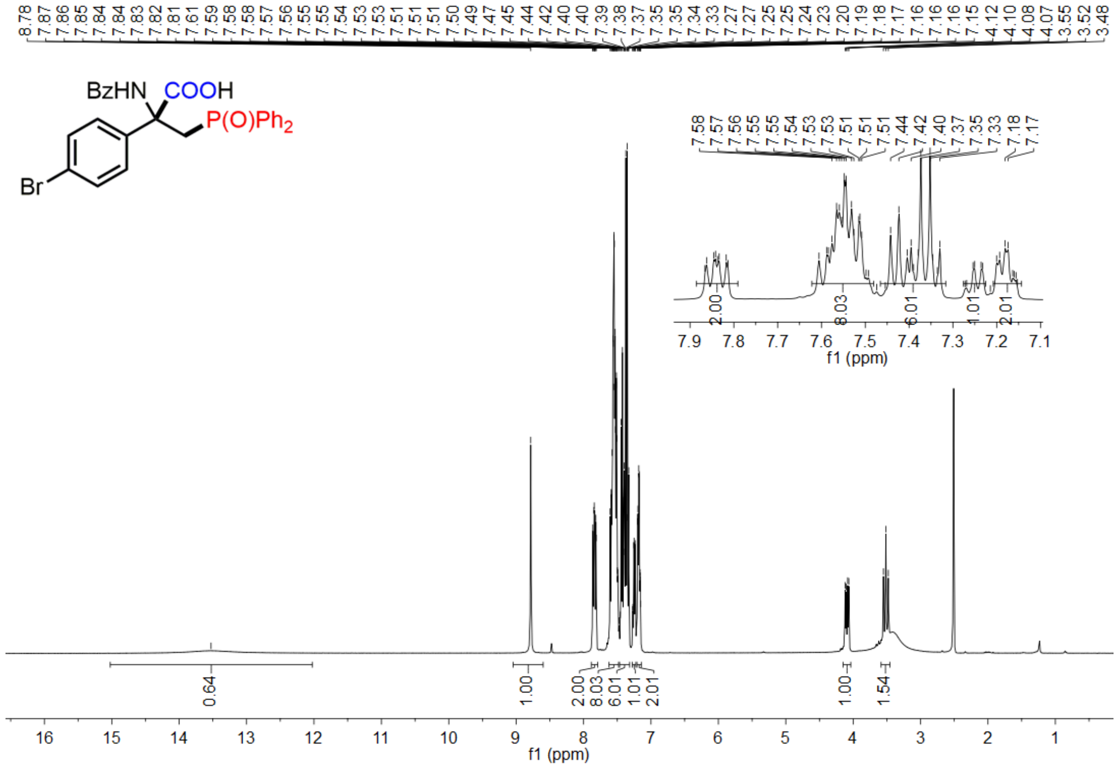

Supplementary Figure 56. ^{1}H NMR spectra of **3oa**

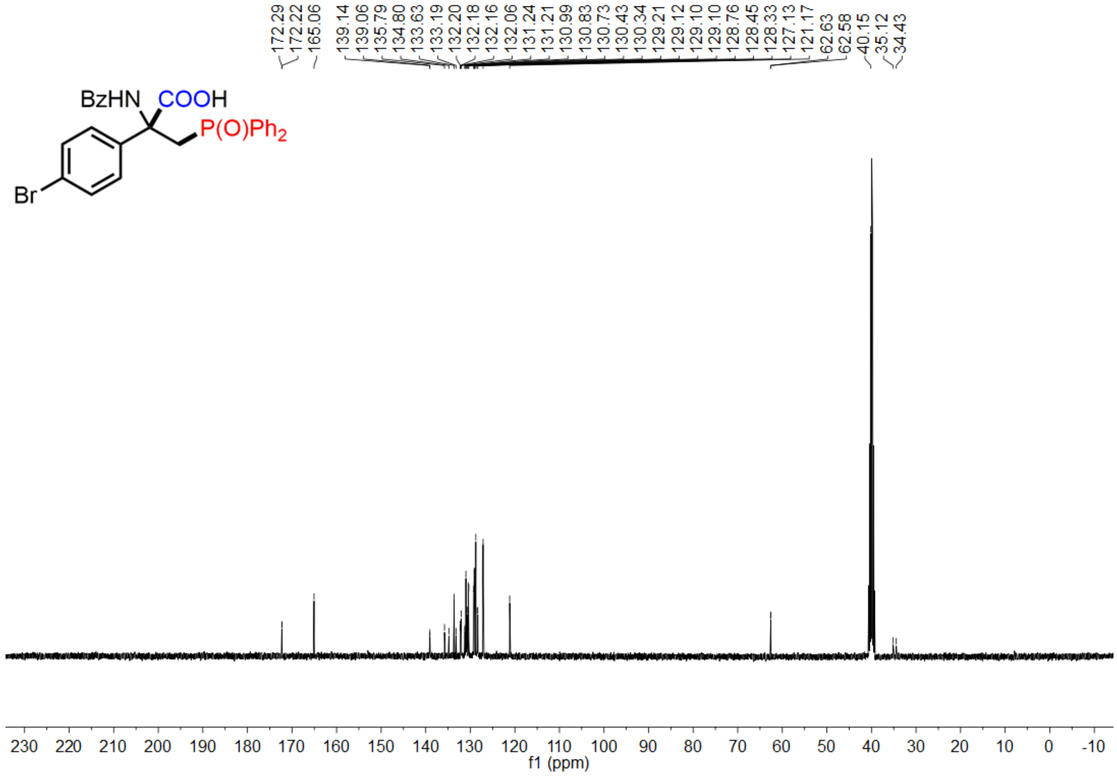

Supplementary Figure 57. ^{13}C NMR spectra of **3oa**

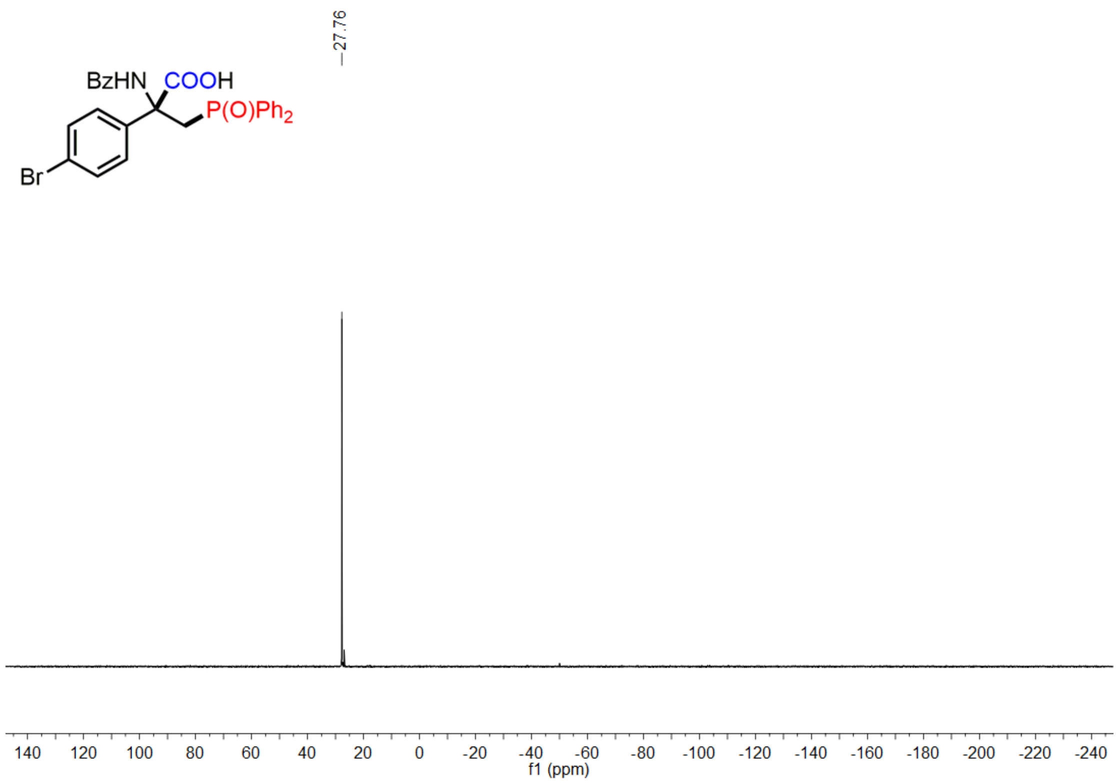

Supplementary Figure 58. ^{31}P NMR spectra of **3oa**

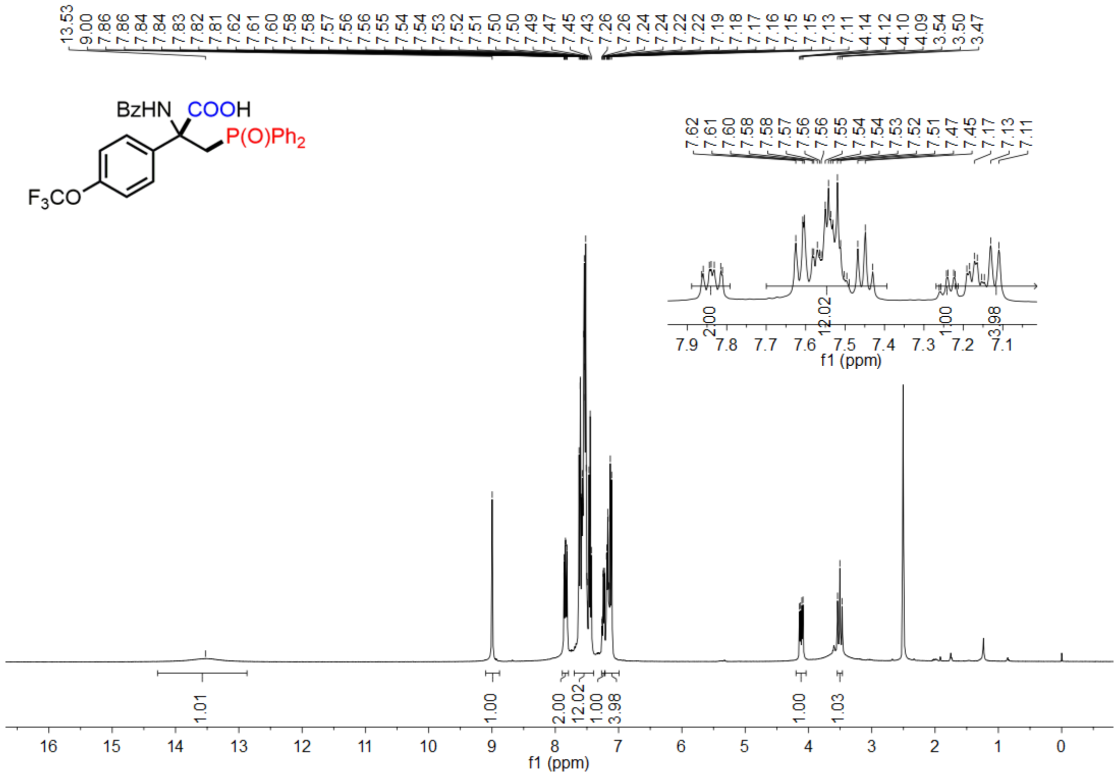

Supplementary Figure 59. ^{1}H NMR spectra of **3pa**

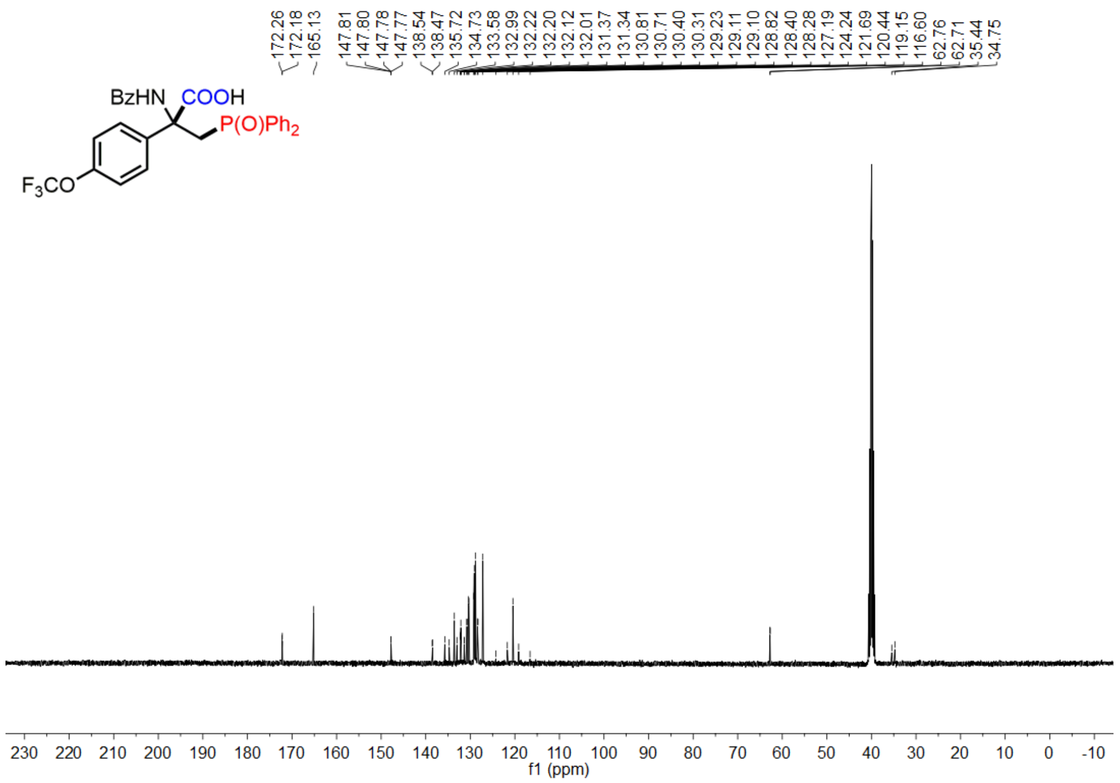

Supplementary Figure 60. ^{13}C NMR spectra of **3pa**

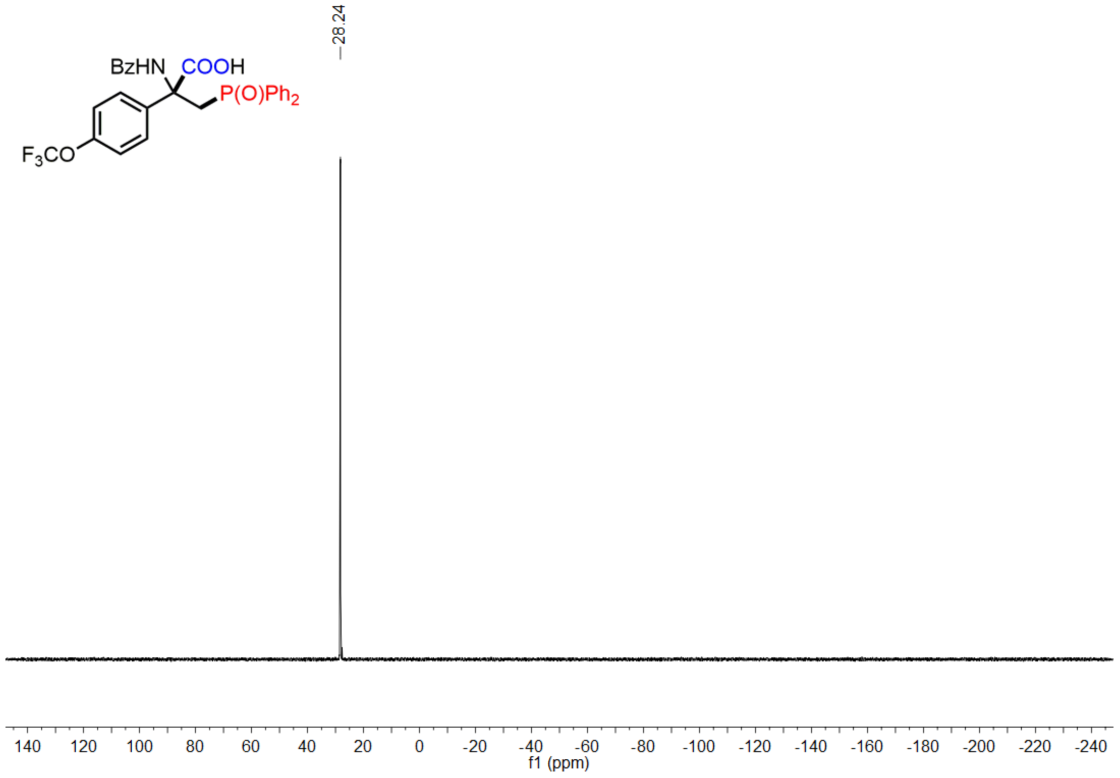

Supplementary Figure 61. ^{31}P NMR spectra of **3pa**

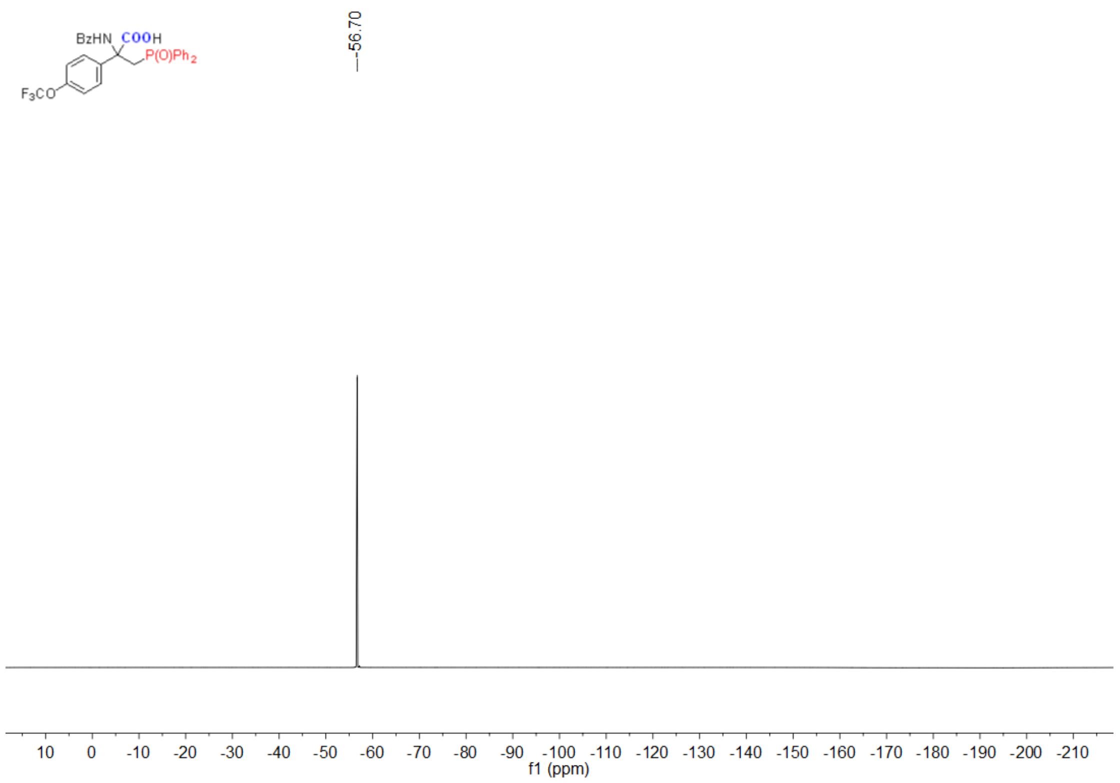

Supplementary Figure 62. ^{19}F NMR spectra of **3pa**

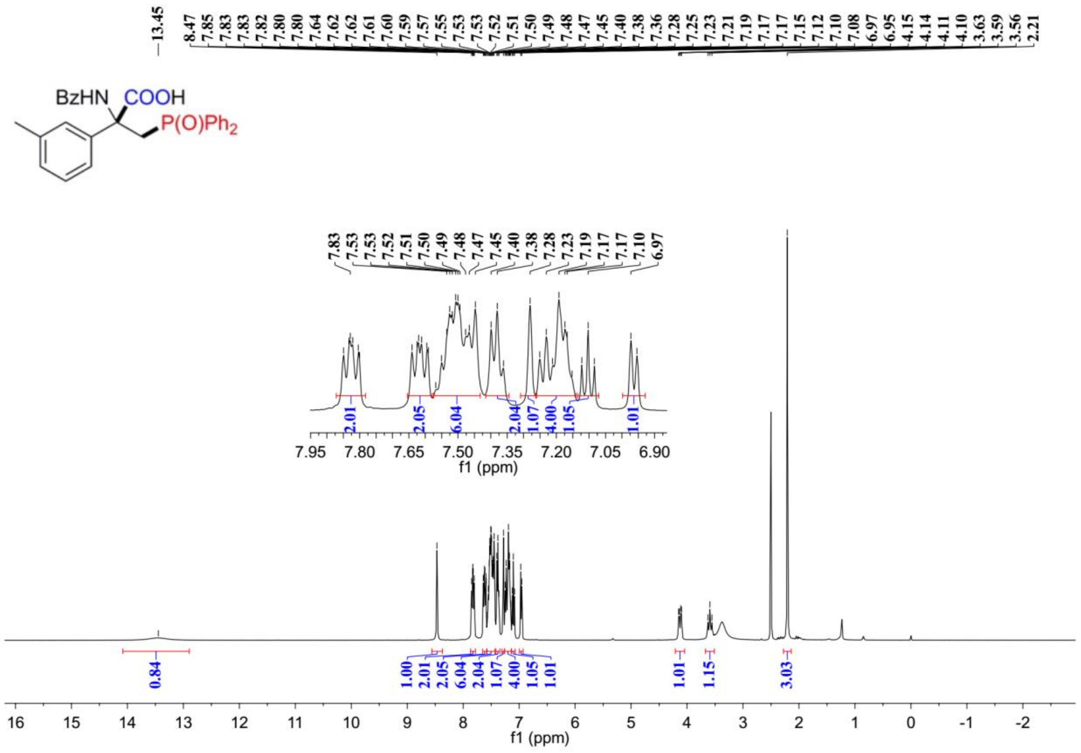

Supplementary Figure 63. ^{1}H NMR spectra of **3qa**

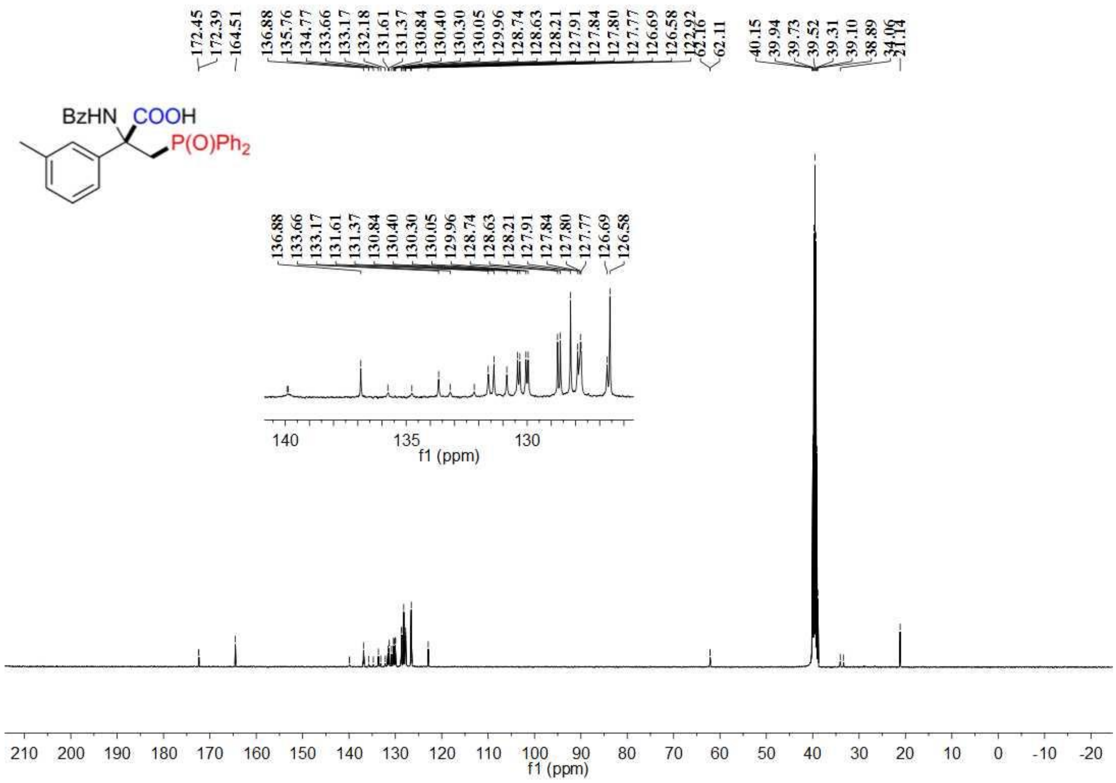

Supplementary Figure 64. ^{13}C NMR spectra of **3qa**

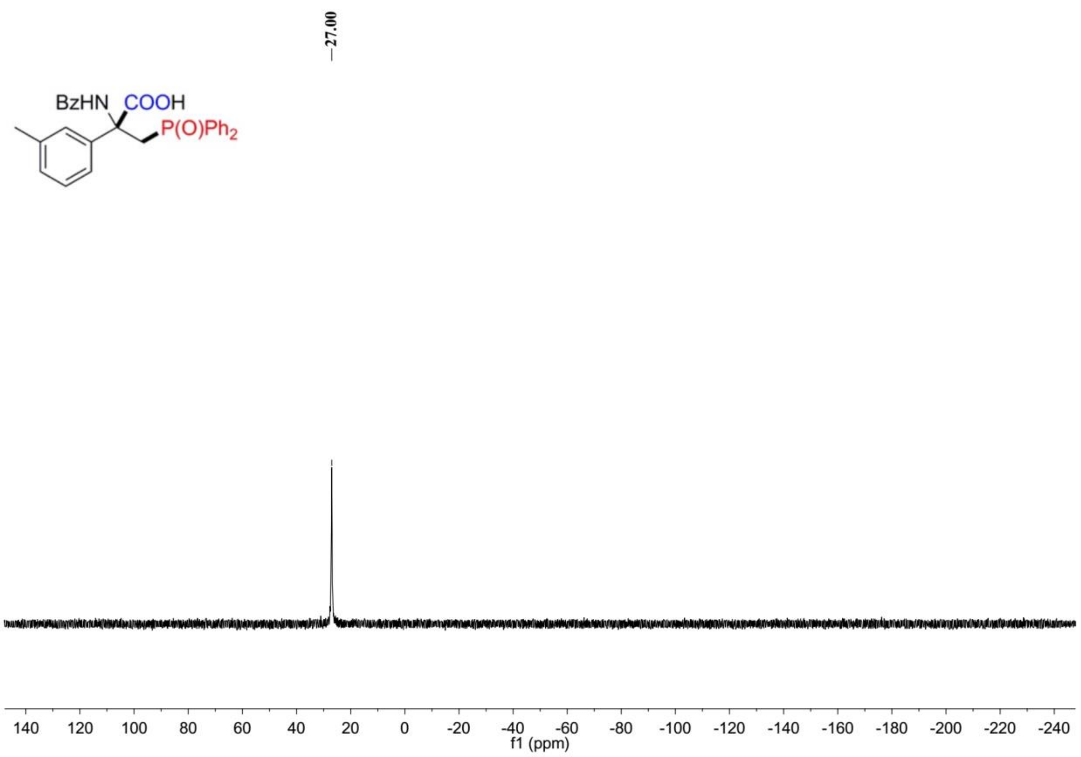

Supplementary Figure 65. ^{31}P NMR spectra of **3qa**

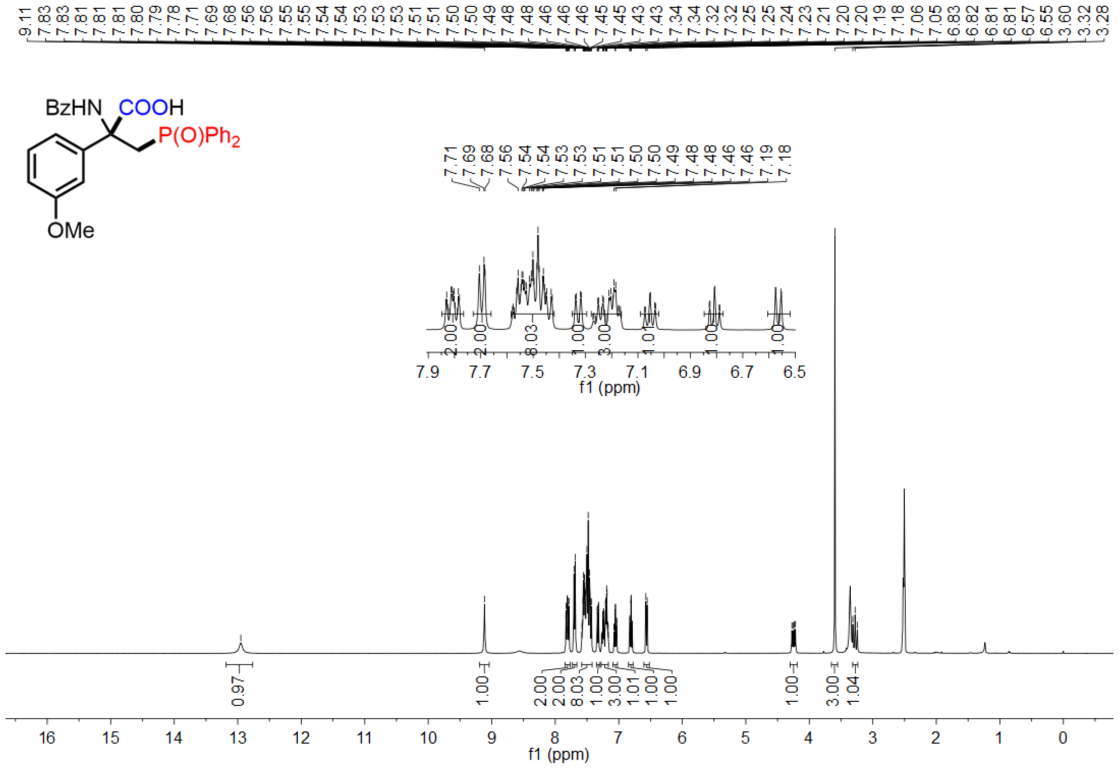

Supplementary Figure 66. ^{1}H NMR spectra of **3ra**

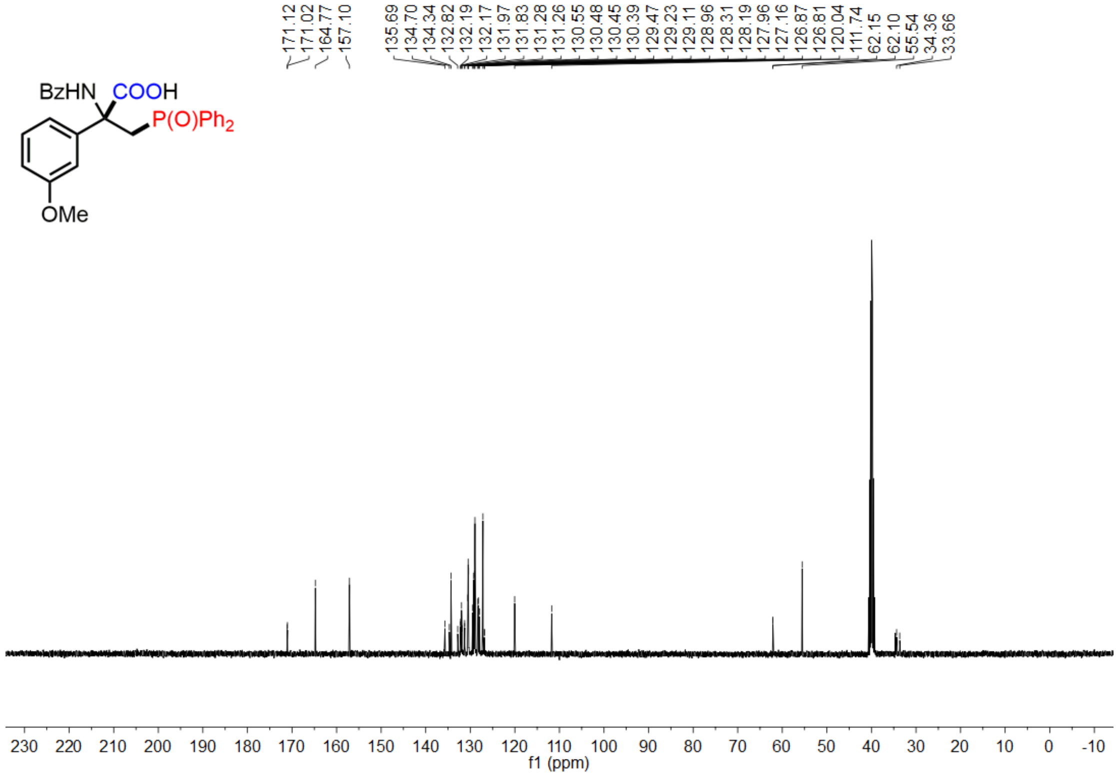

Supplementary Figure 67. ^{13}C NMR spectra of **3ra**

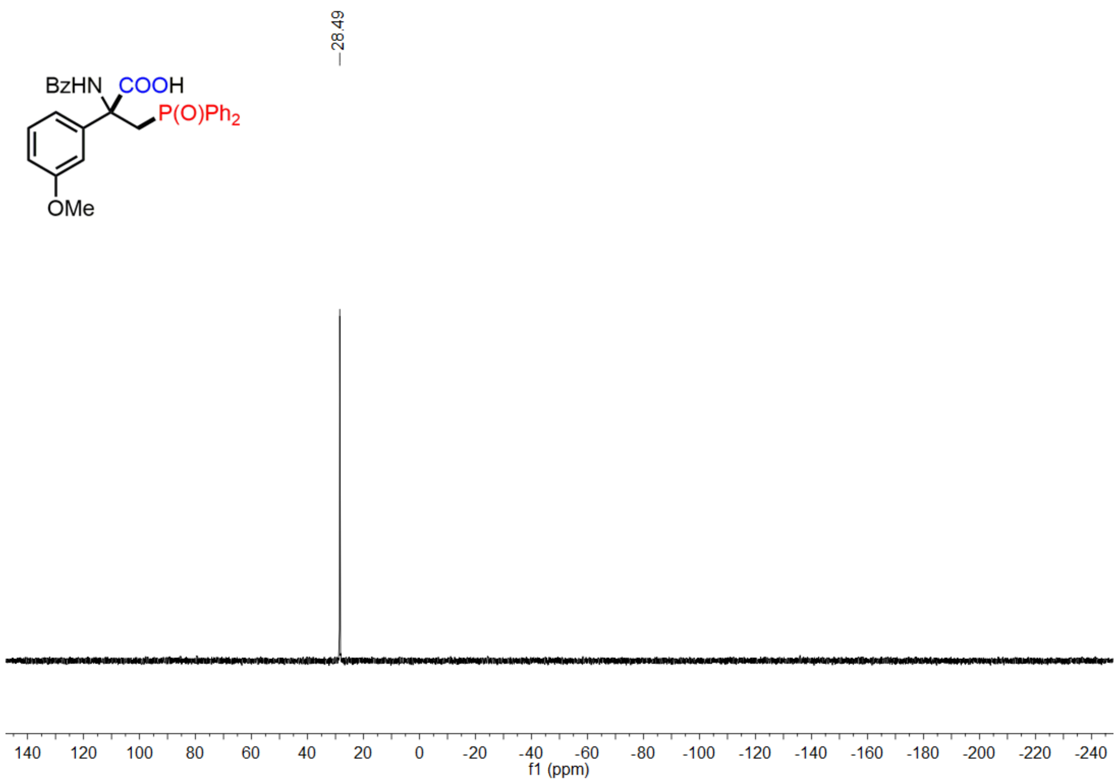

Supplementary Figure 68. ^{31}P NMR spectra of **3ra**

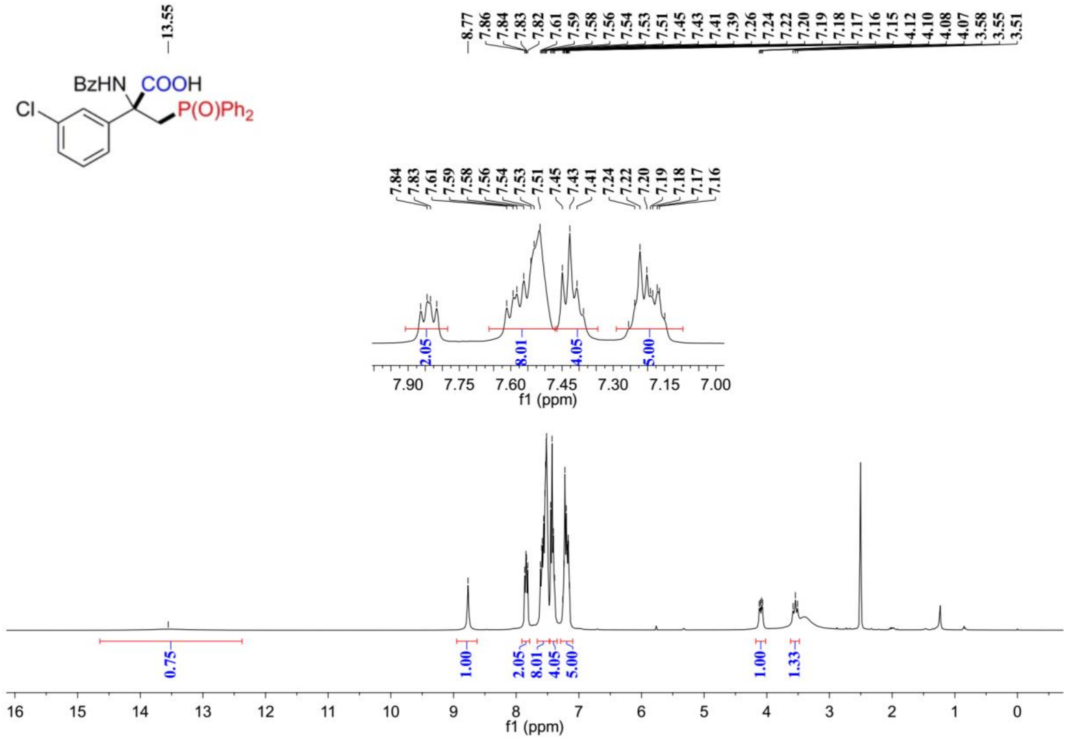

Supplementary Figure 69. ^{1}H NMR spectra of **3sa**

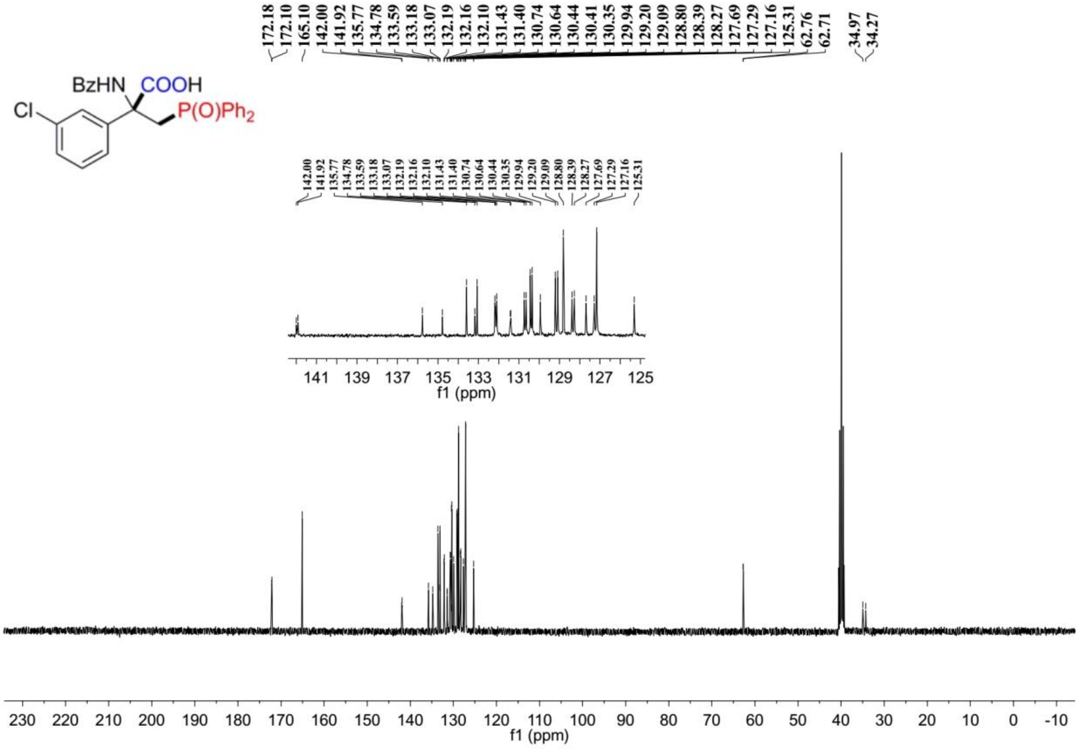

Supplementary Figure 70. ^{13}C NMR spectra of **3sa**

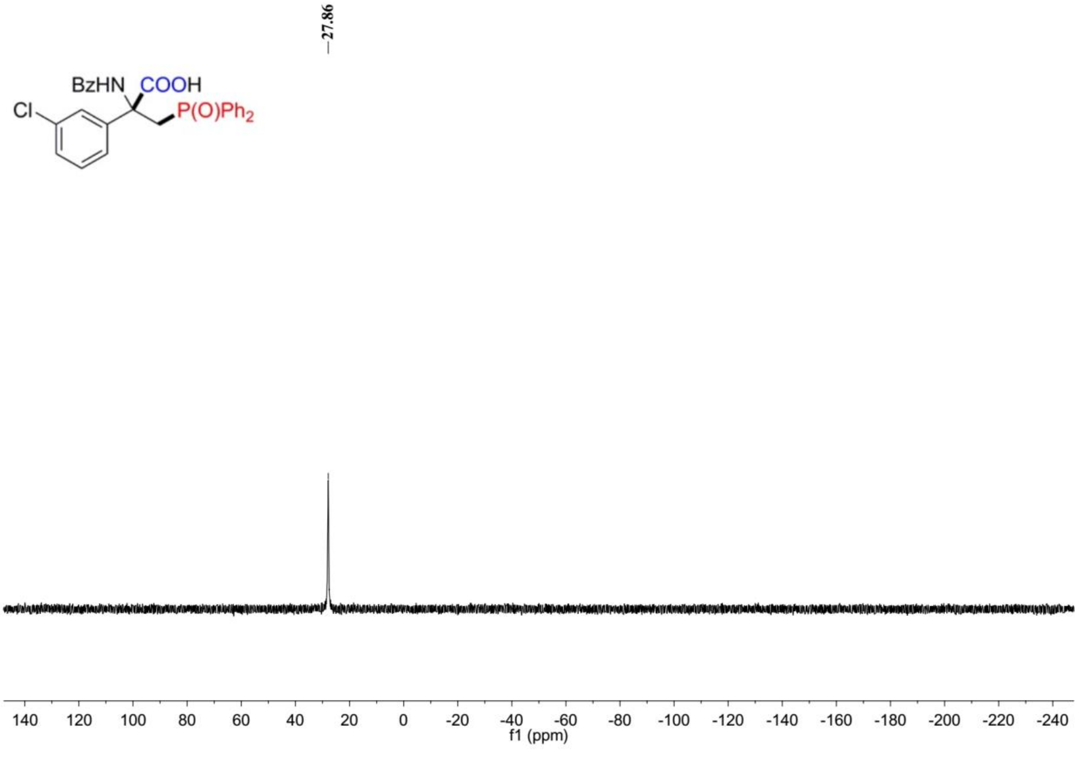

Supplementary Figure 71. ^{31}P NMR spectra of **3sa**

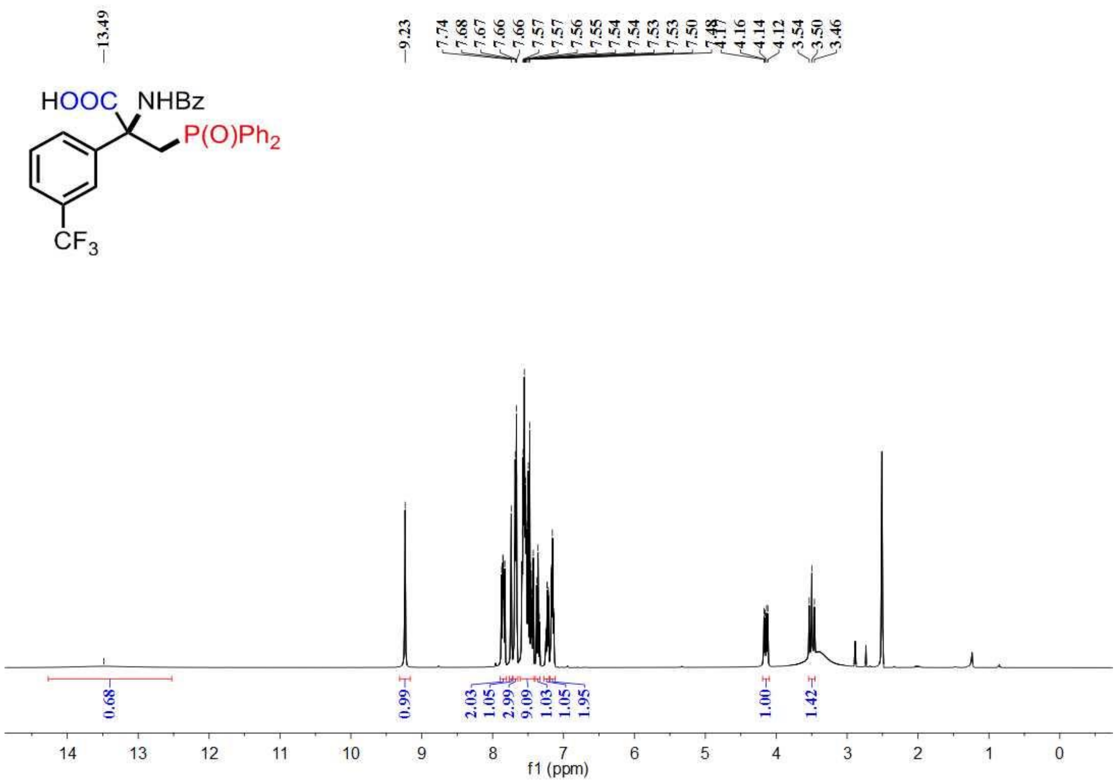

Supplementary Figure 72. ^{1}H NMR spectra of **3ta**

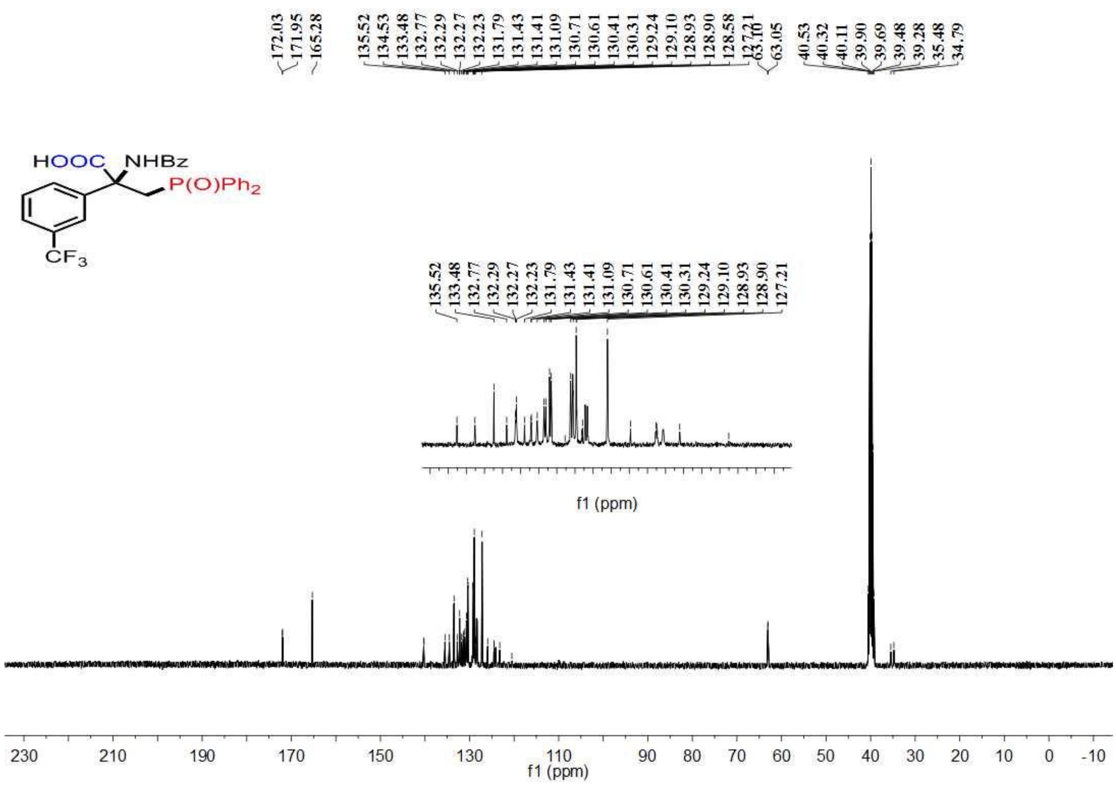

Supplementary Figure 73. ^{13}C NMR spectra of **3ta**

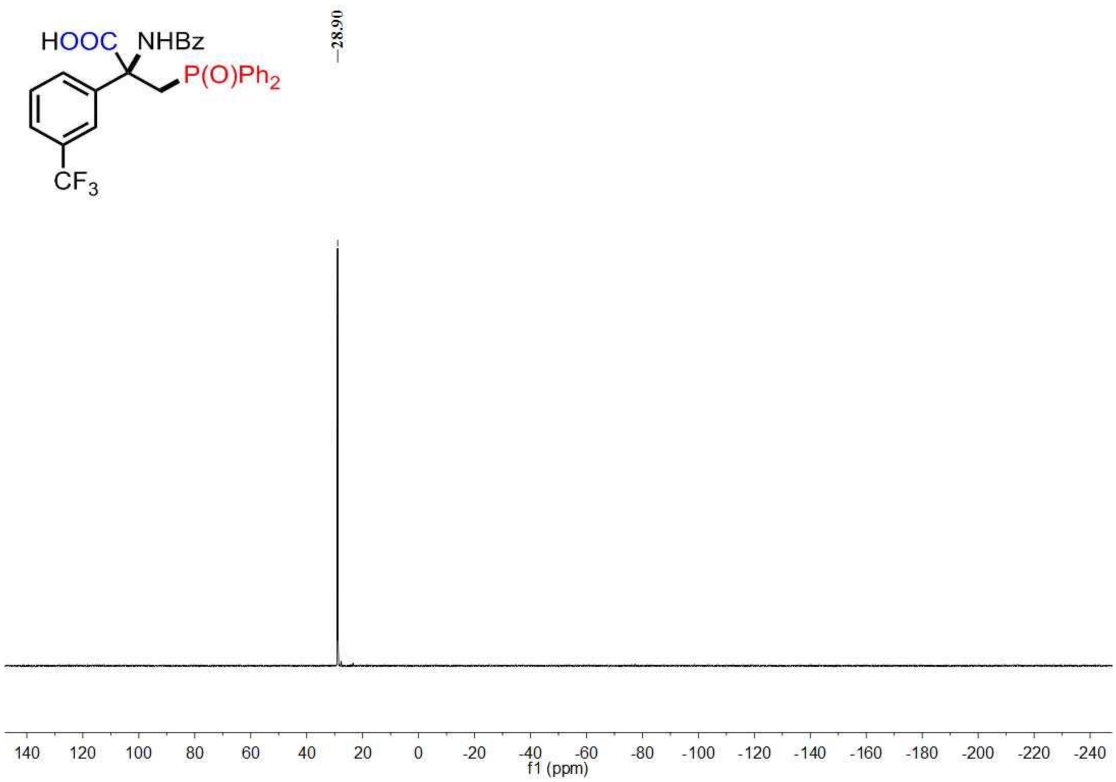

Supplementary Figure 74. ^{31}P NMR spectra of **3ta**

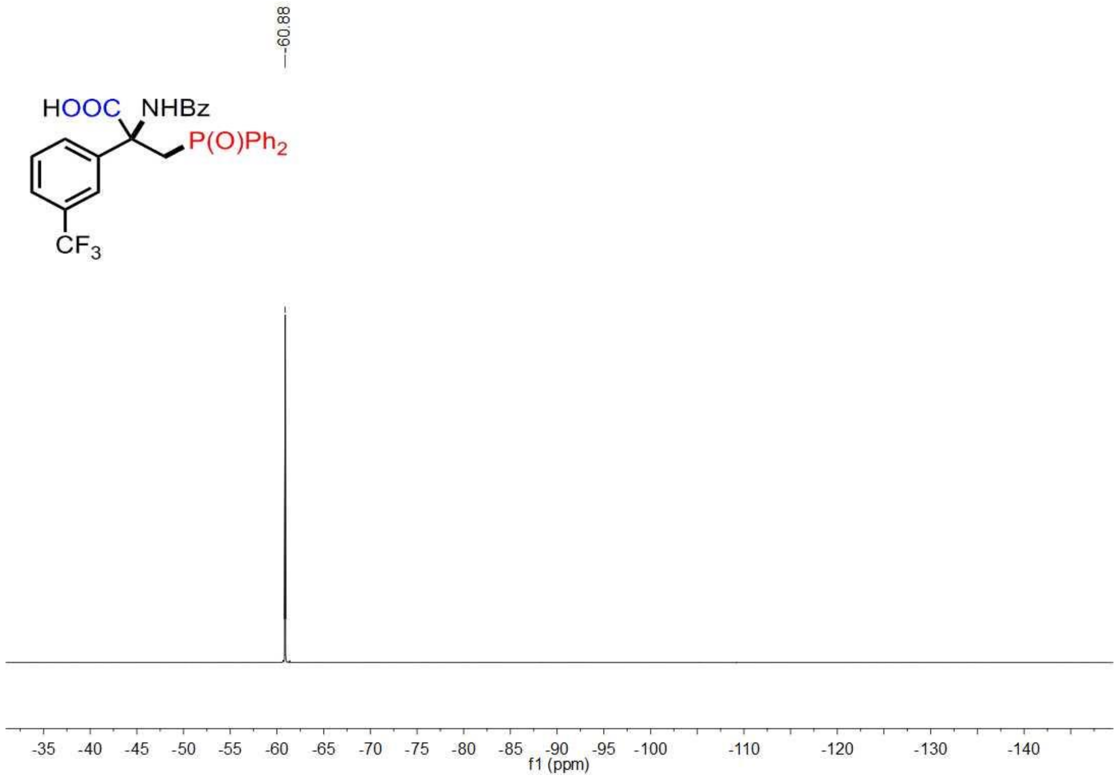

Supplementary Figure 75. ^{19}F NMR spectra of **3ta**

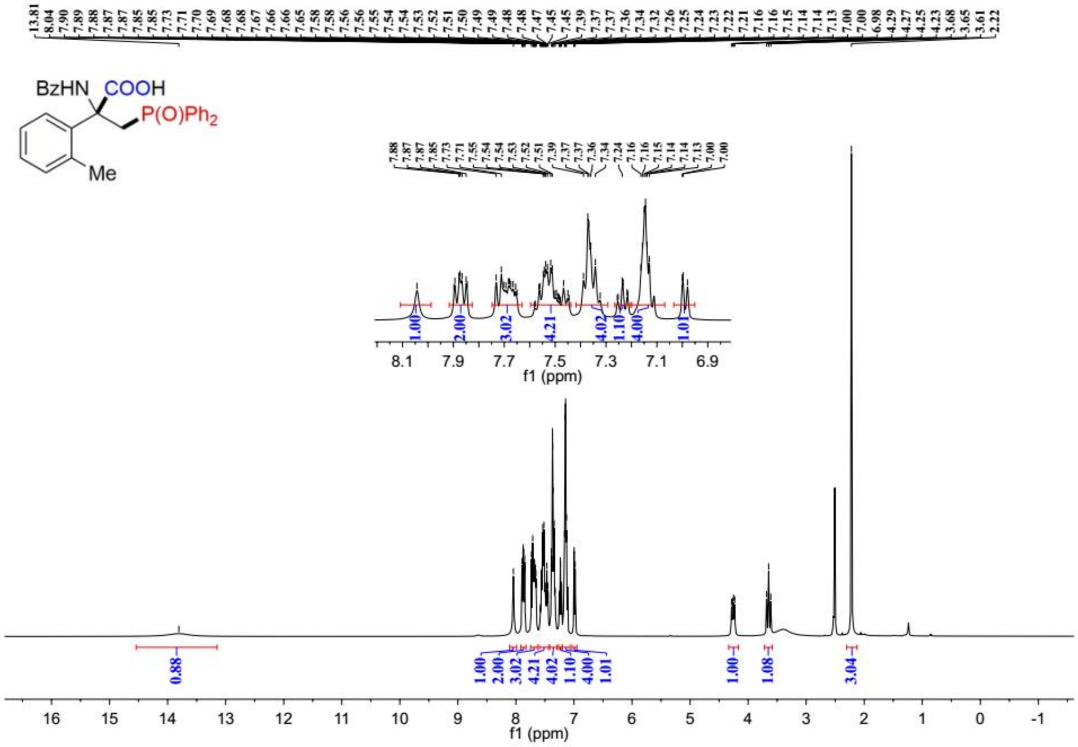

Supplementary Figure 76. ^{1}H NMR spectra of **3ua**

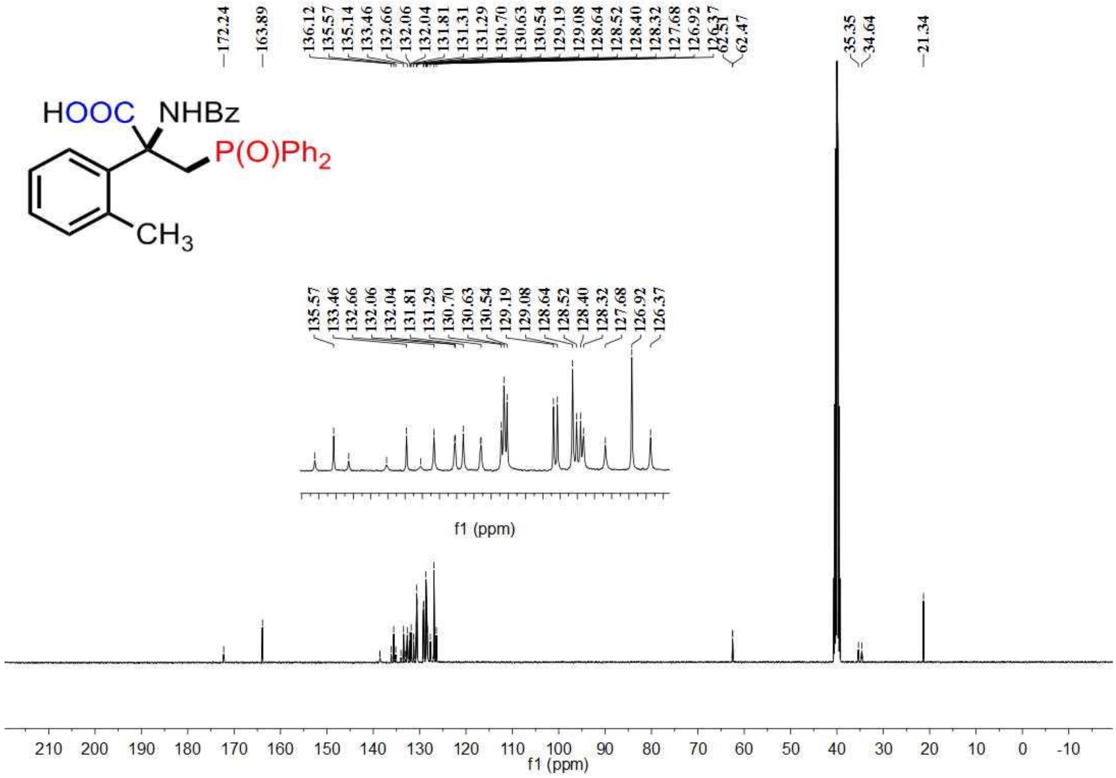

Supplementary Figure 77. ^{13}C NMR spectra of **3ua**

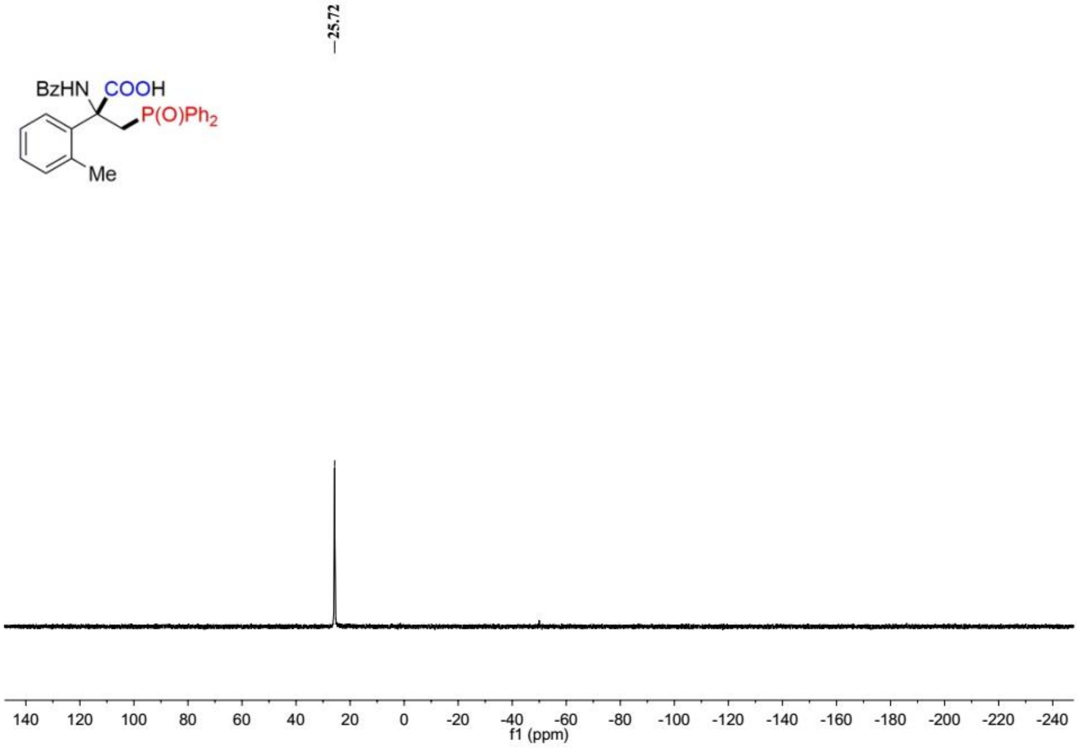

Supplementary Figure 78. ^{31}P NMR spectra of **3ua**

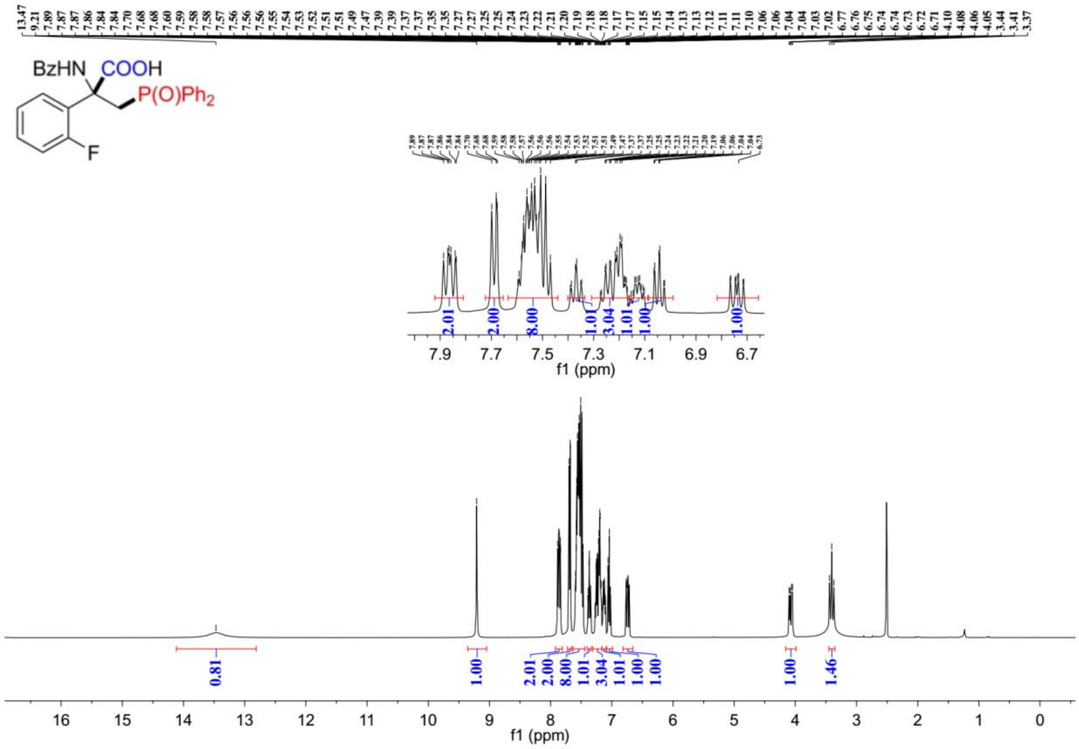

Supplementary Figure 79. ^{1}H NMR spectra of **3va**

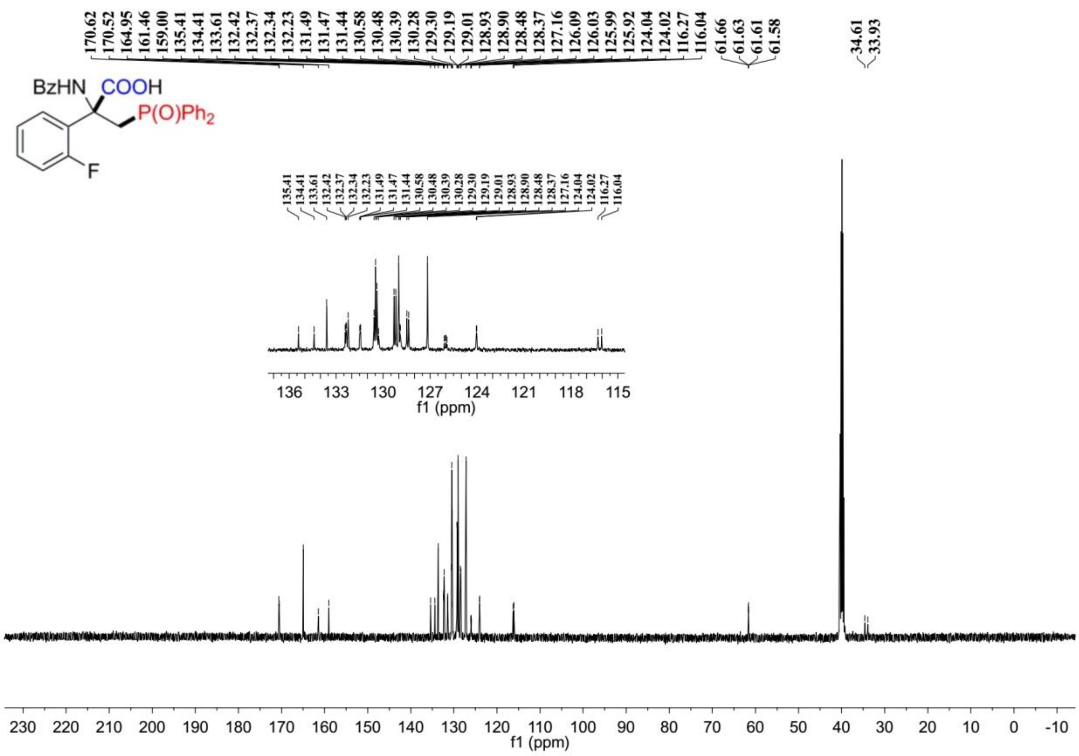

Supplementary Figure 80. ^{13}C NMR spectra of **3va**

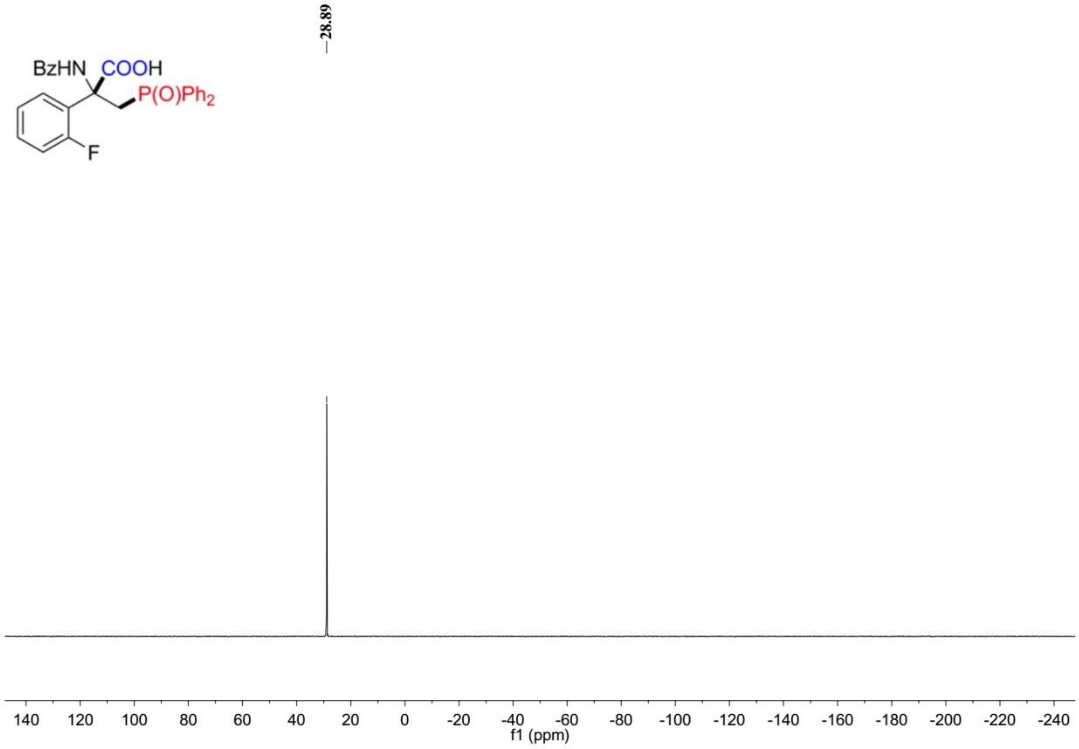

Supplementary Figure 81. ^{31}P NMR spectra of **3va**

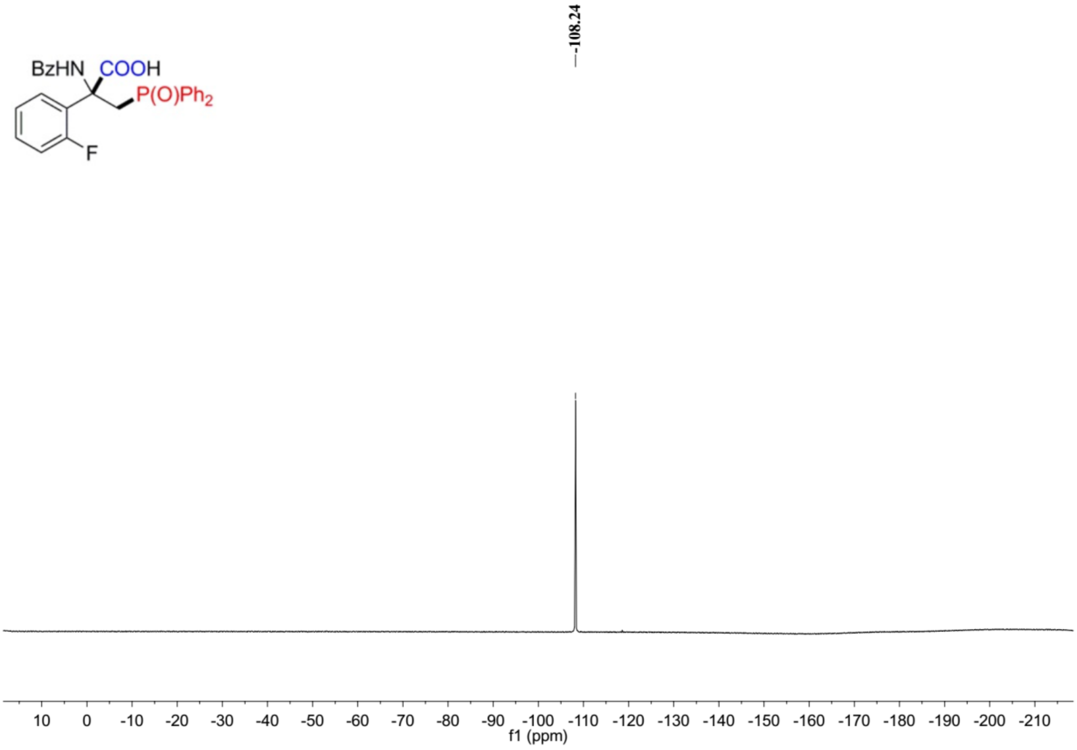

Supplementary Figure 82. ^{19}F NMR spectra of **3va**

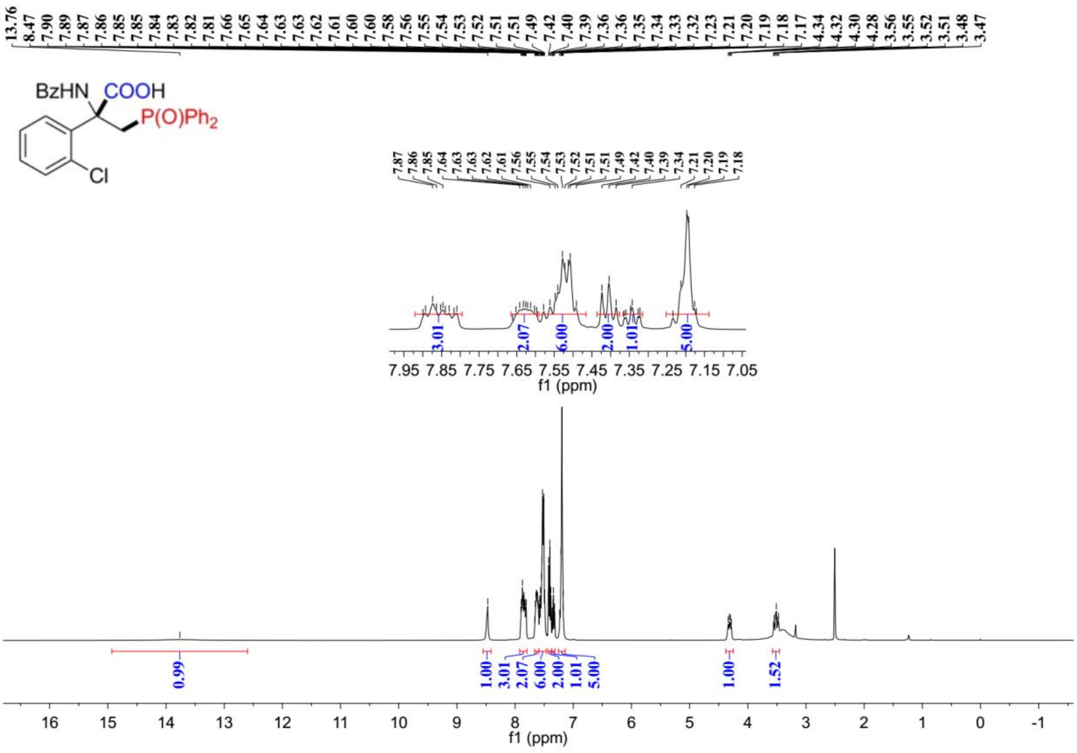

Supplementary Figure 83. ^{1}H NMR spectra of **3wa**

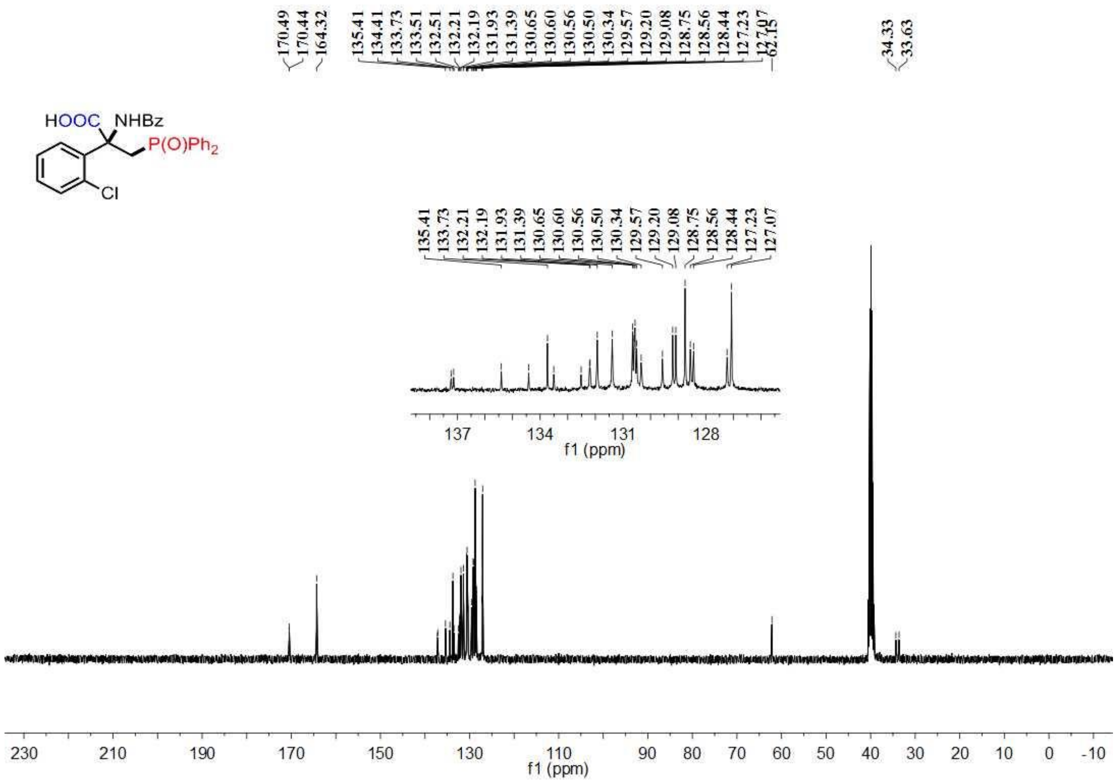

Supplementary Figure 84. ^{13}C NMR spectra of **3wa**

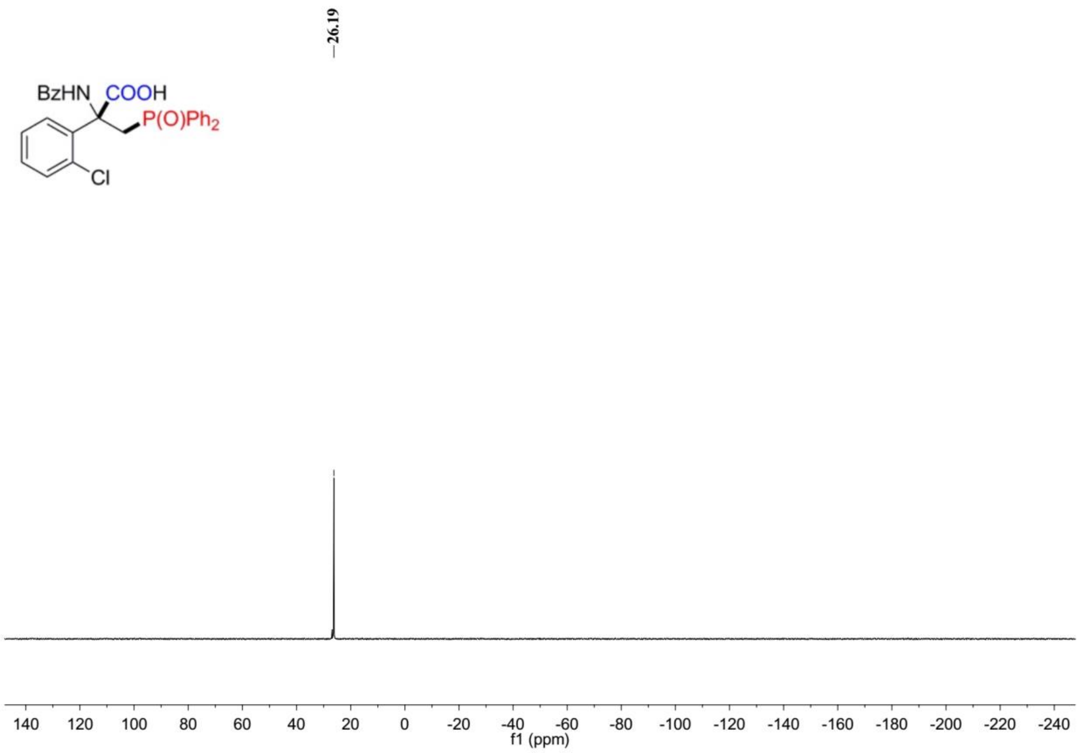

Supplementary Figure 85. ^{31}P NMR spectra of **3wa**

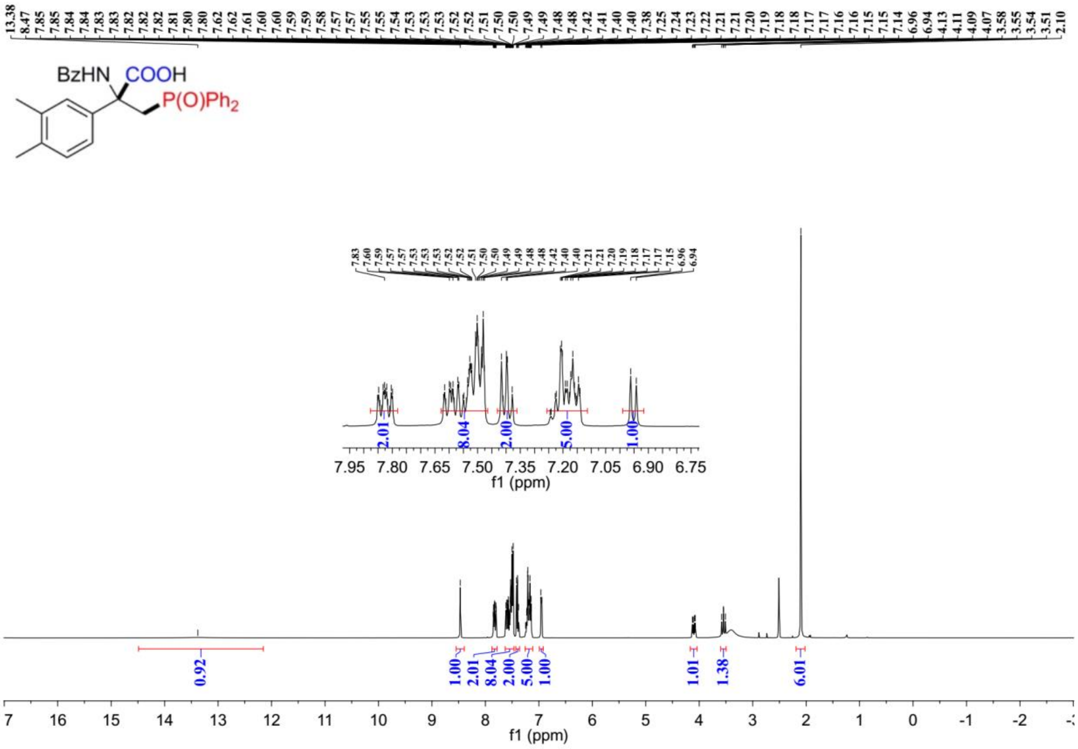

Supplementary Figure 86. ^{1}H NMR spectra of **3xa**

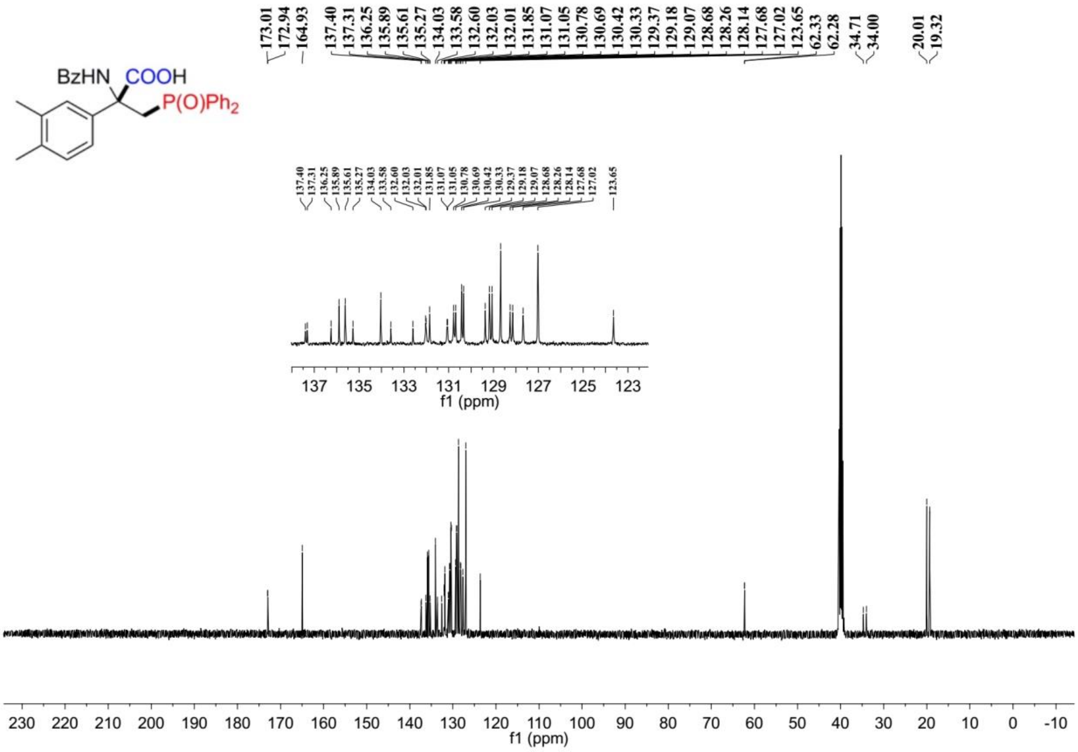

Supplementary Figure 87. ^{13}C NMR spectra of **3xa**

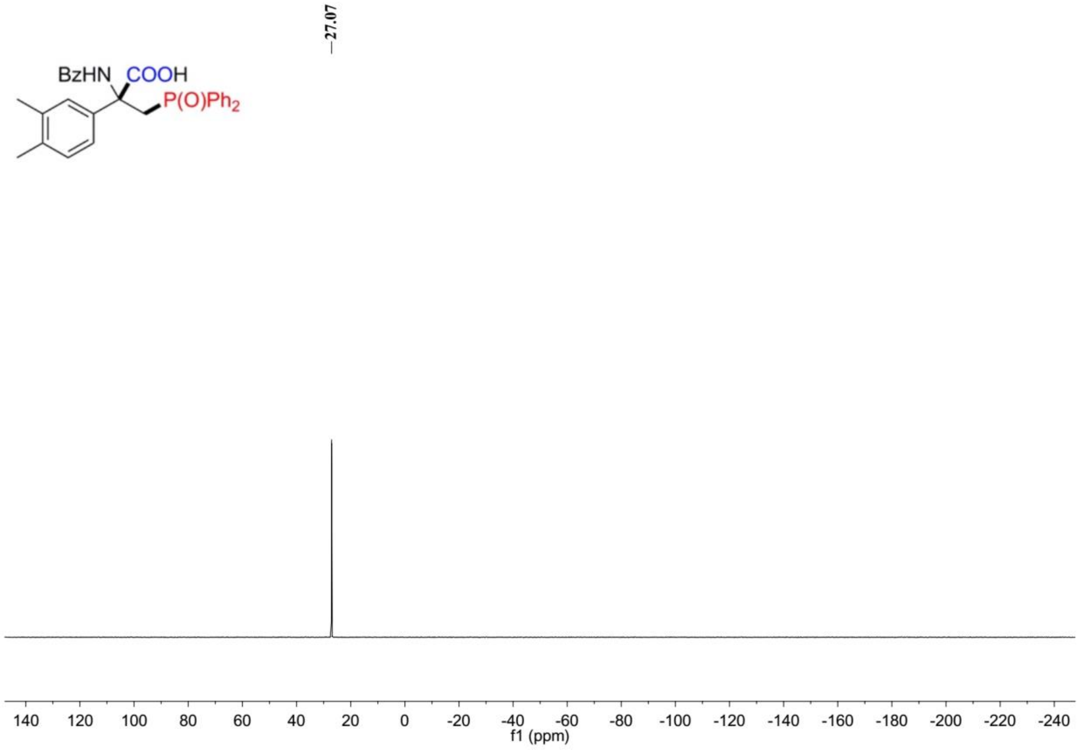

Supplementary Figure 88. ^{31}P NMR spectra of **3xa**

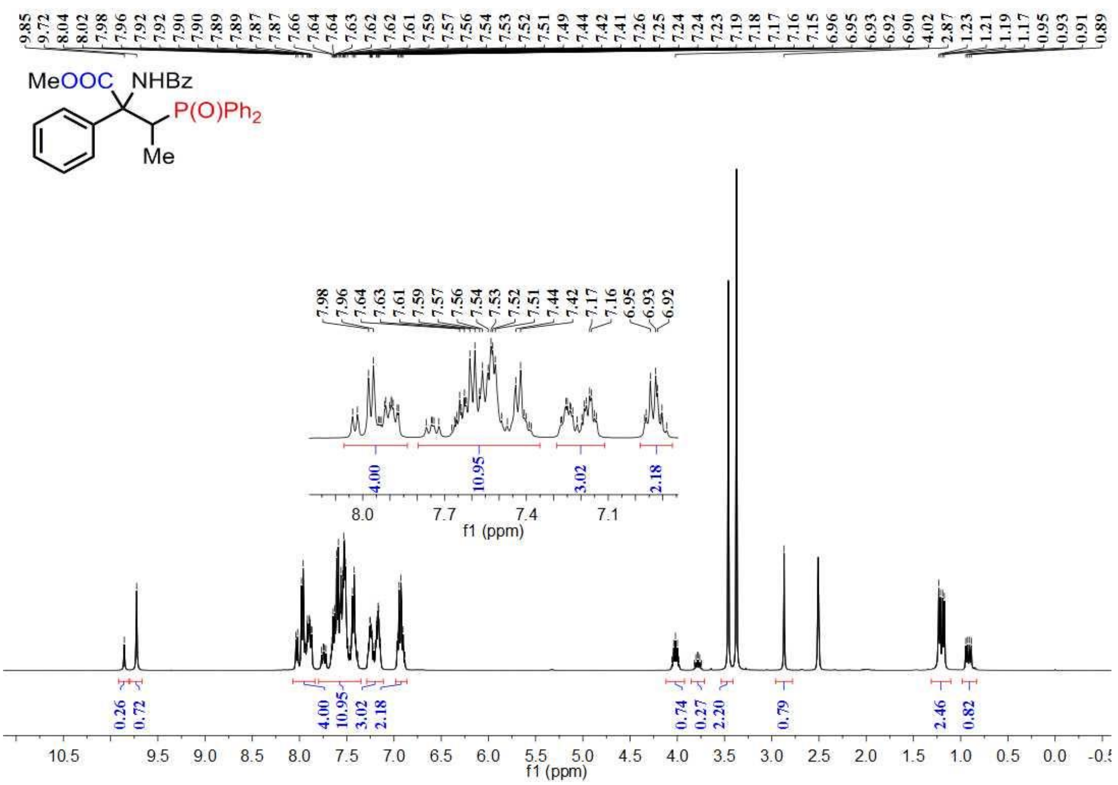

Supplementary Figure 89. ^{1}H NMR spectra of **3ya**

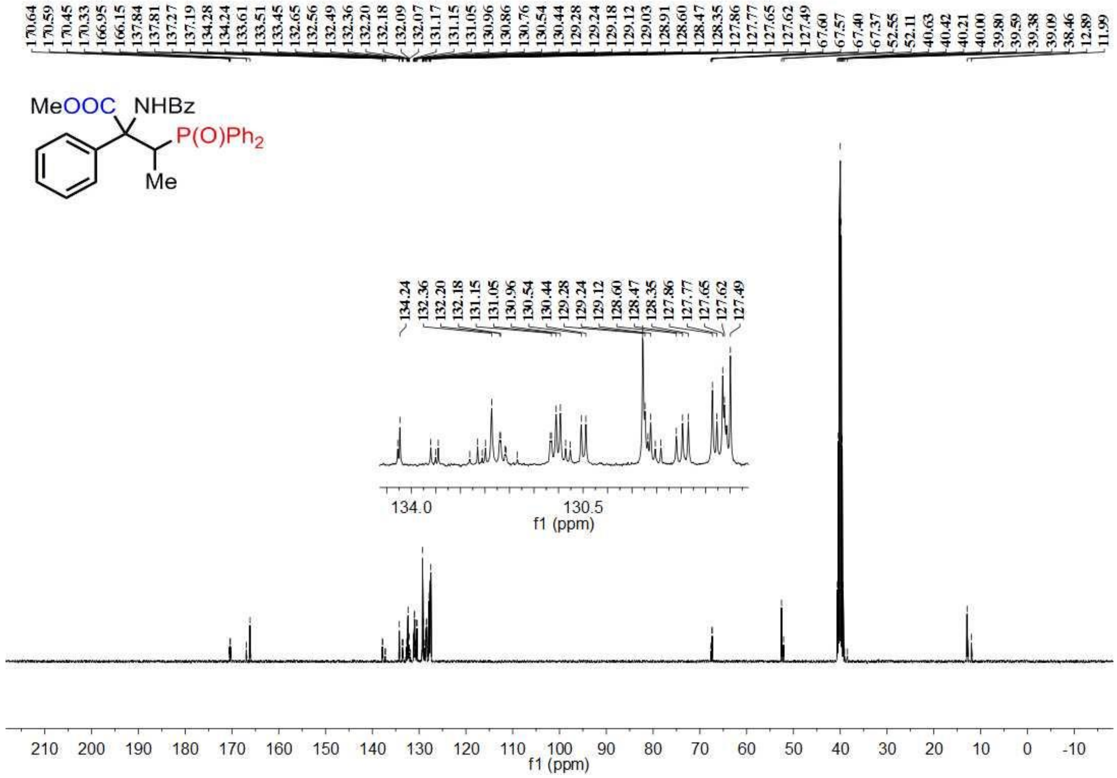

Supplementary Figure 90. ^{13}C NMR spectra of **3ya**

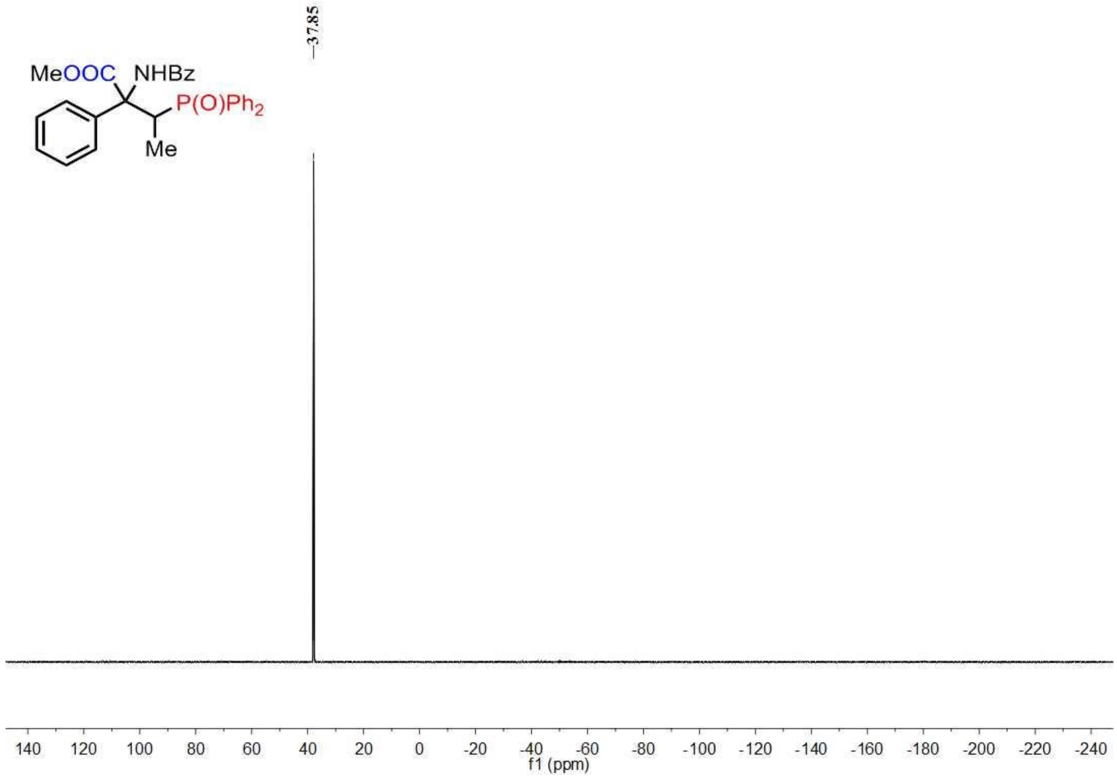

Supplementary Figure 91. ^{31}P NMR spectra of **3ya**

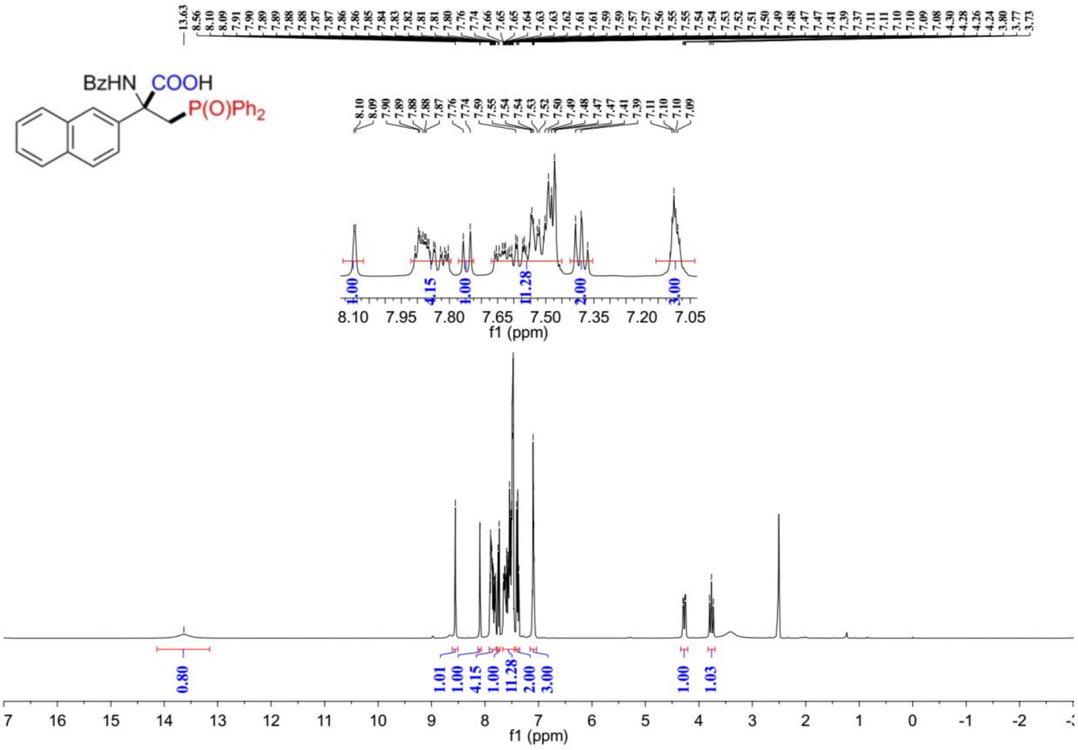

Supplementary Figure 92. ^{1}H NMR spectra of **3za**

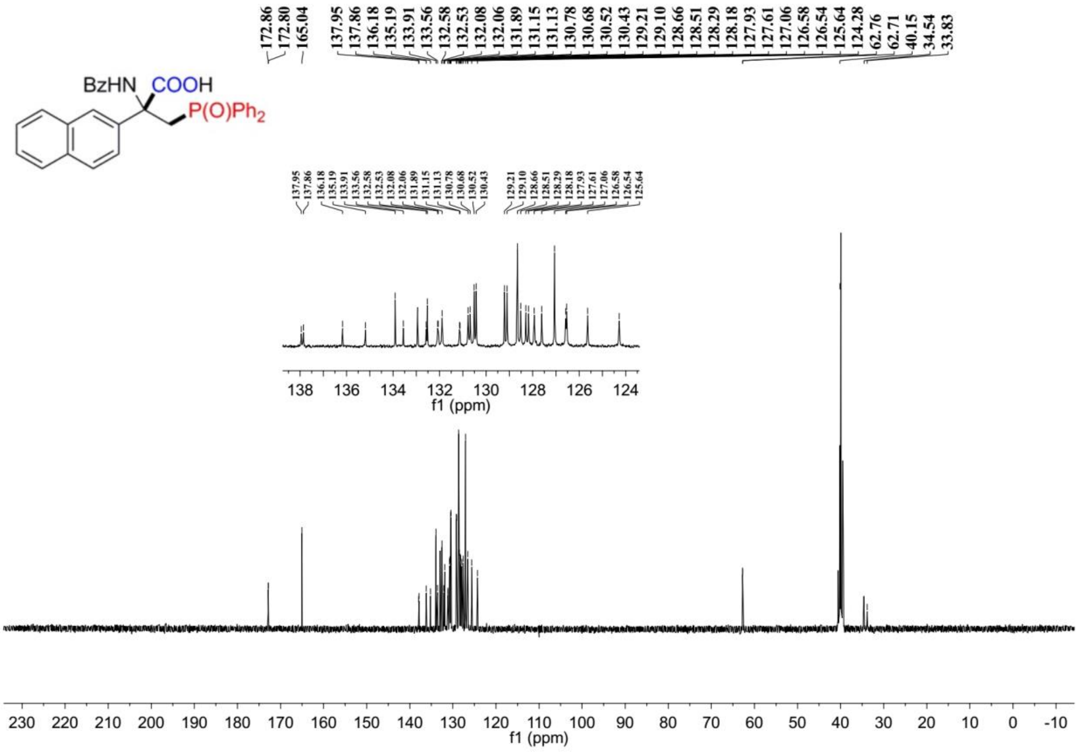

Supplementary Figure 93. ^{13}C NMR spectra of **3za**

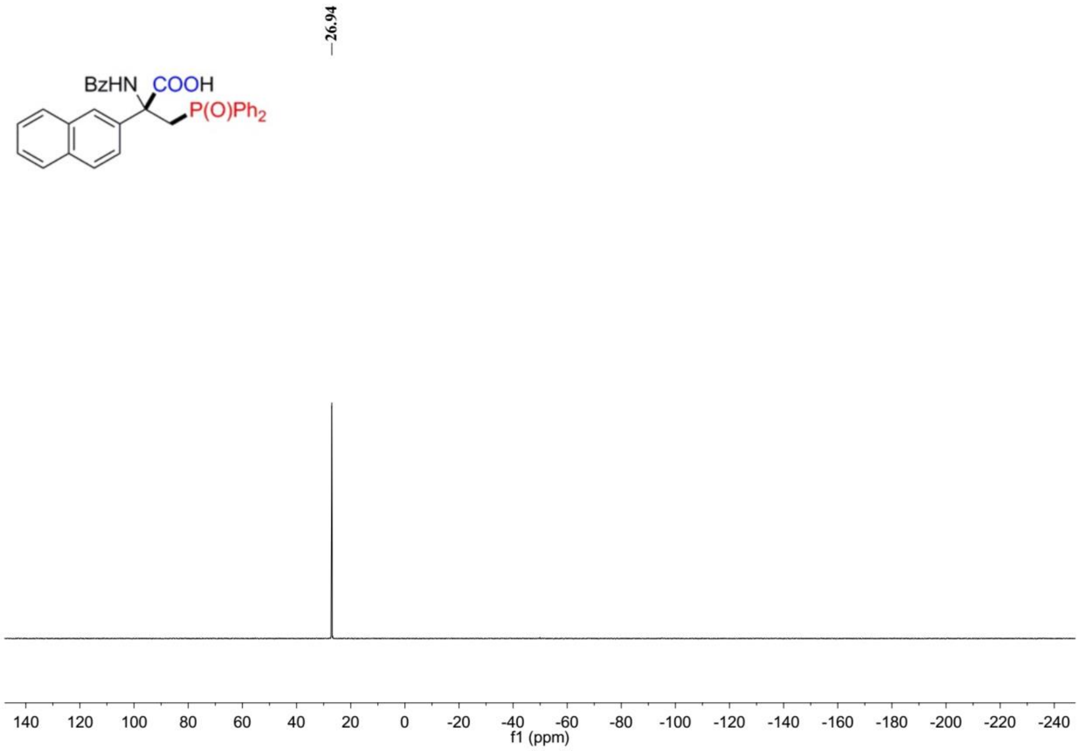

Supplementary Figure 94. ^{31}P NMR spectra of **3za**

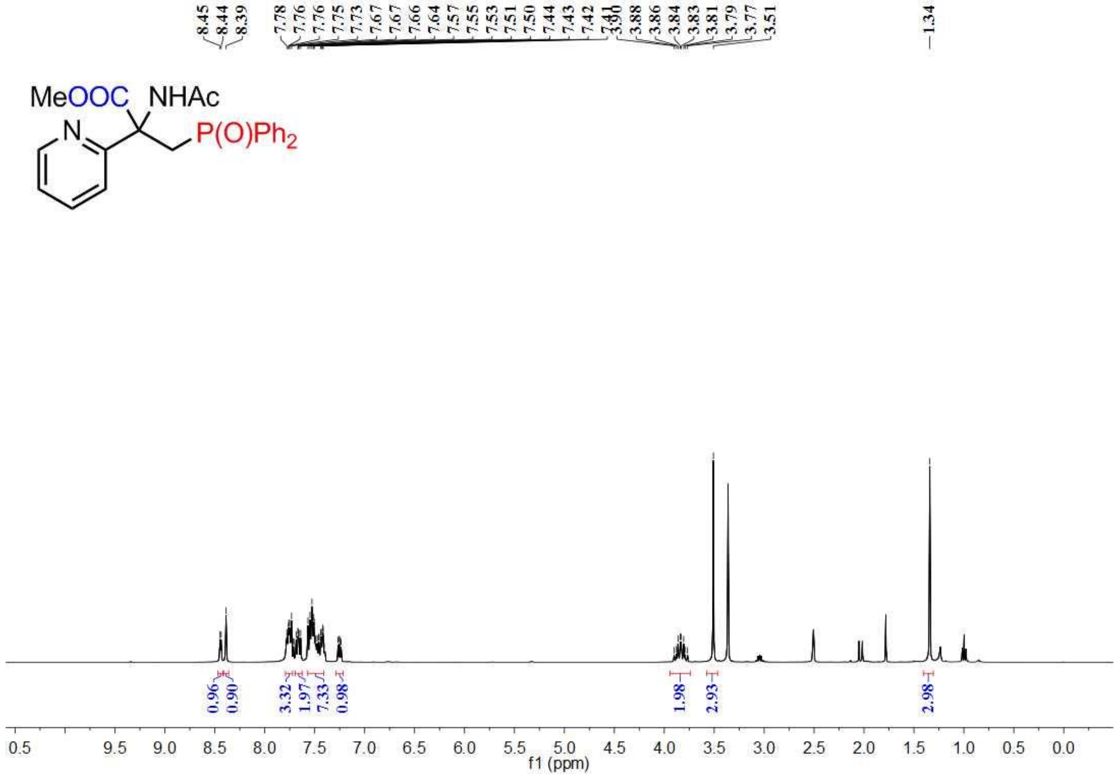

Supplementary Figure 95. ^{1}H NMR spectra of **3aaa**

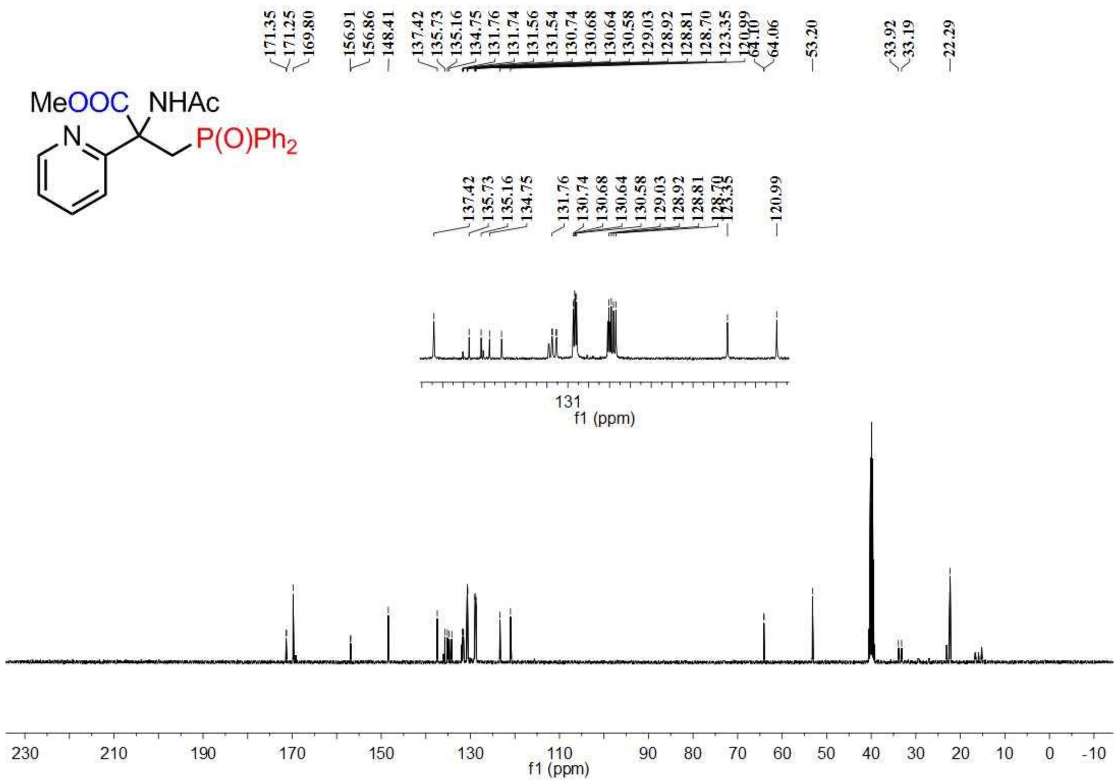

Supplementary Figure 96. ^{13}C NMR spectra of **3aaa**

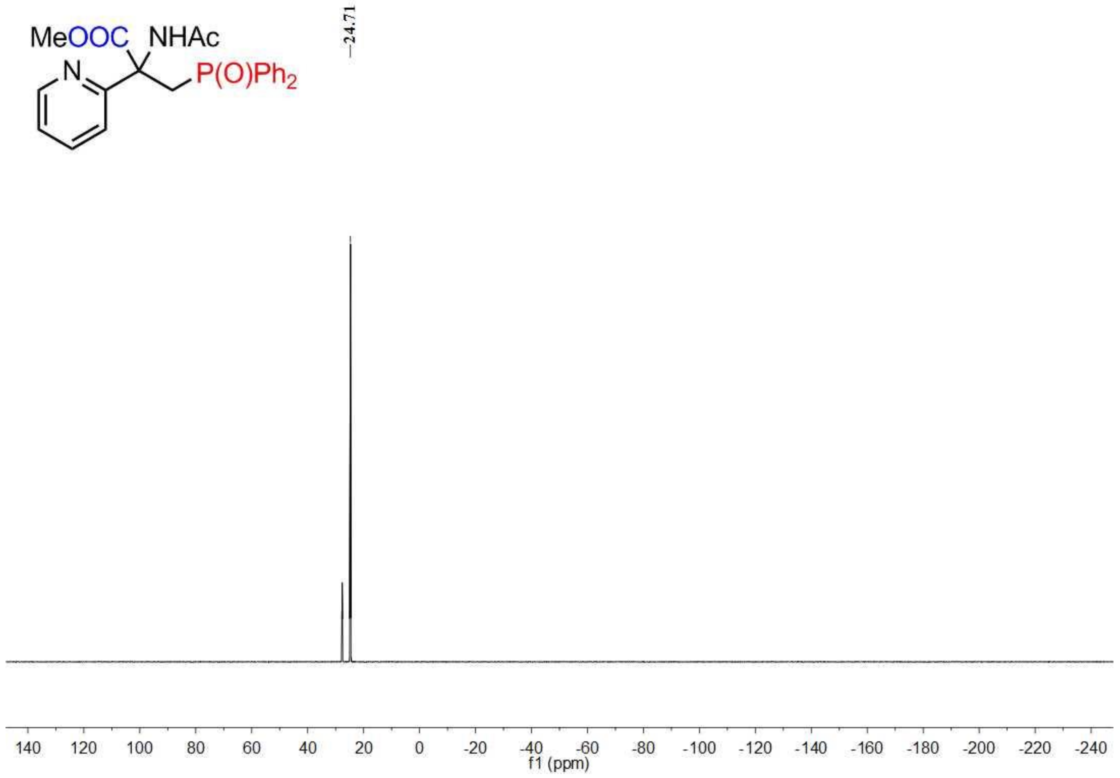

Supplementary Figure 97. ^{31}P NMR spectra of **3aaa**

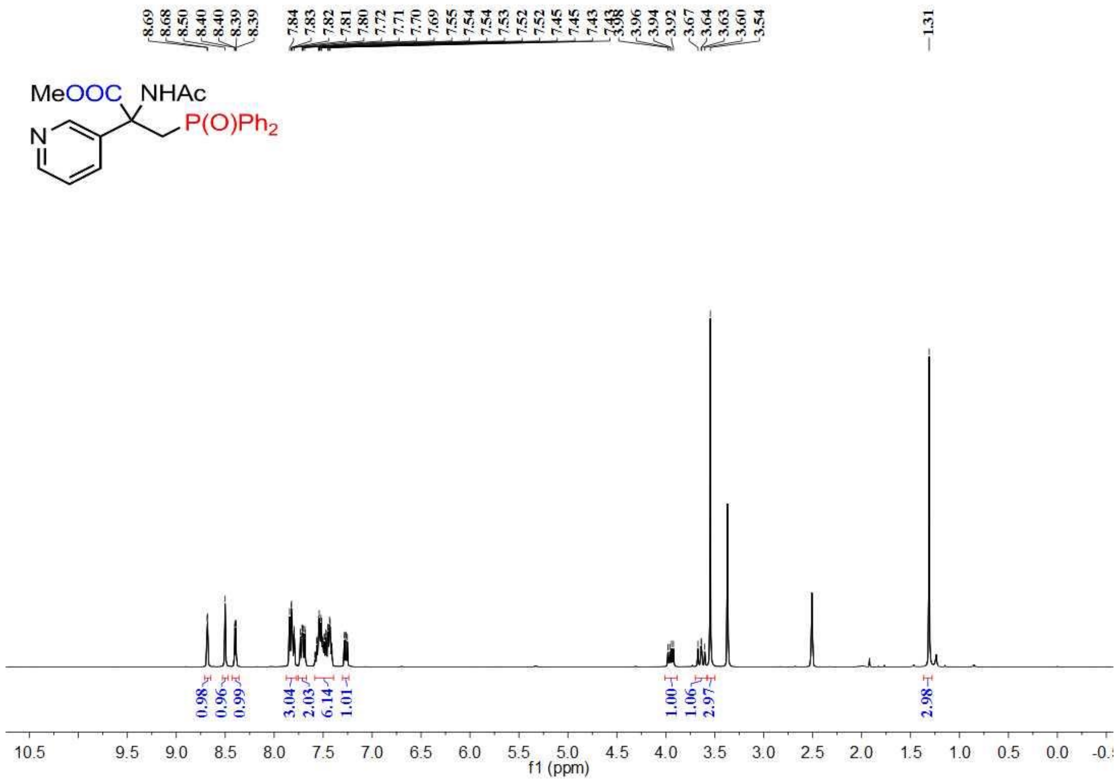

Supplementary Figure 98. ^{1}H NMR spectra of **3aba**

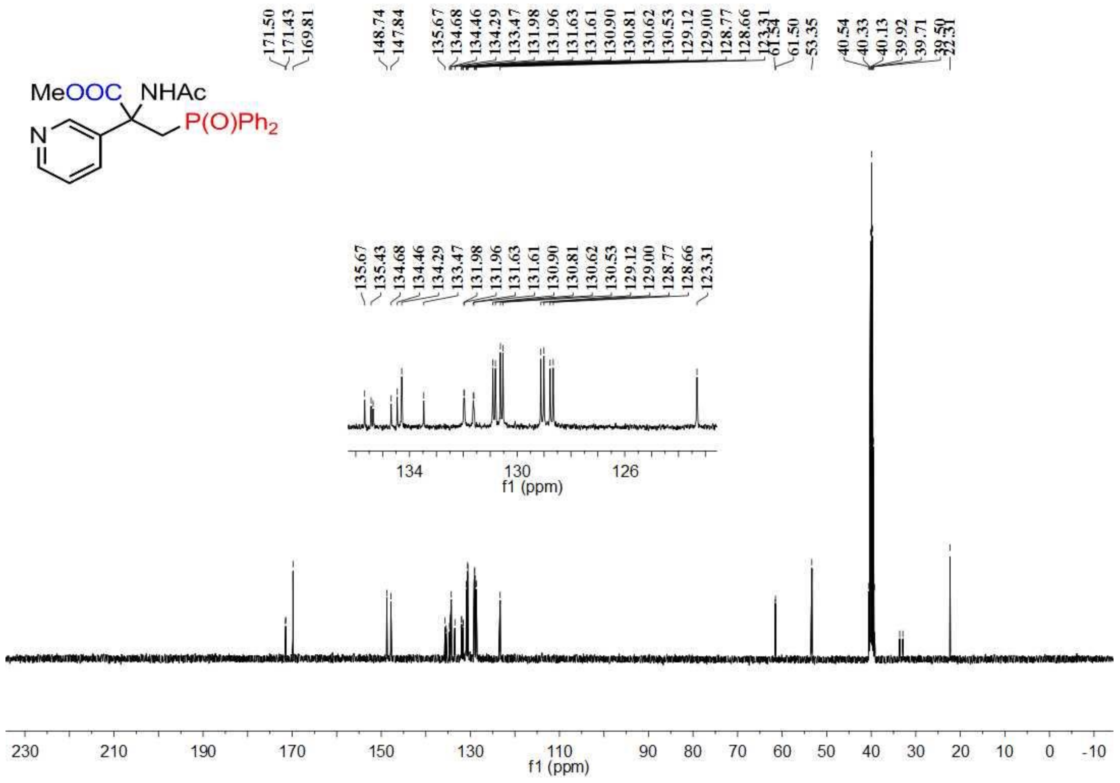

Supplementary Figure 99. ^{13}C NMR spectra of **3aba**

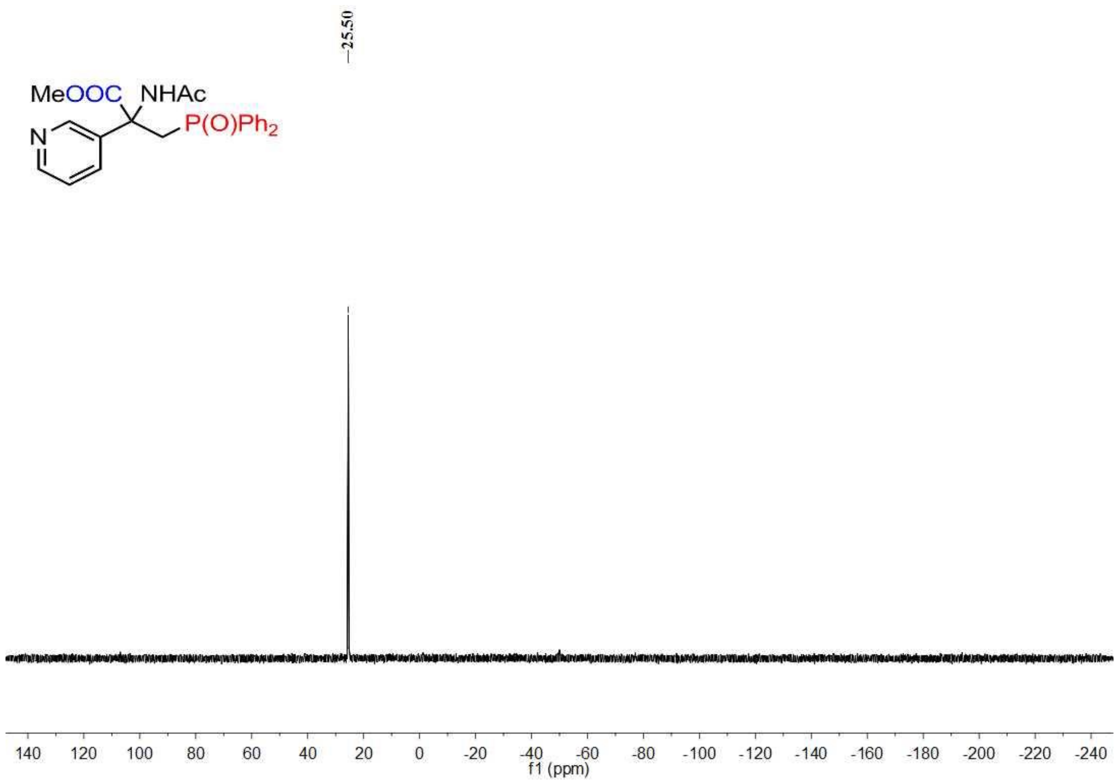

Supplementary Figure 100. ^{31}P NMR spectra of **3aba**

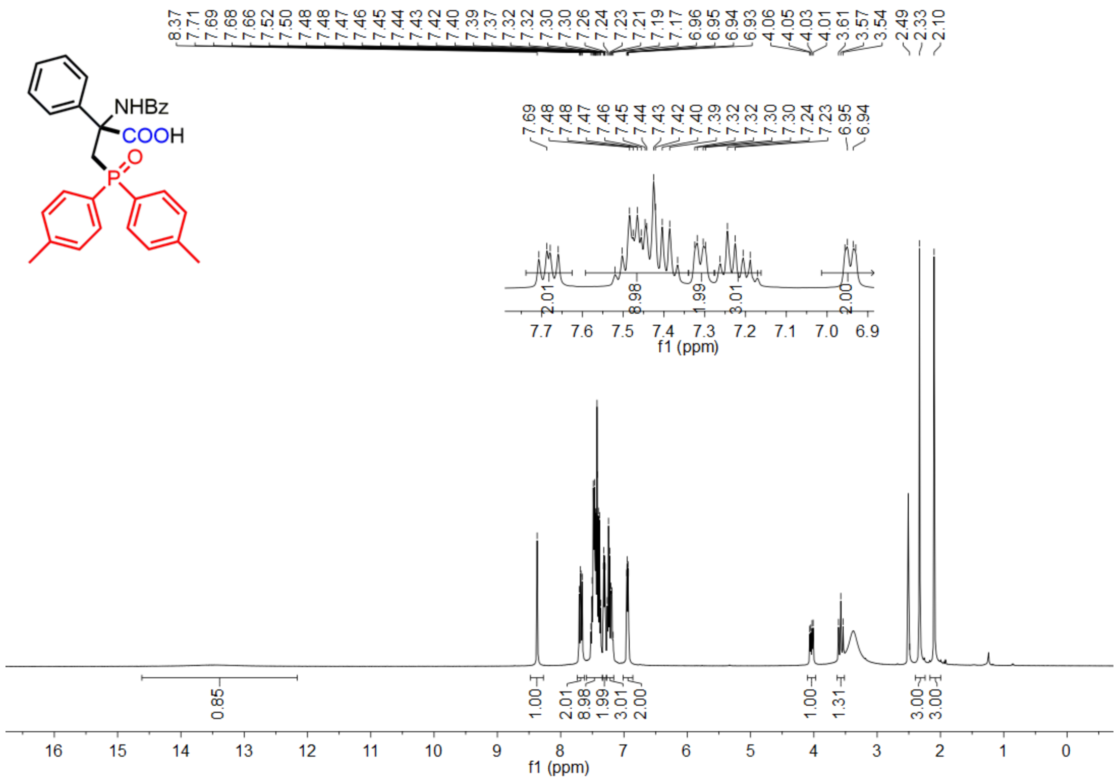

Supplementary Figure 101. ^{1}H NMR spectra of **3ab**

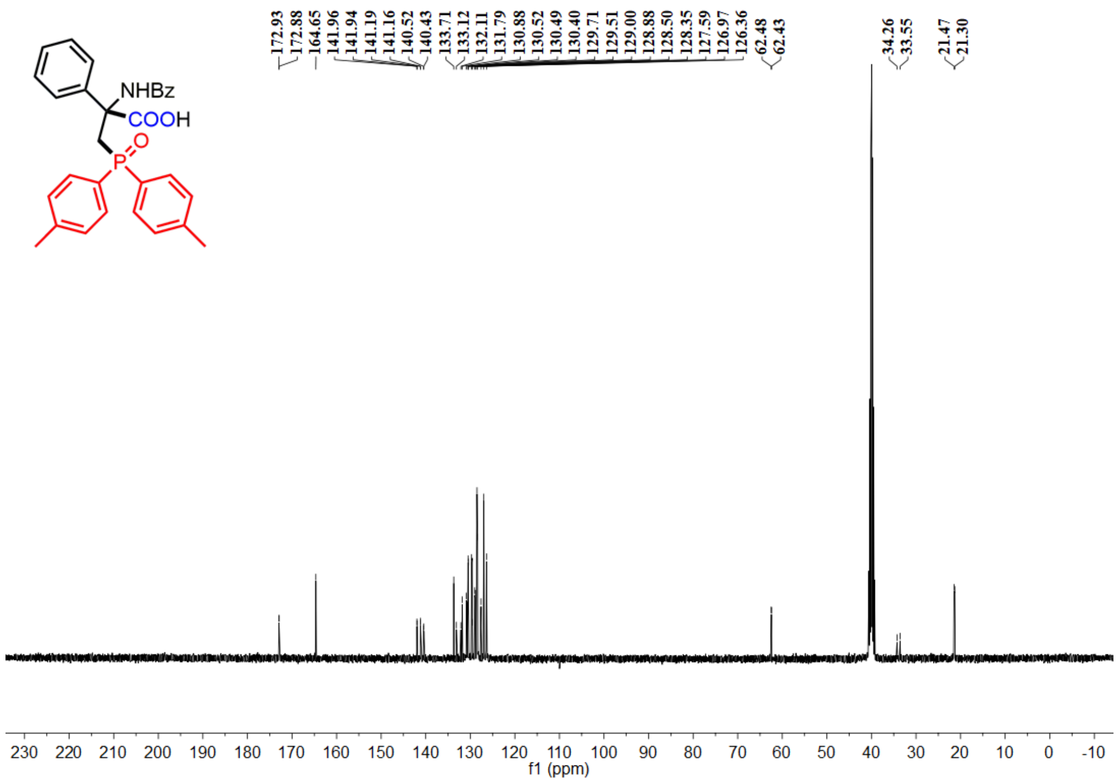

Supplementary Figure 102. ^{13}C NMR spectra of **3ab**

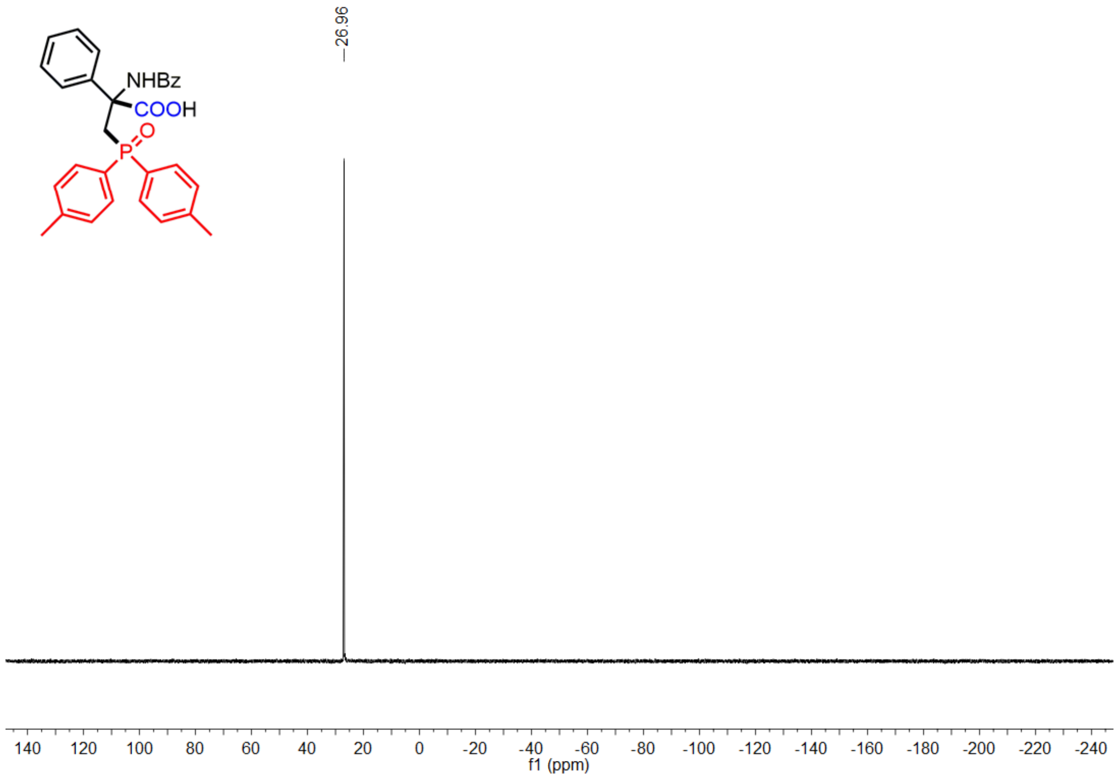

Supplementary Figure 103. ^{31}P NMR spectra of **3ab**

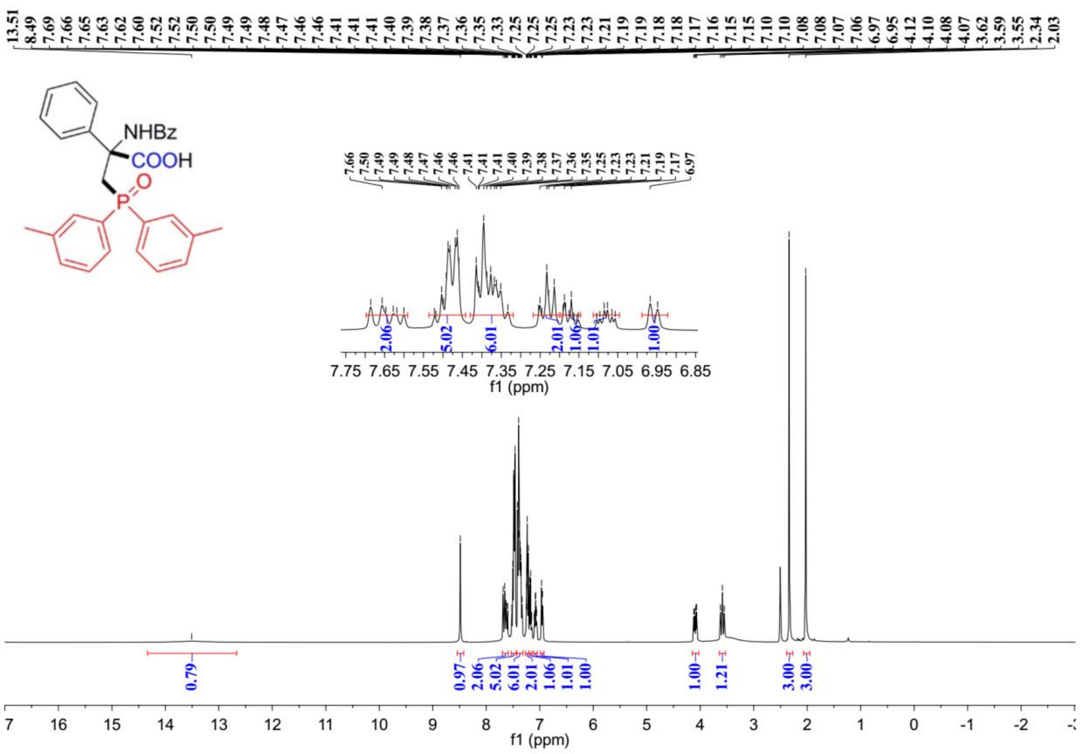

Supplementary Figure 104. ^{1}H NMR spectra of **3ac**

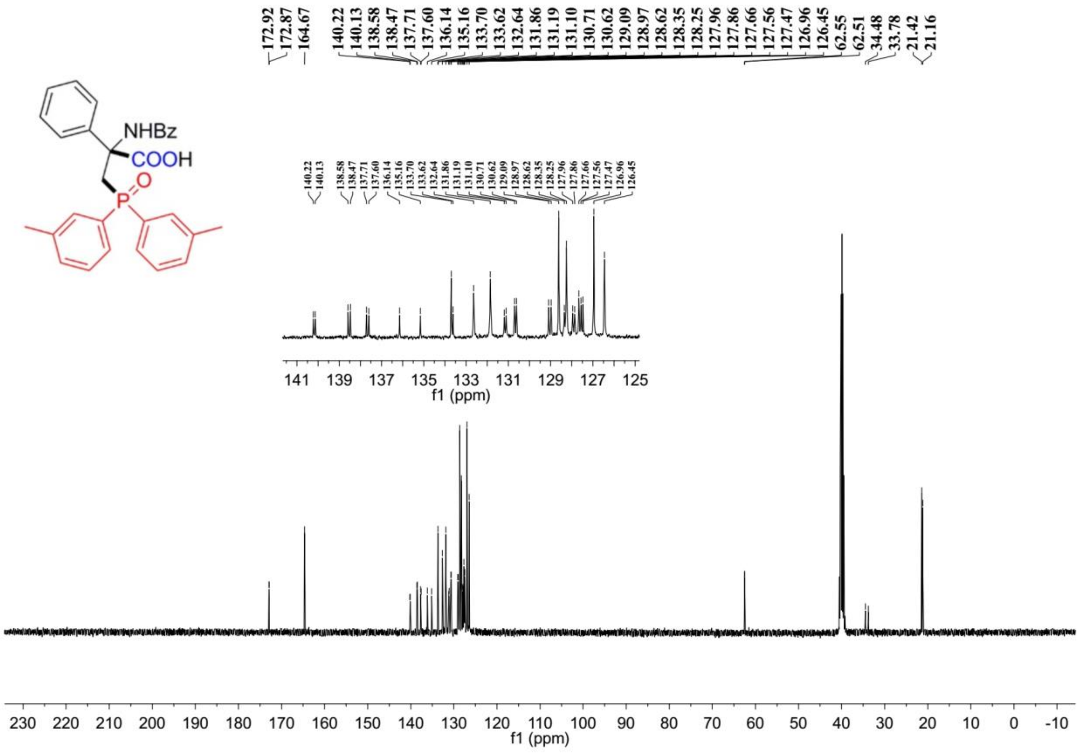

Supplementary Figure 105. ^{13}C NMR spectra of **3ac**

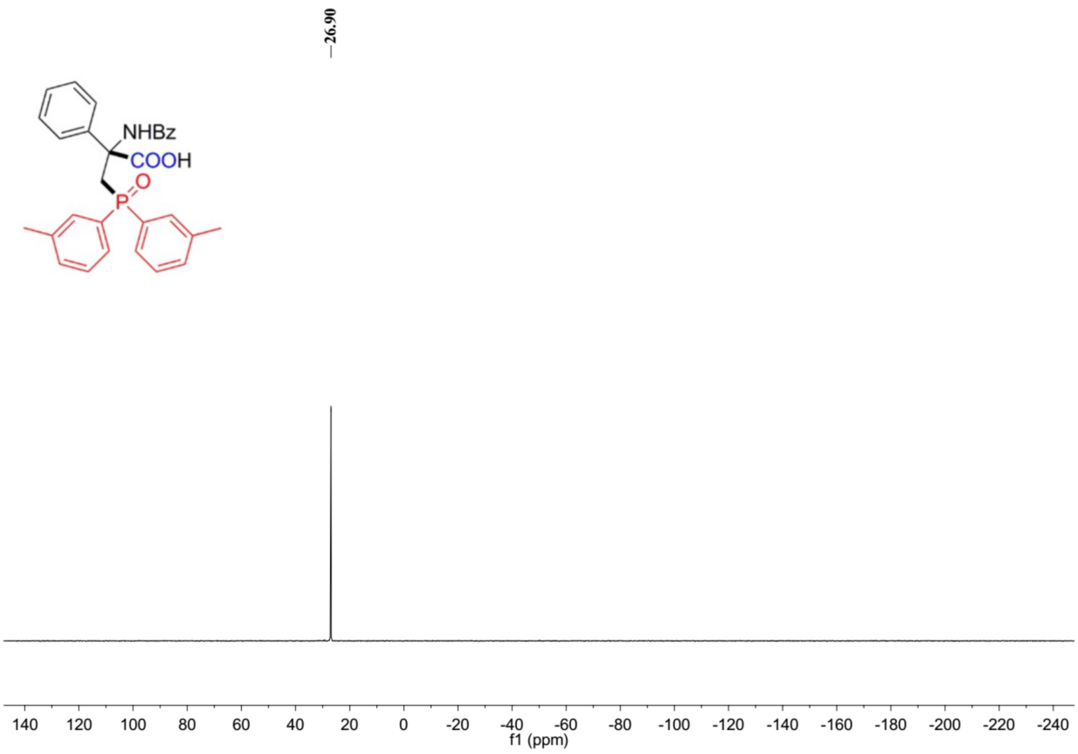

Supplementary Figure 106. ^{31}P NMR spectra of **3ac**

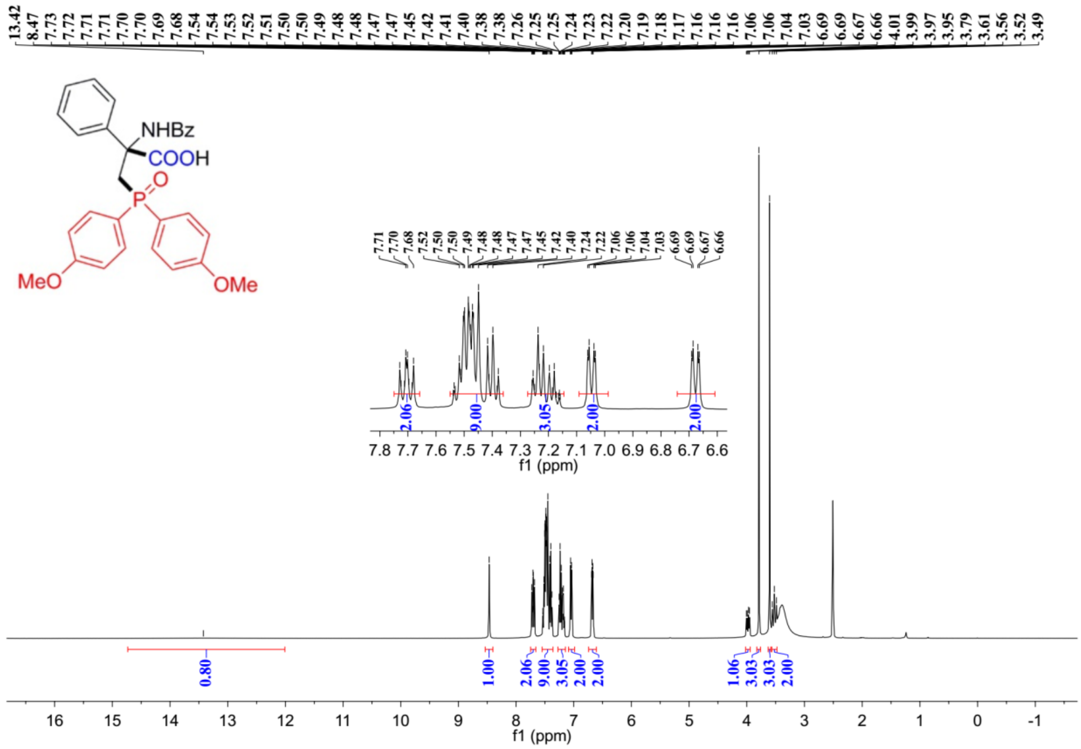

Supplementary Figure 107. ^{1}H NMR spectra of **3ca**

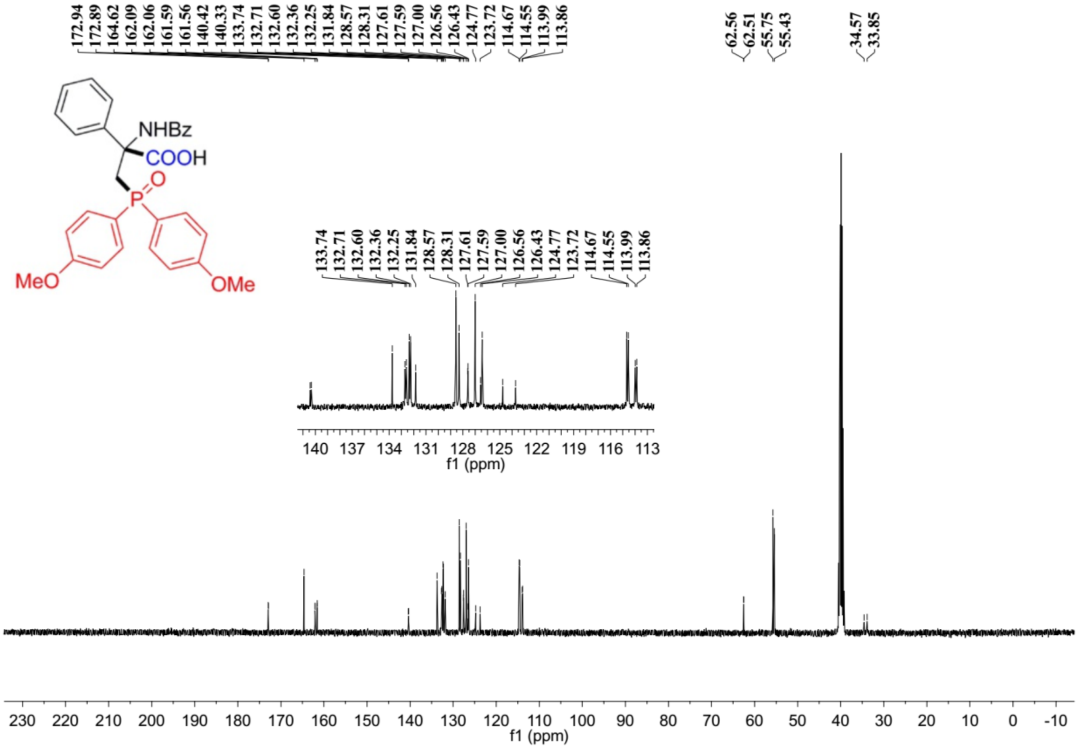

Supplementary Figure 108. ^{13}C NMR spectra of **3ca**

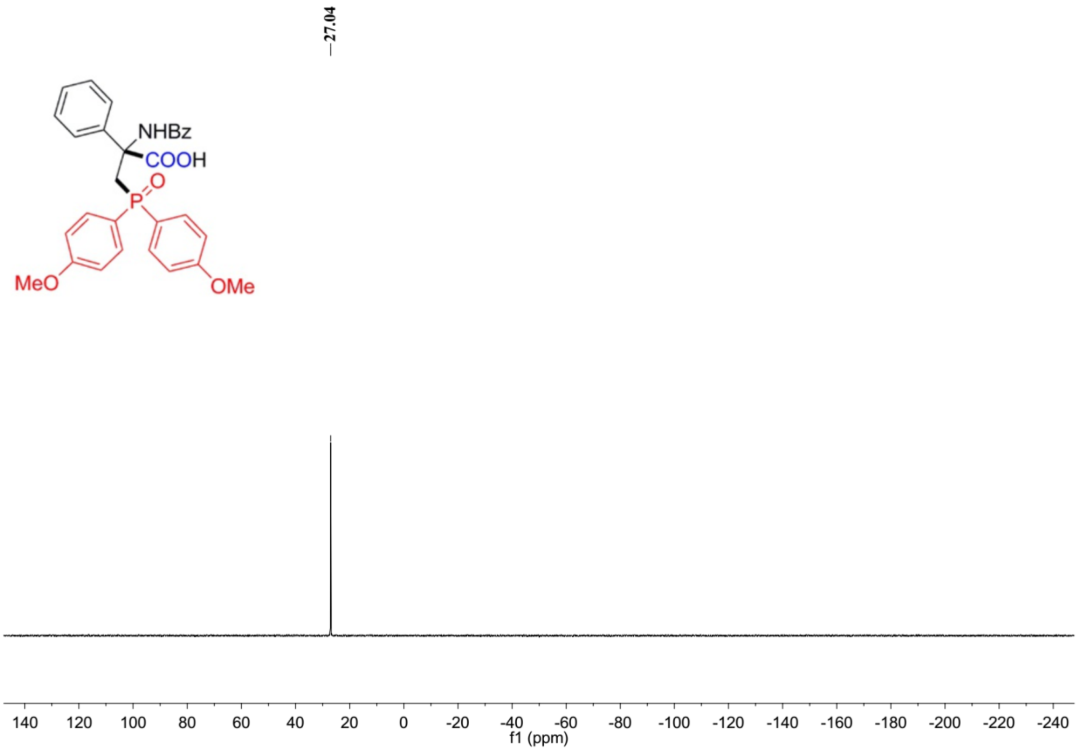

Supplementary Figure 109. ^{31}P NMR spectra of **3ca**

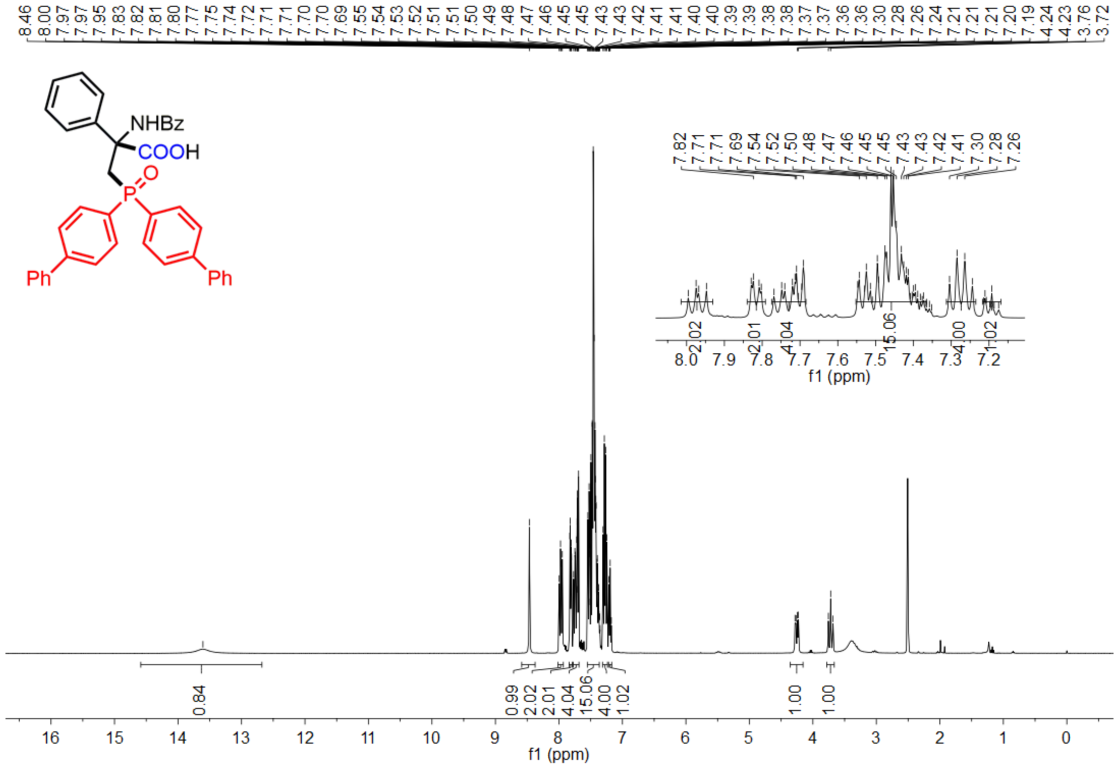

Supplementary Figure 110. ^{1}H NMR spectra of **3ae**

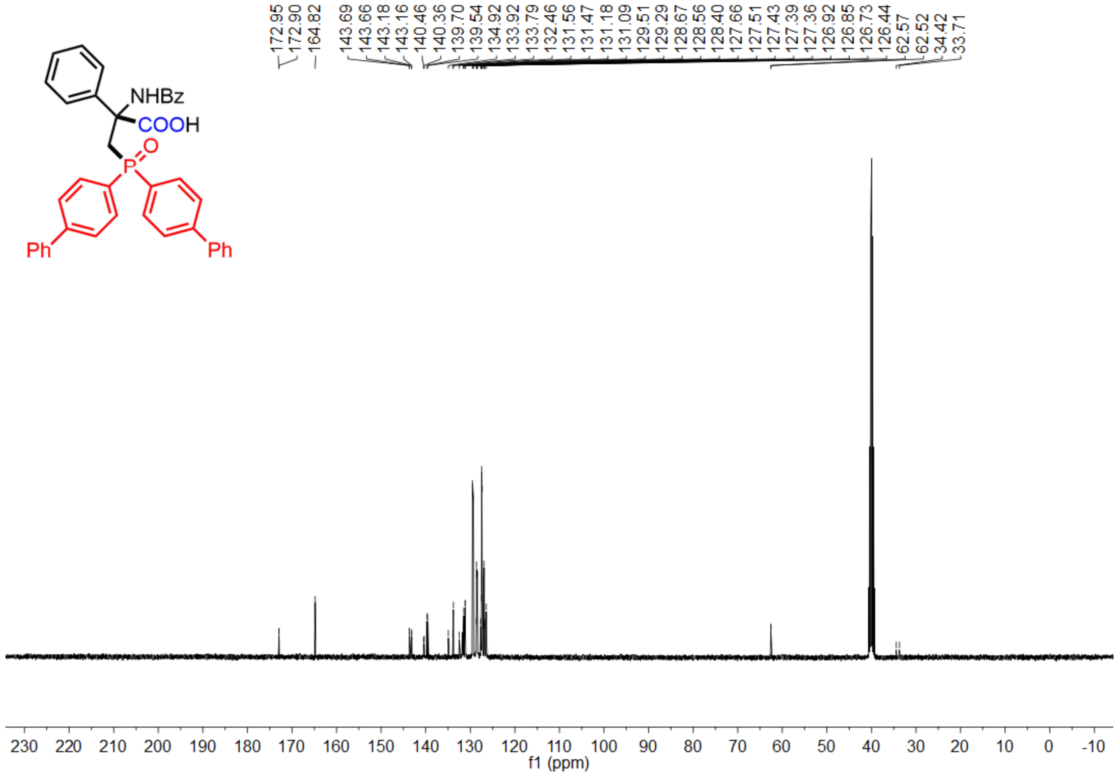

Supplementary Figure 111. ^{13}C NMR spectra of **3ae**

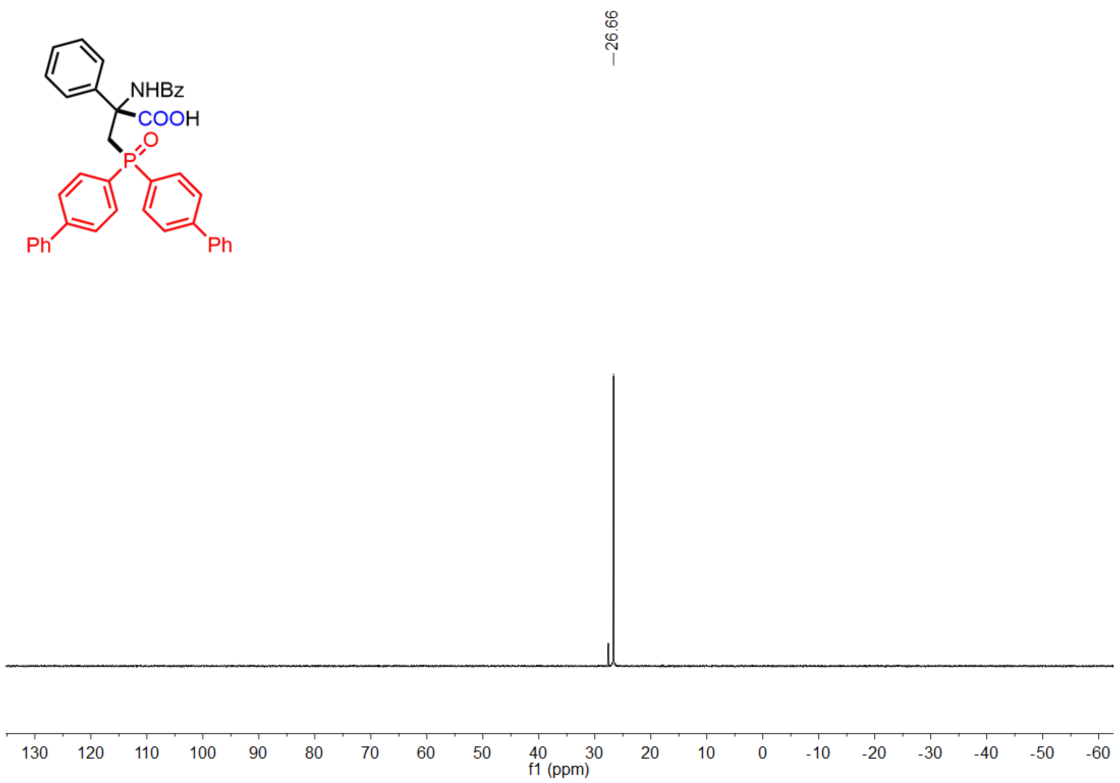

Supplementary Figure 112. ^{31}P NMR spectra of **3ae**

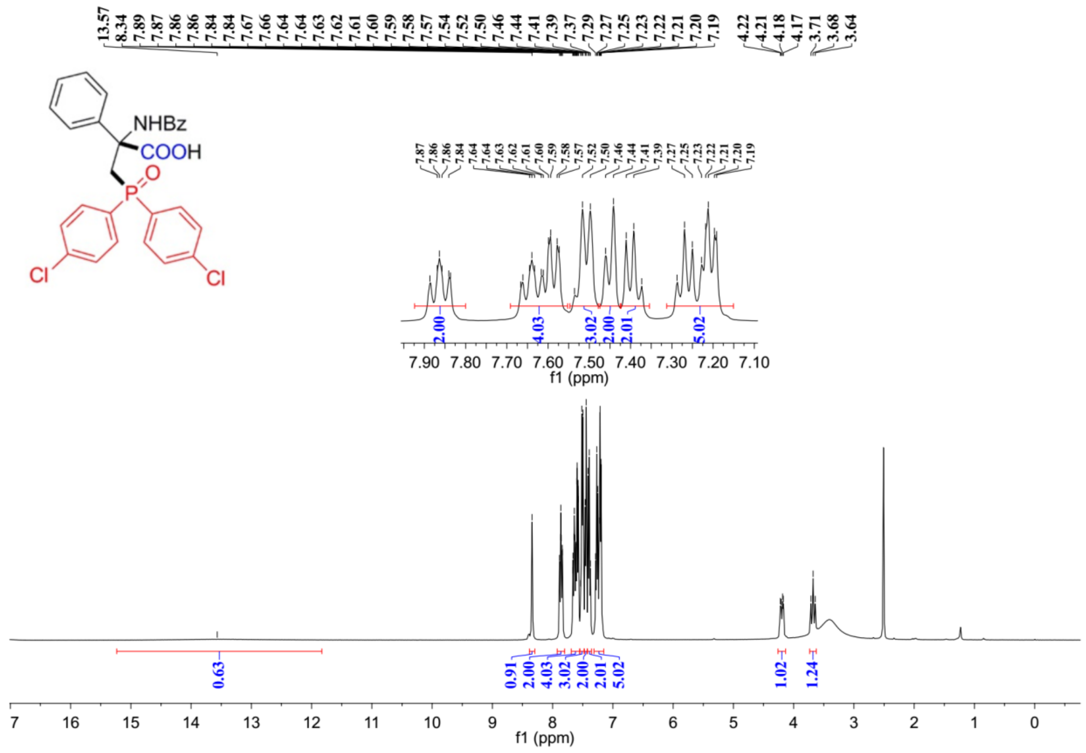

Supplementary Figure 113. ^{1}H NMR spectra of **3af**

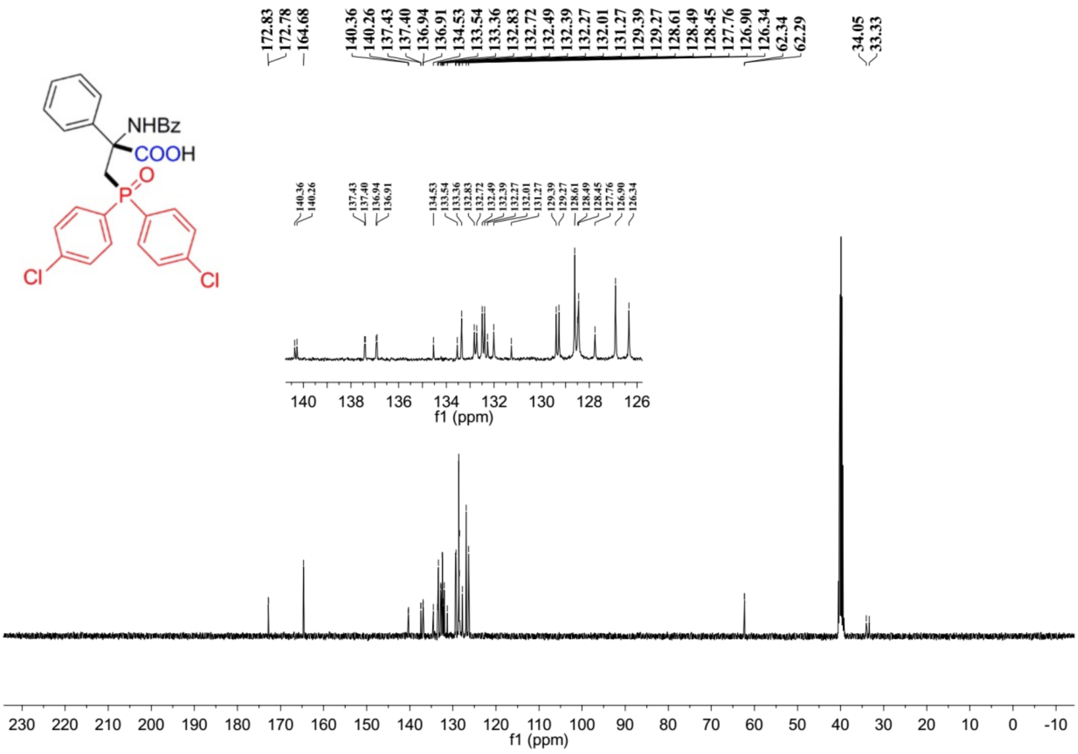

Supplementary Figure 114. ^{13}C NMR spectra of **3af**

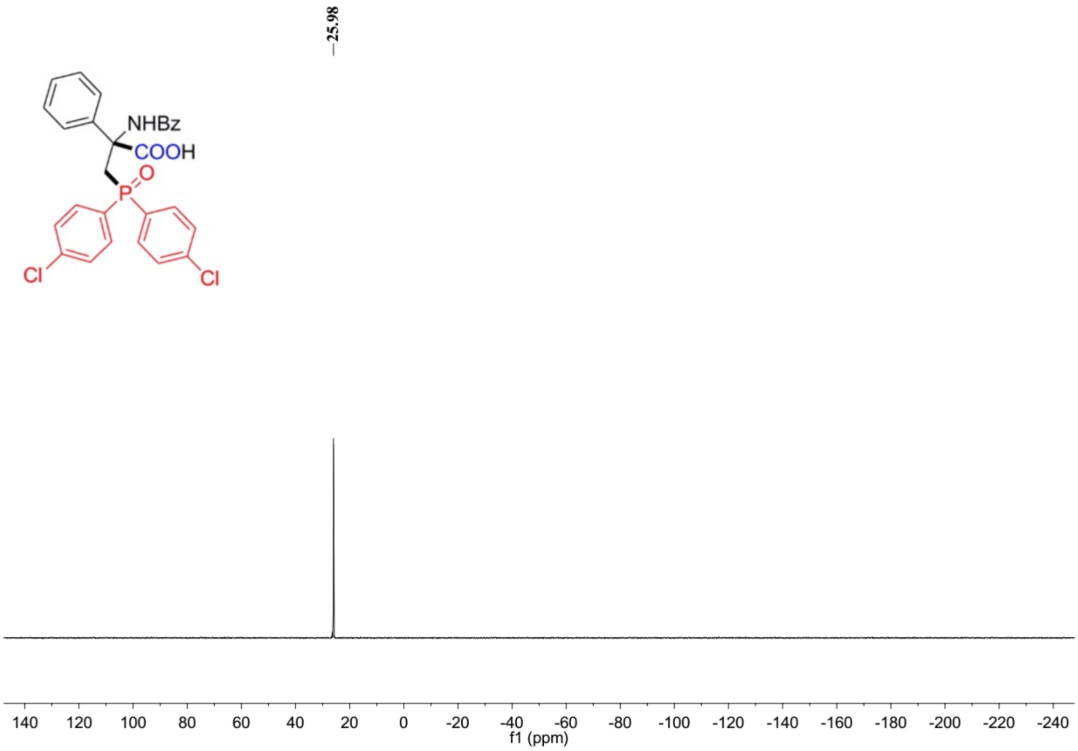

Supplementary Figure 115. ^{31}P NMR spectra of **3af**

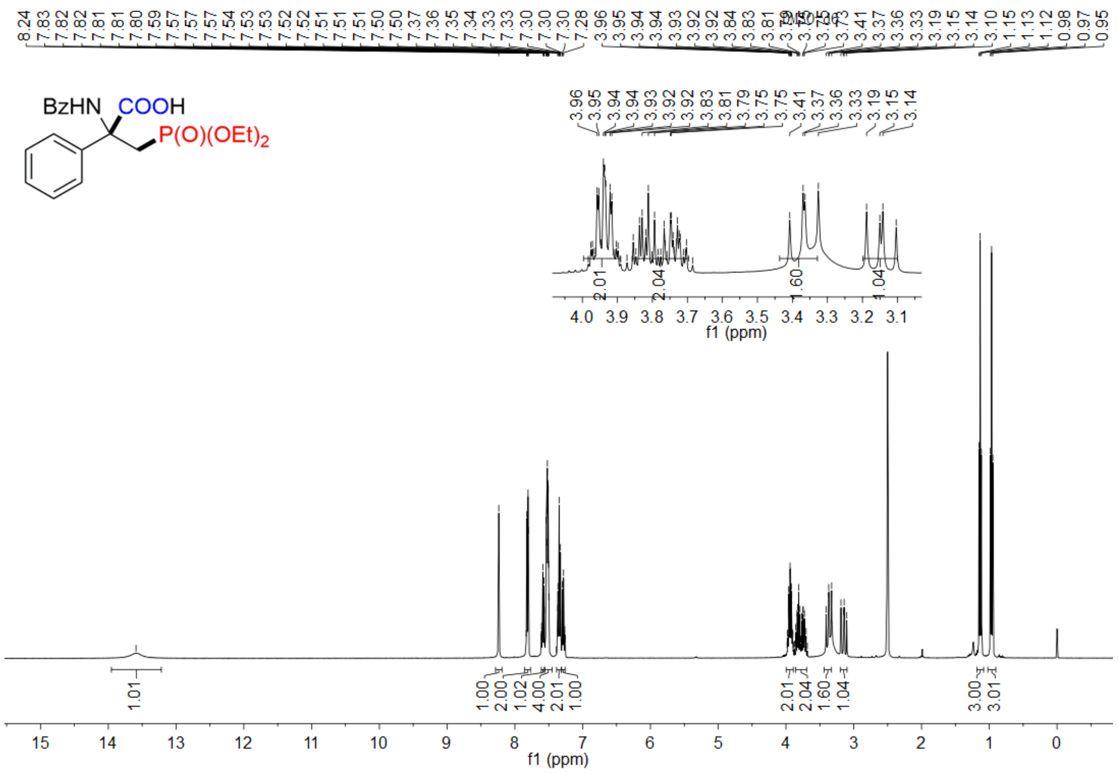

Supplementary Figure 116. ^{1}H NMR spectra of **3ag**

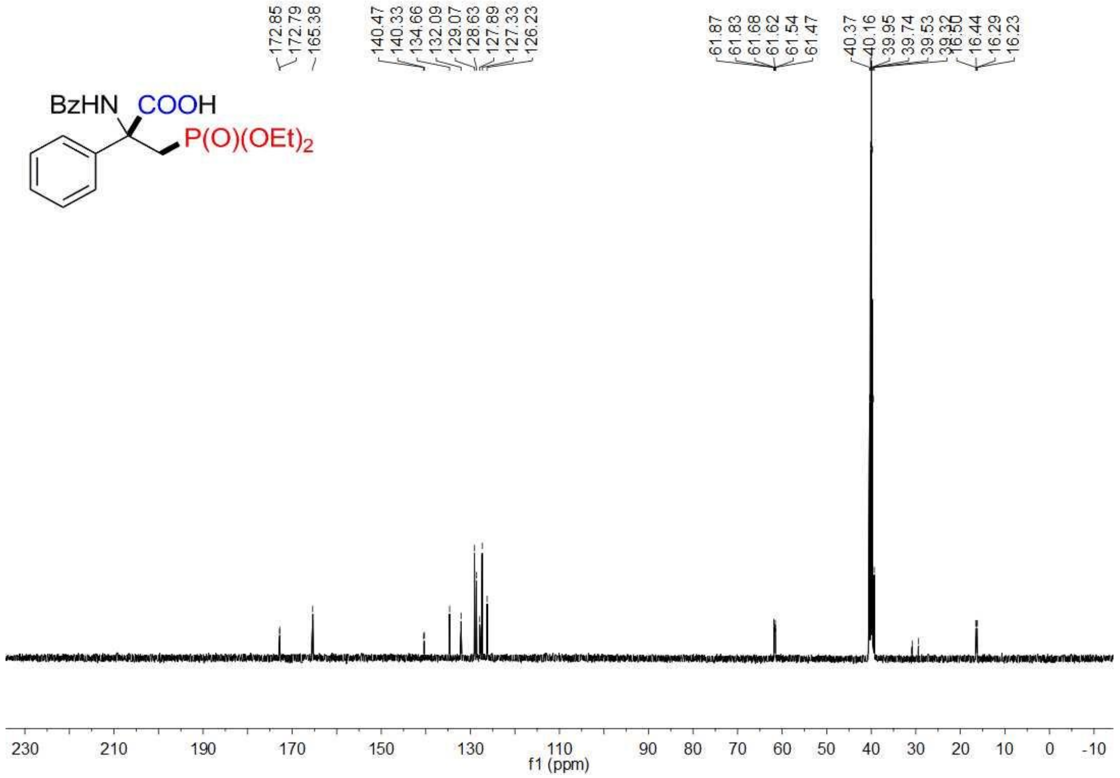

Supplementary Figure 117. ^{13}C NMR spectra of **3ag**

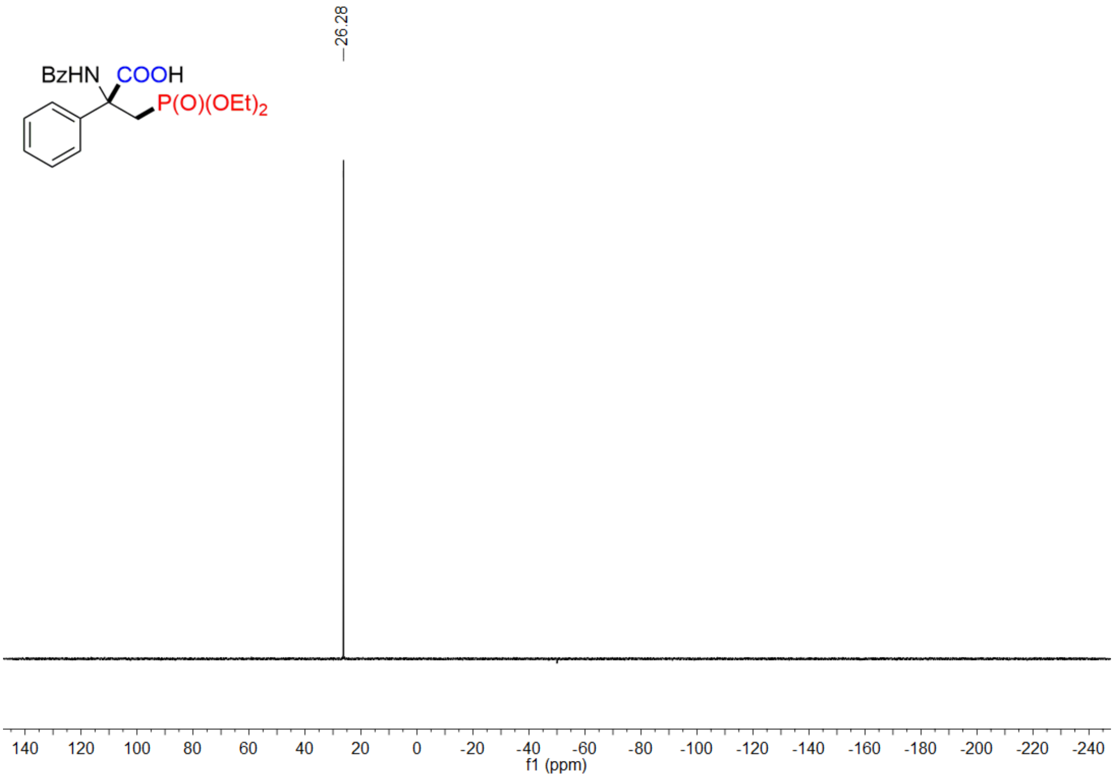

Supplementary Figure 118. ^{31}P NMR spectra of **3ag**

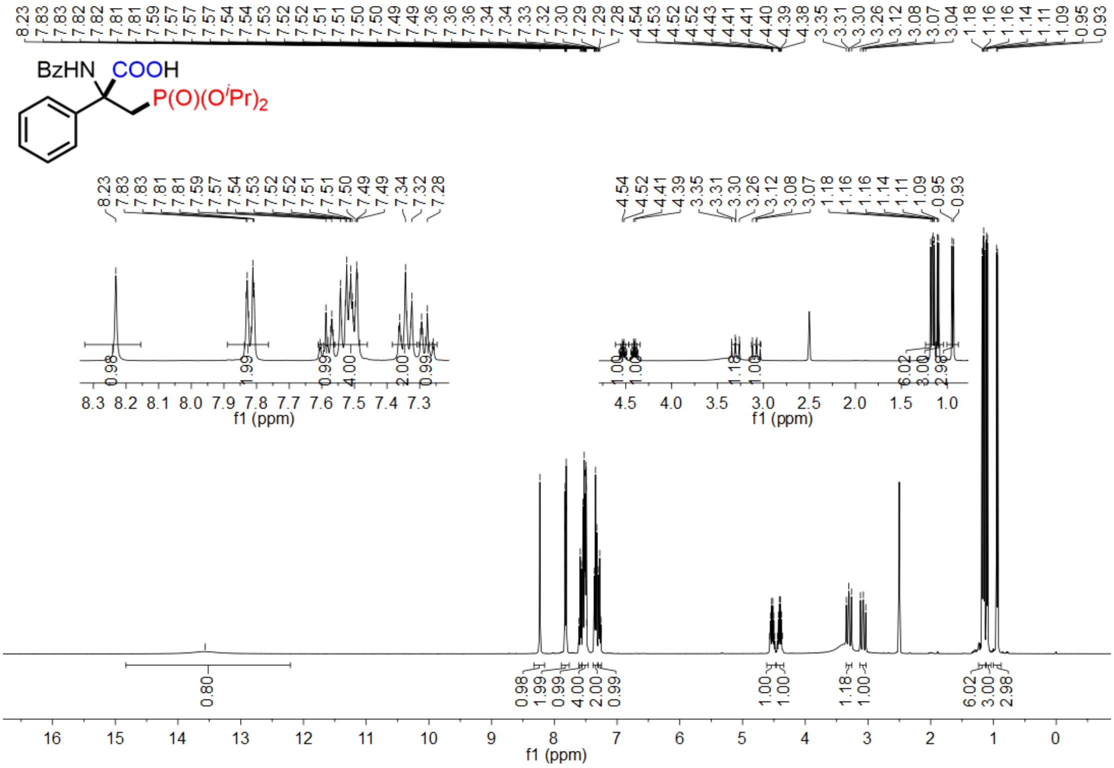

Supplementary Figure 119. ^{1}H NMR spectra of **3ah**

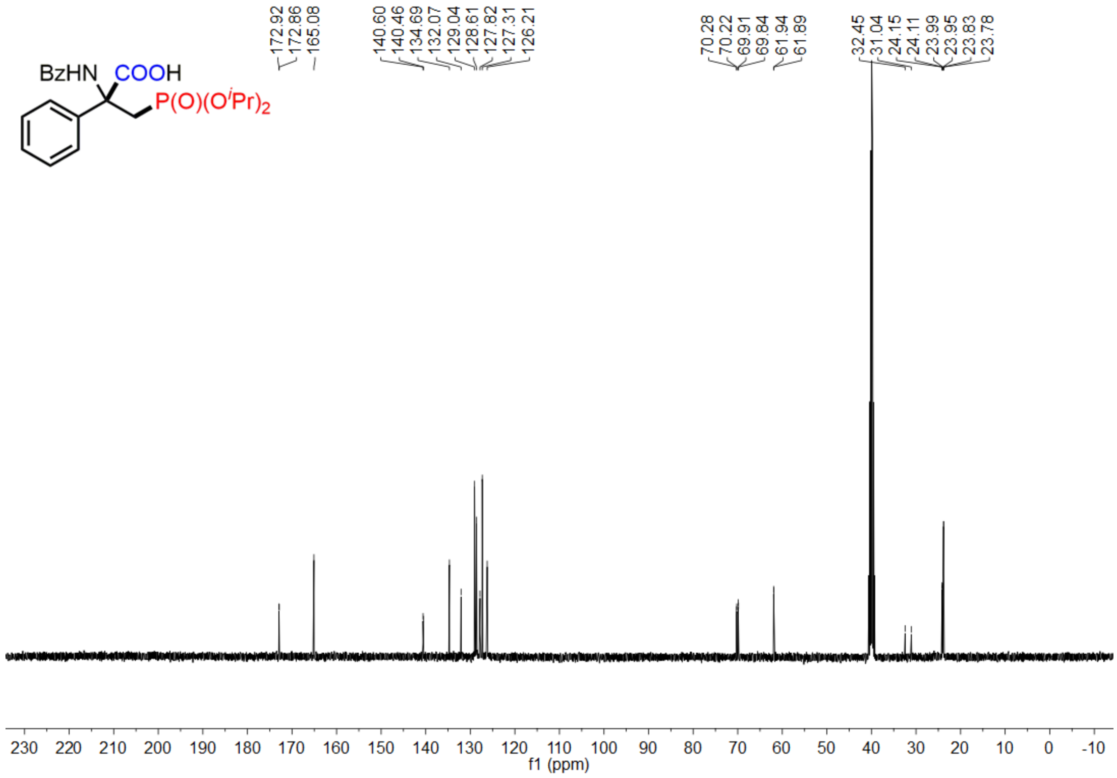

Supplementary Figure 120. ^{13}C NMR spectra of **3ah**

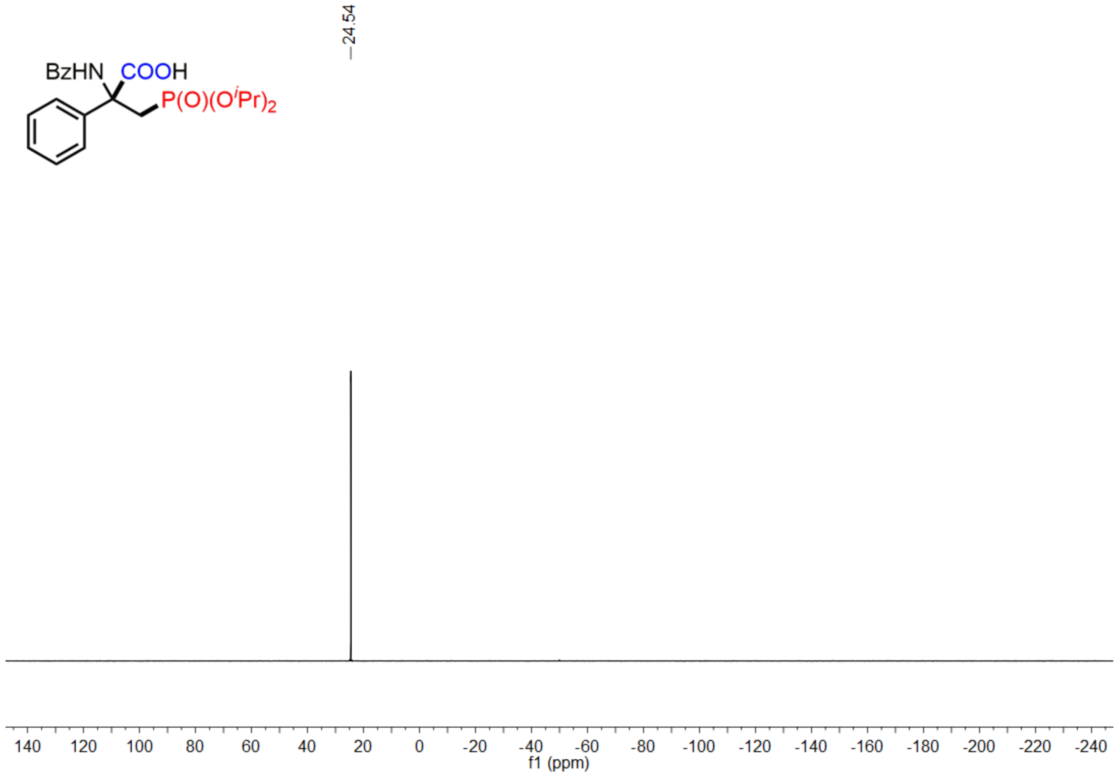

Supplementary Figure 121. ^{31}P NMR spectra of **3ah**

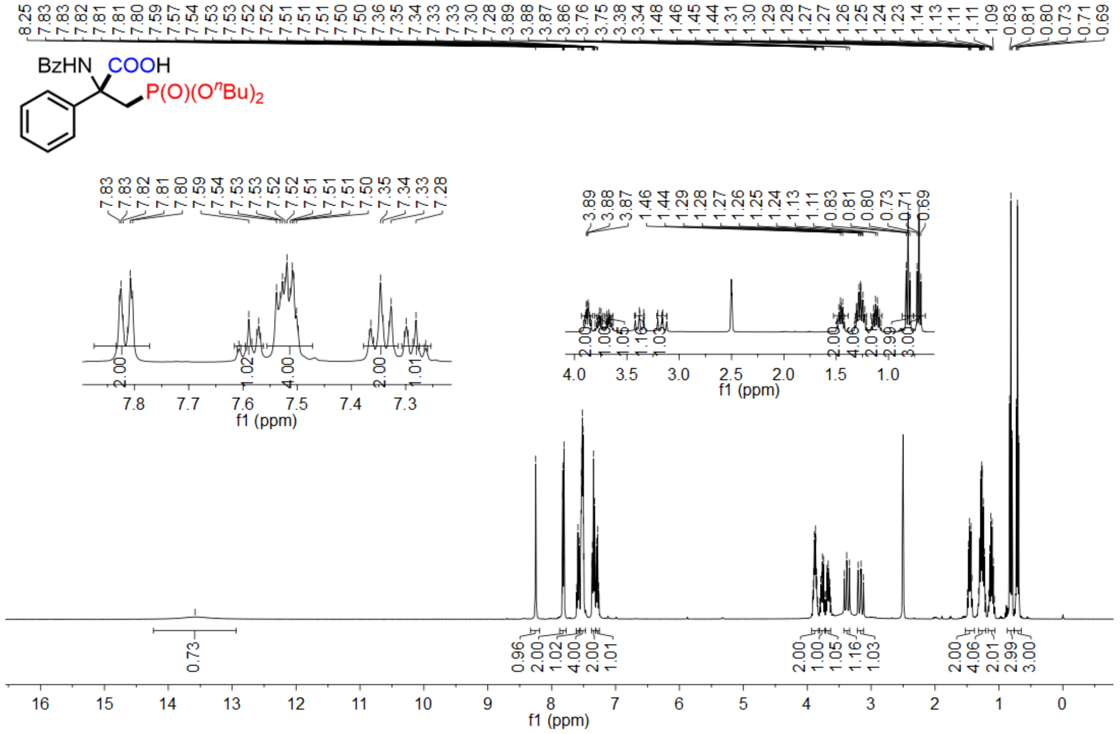

Supplementary Figure 122. ^{1}H NMR spectra of **3ai**

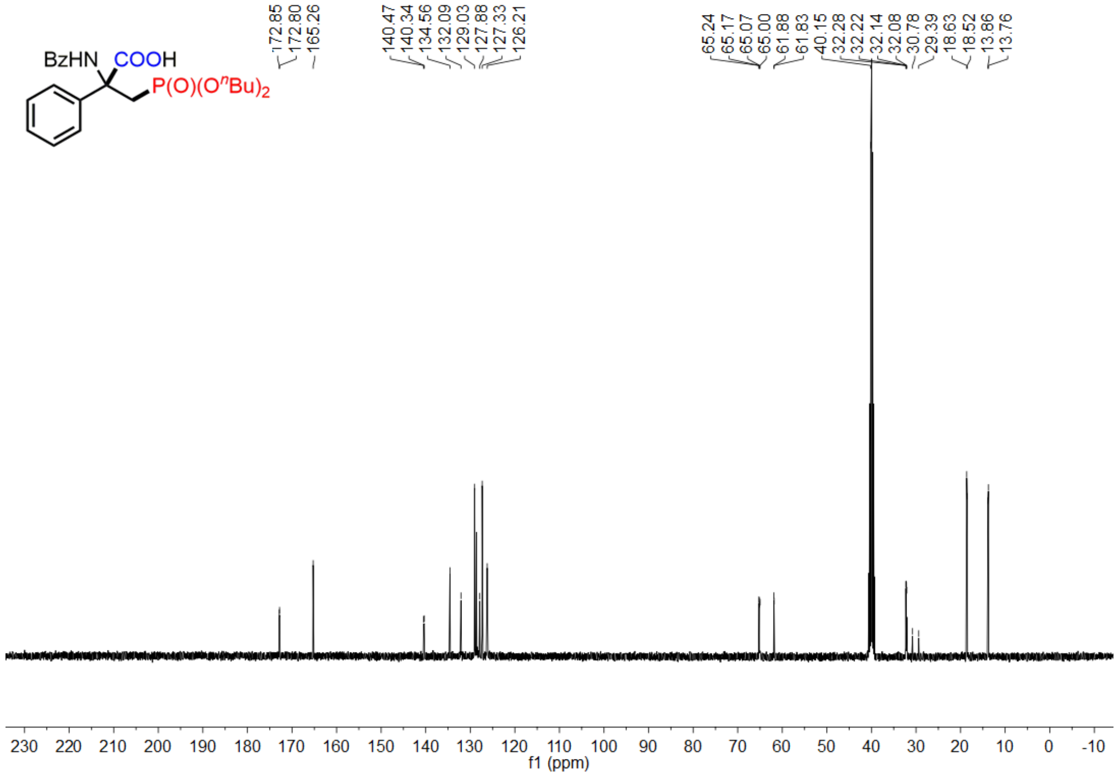

Supplementary Figure 123. ^{13}C NMR spectra of **3ai**

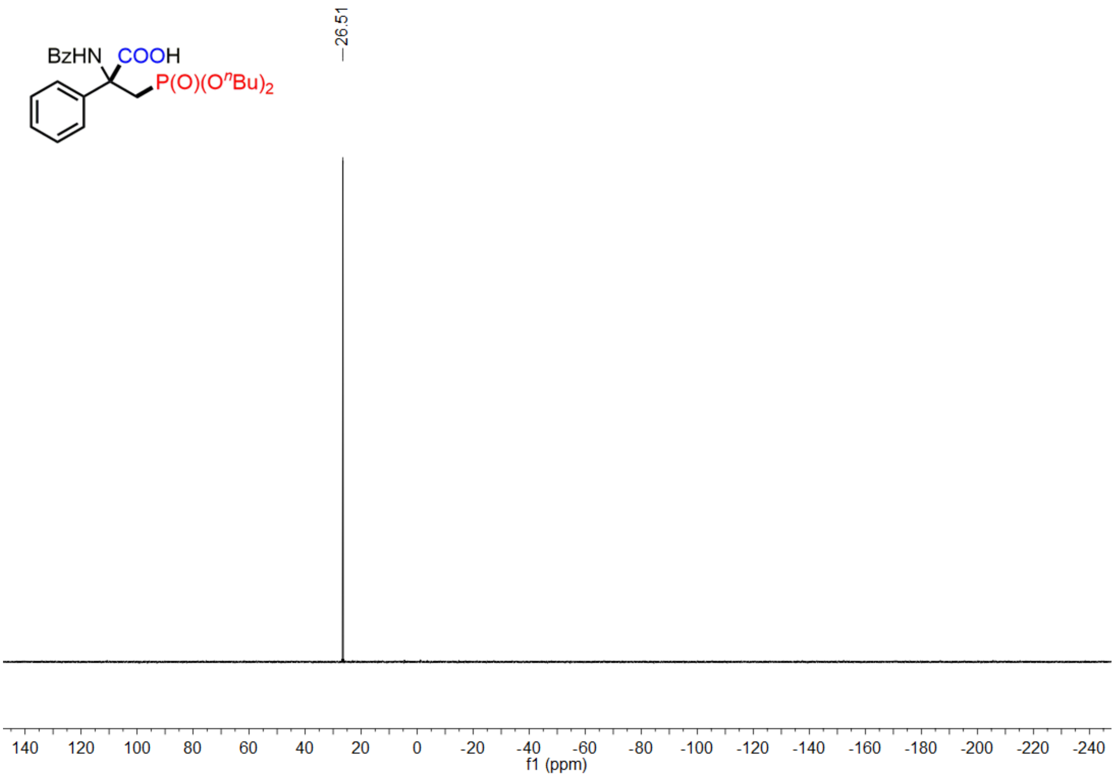

Supplementary Figure 124. ^{31}P NMR spectra of **3ai**

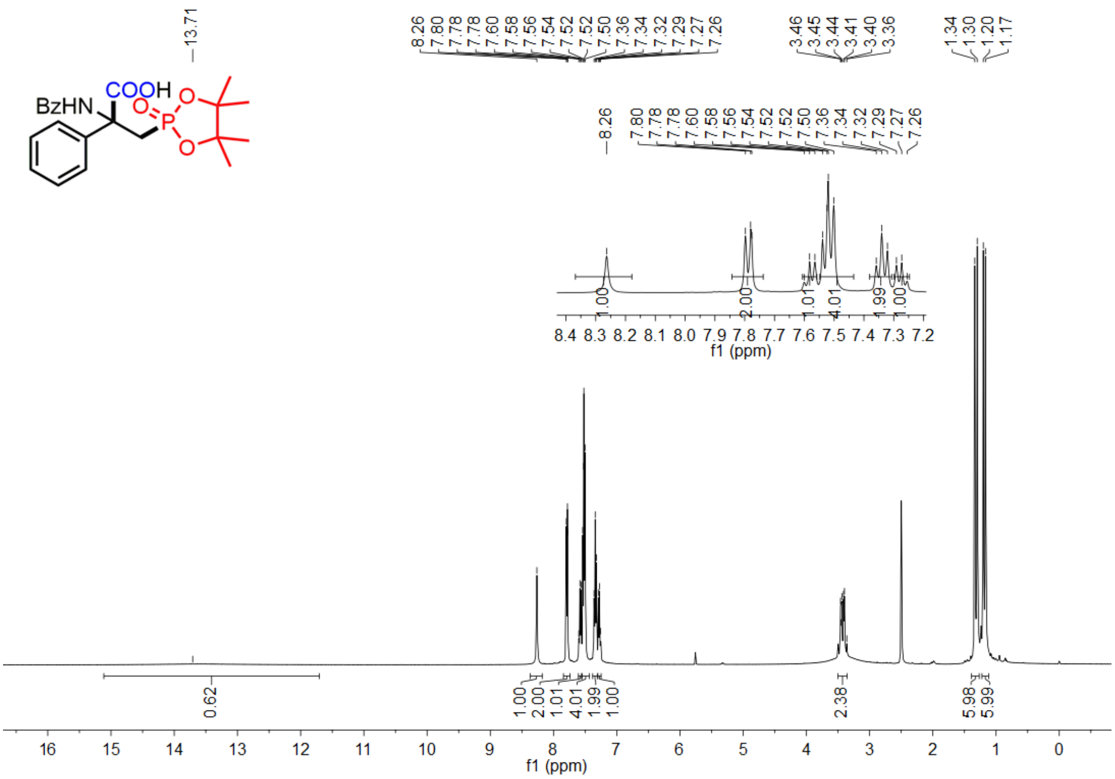

Supplementary Figure 125. ^{1}H NMR spectra of **3ak**

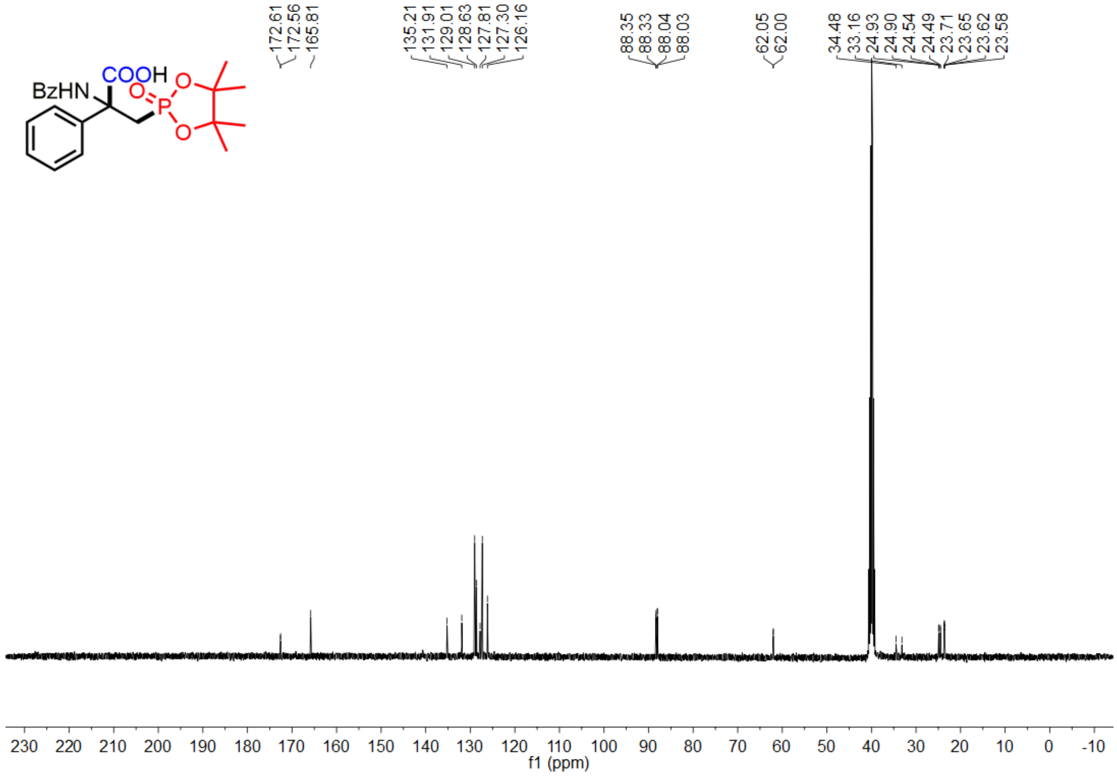

Supplementary Figure 126. ^{13}C NMR spectra of **3ak**

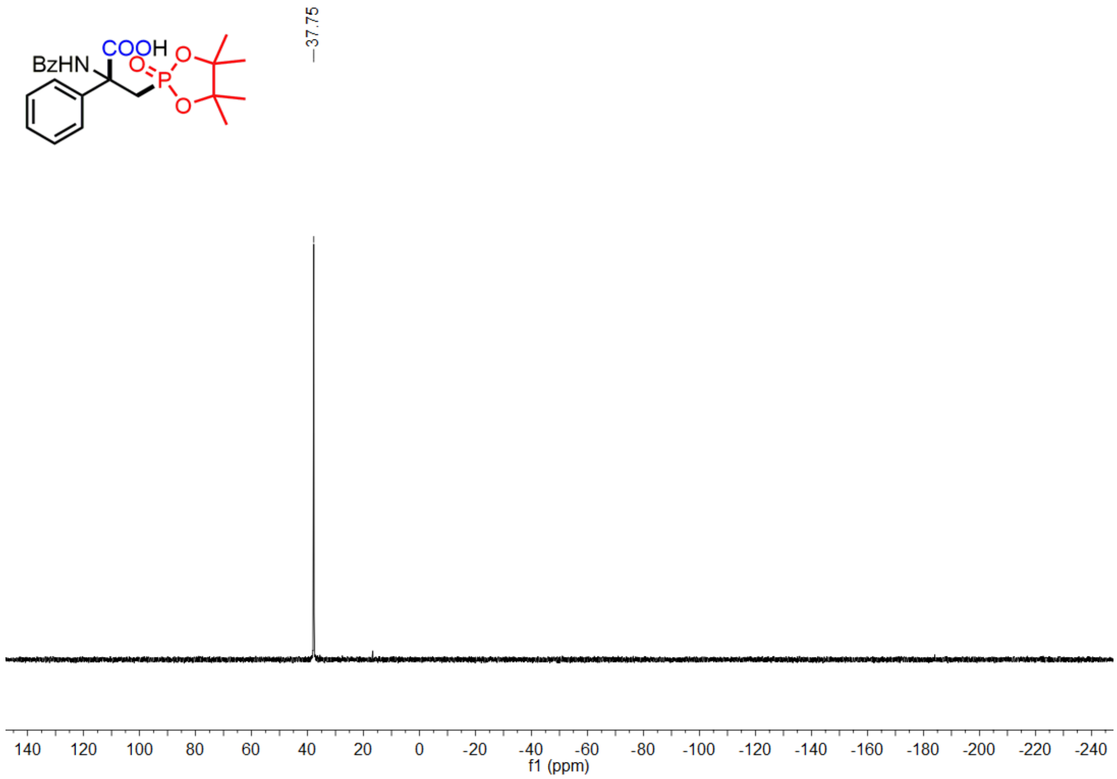

Supplementary Figure 127. ^{31}P NMR spectra of **3ak**

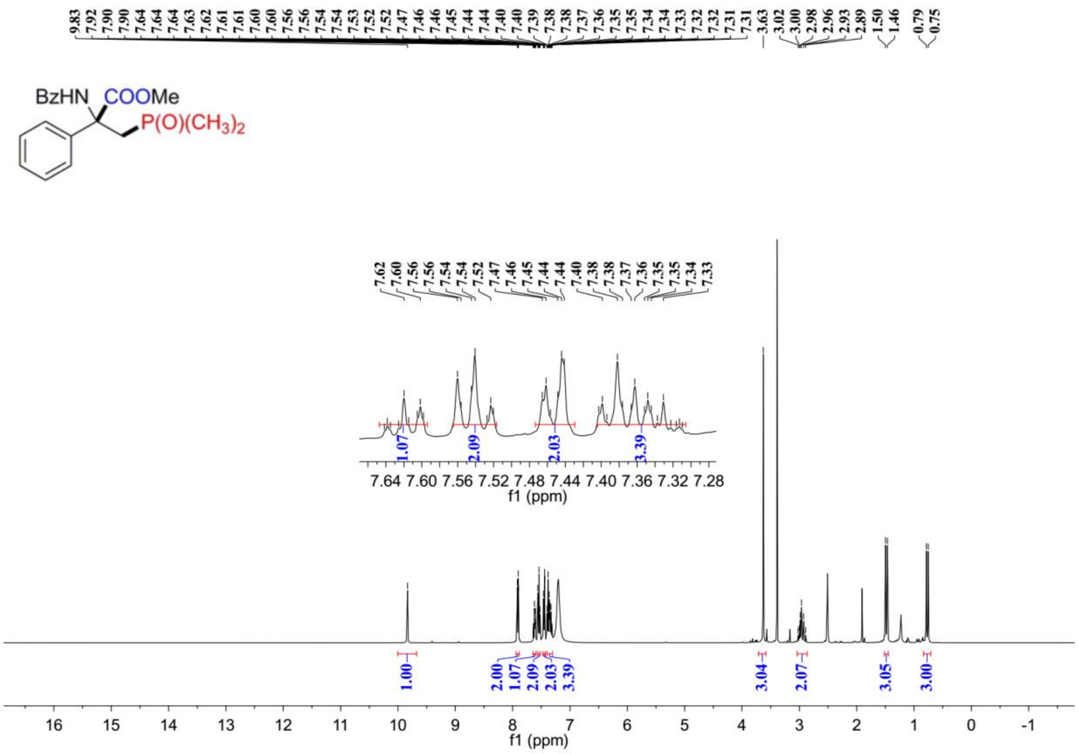

Supplementary Figure 128. ^{1}H NMR spectra of **3al**

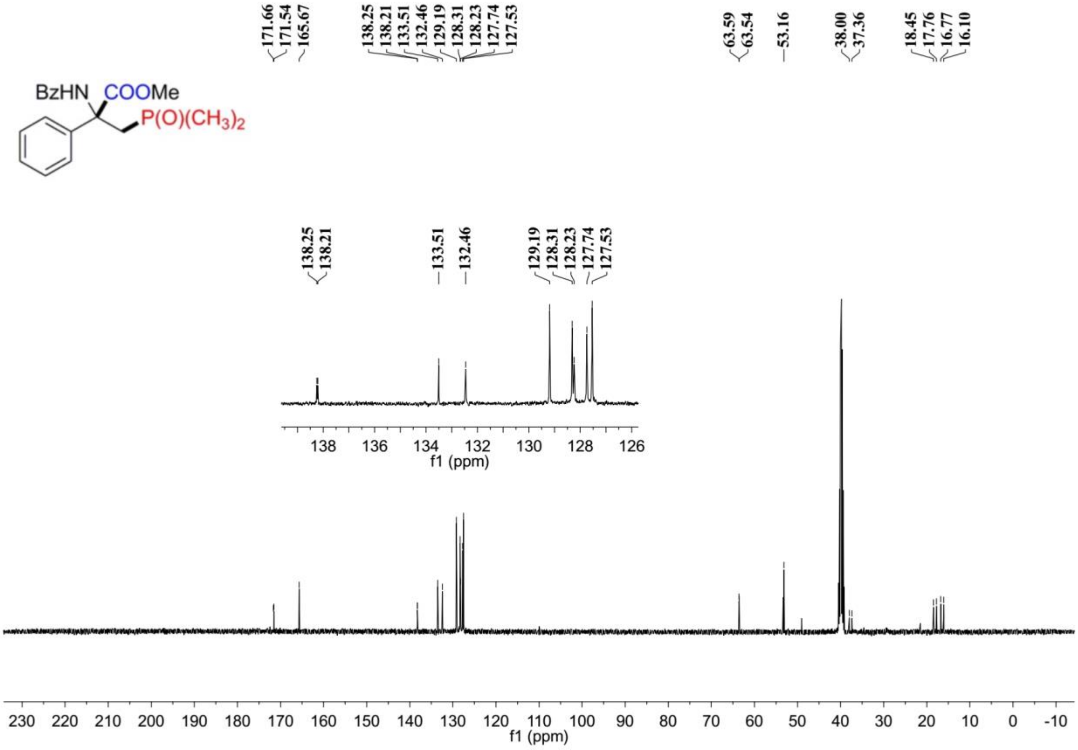

Supplementary Figure 129. ^{13}C NMR spectra of **3al**

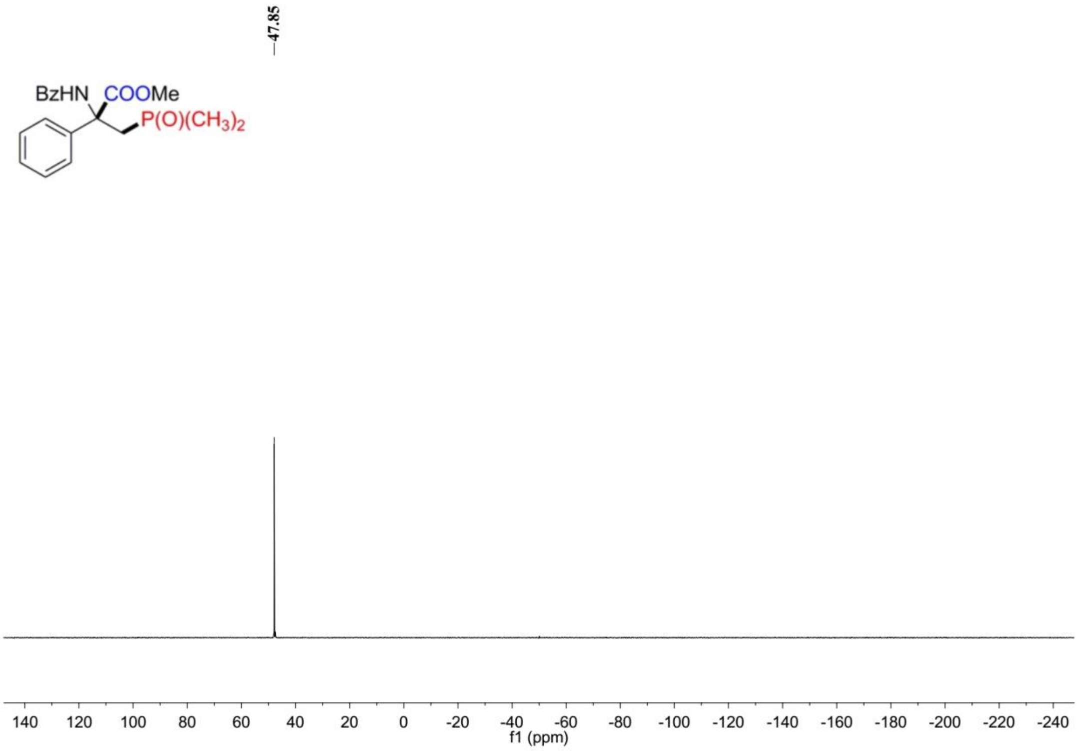

Supplementary Figure 130. ^{31}P NMR spectra of **3al**

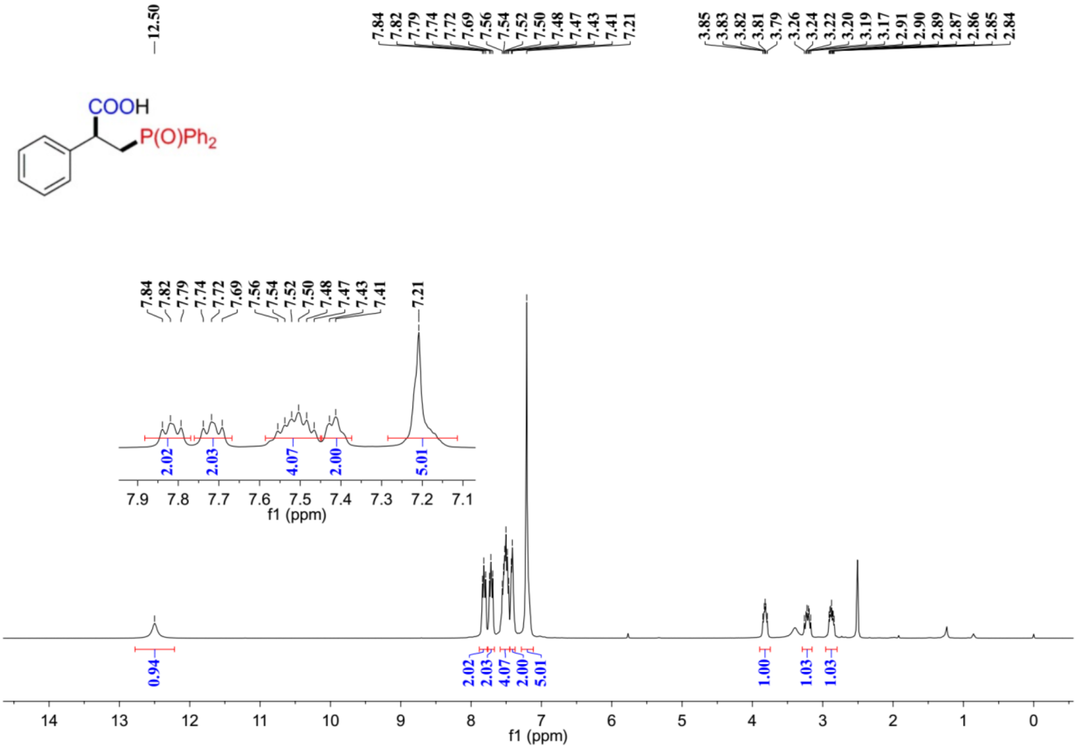

Supplementary Figure 131. ^{1}H NMR spectra of **9aa**

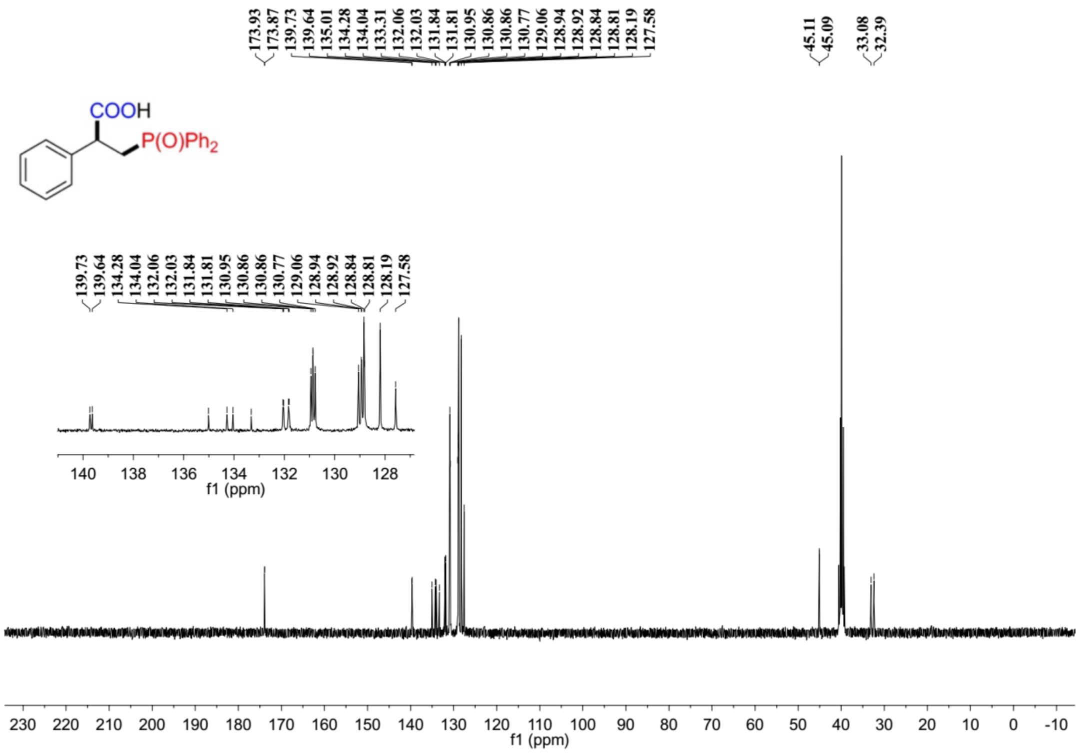

Supplementary Figure 132. ^{13}C NMR spectra of **9aa**

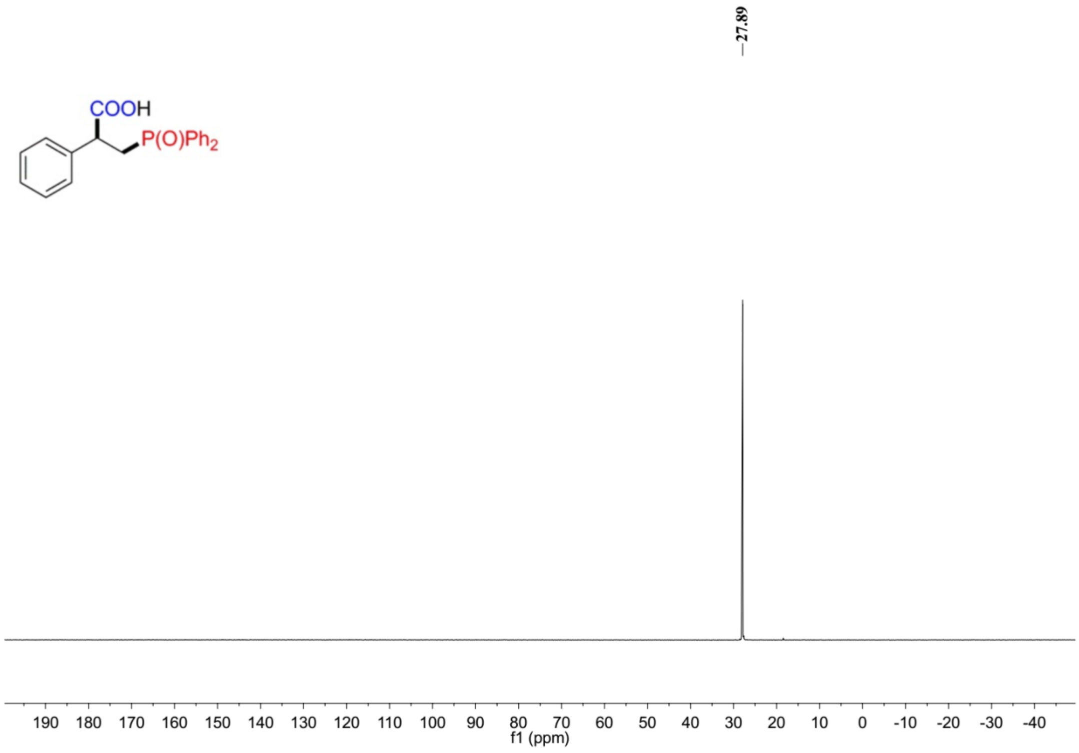

Supplementary Figure 133. ^{31}P NMR spectra of **9aa**

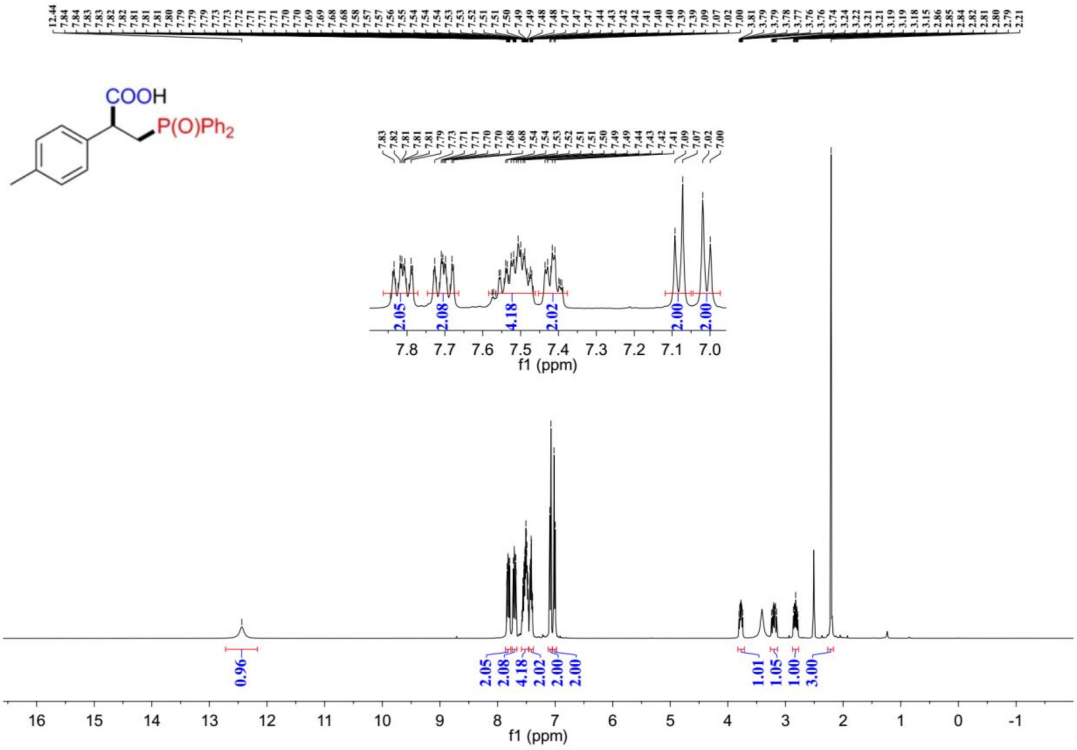

Supplementary Figure 134. ^{1}H NMR spectra of **9ba**

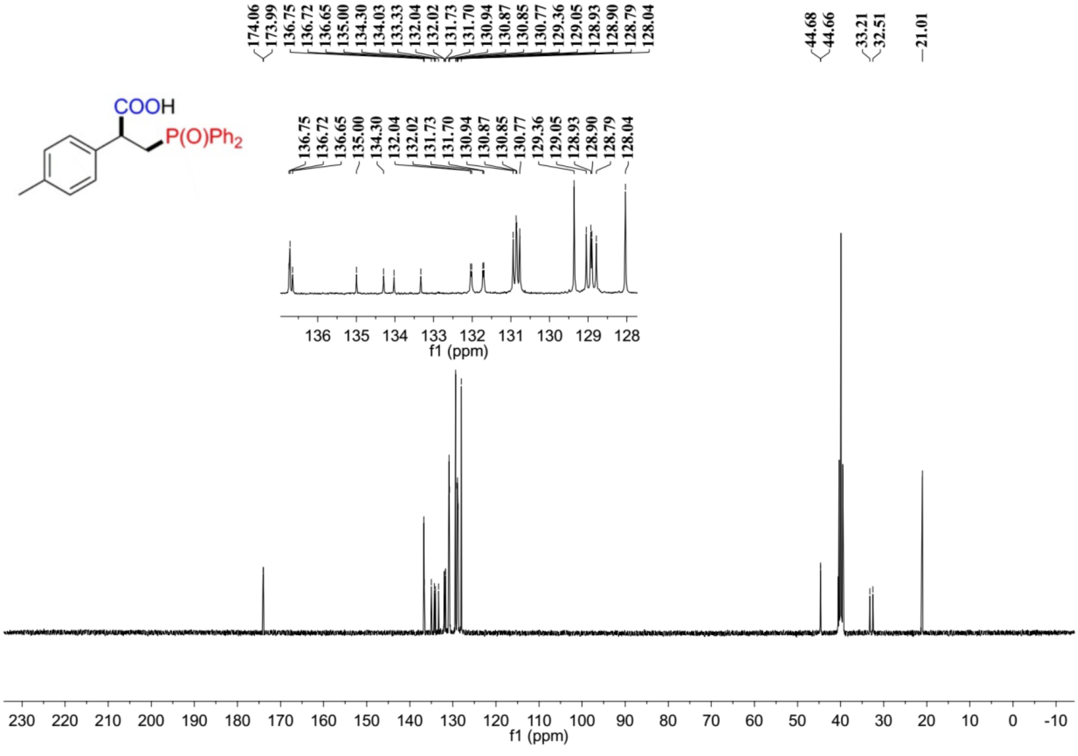

Supplementary Figure 135. ^{13}C NMR spectra of **9ba**

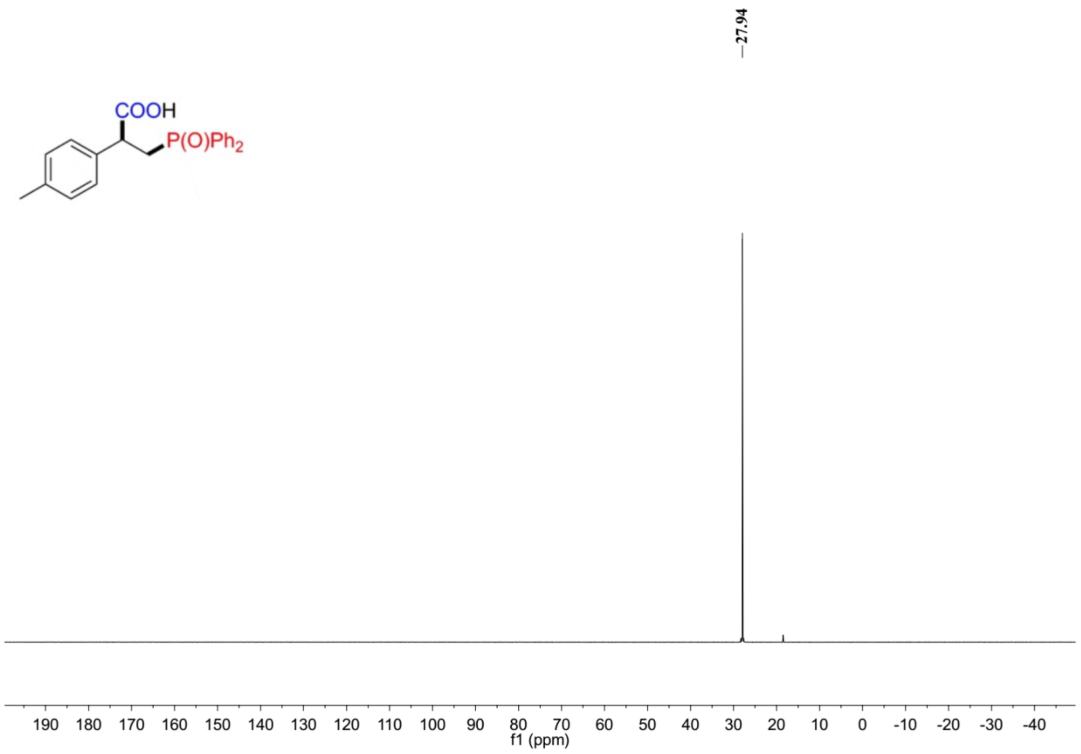

Supplementary Figure 136. ^{31}P NMR spectra of **9ba**

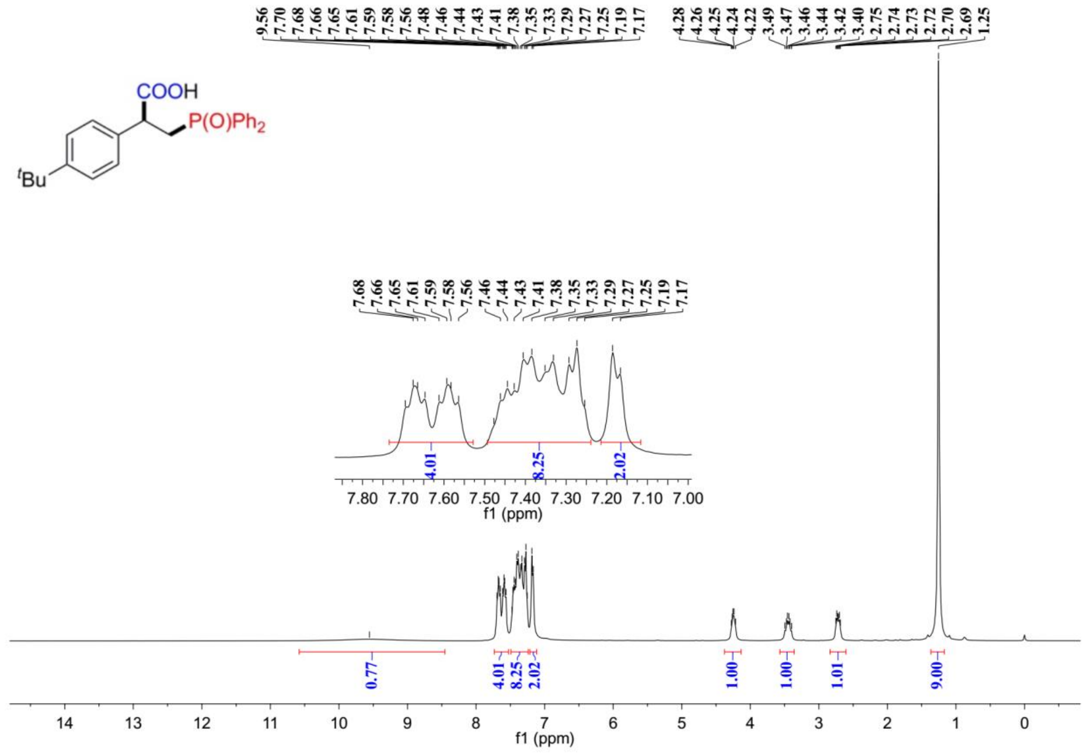

Supplementary Figure 137. ^{1}H NMR spectra of **9ca**

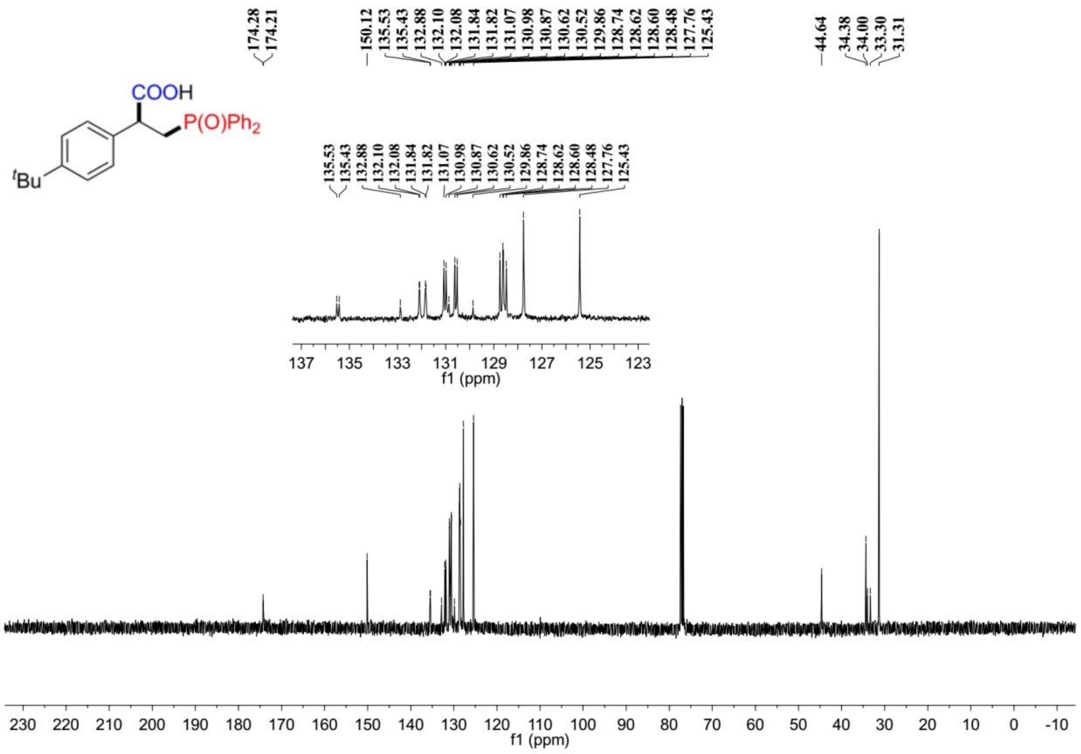

Supplementary Figure 138. ^{13}C NMR spectra of **9ca**

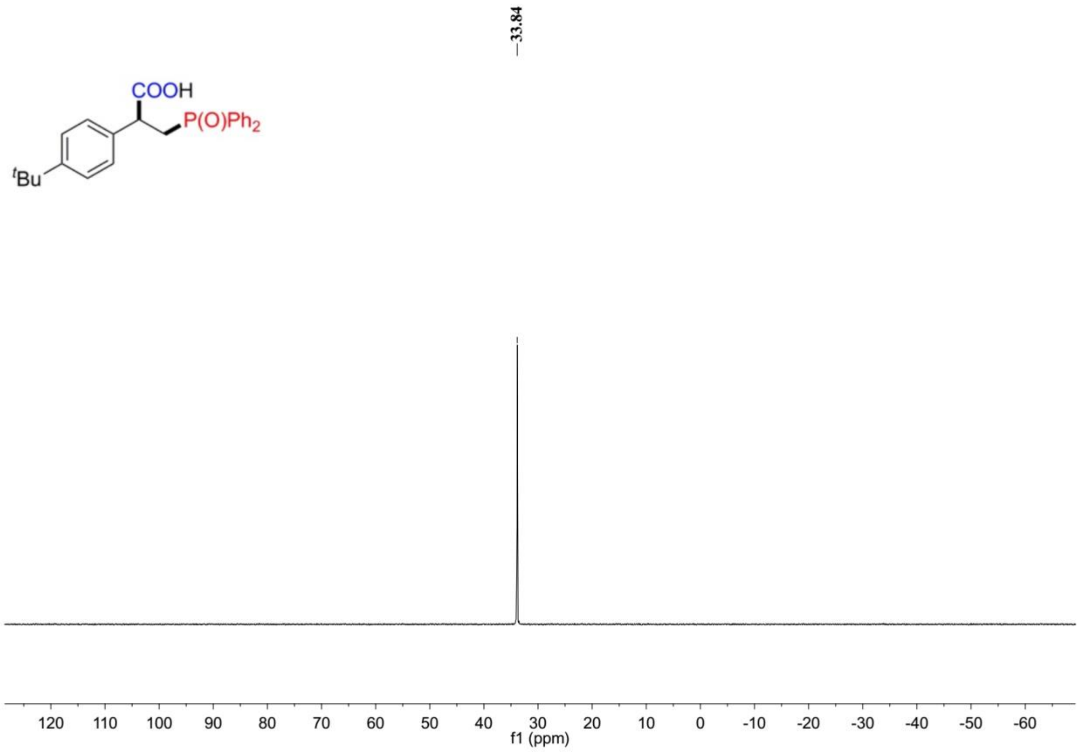

Supplementary Figure 139. ^{31}P NMR spectra of **9ca**

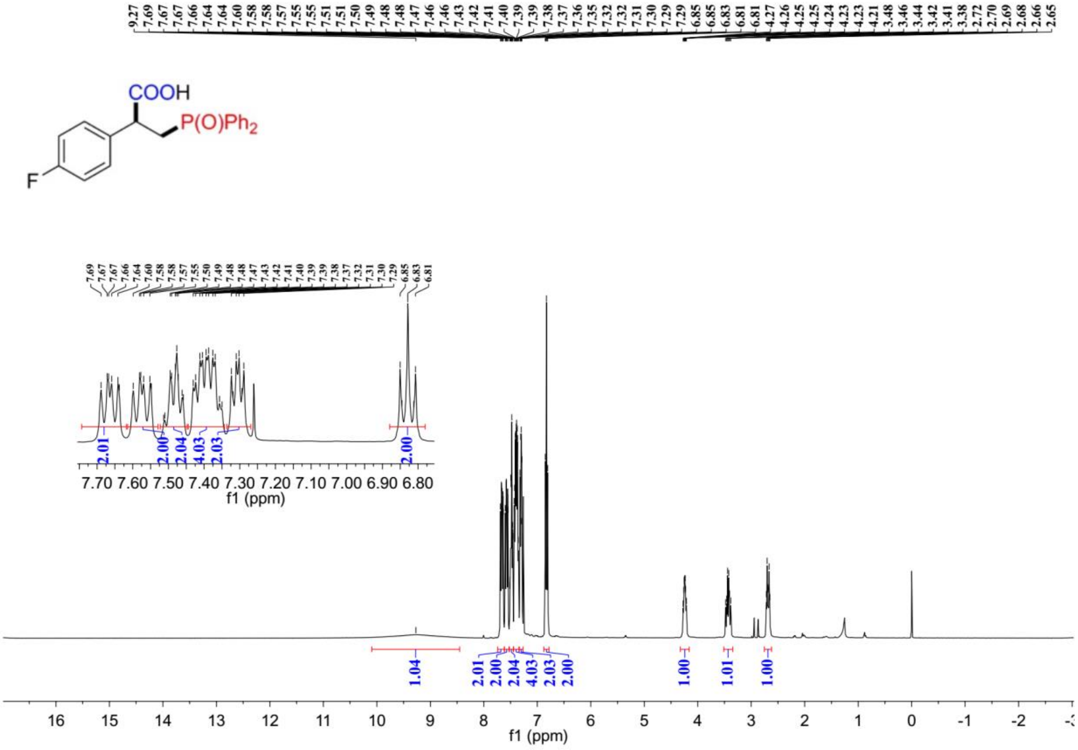

Supplementary Figure 140. ^{1}H NMR spectra of **9da**

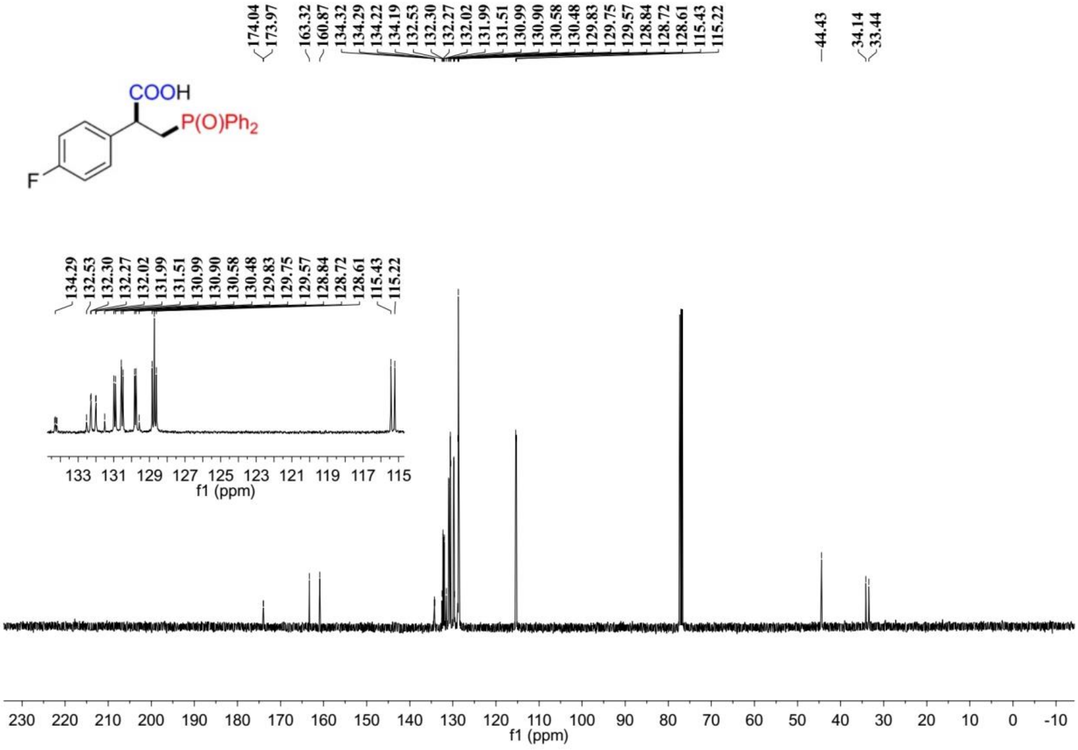

Supplementary Figure 141. ^{13}C NMR spectra of **9da**

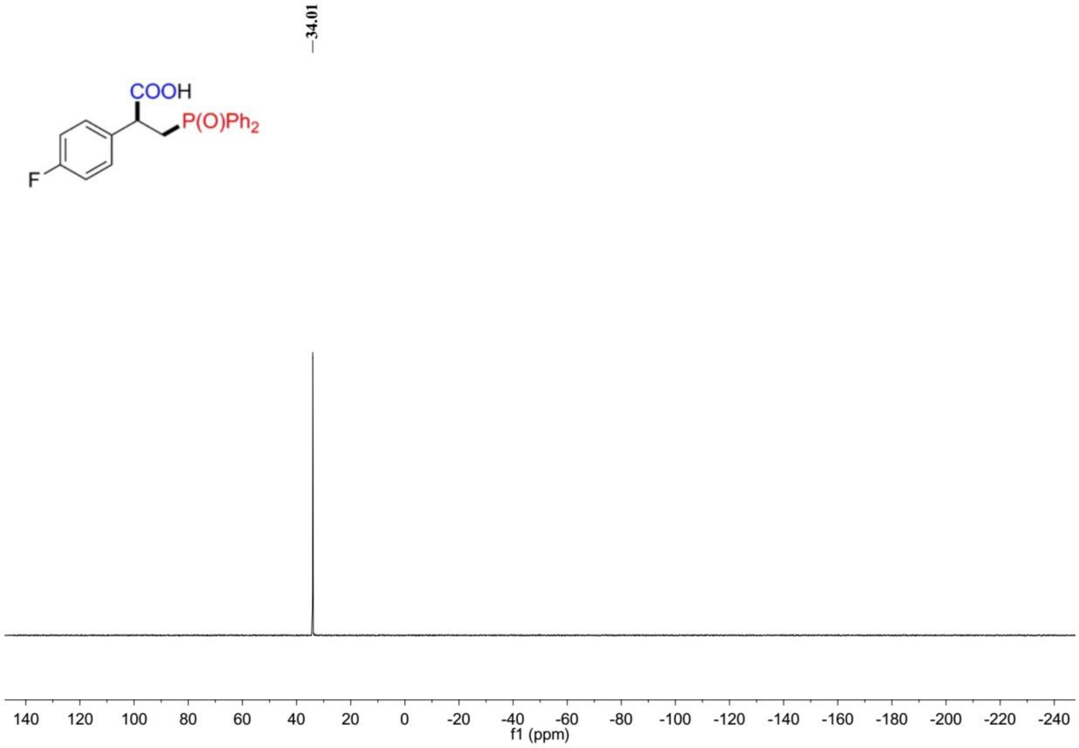

Supplementary Figure 142. ^{31}P NMR spectra of **9da**

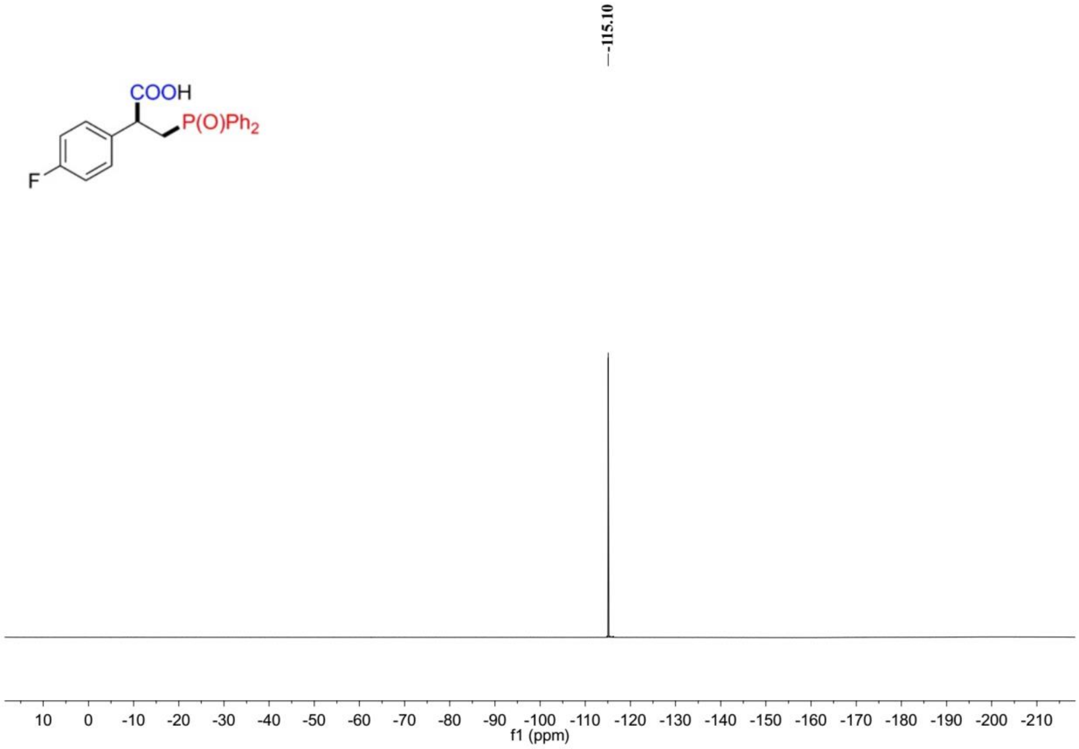

Supplementary Figure 143. ^{19}F NMR spectra of **9da**

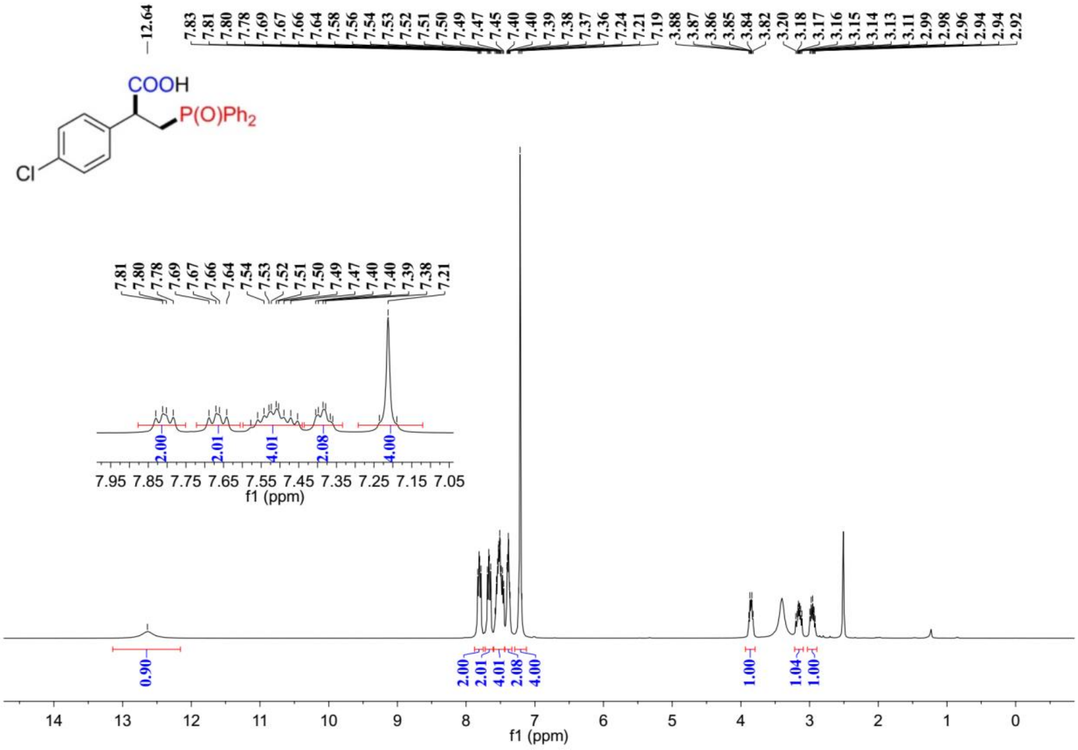

Supplementary Figure 144. ^{1}H NMR spectra of **9ea**

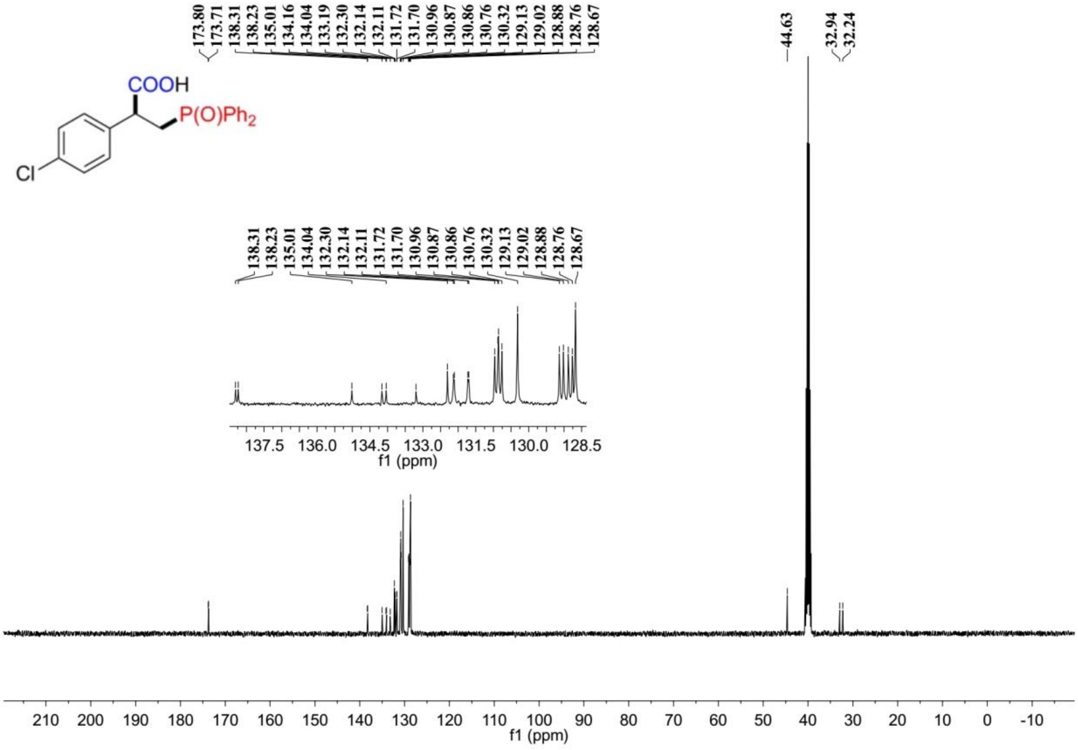

Supplementary Figure 145. ^{13}C NMR spectra of **9ea**

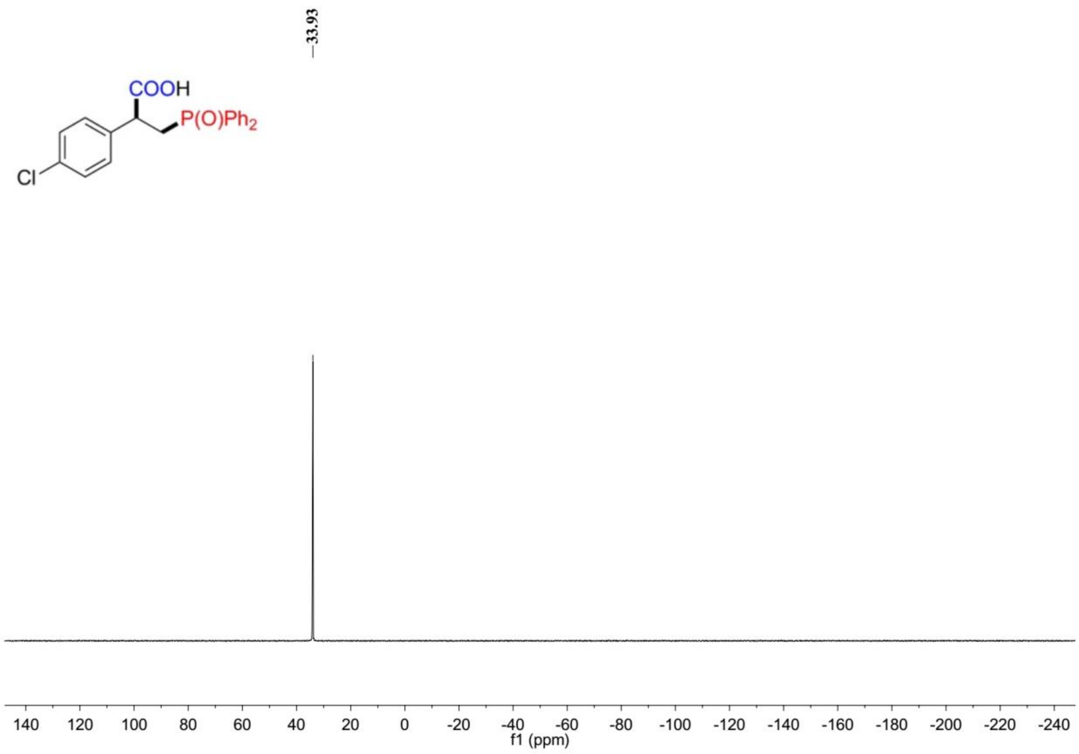

Supplementary Figure 146. ^{31}P NMR spectra of **9ea**

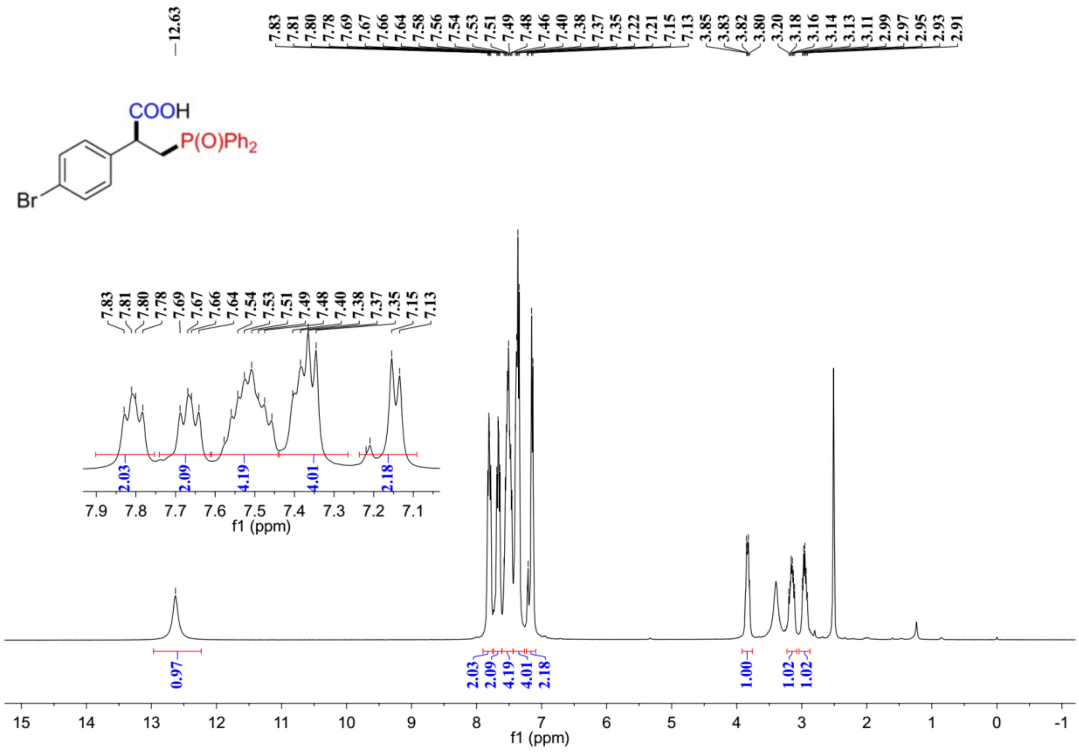

Supplementary Figure 147. ^{1}H NMR spectra of **9fa**

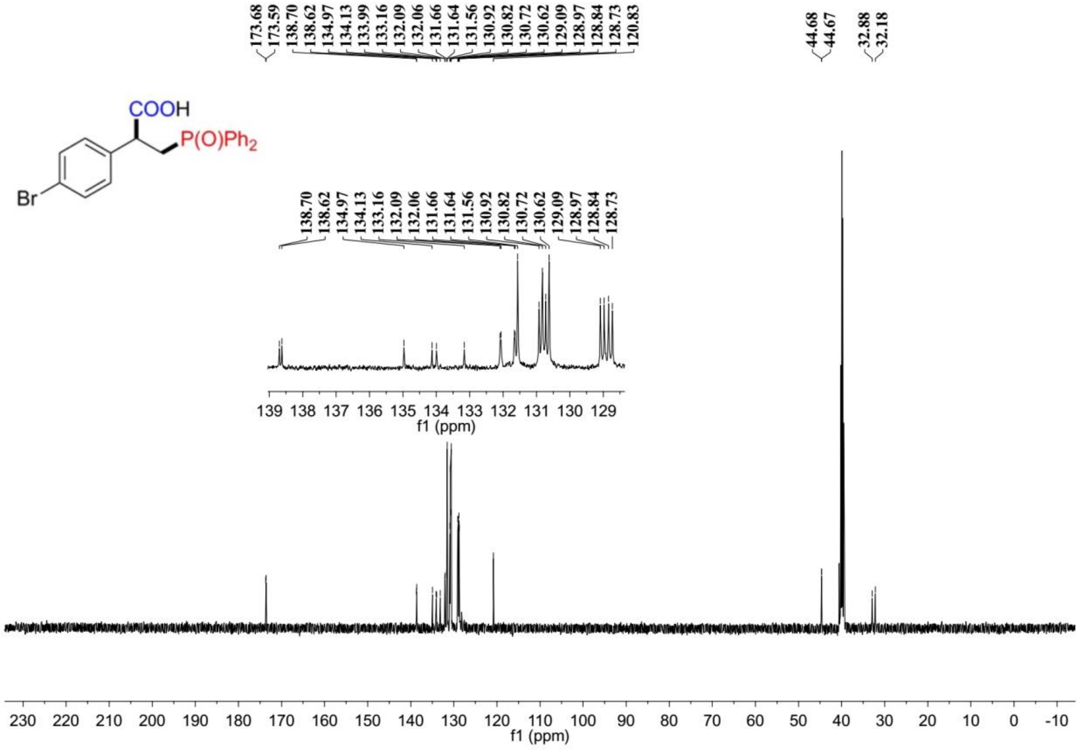

Supplementary Figure 148. ^{13}C NMR spectra of **9fa**

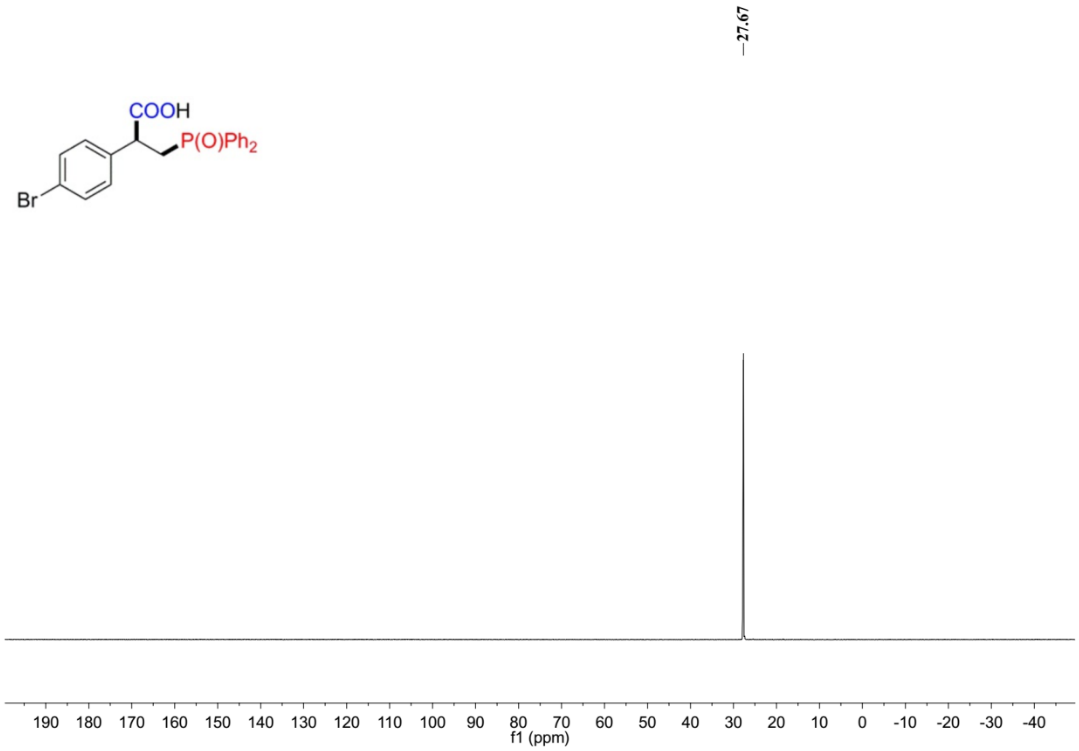

Supplementary Figure 149. ^{31}P NMR spectra of **9fa**

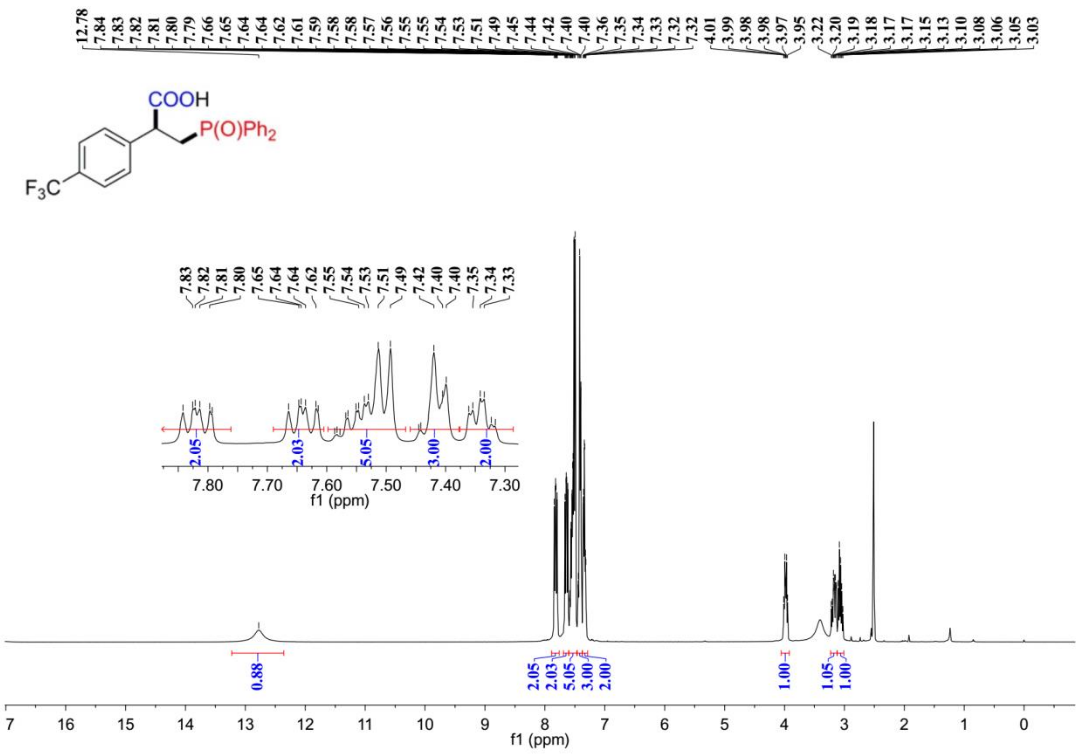

Supplementary Figure 150. ^{1}H NMR spectra of **9ga**

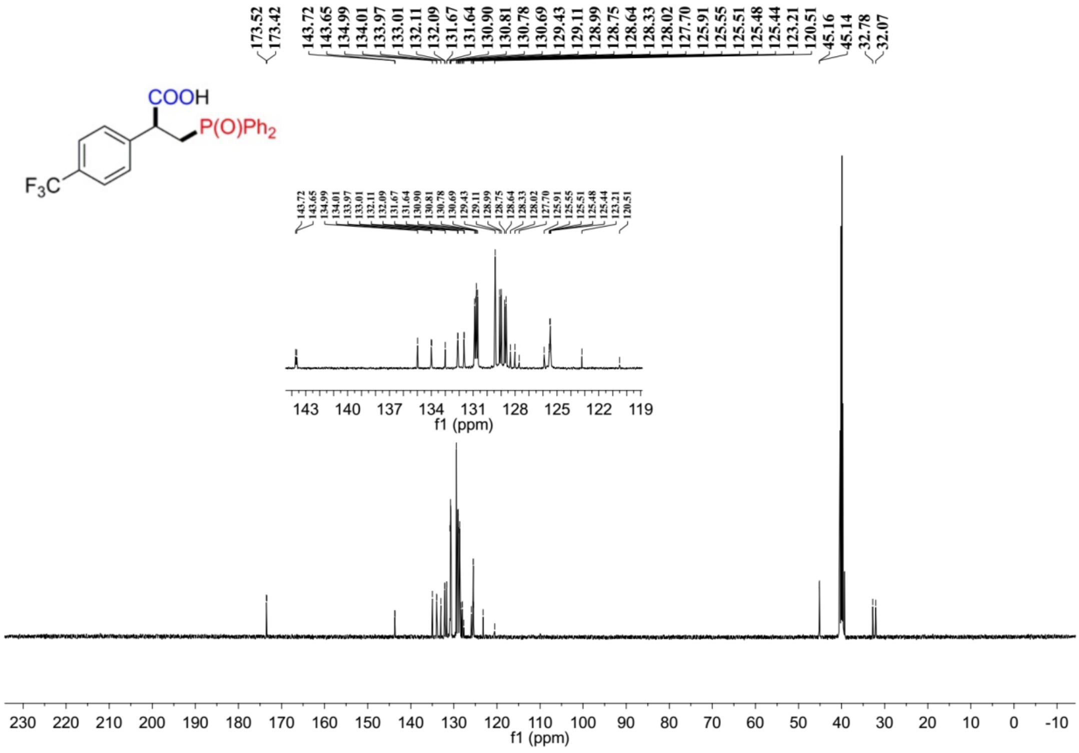

Supplementary Figure 151. ^{13}C NMR spectra of **9ga**

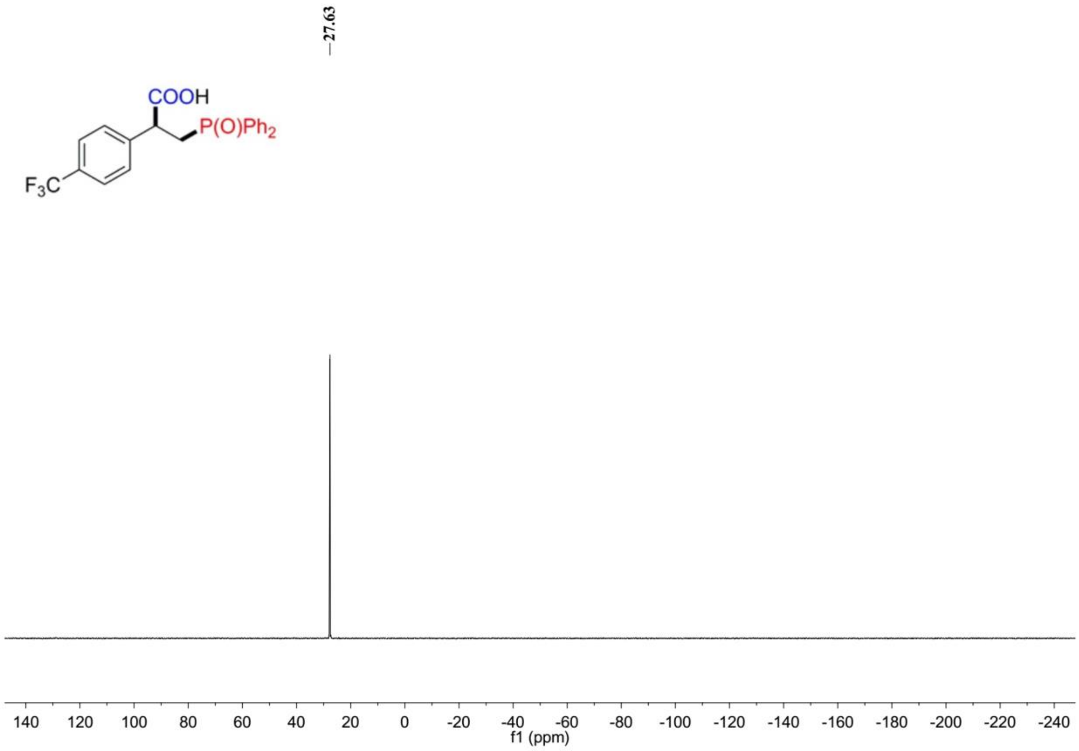

Supplementary Figure 152. ^{31}P NMR spectra of **9ga**

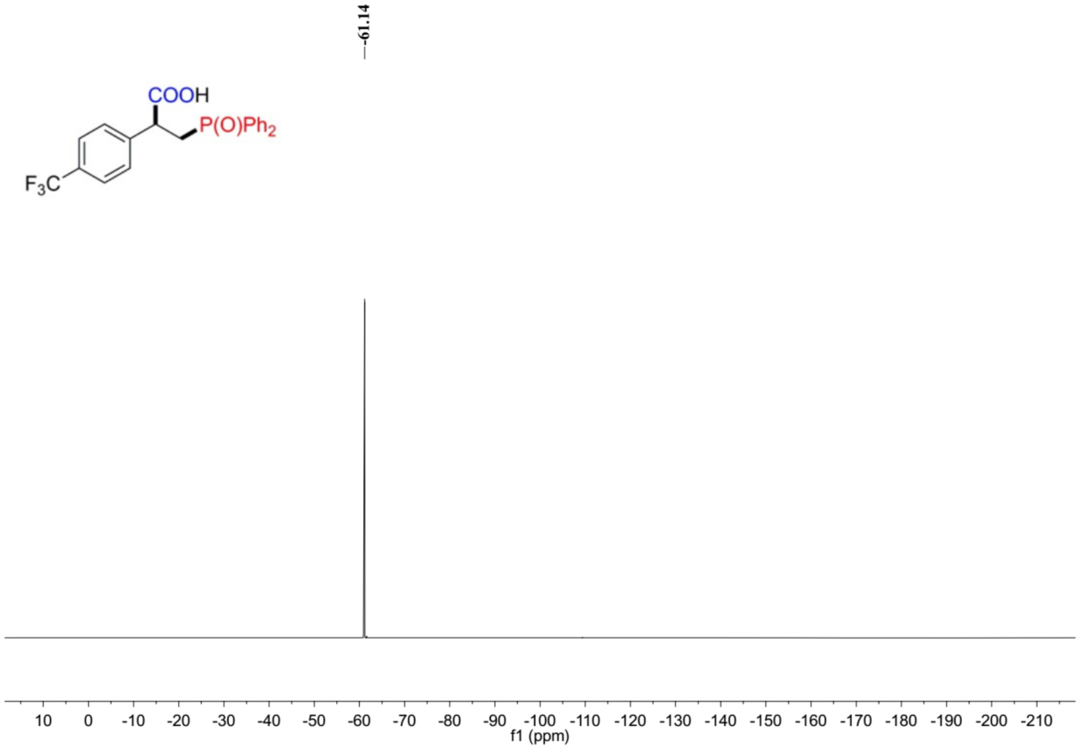

Supplementary Figure 153. ^{19}F NMR spectra of **9ga**

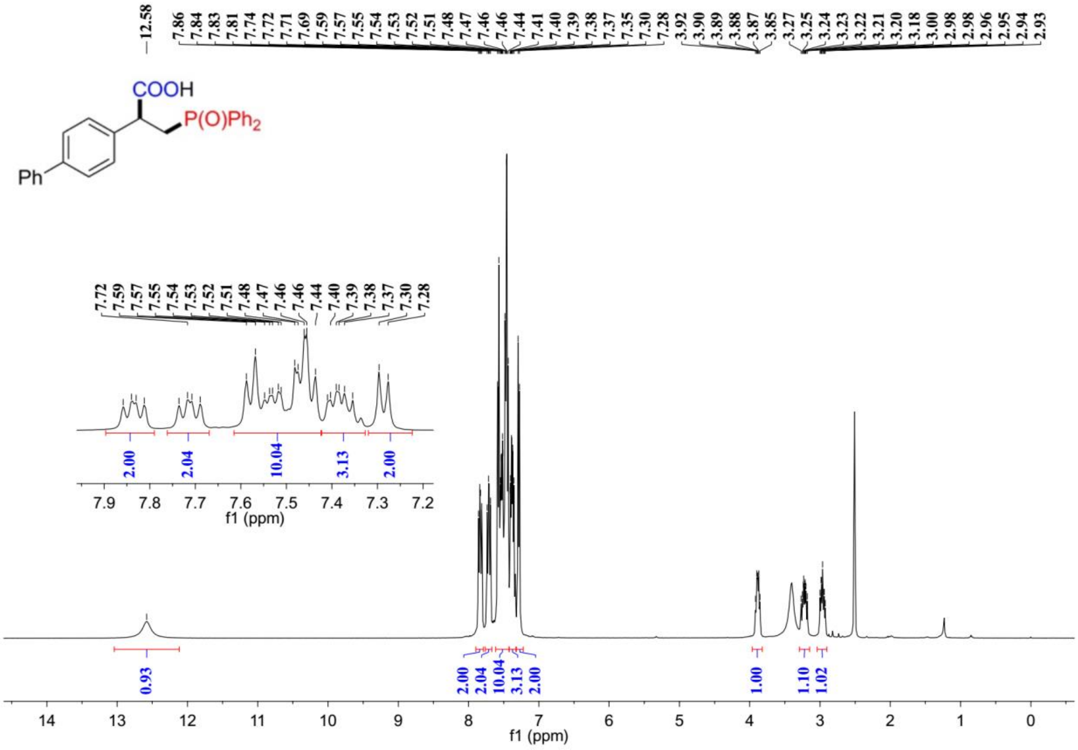

Supplementary Figure 154. ^{1}H NMR spectra of **9ha**

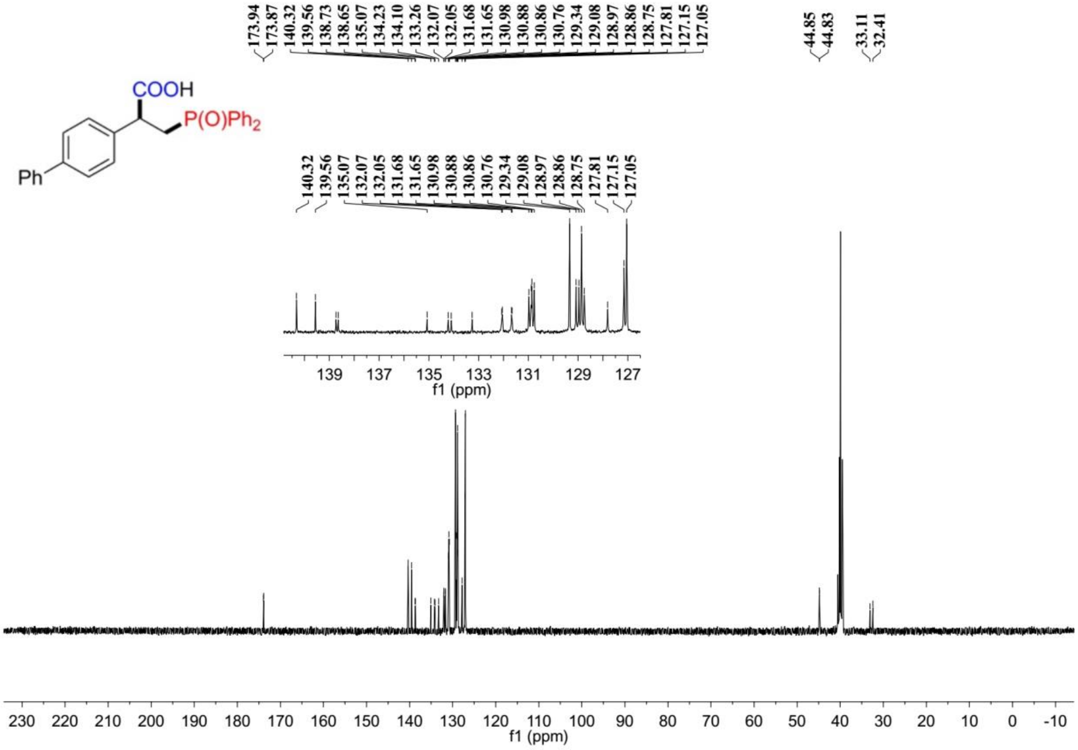

Supplementary Figure 155. ^{13}C NMR spectra of **9ha**

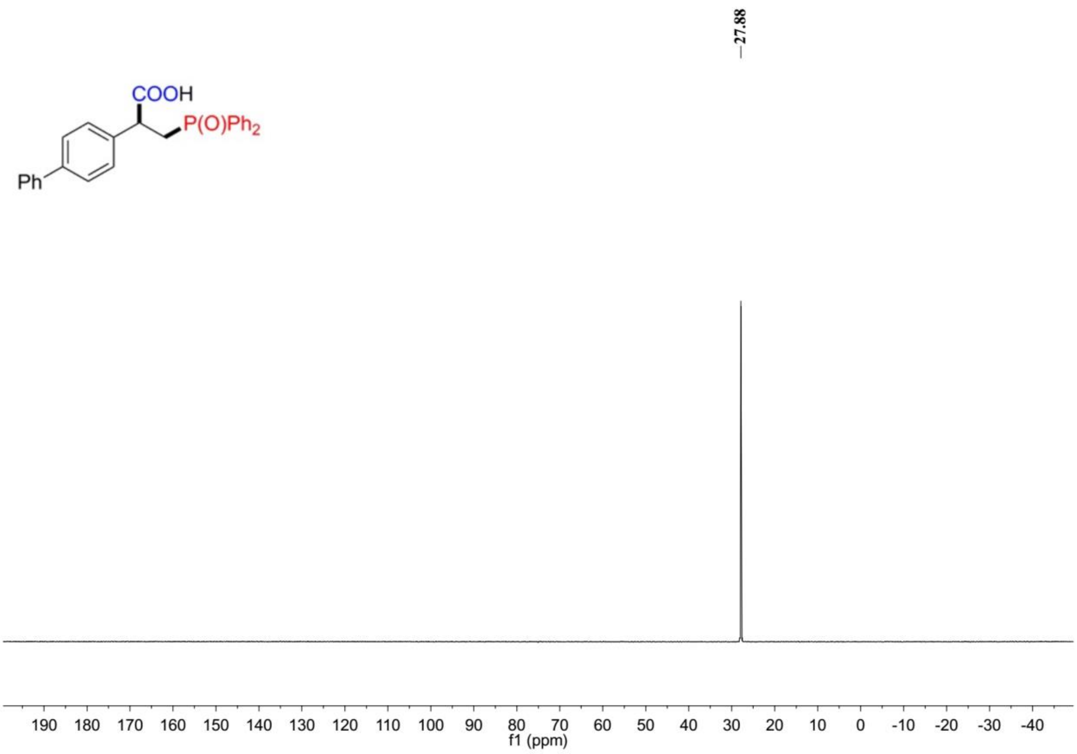

Supplementary Figure 156. ^{31}P NMR spectra of **9ha**

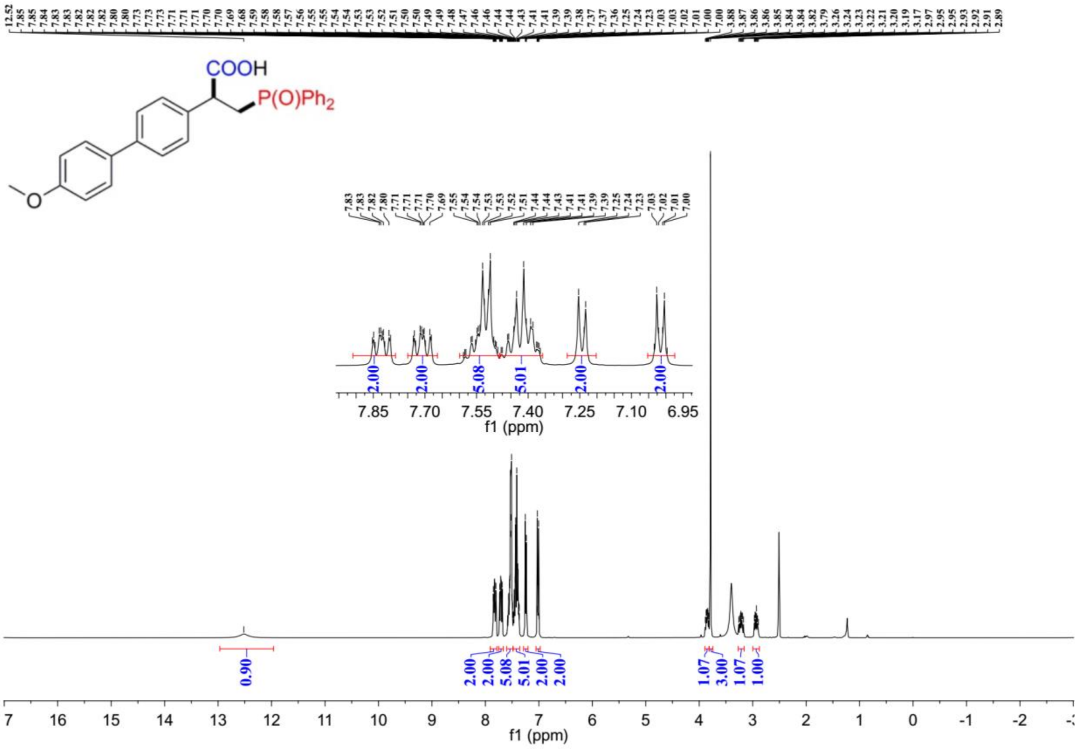

Supplementary Figure 157. ^{1}H NMR spectra of **9ia**

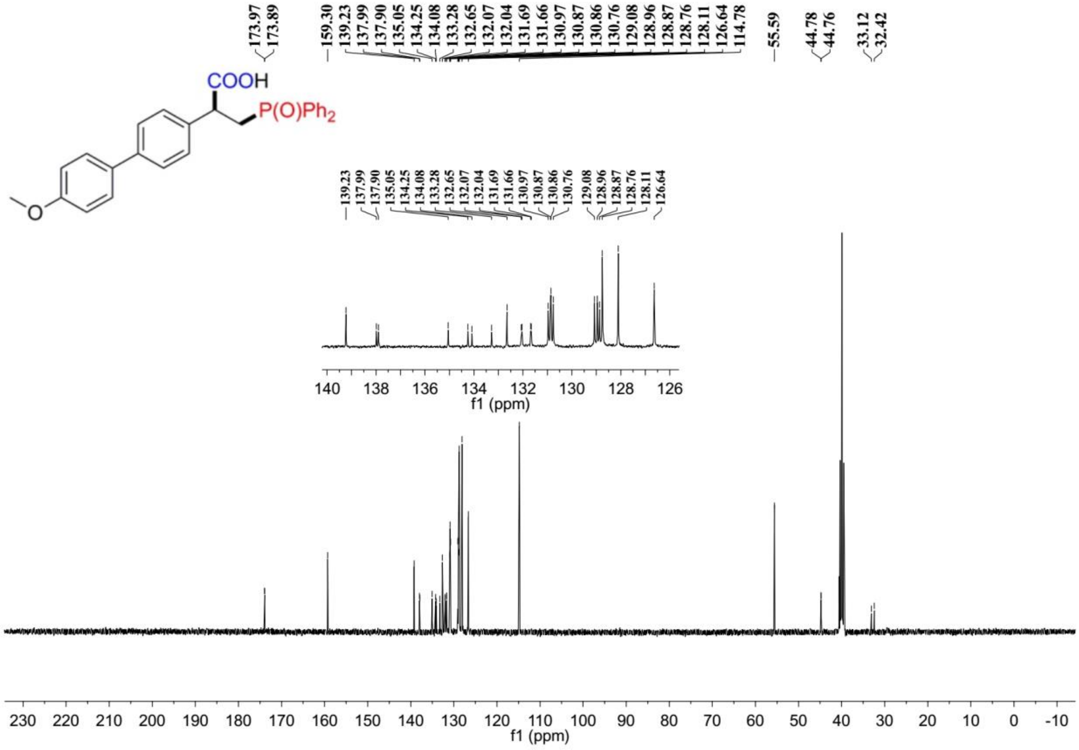

Supplementary Figure 158. ^{13}C NMR spectra of **9ia**

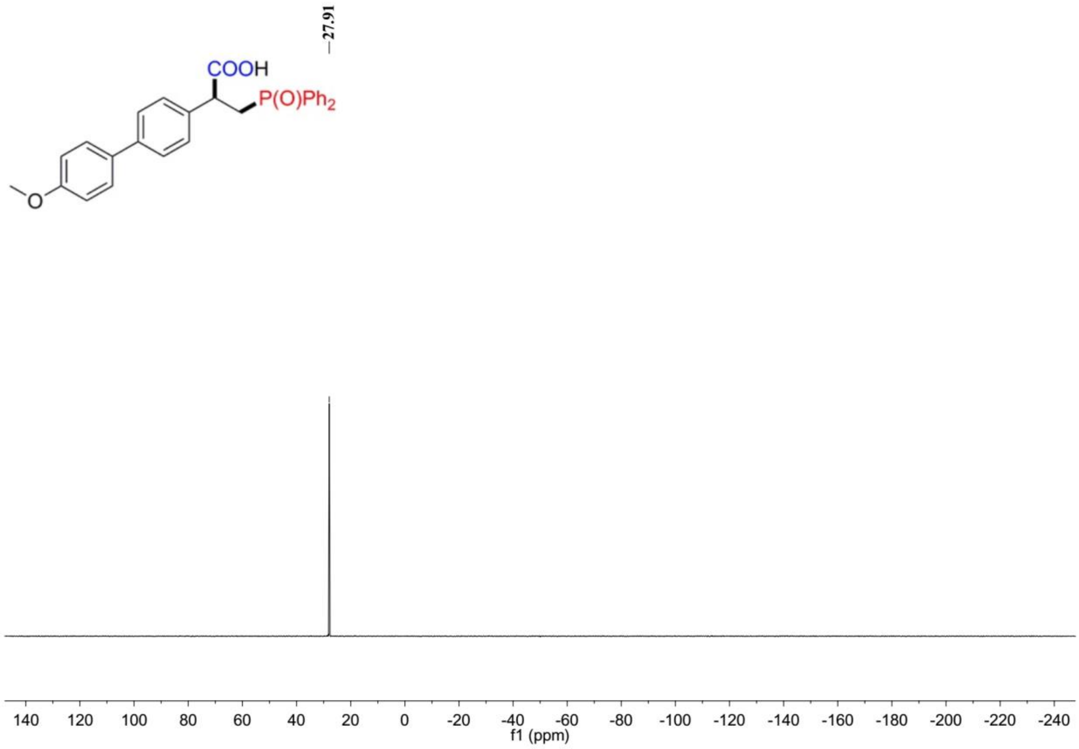

Supplementary Figure 159. ^{31}P NMR spectra of **9ia**

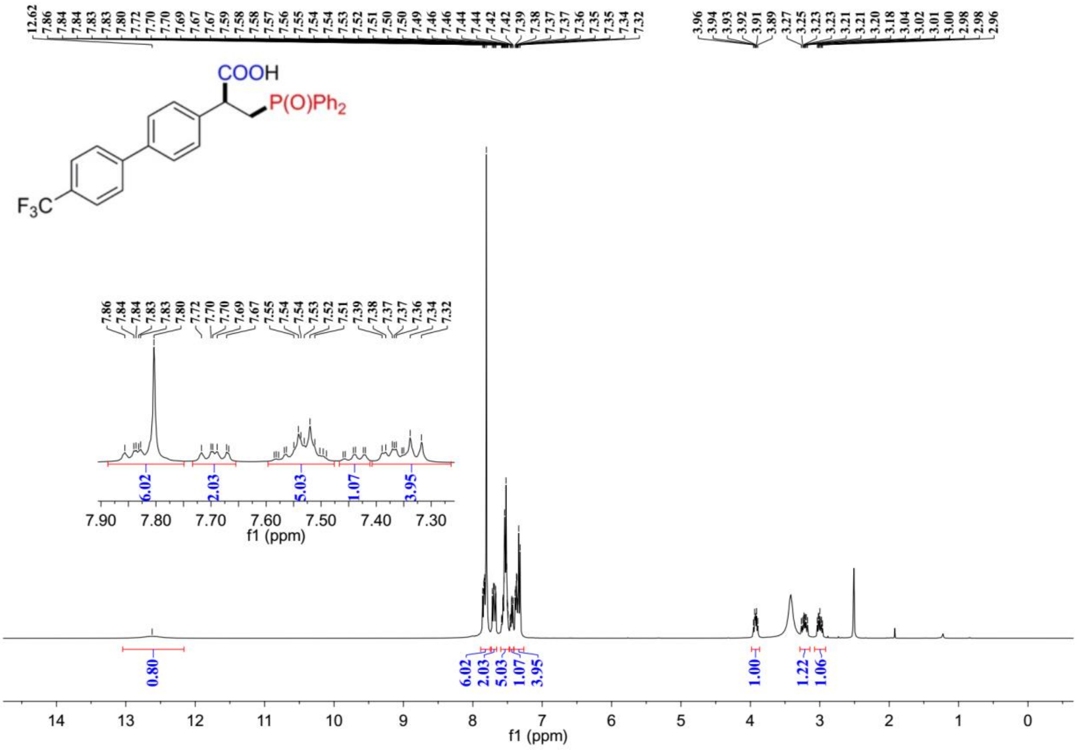

Supplementary Figure 160. ^{1}H NMR spectra of **9ja**

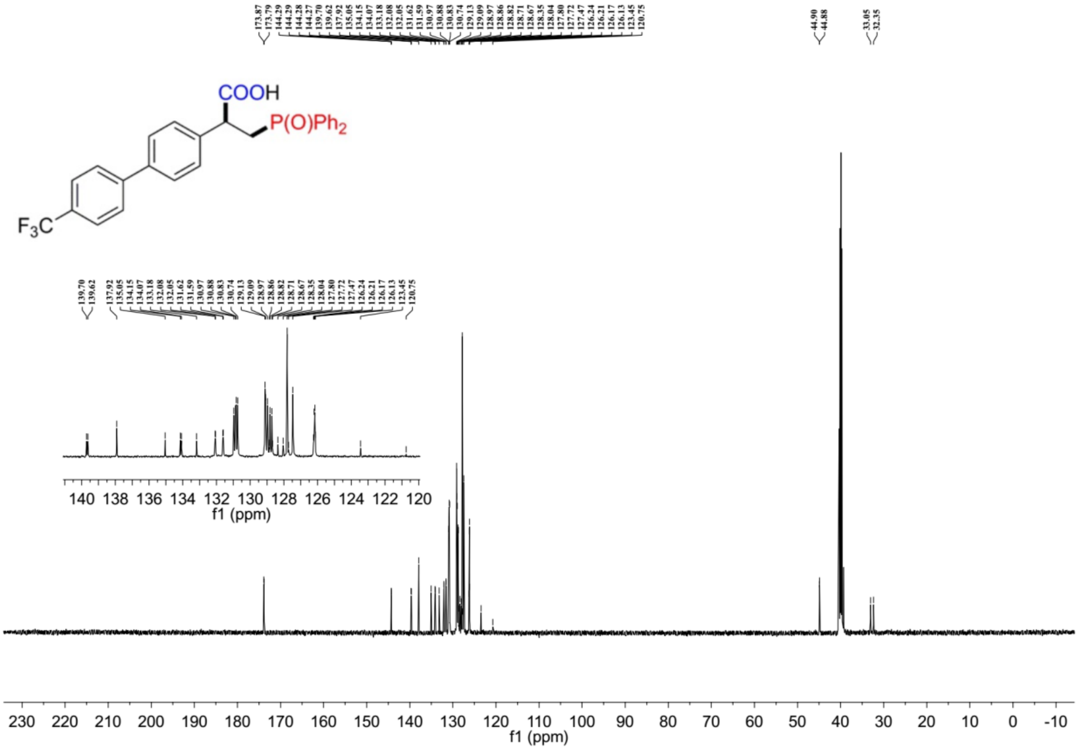

Supplementary Figure 161. ^{13}C NMR spectra of **9ja**

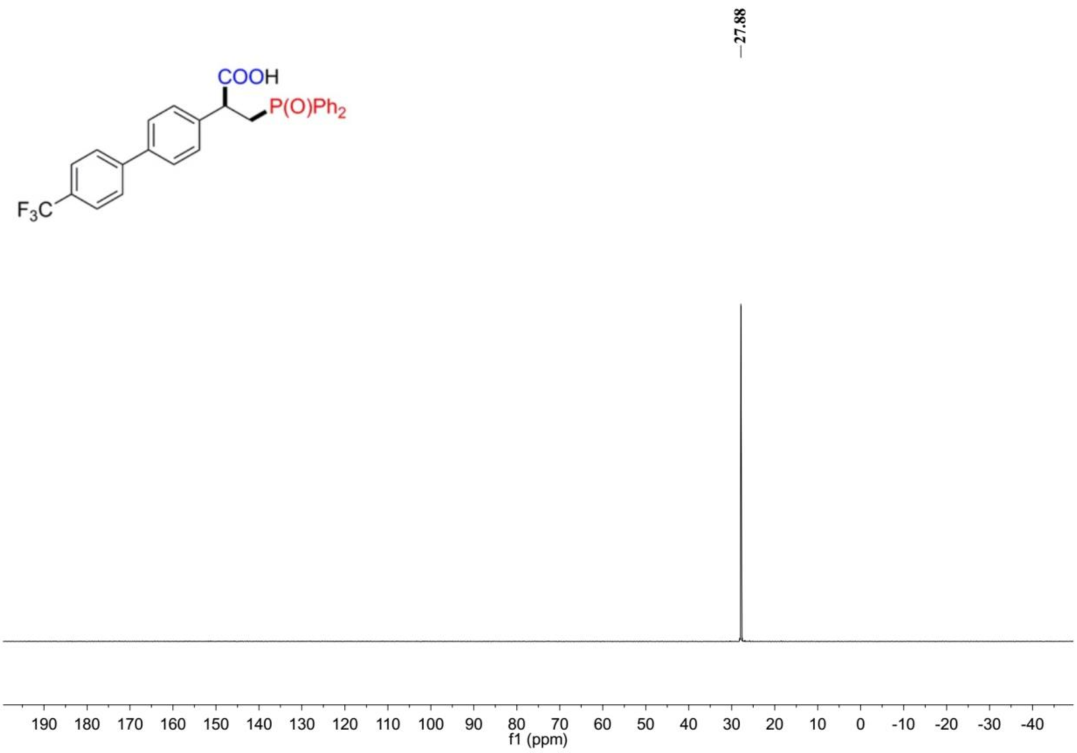

Supplementary Figure 162. ^{31}P NMR spectra of **9ja**

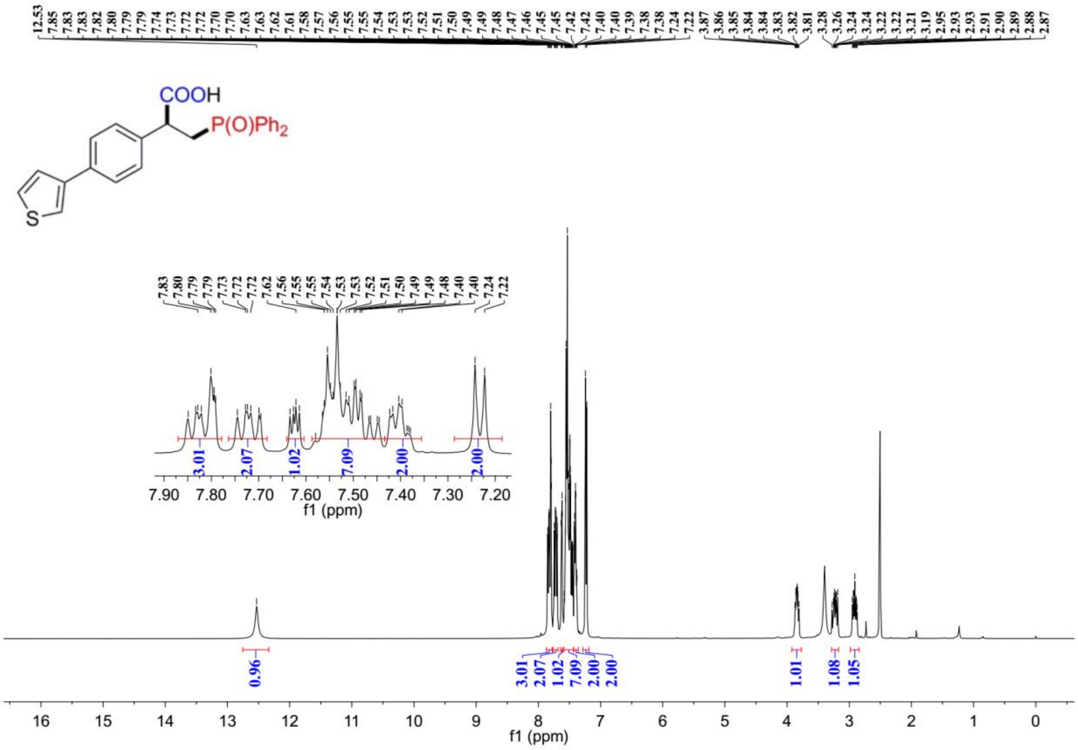

Supplementary Figure 163. ^{1}H NMR spectra of **9ka**

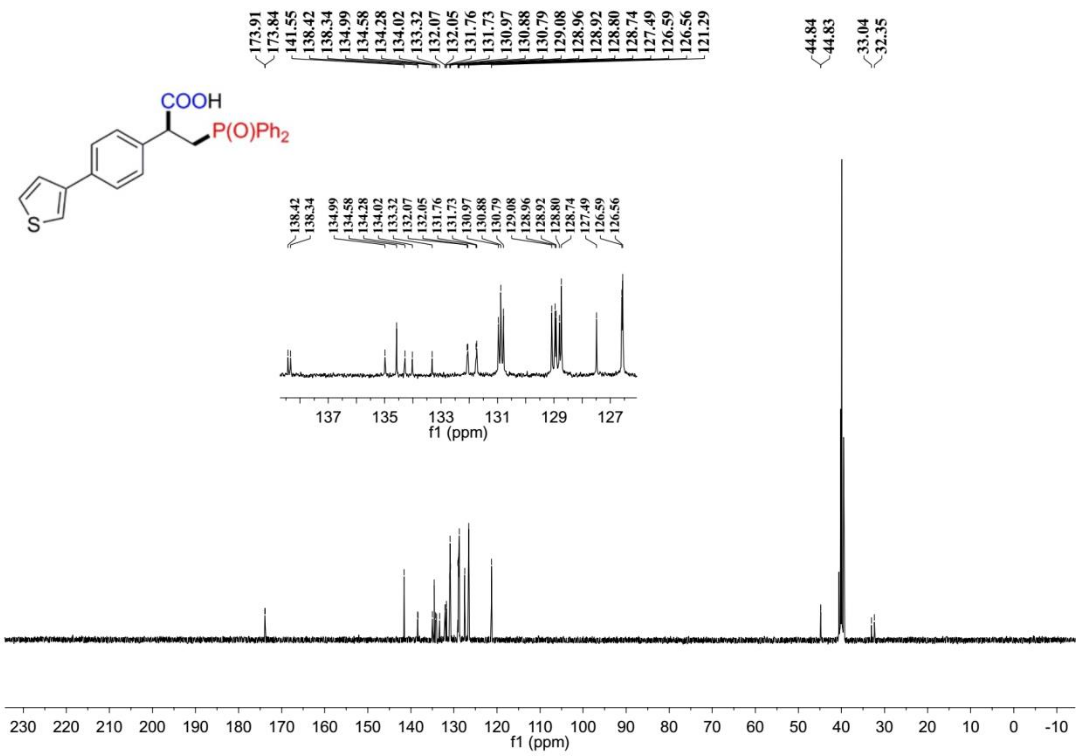

Supplementary Figure 164. ^{13}C NMR spectra of **9ka**

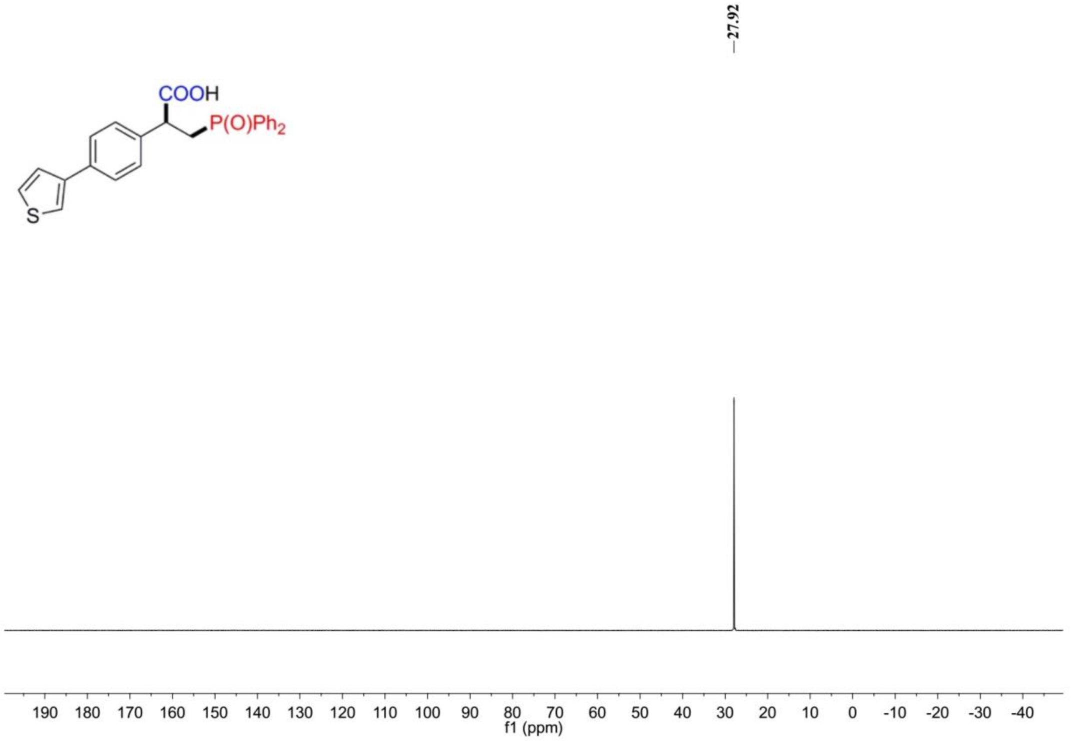

Supplementary Figure 165. ^{31}P NMR spectra of **9ka**

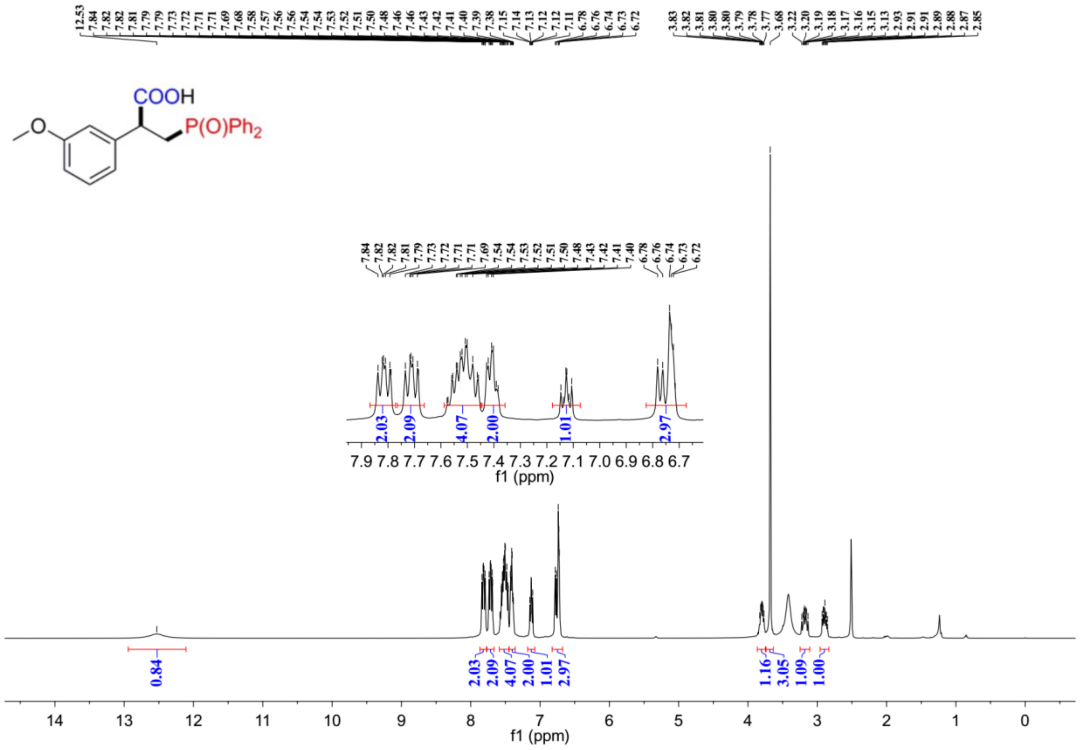

Supplementary Figure 166. ^{1}H NMR spectra of **9la**

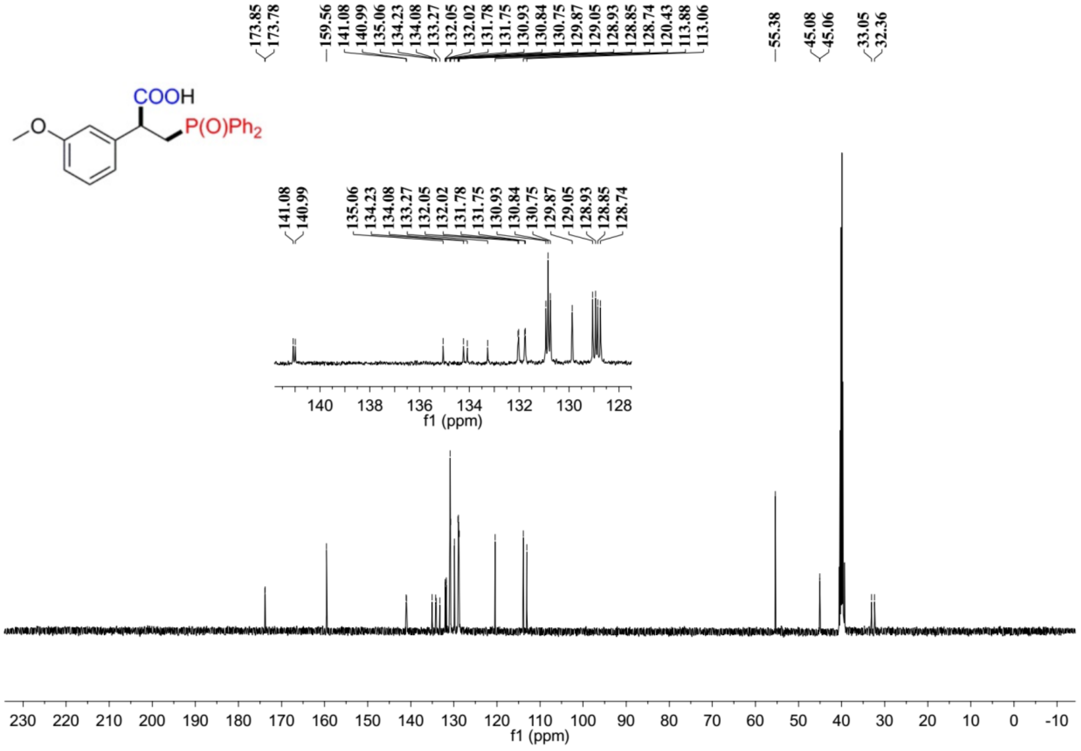

Supplementary Figure 167. ^{13}C NMR spectra of **9la**

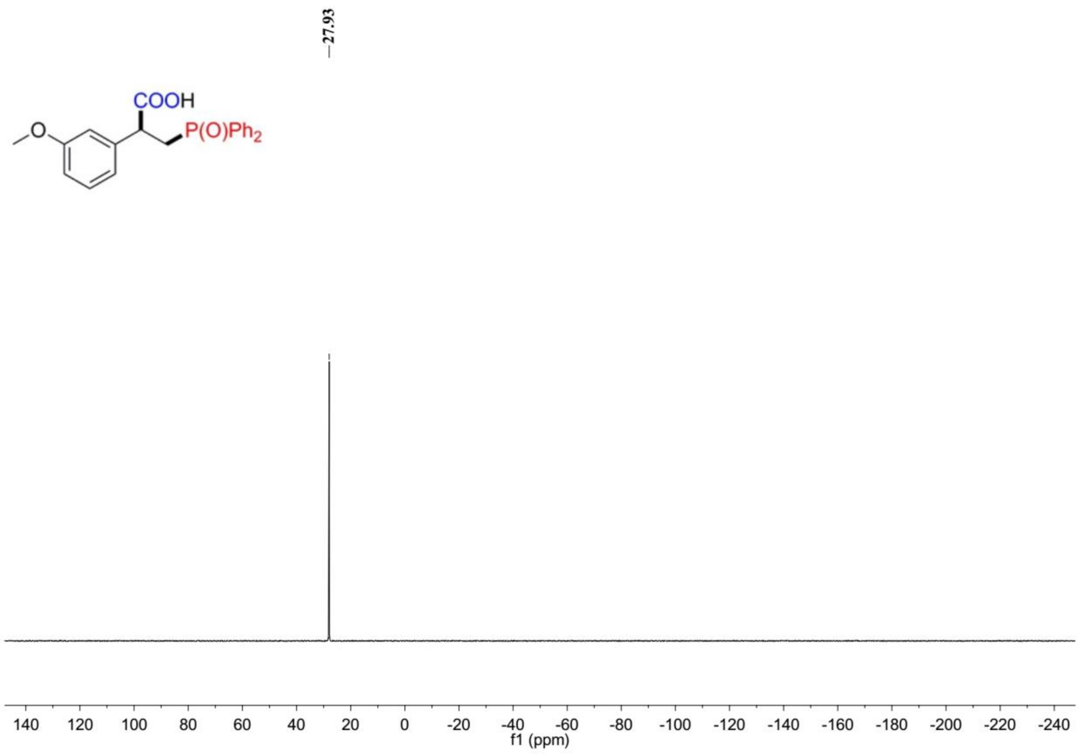

Supplementary Figure 168. ^{31}P NMR spectra of **9la**

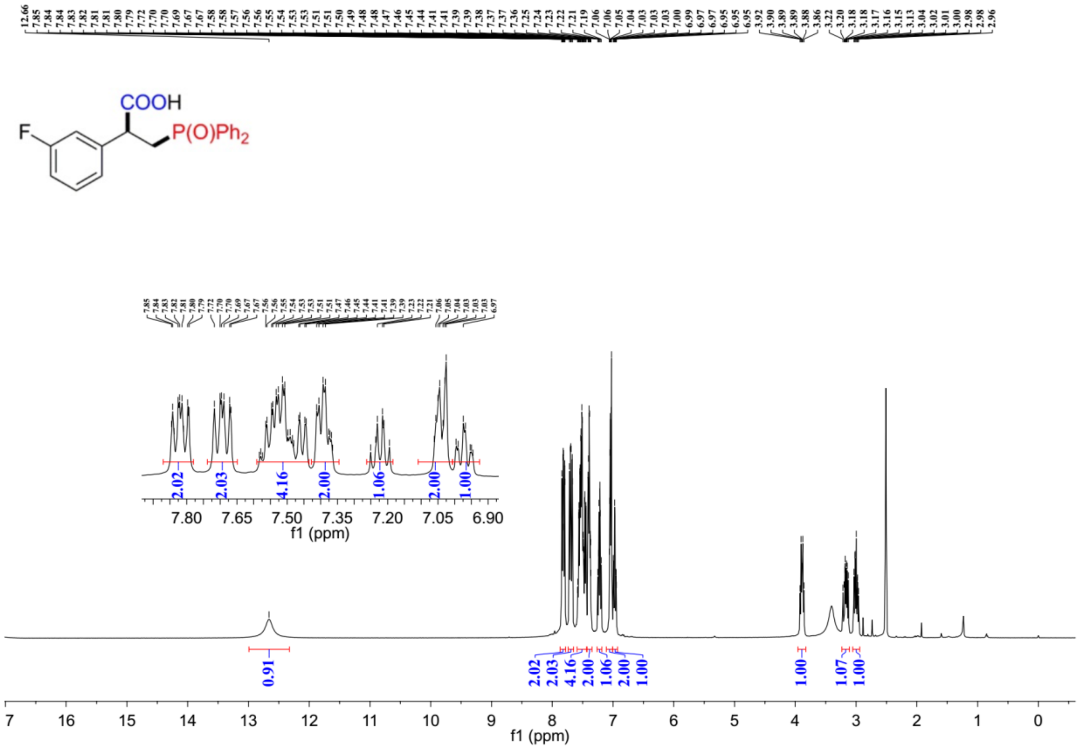

Supplementary Figure 169. ^{1}H NMR spectra of **9ma**

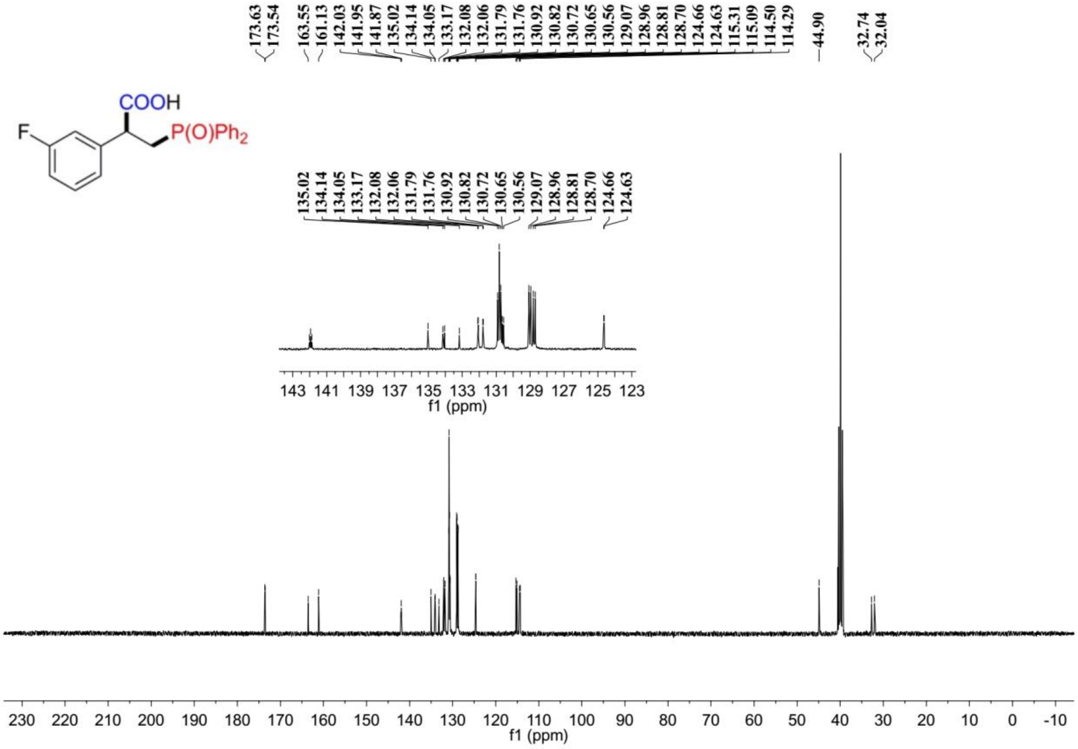

Supplementary Figure 170. ^{13}C NMR spectra of **9ma**

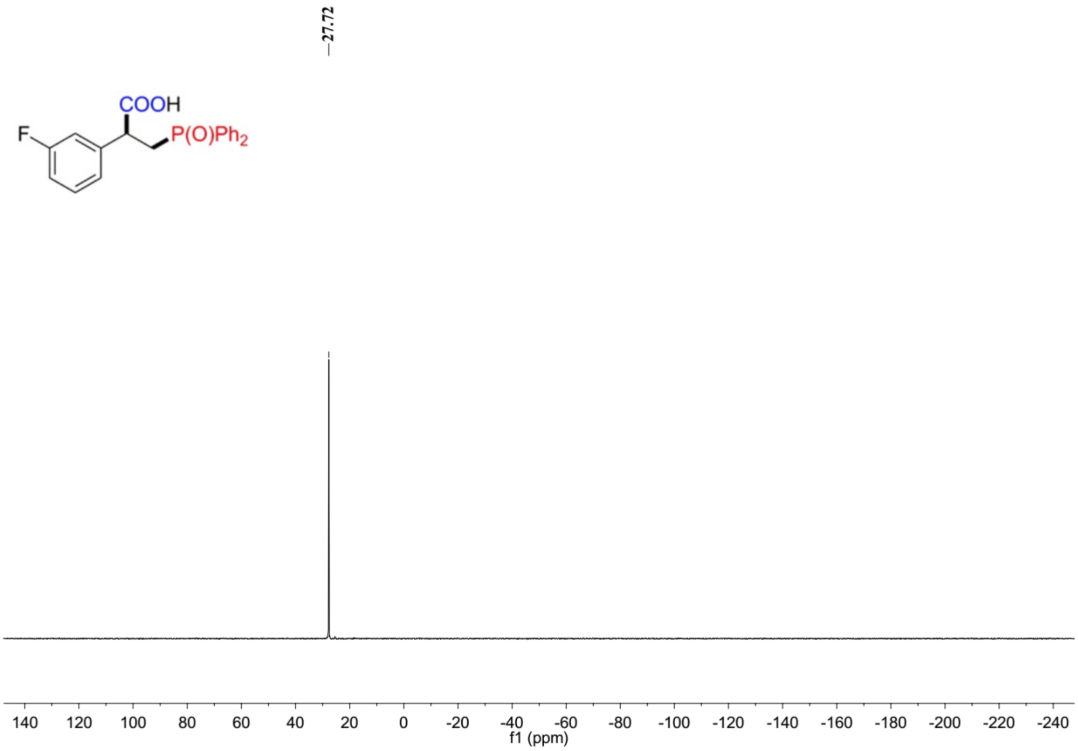

Supplementary Figure 171. ^{31}P NMR spectra of **9ma**

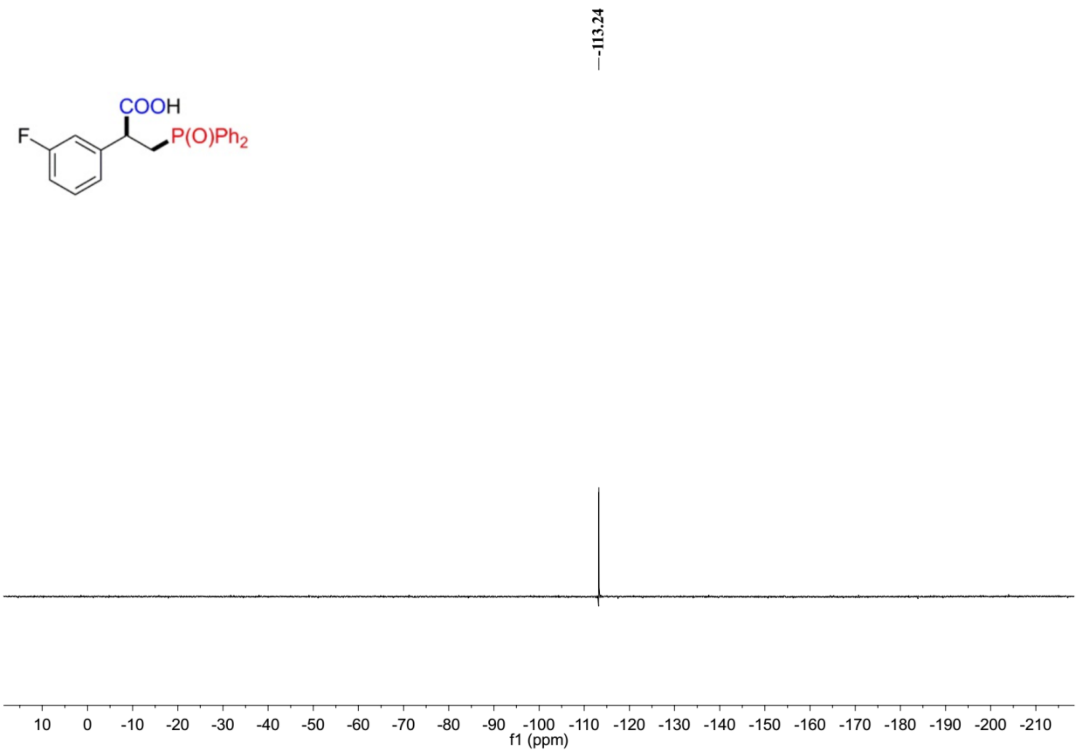

Supplementary Figure 172. ^{19}F NMR spectra of **9ma**

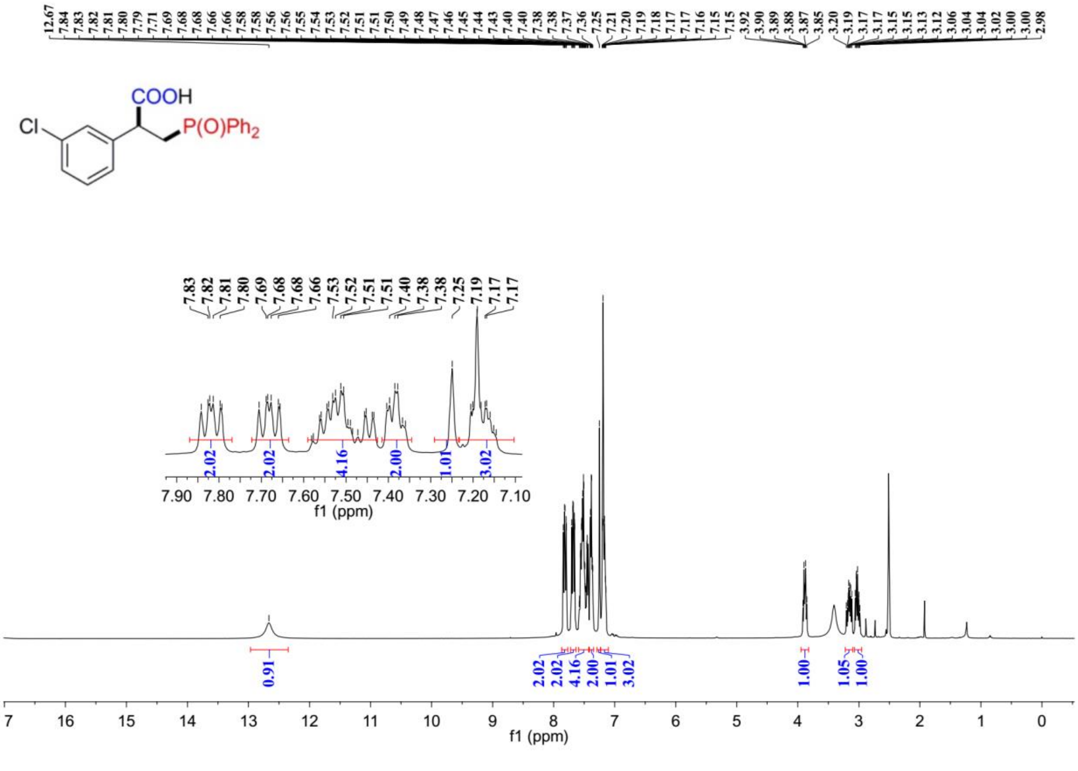

Supplementary Figure 173. ^{1}H NMR spectra of **9na**

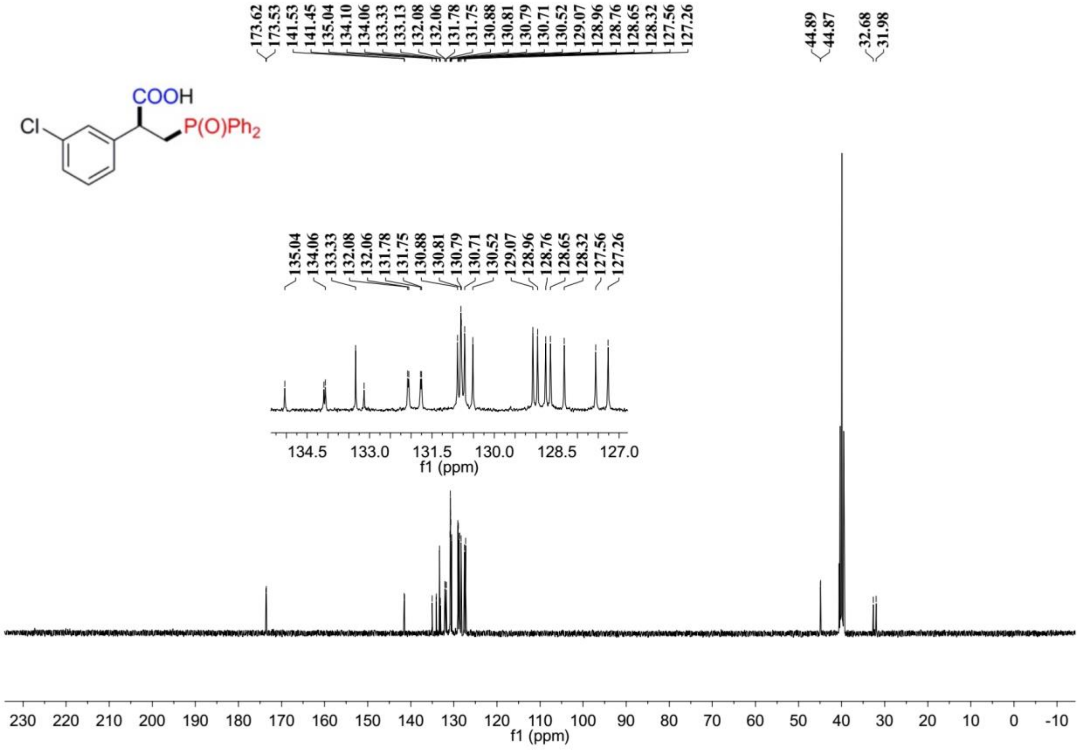

Supplementary Figure 174. ^{13}C NMR spectra of **9na**

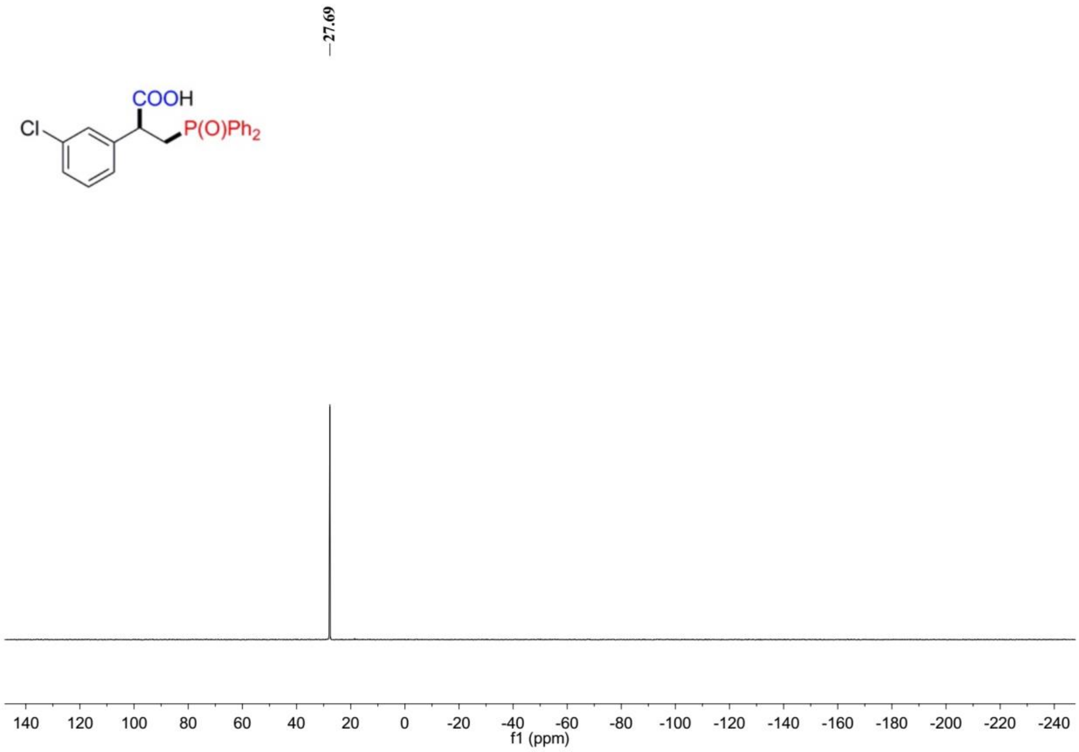

Supplementary Figure 175. ^{31}P NMR spectra of **9na**

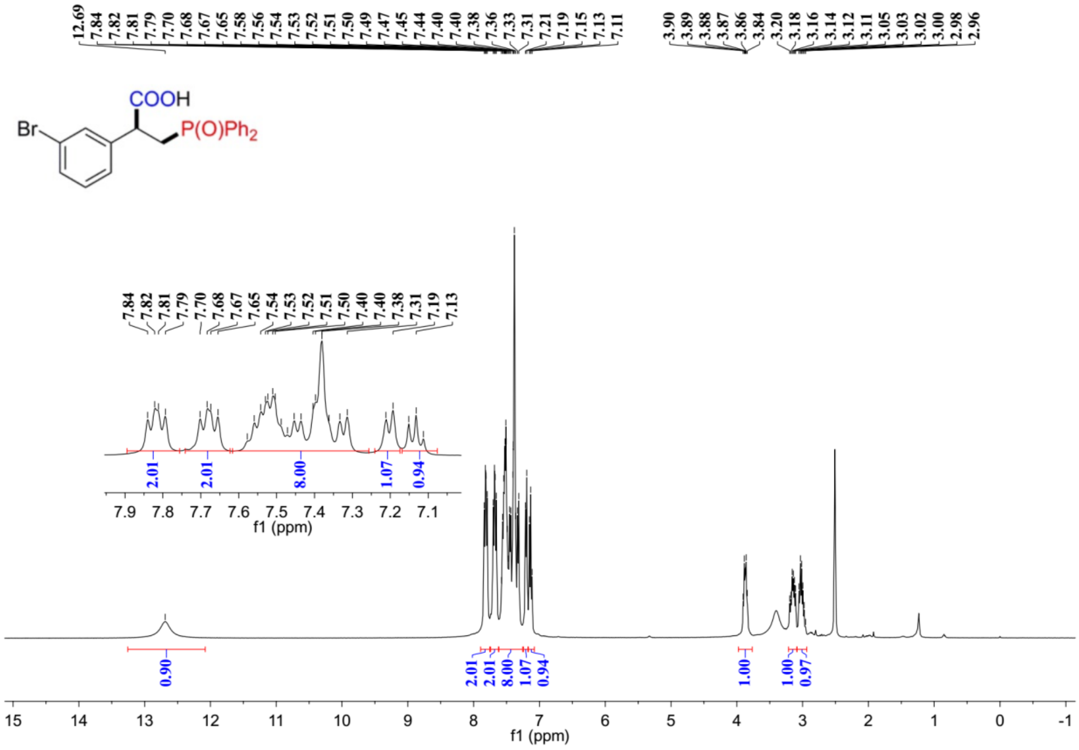

Supplementary Figure 176. ^{1}H NMR spectra of **9oa**

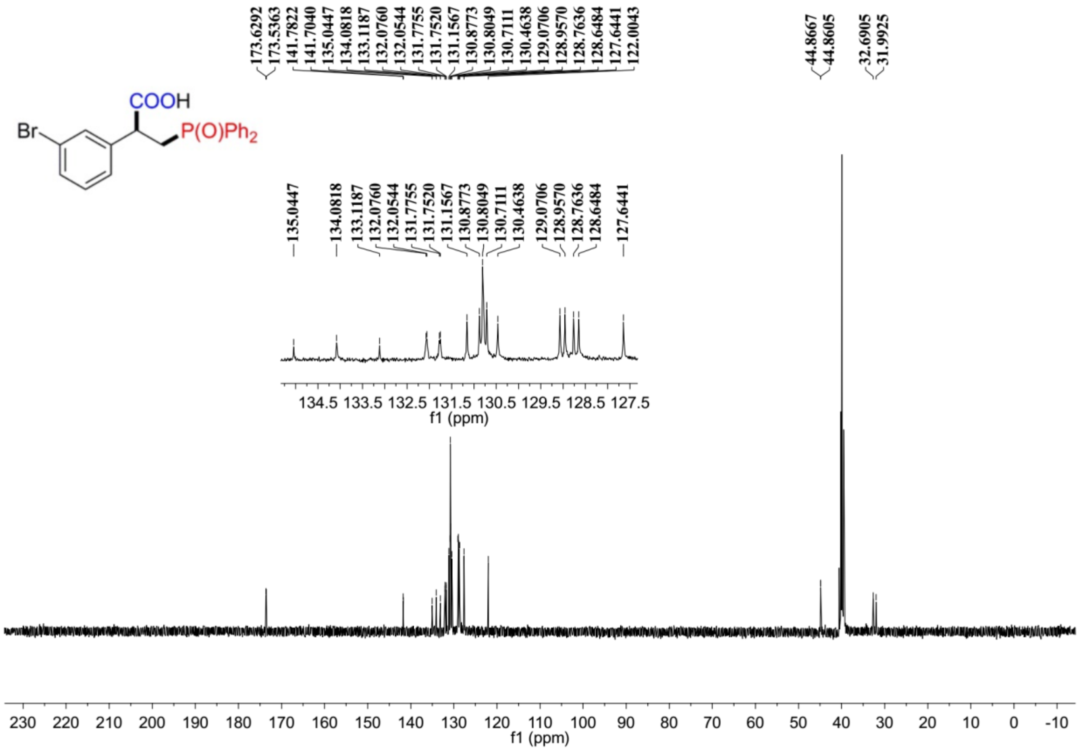

Supplementary Figure 177. ^{13}C NMR spectra of **3ca**

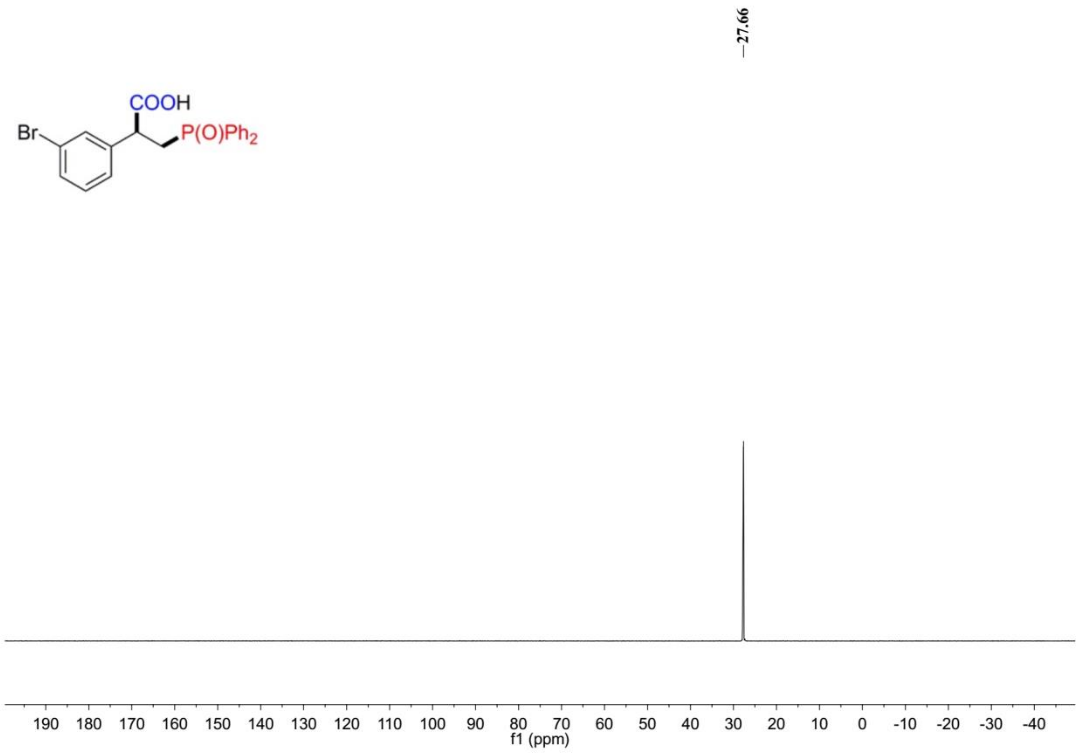

Supplementary Figure 178. ^{31}P NMR spectra of **3ca**

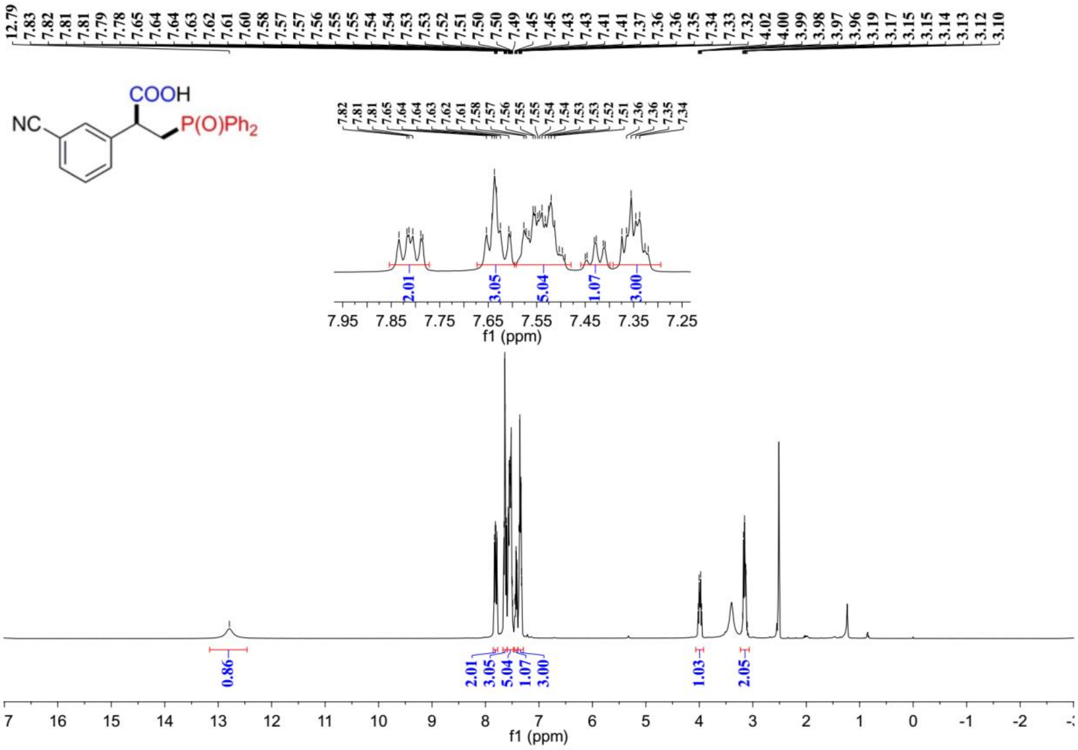

Supplementary Figure 179. ^{1}H NMR spectra of **9pa**

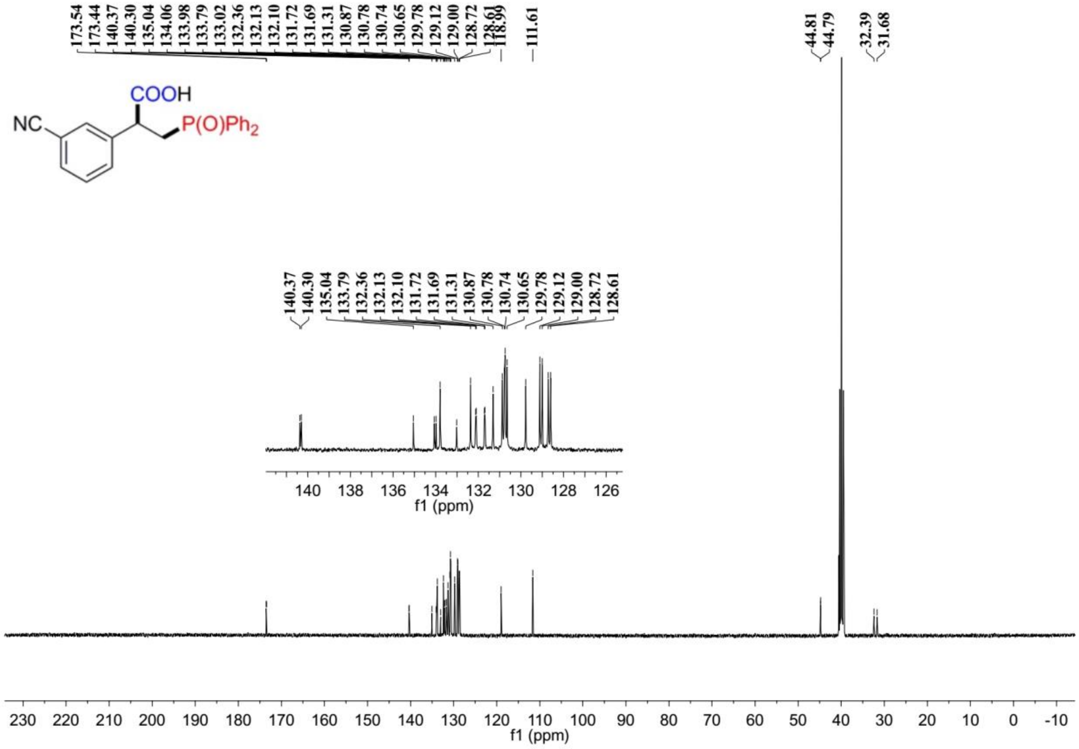

Supplementary Figure 180. ^{13}C NMR spectra of **9pa**

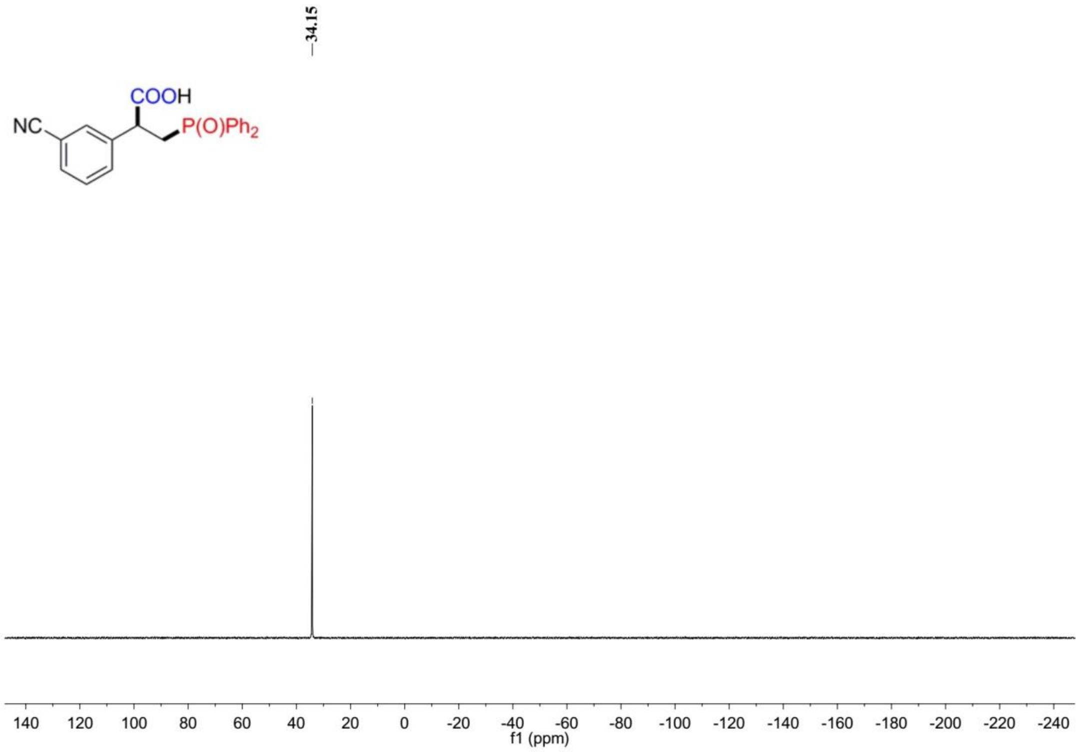

Supplementary Figure 181. ^{31}P NMR spectra of **9pa**

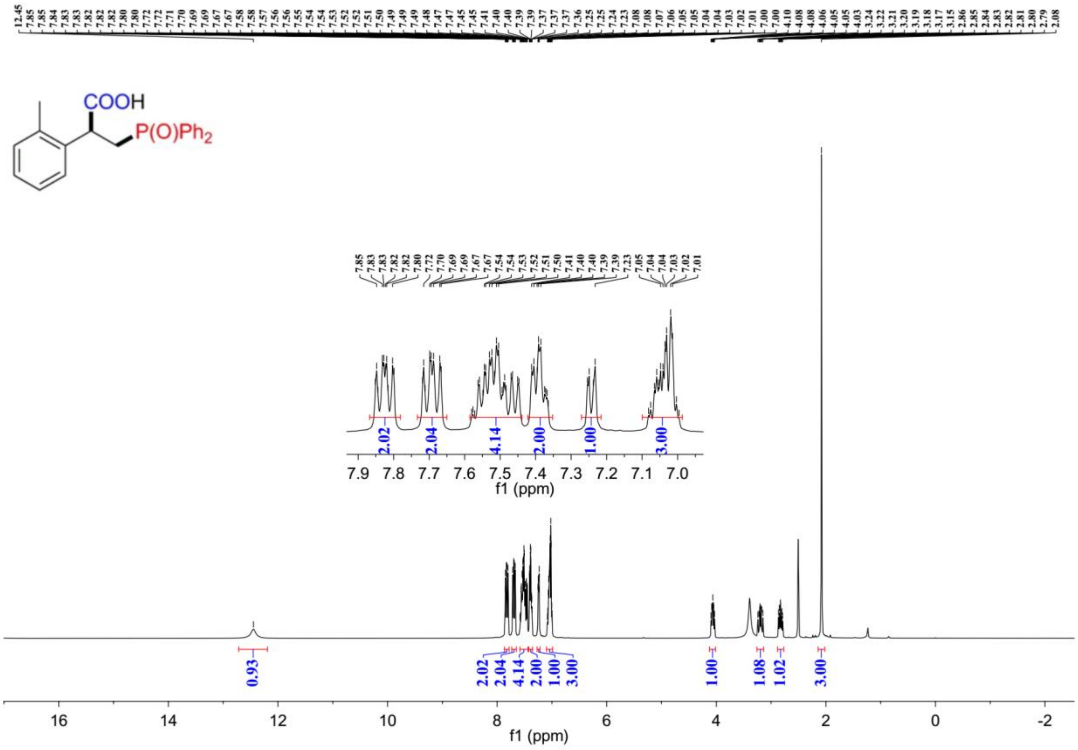

Supplementary Figure 182. ^{1}H NMR spectra of **9qa**

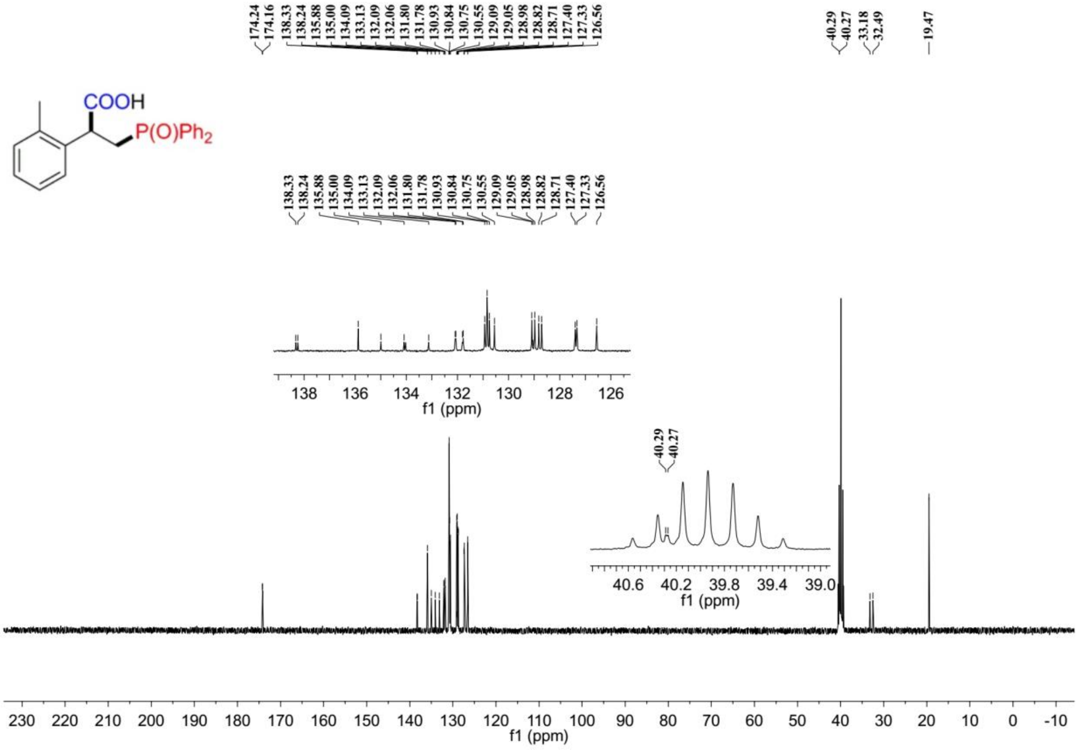

Supplementary Figure 183. ^{13}C NMR spectra of **9qa**

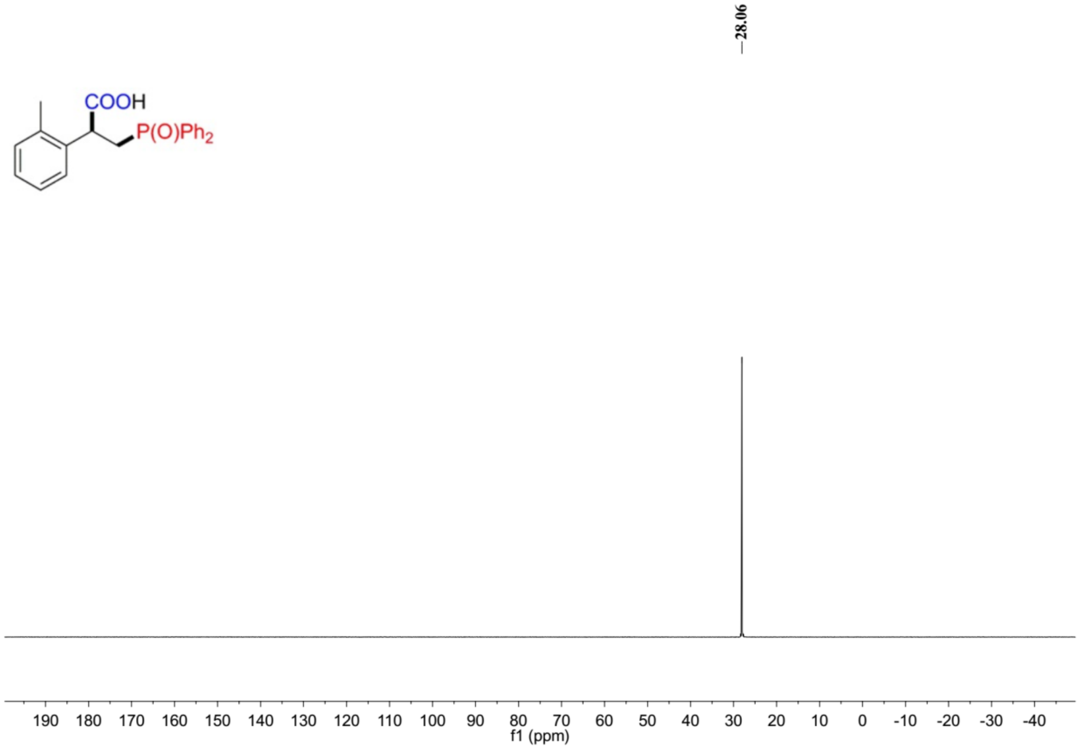

Supplementary Figure 184. ^{31}P NMR spectra of **9qa**

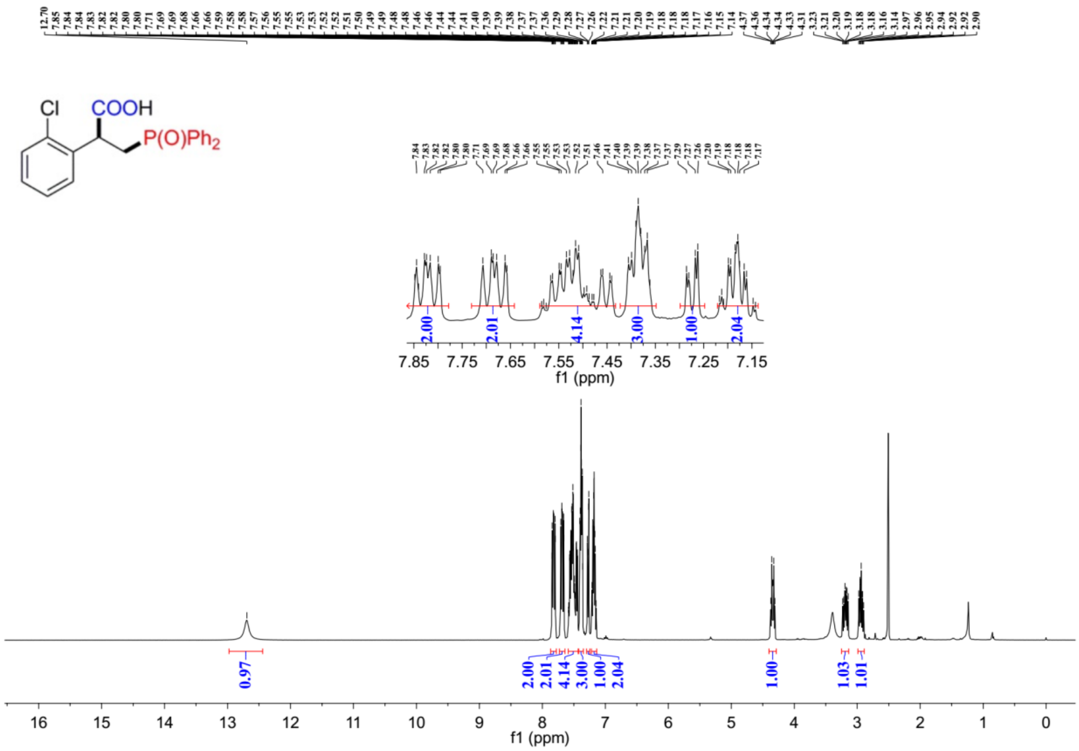

Supplementary Figure 185. ^{1}H NMR spectra of **9ra**

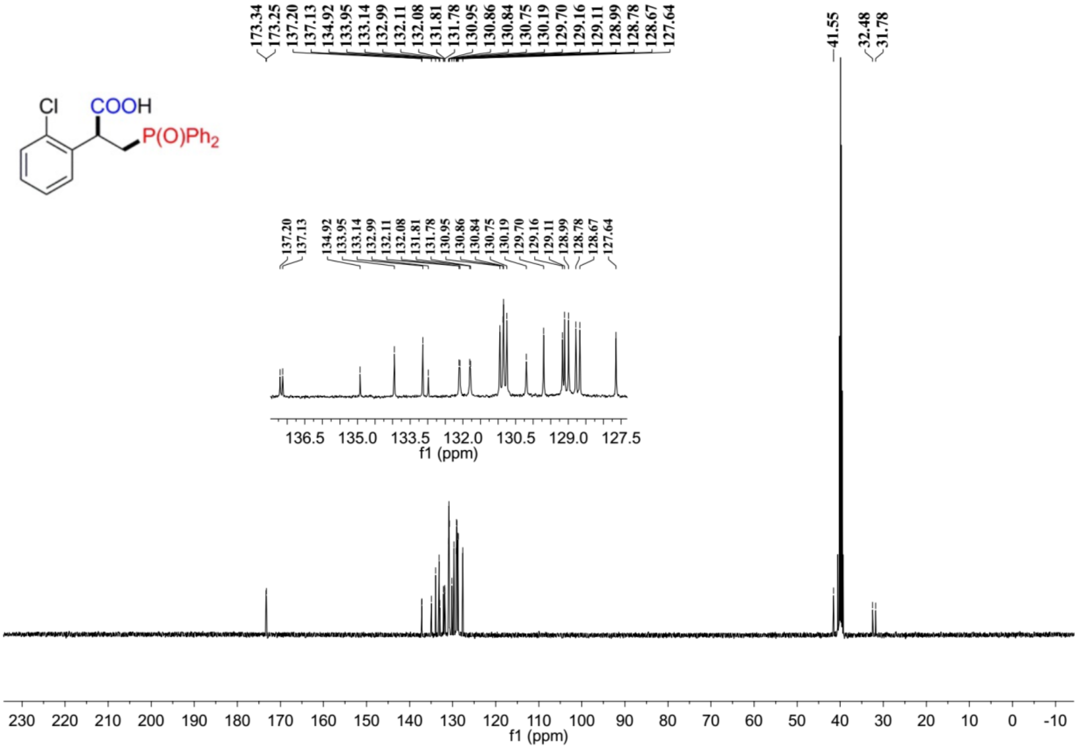

Supplementary Figure 186. ^{13}C NMR spectra of **9ra**

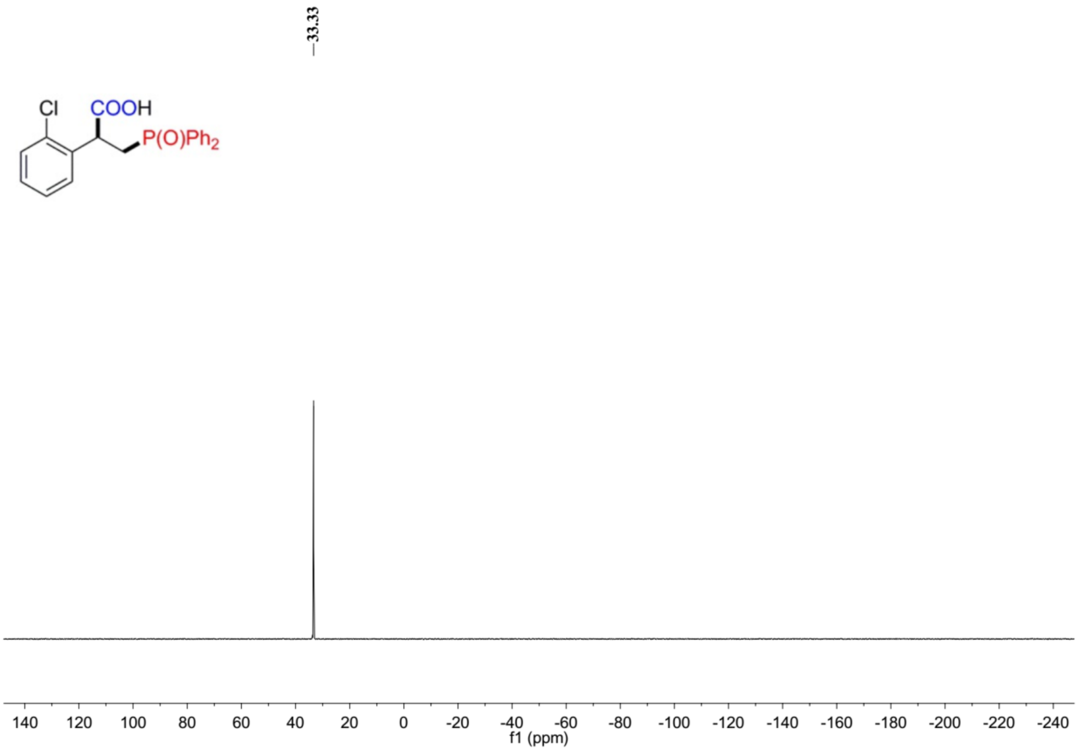

Supplementary Figure 187. ^{31}P NMR spectra of **9ra**

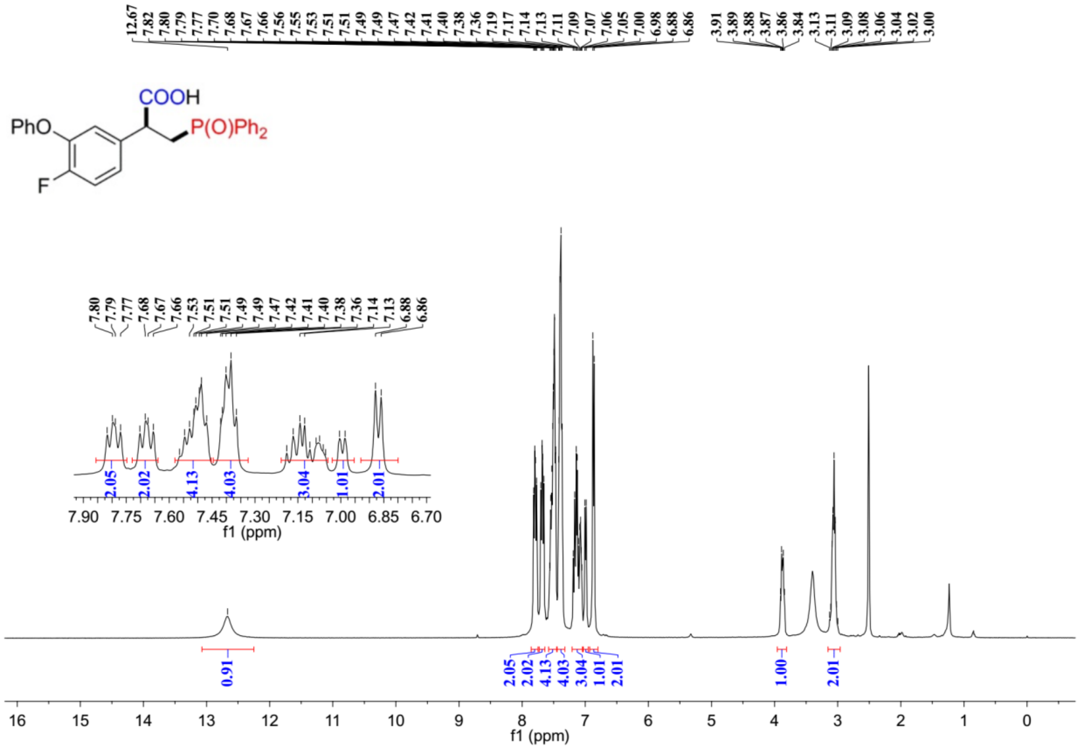

Supplementary Figure 188. ^{1}H NMR spectra of **9sa**

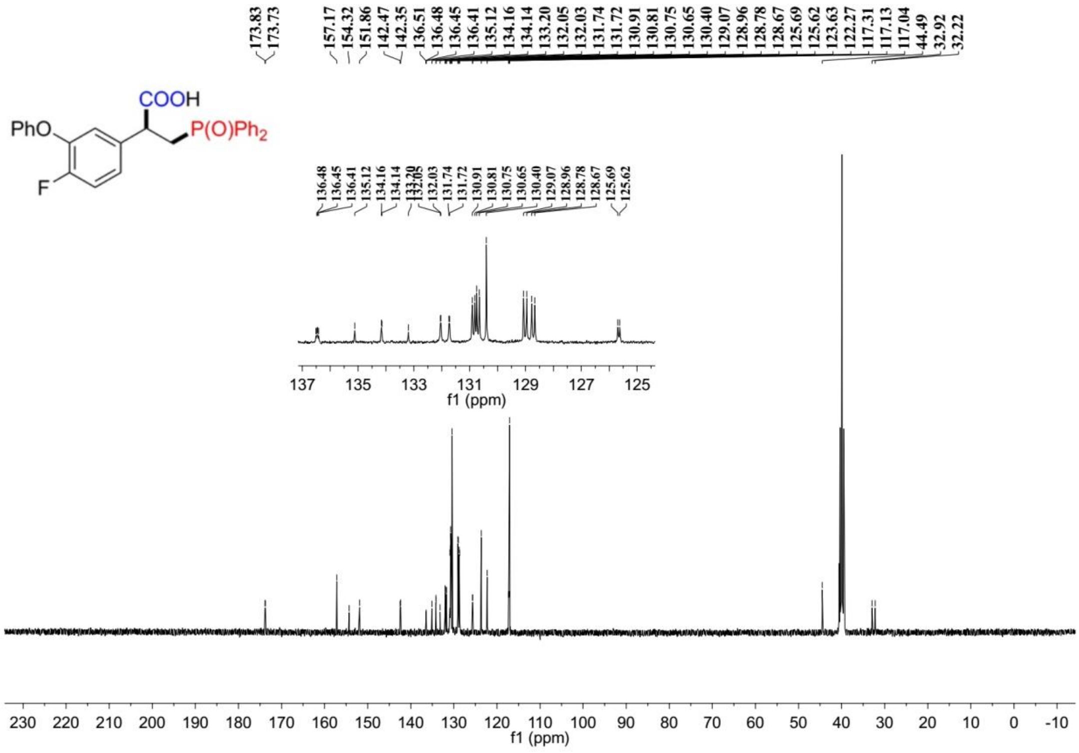

Supplementary Figure 189. ^{13}C NMR spectra of **9sa**

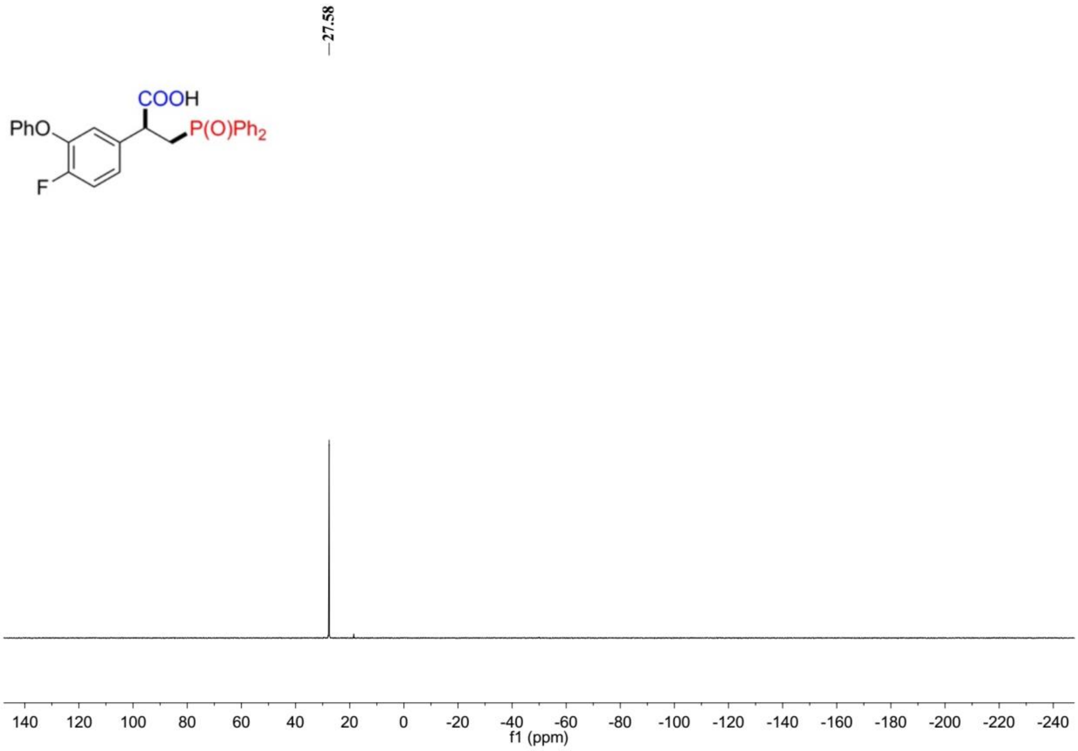

Supplementary Figure 190. ^{31}P NMR spectra of **9sa**

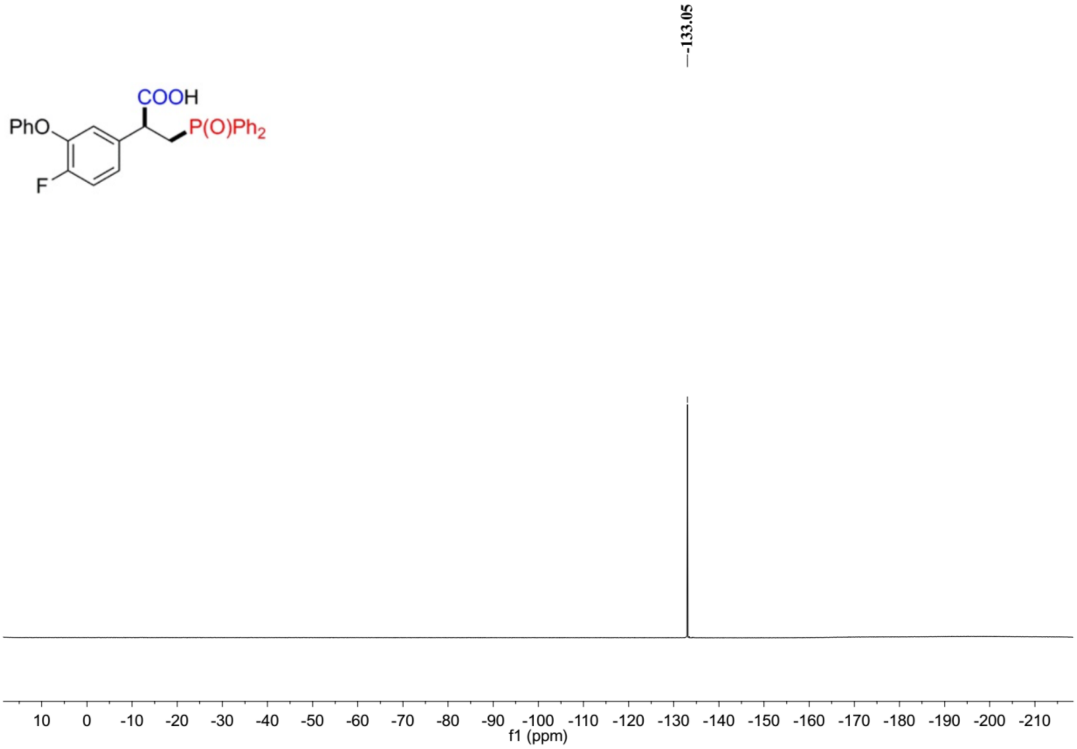

Supplementary Figure 191. ^{19}F NMR spectra of **9sa**

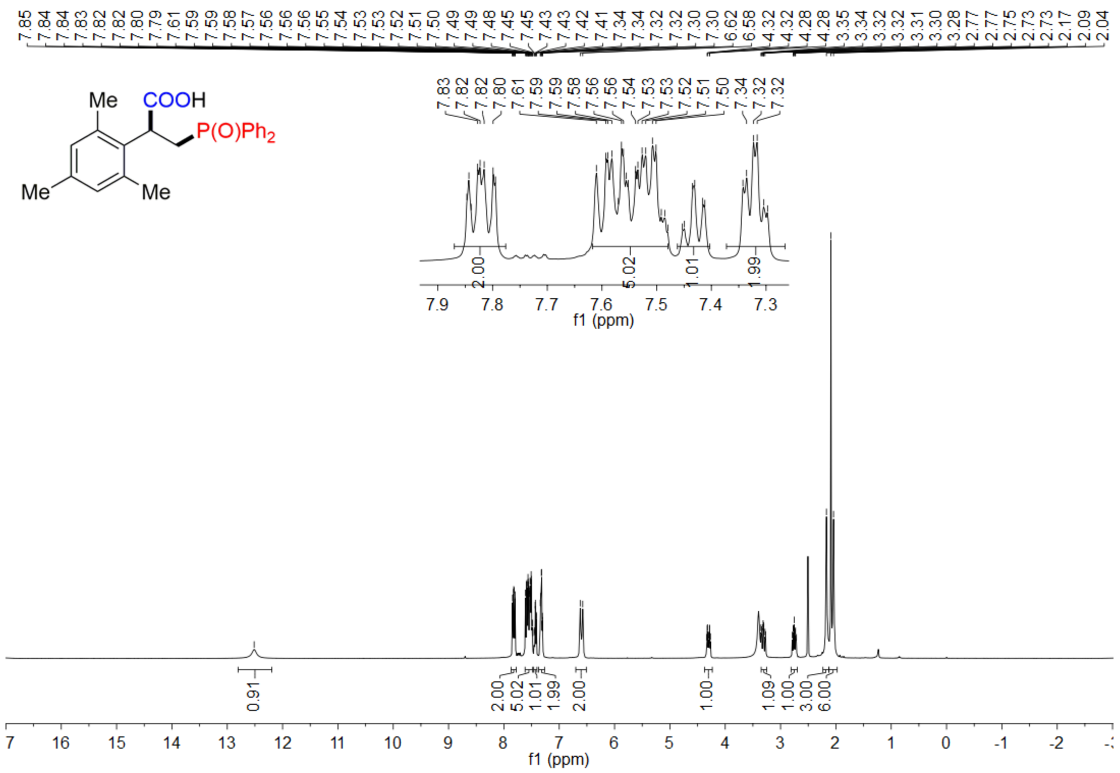

Supplementary Figure 192. ^{1}H NMR spectra of **9ta**

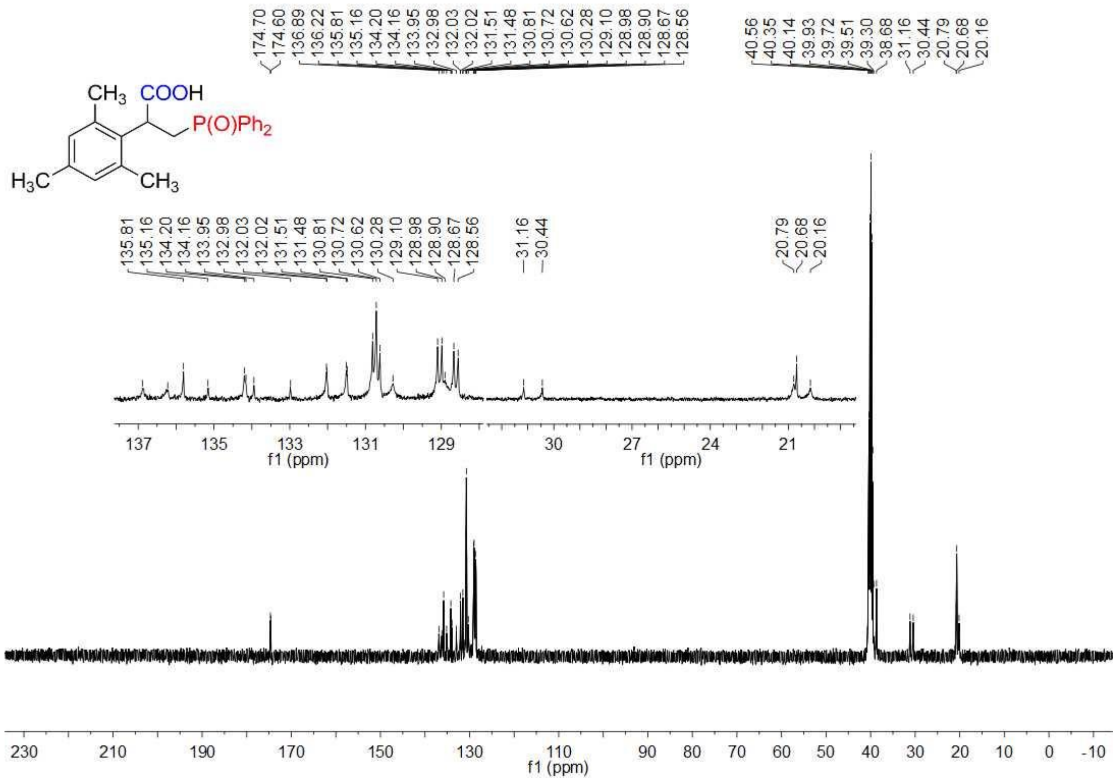

Supplementary Figure 193. ^{13}C NMR spectra of **9ta**

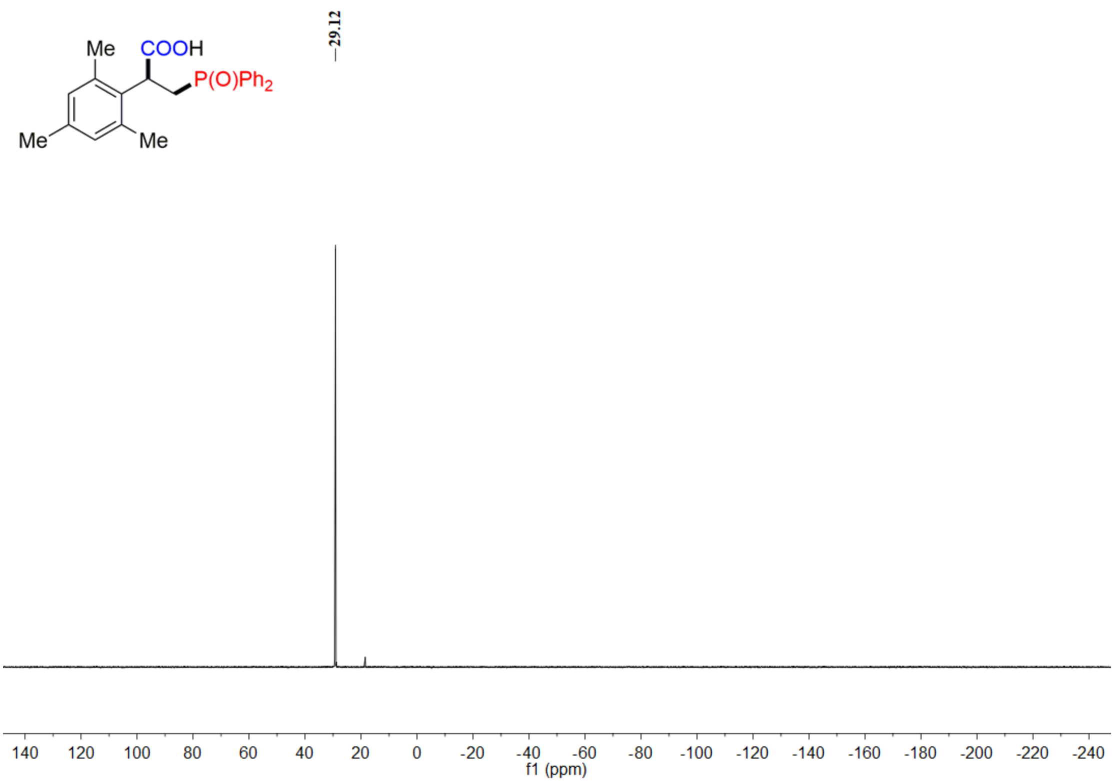

Supplementary Figure 194. ^{31}P NMR spectra of **9ta**

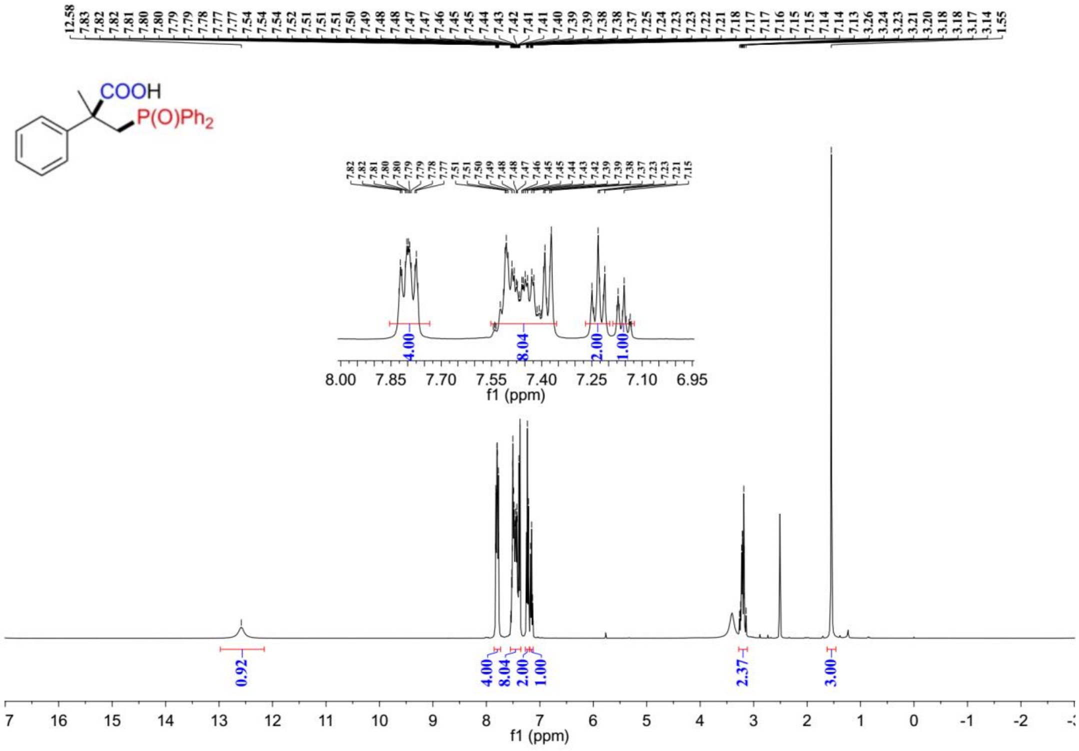

Supplementary Figure 195. ^{1}H NMR spectra of **9ua**

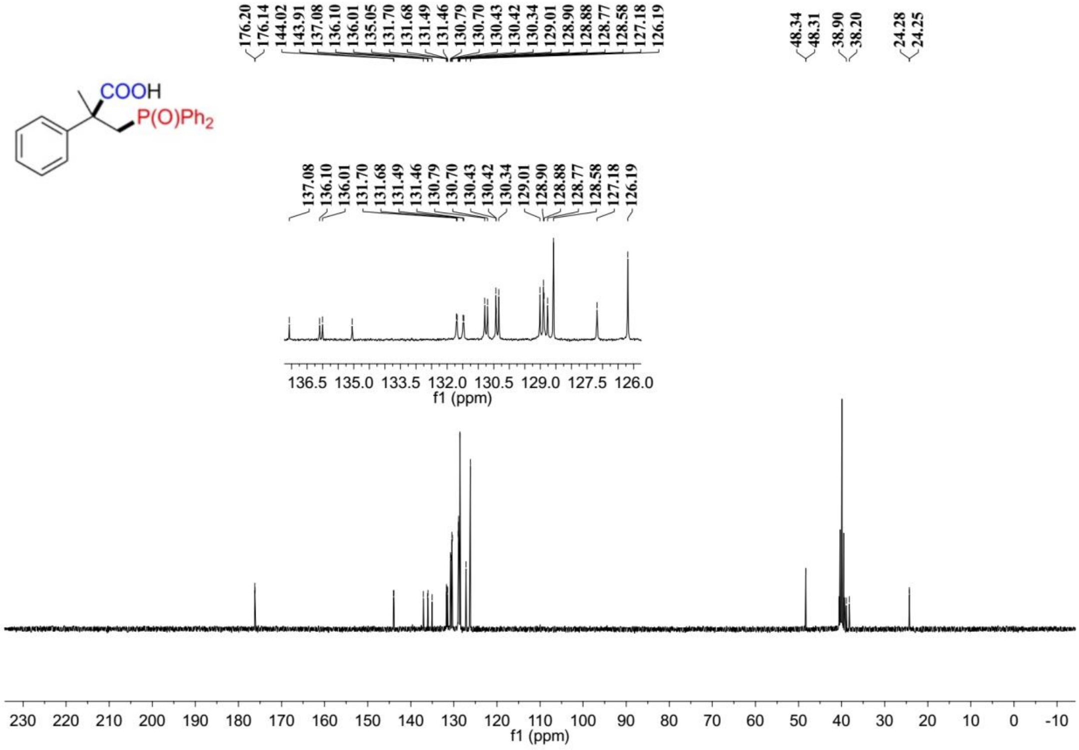

Supplementary Figure 196. ^{13}C NMR spectra of **9ua**

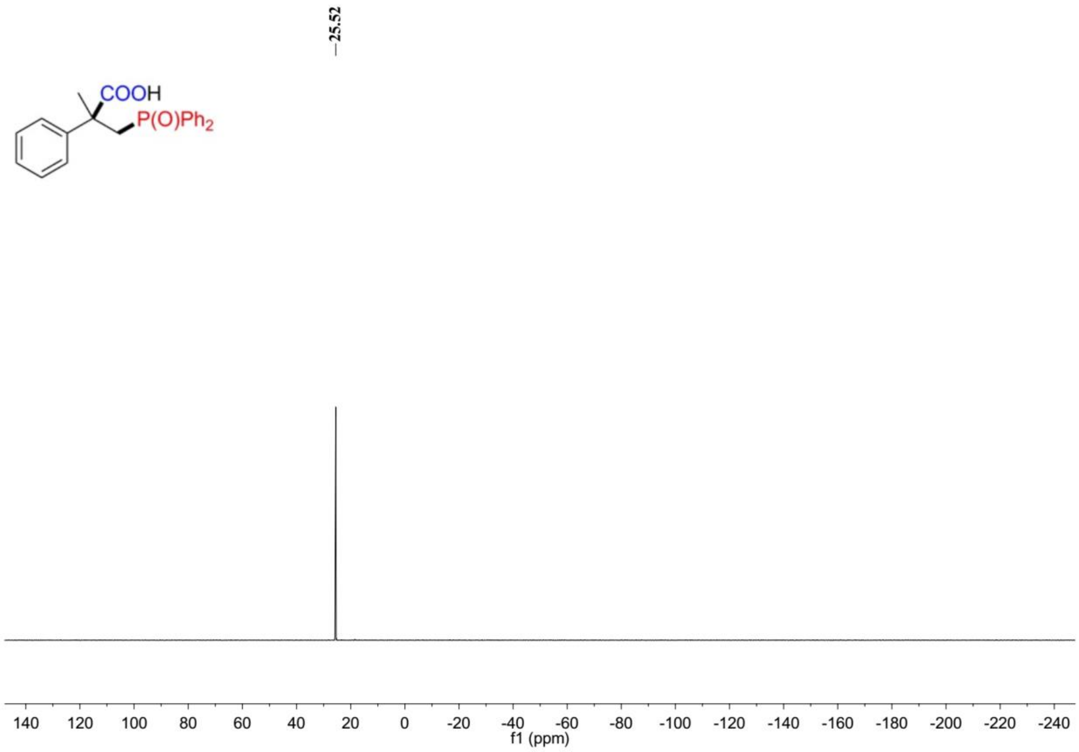

Supplementary Figure 197. ^{31}P NMR spectra of **9ua**

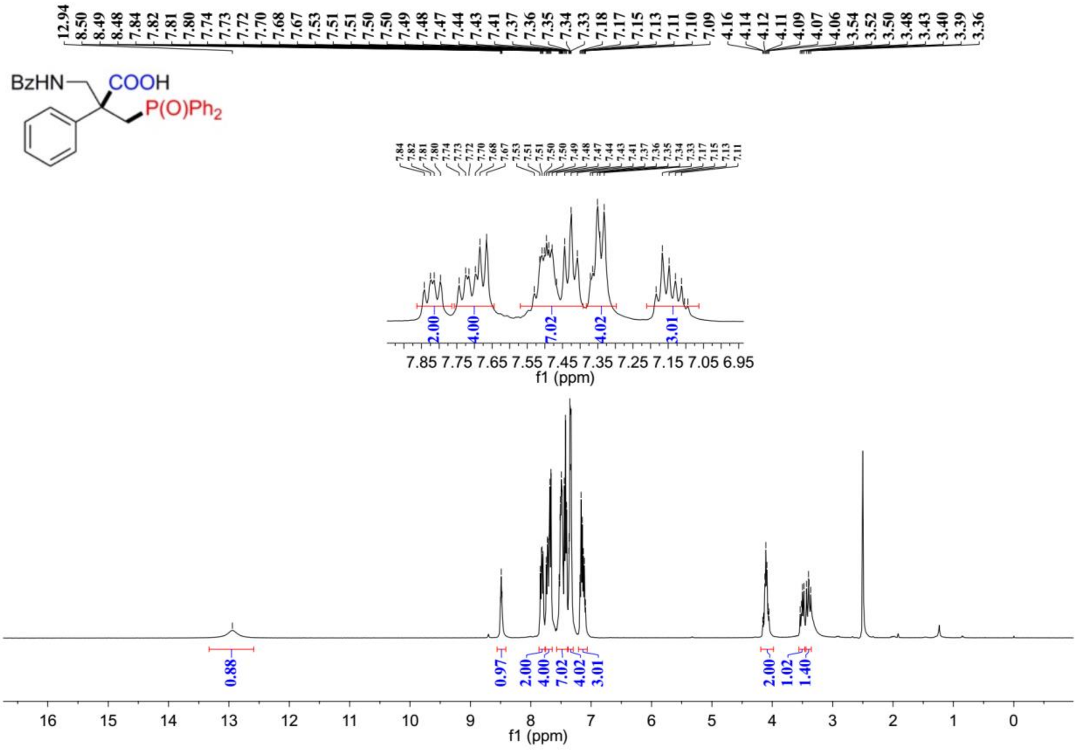

Supplementary Figure 198. ^{1}H NMR spectra of **9va**

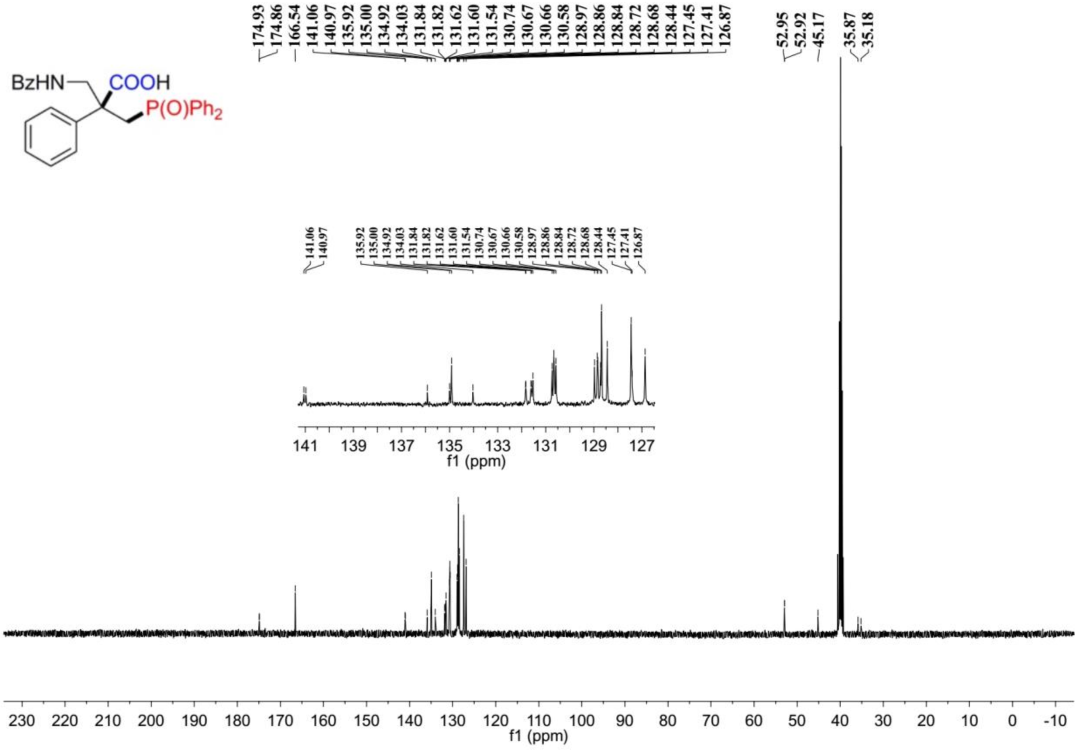

Supplementary Figure 199. ^{13}C NMR spectra of **9va**

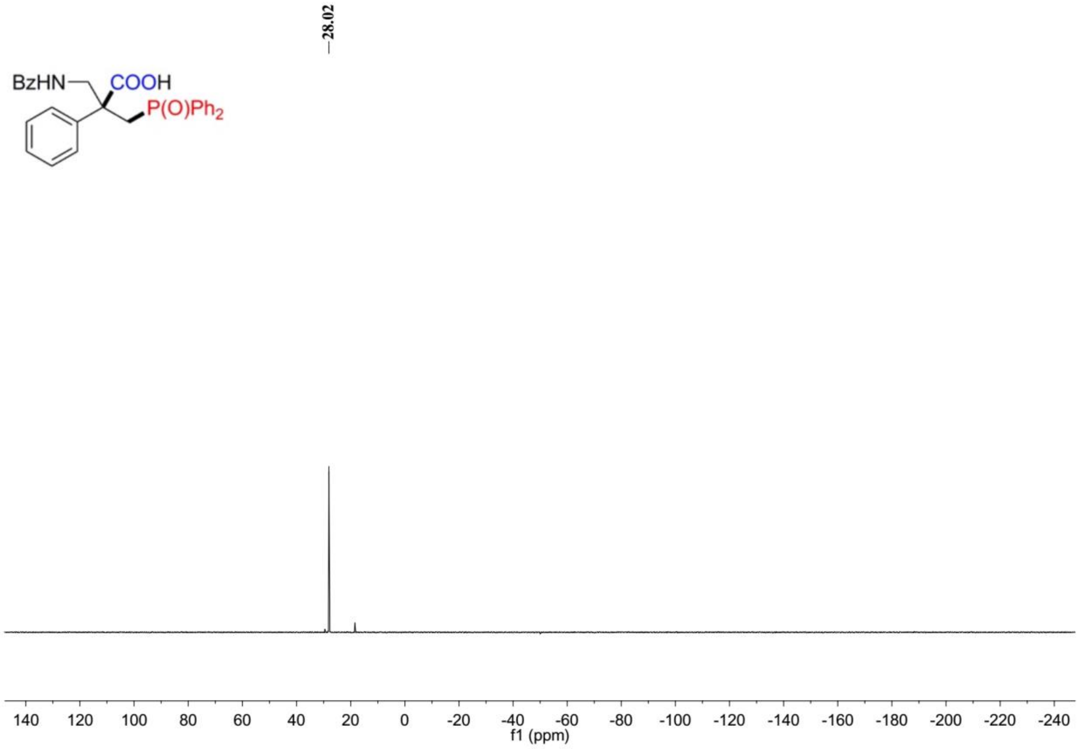

Supplementary Figure 200. ^{31}P NMR spectra of **9va**

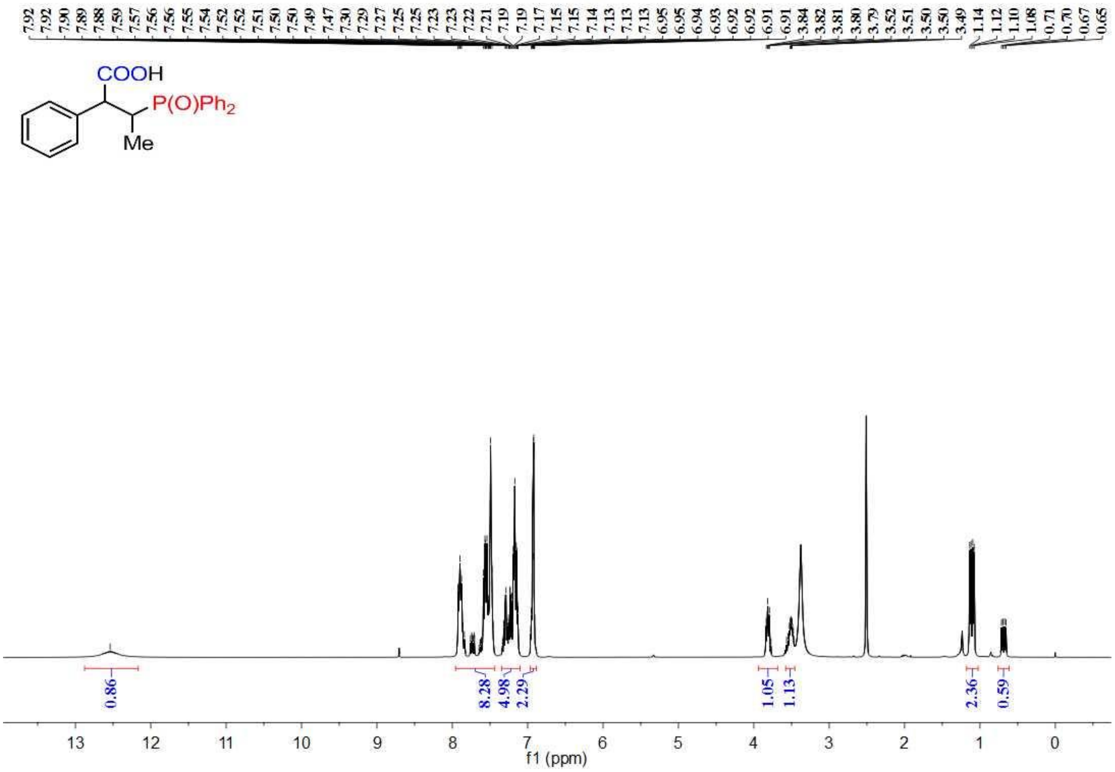

Supplementary Figure 201. ^{1}H NMR spectra of **9wa**

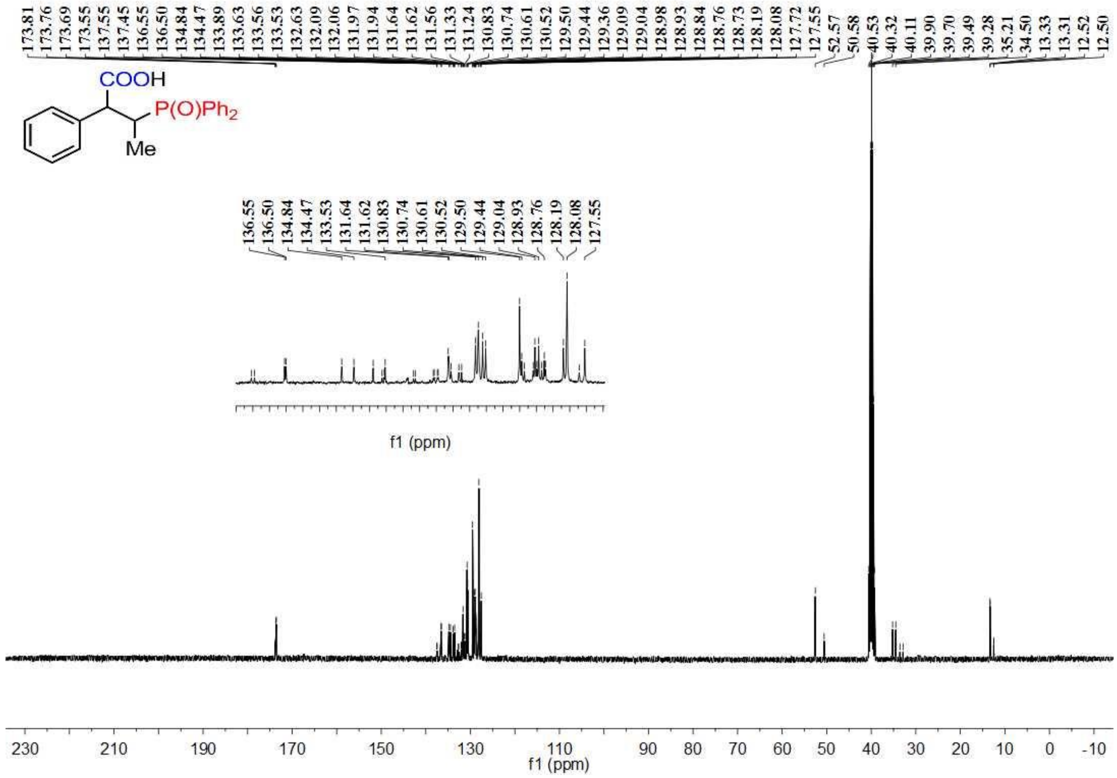

Supplementary Figure 202. ^{13}C NMR spectra of **9wa**

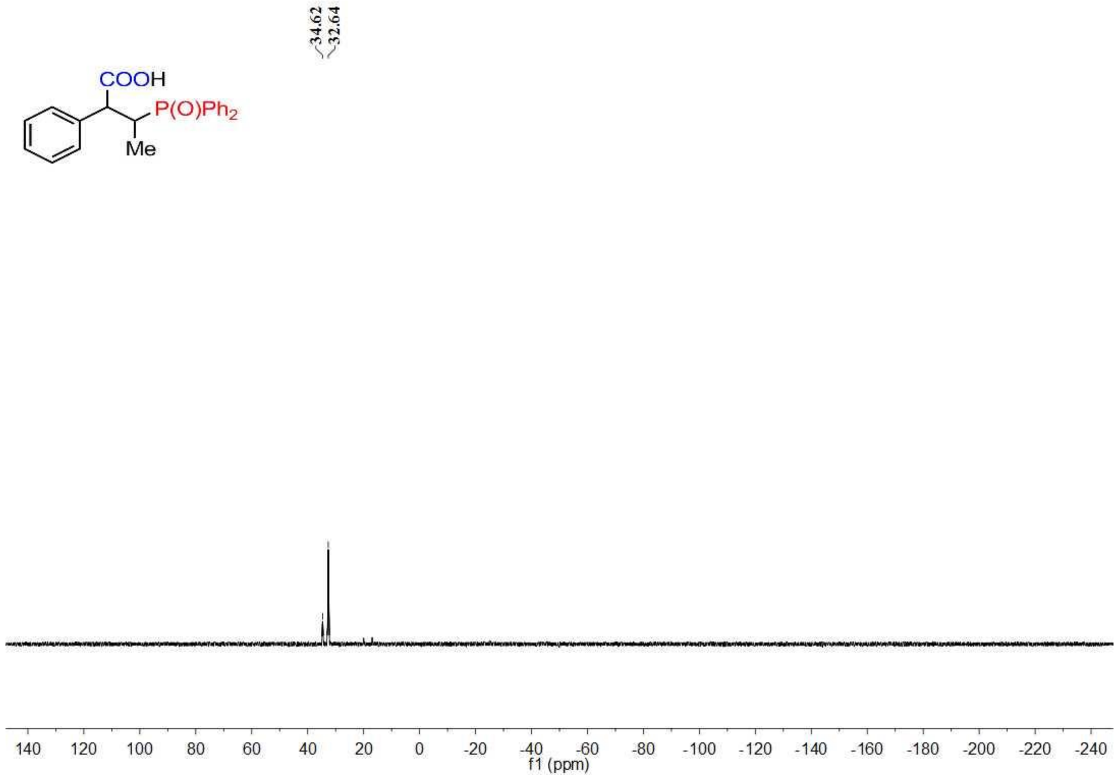

Supplementary Figure 203. ^{31}P NMR spectra of **9wa**

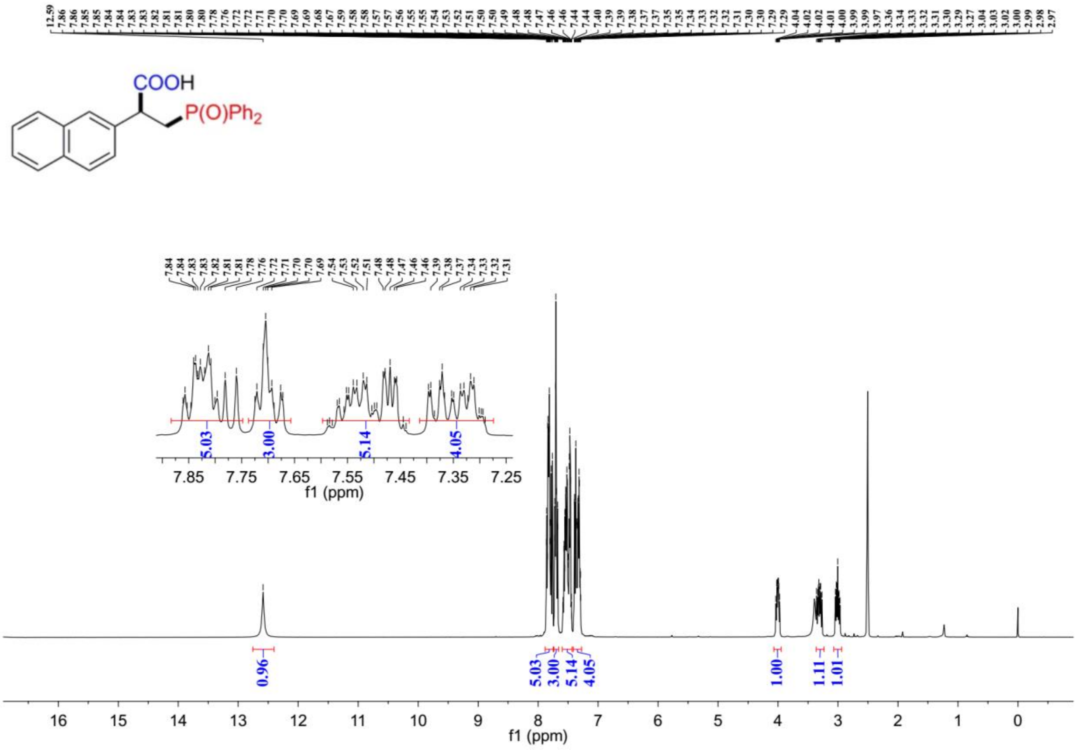

Supplementary Figure 204. ^{1}H NMR spectra of **9xa**

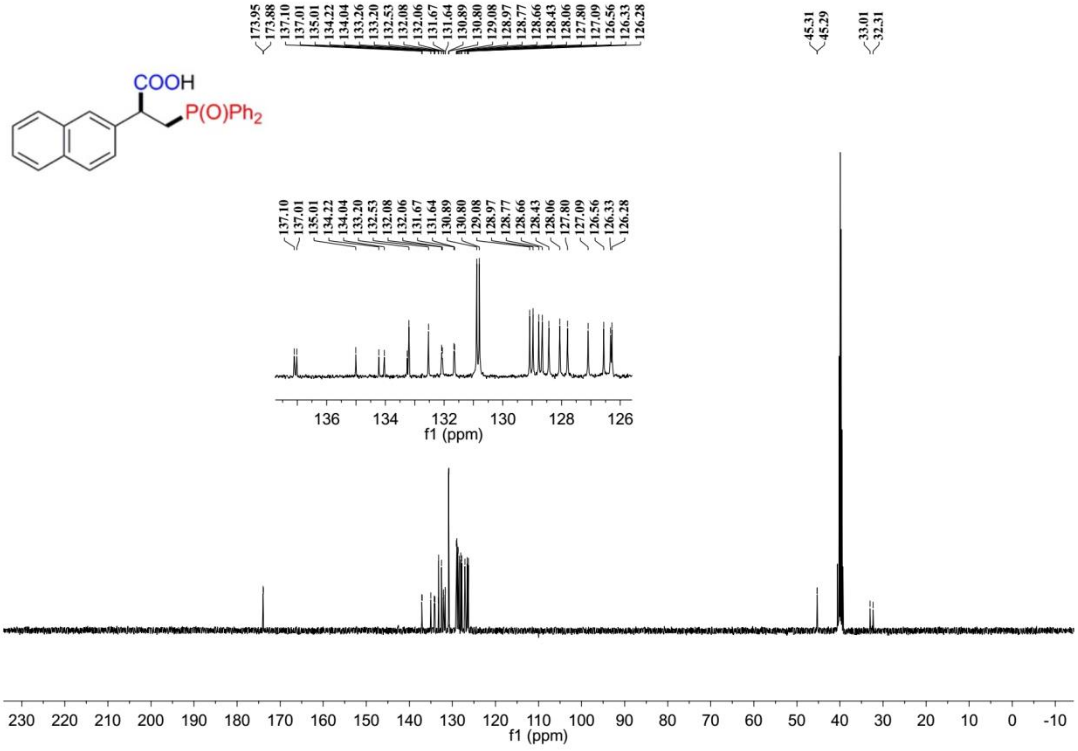

Supplementary Figure 205. ^{13}C NMR spectra of **9xa**

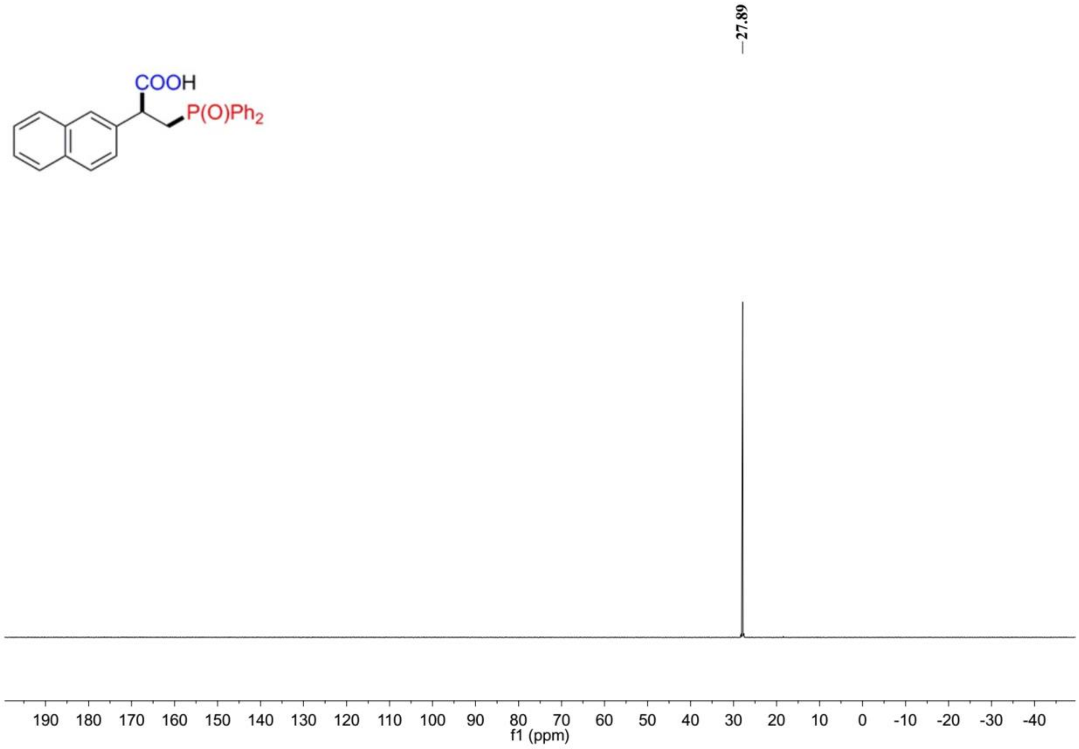

Supplementary Figure 206. ^{31}P NMR spectra of **9xa**

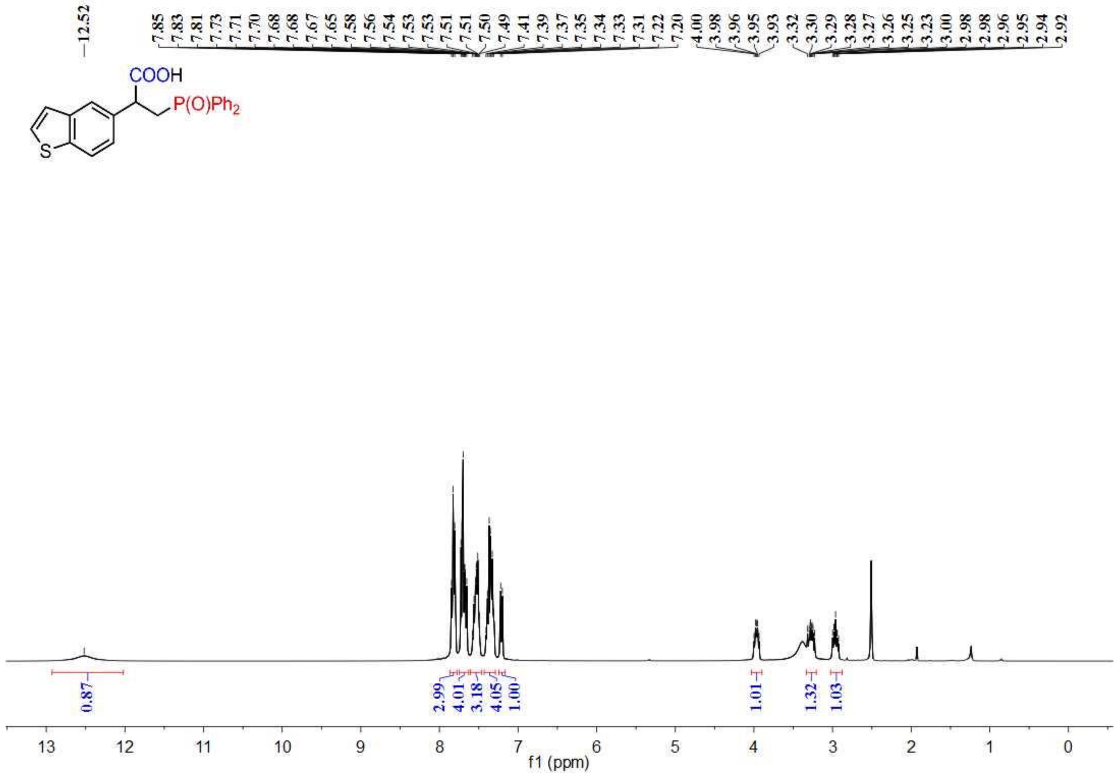

Supplementary Figure 207. ^{1}H NMR spectra of **9ya**

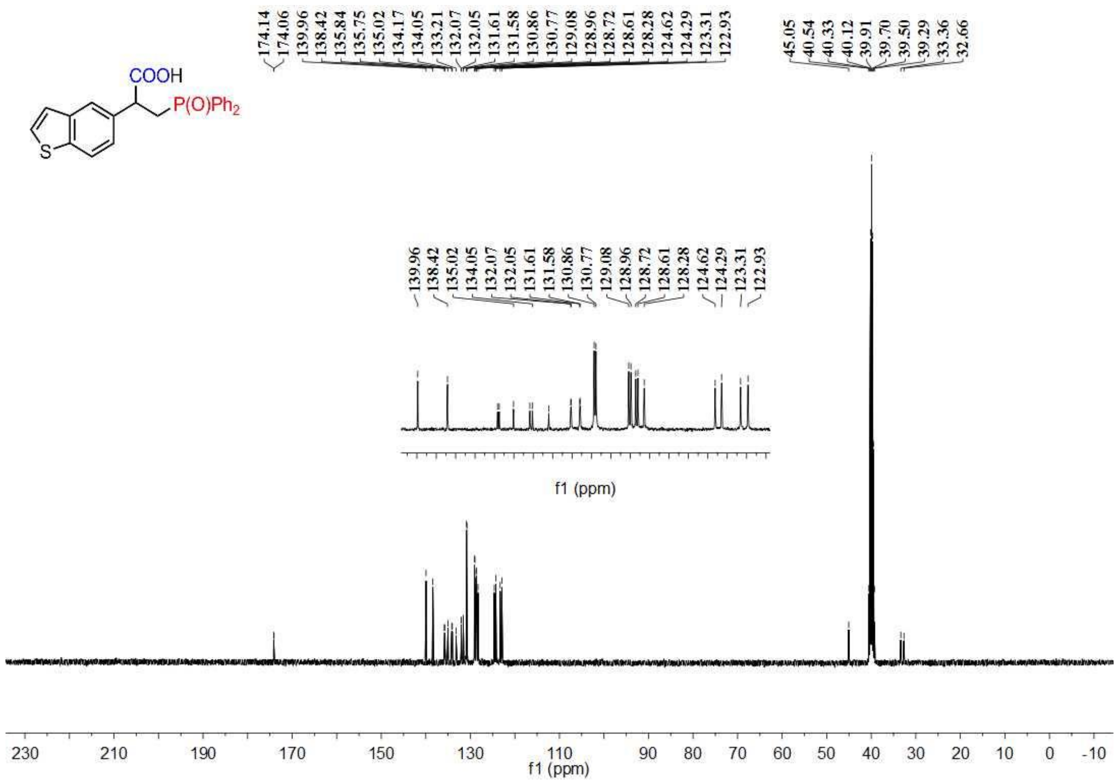

Supplementary Figure 208. ^{13}C NMR spectra of **9ya**

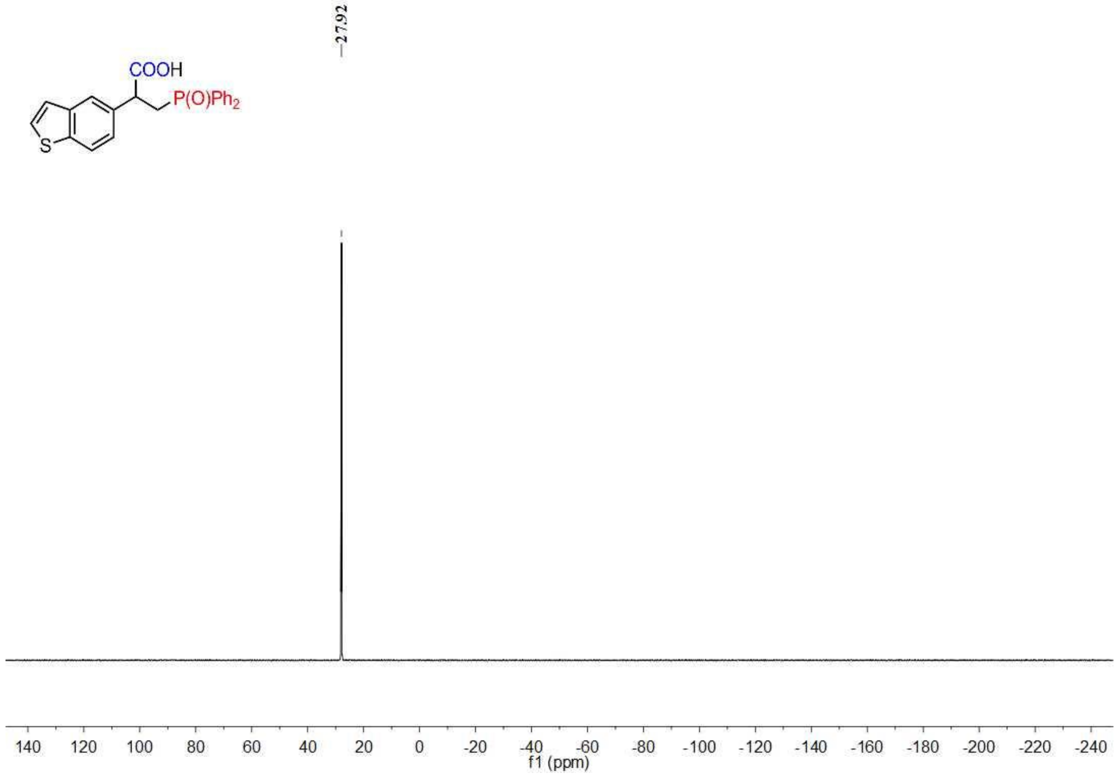

Supplementary Figure 209. ^{31}P NMR spectra of **9ya**

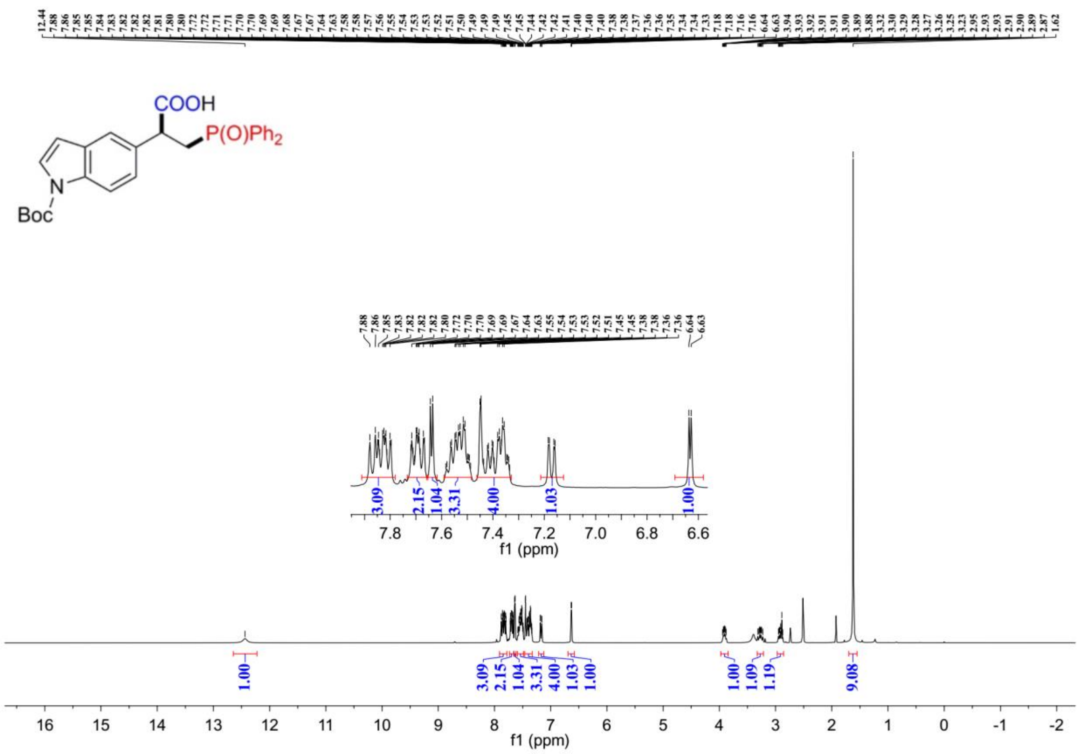

Supplementary Figure 210. ^{1}H NMR spectra of **9za**

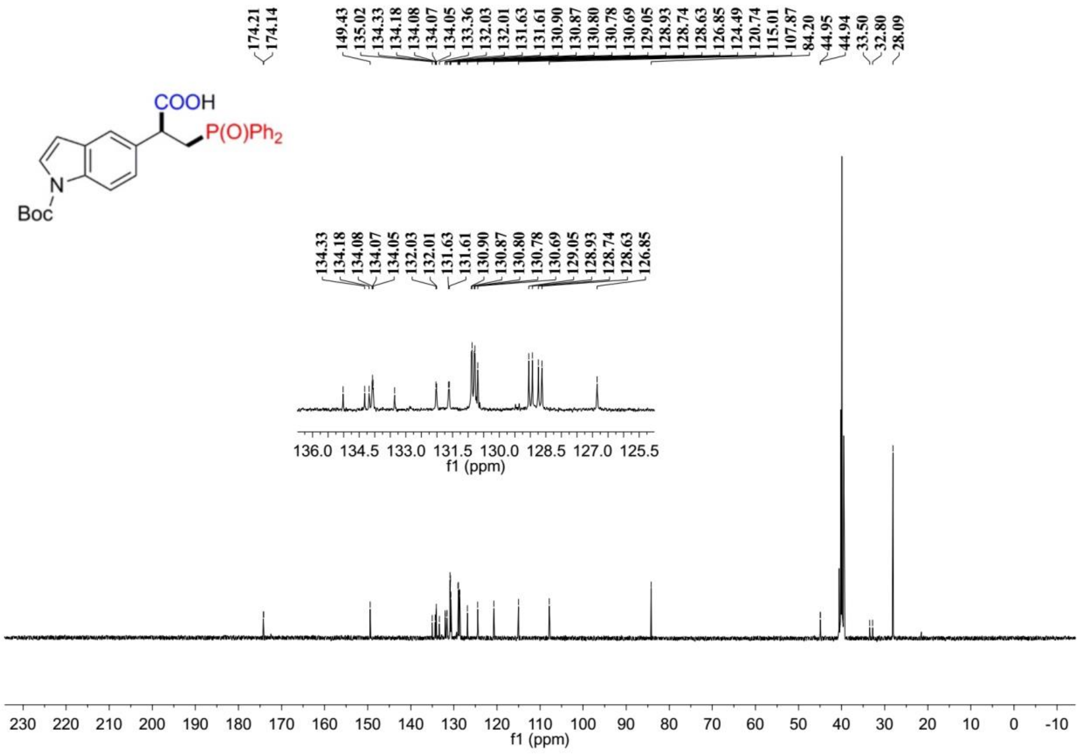

Supplementary Figure 211. ^{13}C NMR spectra of **9za**

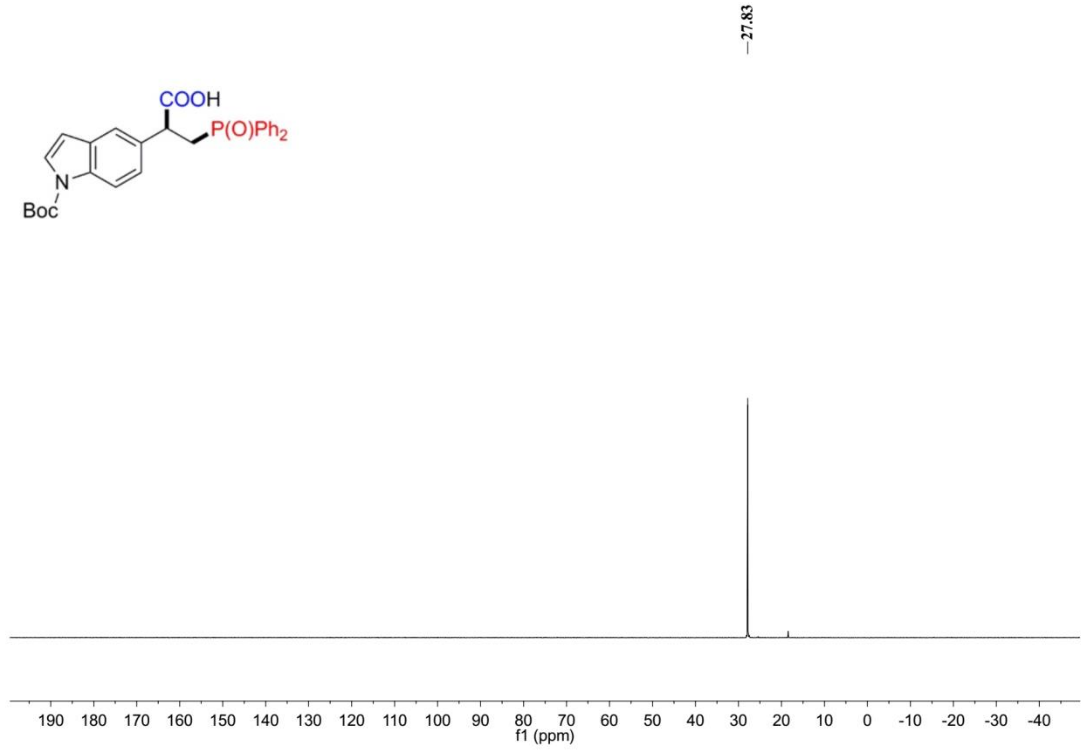

Supplementary Figure 212. ^{31}P NMR spectra of **9za**

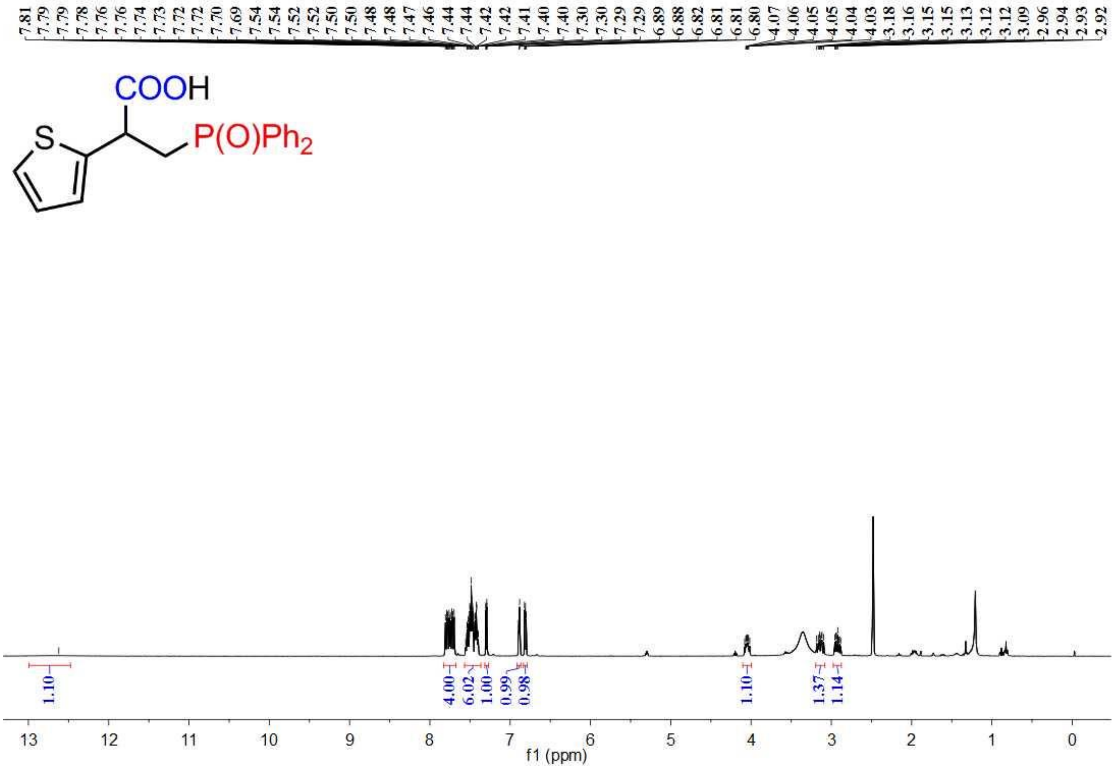

Supplementary Figure 213. ^{1}H NMR spectra of **9aaa**

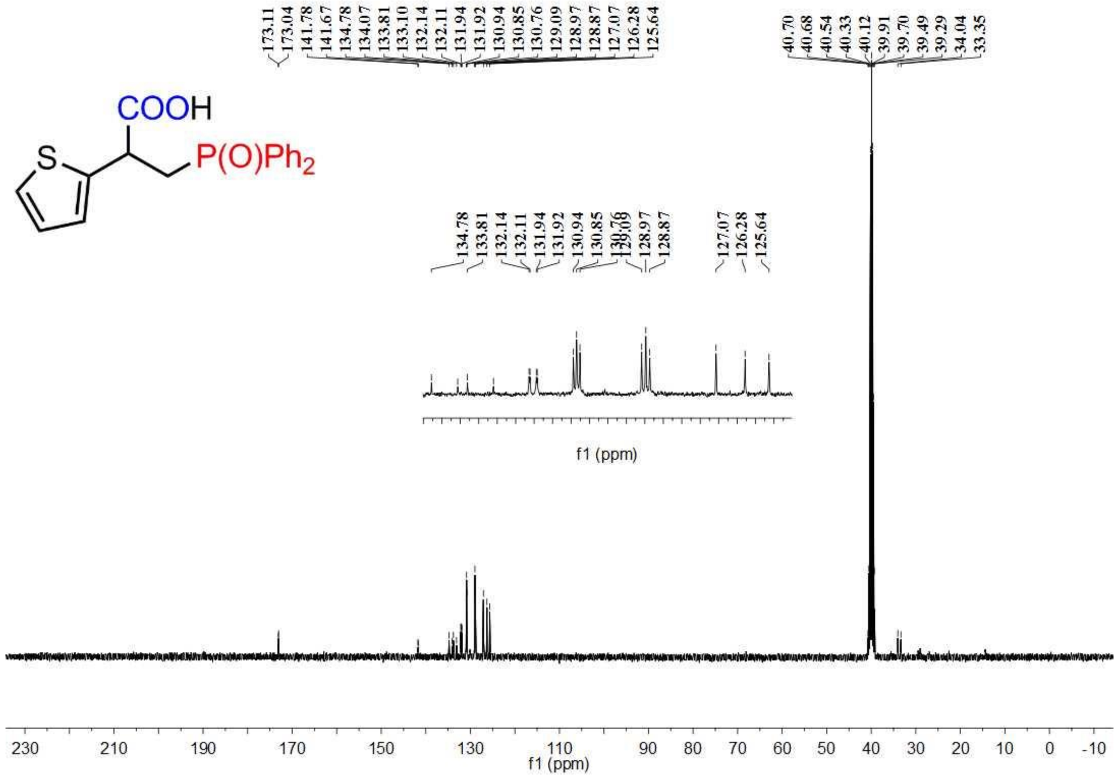

Supplementary Figure 214. ^{13}C NMR spectra of **9aaa**

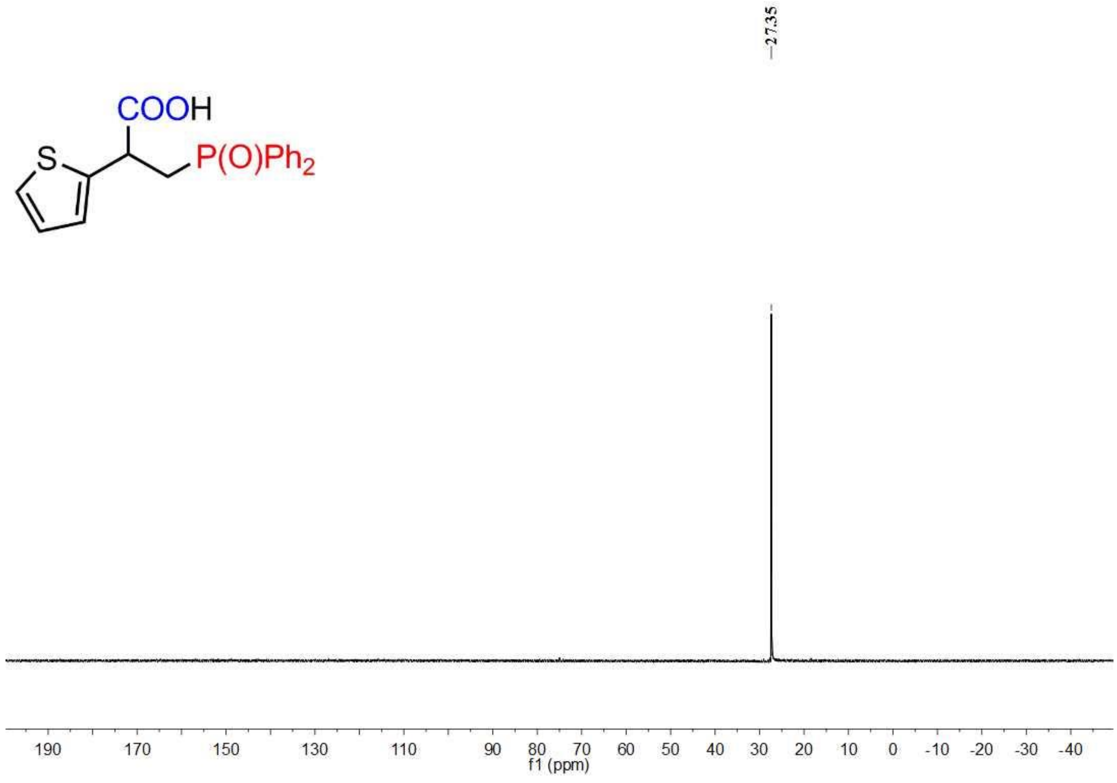

Supplementary Figure 215. ^{31}P NMR spectra of **9aaa**

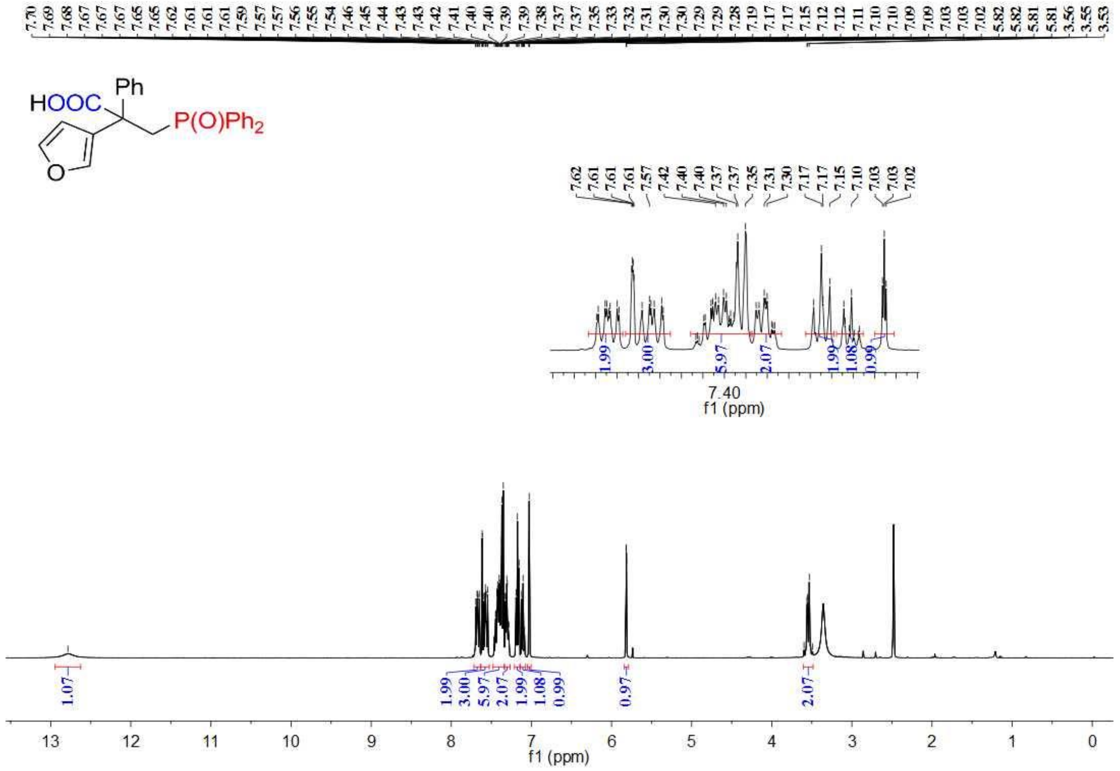

Supplementary Figure 216. ^{1}H NMR spectra of **9aba**

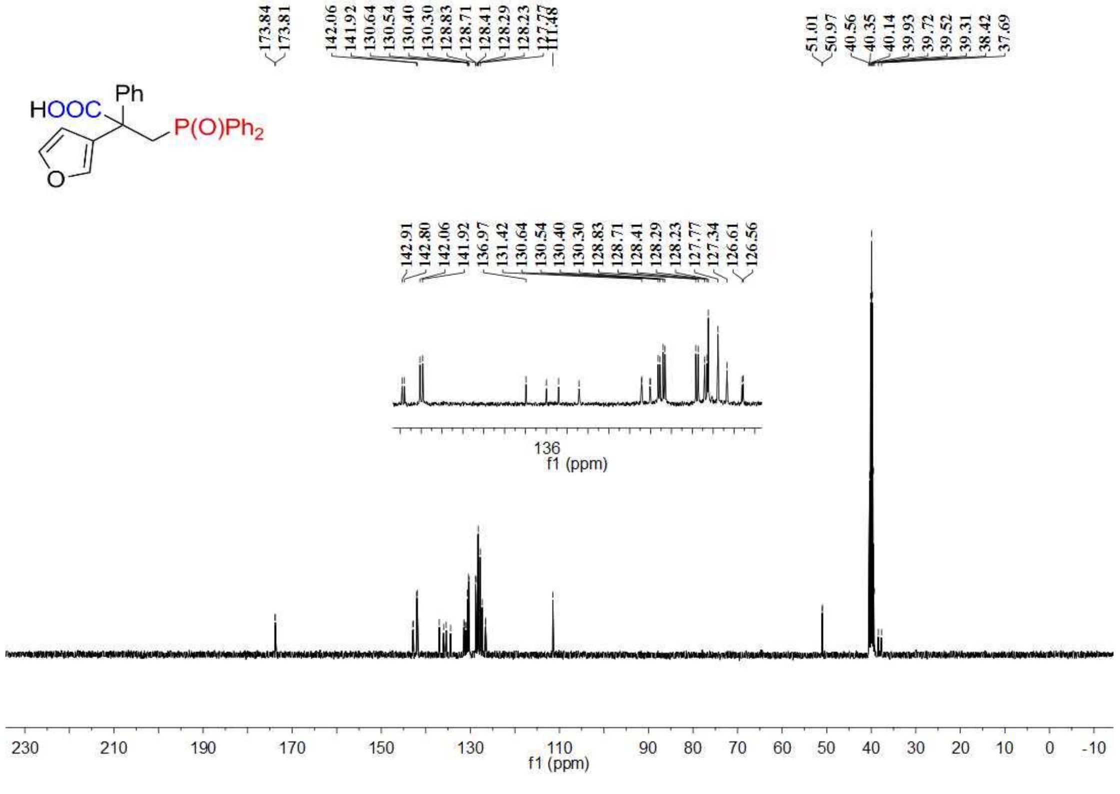

Supplementary Figure 217. ^{13}C NMR spectra of **9aba**

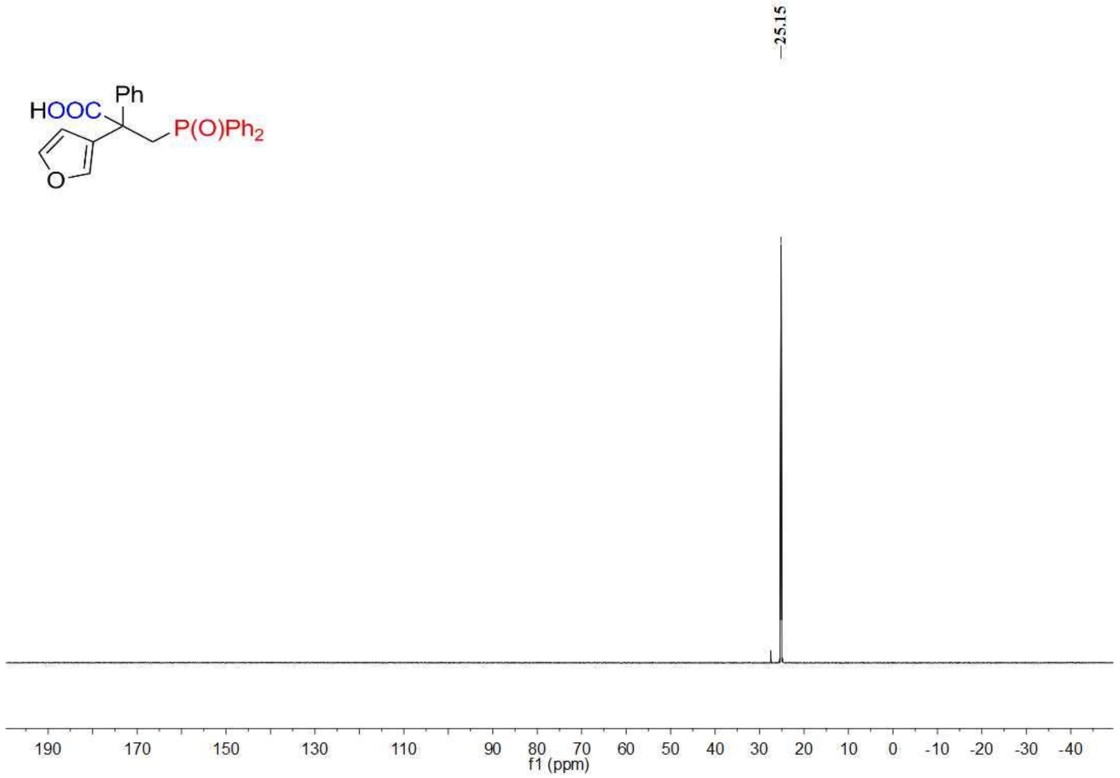

Supplementary Figure 218. ^{31}P NMR spectra of **9aba**

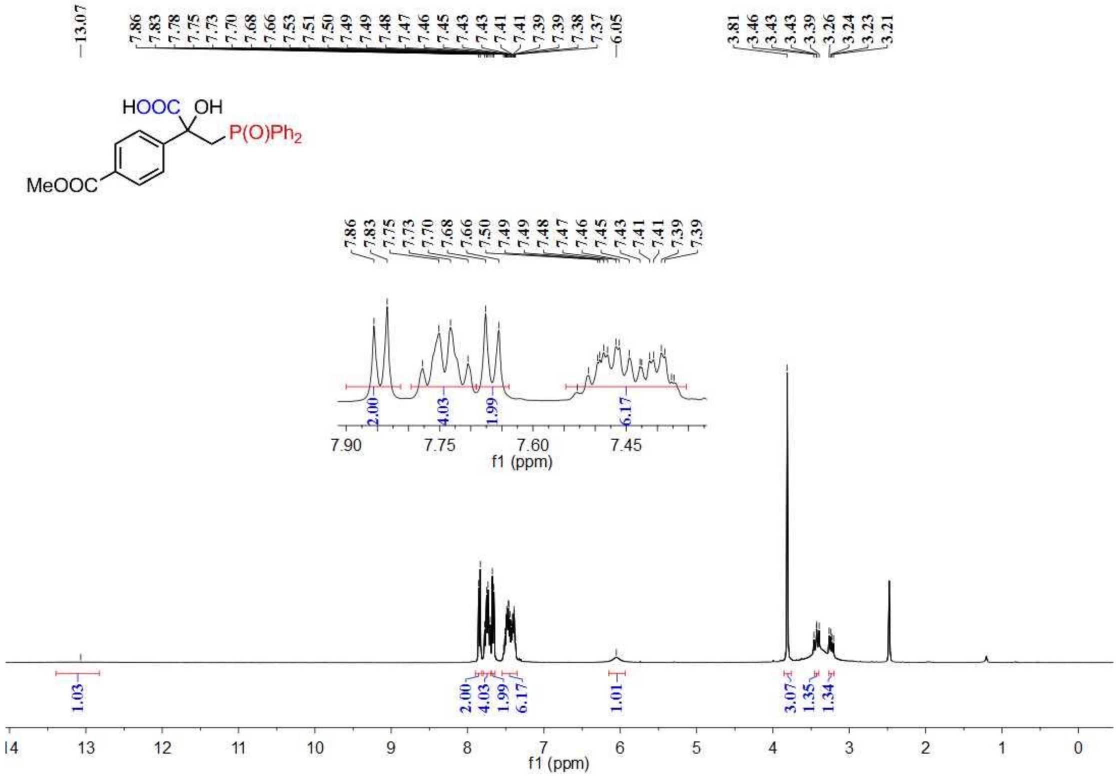

Supplementary Figure 219. ^{1}H NMR spectra of **9aca**

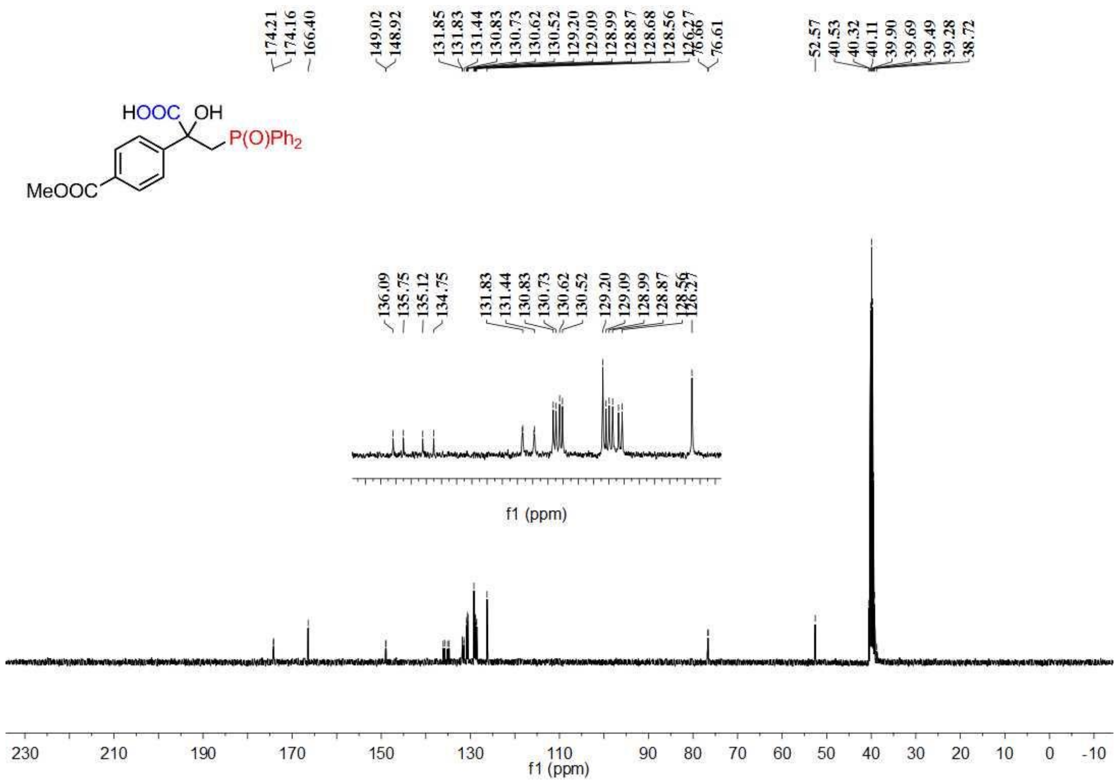

Supplementary Figure 220. ^{13}C NMR spectra of **9aca**

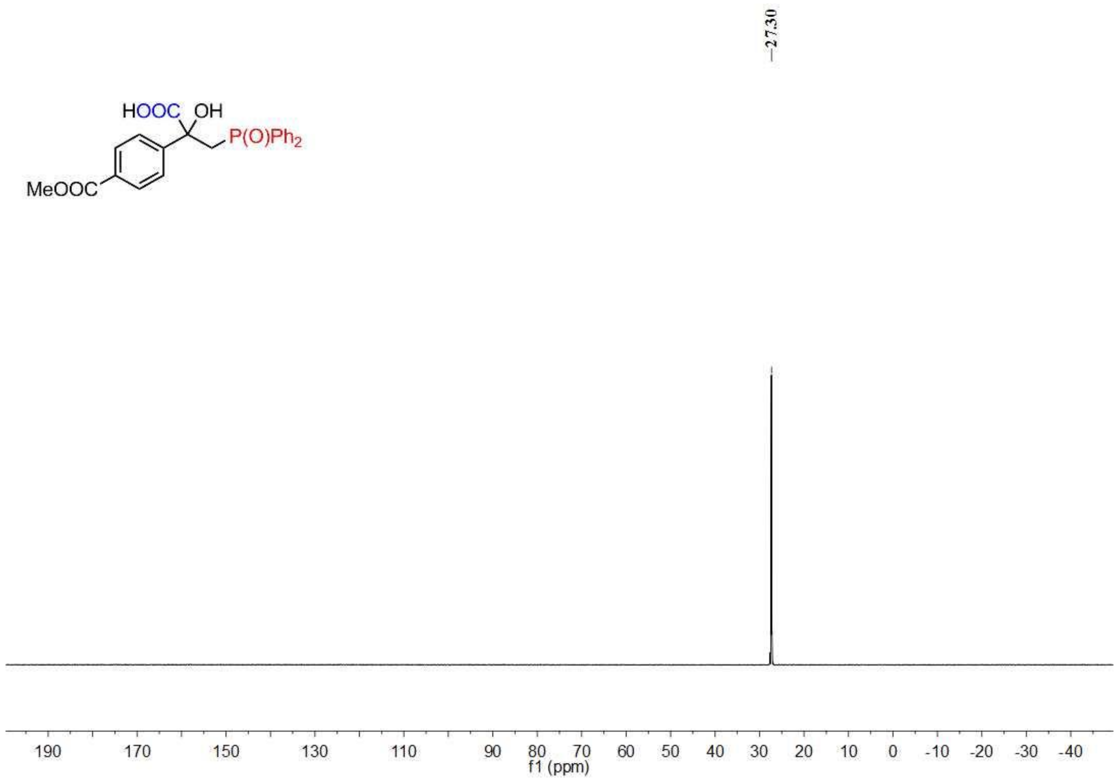

Supplementary Figure 221. ^{31}P NMR spectra of **9aca**

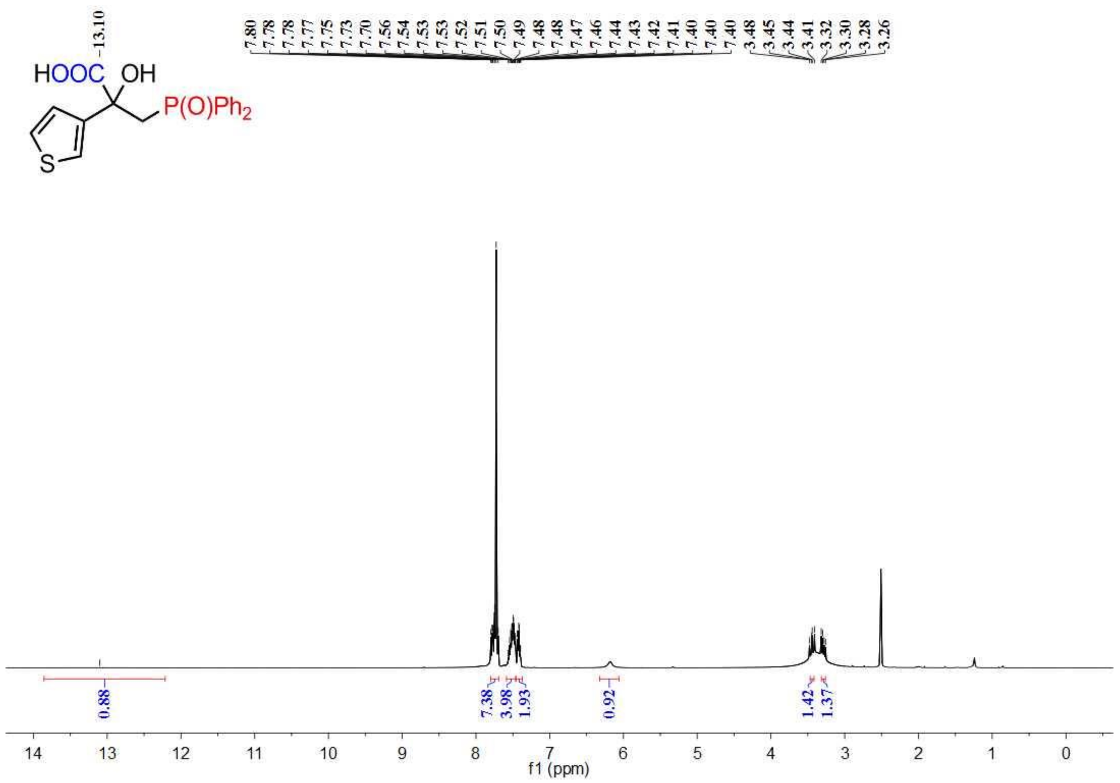

Supplementary Figure 222. ^{1}H NMR spectra of **9ada**

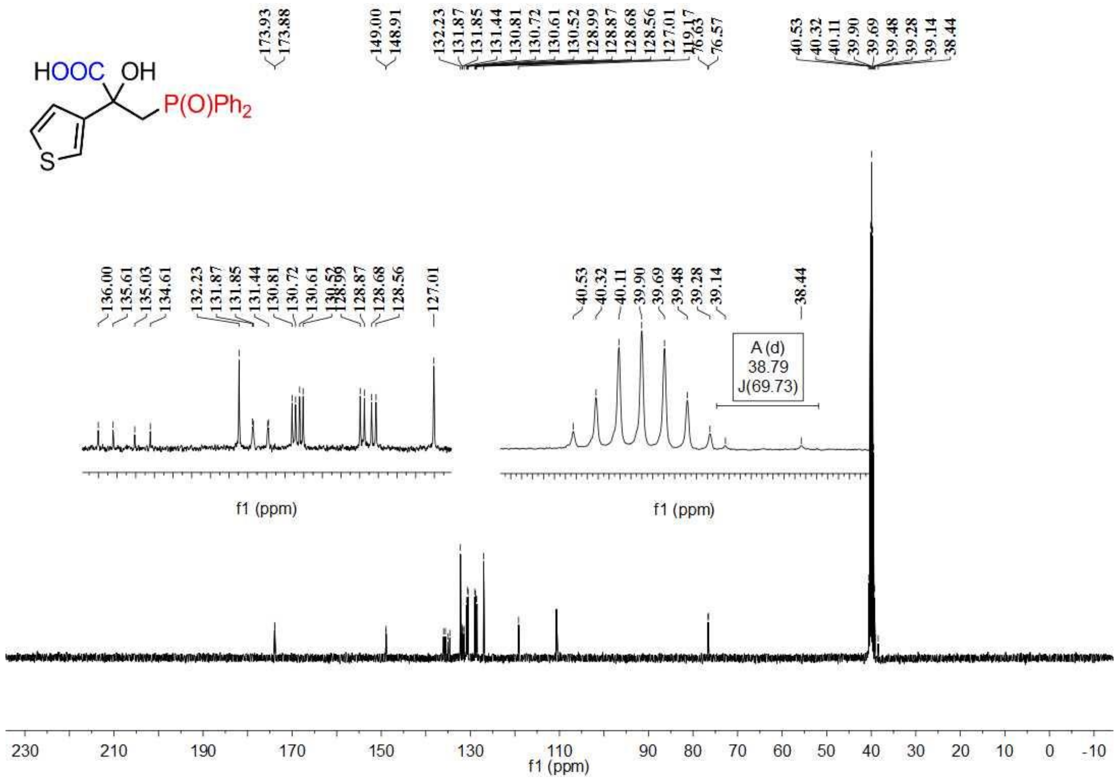

Supplementary Figure 223. ^{13}C NMR spectra of **9ada**

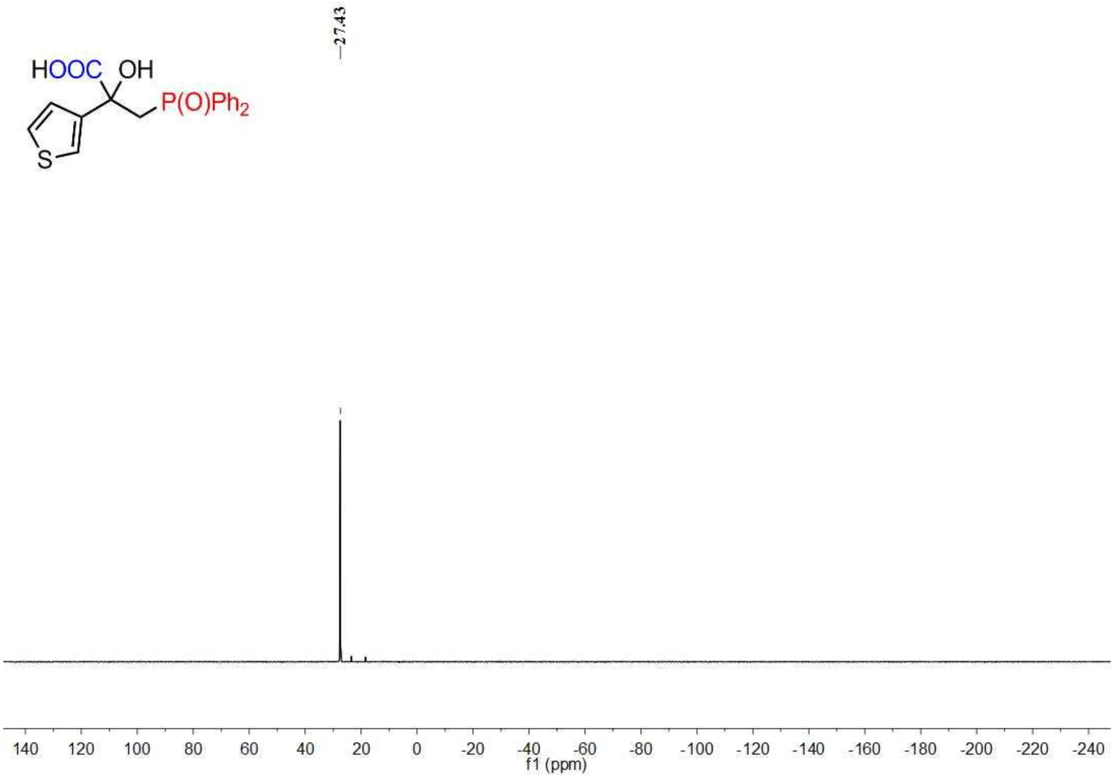

Supplementary Figure 224. ^{31}P NMR spectra of **9ada**

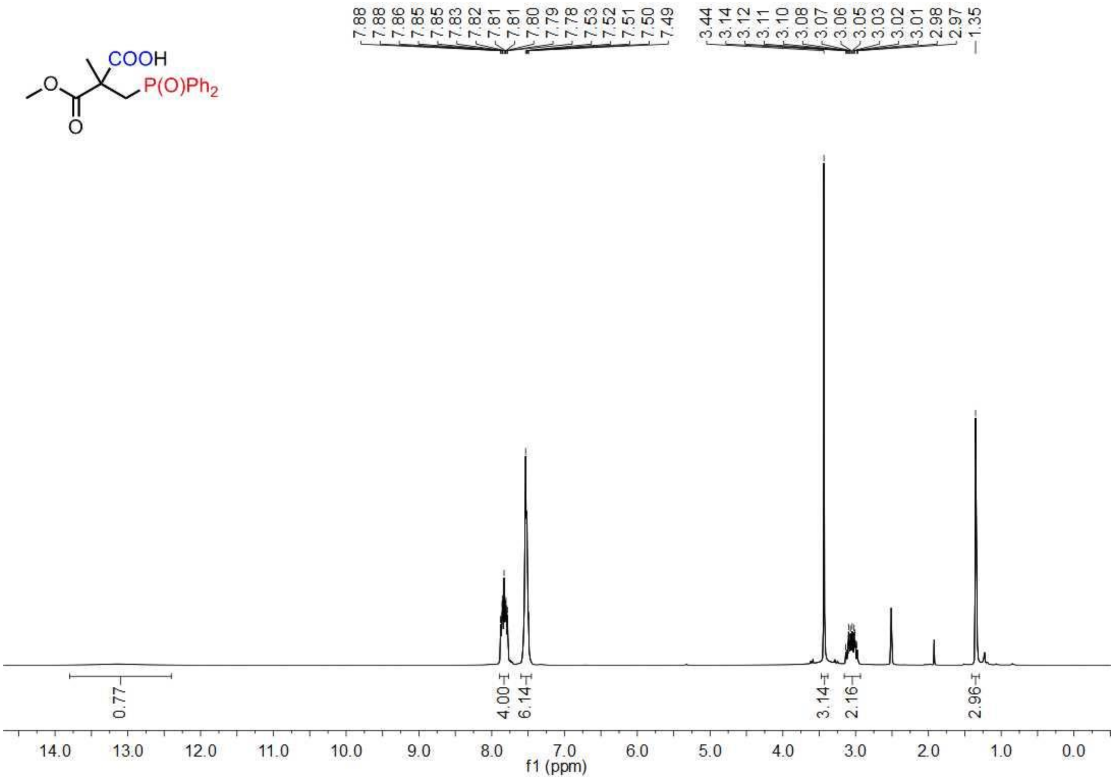

Supplementary Figure 225. ^{1}H NMR spectra of **11aa**

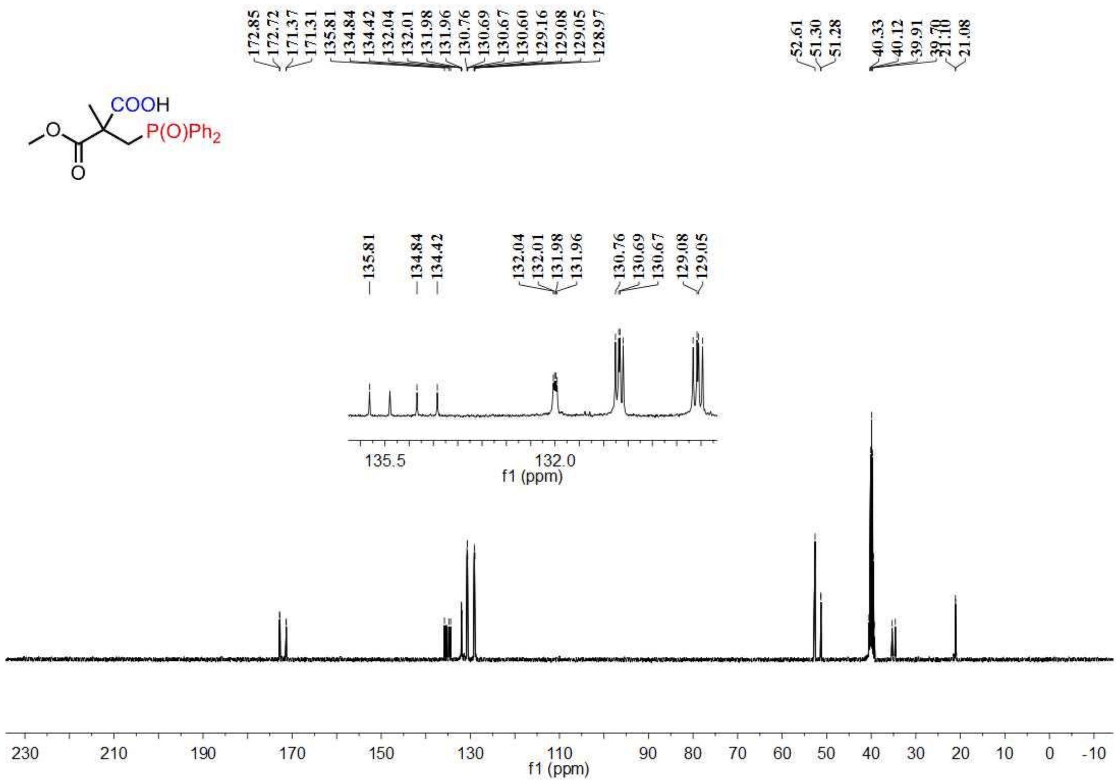

Supplementary Figure 226. ^{13}C NMR spectra of **11aa**

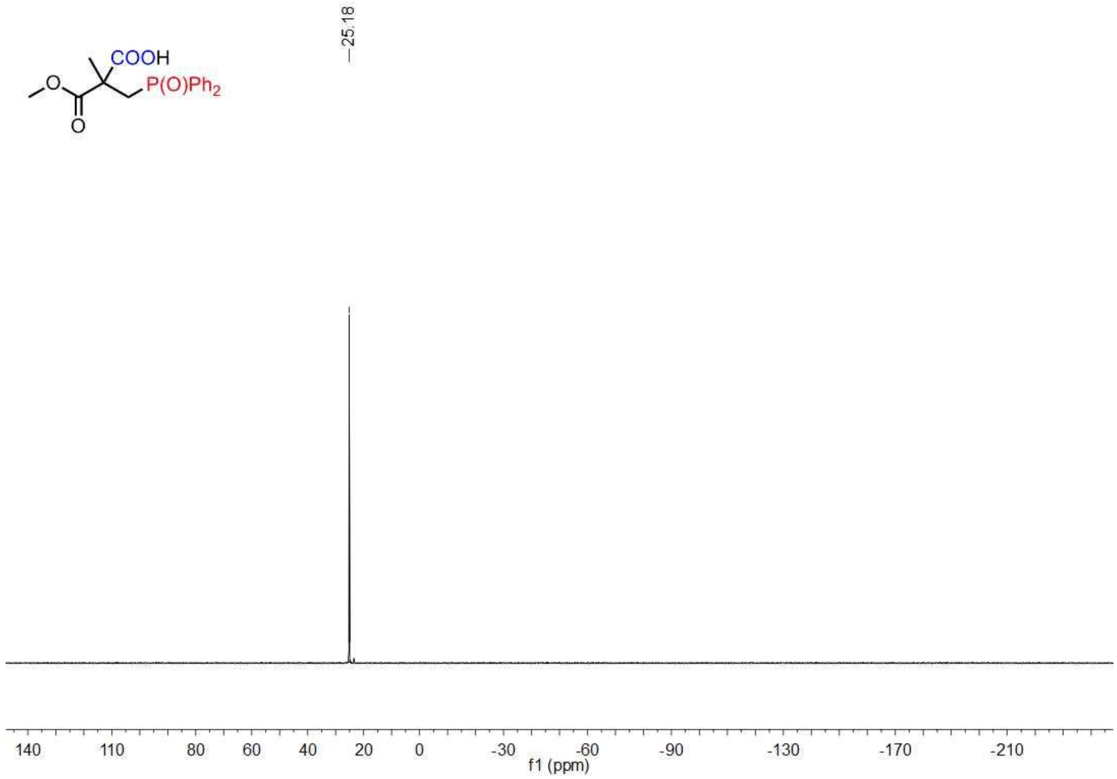

Supplementary Figure 227. ^{31}P NMR spectra of **11aa**

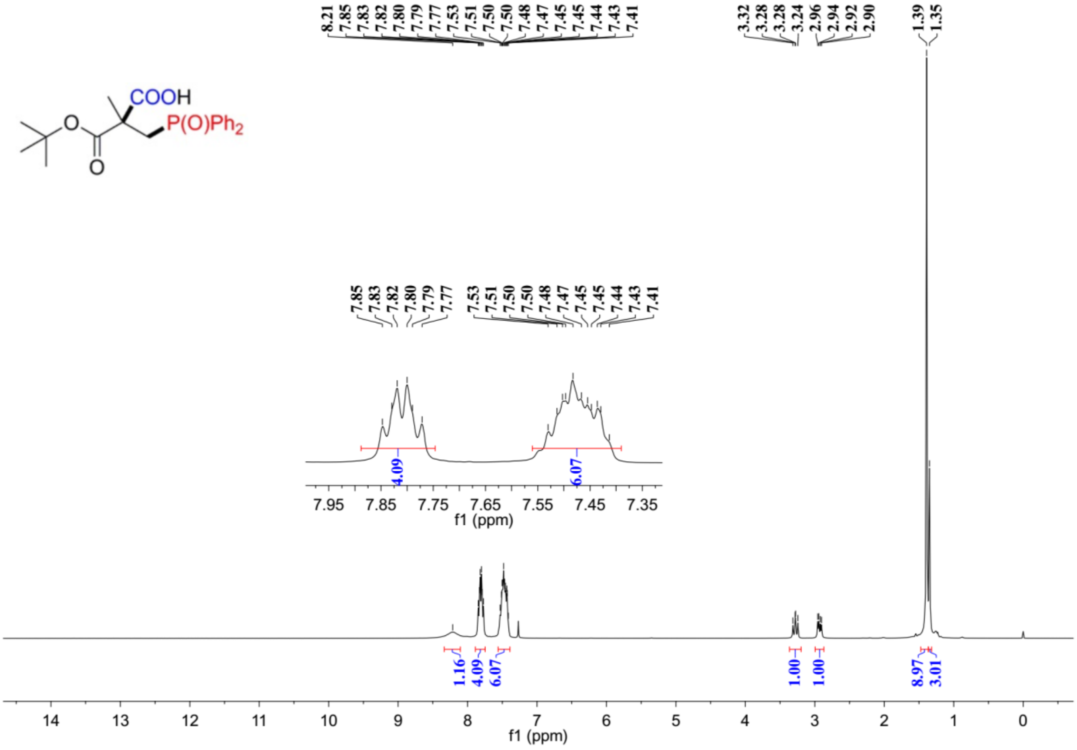

Supplementary Figure 228. ^{1}H NMR spectra of **11ba**

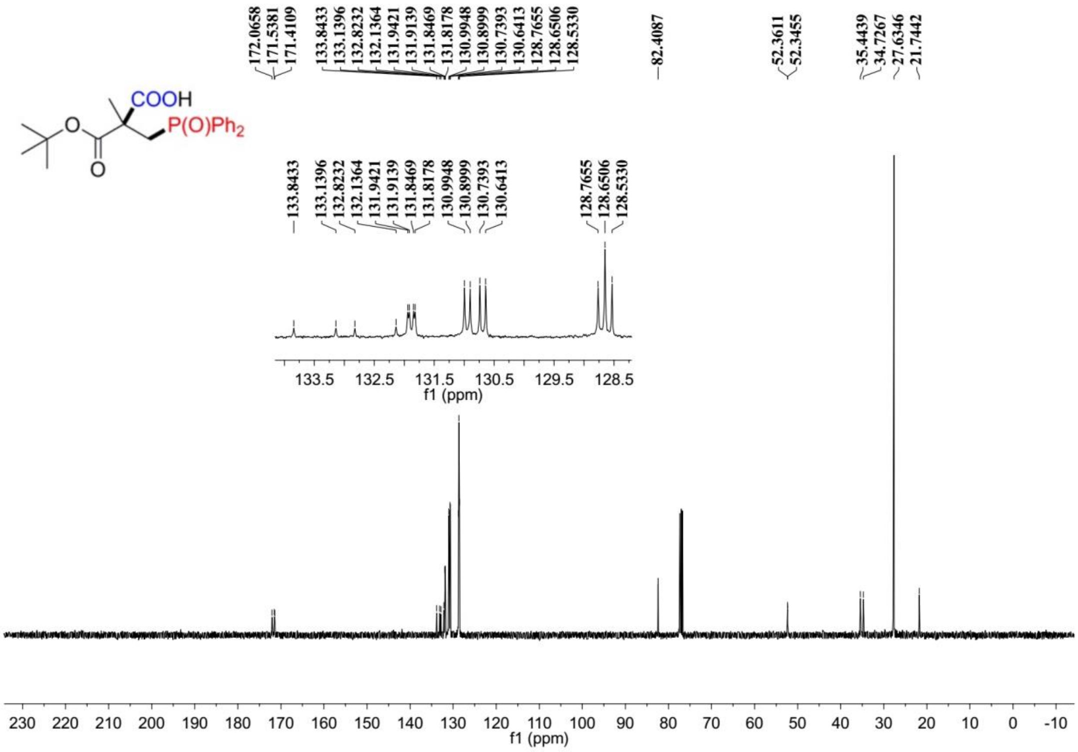

Supplementary Figure 229. ^{13}C NMR spectra of **11ba**

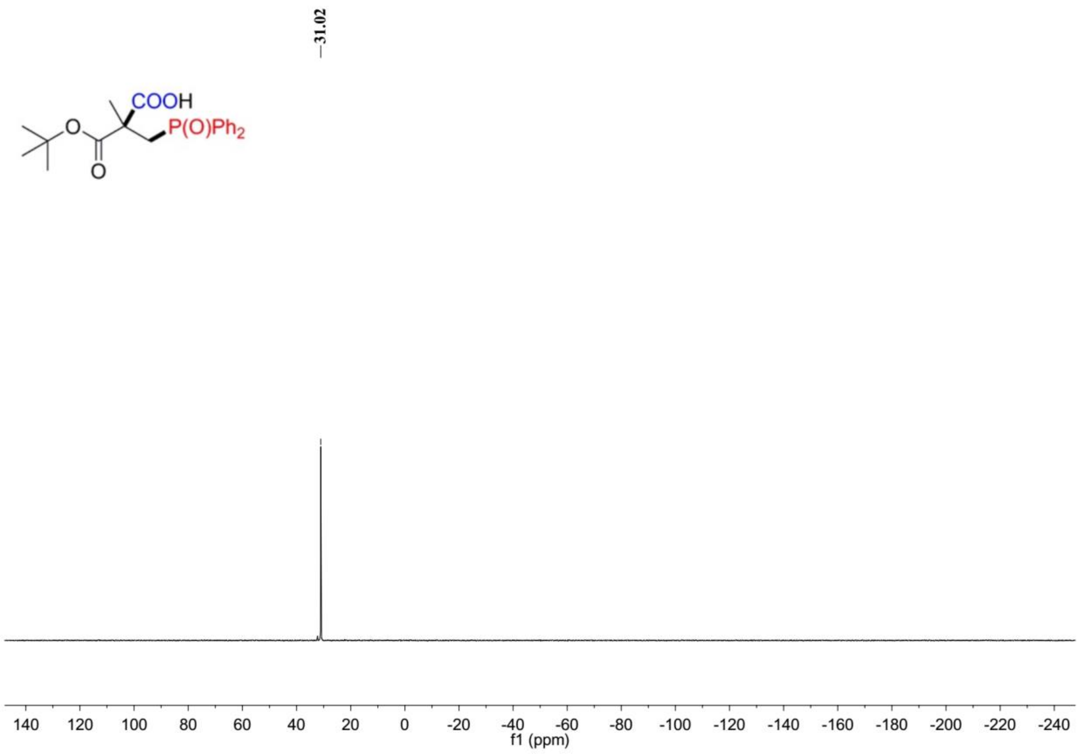

Supplementary Figure 230. ^{31}P NMR spectra of **11ba**

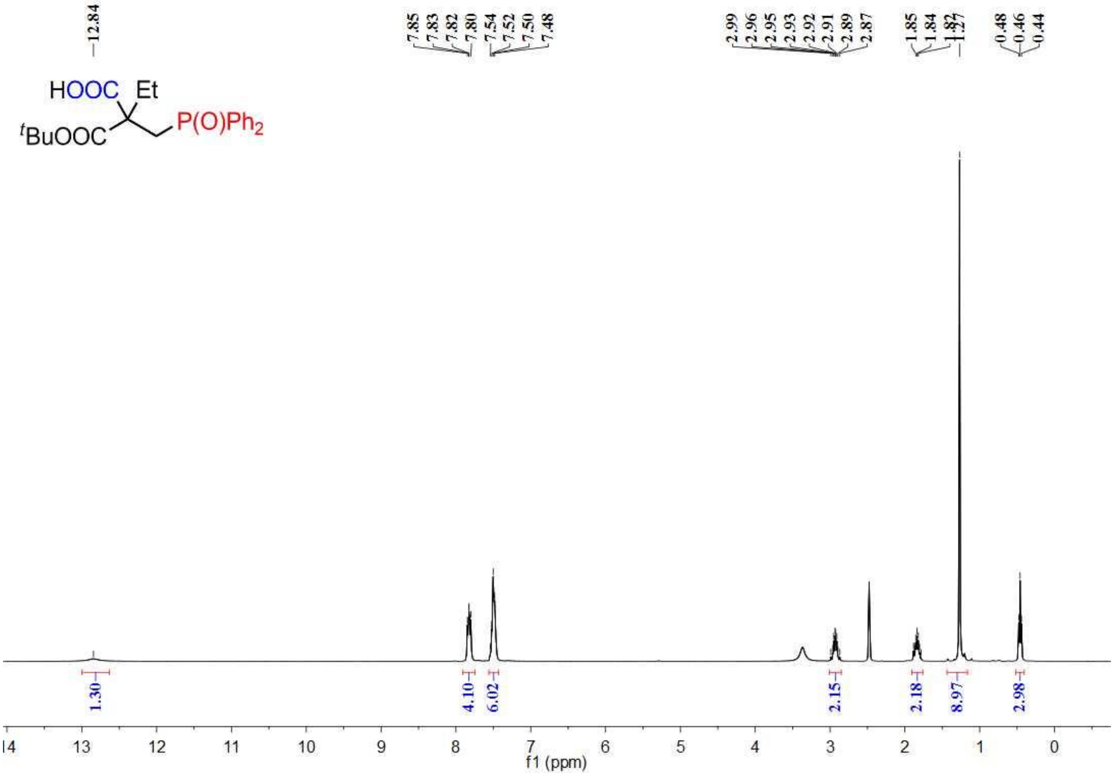

Supplementary Figure 231. ^{1}H NMR spectra of **11ca**

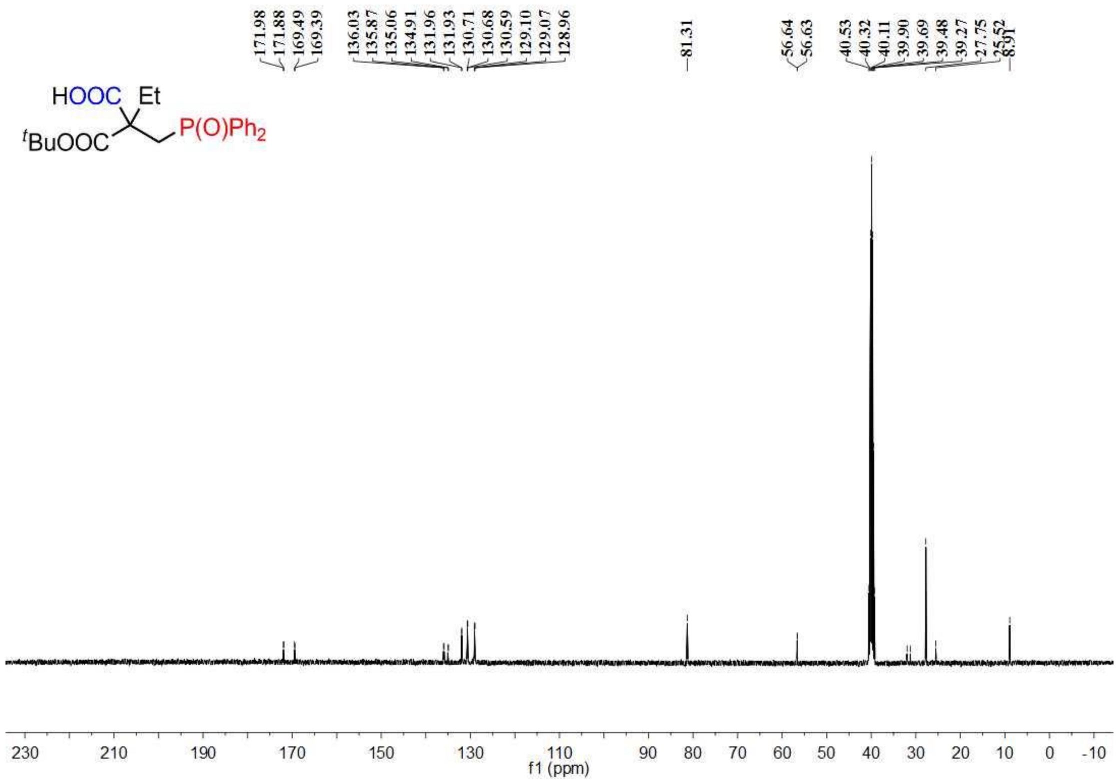

Supplementary Figure 232. ^{13}C NMR spectra of **11ca**

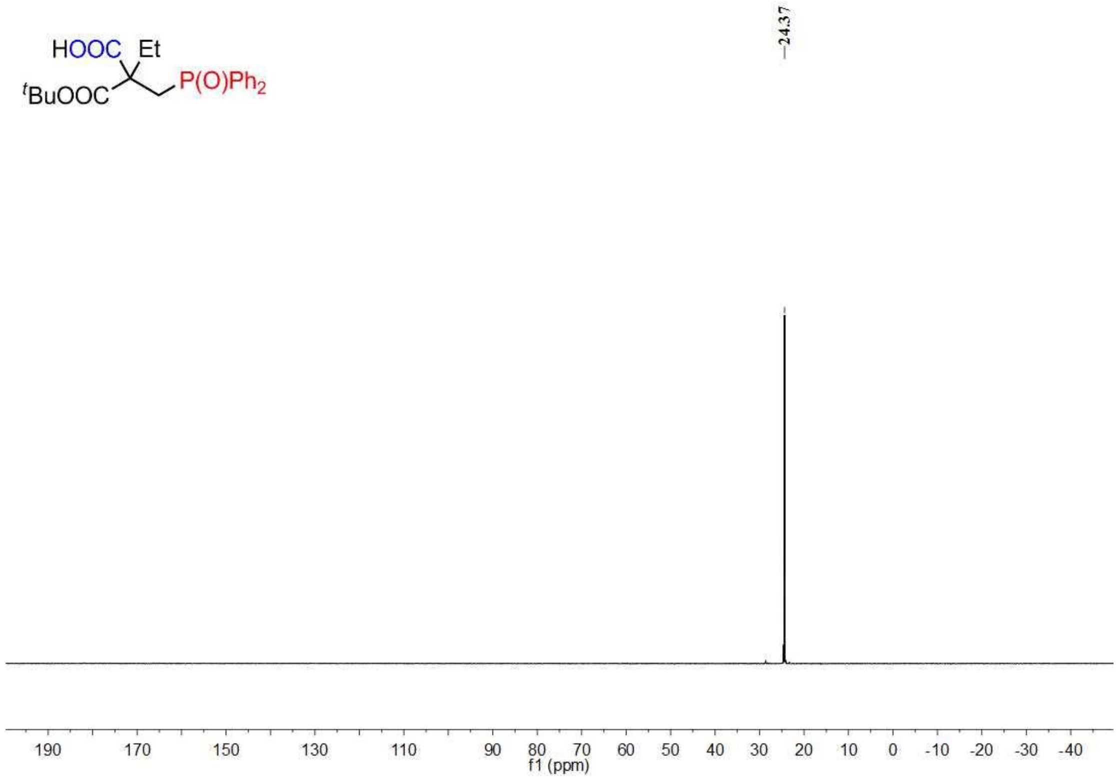

Supplementary Figure 233. ^{31}P NMR spectra of **11ca**

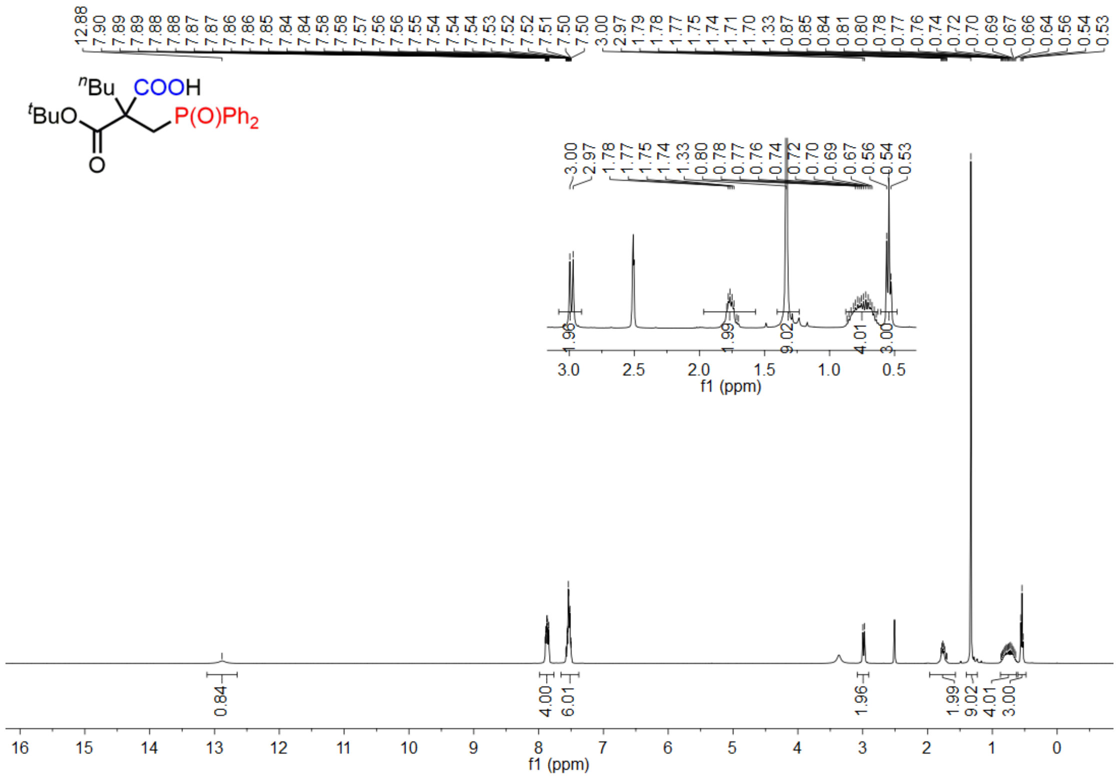

Supplementary Figure 234. ^{1}H NMR spectra of **11da**

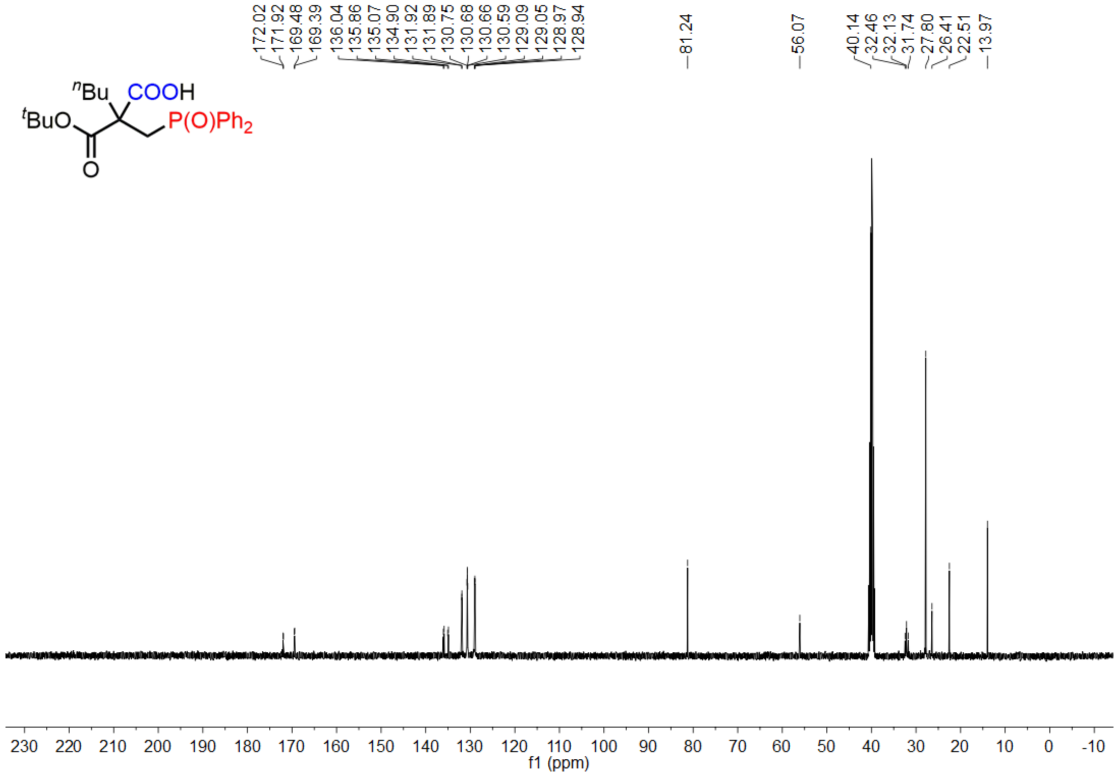

Supplementary Figure 235. ^{13}C NMR spectra of **11da**

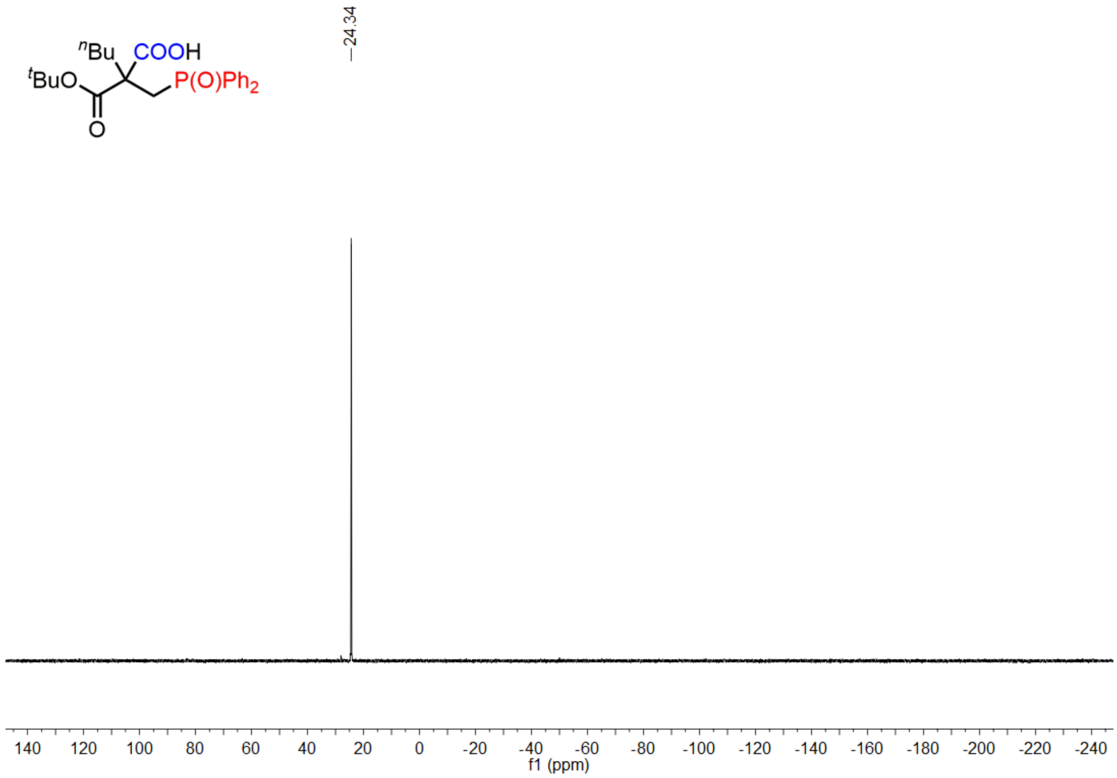

Supplementary Figure 236. ^{31}P NMR spectra of **11da**

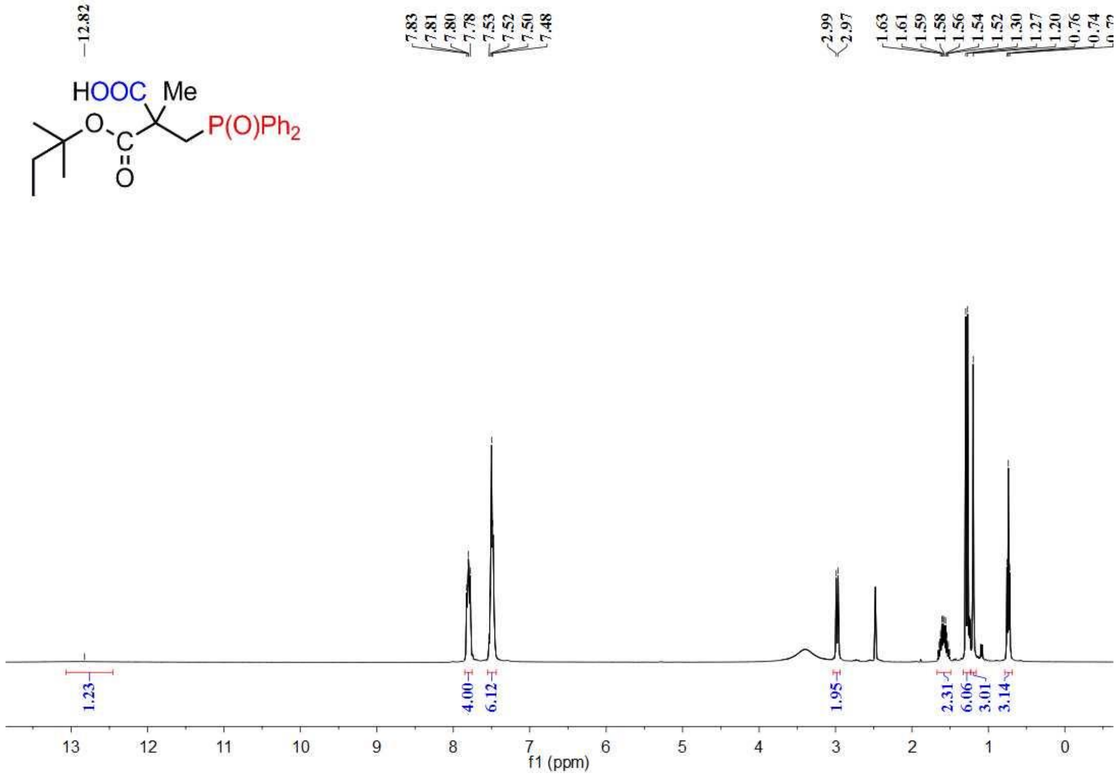

Supplementary Figure 237. ^{1}H NMR spectra of **11ea**

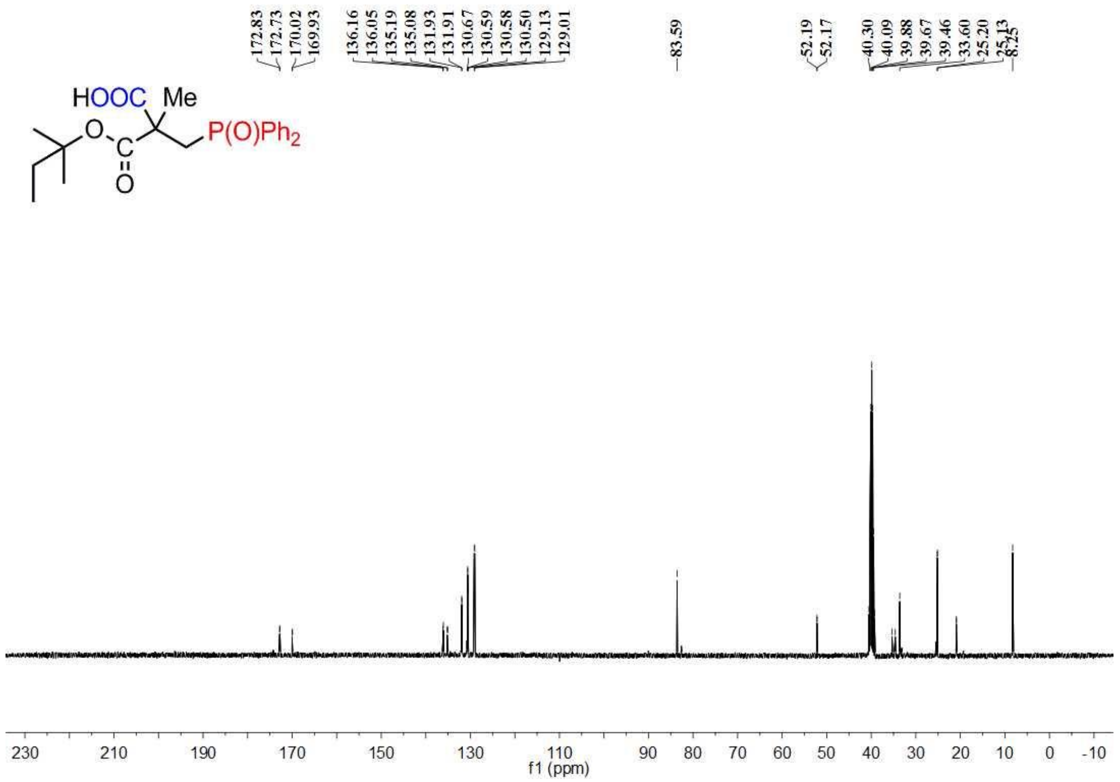

Supplementary Figure 238. ^{13}C NMR spectra of **11ea**

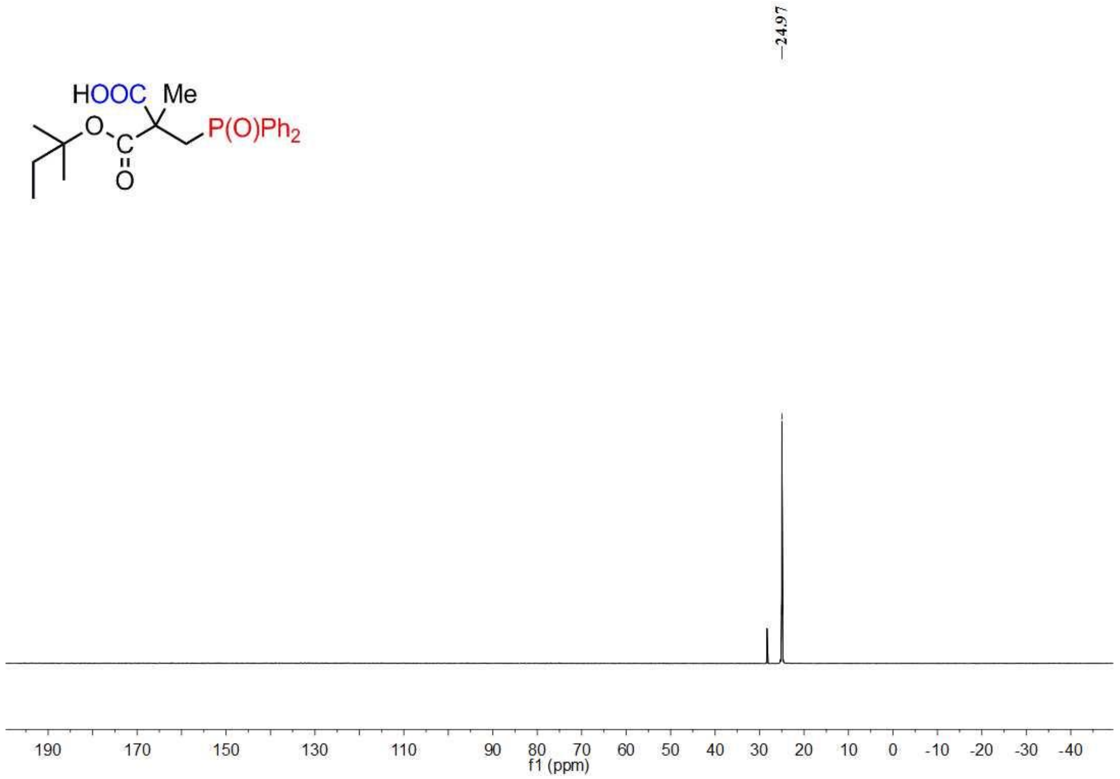

Supplementary Figure 239. ^{31}P NMR spectra of **11ea**

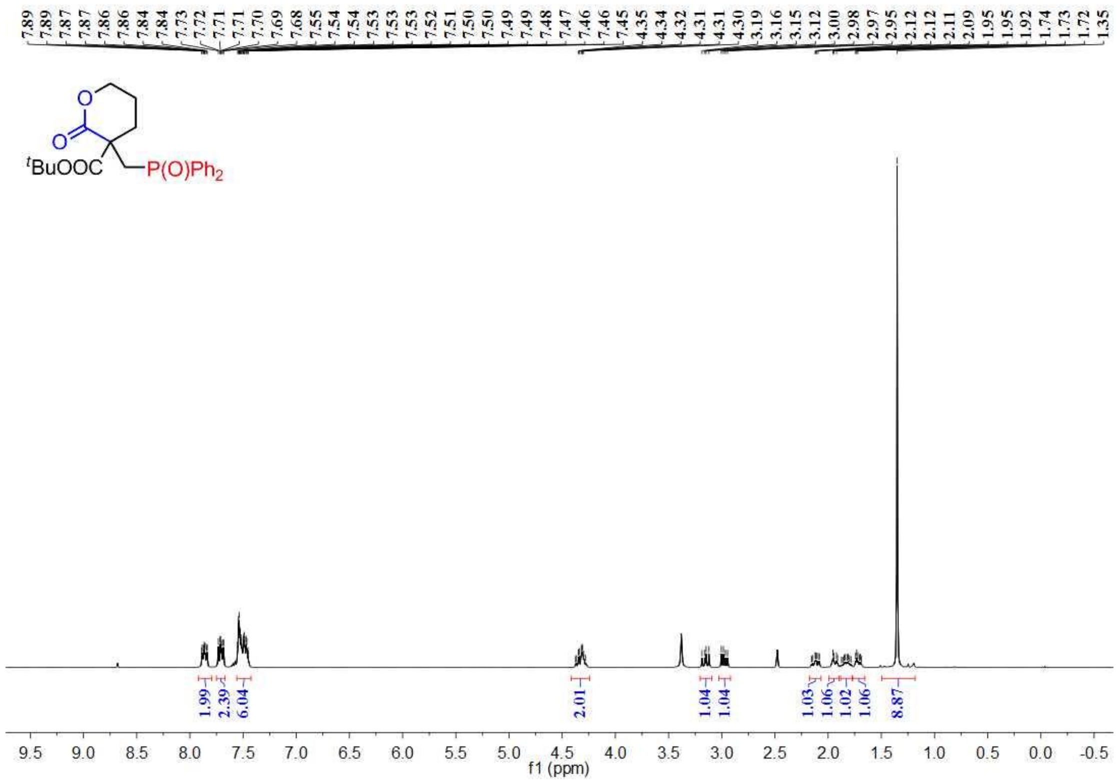

Supplementary Figure 240. ^{1}H NMR spectra of **11fa**

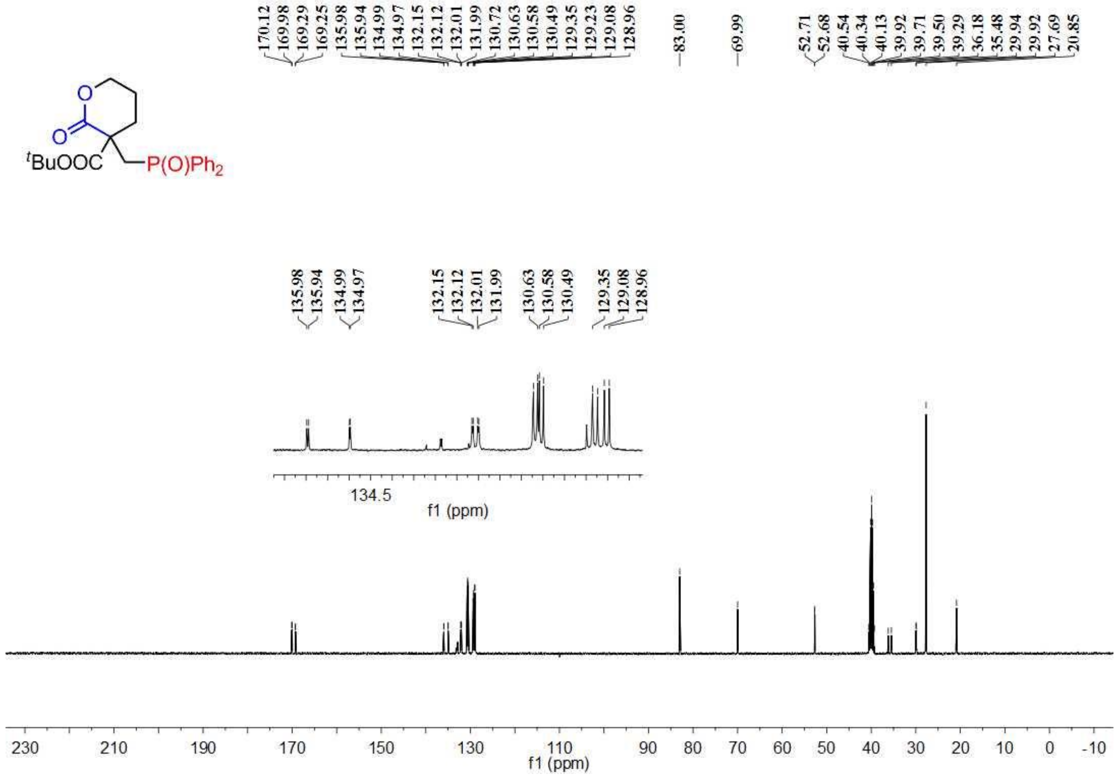

Supplementary Figure 241. ^{13}C NMR spectra of **11fa**

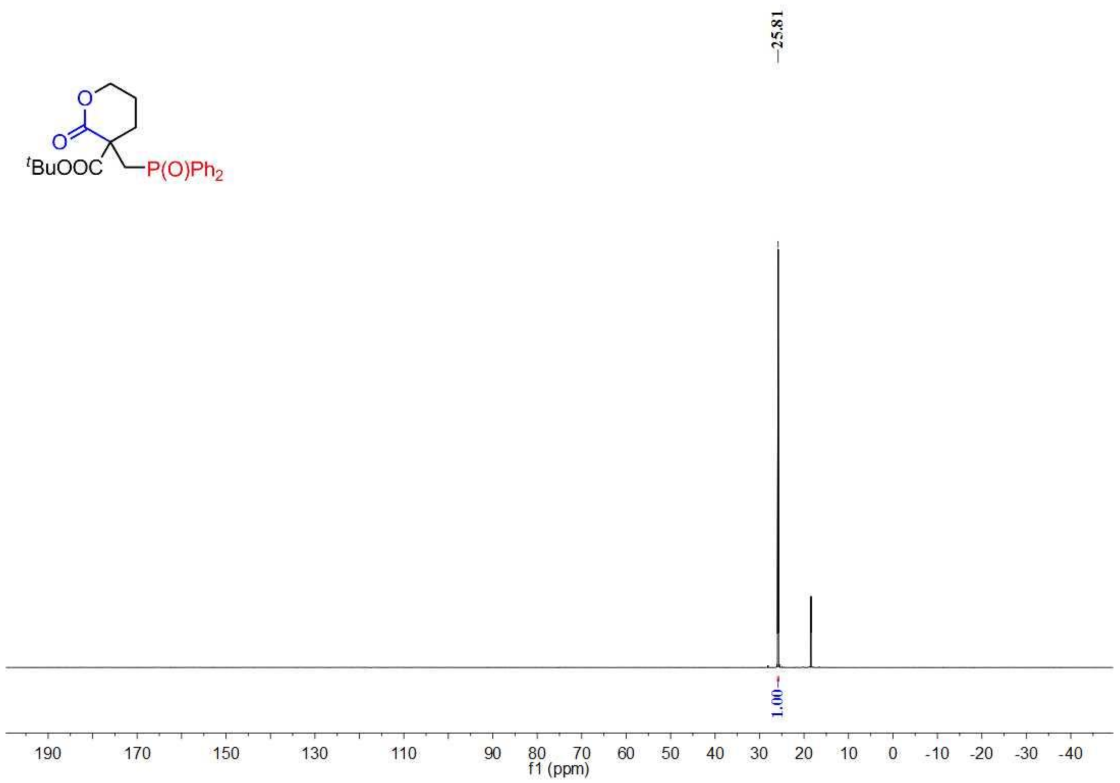

Supplementary Figure 242. ^{31}P NMR spectra of **11fa**

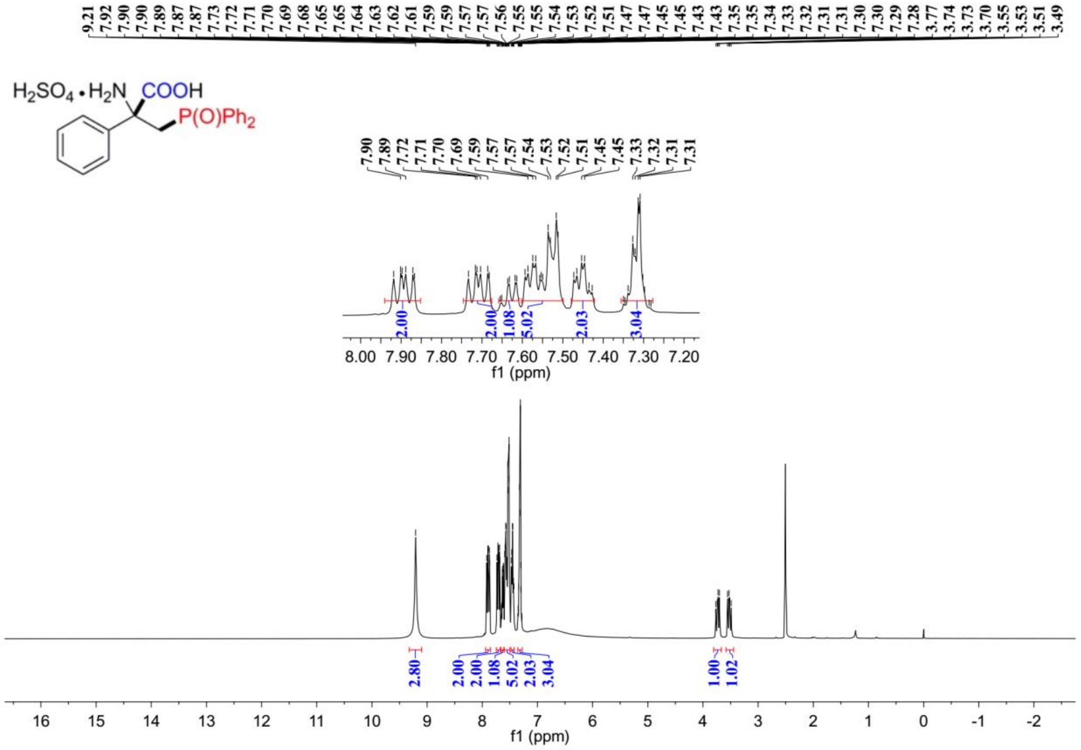

Supplementary Figure 243. ^{1}H NMR spectra of **12**

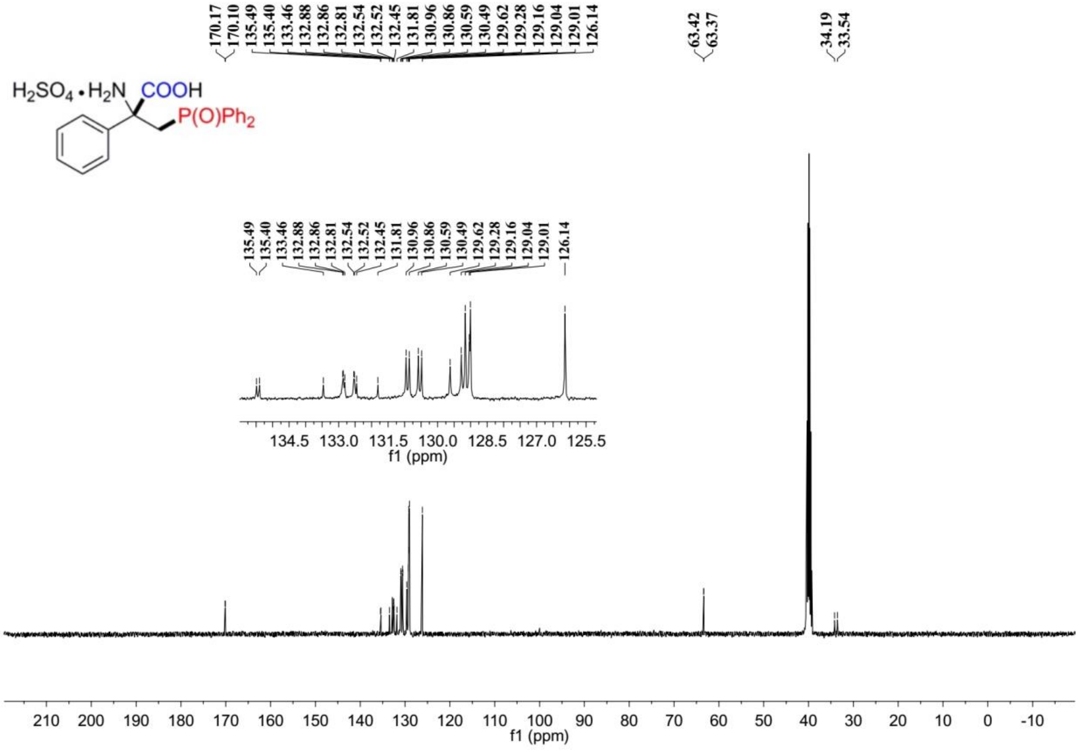

Supplementary Figure 244. ^{13}C NMR spectra of **12**

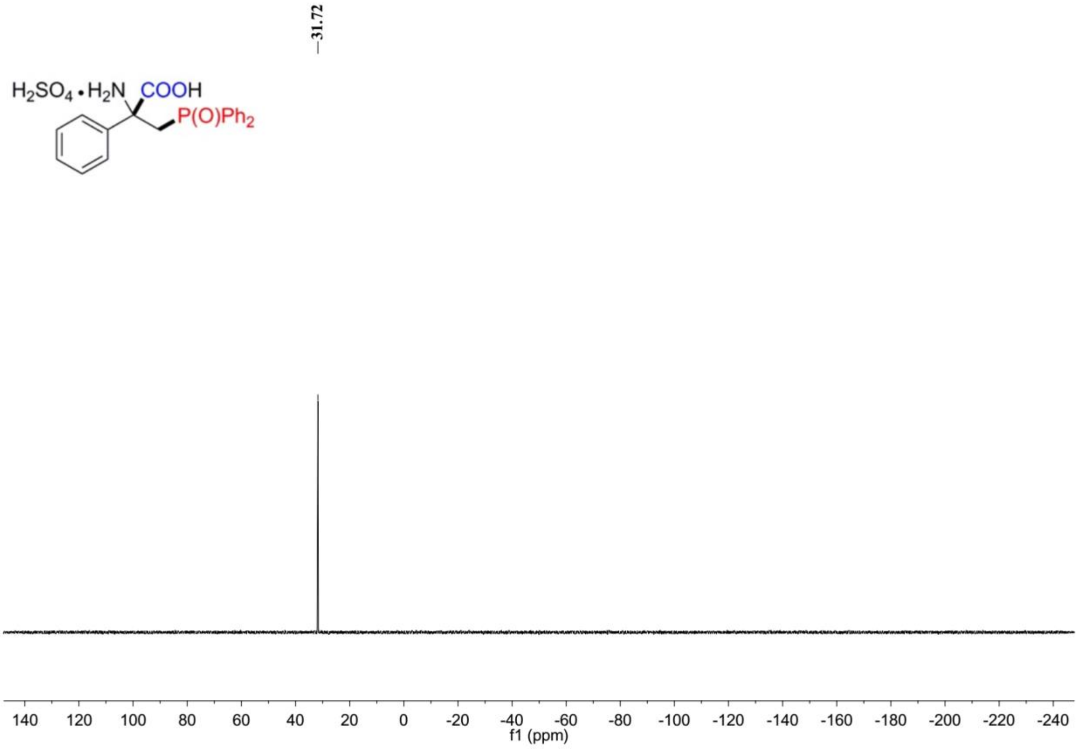

Supplementary Figure 245. ^{31}P NMR spectra of **12**

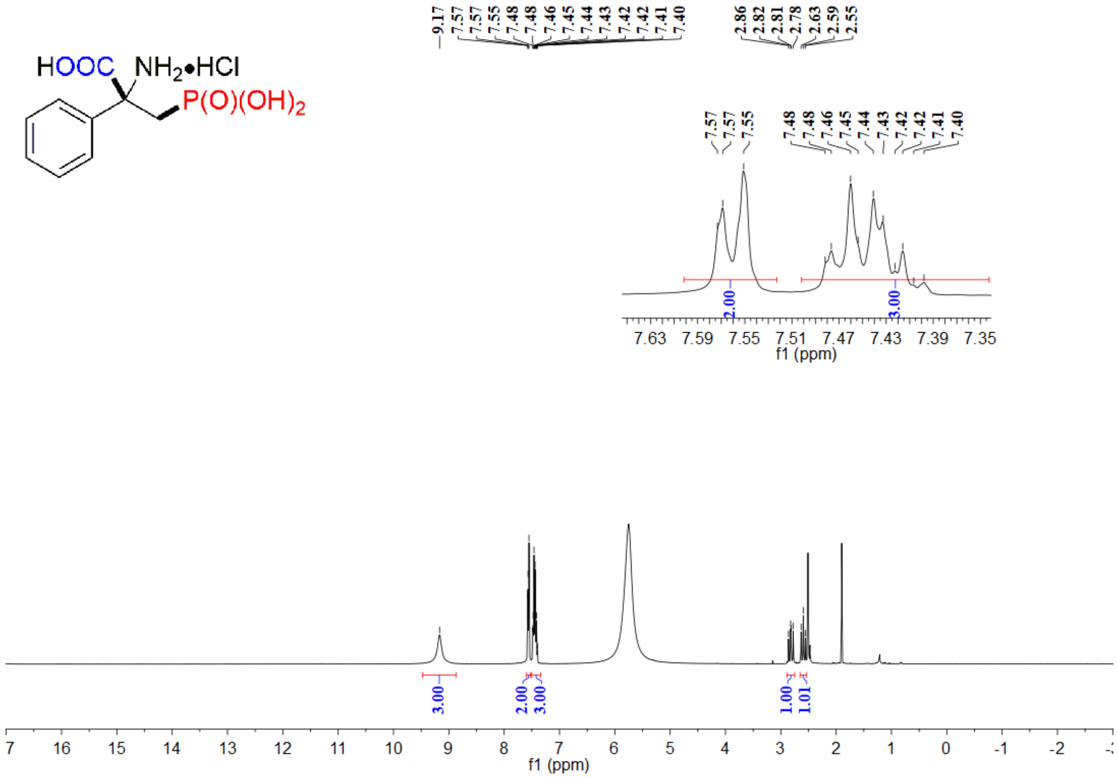

Supplementary Figure 246. ^{1}H NMR spectra of **13**

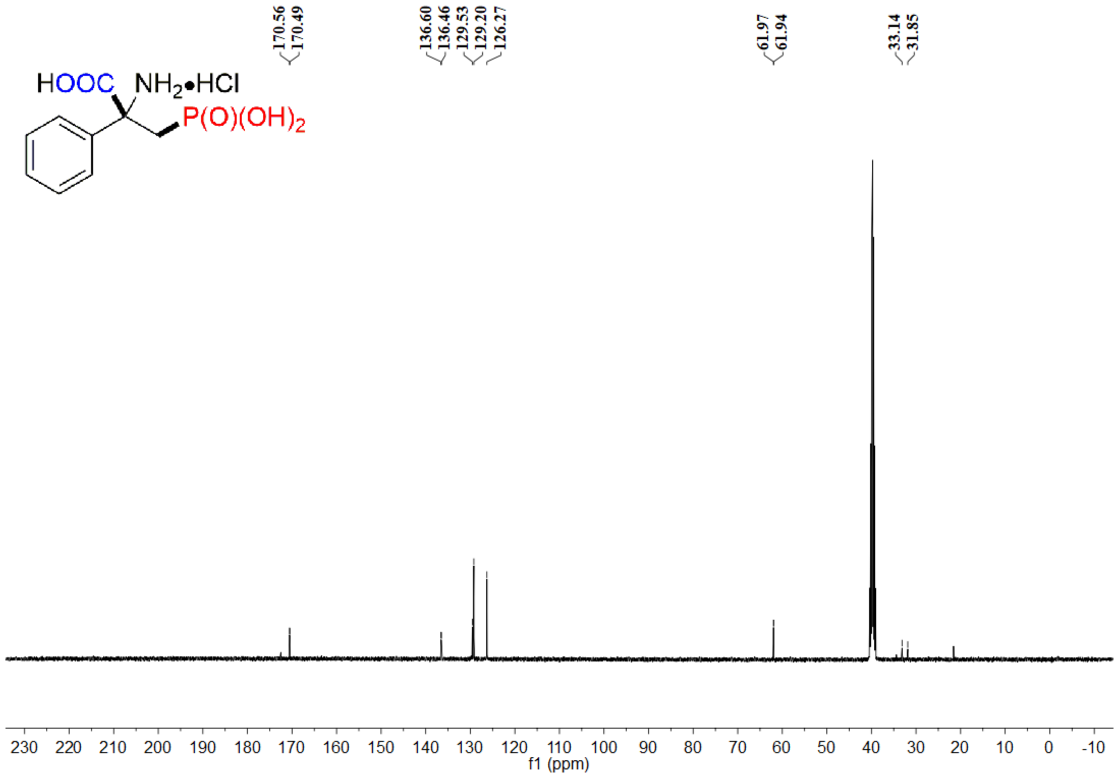

Supplementary Figure 247. ^{13}C NMR spectra of **13**

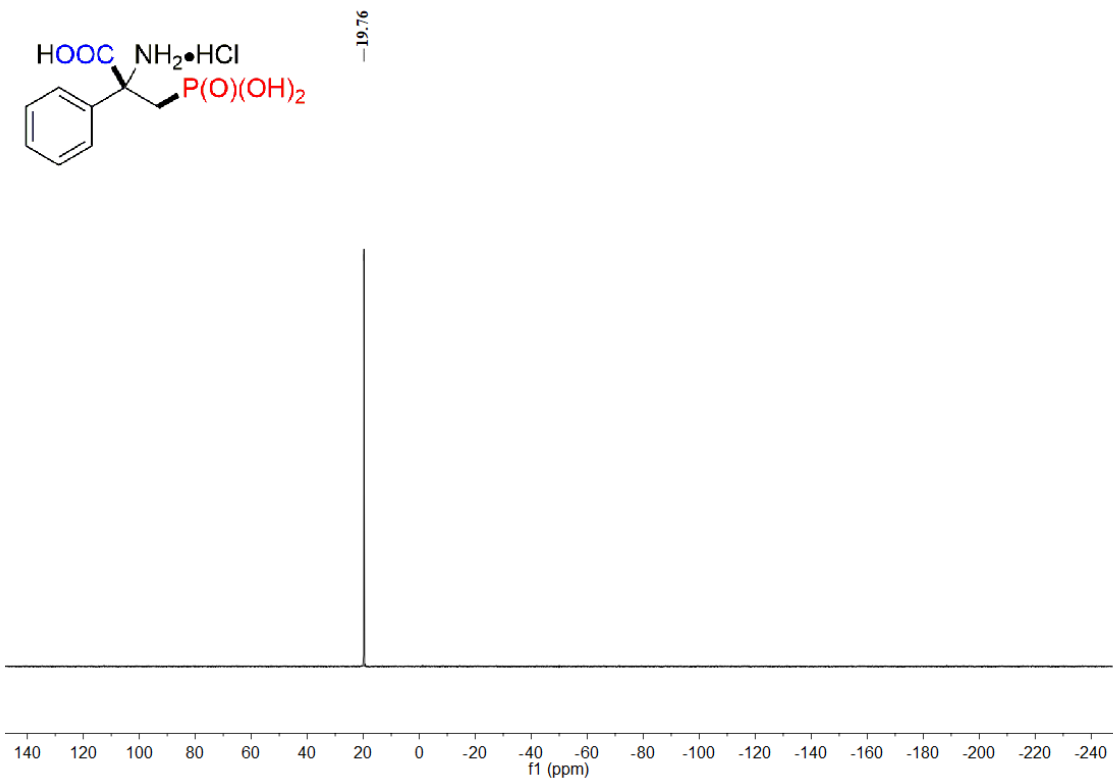

Supplementary Figure 248. ^{31}P NMR spectra of **13**

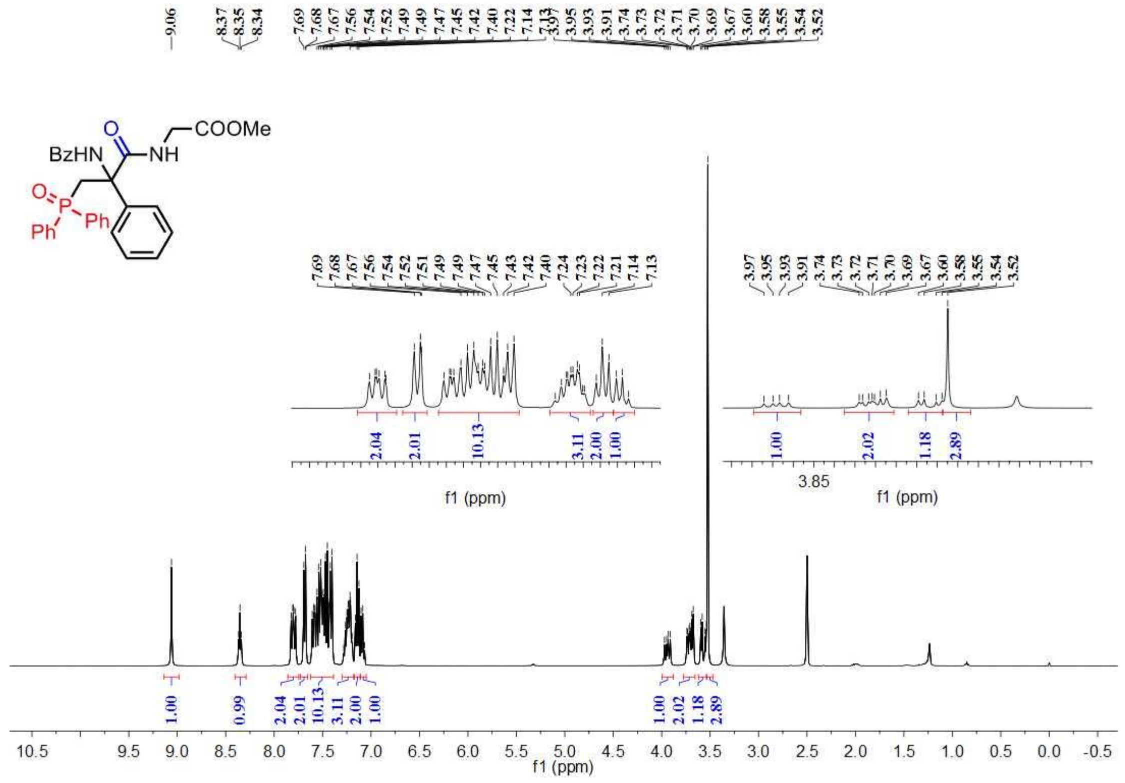

Supplementary Figure 249. ^{1}H NMR spectra of **14**

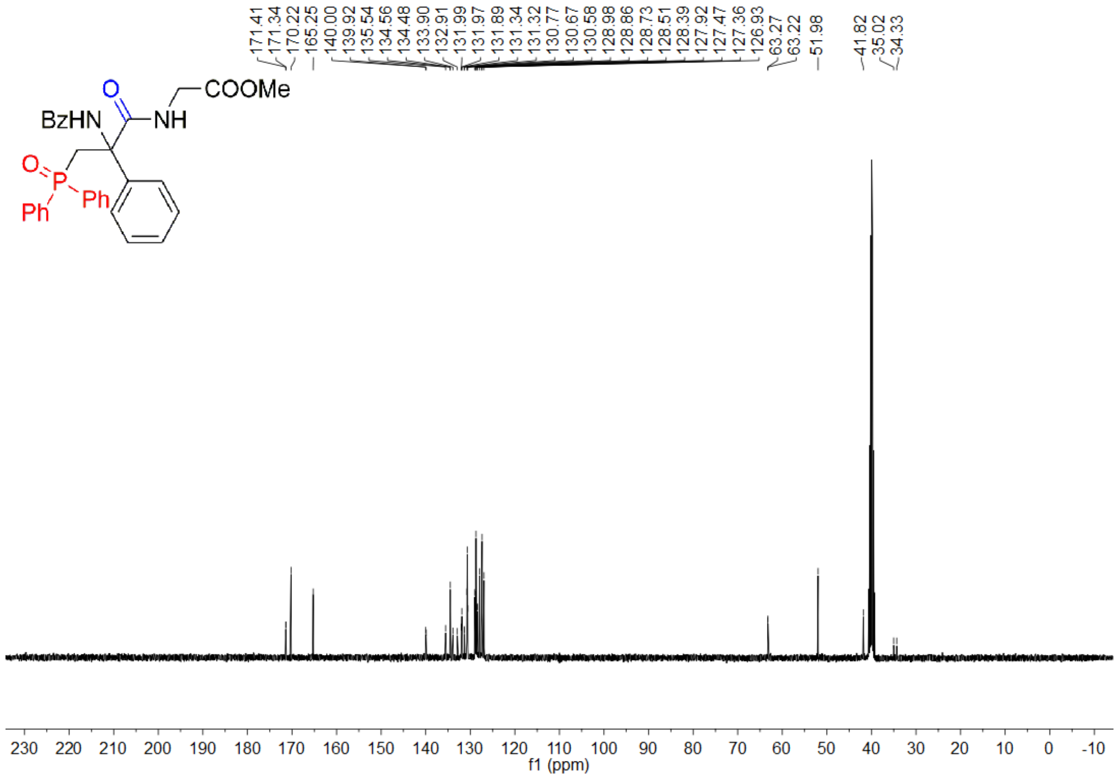

Supplementary Figure 250. ^{13}C NMR spectra of **14**

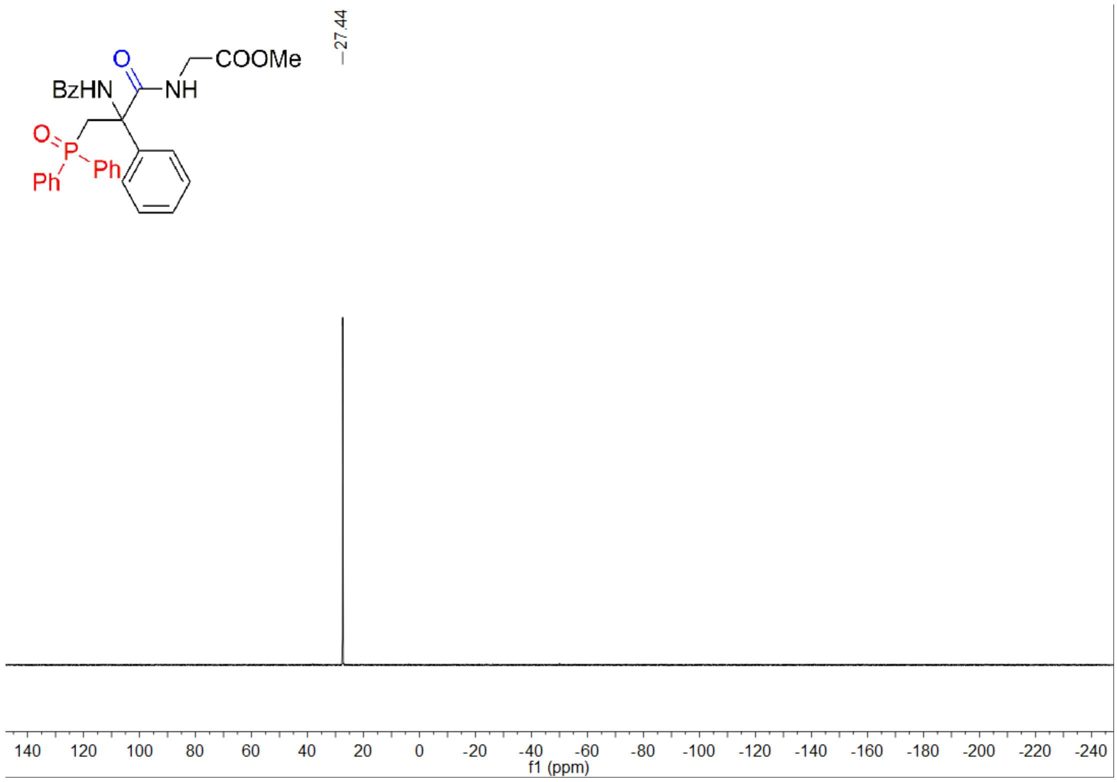

Supplementary Figure 251. ^{31}P NMR spectra of **14**

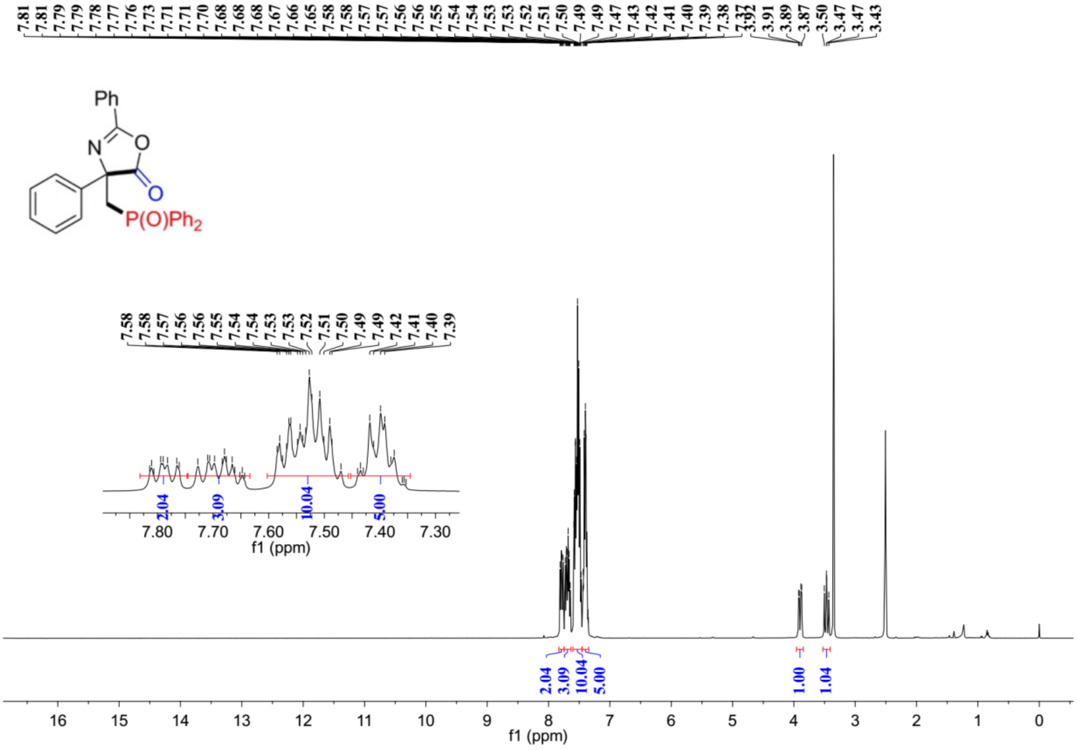

Supplementary Figure 252. ^{1}H NMR spectra of **15**

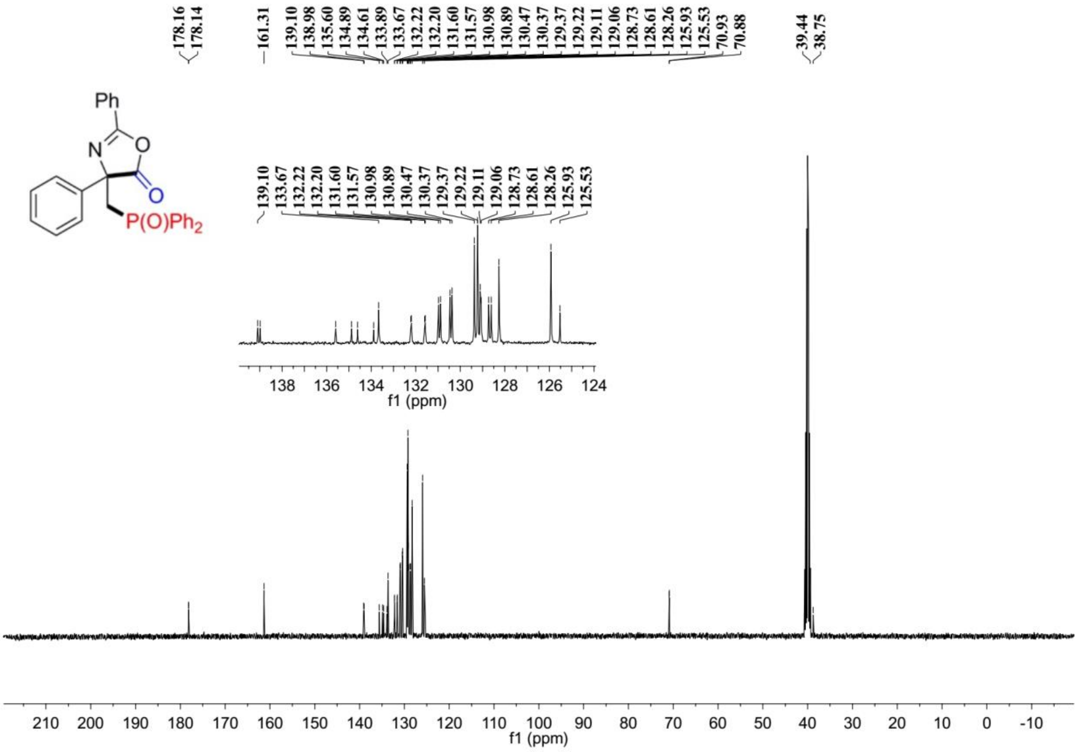

Supplementary Figure 253. ^{13}C NMR spectra of **15**

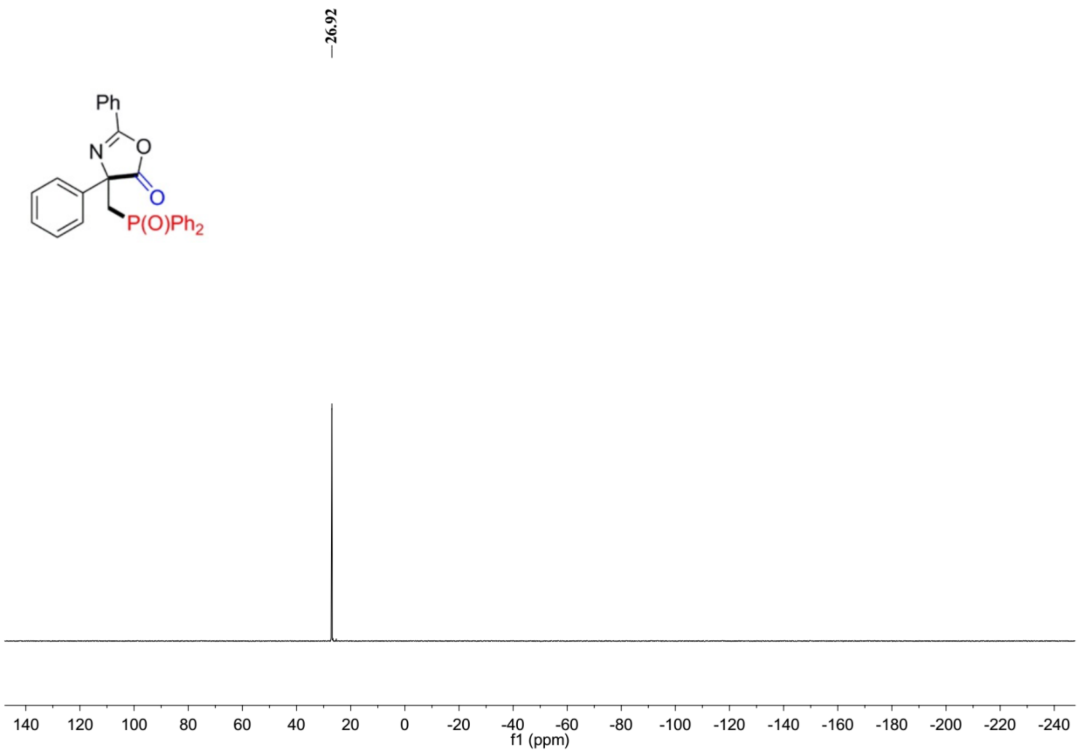

Supplementary Figure 254. ^{31}P NMR spectra of **15**

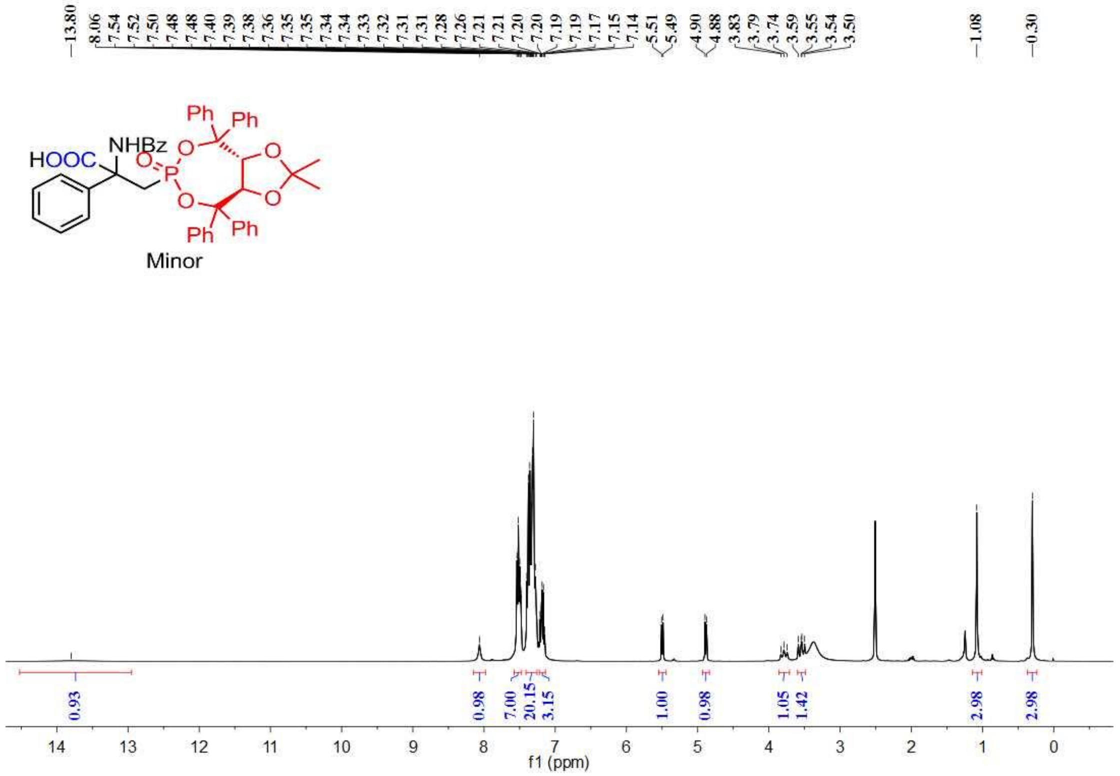

Supplementary Figure 255. ^{1}H NMR spectra of **3am-minor**

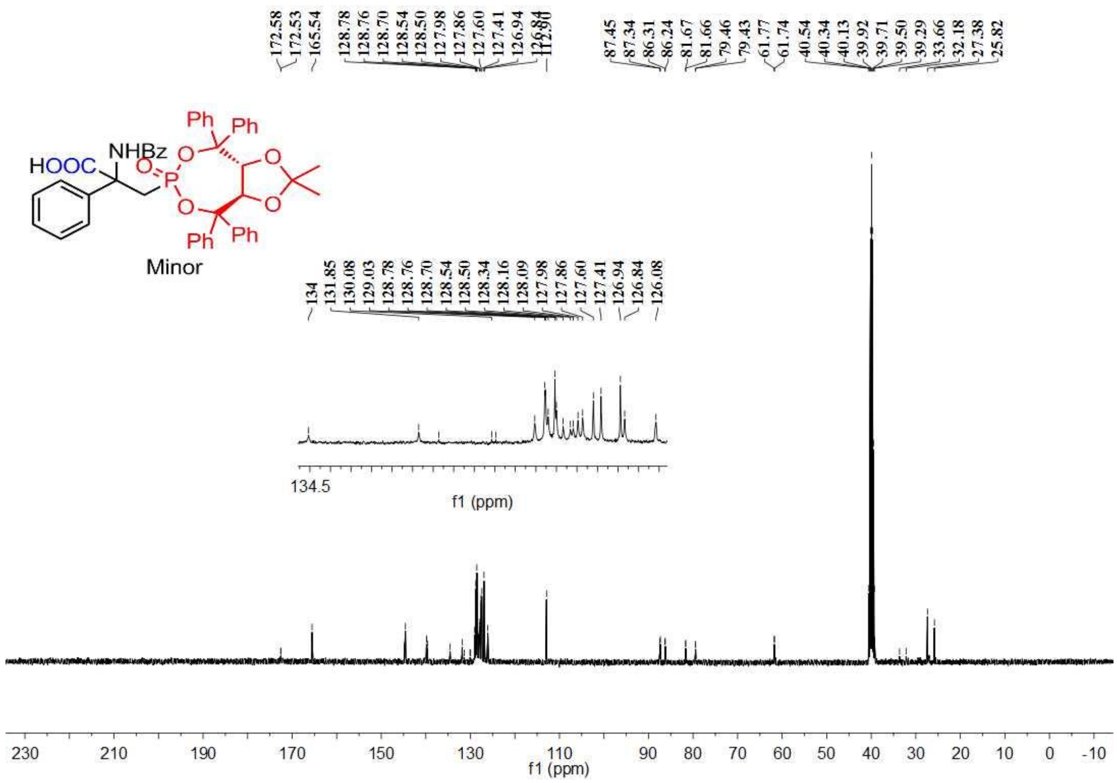

Supplementary Figure 256. ^{13}C NMR spectra of **3am-minor**

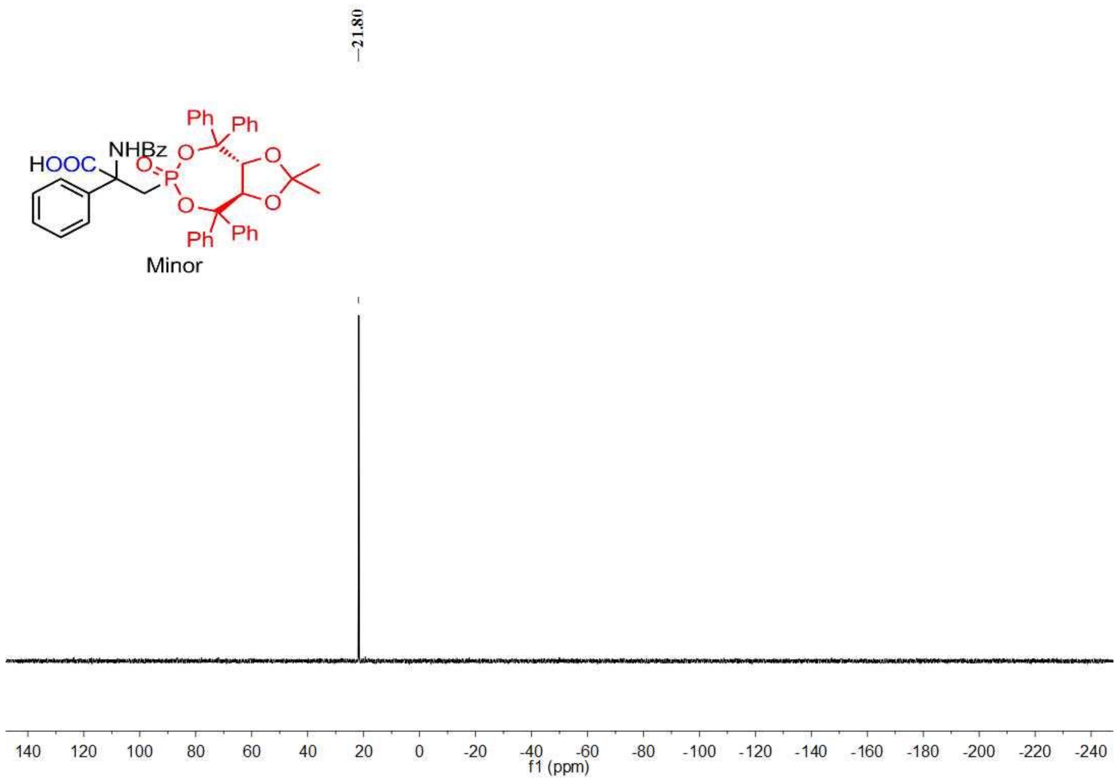

Supplementary Figure 257. ^{31}P NMR spectra of **3am-minor**

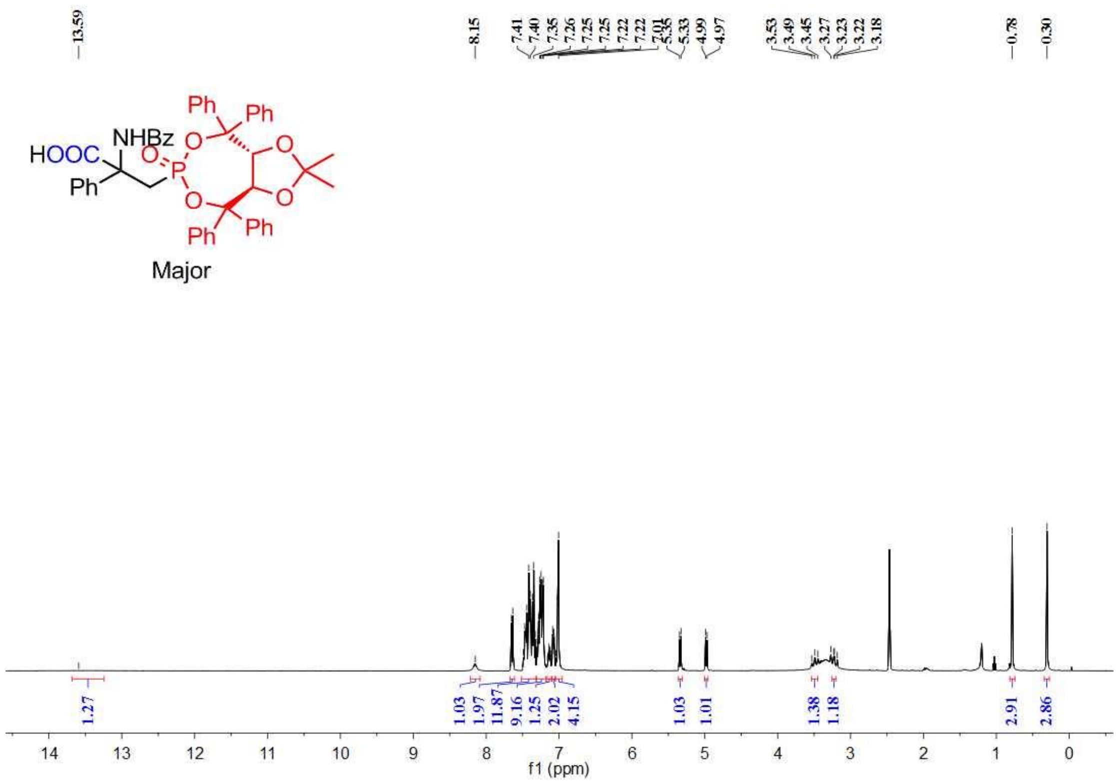

Supplementary Figure 258. ^{1}H NMR spectra of **3am-major**

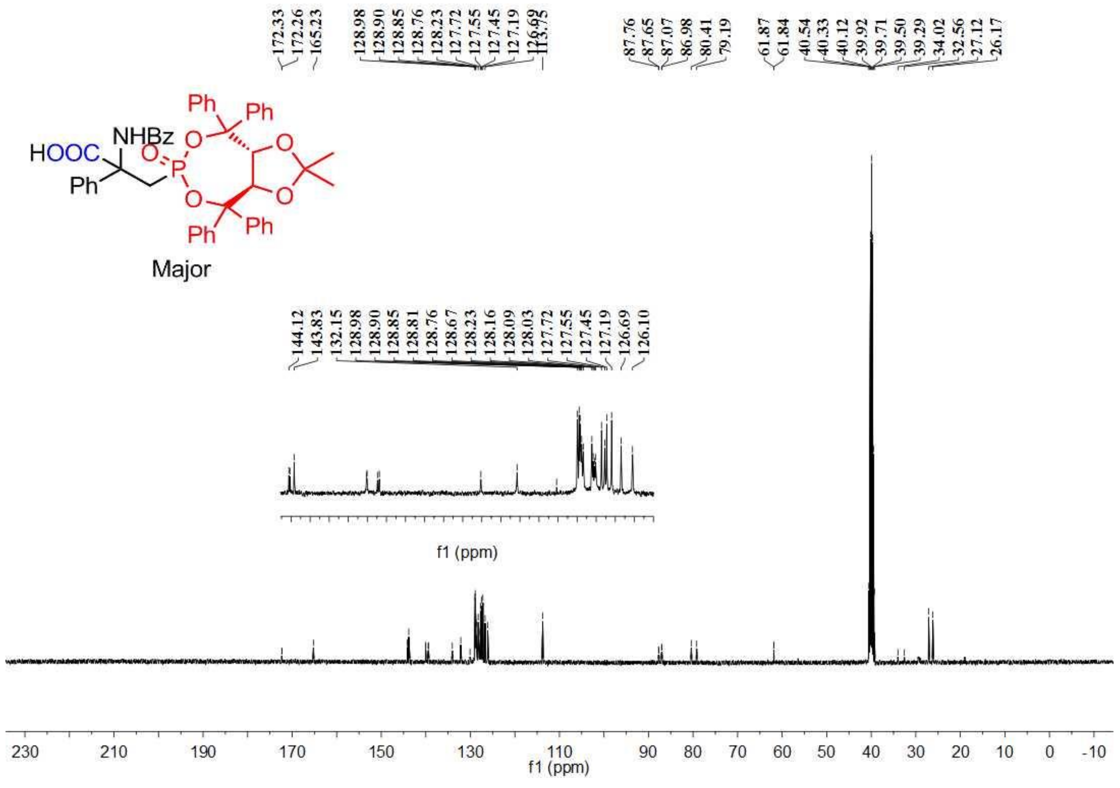

Supplementary Figure 259. ^{13}C NMR spectra of **3am-major**

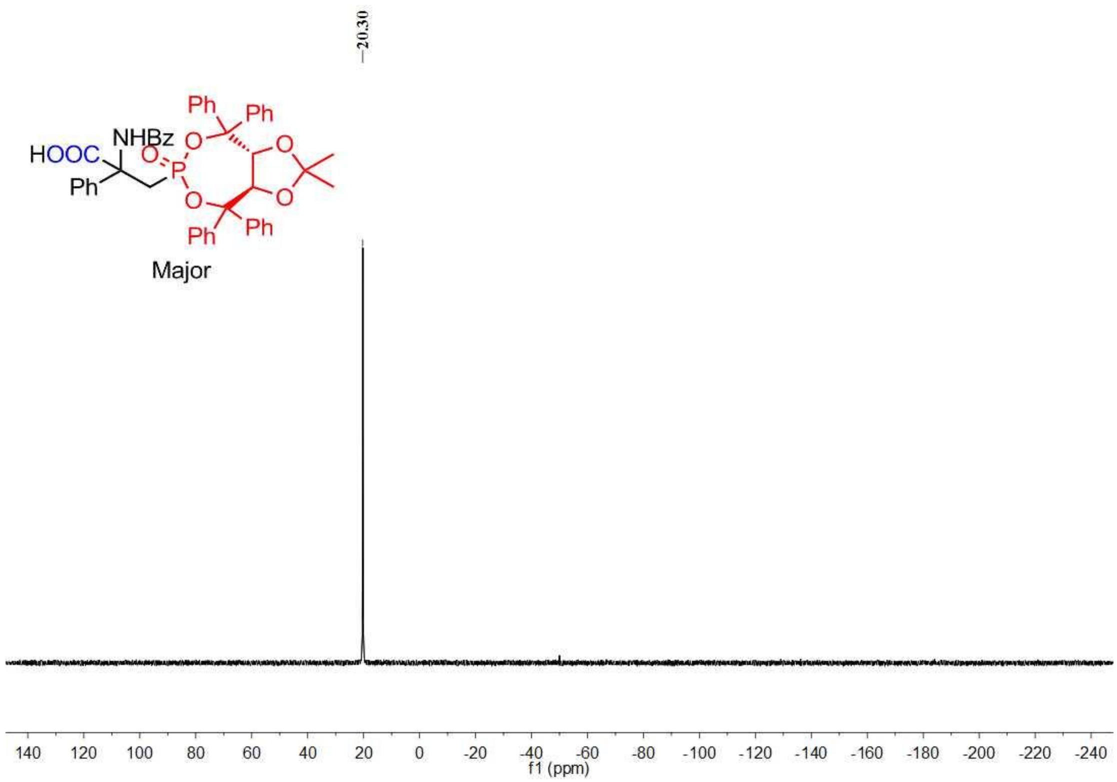

Supplementary Figure 260. ^{31}P NMR spectra of **3am-major**

Supplementary References

1. Guan, Z.-H., Zhang, Z.-Y., Ren, Z.-H., Wang, Y.-Y. & Zhang, X. Synthesis of enamides via CuI-catalyzed reductive acylation of ketoximes with Na_2SO_3. *J. Org. Chem.* **76**, 339-341 (2011).
2. Hu, N. et al. Synthesis of chiral α-amino tertiary boronic esters by enantioselective hydroboration of α-arylenamides. *J. Am. Chem. Soc.* **137**, 6746-6749 (2015).
3. Zhang, Z. et al. Lactonization of C(sp^2)-H bonds in enamides with CO_2. *Chin. J. Chem* . **36**, 430-436 (2018).
4. Ke, J. et al. Copper-catalyzed radical/radical $C_{sp}{}^3$-H/P-H cross-coupling: α-phosphorylation of aryl ketone *o*-acetyloximes. *Angew. Chem. Int. Ed.* **54**, 6604-6607 (2015).
